# The role of trauma and positive youth development in polysubstance use among rural middle school students: a latent class analysis

**DOI:** 10.1186/s12889-022-14795-1

**Published:** 2022-12-14

**Authors:** Andrew P. Zervos, Devon J. Hensel, Rebecca James, Abby Hunt, Mary A. Ott

**Affiliations:** 1 Department of Pediatrics, Section of Adolescent Medicine, Indiana University School of Medicine, 410 W. 10thStreet, Suite 1001, Indianapolis, IN 46202 USA; 2 Health Care Education and Training, Inc, 1630 N. Meridian Street, Suite 430, Indianapolis, IN 46202 USA

**Keywords:** Polysubstance use, Substance use, Rural, Middle school, Trauma, Connectedness

## Abstract

**Background:**

Rural youth often begin developing polysubstance use and other risk behaviors during middle school. However, little polysubstance use research focuses on rural middle school youth. Our research uses Latent Class Analysis to understand existing patterns of rural middle school polysubstance use and risk and protective factors associated with polysubstance use.

**Methods:**

We used survey data from a rural middle school pregnancy prevention program (*N* = 2,708). The survey included measures of demographics, lifetime substance use, trauma (adverse childhood experiences and bullying victimization) and aspects of youth development (parent communication on drugs and alcohol, parent connectedness and school connectedness). We used latent class analysis to produce participant polysubstance use profiles and multinomial regression to examine associations between polysubstance use, demographics, trauma and aspects of youth development.

**Results:**

We categorized our participants into four latent classes. Our analysis classified 2.2% of participants as Regular Polysubstance users, 6.9% as Polysubstance experimenters, 19% as Vape + Alcohol experimenters and 71.9% as Non-Users. More adverse childhood experiences were associated with greater risk of polysubstance use and experimentation. Bullying was positively associated with greater risk of vape and alcohol experimentation. Higher reported parental and school connectedness were associated with lower risk of high frequency polysubstance use. Higher reported school connection was also associated with lower risk of polysubstance experimentation.

**Conclusion:**

Rural substance use prevention programs should begin during middle school, as polysubstance use development is common among rural middle schoolers. These programs should be trauma informed and focus on connectedness as a modifiable factor to reduce risk of polysubstance use development.

**Trial registration:**

This article does not report results of a health care intervention on human participants.

**Supplementary Information:**

The online version contains supplementary material available at 10.1186/s12889-022-14795-1.

## Background

The purpose of this paper is to characterize existing patterns of rural middle school youth polysubstance use – use of multiple substances concurrently—and identify risk and protective factors associated with rural polysubstance use, with the goal of better informing substance use prevention programs. Middle school is a developmentally important time for substance use prevention. Substance use often initiates during middle school, as youth adopt and experiment with risk behaviors [1]. Young adults with substance use disorders frequently report they initiated substance experimentation and developed patterns of ongoing substance use during middle school years [2, 3]. Also important to prevention, it is during middle school that young people develop positive social skills and adopt protective behaviors, such as open communication with parents, parent connectedness, and school connectedness [4, 5].

Youth often begin experimenting with substances at middle school ages [1]. Currently, one in four U.S. 8^th^ graders report using alcohol, a similar number vape, nearly one in seven report using marijuana, one in eight report using tobacco cigarettes and approximately one in five report using other illicit substances [6]. Rural middle school youth are at particular risk for substance use development. Rural youth often experience more social isolation and have different access to substances, as compared to urban youth, which may lead to patterns of substance use development specific to their population. Compared to urban and suburban youth, rural middle school youth report higher rates of alcohol, tobacco and marijuana use [7]. Most research with rural youth focuses on a single substance, and little research examines patterns of polysubstance use development among rural youth. Understanding rural youth polysubstance use is important, as youth substance use initiation is rarely isolated to single substance use over time [8].

Polysubstance use emerges in various patterns across middle school youth. For example, some youth are more likely to experiment with only a small number of different substances during middle school, while others may develop a proclivity for regularly using a wide range of substances by this time. This places importance on examining multiple groups or clusters of polysubstance use behaviors across rural youth. Prior research has demonstrated the utility of Latent Class Analysis (LCA) for identifying the most likely patterns of polysubstance use among different youth populations [9–12]. LCA is a person-centered statistical approach, which assigns respondents to unobserved latent classes based on patterns within their responses. For example, Evans-Police et al., used LCA when researching tobacco, alcohol and other substance use among college students to identify latent classes such as “Non/Low Users”, “Polysubstance Users”, etc. [13]. We use a similar LCA approach in this work to group rural youth into the most likely classes based on patterns of lifetime polysubstance use behaviors.

Prior research has demonstrated that risk and protective factors play an important role in rural youth polysubstance use development. Among all youth, trauma, such as bullying victimization and adverse childhood experiences (ACEs), are associated with increased risk of overall substance use [14, 15], while protective factors, such as school and parental connectedness, often are associated with lower youth substance use [4, 5]. The influence of both trauma and protective factors on polysubstance use development is important to examine, as emphasizing these factors in adolescent prevention efforts may reduce or slow rates of polysubstance use development in early adulthood. Substance use prevention programs built on positive youth development principles incorporate these types of modifiable protective factors. However, it is unclear whether common risk and protective factors outlined in prior research maintain the same relationships to polysubstance use in populations of rural youth. Little research has examined accepted youth risk and protective factors (e.g. trauma, connectedness) in relation to how they influence rural youth polysubstance use specifically. Analyzing these factors in rural youth populations can better inform rural prevention programs, as they may display unique associations to substance use development.

Accordingly, the focus of this research was to understand existing patterns of polysubstance use among rural middle schoolers using Latent Class Analysis (LCA); and to examine relationships between demographic characteristics, experiences with trauma, positive youth developmental factors and patterns of polysubstance use. We aimed to expand on past research of rural substance use by analyzing multiple concurrent substance use rather than single substance use, and by testing relationships between common risk/protective factors to see if demonstrated relationships from other youth populations are maintained in a rural youth population. We expected that increased experiences with trauma would be associated with higher odds of polysubstance use (relative to non-use), while increased positive youth developmental factors would be associated with lower odds of polysubstance use (relative to non-use) among rural middle schoolers.

## Methods

### Study design and sample

Predominantly white and Hispanic middle school students living in a rural Midwestern low-to-middle income county were recruited as part of an evidence-based pregnancy prevention program between 2016 and 2020 (Table 1; *N* = 2,708). Eligible participants were any student enrolled in health classes at the selected high schools between the years 2016 and 2020, who provided informed consent (2016, *N* = 672; 2017, *N* = 647; 2018, *N* = 688; 2019, *N* = 587; 2020, *N* = 114). Participants completed baseline paper surveys containing measures of youth risk and protective factors. Each student gave informed consent prior to the program start date, and parents of participants were given information on the survey and the ability to withdraw their child from the pregnancy prevention program at any time. Those who were withdrawn did not provide further surveys, however their already completed surveys were retained for analysis. Participants could take the survey up to three times, once in 6^th^ grade health class, once in 7^th^ grade health class, and once in 8^th^ grade health class.

**Table 1:** Participant characteristics among a sample of rural middle school students (*N* = 2078)

Characteristic	Overall n (%) or mean (SD)
**Gender**	
Female	1,352 (51.0)
Male	1,297 (49.0)
**Sexual Identity**	
Heterosexual	2,088 (89.4)
Sexual Minority	248 (10.6)
**Grade**	
6th	941 (35.0)
7th	904 (33.5)
8th	847 (31.5)
**Ethnicity**	
Non Hispanic or Latino	1,272 (48.6)
Hispanic or Latino	1,343 (51.4)
**Language Spoken at Home**	
Only English	1,126 (42.1)
Mostly English	312 (11.7)
English and another language equally	867 (32.4)
Mostly another language	275 (10.3)
Only another language	92 (3.4)
**Language Spoken With Friends**	
Only English	1,325 (49.8)
Mostly English	701 (26.4)
English and another language equally	568 (21.4)
Mostly another language	43 (1.6)
Only another language	21 (0.8)
**Parent Communication on Drugs and Alcohol**	
Never	511 (41.1)
Once or Twice	339 (27.3)
Many Times	393 (31.6)
**Adverse Childhood Experiences (Range: 0—8)**	1.76 (1.89)
**Bullying (Range: 0—24)**	4.22 (4.59)
**Parental Connectedness (0—20)**	16.56 (4.89)
**School Connectedness (0—20)**	14.36 (3.21)
**Vape Use (lifetime)**	
Never	2,053 (77.2)
1 to 9 times	441 (16.6)
10 or more times	165 (6.2)
**Tobacco Use (lifetime)**	
Never	2,339 (87.2)
1 to 9 times	268 (9.9)
10 or more times	74 (2.9)
**Alcohol Use (lifetime)**	
Never	1,734 (65.0)
1 to 9 times	766 (28.8)
10 or more times	165 (6.2)
**Marijuana Use (lifetime)**	
Never	2,402 (89.8)
1 to 9 times	159 (6.0)
10 or more times	115 (4.2)
**Synthetic Marijuana Use (lifetime)**	
Never	2,552 (95.9)
1 to 9 times	69 (2.6)
10 or more times	41 (1.5)
**Prescription Drug Use (lifetime)**	
Never	2,493 (93.1)
1 to 9 times	155 (5.9)
10 or more times	30 (1.0)
**Inhalant Use (lifetime)**	
Never	2,468 (92.2)
1 to 9 times	174 (6.5)
10 or more times	35 (1.3)
**Injected Illegal Drugs (lifetime)**	
Never	2,652 (99.0)
1 to 9 times	19 (0.7)
10 or more times	8 (0.3)

*SD* Standard deviation

To maintain youth confidentiality and to adhere to state mandated reporting laws about early adolescent sexual behavior, surveys were anonymous and were not linked across years. In lieu of identifying themselves on the survey, youth were provided with resources and safe spaces if they wanted to disclose. The Indiana University Institutional Review Board and the Community Schools of Frankfort School Board approved the prevention program. Our analysis used data from items measuring youth substance use, experiences with trauma, and protective factors.

### Measures

Eight variables measuring substance use were incorporated in our analysis. Each measure was adapted from the Center for Disease Control’s Youth Risk Behavior Survey. Respondents were asked to report lifetime substance use, “How often in your lifetime, have you.. “used and electronic vapor product,” tried any tobacco products,” had at least one drink of alcohol,” “used marijuana,” “used synthetic marijuana,” “taken prescription drugs without a prescription,” “sniffed glue, breathed contents of spray cans, or inhaled paints or sprays to get high,” and “used a needle to inject illegal drugs.” Responses for each item included “Never,” “1 or 2 times,” “3 to 9 times,” “10 to 19 times,” “20–39 times,” and “40 or more times.” Responses were recoded trichotomously during analysis to identify, “Never,” “1 to 9 times,” and “10 or more times.”

Demographic measures included: gender (male/female/gender minority) recoded as (“Male” or “Female”), sexual identity (heterosexual/sexual minority inclusive of gay, lesbian bisexual, questioning or unsure), grade and Hispanic/Latino ethnicity. We additionally measured language most often spoken at home and with friends (single five-point Likert-scale responses ranging from “Only English'' to “Only another language”). Measures of trauma included adverse childhood experiences (eight yes/no items, e.g., “have you ever lived with a parent or guardian that died?”) and bullying (six items total, five-point Likert-scale items, ɑ = 0.806; e.g., “How often in the PAST 3 MONTHS have you been excluded from a group or completely ignored?”). Measures of youth development included parent connectedness [16] (five items total, five-point Likert-scale items, ɑ = 0.927; e.g. “How loved do you feel by your parent(s) or guardian(s) who raised you?”), school connectedness [17] (five items total, five-point Likert-scale items, ɑ = 0.803; e.g., “I am happy to be at my school.”) and parent communication on drugs and alcohol (one item, three-point Likert-scale item, “How many times have you and a parent or guardian talked about drug or alcohol use?”, ranging from “Never” to “Many Times”).

### Statistical analysis

Since multiple observations from the same participant are correlated, all analyses used a robust standard error to adjust for participants who had completed the survey in multiple years. We used two statistical approaches to analyze the data. First we perform LCA to produce classes of rural youth polysubstance use. Second we use multinomial logistic regression analysis to examine how trauma and youth developmental factors affect latent class membership. LCA modeling was executed using MPlus version 7.1 and SPSS version 26 was used for all data management, descriptive analyses and multinomial logistic regression analyses.

Latent Class Analysis. We used LCA to group youth based on response patterns across observed substance use variables to produce unobserved youth substance use profiles. Each participant is assigned probabilities of each substance use behavior incorporated in the analysis and placed into the best fitting latent class in relation to their probabilities [18]. For further background on the LCA method and formulae used in this analysis, please see the following citations [19–21]. For our study, substance use profiles (latent classes) are based on probabilities across substances used and display underlying patterns of youth polysubstance use. LCA gave us the ability to compare relative heterogeneity and homogeneity of respondents according to their substance use profiles.

Our LCA began with determining the best class solution by evaluating across various statistical fit indices. These included: Akaike Information Criterion (AIC), Bayesian Information Criterion (BIC), Sample Size Adjusted Bayesian Information Criterion (SSBIC), Lo-Mendell-Rubin Likelihood ratio test (LMR) and entropy [19]. As is necessary with LCA, we also employed theoretical reasoning to evaluate LCA models [20]. We used an established substance use theory, as well as a logical evaluation to ensure that the selected solution showed logical relationships, contained relatively homogenous classes and retained classes with high enough proportions of the sample [21].

Multinomial Logistic Regression. Once the optimal number of classes was established, we used multinomial logistic regression to analyze relationships between demographics, trauma factors, youth development factors and latent class membership. Multinomial logistic regression allowed us to compare classes in relation to their associations with all included covariates. We conducted one model in which “Non-Users” were used as the reference category against the other three classes.

## Results

### Participants, trauma and youth development factors, and substance use behaviors

Participants were 49% male, 51% female, < 1% gender minority, and 35% were in 6^th^ grade, 34% 7^th^ grade, and 31% 8^th^grade (Table 1). Nearly half identified as Hispanic/Latino reflecting local population demographics, and over half reported speaking at least some Spanish at home or with friends. One in three respondents (32%) reported that they have talked with parents about drugs and alcohol “many times” in the past. The average number of adverse childhood experiences among our sample was 1.76 (sd = 1.89) experiences. Respondents on average scored 16.6 (sd = 4.9) out of 20.0 on the parent connectedness scale and 14.4 (sd = 3.2) out of 20 on the school connectedness scale, indicating moderate to high mean levels of parent and school connectedness.

A high proportion abstained from ever using electronic vapor products (77%), tobacco (87%), alcohol (65%) and marijuana (90%), synthetic marijuana (96%), prescription drugs (93%), inhalants (92%) or injected drugs (99%). The most commonly used substance among our sample was alcohol, with 29% using between one to nine times and 6% using ten or more times in their lives.

### Latent class analysis

Model Selection. Table 2 represents the five class solutions that we modeled, each containing a different number of total classes (size 2–6 class solutions). We first evaluated class solutions based on five statistical fit indices. We began by evaluating solutions using AIC, BIC and SSBIC values. The three, four and five class solutions retained the lowest values within the three indices. We then looked for low and non-significant Lo-Mendell-Rubin test values across solutions. The lowest non-significant values retained for the seven and eight class solutions. Finally we evaluated solutions for a high entropy value. The two and three class solutions retained the highest entropy, however the range of entropy values from the smallest value (four class solution) to the highest value (two class solution) was rather negligible. Based on evaluation across fit indices, as well as a logical assessment of within class probabilities and sample size, and reviewing substance use literature, we identified the four-class solution as the optimal solution for our analysis.

**Table 2:** Statistical fit indices – 2 through 8 latent classes

Class solution	AIC	BIC	SSBIC	LMR Chi-sq	Entropy
2	13964.248	14158.883	14054.032	3068.351***	0.900
3	13368.639	13663.541	13504.676	624.954***	0.870
4	13270.215	13665.383	13452.503	131.446*	0.832
5	13239.334	13734.769	13467.875	64.401	0.860
6	13222.481	13818.183	13497.274	52.156	0.843
7	13215.779	13911.747	13536.825	40.407	0.858
8	13220.463	14016.699	13587.762	30.516	0.845

*Chi-sq* Chi Square test value produced with LMR test; *p < 0.05, **p < 0.01, ***p < 0.001

Latent Classes. Table 3 displays within class sample proportions and probabilities of the four class solution. We assigned name labels to each class based on the distribution of probabilities within classes. Class 1 (2.2% of final sample) presented the highest probabilities of 10 or more lifetime uses of tobacco (0.559), vapes (0.863), alcohol (0.850), marijuana (0.975) and synthetic marijuana (0.617). Additionally, class 1 contained low probabilities of ever using prescription drugs without a prescription, inhalants or injecting drugs. This class was labeled “Regular Polysubstance users.” Class 2 (19% of final sample) contained the highest probabilities of 1 to 9 lifetime uses of alcohol (0.696) and vapes (0.551). This class also contained extremely low probabilities of ever using any of the other six substances measures. Class 2 was labeled “Vape + Alcohol experimenters.” class 3 (6.9% of final sample) presented high probabilities of 1 to 9 lifetime uses of tobacco (0.593), vapes (0.469), alcohol (0.619) and marijuana (0.598). Class 3 contained very low probabilities of using any of the other four substances measured. We labeled this class “Polysubstance experimenters.” Finally, Class 4 (71.9% of final sample) contained the highest probabilities of never using any of the eight substances measured. Class 4 was labeled “Non-Users.”

**Table 3:** Four class solution—class probabilities for substance use in rural middle school students

Substance	Frequency of Use	Class 1: Regular Polysubstance Users (2.23%)	Class 2: Vape + Alcohol Experimenters (18.98%)	Class 3: Polysubstance Experimenters (6.87%)	Class 4: Non-Users (71.92%)
Tobacco	Never	0.106	0.755	0.242	1.000
	1 to 9 times	0.335	0.234	0.593	0.000
	10 or more times	0.559	0.012	0.165	0.000
Vape	Never	0.019	0.408	0.071	0.983
	1 to 9 times	0.118	0.551	0.469	0.015
	10 or more times	0.863	0.041	0.460	0.001
Alcohol	Never	0.000	0.233	0.073	0.863
	1 to 9 times	0.150	0.696	0.619	0.130
	10 or more times	0.850	0.070	0.308	0.007
Marijuana	Never	0.000	0.908	0.195	0.997
	1 to 9 times	0.025	0.074	0.598	0.002
	10 or more times	0.975	0.019	0.207	0.001
Synthetic Mar	Never	0.251	0.991	0.693	0.999
	1 to 9 times	0.132	0.009	0.300	0.001
	10 or more times	0.617	0.000	0.007	0.000
RX Pills	Never	0.459	0.886	0.739	0.981
	1 to 9 times	0.285	0.107	0.224	0.018
	10 or more times	0.256	0.007	0.037	0.001
Inhalants	Never	0.599	0.836	0.710	0.982
	1 to 9 times	0.238	0.149	0.208	0.018
	10 or more times	0.162	0.015	0.082	0.000
Injections	Never	0.757	0.995	0.966	0.999
	1 to 9 times	0.136	0.005	0.034	0.000
	10 or more times	0.107	0.000	0.000	0.001

### Multinomial logistic regression of covariates and class membership

Tables 4 and 5, respectively, outline multinomial regression models analyzing predictive associations between demographic characteristics, risk and protective factors and polysubstance use classes of Regular Polysubstance Use compared to other use patterns and Non-Users compared to other use patterns.

**Table 4:** Predictors of Regular Polysubstance Use compared to other use patterns in a sample of rural middle school students

Predictors	% (N) or Mean (SD)	Non-Users	Vape and Alcohol Experimenter	Polysubstance Experimenters
		Relative Risk Ratios [RRR] (95% CI)		
Gender (female)	48.8 (441)	2.01 (0.88 – 4.55)	2.19 (0.95 – 5.06)	1.22 (0.51 – 2.91)
Sexual identity (sexual minority)	11.4 (98)	0.82 (0.31 – 2.15)	0.43 (0.15 – 1.18)	0.91 (0.32 – 2.63)
Grade				
6th (referent)	33.5 (289)	-	-	-
7th	34.9 (301)	0.26 (0.06 – 1.02)	0.38 (0.09 – 1.49)	0.61 (0.14 – 2.71)
8th	31.6 (272)	0.08 (0.02 – 0.31)***	0.15 (0.05 – 0.58)**	0.47 (0.11 – 1.98)
Ethnicity (Hispanic)	59.5 (513)	1.01 (0.37 – 2.76)	0.87 (0.32 – 1.37)	1.01 (0.33 – 3.06)
Mostly/all English spoken at home (yes)	49.7 (428)	3.12 (1.16 – 8.36)*	1.16 (0.42 – 3.17)	2.92 (0.93 – 9.21)
Mostly/all English spoken at with friends (yes)	72.5 (625)	1.22 (0.44 – 3.38)	1.08 (0.39 – 2.99)	0.92 (0.31 – 2.74)
ACEs	1.76 (1.88)	0.50 (0.39 – 0.63)***	0.63 (0.49 – 0.79)***	0.71 (0.58 – 0.90)***
Bullying	4.22 (.56)	0.98 (0.90 – 1.06)	1.03 (0.95 – 1.12)	1.00 (0.91 – 1.09)
Drug or alcohol communication with parents		0.48 (0.29 – 0.84)**	0.62 (0.36 – 1.08)	1.02 (0.58 – 1.82)
Never	36.9 (318)			
Once or Twice	29.5 (246)			
Many times	298 (34.6)			
Parent connectedness	16.5 (4.8)	1.11 (1.04 – 1.19)**	1.09 (1.02 – 1.25)**	1.08 (1.01 – 1.16)*
School Connectedness	14.4 (3.2)	1.21 (1.08 – 1.36)**	1.12 (1.01 – 1.25)*	1.04 (0.93 – 1.17)

^*^p < .05; **p < .01; ***p < .001*;* Note: Nagelkerke *R*^2^ = 0.259

**Table 5:** Predictors of Non-Use compared to other use patterns in a sample of rural middle school students

Predictors	% (N) or Mean (SD)	Vape and Alcohol Experimenter	Polysubstance Experimenters	Polysubstance Regular Users
		Relative Risk Ratios [RRR] (95% CI)		
Gender (female)	51.2 (539)	1.07 (0.74 – 1.55)	0.61 (0.35 – 1.04)	0.49 (0.21 – 1.13)
Sexual identity (sexual minority)	11.4 (98)	0.53 (0.28 – 1.01)	1.11 (0.52 – 2.40)	1.21 (0.46 – 3.19)
Grade				
6th (referent)	33.5 (289)	-	-	-
7th	34.9 (301)	1.45 (0.94 – 2.26)	2.33 (1.07 – 5.10)*	3.79 (0.97 – 14.7)
8th	31.6 (272)	1.83 (1.15 – 2.93)*	5.68 (2.65 – 12.17)***	11.92 (31.7 – 44.75)***
Ethnicity (Hispanic)	59.5 (513)	0.85 (0.46 – 1.59)	0.98 (0.41 – 2.34)	0.97 (0.36 – 2.64)
Mostly/all English spoken at home (yes)	50.1 (442)	0.37 (0.20 – 0.70)**	0.93 (0.38 – 2.26)	0.32 (0.12 – 0.85)*
Mostly/all English spoken at with friends (yes)	72.6 (640)	0.87 (0.57 – 1.34)	0.75 (0.37 – 1.51)	0.81 (0.29 – 2.25)
ACEs	1.76 (1.88)	1.26 (1.13 – 1.41)***	1.42 (1.22 – 1.65)***	1.99 (1.58 – 2.52)***
Bullying	4.22 (.56)	1.05 (1.01 – 1.10)*	1.02 (0,96 – 1.10)	1.02 (0.94 – 1.10)
Parent communication about alcohol and drugs		1.28 (1.03 – 1.59)*	2.10 (1.51 – 2.91)***	2.04 (1.18 – 3.41)*
Never	36.9 (318)			
Once or Twice	29.5 (246)			
Many times	298 (34.6)			
Parent connectedness	16.5 (4.8)	0.98 (0.94 – 1.02)	0.96 (0.91 – 1.26)	0.89 (0.83 – 0.96)**
School Connectedness	14.4 (3.2)	0.92 (0.87 – 0.97)**	0.85 (0.78 – 0.94)**	0.82 (0.73 – 0.91)**

^*^p < .05; **p < .01; ***p < .001*;* Note: Nagelkerke *R*^2^ = 0.316

Demographics. Gender and sexual identity were not associated with polysubstance use. Youth in higher grade levels were significantly more likely to be Polysubstance regular users, Polysubstance experimenters and Alcohol + Vape experimenters as compared to being Abstainers. Additionally, youth in higher grade levels were significantly more likely to be Polysubstance regular users and Polysubstance experimenters as compared to being Alcohol + Vape experimenters. Language acculturation with friends was not associated with latent class membership. However, less English spoken at home was associated with higher likelihood of being an Alcohol + Vape experimenter as compared to being a Non-User.

Trauma and Youth Development Factors. Higher cumulative ACEs were significantly associated with greater likelihood of regular polysubstance use. For each additional ACE experienced by a participant, the likelihood of being a Polysubstance regular user increased by 37.4% compared to being a Polysubstance experimenter, 56.8% compared to being an Alcohol + Vape experimenter and 97% compared to being a Non-User. Compared to being an Non-User, each additional ACE increased the likelihood of being a Polysubstance experimenter by 43.4% and the likelihood of being an Alcohol + Vape experimenter by 25.6%. Youth who experienced more bullying and victimization were significantly more likely to be an Alcohol + Vape experimenter as compared to being a Non-User.

More parental communication about drugs and alcohol was associated with higher odds of being a Polysubstance experimenter as compared to being an Alcohol + Vape experimenter and an Abstainer. Additionally, more parental communication about drugs and alcohol was associated with higher odds of being a Polysubstance regular user and an Alcohol + Vape experimenter as compared to being a Non-User. Finally, greater school connectedness was associated with significantly lower odds of being a Polysubstance regular user, Polysubstance experimenter and an Alcohol + Vape experimenter as compared to being a Non-User. More school connection was also associated with greater odds of being a Polysubstance regular user compared to being an Alcohol + Vape experimenter.

## Discussion

We identified four latent classes which describe patterns of lifetime polysubstance use among rural middle school students. Lifetime use of multiple substances was common. Few middle schoolers were assigned to latent classes based on regular polysubstance use (more than 9 lifetime uses per substance). Yet, nearly one in four middle schoolers were assigned to latent classes characterized by polysubstance use experimentation (between 1 and 9 lifetime uses per substance). These results are consistent with the prevalence of single substance use in the overall middle school population [6] and suggest that initiation of multiple substance use in rural youth is common during middle school years. This finding demonstrates the importance of future rural substance use prevention programs beginning during middle school, as it is a time youth begin developing polysubstance use behaviors. While rural substance use prevention programs often primarily focus on tobacco use, our results show that a substantial proportion of rural youth begin experimenting with a range of substances during middle school (e.g. vapes, marijuana and alcohol). Based on this finding, prevention programs should be comprehensive in targeting a wide range substances with which rural middle schoolers commonly experiment.

Relationships between demographic predictors and rural middle school polysubstance use varied in our results. While gender and sexual identity have been found to be associated with polysubstance use during adolescence [22, 23], these associations did not exist among rural middle schoolers. A possible explanation for this difference is that gender and sexual identity is that the frequency of participants identifying as gender or sexual minorities was relatively low, which may have resulted in statistical power too small to produce small or moderate size effects. Positive associations between polysubstance use and grade or age are shown in prior research [9] and are expected as risk of substance use initiation and routinization increase during later stages of adolescence [3, 24]. Less family language acculturation (less English spoken at home) was associated with higher probabilities of alcohol and vape experimentation. Prior research has demonstrated associations between linguistic acculturation and substance use behaviors and attitudes [25–27]. However, these processes have rarely been examined in rural middle school youth. More research is needed to better understand how linguistic acculturation is associated with polysubstance use in rural youth.

As hypothesized, childhood experiences with trauma were positively associated with higher levels of polysubstance use initiation and routinization among rural youth. Rural middle schoolers’ risk for regular polysubstance use and experimentation increased with each additional ACE reported. This finding extends on past research which has shown similar associations between single substance use and traumatic experiences in the overall youth population [14, 15]. Trauma has a negative effect on overall youth development and mental health, and has been shown to promote risk behaviors throughout adolescence [28]. Youth who experience ACEs and other significant trauma frequently begin using substances as a coping strategy and have often been exposed to family substance use which can lead to youth repeating family substance use patterns [29]. As middle school is a time characterized by the initiation of many common risk behaviors, including polysubstance use, it is important that rural middle school prevention programs are well informed on the effects of past youth trauma.

Contrary to ACEs, experiences with bullying were only positively associated with greater odds of polysubstance use experimentation, rather than polysubstance use routinization. However, the effect that experiences of bullying had on polysubstance use experimentation was small. It is possible that bullying may contribute less of an impact on polysubstance use development during middle school years than at later stages of rural adolescence (e.g. high school). More research is needed to understand the relationship between experiences with bullying and initial experimentation of multiple substances among rural youth.

Our findings suggest that rural middle school prevention programs can target connectedness as a modifiable protective factor against polysubstance use development. Rural middle schoolers with greater reported school connectedness were at substantially lower risk for both high frequency polysubstance use and polysubstance use experimentation, while those with greater parental connectedness were also at lower risk for high frequency polysubstance use. Connectedness is a strong protective factor against risk in early adolescent development. Youth who perceive greater positive connection to their parents and school environment are more likely to delay adopting risk behaviors or never engage in some risky behaviors at all [13]. Prevention programs can promote connectedness through school and family oriented strategies, such as encouraging extracurricular activity participation (e.g. school clubs) [30], creating a safe and supportive learning environment [31], increasing parent-youth communication and support and providing positive mentor–mentee relationships [32]. Future rural middle school prevention programs should develop strategies to target protective factors like connectedness which can be modified, rather giving primary emphasis to unmodifiable factors such as past trauma.

Our data was limited to retrospective self-reports of lifetime substance use. Additionally, using only lifetime measures meant that we did not have the ability to analyze the amount of time between instances of use or how recently substances had been used. We did not implement any survey items to measure access to substances. Our primary analytical method, LCA, carries some inherent limitations. Researchers are responsible for evaluating LCA models with a degree of subjectivity. While we used logical reasoning and established research to make our final model decisions, researcher bias could have been introduced.

## Conclusion

The development of polysubstance use is common during middle school among rural youth. Rural youth substance use prevention programs should begin during middle school, as this is commonly a time for initiating polysubstance use behaviors. These programs should be trauma informed, as experiences with trauma among rural youth are significantly related to higher frequency polysubstance use. Modifiable protective factors, namely connectedness, should be the primary focus of future rural substance use interventions.

## Supplementary Information

**Additional file 1.**

**Additional file 2.**

## Data Availability

All data generated or analyzed during this study are included in this published article [and its supplementary information files].

## Abbreviations

LCA: Latent Class Analysis
ACEs: Adverse Childhood Experiences
AIC: Akaike Information Criterion
BIC: Bayesian Information Criterion
SSBIC: Sample Size Adjusted Bayesian Information Criterion
LMR: Lo-Mendell-Rubin Likelihood Ratio Test
