# Supplementary material for: The role of trauma and positive youth development in polysubstance use among rural middle school students: a latent class analysis
Source: BMC Public Health. 2022 Dec 14;22:2350. doi: 10.1186/s12889-022-14795-1 (PMC9753425; doi:10.1186/s12889-022-14795-1)
Supplement: Supplementary file 1 — Additional file 1. [file 12889_2022_14795_MOESM1_ESM.docx]

| General Instructions |
| --- |

Thank you for your help with this evaluation! The information you provide will help us improve the program. We want you to know that:

1. This survey is completely anonymous. You will not provide your name on the survey.

2. All of your answers will be kept private and will not be shared with anyone outside of the evaluation team.

3. We hope you will answer all of the questions, but you may choose not to answer any question.

4. Your participation is voluntary. We hope you agree that the information you provide is very important, but you can choose to stop participating at any time.

| **PLEASE READ EACH QUESTION CAREFULLY: There are different ways to answer the questions in this survey. It is important that you follow the instructions when answering each kind of question.**   - **PLEASE MARK ALL ANSWERS WITHIN THE WHITE BOXES PROVIDED.** - **USE A PEN OR PENCIL.** |
| --- |

**Please write in the first two letters of your middle name.**

**For instance, if your middle name is John, you write JO, if your middle name is Marie, you would write MA**

First letter ___ __ Second letter______

**Next, please circle the month and date of your birth.**

**For instance, if you were born on July 21, you would circle JULY and 21**

JANUARY FEBRUARY MARCH APRIL MAY JUNE JULY AUGUST SEPTEMBER

OCTOBER NOVEMBER DECEMBER

1 2 3 4 5 6 7 8 9 10 11 12 13 14 15 16 17 18

19 20 21 22 23 24 25 26 27 28 29 30 31

**section 1: about you**

**1. How old are you?**

MARK ONE ANSWER WITH AN X

□ 10 or younger □ 11 □ 12 □ 13 □ 14 □ 15 □ 16

**2. What grade are you in?** (If you are on vacation between grades, please indicate the grade you will be in when you go back to school.) MARK ONE ANSWER WITH AN X

□ 6^th^ □ 7^th^ □ 8^th^

**3. Are you male or female?**

MARK ONE ANSWER WITH AN X

□ Male □ Female □ Transgender □ Do not identify as male, female, or transgender

□ Unsure

**4. Do you consider yourself to be one or more of the following?**

MARK (X) ALL THAT APPLY

□ Straight □ Lesbian □ Gay □ Bisexual □ Unsure

□ I’m uncomfortable answering this question

**5. Are you Hispanic or Latino?**

MARK ONE ANSWER WITH AN X

□ Yes

□ No

**6. What is your race?**

MARK (X) ALL THAT APPLY

□ Native American or American Indian or Alaska Native

□ Native Hawaiian or Other Pacific Islander

□ Asian or South Asian

□ White

□ Black or African-American

□ Other (please specify)_________________

**7. Which answer best describes who you are currently living with most of the time:**

MARK ONE ANSWER WITH AN X

□ With both of my parents

□ With my mother only

□ With my father only

□ With one of my parents and a step-parent or my parent’s boyfriend or girlfriend

□ With a grandparent(s) or other family member, such as an aunt or uncle

□ With a legal guardian (that is not a family member)

□ Other (please specify)_____________________________________________________________

**8. Does your family own a car, van, or truck?** □ No □ Yes, one □ Yes, two or more

**9. Do you have a bedroom for yourself?** □ No □ Yes

**10. How many computers does your family own?** □ None □ One □ Two □ More than two

**11. During the past 12 months, how many times did you travel away on holiday (vacation) with your family?**

□ Not at all □ Once □ Twice □ More than twice

**12. Have you ever experienced any of the following?**

|  | No | Yes |
| --- | --- | --- |
| a. lived with a parent that got divorced. | □ | □ |
| b. lived with a parent or guardian that died. | □ | □ |
| c. lived with a parent or guardian that served time in jail or prison. | □ | □ |
| d. lived with anyone with a mental illness, was suicidal, or severely depressed for more than a couple of weeks. | □ | □ |
| e. lived with anyone that had a problem with drugs or alcohol. | □ | □ |
| f. witnessed a parent, guardian, or other adult in the household behaving violently toward another person (such as slapping, hitting, kicking, punching, or beating each other up). | □ | □ |
| g. was the victim of violence or witnessed violence in your neighborhood. | □ | □ |
| h. frequent or often economic hardship (such as your family had a hard time affording food or housing). | □ | □ |

**13. When you are at home or with your family, what language or languages do you usually speak?**

MARK (X) ALL THAT APPLY

□ English

□ Spanish

□ Chinese language such as Mandarin or Cantonese

□ Some other language: _______________________

**14. These questions ask you about what language you commonly speak…**

|  | Only English | Mostly English | English and another language equally | Mostly another language | Only another language |
| --- | --- | --- | --- | --- | --- |
| a. in your home, do you speak… | □ | □ | □ | □ | □ |
| b. with your friends, do you speak… | □ | □ | □ | □ | □ |

**SECTION 2: ACTIONS AND BEHAVIORS**

**The next questions ask about your actions, behaviors, sexual intercourse and your risk of pregnancy and sexually transmitted diseases. *Some teens call sexual intercourse ’vaginal sex’ or ‘regular sex ‘.* Questions on this survey mean behaviors that you choose to participate in—do not count behaviors you were forced to do against your will.**

**15. How often in in your lifetime, have you:**

|  | Never | 1 or 2 times | 3 to 9 times | 10 to 19 times | 20 to 39 times | 40 or more times |
| --- | --- | --- | --- | --- | --- | --- |
| a. tried any tobacco products (such as cigarettes, cigars, chewing tobacco, dip)? | □ | □ | □ | □ | □ | □ |
| b. used an electronic vapor product, such as e-cigarettes, e-cigars, e-pipes, vape pipes, vaping pens, ehookahs, and/or hookah pens? | □ | □ | □ | □ | □ | □ |
| c. had at least one drink of alcohol? | □ | □ | □ | □ | □ | □ |
| d. used marijuana? | □ | □ | □ | □ | □ | □ |
| e. used synthetic marijuana (also called K2, Spice, fake weed, King Kong, Yucatan Fire, Skunk, or Moon Rocks)? | □ | □ | □ | □ | □ | □ |
| f. taken a prescription drug (such as OxyContin, Percocet, Vicodin, codeine, Adderall, Ritalin, or Xanax) without a doctor's prescription? | □ | □ | □ | □ | □ | □ |
| g. ever sniffed glue, breathed the contents of spray cans, or inhaled any paints or sprays to get high? | □ | □ | □ | □ | □ | □ |
| h. used a needle to inject any illegal drug into your body? | □ | □ | □ | □ | □ | □ |

**16. Have you ever had vaginal sexual intercourse?**

□No □Yes □ I’m uncomfortable answering this question

17. In your whole life, with how many people did you have vaginal sexual intercourse?

□1 person □2 people □3 people □4 people □5 people □6 or more people

□I have never had sexual intercourse □I’m uncomfortable answering this question

18. The LAST TIME you had vaginal sexual intercourse, did you or your partner use a condom?

□ No □ Yes □ I have not had sexual intercourse

□I’m uncomfortable answering this question

**19. Have you ever had oral sex?**

□No □Yes □I’m uncomfortable answering this question

**SECTION 3: YOUR OPINIONS**

**The next set of questions asks you about your opinions about sexual intercourse, pregnancy, and relationships. Please answer to the best of your ability.**

**20. Please indicate how strongly you agree or disagree with the following….**

|  | Strongly Agree | Agree | Disagree | Strongly Disagree |
| --- | --- | --- | --- | --- |
| a. people should never take “no” for an answer when they want to have sex. | □ | □ | □ | □ |
| b. no one should pressure another person into sexual activity. | □ | □ | □ | □ |
| c. it is alright to pressure someone into sexual activity. | □ | □ | □ | □ |
| d. people should not pressure others to have sex with them. | □ | □ | □ | □ |
| e. it is alright to demand sex from a girlfriend or boyfriend. | □ | □ | □ | □ |

**21. Please indicate how much you agree or disagree with each statement…**

|  | Strongly Agree | Agree | Disagree | Strongly Disagree |
| --- | --- | --- | --- | --- |
| a. it is important for me not to have sex before I get married. | □ | □ | □ | □ |
| b. having sex should be viewed as just a normal and expected part of teen dating relationships. | □ | □ | □ | □ |
| c. even if I am physically mature, that doesn't mean I'm ready to have sex. | □ | □ | □ | □ |
| d. it is alright for teens to have sex if they are in love. | □ | □ | □ | □ |
| e. I think it is OK for kids my age to have sex. | □ | □ | □ | □ |

**22. These questions ask about pregnancy. Indicate how much you agree or disagree with each statement.**

| **If you or your partner got pregnant …** | Strongly  Agree | Agree | Disagree | Strongly Disagree |
| --- | --- | --- | --- | --- |
| a. it would be embarrassing for your family. | □ | □ | □ | □ |
| b. it would be embarrassing for you. | □ | □ | □ | □ |
| c. you would have to decide whether or not to have the baby, and that would be stressful and difficult. | □ | □ | □ | □ |
| d. you would be forced to grow up too fast. | □ | □ | □ | □ |
| e. it would be one of the worst things that could happen to you. | □ | □ | □ | □ |

**SECTION 4: FAMILY AND SCHOOL EXPERIENCES**

These next questions ask you about your experiences and relationships with your family and school. Please read the questions carefully and answer to the best of your ability.

23. How many times have you and a parent or guardian talked about…

|  | Never | Once or Twice | Many Times |
| --- | --- | --- | --- |
| a. sex? | □ | □ | □ |
| b. STDs (sexually transmitted diseases) or HIV? | □ | □ | □ |
| c. abstinence or waiting to have sex? | □ | □ | □ |
| d. relationships? | □ | □ | □ |
| e. how to know if you are ready for sex? | □ | □ | □ |
| f. how to handle sexual pressure? | □ | □ | □ |
| g. homosexuality? | □ | □ | □ |
| h. how to use a condom? | □ | □ | □ |
| i. how babies are made or preventing pregnancy? | □ | □ | □ |

**THESE NEXT FEW QUESTIONS ASK YOU ABOUT THE PAST 3 MONTHS.**

**24. How often in the *PAST 3 MONTHS*, have you:**

|  | Never | Once | Two or three times | About once a week | Several times a week |
| --- | --- | --- | --- | --- | --- |
| a. had someone say mean things or hurt ***your*** feelings? | □ | □ | □ | □ | □ |
| b. been excluded from a group or completely ignored? | □ | □ | □ | □ | □ |
| c. had someone hurt ***you*** physically? | □ | □ | □ | □ | □ |
| d. had false rumors spread about ***you***? | □ | □ | □ | □ | □ |
| e. had someone say mean things or hurt ***your*** feelings electronically (such as through e-mail, cell phone, or social networking sites)? | □ | □ | □ | □ | □ |
| f. had anyone use ***your*** username or screen-name to spread rumors or lies about someone else? | □ | □ | □ | □ | □ |

**25. We would like to know about the parent(s) or guardian(s) you feel raised you.**

|  | Not at all | Very little | Somewhat | Quite a bit | Very much |
| --- | --- | --- | --- | --- | --- |
| a. how close do you feel to that person or people? | □ | □ | □ | □ | □ |
| b. how much do you think that person or people cares about you? | □ | □ | □ | □ | □ |
| c. how satisfied are you with your relationship with that person or people? | □ | □ | □ | □ | □ |
| d. how loved do you feel by that person or people? | □ | □ | □ | □ | □ |
| e. how wanted do you feel by that person or people? | □ | □ | □ | □ | □ |

26. Now we would like to know more about your experiences at school, within the past school year. How strongly do you agree or disagree with the following statements?

|  | Strongly Agree | Agree | Disagree | Strongly Disagree |
| --- | --- | --- | --- | --- |
| a. I feel close to people at my school. | □ | □ | □ | □ |
| b. I feel like I am a part of my school. | □ | □ | □ | □ |
| c. I am happy to be at my school. | □ | □ | □ | □ |
| d. the teachers at my school treat students fairly. | □ | □ | □ | □ |
| e. I feel safe at my school. | □ | □ | □ | □ |

The End.
