# Supplementary material for: The role of trauma and positive youth development in polysubstance use among rural middle school students: a latent class analysis
Source: BMC Public Health. 2022 Dec 14;22:2350. doi: 10.1186/s12889-022-14795-1 (PMC9753425; doi:10.1186/s12889-022-14795-1)
Supplement: Supplementary file 2 — Additional file 2. [file 12889_2022_14795_MOESM2_ESM.pdf]

record_id	redcap_event_name	pre_program	pre_cohort	survey_year	pre_site	age_pre	grade_pre
1	presurvey_arm_1	1	230	4	1	11	6
2	presurvey_arm_1	1	230	4	1	11	6
3	presurvey_arm_1	1	230	3	1	11	6
4	presurvey_arm_1	1	230	4	1	12	6
5	presurvey_arm_1	1	230	4	1	11	6
6	presurvey_arm_1	1	230	4	1	11	6
7	presurvey_arm_1	1	230	4	1	11	6
8	presurvey_arm_1	1	230	3	1	11	6
9	presurvey_arm_1	1	230	4	1	11	6
10	presurvey_arm_1	1	230	4	1	11	6
11	presurvey_arm_1	1	230	4	1	11	6
12	presurvey_arm_1	1	230	4	1	11	6
13	presurvey_arm_1	1	230	4	1	11	6
14	presurvey_arm_1	1	230	3	1	11	6
15	presurvey_arm_1	1	230	3	1	11	6
16	presurvey_arm_1	1	230	4	1	11	6
17	presurvey_arm_1	1	230	4	1	11	6
18	presurvey_arm_1	1	230	4	1	13	6
19	presurvey_arm_1	1	230	4	1	11	6
20	presurvey_arm_1	1	230	4	1	12	6
21	presurvey_arm_1	1	230	4	1	11	6
22	presurvey_arm_1	1	230	4	1	11	6
23	presurvey_arm_1	1	230	4	1	11	6
24	presurvey_arm_1	1	231	4	1	11	6
25	presurvey_arm_1	1	231	4	1	11	6
26	presurvey_arm_1	1	231	4	1	11	6
27	presurvey_arm_1	1	231	4	1	11	6
28	presurvey_arm_1	1	231	4	1	11	6
29	presurvey_arm_1	1	231	4	1	11	6
30	presurvey_arm_1	1	231	4	1	11	6
31	presurvey_arm_1	1	231	4	1	12	6
32	presurvey_arm_1	1	231	4	1	12	6
33	presurvey_arm_1	1	231	4	1	11	6
34	presurvey_arm_1	1	231	4	1	11	6
35	presurvey_arm_1	1	231	4	1	11	6
36	presurvey_arm_1	1	231	4	1	11	6
37	presurvey_arm_1	1	231	4	1	11	6
38	presurvey_arm_1	1	231	4	1	11	6
39	presurvey_arm_1	1	231	4	1	11	6
40	presurvey_arm_1	1	231	4	1	11	6
41	presurvey_arm_1	1	231	4	1	12	6
42	presurvey_arm_1	1	231	4	1	11	6
43	presurvey_arm_1	1	231	4	1	11	6
44	presurvey_arm_1	1	231	4	1	11	6
45	presurvey_arm_1	2	232	3	1	12	7
46	presurvey_arm_1	2	232	4	1	12	7
47	presurvey_arm_1	2	232	4	1	13	7

48	presurvey_arm_1	2	232	4	1	12	7
49	presurvey_arm_1	2	232	4	1	12	7
50	presurvey_arm_1	2	232	4	1	12	7
51	presurvey_arm_1	2	232	4	1	12	7
52	presurvey_arm_1	2	232	4	1	12	7
53	presurvey_arm_1	2	232	4	1	12	7
54	presurvey_arm_1	2	232	4	1	12	7
55	presurvey_arm_1	2	232	4	1	12	7
56	presurvey_arm_1	2	232	4	1	12	7
57	presurvey_arm_1	2	232	4	1	12	7
58	presurvey_arm_1	2	232	4	1	12	7
59	presurvey_arm_1	2	232	4	1	12	7
60	presurvey_arm_1	2	232	4	1	12	7
61	presurvey_arm_1	2	233	4	1	13	7
62	presurvey_arm_1	2	233	4	1	12	7
63	presurvey_arm_1	2	233	4	1	12	7
64	presurvey_arm_1	2	233	4	1	12	7
65	presurvey_arm_1	2	233	4	1	12	7
66	presurvey_arm_1	2	233	4	1	12	7
67	presurvey_arm_1	2	233	4	1	11	7
68	presurvey_arm_1	2	233	4	1	12	7
69	presurvey_arm_1	2	233	4	1	12	7
70	presurvey_arm_1	2	233	4	1	12	7
71	presurvey_arm_1	2	233	4	1	12	7
72	presurvey_arm_1	2	233	4	1	12	7
73	presurvey_arm_1	2	233	4	1	12	7
74	presurvey_arm_1	2	233	4	1	12	7
75	presurvey_arm_1	2	233	4	1	12	7
76	presurvey_arm_1	2	233	4	1	13	7
77	presurvey_arm_1	2	233	4	1	12	7
78	presurvey_arm_1	2	233	4	1	12	7
79	presurvey_arm_1	2	233	3	1	12	7
80	presurvey_arm_1	2	233	4	1	12	7
81	presurvey_arm_1	2	233	4	1	13	7
82	presurvey_arm_1	2	233	4	1	12	7
83	presurvey_arm_1	2	233	4	1	12	7
84	presurvey_arm_1	3	234	4	1	13	8
85	presurvey_arm_1	3	234	4	1	13	8
86	presurvey_arm_1	3	234	4	1	13	8
87	presurvey_arm_1	3	234	4	1	14	8
88	presurvey_arm_1	3	234	4	1	13	8
89	presurvey_arm_1	3	234	3	1	13	8
90	presurvey_arm_1	3	234	4	1	13	8
91	presurvey_arm_1	3	234	4	1	14	8
92	presurvey_arm_1	3	234	4	1	13	8
93	presurvey_arm_1	3	234	3	1	13	8
94	presurvey_arm_1	3	234	4	1	13	8
95	presurvey_arm_1	3	234	4	1	13	8

96	presurvey_arm_1	3	234	4	1	14	8
97	presurvey_arm_1	3	234	4	1	14	8
98	presurvey_arm_1	3	234	4	1	13	8
99	presurvey_arm_1	3	234	4	1	13	8
100	presurvey_arm_1	3	235	4	1	13	8
101	presurvey_arm_1	3	235	4	1	13	8
102	presurvey_arm_1	3	235	4	1	13	8
103	presurvey_arm_1	3	235	4	1	13	8
104	presurvey_arm_1	3	235	4	1	13	8
105	presurvey_arm_1	3	235	4	1	13	8
106	presurvey_arm_1	3	235	4	1	14	8
107	presurvey_arm_1	3	235	4	1	13	8
108	presurvey_arm_1	3	235	4	1	13	8
109	presurvey_arm_1	3	235	4	1	13	8
110	presurvey_arm_1	3	235	4	1	13	8
111	presurvey_arm_1	3	235	4	1	13	8
112	presurvey_arm_1	3	235	4	1	14	8
113	presurvey_arm_1	3	235	4	1	13	8
114	presurvey_arm_1	3	235	4	1	14	8
115	presurvey_arm_1	3	235	4	1	13	8
226	presurvey_arm_1	2	241	4	1	12	7
227	presurvey_arm_1	2	241	4	1	12	7
228	presurvey_arm_1	2	241	4	1	13	7
229	presurvey_arm_1	2	241	4	1	13	7
230	presurvey_arm_1	2	241	4	1	12	7
231	presurvey_arm_1	2	241	4	1	12	7
232	presurvey_arm_1	2	241	4	1	12	7
233	presurvey_arm_1	2	241	3	1	13	7
234	presurvey_arm_1	2	241	4	1	12	7
235	presurvey_arm_1	2	241	4	1	12	7
236	presurvey_arm_1	2	241	3	1	16	7
237	presurvey_arm_1	2	241	4	1	12	7
238	presurvey_arm_1	2	241	4	1	12	7
239	presurvey_arm_1	2	241	4	1	13	7
240	presurvey_arm_1	2	241	4	1	12	7
241	presurvey_arm_1	2	241	4	1	12	7
242	presurvey_arm_1	2	241	4	1	12	7
243	presurvey_arm_1	2	242	4	1	12	7
244	presurvey_arm_1	2	242	4	1	12	7
245	presurvey_arm_1	2	242	4	1	13	7
246	presurvey_arm_1	2	242	4	1	12	7
247	presurvey_arm_1	2	242	4	1	12	7
248	presurvey_arm_1	2	242	4	1	12	7
249	presurvey_arm_1	2	242	4	1	12	7
250	presurvey_arm_1	2	242	4	1	13	7
251	presurvey_arm_1	2	242	4	1	12	7
252	presurvey_arm_1	2	242	4	1	12	7
253	presurvey_arm_1	2	242	4	1	12	7

254	presurvey_arm_1	2	242	4	1	12	7
255	presurvey_arm_1	2	242	3	1	13	7
256	presurvey_arm_1	2	242	4	1	13	7
257	presurvey_arm_1	2	242	4	1	13	7
258	presurvey_arm_1	2	242	4	1	13	7
259	presurvey_arm_1	2	242	4	1	13	7
260	presurvey_arm_1	2	242	4	1	13	7
261	presurvey_arm_1	2	242	4	1	12	7
262	presurvey_arm_1	2	242	4	1	12	7
263	presurvey_arm_1	2	242	4	1	13	7
264	presurvey_arm_1	2	242	4	1	12	7
265	presurvey_arm_1	2	242	4	1	12	7
266	presurvey_arm_1	2	242	4	1	12	7
267	presurvey_arm_1	1	239	4	1	11	6
268	presurvey_arm_1	1	239	4	1	11	6
269	presurvey_arm_1	1	239	4	1	12	6
270	presurvey_arm_1	1	239	4	1	11	6
271	presurvey_arm_1	1	239	4	1	12	6
272	presurvey_arm_1	1	239	4	1	12	6
273	presurvey_arm_1	1	239	4	1	11	6
274	presurvey_arm_1	1	239	4	1	11	6
275	presurvey_arm_1	1	239	4	1	11	6
276	presurvey_arm_1	1	239	4	1	11	6
277	presurvey_arm_1	1	239	4	1	11	6
278	presurvey_arm_1	1	239	4	1	11	6
279	presurvey_arm_1	1	239	4	1	11	6
280	presurvey_arm_1	1	239	4	1	11	6
281	presurvey_arm_1	1	239	4	1	12	6
282	presurvey_arm_1	1	239	4	1	12	6
283	presurvey_arm_1	1	239	4	1	11	6
284	presurvey_arm_1	1	239	4	1	11	6
285	presurvey_arm_1	1	239	4	1	11	6
286	presurvey_arm_1	1	239	4	1	11	6
287	presurvey_arm_1	1	240	4	1	99	6
288	presurvey_arm_1	1	240	4	1	11	6
289	presurvey_arm_1	1	240	4	1	12	6
290	presurvey_arm_1	1	240	4	1	11	6
291	presurvey_arm_1	1	240	4	1	11	6
292	presurvey_arm_1	1	240	4	1	12	6
293	presurvey_arm_1	1	240	4	1	12	6
294	presurvey_arm_1	1	240	3	1	11	6
295	presurvey_arm_1	1	240	4	1	11	6
296	presurvey_arm_1	1	240	4	1	12	6
297	presurvey_arm_1	1	240	4	1	11	6
298	presurvey_arm_1	1	240	4	1	11	6
299	presurvey_arm_1	1	240	4	1	11	6
300	presurvey_arm_1	1	240	4	1	11	6
301	presurvey_arm_1	1	240	4	1	11	6

302	presurvey_arm_1	1	240	4	1	11	6
303	presurvey_arm_1	1	240	4	1	11	6
304	presurvey_arm_1	1	240	3	1	11	6
305	presurvey_arm_1	1	240	4	1	12	6
306	presurvey_arm_1	1	240	3	1	11	6
307	presurvey_arm_1	3	243	4	1	13	8
308	presurvey_arm_1	3	243	4	1	14	8
309	presurvey_arm_1	3	243	4	1	13	8
310	presurvey_arm_1	3	243	4	1	14	8
311	presurvey_arm_1	3	243	4	1	13	8
312	presurvey_arm_1	3	243	4	1	13	8
313	presurvey_arm_1	3	243	4	1	14	8
314	presurvey_arm_1	3	243	4	1	13	8
315	presurvey_arm_1	3	243	4	1	13	8
316	presurvey_arm_1	3	243	4	1	13	8
317	presurvey_arm_1	3	243	4	1	14	8
318	presurvey_arm_1	3	243	4	1	13	8
319	presurvey_arm_1	3	243	4	1	13	8
320	presurvey_arm_1	3	243	4	1	13	8
321	presurvey_arm_1	3	243	4	1	13	8
322	presurvey_arm_1	3	243	4	1	14	8
323	presurvey_arm_1	3	243	4	1	15	8
324	presurvey_arm_1	3	243	4	1	13	8
325	presurvey_arm_1	3	243	4	1	13	8
326	presurvey_arm_1	3	243	4	1	13	8
327	presurvey_arm_1	3	244	4	1	14	8
328	presurvey_arm_1	3	244	4	1	13	8
329	presurvey_arm_1	3	244	4	1	13	8
330	presurvey_arm_1	3	244	3	1	13	8
331	presurvey_arm_1	3	244	4	1	13	8
332	presurvey_arm_1	3	244	3	1	13	8
333	presurvey_arm_1	3	244	4	1	13	8
334	presurvey_arm_1	3	244	4	1	13	8
335	presurvey_arm_1	3	244	4	1	14	8
336	presurvey_arm_1	3	244	4	1	13	8
337	presurvey_arm_1	3	244	4	1	13	8
338	presurvey_arm_1	3	244	4	1	13	8
339	presurvey_arm_1	3	244	4	1	13	8
340	presurvey_arm_1	3	244	4	1	13	8
341	presurvey_arm_1	3	244	4	1	14	8
342	presurvey_arm_1	3	244	4	1	14	99
343	presurvey_arm_1	3	244	4	1	13	8
344	presurvey_arm_1	3	244	4	1	13	8
363	presurvey_arm_1	1	246	4	1	12	6
364	presurvey_arm_1	1	246	4	1	12	6
365	presurvey_arm_1	1	246	4	1	11	6
366	presurvey_arm_1	1	246	4	1	12	6
367	presurvey_arm_1	1	246	3	1	11	6

368	presurvey_arm_1	1	246	4	1	12	6
369	presurvey_arm_1	1	246	4	1	11	6
370	presurvey_arm_1	1	246	4	1	11	6
371	presurvey_arm_1	1	246	4	1	12	6
372	presurvey_arm_1	1	246	4	1	12	6
373	presurvey_arm_1	1	246	4	1	11	6
374	presurvey_arm_1	1	246	4	1	11	6
375	presurvey_arm_1	1	246	4	1	12	6
376	presurvey_arm_1	1	246	4	1	11	6
377	presurvey_arm_1	1	246	4	1	13	6
378	presurvey_arm_1	1	246	4	1	12	6
379	presurvey_arm_1	1	246	4	1	12	6
380	presurvey_arm_1	1	246	4	1	12	6
381	presurvey_arm_1	1	246	4	1	12	6
382	presurvey_arm_1	1	246	4	1	11	6
383	presurvey_arm_1	1	246	4	1	12	6
384	presurvey_arm_1	1	246	4	1	11	6
385	presurvey_arm_1	1	246	4	1	11	6
386	presurvey_arm_1	1	247	4	1	12	6
387	presurvey_arm_1	1	247	4	1	12	6
388	presurvey_arm_1	1	247	4	1	11	6
389	presurvey_arm_1	1	247	4	1	11	6
390	presurvey_arm_1	1	247	4	1	11	6
391	presurvey_arm_1	1	247	4	1	12	6
392	presurvey_arm_1	1	247	4	1	11	6
393	presurvey_arm_1	1	247	4	1	11	6
394	presurvey_arm_1	1	247	4	1	11	6
395	presurvey_arm_1	1	247	4	1	11	6
396	presurvey_arm_1	1	247	4	1	12	6
397	presurvey_arm_1	1	247	4	1	11	6
398	presurvey_arm_1	1	247	4	1	11	6
399	presurvey_arm_1	1	247	4	1	11	6
400	presurvey_arm_1	1	247	4	1	11	6
401	presurvey_arm_1	1	247	4	1	11	6
402	presurvey_arm_1	1	247	4	1	12	6
403	presurvey_arm_1	2	248	4	1	13	7
404	presurvey_arm_1	2	248	4	1	12	7
405	presurvey_arm_1	2	248	4	1	12	7
406	presurvey_arm_1	2	248	4	1	12	7
407	presurvey_arm_1	2	248	4	1	13	7
408	presurvey_arm_1	2	248	4	1	12	7
409	presurvey_arm_1	2	248	4	1	12	7
410	presurvey_arm_1	2	248	4	1	12	7
411	presurvey_arm_1	2	248	4	1	12	7
412	presurvey_arm_1	2	248	4	1	13	7
413	presurvey_arm_1	2	248	4	1	12	7
414	presurvey_arm_1	2	248	4	1	12	7
415	presurvey_arm_1	2	248	4	1	13	7

416	presurvey_arm_1	2	248	4	1	12	7
417	presurvey_arm_1	2	248	4	1	12	7
418	presurvey_arm_1	2	248	4	1	13	7
419	presurvey_arm_1	2	248	4	1	12	7
420	presurvey_arm_1	2	248	4	1	13	7
421	presurvey_arm_1	2	248	4	1	12	7
422	presurvey_arm_1	2	248	4	1	13	7
423	presurvey_arm_1	2	248	4	1	12	7
424	presurvey_arm_1	2	248	4	1	13	7
425	presurvey_arm_1	2	248	4	1	13	7
426	presurvey_arm_1	2	248	4	1	12	7
427	presurvey_arm_1	2	249	4	1	12	7
428	presurvey_arm_1	2	249	4	1	12	7
429	presurvey_arm_1	2	249	4	1	12	7
430	presurvey_arm_1	2	249	4	1	13	7
431	presurvey_arm_1	2	249	4	1	12	7
432	presurvey_arm_1	2	249	4	1	11	7
433	presurvey_arm_1	2	249	4	1	13	7
434	presurvey_arm_1	2	249	4	1	12	7
435	presurvey_arm_1	2	249	4	1	12	7
436	presurvey_arm_1	2	249	4	1	12	7
437	presurvey_arm_1	2	249	4	1	13	7
438	presurvey_arm_1	2	249	4	1	13	7
439	presurvey_arm_1	2	249	4	1	13	7
440	presurvey_arm_1	2	249	4	1	13	7
441	presurvey_arm_1	2	249	4	1	12	7
442	presurvey_arm_1	2	249	4	1	13	7
443	presurvey_arm_1	2	249	4	1	12	7
444	presurvey_arm_1	2	249	4	1	13	7
445	presurvey_arm_1	2	249	4	1	12	7
446	presurvey_arm_1	2	249	4	1	12	7
447	presurvey_arm_1	2	249	4	1	13	7
448	presurvey_arm_1	2	249	4	1	14	7
449	presurvey_arm_1	3	250	4	1	13	8
450	presurvey_arm_1	3	250	4	1	11	8
451	presurvey_arm_1	3	250	4	1	14	8
452	presurvey_arm_1	3	250	4	1	13	8
453	presurvey_arm_1	3	250	4	1	13	8
454	presurvey_arm_1	3	250	4	1	13	8
455	presurvey_arm_1	3	250	4	1	13	8
456	presurvey_arm_1	3	250	4	1	14	8
457	presurvey_arm_1	3	250	4	1	14	8
458	presurvey_arm_1	3	250	4	1	14	8
459	presurvey_arm_1	3	250	4	1	14	8
460	presurvey_arm_1	3	250	4	1	13	8
461	presurvey_arm_1	3	250	4	1	13	8
462	presurvey_arm_1	3	250	4	1	13	8
463	presurvey_arm_1	3	250	4	1	13	8

464	presurvey_arm_1	3	250	4	1	13	8
465	presurvey_arm_1	3	250	4	1	13	8
466	presurvey_arm_1	3	250	4	1	13	8
467	presurvey_arm_1	3	250	4	1	14	8
468	presurvey_arm_1	3	250	4	1	13	8
469	presurvey_arm_1	3	250	4	1	14	8
470	presurvey_arm_1	3	251	4	1	13	8
471	presurvey_arm_1	3	251	4	1	13	8
472	presurvey_arm_1	3	251	4	1	14	8
473	presurvey_arm_1	3	251	4	1	13	8
474	presurvey_arm_1	3	251	4	1	14	8
475	presurvey_arm_1	3	251	4	1	13	8
476	presurvey_arm_1	3	251	4	1	13	8
477	presurvey_arm_1	3	251	4	1	14	8
478	presurvey_arm_1	3	251	4	1	14	8
479	presurvey_arm_1	3	251	4	1	13	8
480	presurvey_arm_1	3	251	4	1	14	8
481	presurvey_arm_1	3	251	4	1	13	8
482	presurvey_arm_1	3	251	4	1	13	8
483	presurvey_arm_1	3	251	4	1	13	8
484	presurvey_arm_1	3	251	4	1	13	8
485	presurvey_arm_1	3	251	4	1	13	8
486	presurvey_arm_1	3	251	4	1	13	99
711	presurvey_arm_1	1	254	4	1	11	6
712	presurvey_arm_1	1	254	4	1	12	6
713	presurvey_arm_1	1	254	4	1	12	6
714	presurvey_arm_1	1	254	4	1	12	6
715	presurvey_arm_1	1	254	4	1	11	6
716	presurvey_arm_1	1	254	4	1	11	6
717	presurvey_arm_1	1	254	4	1	12	6
718	presurvey_arm_1	1	254	4	1	11	6
719	presurvey_arm_1	1	254	4	1	13	6
720	presurvey_arm_1	1	254	4	1	11	6
721	presurvey_arm_1	1	254	4	1	11	6
722	presurvey_arm_1	1	254	4	1	11	6
723	presurvey_arm_1	1	254	4	1	12	6
724	presurvey_arm_1	1	254	4	1	11	6
725	presurvey_arm_1	1	254	4	1	12	6
726	presurvey_arm_1	1	254	4	1	11	6
727	presurvey_arm_1	1	254	4	1	12	6
728	presurvey_arm_1	1	254	4	1	12	6
729	presurvey_arm_1	1	254	4	1	12	6
730	presurvey_arm_1	1	254	4	1	11	6
731	presurvey_arm_1	1	254	4	1	11	6
732	presurvey_arm_1	1	254	4	1	12	6
733	presurvey_arm_1	1	255	4	1	12	6
734	presurvey_arm_1	1	255	4	1	12	6
735	presurvey_arm_1	1	255	4	1	11	6

736	presurvey_arm_1	1	255	4	1	11	6
737	presurvey_arm_1	1	255	4	1	11	6
738	presurvey_arm_1	1	255	4	1	12	6
739	presurvey_arm_1	1	255	4	1	11	6
740	presurvey_arm_1	1	255	4	1	12	6
741	presurvey_arm_1	1	255	4	1	12	6
742	presurvey_arm_1	1	255	4	1	11	6
743	presurvey_arm_1	1	255	4	1	12	6
744	presurvey_arm_1	1	255	4	1	11	6
745	presurvey_arm_1	1	255	4	1	12	6
746	presurvey_arm_1	1	255	4	1	12	6
747	presurvey_arm_1	1	255	4	1	12	6
748	presurvey_arm_1	1	255	4	1	11	6
749	presurvey_arm_1	1	255	4	1	11	6
750	presurvey_arm_1	1	255	4	1	11	6
751	presurvey_arm_1	2	256	4	1	13	7
752	presurvey_arm_1	2	256	4	1	13	7
753	presurvey_arm_1	2	256	4	1	12	7
754	presurvey_arm_1	2	256	4	1	13	7
755	presurvey_arm_1	2	256	4	1	12	7
756	presurvey_arm_1	2	256	4	1	13	7
757	presurvey_arm_1	2	256	4	1	12	7
758	presurvey_arm_1	2	256	4	1	12	7
759	presurvey_arm_1	2	256	4	1	13	7
760	presurvey_arm_1	2	256	4	1	12	7
761	presurvey_arm_1	2	256	4	1	12	7
762	presurvey_arm_1	2	256	4	1	12	7
763	presurvey_arm_1	2	256	4	1	12	7
764	presurvey_arm_1	2	256	4	1	13	7
765	presurvey_arm_1	2	256	4	1	12	7
766	presurvey_arm_1	2	256	4	1	13	7
767	presurvey_arm_1	2	256	4	1	13	7
768	presurvey_arm_1	2	256	4	1	12	7
769	presurvey_arm_1	2	256	4	1	13	7
770	presurvey_arm_1	2	256	4	1	12	7
771	presurvey_arm_1	2	257	4	1	13	7
772	presurvey_arm_1	2	257	4	1	12	7
773	presurvey_arm_1	2	257	4	1	12	7
774	presurvey_arm_1	2	257	4	1	13	7
775	presurvey_arm_1	2	257	4	1	12	7
776	presurvey_arm_1	2	257	4	1	13	7
777	presurvey_arm_1	2	257	4	1	13	7
778	presurvey_arm_1	2	257	4	1	12	7
779	presurvey_arm_1	2	257	4	1	13	7
780	presurvey_arm_1	2	257	4	1	13	7
781	presurvey_arm_1	2	257	4	1	13	7
782	presurvey_arm_1	2	257	4	1	13	7
783	presurvey_arm_1	2	257	4	1	13	7

784	presurvey_arm_1	2	257	4	1	13	7
785	presurvey_arm_1	2	257	4	1	12	7
786	presurvey_arm_1	2	257	4	1	12	7
787	presurvey_arm_1	2	257	4	1	12	7
788	presurvey_arm_1	3	258	4	1	13	8
789	presurvey_arm_1	3	258	4	1	13	8
790	presurvey_arm_1	3	258	4	1	14	8
791	presurvey_arm_1	3	258	4	1	13	8
792	presurvey_arm_1	3	258	4	1	14	8
793	presurvey_arm_1	3	258	4	1	14	8
794	presurvey_arm_1	3	258	4	1	14	8
795	presurvey_arm_1	3	258	4	1	14	8
796	presurvey_arm_1	3	258	4	1	13	8
797	presurvey_arm_1	3	258	4	1	14	8
798	presurvey_arm_1	3	258	4	1	14	8
799	presurvey_arm_1	3	258	4	1	13	8
800	presurvey_arm_1	3	258	4	1	14	8
801	presurvey_arm_1	3	258	4	1	14	8
802	presurvey_arm_1	3	258	4	1	14	8
803	presurvey_arm_1	3	258	4	1	13	8
804	presurvey_arm_1	3	258	4	1	14	8
805	presurvey_arm_1	3	258	4	1	14	8
806	presurvey_arm_1	3	259	4	1	14	8
807	presurvey_arm_1	3	259	4	1	13	8
808	presurvey_arm_1	3	259	4	1	14	8
809	presurvey_arm_1	3	259	4	1	14	8
810	presurvey_arm_1	3	259	4	1	13	8
811	presurvey_arm_1	3	259	4	1	13	8
812	presurvey_arm_1	3	259	4	1	13	8
813	presurvey_arm_1	3	259	4	1	13	8
814	presurvey_arm_1	3	259	4	1	13	8
815	presurvey_arm_1	3	259	4	1	14	8
816	presurvey_arm_1	3	259	4	1	13	8
817	presurvey_arm_1	3	259	4	1	14	8
818	presurvey_arm_1	3	259	4	1	14	8
819	presurvey_arm_1	3	259	4	1	13	8
820	presurvey_arm_1	3	259	4	1	13	8
821	presurvey_arm_1	3	259	4	1	13	8
822	presurvey_arm_1	3	259	4	1	14	8
823	presurvey_arm_1	3	259	4	1	13	8
824	presurvey_arm_1	3	259	4	1	14	8
825	presurvey_arm_1	3	259	4	1	13	8
938	presurvey_arm_1	1	263	4	1	12	6
939	presurvey_arm_1	1	263	4	1	11	6
940	presurvey_arm_1	1	263	4	1	12	6
941	presurvey_arm_1	1	263	5	1	12	6
942	presurvey_arm_1	1	263	4	1	11	6
943	presurvey_arm_1	1	263	4	1	11	6

944	presurvey_arm_1	1	263	4	1	11	6
945	presurvey_arm_1	1	263	4	1	12	6
946	presurvey_arm_1	1	263	4	1	12	6
947	presurvey_arm_1	1	263	4	1	12	6
948	presurvey_arm_1	1	263	4	1	11	6
949	presurvey_arm_1	1	263	5	1	11	6
950	presurvey_arm_1	1	263	4	1	11	6
951	presurvey_arm_1	1	263	4	1	12	6
952	presurvey_arm_1	1	263	4	1	13	6
953	presurvey_arm_1	1	263	4	1	12	6
954	presurvey_arm_1	1	263	4	1	12	6
955	presurvey_arm_1	1	263	4	1	12	6
956	presurvey_arm_1	1	263	4	1	11	6
957	presurvey_arm_1	1	263	4	1	12	6
958	presurvey_arm_1	1	263	4	1	12	6
959	presurvey_arm_1	1	263	4	1	12	6
960	presurvey_arm_1	1	264	4	1	11	6
961	presurvey_arm_1	1	264	4	1	11	6
962	presurvey_arm_1	1	264	4	1	12	6
963	presurvey_arm_1	1	264	4	1	12	6
964	presurvey_arm_1	1	264	5	1	12	6
965	presurvey_arm_1	1	264	4	1	12	6
966	presurvey_arm_1	1	264	5	1	11	6
967	presurvey_arm_1	1	264	4	1	12	6
968	presurvey_arm_1	1	264	4	1	11	6
969	presurvey_arm_1	1	264	4	1	11	6
970	presurvey_arm_1	1	264	4	1	12	6
971	presurvey_arm_1	1	264	4	1	11	6
972	presurvey_arm_1	1	264	4	1	12	6
973	presurvey_arm_1	1	264	4	1	11	6
974	presurvey_arm_1	1	264	5	1	12	6
975	presurvey_arm_1	1	264	4	1	13	6
976	presurvey_arm_1	1	264	5	1	12	6
977	presurvey_arm_1	1	264	4	1	13	7
978	presurvey_arm_1	1	264	4	1	13	7
979	presurvey_arm_1	2	265	4	1	13	7
980	presurvey_arm_1	2	265	4	1	13	7
981	presurvey_arm_1	2	265	4	1	12	7
982	presurvey_arm_1	2	265	4	1	13	7
983	presurvey_arm_1	2	265	4	1	12	7
984	presurvey_arm_1	2	265	4	1	12	7
985	presurvey_arm_1	2	265	4	1	13	7
986	presurvey_arm_1	2	265	4	1	12	7
987	presurvey_arm_1	2	265	4	1	13	7
988	presurvey_arm_1	2	265	4	1	12	7
989	presurvey_arm_1	2	265	5	1	12	7
990	presurvey_arm_1	2	265	5	1	13	7
991	presurvey_arm_1	2	265	4	1	12	7

992	presurvey_arm_1	2	265	5	1	13	7
993	presurvey_arm_1	2	265	4	1	12	7
994	presurvey_arm_1	2	265	4	1	12	7
995	presurvey_arm_1	2	265	4	1	12	7
996	presurvey_arm_1	2	265	5	1	13	7
997	presurvey_arm_1	2	265	4	1	13	7
998	presurvey_arm_1	2	265	4	1	13	7
999	presurvey_arm_1	2	265	4	1	12	7
1000	presurvey_arm_1	2	265	4	1	13	7
1001	presurvey_arm_1	2	266	4	1	12	7
1002	presurvey_arm_1	2	266	5	1	12	7
1003	presurvey_arm_1	2	266	4	1	12	7
1004	presurvey_arm_1	2	266	4	1	13	7
1005	presurvey_arm_1	2	266	4	1	14	7
1006	presurvey_arm_1	2	266	4	1	13	7
1007	presurvey_arm_1	2	266	4	1	12	7
1008	presurvey_arm_1	2	266	4	1	13	7
1009	presurvey_arm_1	2	266	4	1	13	7
1010	presurvey_arm_1	2	266	4	1	12	7
1011	presurvey_arm_1	2	266	4	1	13	7
1012	presurvey_arm_1	2	266	4	1	13	7
1013	presurvey_arm_1	2	266	4	1	13	7
1014	presurvey_arm_1	2	266	4	1	12	7
1015	presurvey_arm_1	2	266	4	1	13	7
1016	presurvey_arm_1	2	266	4	1	13	7
1017	presurvey_arm_1	2	266	5	1	12	7
1018	presurvey_arm_1	2	266	4	1	13	7
1019	presurvey_arm_1	2	266	5	1	13	7
1020	presurvey_arm_1	2	266	4	1	13	7
1021	presurvey_arm_1	2	266	4	1	12	7
1022	presurvey_arm_1	3	267	4	1	13	8
1023	presurvey_arm_1	3	267	4	1	13	8
1024	presurvey_arm_1	3	267	5	1	14	99
1025	presurvey_arm_1	3	267	4	1	13	8
1026	presurvey_arm_1	3	267	4	1	14	8
1027	presurvey_arm_1	3	267	4	1	13	8
1028	presurvey_arm_1	3	267	4	1	13	8
1029	presurvey_arm_1	3	267	4	1	14	8
1030	presurvey_arm_1	3	267	4	1	13	8
1031	presurvey_arm_1	3	267	4	1	14	8
1032	presurvey_arm_1	3	267	4	1	14	8
1033	presurvey_arm_1	3	267	4	1	14	8
1034	presurvey_arm_1	3	267	4	1	14	8
1035	presurvey_arm_1	3	267	5	1	15	8
1036	presurvey_arm_1	3	267	4	1	14	8
1037	presurvey_arm_1	3	267	4	1	13	8
1038	presurvey_arm_1	3	267	4	1	13	8
1039	presurvey_arm_1	3	268	4	1	13	8

1040	presurvey_arm_1	3	268	4	1	14	8
1041	presurvey_arm_1	3	268	4	1	14	8
1042	presurvey_arm_1	3	268	4	1	13	8
1043	presurvey_arm_1	3	268	4	1	13	8
1044	presurvey_arm_1	3	268	4	1	14	8
1045	presurvey_arm_1	3	268	4	1	14	8
1046	presurvey_arm_1	3	268	4	1	14	8
1047	presurvey_arm_1	3	268	4	1	14	8
1048	presurvey_arm_1	3	268	4	1	13	8
1049	presurvey_arm_1	3	268	4	1	13	8
1050	presurvey_arm_1	3	268	4	1	14	8
1051	presurvey_arm_1	3	268	4	1	14	8
1052	presurvey_arm_1	1	183	3	1	11	6
1053	presurvey_arm_1	1	183	3	1	12	6
1054	presurvey_arm_1	1	183	3	1	11	6
1055	presurvey_arm_1	1	183	3	1	11	6
1056	presurvey_arm_1	1	183	3	1	12	6
1057	presurvey_arm_1	1	183	3	1	11	6
1058	presurvey_arm_1	1	183	3	1	11	6
1059	presurvey_arm_1	1	183	3	1	11	6
1060	presurvey_arm_1	1	183	3	1	11	6
1061	presurvey_arm_1	1	183	3	1	12	6
1062	presurvey_arm_1	1	183	3	1	11	6
1063	presurvey_arm_1	1	183	2	1	11	6
1064	presurvey_arm_1	1	183	2	1	11	6
1065	presurvey_arm_1	1	183	3	1	11	6
1066	presurvey_arm_1	1	183	3	1	11	6
1067	presurvey_arm_1	1	183	2	1	11	6
1068	presurvey_arm_1	1	183	3	1	12	6
1069	presurvey_arm_1	1	183	3	1	11	6
1070	presurvey_arm_1	1	183	3	1	99	99
1071	presurvey_arm_1	1	183	3	1	12	6
1072	presurvey_arm_1	1	183	2	1	11	6
1073	presurvey_arm_1	1	183	3	1	11	6
1074	presurvey_arm_1	1	183	3	1	11	6
1075	presurvey_arm_1	1	183	3	1	11	6
1076	presurvey_arm_1	1	183	3	1	11	6
1077	presurvey_arm_1	1	183	3	1	11	6
1078	presurvey_arm_1	1	184	3	1	11	6
1079	presurvey_arm_1	1	184	3	1	11	6
1080	presurvey_arm_1	1	184	3	1	11	6
1081	presurvey_arm_1	1	184	3	1	11	6
1082	presurvey_arm_1	1	184	2	1	11	6
1083	presurvey_arm_1	1	184	3	1	10	6
1084	presurvey_arm_1	1	184	3	1	11	6
1085	presurvey_arm_1	1	184	3	1	11	6
1086	presurvey_arm_1	1	184	3	1	11	6
1087	presurvey_arm_1	1	184	3	1	11	6

1088	presurvey_arm_1	1	184	3	1	11	6
1089	presurvey_arm_1	1	184	3	1	11	6
1090	presurvey_arm_1	1	184	3	1	11	6
1091	presurvey_arm_1	1	184	3	1	11	6
1092	presurvey_arm_1	1	184	3	1	12	6
1093	presurvey_arm_1	1	184	3	1	12	6
1094	presurvey_arm_1	1	184	3	1	11	6
1095	presurvey_arm_1	1	184	3	1	11	6
1096	presurvey_arm_1	1	184	3	1	11	6
1097	presurvey_arm_1	1	184	2	1	11	6
1098	presurvey_arm_1	1	184	3	1	11	6
1099	presurvey_arm_1	1	184	3	1	11	6
1100	presurvey_arm_1	1	184	2	1	11	6
1101	presurvey_arm_1	1	184	3	1	11	6
1102	presurvey_arm_1	2	185	3	1	13	7
1103	presurvey_arm_1	2	185	3	1	12	7
1104	presurvey_arm_1	2	185	3	1	12	7
1105	presurvey_arm_1	2	185	3	1	12	7
1106	presurvey_arm_1	2	185	3	1	13	7
1107	presurvey_arm_1	2	185	3	1	13	7
1108	presurvey_arm_1	2	185	3	1	12	7
1109	presurvey_arm_1	2	185	2	1	12	7
1110	presurvey_arm_1	2	185	3	1	12	7
1111	presurvey_arm_1	2	185	3	1	12	7
1112	presurvey_arm_1	2	185	3	1	13	7
1113	presurvey_arm_1	2	185	3	1	14	7
1114	presurvey_arm_1	2	185	3	1	12	7
1115	presurvey_arm_1	2	185	3	1	11	7
1116	presurvey_arm_1	2	185	3	1	12	7
1117	presurvey_arm_1	2	185	3	1	13	7
1118	presurvey_arm_1	2	185	3	1	12	7
1119	presurvey_arm_1	2	185	3	1	12	7
1120	presurvey_arm_1	2	185	3	1	12	7
1121	presurvey_arm_1	2	185	3	1	13	7
1122	presurvey_arm_1	2	185	3	1	12	7
1123	presurvey_arm_1	2	185	3	1	13	7
1124	presurvey_arm_1	2	185	3	1	14	8
1125	presurvey_arm_1	2	185	3	1	13	7
1126	presurvey_arm_1	2	186	3	1	12	7
1127	presurvey_arm_1	2	186	3	1	12	7
1128	presurvey_arm_1	2	186	3	1	13	7
1129	presurvey_arm_1	2	186	3	1	12	7
1130	presurvey_arm_1	2	186	3	1	12	7
1131	presurvey_arm_1	2	186	3	1	12	7
1132	presurvey_arm_1	2	186	3	1	12	7
1133	presurvey_arm_1	2	186	3	1	11	7
1134	presurvey_arm_1	2	186	2	1	12	7
1135	presurvey_arm_1	2	186	3	1	12	7

1136	presurvey_arm_1	2	186	3	1	12	7
1137	presurvey_arm_1	2	186	3	1	11	7
1138	presurvey_arm_1	2	186	2	1	12	7
1139	presurvey_arm_1	2	186	3	1	12	7
1140	presurvey_arm_1	2	186	3	1	12	7
1141	presurvey_arm_1	2	186	3	1	12	7
1142	presurvey_arm_1	3	187	3	1	15	8
1143	presurvey_arm_1	3	187	3	1	13	8
1144	presurvey_arm_1	3	187	3	1	13	8
1145	presurvey_arm_1	3	187	2	1	13	8
1146	presurvey_arm_1	3	187	3	1	13	8
1147	presurvey_arm_1	3	187	2	1	13	8
1148	presurvey_arm_1	3	187	3	1	13	8
1149	presurvey_arm_1	3	187	3	1	14	8
1150	presurvey_arm_1	3	187	3	1	14	8
1151	presurvey_arm_1	3	187	3	1	14	8
1152	presurvey_arm_1	3	187	2	1	13	8
1153	presurvey_arm_1	3	187	2	1	13	8
1154	presurvey_arm_1	3	187	3	1	13	8
1155	presurvey_arm_1	3	187	3	1	13	8
1156	presurvey_arm_1	3	187	3	1	13	8
1157	presurvey_arm_1	3	187	3	1	13	8
1158	presurvey_arm_1	3	187	3	1	13	8
1159	presurvey_arm_1	3	187	3	1	14	8
1160	presurvey_arm_1	3	187	3	1	13	8
1161	presurvey_arm_1	1	191	2	1	12	6
1162	presurvey_arm_1	1	191	3	1	11	6
1163	presurvey_arm_1	1	191	3	1	11	6
1164	presurvey_arm_1	1	191	3	1	11	6
1165	presurvey_arm_1	1	191	3	1	11	99
1166	presurvey_arm_1	1	191	3	1	11	6
1167	presurvey_arm_1	1	191	3	1	12	6
1168	presurvey_arm_1	1	191	3	1	12	6
1169	presurvey_arm_1	1	191	3	1	11	6
1170	presurvey_arm_1	1	191	3	1	11	6
1171	presurvey_arm_1	1	191	3	1	11	6
1172	presurvey_arm_1	1	191	3	1	12	6
1173	presurvey_arm_1	1	191	3	1	12	6
1174	presurvey_arm_1	1	191	3	1	11	6
1175	presurvey_arm_1	1	191	3	1	11	6
1176	presurvey_arm_1	1	191	3	1	11	6
1177	presurvey_arm_1	1	191	3	1	11	6
1178	presurvey_arm_1	1	191	3	1	11	6
1179	presurvey_arm_1	1	191	3	1	11	6
1180	presurvey_arm_1	1	191	3	1	11	6
1181	presurvey_arm_1	1	191	3	1	11	6
1182	presurvey_arm_1	1	191	3	1	12	6
1183	presurvey_arm_1	1	191	3	1	12	6

1184	presurvey_arm_1	1	191	3	1	11	6
1185	presurvey_arm_1	1	191	3	1	11	6
1186	presurvey_arm_1	1	192	3	1	11	6
1187	presurvey_arm_1	1	192	3	1	11	6
1188	presurvey_arm_1	1	192	3	1	11	6
1189	presurvey_arm_1	1	192	2	1	11	6
1190	presurvey_arm_1	1	192	3	1	11	6
1191	presurvey_arm_1	1	192	3	1	11	6
1192	presurvey_arm_1	1	192	3	1	11	6
1193	presurvey_arm_1	1	192	3	1	11	6
1194	presurvey_arm_1	1	192	3	1	11	6
1195	presurvey_arm_1	1	192	3	1	11	6
1196	presurvey_arm_1	1	192	3	1	11	7
1197	presurvey_arm_1	1	192	3	1	11	6
1198	presurvey_arm_1	1	192	3	1	11	6
1199	presurvey_arm_1	1	192	3	1	11	6
1200	presurvey_arm_1	1	192	3	1	11	6
1201	presurvey_arm_1	1	192	3	1	11	6
1202	presurvey_arm_1	1	192	3	1	11	6
1203	presurvey_arm_1	1	192	3	1	99	6
1204	presurvey_arm_1	2	193	3	1	13	7
1205	presurvey_arm_1	2	193	2	1	12	7
1206	presurvey_arm_1	2	193	3	1	12	7
1207	presurvey_arm_1	2	193	3	1	12	7
1208	presurvey_arm_1	2	193	3	1	12	7
1209	presurvey_arm_1	2	193	3	1	12	7
1210	presurvey_arm_1	2	193	3	1	12	7
1211	presurvey_arm_1	2	193	2	1	12	7
1212	presurvey_arm_1	2	193	3	1	12	7
1213	presurvey_arm_1	2	193	3	1	12	7
1214	presurvey_arm_1	2	193	3	1	12	7
1215	presurvey_arm_1	2	193	3	1	12	7
1216	presurvey_arm_1	2	193	3	1	12	7
1217	presurvey_arm_1	2	193	3	1	12	7
1218	presurvey_arm_1	2	193	3	1	12	7
1219	presurvey_arm_1	2	193	3	1	12	7
1220	presurvey_arm_1	2	193	3	1	12	7
1221	presurvey_arm_1	2	193	2	1	13	7
1222	presurvey_arm_1	2	193	3	1	12	7
1223	presurvey_arm_1	2	193	3	1	12	7
1224	presurvey_arm_1	2	193	3	1	13	7
1225	presurvey_arm_1	2	193	3	1	12	7
1226	presurvey_arm_1	2	193	3	1	12	7
1227	presurvey_arm_1	2	193	3	1	12	7
1228	presurvey_arm_1	2	194	2	1	12	7
1229	presurvey_arm_1	2	194	3	1	12	7
1230	presurvey_arm_1	2	194	3	1	12	7
1231	presurvey_arm_1	2	194	3	1	12	7

1232	presurvey_arm_1	2	194	3	1	13	7
1233	presurvey_arm_1	2	194	3	1	12	7
1234	presurvey_arm_1	2	194	3	1	12	7
1235	presurvey_arm_1	2	194	3	1	12	7
1236	presurvey_arm_1	2	194	3	1	12	7
1237	presurvey_arm_1	2	194	3	1	12	7
1238	presurvey_arm_1	2	194	3	1	12	7
1239	presurvey_arm_1	2	194	3	1	12	7
1240	presurvey_arm_1	2	194	3	1	12	7
1241	presurvey_arm_1	2	194	3	1	13	7
1242	presurvey_arm_1	2	194	3	1	12	7
1243	presurvey_arm_1	3	195	3	1	13	8
1244	presurvey_arm_1	3	195	3	1	14	8
1245	presurvey_arm_1	3	195	3	1	14	8
1246	presurvey_arm_1	3	195	3	1	13	8
1247	presurvey_arm_1	3	195	3	1	14	8
1248	presurvey_arm_1	3	195	3	1	13	8
1249	presurvey_arm_1	3	195	3	1	13	8
1250	presurvey_arm_1	3	195	3	1	14	8
1251	presurvey_arm_1	3	195	3	1	13	8
1252	presurvey_arm_1	3	195	3	1	13	8
1253	presurvey_arm_1	3	195	3	1	14	8
1254	presurvey_arm_1	3	195	3	1	13	8
1255	presurvey_arm_1	3	195	3	1	13	8
1256	presurvey_arm_1	3	195	3	1	13	8
1257	presurvey_arm_1	3	195	3	1	14	8
1258	presurvey_arm_1	3	195	3	1	14	8
1452	presurvey_arm_1	1	196	3	1	12	6
1453	presurvey_arm_1	1	196	3	1	12	6
1454	presurvey_arm_1	1	196	3	1	11	6
1455	presurvey_arm_1	1	196	3	1	12	6
1456	presurvey_arm_1	1	196	3	1	12	6
1457	presurvey_arm_1	1	196	3	1	11	6
1458	presurvey_arm_1	1	196	3	1	11	6
1459	presurvey_arm_1	1	196	3	1	11	6
1460	presurvey_arm_1	1	196	3	1	12	6
1461	presurvey_arm_1	1	196	3	1	12	6
1462	presurvey_arm_1	1	196	3	1	12	6
1463	presurvey_arm_1	1	196	3	1	11	6
1464	presurvey_arm_1	1	196	3	1	11	6
1465	presurvey_arm_1	1	196	3	1	12	6
1466	presurvey_arm_1	1	196	3	1	11	6
1467	presurvey_arm_1	1	196	3	1	11	6
1468	presurvey_arm_1	1	196	3	1	11	6
1469	presurvey_arm_1	1	196	3	1	12	6
1470	presurvey_arm_1	1	196	3	1	11	6
1471	presurvey_arm_1	1	196	3	1	11	6
1472	presurvey_arm_1	1	196	3	1	12	6

1473	presurvey_arm_1	1	196	3	1	11	6
1474	presurvey_arm_1	1	196	3	1	11	6
1475	presurvey_arm_1	1	196	3	1	12	6
1476	presurvey_arm_1	1	196	3	1	12	6
1477	presurvey_arm_1	1	196	3	1	11	6
1478	presurvey_arm_1	1	197	3	1	11	6
1479	presurvey_arm_1	1	197	3	1	11	6
1480	presurvey_arm_1	1	197	3	1	11	6
1481	presurvey_arm_1	1	197	3	1	11	6
1482	presurvey_arm_1	1	197	3	1	11	6
1483	presurvey_arm_1	1	197	3	1	12	6
1484	presurvey_arm_1	1	197	3	1	12	6
1485	presurvey_arm_1	1	197	3	1	11	6
1486	presurvey_arm_1	1	197	3	1	11	6
1487	presurvey_arm_1	1	197	3	1	11	6
1488	presurvey_arm_1	1	197	3	1	12	6
1489	presurvey_arm_1	1	197	2	1	11	6
1490	presurvey_arm_1	1	197	3	1	11	6
1491	presurvey_arm_1	1	197	3	1	12	6
1492	presurvey_arm_1	1	197	3	1	11	6
1493	presurvey_arm_1	2	198	3	1	12	7
1494	presurvey_arm_1	2	198	3	1	12	7
1495	presurvey_arm_1	2	198	3	1	13	7
1496	presurvey_arm_1	2	198	3	1	12	7
1497	presurvey_arm_1	2	198	3	1	12	7
1498	presurvey_arm_1	2	198	3	1	13	7
1499	presurvey_arm_1	2	198	3	1	12	7
1500	presurvey_arm_1	2	198	3	1	12	99
1501	presurvey_arm_1	2	198	2	1	12	7
1502	presurvey_arm_1	2	198	3	1	13	7
1503	presurvey_arm_1	2	198	3	1	12	7
1504	presurvey_arm_1	2	198	3	1	12	7
1505	presurvey_arm_1	2	198	3	1	12	7
1506	presurvey_arm_1	2	198	3	1	12	7
1507	presurvey_arm_1	2	198	3	1	12	7
1508	presurvey_arm_1	2	198	3	1	12	7
1509	presurvey_arm_1	2	198	3	1	13	7
1510	presurvey_arm_1	2	198	3	1	13	7
1511	presurvey_arm_1	2	198	3	1	13	7
1512	presurvey_arm_1	2	198	3	1	13	7
1513	presurvey_arm_1	2	198	3	1	12	7
1514	presurvey_arm_1	2	198	3	1	12	7
1515	presurvey_arm_1	2	198	3	1	12	7
1516	presurvey_arm_1	2	199	3	1	12	7
1517	presurvey_arm_1	2	199	3	1	12	7
1518	presurvey_arm_1	2	199	3	1	12	7
1519	presurvey_arm_1	2	199	3	1	13	7
1520	presurvey_arm_1	2	199	3	1	13	7

1521	presurvey_arm_1	2	199	3	1	12	7
1522	presurvey_arm_1	2	199	3	1	12	7
1523	presurvey_arm_1	2	199	3	1	12	7
1524	presurvey_arm_1	2	199	3	1	12	7
1525	presurvey_arm_1	2	199	3	1	12	7
1526	presurvey_arm_1	2	199	3	1	13	7
1527	presurvey_arm_1	2	199	3	1	12	7
1528	presurvey_arm_1	2	199	3	1	12	7
1529	presurvey_arm_1	2	199	3	1	13	7
1530	presurvey_arm_1	2	199	3	1	12	7
1531	presurvey_arm_1	3	200	3	1	14	8
1532	presurvey_arm_1	3	200	3	1	14	8
1533	presurvey_arm_1	3	200	3	1	13	8
1534	presurvey_arm_1	3	200	3	1	13	8
1535	presurvey_arm_1	3	200	3	1	14	8
1536	presurvey_arm_1	3	200	2	1	14	8
1537	presurvey_arm_1	3	200	3	1	14	8
1538	presurvey_arm_1	3	200	3	1	13	8
1539	presurvey_arm_1	3	200	3	1	13	8
1540	presurvey_arm_1	3	200	3	1	13	8
1541	presurvey_arm_1	3	200	3	1	13	8
1542	presurvey_arm_1	3	200	3	1	13	8
1543	presurvey_arm_1	3	200	3	1	14	8
1544	presurvey_arm_1	3	200	3	1	13	8
1545	presurvey_arm_1	3	200	3	1	13	8
1546	presurvey_arm_1	3	200	3	1	13	8
1547	presurvey_arm_1	3	201	3	1	13	8
1548	presurvey_arm_1	3	201	3	1	14	8
1549	presurvey_arm_1	3	201	2	1	13	8
1550	presurvey_arm_1	3	201	3	1	14	8
1551	presurvey_arm_1	3	201	3	1	13	8
1552	presurvey_arm_1	3	201	3	1	14	8
1553	presurvey_arm_1	3	201	3	1	13	8
1554	presurvey_arm_1	3	201	3	1	13	8
1555	presurvey_arm_1	3	201	3	1	15	8
1556	presurvey_arm_1	3	201	3	1	14	8
1557	presurvey_arm_1	3	201	3	1	14	8
1558	presurvey_arm_1	3	201	3	1	13	8
1559	presurvey_arm_1	3	201	3	1	13	8
1560	presurvey_arm_1	3	201	3	1	13	8
1561	presurvey_arm_1	3	201	3	1	13	8
1562	presurvey_arm_1	3	201	3	1	13	8
1563	presurvey_arm_1	3	201	3	1	13	8
1564	presurvey_arm_1	3	201	3	1	13	8
1565	presurvey_arm_1	3	201	3	1	13	8
1566	presurvey_arm_1	3	201	3	1	14	8
1567	presurvey_arm_1	3	201	3	1	13	8
1568	presurvey_arm_1	3	201	3	1	14	8

1569	presurvey_arm_1	3	201	3	1	13	8
1570	presurvey_arm_1	3	201	3	1	13	8
1571	presurvey_arm_1	3	201	3	1	14	8
1572	presurvey_arm_1	3	201	3	1	13	8
1573	presurvey_arm_1	3	201	3	1	10	6
1574	presurvey_arm_1	3	201	3	1	14	8
1575	presurvey_arm_1	3	201	3	1	14	8
1576	presurvey_arm_1	3	201	3	1	14	8
1705	presurvey_arm_1	1	205	3	1	11	6
1706	presurvey_arm_1	1	205	3	1	12	6
1707	presurvey_arm_1	1	205	3	1	11	6
1708	presurvey_arm_1	1	205	3	1	12	6
1709	presurvey_arm_1	1	205	3	1	11	6
1710	presurvey_arm_1	1	205	3	1	11	6
1711	presurvey_arm_1	1	205	3	1	11	6
1712	presurvey_arm_1	1	205	3	1	11	6
1713	presurvey_arm_1	1	205	3	1	11	6
1714	presurvey_arm_1	1	205	3	1	11	6
1715	presurvey_arm_1	1	205	3	1	11	6
1716	presurvey_arm_1	1	205	3	1	12	6
1717	presurvey_arm_1	1	205	3	1	12	6
1718	presurvey_arm_1	1	205	3	1	11	6
1719	presurvey_arm_1	1	205	3	1	11	6
1720	presurvey_arm_1	1	205	3	1	12	6
1721	presurvey_arm_1	1	205	3	1	11	6
1722	presurvey_arm_1	1	205	3	1	11	6
1723	presurvey_arm_1	1	205	3	1	12	7
1724	presurvey_arm_1	1	205	3	1	11	6
1725	presurvey_arm_1	1	205	3	1	12	6
1726	presurvey_arm_1	1	205	3	1	12	6
1727	presurvey_arm_1	1	205	3	1	12	6
1728	presurvey_arm_1	1	205	3	1	12	6
1729	presurvey_arm_1	1	205	3	1	11	6
1730	presurvey_arm_1	1	205	3	1	12	6
1731	presurvey_arm_1	1	205	3	1	12	6
1732	presurvey_arm_1	1	205	3	1	11	6
1733	presurvey_arm_1	1	206	3	1	12	6
1734	presurvey_arm_1	1	206	3	1	11	6
1735	presurvey_arm_1	1	206	3	1	11	6
1736	presurvey_arm_1	1	206	3	1	12	6
1737	presurvey_arm_1	1	206	3	1	11	6
1738	presurvey_arm_1	1	206	3	1	12	6
1739	presurvey_arm_1	1	206	3	1	12	6
1740	presurvey_arm_1	1	206	3	1	12	6
1741	presurvey_arm_1	1	206	3	1	12	6
1742	presurvey_arm_1	1	206	3	1	11	6
1743	presurvey_arm_1	1	206	3	1	11	6
1744	presurvey_arm_1	1	206	3	1	11	6

1745	presurvey_arm_1	1	206	3	1	11	6
1746	presurvey_arm_1	1	206	3	1	12	6
1747	presurvey_arm_1	1	206	3	1	12	6
1748	presurvey_arm_1	1	206	3	1	12	6
1749	presurvey_arm_1	1	206	3	1	11	6
1750	presurvey_arm_1	1	206	3	1	11	6
1751	presurvey_arm_1	1	206	3	1	12	6
1752	presurvey_arm_1	1	206	3	1	12	6
1753	presurvey_arm_1	1	206	3	1	11	6
1754	presurvey_arm_1	1	206	3	1	12	6
1755	presurvey_arm_1	1	206	3	1	11	6
1756	presurvey_arm_1	2	207	3	1	13	7
1757	presurvey_arm_1	2	207	3	1	13	7
1758	presurvey_arm_1	2	207	3	1	12	7
1759	presurvey_arm_1	2	207	3	1	13	7
1760	presurvey_arm_1	2	207	3	1	13	7
1761	presurvey_arm_1	2	207	3	1	12	7
1762	presurvey_arm_1	2	207	3	1	13	7
1763	presurvey_arm_1	2	207	3	1	12	7
1764	presurvey_arm_1	2	207	3	1	12	7
1765	presurvey_arm_1	2	207	3	1	13	7
1766	presurvey_arm_1	2	207	3	1	13	7
1767	presurvey_arm_1	2	207	3	1	13	7
1768	presurvey_arm_1	2	207	3	1	12	7
1769	presurvey_arm_1	2	207	3	1	12	7
1770	presurvey_arm_1	2	207	3	1	12	7
1771	presurvey_arm_1	2	207	3	1	13	7
1772	presurvey_arm_1	2	207	3	1	12	7
1773	presurvey_arm_1	2	207	3	1	13	7
1774	presurvey_arm_1	2	207	3	1	12	7
1775	presurvey_arm_1	2	207	3	1	13	7
1776	presurvey_arm_1	2	207	3	1	13	7
1777	presurvey_arm_1	2	207	3	1	13	7
1778	presurvey_arm_1	2	207	3	1	14	7
1779	presurvey_arm_1	2	207	3	1	12	7
1780	presurvey_arm_1	2	207	3	1	12	7
1781	presurvey_arm_1	2	208	3	1	13	7
1782	presurvey_arm_1	2	208	3	1	13	7
1783	presurvey_arm_1	2	208	3	1	13	7
1784	presurvey_arm_1	2	208	3	1	13	7
1785	presurvey_arm_1	2	208	3	1	13	7
1786	presurvey_arm_1	2	208	3	1	12	7
1787	presurvey_arm_1	2	208	3	1	13	7
1788	presurvey_arm_1	2	208	3	1	12	7
1789	presurvey_arm_1	2	208	3	1	13	7
1790	presurvey_arm_1	2	208	3	1	12	7
1791	presurvey_arm_1	2	208	3	1	13	7
1792	presurvey_arm_1	2	208	3	1	13	7

1793	presurvey_arm_1	2	208	3	1	13	7
1794	presurvey_arm_1	2	208	3	1	12	7
1795	presurvey_arm_1	2	208	3	1	13	7
1796	presurvey_arm_1	2	208	3	1	13	7
1797	presurvey_arm_1	3	209	3	1	14	8
1798	presurvey_arm_1	3	209	3	1	13	8
1799	presurvey_arm_1	3	209	3	1	14	8
1800	presurvey_arm_1	3	209	3	1	14	8
1801	presurvey_arm_1	3	209	3	1	13	8
1802	presurvey_arm_1	3	209	3	1	13	8
1803	presurvey_arm_1	3	209	3	1	14	8
1804	presurvey_arm_1	3	209	3	1	13	8
1805	presurvey_arm_1	3	209	3	1	14	8
1806	presurvey_arm_1	3	209	3	1	14	8
1807	presurvey_arm_1	3	209	3	1	13	8
1808	presurvey_arm_1	3	209	3	1	14	8
1809	presurvey_arm_1	3	209	3	1	14	8
1810	presurvey_arm_1	3	209	3	1	13	8
1811	presurvey_arm_1	3	209	3	1	14	8
1812	presurvey_arm_1	3	209	3	1	14	8
1813	presurvey_arm_1	3	209	3	1	14	8
1814	presurvey_arm_1	3	210	3	1	14	8
1815	presurvey_arm_1	3	210	3	1	14	8
1816	presurvey_arm_1	3	210	3	1	14	8
1817	presurvey_arm_1	3	210	3	1	14	8
1818	presurvey_arm_1	3	210	3	1	13	8
1819	presurvey_arm_1	3	210	3	1	14	8
1820	presurvey_arm_1	3	210	3	1	13	8
1821	presurvey_arm_1	3	210	3	1	14	8
1822	presurvey_arm_1	3	210	3	1	14	8
1823	presurvey_arm_1	3	210	3	1	14	8
1824	presurvey_arm_1	3	210	3	1	14	8
1825	presurvey_arm_1	3	210	3	1	14	8
1826	presurvey_arm_1	3	210	3	1	14	8
1827	presurvey_arm_1	3	210	3	1	13	8
1828	presurvey_arm_1	3	210	3	1	14	8
1829	presurvey_arm_1	3	210	3	1	14	8
1830	presurvey_arm_1	3	210	3	1	13	8
1831	presurvey_arm_1	3	210	3	1	13	8
1832	presurvey_arm_1	3	210	3	1	15	8
1833	presurvey_arm_1	3	210	3	1	14	8
1834	presurvey_arm_1	3	210	3	1	15	8
1835	presurvey_arm_1	3	210	3	1	14	8
1836	presurvey_arm_1	3	210	3	1	14	8
1837	presurvey_arm_1	3	210	3	1	13	8
1838	presurvey_arm_1	3	210	3	1	14	8
1839	presurvey_arm_1	3	210	3	1	13	99
1840	presurvey_arm_1	1	211	3	1	12	6

1841	presurvey_arm_1	1	211	3	1	11	6
1842	presurvey_arm_1	1	211	3	1	12	6
1843	presurvey_arm_1	1	211	3	1	12	6
1844	presurvey_arm_1	1	211	4	1	11	6
1845	presurvey_arm_1	1	211	3	1	11	6
1846	presurvey_arm_1	1	211	3	1	11	6
1847	presurvey_arm_1	1	211	3	1	12	6
1848	presurvey_arm_1	1	211	3	1	12	6
1849	presurvey_arm_1	1	212	3	1	12	6
1850	presurvey_arm_1	1	212	3	1	12	6
1851	presurvey_arm_1	1	212	3	1	11	6
1852	presurvey_arm_1	1	212	3	1	12	6
1853	presurvey_arm_1	1	212	4	1	12	6
1854	presurvey_arm_1	1	212	3	1	11	6
1855	presurvey_arm_1	1	212	3	1	12	99
1856	presurvey_arm_1	1	212	3	1	11	6
1857	presurvey_arm_1	1	212	3	1	12	6
1858	presurvey_arm_1	1	212	4	1	13	6
1859	presurvey_arm_1	1	212	3	1	11	6
1860	presurvey_arm_1	1	212	3	1	12	6
1861	presurvey_arm_1	1	212	3	1	11	6
1862	presurvey_arm_1	1	212	3	1	11	6
1863	presurvey_arm_1	1	212	3	1	11	6
1864	presurvey_arm_1	1	212	4	1	12	6
1865	presurvey_arm_1	1	212	3	1	12	6
1866	presurvey_arm_1	1	212	4	1	12	6
1867	presurvey_arm_1	1	212	3	1	11	6
1868	presurvey_arm_1	1	212	3	1	12	6
1869	presurvey_arm_1	1	212	3	1	12	6
1870	presurvey_arm_1	1	212	3	1	11	6
1871	presurvey_arm_1	1	212	4	1	11	6
1872	presurvey_arm_1	2	213	3	1	13	7
1873	presurvey_arm_1	2	213	3	1	12	7
1874	presurvey_arm_1	2	213	3	1	12	7
1875	presurvey_arm_1	2	213	3	1	13	7
1876	presurvey_arm_1	2	213	3	1	13	7
1877	presurvey_arm_1	2	213	4	1	13	7
1878	presurvey_arm_1	2	213	3	1	13	7
1879	presurvey_arm_1	2	213	4	1	12	7
1880	presurvey_arm_1	2	213	3	1	13	7
1881	presurvey_arm_1	2	213	3	1	12	7
1882	presurvey_arm_1	2	213	3	1	13	7
1883	presurvey_arm_1	2	214	3	1	14	7
1884	presurvey_arm_1	2	214	3	1	13	7
1885	presurvey_arm_1	2	214	3	1	13	7
1886	presurvey_arm_1	2	214	3	1	13	7
1887	presurvey_arm_1	2	214	4	1	12	7
1888	presurvey_arm_1	2	214	3	1	13	7

1889	presurvey_arm_1	2	214	3	1	12	7
1890	presurvey_arm_1	2	214	3	1	12	7
1891	presurvey_arm_1	2	214	3	1	13	7
1892	presurvey_arm_1	2	214	3	1	12	7
1893	presurvey_arm_1	2	214	3	1	13	7
1894	presurvey_arm_1	2	214	3	1	12	7
1895	presurvey_arm_1	2	214	4	1	12	7
1896	presurvey_arm_1	2	214	3	1	12	7
1897	presurvey_arm_1	2	214	3	1	12	7
1898	presurvey_arm_1	2	214	3	1	13	7
1899	presurvey_arm_1	2	214	3	1	16	6
1900	presurvey_arm_1	2	214	3	1	12	7
1901	presurvey_arm_1	2	214	3	1	13	7
1902	presurvey_arm_1	2	214	3	1	12	7
1903	presurvey_arm_1	3	215	4	1	13	8
1904	presurvey_arm_1	3	215	3	1	14	8
1905	presurvey_arm_1	3	215	3	1	14	8
1906	presurvey_arm_1	3	215	4	1	14	8
1907	presurvey_arm_1	3	215	3	1	14	8
1908	presurvey_arm_1	3	215	3	1	14	8
1909	presurvey_arm_1	3	215	3	1	14	8
1910	presurvey_arm_1	3	215	3	1	14	8
1911	presurvey_arm_1	3	215	3	1	14	8
1912	presurvey_arm_1	3	215	3	1	13	8
1913	presurvey_arm_1	3	215	3	1	13	8
1914	presurvey_arm_1	3	215	3	1	13	8
1915	presurvey_arm_1	3	215	3	1	15	8
1916	presurvey_arm_1	3	215	3	1	14	8
1917	presurvey_arm_1	3	216	3	1	13	8
1918	presurvey_arm_1	3	216	3	1	14	8
1919	presurvey_arm_1	3	216	3	1	14	8
1920	presurvey_arm_1	3	216	3	1	14	8
1921	presurvey_arm_1	3	216	3	1	14	8
1922	presurvey_arm_1	3	126	3	1	13	8
1923	presurvey_arm_1	3	216	3	1	14	8
1924	presurvey_arm_1	3	216	3	1	14	8
1925	presurvey_arm_1	3	216	3	1	14	8
1926	presurvey_arm_1	3	216	3	1	14	8
1927	presurvey_arm_1	3	216	3	1	14	8
1928	presurvey_arm_1	3	216	4	1	13	8
1929	presurvey_arm_1	3	216	3	1	14	8
1930	presurvey_arm_1	3	216	3	1	13	8
1931	presurvey_arm_1	3	216	3	1	13	8
1932	presurvey_arm_1	3	216	3	1	14	8
1933	presurvey_arm_1	3	216	3	1	14	8
1934	presurvey_arm_1	3	216	3	1	13	99
1935	presurvey_arm_1	3	216	3	1	14	8
1936	presurvey_arm_1	3	216	3	1	14	8

1937	presurvey_arm_1	3	216	3	1	14	8
1938	presurvey_arm_1	3	216	3	1	13	8
1939	presurvey_arm_1	3	216	4	1	14	8
1940	presurvey_arm_1	3	216	3	1	13	8
1941	presurvey_arm_1	3	216	3	1	14	99
1942	presurvey_arm_1	3	216	4	1	14	8
1943	presurvey_arm_1	3	216	3	1	13	8
1944	presurvey_arm_1	3	216	3	1	15	8
1945	presurvey_arm_1	3	216	4	1	14	8
1946	presurvey_arm_1	3	216	4	1	13	8
1947	presurvey_arm_1	1	223	4	1	12	6
1948	presurvey_arm_1	1	223	3	1	12	6
1949	presurvey_arm_1	1	223	3	1	11	6
1950	presurvey_arm_1	1	223	3	1	13	6
1951	presurvey_arm_1	1	223	4	1	12	6
1952	presurvey_arm_1	1	223	3	1	11	6
1953	presurvey_arm_1	1	223	3	1	12	6
1954	presurvey_arm_1	1	223	4	1	12	6
1955	presurvey_arm_1	1	223	3	1	12	6
1956	presurvey_arm_1	1	223	4	1	12	6
1957	presurvey_arm_1	1	223	4	1	11	6
1958	presurvey_arm_1	1	224	4	1	12	6
1959	presurvey_arm_1	1	224	4	1	11	6
1960	presurvey_arm_1	1	224	3	1	12	6
1961	presurvey_arm_1	1	224	4	1	11	6
1962	presurvey_arm_1	1	224	3	1	13	6
1963	presurvey_arm_1	1	224	3	1	12	6
1964	presurvey_arm_1	1	224	3	1	12	6
1965	presurvey_arm_1	1	224	4	1	12	6
1966	presurvey_arm_1	1	224	3	1	11	6
1967	presurvey_arm_1	1	224	4	1	12	6
1968	presurvey_arm_1	1	224	4	1	11	6
1969	presurvey_arm_1	1	224	3	1	12	6
1970	presurvey_arm_1	1	224	4	1	12	6
1971	presurvey_arm_1	1	224	3	1	12	6
1972	presurvey_arm_1	1	224	3	1	11	6
1973	presurvey_arm_1	1	224	4	1	12	6
1974	presurvey_arm_1	1	224	4	1	12	6
1975	presurvey_arm_1	1	224	3	1	11	6
1976	presurvey_arm_1	1	224	4	1	11	6
1977	presurvey_arm_1	1	224	4	1	12	6
1978	presurvey_arm_1	1	224	3	1	12	6
1979	presurvey_arm_1	1	224	4	1	12	6
1980	presurvey_arm_1	1	224	3	1	12	6
1981	presurvey_arm_1	1	224	3	1	12	6
1982	presurvey_arm_1	1	224	3	1	11	6
1983	presurvey_arm_1	1	224	3	1	12	6
1984	presurvey_arm_1	1	224	3	1	12	6

1985	presurvey_arm_1	2	225	3	1	13	7
1986	presurvey_arm_1	2	225	3	1	13	7
1987	presurvey_arm_1	2	225	4	1	13	7
1988	presurvey_arm_1	2	225	3	1	13	7
1989	presurvey_arm_1	2	225	4	1	12	7
1990	presurvey_arm_1	2	225	4	1	12	7
1991	presurvey_arm_1	2	225	4	1	13	7
1992	presurvey_arm_1	2	225	4	1	12	7
1993	presurvey_arm_1	2	225	4	1	14	7
1994	presurvey_arm_1	2	225	4	1	13	7
1995	presurvey_arm_1	2	225	3	1	13	7
1996	presurvey_arm_1	2	225	4	1	12	7
1997	presurvey_arm_1	2	225	3	1	13	7
1998	presurvey_arm_1	2	225	4	1	13	7
1999	presurvey_arm_1	2	225	4	1	13	7
2000	presurvey_arm_1	2	226	4	1	13	7
2001	presurvey_arm_1	2	226	3	1	14	7
2002	presurvey_arm_1	2	226	3	1	12	7
2003	presurvey_arm_1	2	226	3	1	13	7
2004	presurvey_arm_1	2	226	3	1	13	7
2005	presurvey_arm_1	2	226	4	1	12	7
2006	presurvey_arm_1	2	226	3	1	13	7
2007	presurvey_arm_1	2	226	3	1	13	7
2008	presurvey_arm_1	2	226	3	1	13	7
2009	presurvey_arm_1	2	226	4	1	13	7
2010	presurvey_arm_1	2	226	4	1	14	7
2011	presurvey_arm_1	2	226	3	1	13	7
2012	presurvey_arm_1	2	226	3	1	13	7
2013	presurvey_arm_1	2	226	4	1	12	7
2014	presurvey_arm_1	2	226	3	1	12	7
2015	presurvey_arm_1	2	226	3	1	13	7
2016	presurvey_arm_1	2	226	4	1	13	7
2017	presurvey_arm_1	2	226	4	1	13	7
2018	presurvey_arm_1	2	226	3	1	13	7
2019	presurvey_arm_1	2	226	3	1	13	7
2020	presurvey_arm_1	3	227	4	1	13	8
2021	presurvey_arm_1	3	227	3	1	14	8
2022	presurvey_arm_1	3	227	4	1	14	8
2023	presurvey_arm_1	3	227	3	1	14	8
2024	presurvey_arm_1	3	227	3	1	14	8
2025	presurvey_arm_1	3	227	3	1	14	8
2026	presurvey_arm_1	3	227	3	1	15	8
2027	presurvey_arm_1	3	227	3	1	14	8
2028	presurvey_arm_1	3	227	3	1	14	8
2029	presurvey_arm_1	3	227	3	1	14	8
2030	presurvey_arm_1	3	227	3	1	13	8
2031	presurvey_arm_1	3	227	4	1	14	8
2032	presurvey_arm_1	3	227	4	1	14	8

2033	presurvey_arm_1	3	227	4	1	14	8
2034	presurvey_arm_1	3	228	4	1	14	8
2035	presurvey_arm_1	3	228	3	1	13	8
2036	presurvey_arm_1	3	228	4	1	14	8
2037	presurvey_arm_1	3	228	4	1	13	8
2038	presurvey_arm_1	3	228	4	1	14	8
2039	presurvey_arm_1	3	228	4	1	14	8
2040	presurvey_arm_1	3	228	4	1	14	8
2041	presurvey_arm_1	3	228	4	1	14	8
2042	presurvey_arm_1	3	228	3	1	14	8
2043	presurvey_arm_1	3	228	3	1	14	8
2044	presurvey_arm_1	3	228	3	1	14	8
2045	presurvey_arm_1	3	228	4	1	14	8
2046	presurvey_arm_1	3	228	4	1	13	8
2047	presurvey_arm_1	3	228	4	1	14	8
2048	presurvey_arm_1	3	228	4	1	14	8
2049	presurvey_arm_1	3	228	4	1	13	8
2050	presurvey_arm_1	3	228	3	1	13	8
2051	presurvey_arm_1	3	228	4	1	14	8
2052	presurvey_arm_1	3	228	3	1	14	8
2053	presurvey_arm_1	3	228	3	1	14	8
2054	presurvey_arm_1	3	228	3	1	14	8
2055	presurvey_arm_1	3	228	3	1	14	99
2056	presurvey_arm_1	3	228	4	1	15	8
2057	presurvey_arm_1	3	228	3	1	14	8
2058	presurvey_arm_1	3	228	3	1	14	8
2059	presurvey_arm_1	3	228	4	1	13	8
2060	presurvey_arm_1	3	228	4	1	13	8
2417	presurvey_arm_1	1	120	1	1	11	6
2418	presurvey_arm_1	1	120	2	1	11	6
2419	presurvey_arm_1	1	120	2	1	11	6
2420	presurvey_arm_1	1	120	2	1	11	6
2421	presurvey_arm_1	1	120	2	1	11	6
2422	presurvey_arm_1	1	120	2	1	11	6
2423	presurvey_arm_1	1	120	2	1	11	6
2424	presurvey_arm_1	1	120	2	1	11	6
2425	presurvey_arm_1	1	120	2	1	11	6
2426	presurvey_arm_1	1	120	1	1	11	6
2427	presurvey_arm_1	1	120	2	1	11	6
2428	presurvey_arm_1	1	120	2	1	11	6
2429	presurvey_arm_1	1	120	2	1	11	6
2430	presurvey_arm_1	1	120	1	1	11	6
2431	presurvey_arm_1	1	120	2	1	11	6
2432	presurvey_arm_1	1	121	2	1	11	6
2433	presurvey_arm_1	1	121	1	1	11	6
2434	presurvey_arm_1	1	121	2	1	11	6
2435	presurvey_arm_1	1	121	2	1	11	6
2436	presurvey_arm_1	1	121	2	1	12	6

2437	presurvey_arm_1	1	121	2	1	11	6
2438	presurvey_arm_1	1	121	2	1	11	6
2439	presurvey_arm_1	1	121	2	1	12	6
2440	presurvey_arm_1	1	121	2	1	11	6
2441	presurvey_arm_1	1	121	2	1	11	6
2442	presurvey_arm_1	1	121	2	1	11	6
2443	presurvey_arm_1	1	121	2	1	11	6
2444	presurvey_arm_1	1	121	2	1	11	6
2445	presurvey_arm_1	1	121	2	1	12	6
2446	presurvey_arm_1	1	121	2	1	11	6
2447	presurvey_arm_1	1	121	2	1	11	6
2448	presurvey_arm_1	1	121	1	1	11	6
2449	presurvey_arm_1	1	121	2	1	10	7
2450	presurvey_arm_1	1	121	2	1	11	6
2451	presurvey_arm_1	1	121	2	1	11	6
2452	presurvey_arm_1	1	121	2	1	11	6
2453	presurvey_arm_1	1	121	2	1	12	6
2454	presurvey_arm_1	1	121	2	1	11	6
2455	presurvey_arm_1	1	121	2	1	12	6
2456	presurvey_arm_1	2	122	2	1	12	7
2457	presurvey_arm_1	2	122	2	1	11	7
2458	presurvey_arm_1	2	122	2	1	12	7
2459	presurvey_arm_1	2	122	2	1	12	7
2460	presurvey_arm_1	2	122	2	1	12	7
2461	presurvey_arm_1	2	122	2	1	13	7
2462	presurvey_arm_1	2	122	2	1	12	7
2463	presurvey_arm_1	2	122	2	1	12	7
2464	presurvey_arm_1	2	122	2	1	12	7
2465	presurvey_arm_1	2	122	2	1	12	7
2466	presurvey_arm_1	2	122	2	1	12	7
2467	presurvey_arm_1	2	122	2	1	12	7
2468	presurvey_arm_1	2	122	2	1	13	7
2469	presurvey_arm_1	2	122	2	1	12	7
2470	presurvey_arm_1	2	122	2	1	13	7
2471	presurvey_arm_1	2	122	2	1	13	7
2472	presurvey_arm_1	2	122	2	1	12	7
2473	presurvey_arm_1	2	122	2	1	13	7
2474	presurvey_arm_1	2	122	2	1	13	7
2475	presurvey_arm_1	2	123	2	1	12	7
2476	presurvey_arm_1	2	123	2	1	12	7
2477	presurvey_arm_1	2	123	2	1	12	7
2478	presurvey_arm_1	2	123	2	1	12	7
2479	presurvey_arm_1	2	123	2	1	12	7
2480	presurvey_arm_1	2	123	2	1	12	7
2481	presurvey_arm_1	2	123	2	1	12	7
2482	presurvey_arm_1	2	123	1	1	13	7
2483	presurvey_arm_1	2	123	2	1	12	7
2484	presurvey_arm_1	2	123	1	1	12	7

2485	presurvey_arm_1	2	123	2	1	12	7
2486	presurvey_arm_1	2	123	2	1	12	7
2487	presurvey_arm_1	2	123	2	1	13	7
2488	presurvey_arm_1	2	123	2	1	12	7
2489	presurvey_arm_1	2	123	2	1	12	7
2490	presurvey_arm_1	2	123	2	1	12	7
2491	presurvey_arm_1	2	123	2	1	12	7
2492	presurvey_arm_1	2	123	2	1	12	7
2493	presurvey_arm_1	2	123	2	1	12	7
2494	presurvey_arm_1	2	123	1	1	13	7
2495	presurvey_arm_1	2	123	2	1	13	7
2496	presurvey_arm_1	3	124	2	1	13	8
2497	presurvey_arm_1	3	124	2	1	13	8
2498	presurvey_arm_1	3	124	2	1	14	8
2499	presurvey_arm_1	3	124	2	1	13	8
2500	presurvey_arm_1	3	124	2	1	13	8
2501	presurvey_arm_1	3	124	2	1	14	8
2502	presurvey_arm_1	3	124	1	1	13	8
2503	presurvey_arm_1	3	124	2	1	13	8
2504	presurvey_arm_1	3	124	2	1	13	8
2505	presurvey_arm_1	3	124	1	1	13	8
2506	presurvey_arm_1	3	124	2	1	13	8
2507	presurvey_arm_1	3	124	2	1	13	8
2508	presurvey_arm_1	3	124	1	1	13	8
2509	presurvey_arm_1	3	124	2	1	14	8
2510	presurvey_arm_1	3	124	2	1	13	8
2511	presurvey_arm_1	3	124	2	1	15	8
2512	presurvey_arm_1	3	125	2	1	14	8
2513	presurvey_arm_1	3	125	2	1	13	8
2514	presurvey_arm_1	3	125	2	1	13	8
2515	presurvey_arm_1	3	125	2	1	13	8
2516	presurvey_arm_1	3	125	2	1	13	8
2517	presurvey_arm_1	3	125	2	1	13	8
2518	presurvey_arm_1	3	125	2	1	14	8
2519	presurvey_arm_1	3	125	2	1	13	8
2520	presurvey_arm_1	3	125	2	1	14	8
2521	presurvey_arm_1	3	125	2	1	13	8
2522	presurvey_arm_1	3	125	2	1	14	8
2523	presurvey_arm_1	3	125	2	1	13	8
2524	presurvey_arm_1	3	125	2	1	13	8
2525	presurvey_arm_1	3	125	2	1	13	8
2526	presurvey_arm_1	3	125	2	1	14	8
2527	presurvey_arm_1	3	125	2	1	13	8
2528	presurvey_arm_1	3	125	2	1	13	8
2529	presurvey_arm_1	3	125	2	1	13	8
2530	presurvey_arm_1	3	125	2	1	14	8
2531	presurvey_arm_1	3	125	2	1	13	8
2532	presurvey_arm_1	3	125	2	1	13	8

2533	presurvey_arm_1	1	130	2	1	11	6
2534	presurvey_arm_1	1	130	2	1	11	6
2535	presurvey_arm_1	1	130	2	1	11	6
2536	presurvey_arm_1	1	130	2	1	11	6
2537	presurvey_arm_1	1	130	2	1	11	6
2538	presurvey_arm_1	1	130	2	1	11	6
2539	presurvey_arm_1	1	130	2	1	12	6
2540	presurvey_arm_1	1	130	2	1	11	6
2541	presurvey_arm_1	1	130	2	1	11	6
2542	presurvey_arm_1	1	130	2	1	12	6
2543	presurvey_arm_1	1	130	2	1	11	6
2544	presurvey_arm_1	1	131	2	1	11	6
2545	presurvey_arm_1	1	131	2	1	11	6
2546	presurvey_arm_1	1	131	2	1	11	6
2547	presurvey_arm_1	1	131	2	1	11	6
2548	presurvey_arm_1	1	131	2	1	11	6
2549	presurvey_arm_1	1	131	2	1	12	6
2550	presurvey_arm_1	1	131	2	1	11	6
2551	presurvey_arm_1	1	131	2	1	11	6
2552	presurvey_arm_1	1	131	2	1	10	6
2553	presurvey_arm_1	1	131	2	1	12	6
2554	presurvey_arm_1	1	131	2	1	10	6
2555	presurvey_arm_1	1	131	2	1	12	6
2556	presurvey_arm_1	1	131	2	1	11	6
2557	presurvey_arm_1	1	131	2	1	11	6
2558	presurvey_arm_1	1	131	2	1	11	6
2559	presurvey_arm_1	1	131	2	1	11	6
2560	presurvey_arm_1	1	131	2	1	11	6
2561	presurvey_arm_1	1	131	2	1	11	6
2562	presurvey_arm_1	1	131	2	1	12	6
2563	presurvey_arm_1	1	131	2	1	12	6
2564	presurvey_arm_1	1	131	2	1	11	6
2565	presurvey_arm_1	1	131	2	1	11	6
2566	presurvey_arm_1	1	131	2	1	11	6
2567	presurvey_arm_1	1	131	2	1	11	6
2568	presurvey_arm_1	1	131	2	1	11	6
2569	presurvey_arm_1	1	131	2	1	11	6
2570	presurvey_arm_1	2	132	1	1	12	7
2571	presurvey_arm_1	2	132	2	1	12	7
2572	presurvey_arm_1	2	132	2	1	12	7
2573	presurvey_arm_1	2	132	2	1	13	7
2574	presurvey_arm_1	2	132	2	1	12	7
2575	presurvey_arm_1	2	132	2	1	12	7
2576	presurvey_arm_1	2	132	2	1	12	7
2577	presurvey_arm_1	2	132	2	1	14	7
2578	presurvey_arm_1	2	132	2	1	12	7
2579	presurvey_arm_1	2	132	2	1	12	7
2580	presurvey_arm_1	2	132	2	1	11	7

2581	presurvey_arm_1	2	132	1	1	13	7
2582	presurvey_arm_1	2	132	2	1	13	7
2583	presurvey_arm_1	2	132	2	1	13	7
2584	presurvey_arm_1	2	132	2	1	13	7
2585	presurvey_arm_1	2	133	2	1	11	7
2586	presurvey_arm_1	2	133	2	1	12	7
2587	presurvey_arm_1	2	133	2	1	12	7
2588	presurvey_arm_1	2	133	2	1	13	7
2589	presurvey_arm_1	2	133	2	1	12	7
2590	presurvey_arm_1	2	133	2	1	13	7
2591	presurvey_arm_1	2	133	2	1	12	7
2592	presurvey_arm_1	2	133	2	1	13	7
2593	presurvey_arm_1	2	133	2	1	12	7
2594	presurvey_arm_1	2	133	2	1	12	7
2595	presurvey_arm_1	2	133	2	1	12	7
2596	presurvey_arm_1	2	133	2	1	12	7
2597	presurvey_arm_1	2	133	2	1	13	7
2598	presurvey_arm_1	2	133	2	1	13	7
2599	presurvey_arm_1	2	133	2	1	13	7
2600	presurvey_arm_1	2	133	2	1	12	7
2601	presurvey_arm_1	2	133	2	1	12	7
2602	presurvey_arm_1	2	133	2	1	12	7
2603	presurvey_arm_1	2	133	2	1	12	7
2604	presurvey_arm_1	2	133	2	1	14	7
2605	presurvey_arm_1	2	133	2	1	12	7
2606	presurvey_arm_1	2	133	2	1	13	7
2607	presurvey_arm_1	2	133	2	1	12	7
2608	presurvey_arm_1	2	133	2	1	12	7
2609	presurvey_arm_1	3	134	2	1	13	8
2610	presurvey_arm_1	3	134	2	1	13	8
2611	presurvey_arm_1	3	134	2	1	13	8
2612	presurvey_arm_1	3	134	2	1	14	8
2613	presurvey_arm_1	3	134	2	1	14	8
2614	presurvey_arm_1	3	134	2	1	13	8
2615	presurvey_arm_1	3	134	2	1	14	8
2616	presurvey_arm_1	3	134	2	1	13	8
2617	presurvey_arm_1	3	134	2	1	14	8
2618	presurvey_arm_1	3	134	2	1	14	8
2619	presurvey_arm_1	3	134	2	1	13	8
2620	presurvey_arm_1	3	134	2	1	13	8
2621	presurvey_arm_1	3	134	2	1	14	8
2622	presurvey_arm_1	3	134	2	1	13	8
2623	presurvey_arm_1	3	134	2	1	13	8
2624	presurvey_arm_1	3	134	2	1	13	8
2625	presurvey_arm_1	3	135	2	1	13	8
2626	presurvey_arm_1	3	135	2	1	13	8
2627	presurvey_arm_1	3	135	2	1	13	8
2628	presurvey_arm_1	3	135	2	1	13	8

2629	presurvey_arm_1	3	135	2	1	13	8
2630	presurvey_arm_1	3	135	2	1	14	8
2631	presurvey_arm_1	3	135	2	1	13	8
2632	presurvey_arm_1	3	135	1	1	13	8
2633	presurvey_arm_1	3	135	2	1	13	8
2634	presurvey_arm_1	3	135	2	1	13	8
2635	presurvey_arm_1	3	135	2	1	13	8
2637	presurvey_arm_1	3	135	2	1	13	8
2638	presurvey_arm_1	3	135	2	1	13	8
2639	presurvey_arm_1	3	135	2	1	13	8
2640	presurvey_arm_1	3	135	2	1	13	8
2641	presurvey_arm_1	3	135	2	1	13	8
2642	presurvey_arm_1	3	135	2	1	13	8
2643	presurvey_arm_1	3	135	2	1	13	8
2644	presurvey_arm_1	3	135	2	1	14	8
2645	presurvey_arm_1	3	135	2	1	13	8
2764	presurvey_arm_1	1	130	2	1	11	6
2867	presurvey_arm_1	1	142	2	1	12	6
2868	presurvey_arm_1	1	142	2	1	11	6
2869	presurvey_arm_1	1	142	2	1	12	6
2870	presurvey_arm_1	1	142	2	1	13	6
2871	presurvey_arm_1	1	142	2	1	11	6
2872	presurvey_arm_1	1	142	2	1	12	6
2873	presurvey_arm_1	1	142	2	1	12	6
2874	presurvey_arm_1	1	142	1	1	12	6
2875	presurvey_arm_1	1	142	2	1	11	99
2876	presurvey_arm_1	1	142	2	1	12	6
2877	presurvey_arm_1	1	142	2	1	12	6
2878	presurvey_arm_1	1	142	2	1	12	6
2879	presurvey_arm_1	1	142	2	1	11	6
2880	presurvey_arm_1	1	142	2	1	12	6
2881	presurvey_arm_1	1	143	2	1	11	6
2882	presurvey_arm_1	1	143	2	1	12	6
2883	presurvey_arm_1	1	143	2	1	11	6
2884	presurvey_arm_1	1	143	2	1	12	6
2885	presurvey_arm_1	1	143	2	1	12	6
2886	presurvey_arm_1	1	143	2	1	12	6
2887	presurvey_arm_1	1	143	2	1	11	99
2888	presurvey_arm_1	1	143	2	1	11	6
2889	presurvey_arm_1	1	143	2	1	11	6
2890	presurvey_arm_1	1	143	2	1	12	6
2891	presurvey_arm_1	1	143	2	1	11	6
2892	presurvey_arm_1	1	143	2	1	11	6
2893	presurvey_arm_1	1	143	2	1	12	6
2894	presurvey_arm_1	1	143	2	1	11	6
2895	presurvey_arm_1	1	143	2	1	11	6
2896	presurvey_arm_1	1	143	1	1	12	6
2897	presurvey_arm_1	1	143	2	1	11	6

2898	presurvey_arm_1	1	143	2	1	11	6
2899	presurvey_arm_1	1	143	2	1	12	6
2900	presurvey_arm_1	1	143	2	1	12	6
2901	presurvey_arm_1	1	143	2	1	99	6
2902	presurvey_arm_1	1	143	2	1	11	6
2903	presurvey_arm_1	1	143	2	1	11	6
2904	presurvey_arm_1	2	144	2	1	13	7
2905	presurvey_arm_1	2	144	2	1	13	7
2906	presurvey_arm_1	2	144	2	1	14	7
2907	presurvey_arm_1	2	144	2	1	12	7
2908	presurvey_arm_1	2	144	2	1	12	7
2909	presurvey_arm_1	2	144	2	1	12	7
2910	presurvey_arm_1	2	144	2	1	12	7
2911	presurvey_arm_1	2	144	2	1	12	7
2912	presurvey_arm_1	2	144	2	1	14	7
2913	presurvey_arm_1	2	144	2	1	12	7
2914	presurvey_arm_1	2	144	2	1	12	7
2915	presurvey_arm_1	2	144	2	1	13	7
2916	presurvey_arm_1	2	144	2	1	13	7
2917	presurvey_arm_1	2	144	2	1	13	7
2918	presurvey_arm_1	2	145	2	1	12	7
2919	presurvey_arm_1	2	145	2	1	12	7
2920	presurvey_arm_1	2	145	2	1	13	7
2921	presurvey_arm_1	2	145	2	1	12	7
2922	presurvey_arm_1	2	145	2	1	14	7
2923	presurvey_arm_1	2	145	2	1	13	7
2924	presurvey_arm_1	2	145	2	1	12	7
2925	presurvey_arm_1	2	145	2	1	13	7
2926	presurvey_arm_1	2	145	2	1	13	7
2927	presurvey_arm_1	2	145	2	1	13	7
2928	presurvey_arm_1	2	145	2	1	13	7
2929	presurvey_arm_1	2	145	2	1	12	7
2930	presurvey_arm_1	2	145	2	1	12	7
2931	presurvey_arm_1	2	145	2	1	12	7
2932	presurvey_arm_1	2	145	2	1	99	7
2933	presurvey_arm_1	2	145	2	1	12	7
2934	presurvey_arm_1	2	145	2	1	12	7
2935	presurvey_arm_1	2	145	2	1	12	7
2936	presurvey_arm_1	2	145	2	1	12	7
2937	presurvey_arm_1	2	145	2	1	13	7
2938	presurvey_arm_1	2	145	2	1	13	7
2939	presurvey_arm_1	2	145	1	1	13	7
2940	presurvey_arm_1	2	145	2	1	12	7
2941	presurvey_arm_1	2	145	2	1	12	7
2942	presurvey_arm_1	3	147	2	1	13	8
2943	presurvey_arm_1	3	147	2	1	14	8
2944	presurvey_arm_1	3	147	2	1	14	8
2945	presurvey_arm_1	3	147	2	1	13	8

2946	presurvey_arm_1	3	147	2	1	14	8
2947	presurvey_arm_1	3	147	2	1	14	8
2948	presurvey_arm_1	3	147	2	1	13	8
2949	presurvey_arm_1	3	147	2	1	13	8
2950	presurvey_arm_1	3	147	2	1	13	8
2951	presurvey_arm_1	3	147	2	1	11	6
2952	presurvey_arm_1	3	147	2	1	13	8
2953	presurvey_arm_1	3	147	2	1	13	8
2954	presurvey_arm_1	3	147	2	1	13	8
2955	presurvey_arm_1	3	147	2	1	13	8
2956	presurvey_arm_1	3	147	2	1	14	8
2957	presurvey_arm_1	3	147	2	1	13	8
2958	presurvey_arm_1	3	147	2	1	13	8
2959	presurvey_arm_1	3	147	1	1	14	8
2960	presurvey_arm_1	3	147	2	1	12	8
2961	presurvey_arm_1	3	147	2	1	14	8
2962	presurvey_arm_1	3	147	2	1	13	8
2963	presurvey_arm_1	3	147	2	1	14	8
2964	presurvey_arm_1	3	148	2	1	14	8
2965	presurvey_arm_1	3	148	2	1	13	8
2966	presurvey_arm_1	3	148	2	1	14	8
2967	presurvey_arm_1	3	148	2	1	14	8
2968	presurvey_arm_1	3	148	2	1	13	8
2969	presurvey_arm_1	3	148	2	1	13	8
2970	presurvey_arm_1	3	148	2	1	14	8
2971	presurvey_arm_1	3	148	2	1	14	8
2972	presurvey_arm_1	3	148	2	1	14	8
2973	presurvey_arm_1	3	148	2	1	13	8
2974	presurvey_arm_1	3	148	2	1	13	8
2975	presurvey_arm_1	3	148	2	1	13	8
2976	presurvey_arm_1	3	148	2	1	14	8
3076	presurvey_arm_1	1	154	2	1	11	6
3077	presurvey_arm_1	1	154	2	1	11	6
3078	presurvey_arm_1	1	154	2	1	11	6
3079	presurvey_arm_1	1	154	2	1	12	6
3080	presurvey_arm_1	1	154	2	1	12	6
3081	presurvey_arm_1	1	154	2	1	12	6
3082	presurvey_arm_1	1	154	2	1	12	6
3083	presurvey_arm_1	1	154	2	1	11	6
3084	presurvey_arm_1	1	154	2	1	12	6
3085	presurvey_arm_1	1	154	2	1	12	6
3086	presurvey_arm_1	1	154	2	1	12	6
3087	presurvey_arm_1	1	154	2	1	11	6
3088	presurvey_arm_1	1	154	2	1	11	6
3089	presurvey_arm_1	1	154	2	1	11	6
3090	presurvey_arm_1	1	154	2	1	12	6
3091	presurvey_arm_1	1	154	2	1	11	6
3092	presurvey_arm_1	1	155	2	1	11	6

3093	presurvey_arm_1	1	155	2	1	12	6
3094	presurvey_arm_1	1	155	2	1	12	6
3095	presurvey_arm_1	1	155	2	1	12	6
3096	presurvey_arm_1	1	155	2	1	12	6
3097	presurvey_arm_1	1	155	2	1	12	6
3098	presurvey_arm_1	1	155	2	1	11	6
3099	presurvey_arm_1	1	155	2	1	13	6
3100	presurvey_arm_1	1	155	2	1	11	6
3101	presurvey_arm_1	1	155	2	1	11	6
3102	presurvey_arm_1	1	155	2	1	11	6
3103	presurvey_arm_1	1	155	2	1	12	6
3104	presurvey_arm_1	1	155	2	1	99	99
3105	presurvey_arm_1	1	155	2	1	11	6
3106	presurvey_arm_1	1	155	2	1	12	6
3107	presurvey_arm_1	1	155	2	1	12	6
3108	presurvey_arm_1	1	155	2	1	12	6
3109	presurvey_arm_1	1	155	2	1	12	6
3110	presurvey_arm_1	1	155	2	1	12	6
3111	presurvey_arm_1	1	155	2	1	11	6
3112	presurvey_arm_1	1	155	2	1	12	6
3113	presurvey_arm_1	1	155	2	1	11	6
3114	presurvey_arm_1	1	155	2	1	11	6
3115	presurvey_arm_1	2	156	2	1	12	7
3116	presurvey_arm_1	2	156	2	1	14	7
3117	presurvey_arm_1	2	156	2	1	13	7
3118	presurvey_arm_1	2	156	2	1	13	7
3119	presurvey_arm_1	2	156	2	1	12	7
3120	presurvey_arm_1	2	156	2	1	13	7
3121	presurvey_arm_1	2	156	2	1	12	7
3122	presurvey_arm_1	2	156	2	1	12	7
3123	presurvey_arm_1	2	156	2	1	13	7
3124	presurvey_arm_1	2	156	2	1	14	7
3125	presurvey_arm_1	2	156	2	1	12	7
3126	presurvey_arm_1	2	156	2	1	13	7
3127	presurvey_arm_1	2	156	2	1	12	7
3128	presurvey_arm_1	2	156	2	1	13	7
3129	presurvey_arm_1	2	156	2	1	12	7
3130	presurvey_arm_1	2	156	2	1	12	7
3131	presurvey_arm_1	2	157	2	1	13	7
3132	presurvey_arm_1	2	157	2	1	13	7
3133	presurvey_arm_1	2	157	2	1	12	7
3134	presurvey_arm_1	2	157	2	1	13	7
3135	presurvey_arm_1	2	157	2	1	14	7
3136	presurvey_arm_1	2	157	2	1	13	7
3137	presurvey_arm_1	2	157	2	1	11	7
3138	presurvey_arm_1	2	157	2	1	12	7
3139	presurvey_arm_1	2	157	2	1	12	7
3140	presurvey_arm_1	2	157	2	1	13	7

3141	presurvey_arm_1	2	157	2	1	13	7
3142	presurvey_arm_1	2	157	2	1	13	7
3143	presurvey_arm_1	2	157	2	1	12	7
3144	presurvey_arm_1	2	157	2	1	12	7
3145	presurvey_arm_1	2	157	2	1	12	7
3146	presurvey_arm_1	2	157	2	1	13	6
3147	presurvey_arm_1	2	157	2	1	13	7
3148	presurvey_arm_1	2	157	2	1	13	7
3149	presurvey_arm_1	2	157	2	1	13	7
3150	presurvey_arm_1	2	157	2	1	13	7
3151	presurvey_arm_1	2	157	2	1	14	7
3152	presurvey_arm_1	3	158	2	1	14	8
3153	presurvey_arm_1	3	158	2	1	13	8
3154	presurvey_arm_1	3	158	2	1	14	8
3155	presurvey_arm_1	3	158	2	1	13	8
3156	presurvey_arm_1	3	158	2	1	13	8
3157	presurvey_arm_1	3	158	2	1	14	8
3158	presurvey_arm_1	3	158	2	1	14	8
3159	presurvey_arm_1	3	158	2	1	13	8
3160	presurvey_arm_1	3	158	2	1	14	8
3161	presurvey_arm_1	3	158	2	1	13	8
3162	presurvey_arm_1	3	158	2	1	13	8
3163	presurvey_arm_1	3	158	2	1	14	8
3164	presurvey_arm_1	3	158	2	1	14	8
3165	presurvey_arm_1	3	158	2	1	13	8
3166	presurvey_arm_1	3	158	2	1	14	8
3167	presurvey_arm_1	3	158	2	1	13	8
3168	presurvey_arm_1	3	158	2	1	13	8
3169	presurvey_arm_1	3	158	2	1	13	8
3170	presurvey_arm_1	3	159	2	1	14	8
3171	presurvey_arm_1	3	159	2	1	14	8
3172	presurvey_arm_1	3	159	2	1	13	8
3173	presurvey_arm_1	3	159	2	1	14	8
3174	presurvey_arm_1	3	159	2	1	13	8
3175	presurvey_arm_1	3	159	2	1	14	8
3176	presurvey_arm_1	3	159	2	1	13	8
3177	presurvey_arm_1	3	159	2	1	14	8
3178	presurvey_arm_1	3	159	2	1	13	8
3179	presurvey_arm_1	3	159	2	1	13	8
3180	presurvey_arm_1	3	159	2	1	13	8
3181	presurvey_arm_1	3	159	2	1	14	8
3182	presurvey_arm_1	3	159	2	1	13	8
3183	presurvey_arm_1	3	159	2	1	14	8
3184	presurvey_arm_1	3	159	2	1	13	8
3185	presurvey_arm_1	3	159	2	1	15	8
3186	presurvey_arm_1	1	160	2	1	13	6
3187	presurvey_arm_1	1	160	2	1	11	6
3188	presurvey_arm_1	1	160	2	1	12	6

3189	presurvey_arm_1	1	160	2	1	11	6
3190	presurvey_arm_1	1	160	3	1	11	6
3191	presurvey_arm_1	1	160	2	1	12	6
3192	presurvey_arm_1	1	160	2	1	11	6
3193	presurvey_arm_1	1	160	2	1	11	6
3194	presurvey_arm_1	1	160	2	1	11	6
3195	presurvey_arm_1	1	160	2	1	12	6
3196	presurvey_arm_1	1	160	2	1	11	6
3197	presurvey_arm_1	1	160	2	1	11	6
3198	presurvey_arm_1	1	161	2	1	11	6
3199	presurvey_arm_1	1	161	2	1	12	6
3200	presurvey_arm_1	1	161	2	1	11	6
3201	presurvey_arm_1	1	161	2	1	11	6
3202	presurvey_arm_1	1	161	2	1	11	6
3203	presurvey_arm_1	1	161	2	1	12	6
3204	presurvey_arm_1	1	161	2	1	11	6
3205	presurvey_arm_1	1	161	2	1	12	6
3206	presurvey_arm_1	1	161	2	1	12	6
3207	presurvey_arm_1	1	161	3	1	11	6
3208	presurvey_arm_1	1	161	2	1	13	6
3209	presurvey_arm_1	1	161	2	1	12	6
3210	presurvey_arm_1	1	161	2	1	12	6
3211	presurvey_arm_1	1	161	2	1	12	6
3212	presurvey_arm_1	2	162	2	1	12	7
3213	presurvey_arm_1	2	162	2	1	13	8
3214	presurvey_arm_1	2	162	2	1	13	7
3215	presurvey_arm_1	2	162	2	1	13	7
3216	presurvey_arm_1	2	162	3	1	13	7
3217	presurvey_arm_1	2	162	2	1	12	7
3218	presurvey_arm_1	2	162	2	1	12	7
3219	presurvey_arm_1	2	162	3	1	14	7
3220	presurvey_arm_1	2	162	2	1	12	7
3221	presurvey_arm_1	2	162	2	1	13	7
3222	presurvey_arm_1	2	162	2	1	13	7
3223	presurvey_arm_1	2	162	2	1	13	7
3224	presurvey_arm_1	2	162	2	1	12	7
3225	presurvey_arm_1	2	162	2	1	12	7
3226	presurvey_arm_1	2	162	2	1	12	7
3227	presurvey_arm_1	2	162	2	1	12	7
3228	presurvey_arm_1	2	162	2	1	13	7
3229	presurvey_arm_1	1	161	3	1	13	6
3230	presurvey_arm_1	1	161	2	1	11	6
3231	presurvey_arm_1	1	161	2	1	12	6
3232	presurvey_arm_1	1	161	2	1	12	6
3233	presurvey_arm_1	1	161	2	1	12	6
3234	presurvey_arm_1	1	161	2	1	12	6
3235	presurvey_arm_1	1	161	2	1	11	6
3236	presurvey_arm_1	1	161	2	1	12	6

3237	presurvey_arm_1	1	161	2	1	12	6
3238	presurvey_arm_1	1	161	2	1	12	6
3239	presurvey_arm_1	2	163	2	1	13	7
3240	presurvey_arm_1	2	163	2	1	13	7
3241	presurvey_arm_1	2	163	2	1	13	7
3242	presurvey_arm_1	2	163	2	1	13	7
3243	presurvey_arm_1	2	163	2	1	13	7
3244	presurvey_arm_1	2	163	2	1	13	7
3245	presurvey_arm_1	2	163	2	1	13	7
3246	presurvey_arm_1	2	163	2	1	12	7
3247	presurvey_arm_1	2	163	2	1	13	7
3248	presurvey_arm_1	2	163	2	1	12	7
3249	presurvey_arm_1	2	163	2	1	13	7
3250	presurvey_arm_1	2	163	2	1	13	7
3251	presurvey_arm_1	2	163	2	1	13	7
3252	presurvey_arm_1	2	163	2	1	12	7
3253	presurvey_arm_1	2	163	2	1	13	7
3254	presurvey_arm_1	2	163	2	1	13	7
3255	presurvey_arm_1	2	163	2	1	13	7
3256	presurvey_arm_1	2	163	2	1	13	7
3257	presurvey_arm_1	2	163	2	1	12	7
3258	presurvey_arm_1	2	163	2	1	13	7
3259	presurvey_arm_1	2	163	2	1	13	7
3260	presurvey_arm_1	2	163	2	1	13	7
3261	presurvey_arm_1	2	163	2	1	13	7
3262	presurvey_arm_1	3	164	2	1	13	8
3263	presurvey_arm_1	3	164	2	1	14	8
3264	presurvey_arm_1	3	164	2	1	13	8
3265	presurvey_arm_1	3	164	2	1	13	8
3266	presurvey_arm_1	3	164	2	1	14	8
3267	presurvey_arm_1	3	164	2	1	13	8
3268	presurvey_arm_1	3	164	2	1	14	8
3269	presurvey_arm_1	3	164	2	1	14	8
3270	presurvey_arm_1	3	164	2	1	14	8
3271	presurvey_arm_1	3	164	2	1	14	8
3272	presurvey_arm_1	3	164	2	1	14	8
3273	presurvey_arm_1	3	165	2	1	13	8
3274	presurvey_arm_1	3	165	3	1	13	8
3275	presurvey_arm_1	3	165	2	1	14	8
3276	presurvey_arm_1	3	165	2	1	14	8
3277	presurvey_arm_1	3	165	2	1	14	8
3278	presurvey_arm_1	3	165	2	1	14	8
3279	presurvey_arm_1	3	165	3	1	13	8
3280	presurvey_arm_1	3	165	2	1	14	8
3281	presurvey_arm_1	3	165	2	1	14	8
3282	presurvey_arm_1	3	165	2	1	13	8
3283	presurvey_arm_1	3	165	3	1	14	8
3284	presurvey_arm_1	3	165	2	1	13	8

3285	presurvey_arm_1	3	165	2	1	14	8
3286	presurvey_arm_1	3	165	2	1	14	8
3287	presurvey_arm_1	3	165	2	1	13	8
3288	presurvey_arm_1	3	165	2	1	15	8
3289	presurvey_arm_1	3	165	3	1	14	8
3290	presurvey_arm_1	3	165	2	1	14	8
3291	presurvey_arm_1	1	174	3	1	12	6
3292	presurvey_arm_1	1	174	2	1	12	6
3293	presurvey_arm_1	1	174	3	1	12	6
3294	presurvey_arm_1	1	174	2	1	11	6
3295	presurvey_arm_1	1	174	2	1	12	6
3296	presurvey_arm_1	1	174	2	1	12	6
3297	presurvey_arm_1	1	174	2	1	12	6
3298	presurvey_arm_1	1	174	2	1	12	6
3299	presurvey_arm_1	1	174	2	1	12	6
3300	presurvey_arm_1	1	174	3	1	11	6
3301	presurvey_arm_1	1	174	3	1	11	6
3302	presurvey_arm_1	1	174	2	1	12	6
3303	presurvey_arm_1	1	174	3	1	12	6
3304	presurvey_arm_1	1	175	3	1	12	6
3305	presurvey_arm_1	1	175	2	1	12	6
3306	presurvey_arm_1	1	175	3	1	12	6
3307	presurvey_arm_1	1	174	3	1	12	6
3308	presurvey_arm_1	1	175	3	1	12	6
3309	presurvey_arm_1	1	175	2	1	12	6
3310	presurvey_arm_1	1	175	2	1	12	6
3311	presurvey_arm_1	1	175	3	1	12	6
3312	presurvey_arm_1	1	175	3	1	11	6
3313	presurvey_arm_1	1	175	3	1	12	6
3314	presurvey_arm_1	1	175	2	1	12	6
3315	presurvey_arm_1	1	175	2	1	11	6
3316	presurvey_arm_1	1	175	2	1	12	6
3317	presurvey_arm_1	1	175	3	1	12	6
3318	presurvey_arm_1	1	175	3	1	12	6
3319	presurvey_arm_1	1	175	3	1	12	6
3320	presurvey_arm_1	1	175	2	1	12	6
3321	presurvey_arm_1	1	175	3	1	11	6
3322	presurvey_arm_1	1	175	2	1	12	6
3323	presurvey_arm_1	1	175	3	1	12	6
3324	presurvey_arm_1	1	175	2	1	11	6
3325	presurvey_arm_1	1	175	3	1	12	6
3326	presurvey_arm_1	2	176	3	1	13	7
3327	presurvey_arm_1	2	176	2	1	13	7
3328	presurvey_arm_1	2	176	2	1	12	7
3329	presurvey_arm_1	2	176	3	1	14	7
3330	presurvey_arm_1	2	176	3	1	13	7
3331	presurvey_arm_1	2	176	3	1	13	7
3332	presurvey_arm_1	2	176	3	1	13	7

3333	presurvey_arm_1	2	176	3	1	13	7
3334	presurvey_arm_1	2	176	2	1	13	7
3335	presurvey_arm_1	2	176	3	1	14	7
3336	presurvey_arm_1	2	176	2	1	12	7
3337	presurvey_arm_1	2	176	3	1	13	7
3338	presurvey_arm_1	2	176	3	1	12	7
3339	presurvey_arm_1	2	176	3	1	12	7
3340	presurvey_arm_1	2	176	3	1	13	7
3341	presurvey_arm_1	2	177	3	1	13	7
3342	presurvey_arm_1	2	177	3	1	13	7
3343	presurvey_arm_1	2	177	3	1	13	7
3344	presurvey_arm_1	2	177	2	1	13	7
3345	presurvey_arm_1	2	177	3	1	14	7
3346	presurvey_arm_1	2	177	2	1	13	7
3347	presurvey_arm_1	2	177	2	1	12	7
3348	presurvey_arm_1	2	177	3	1	13	7
3349	presurvey_arm_1	2	177	2	1	13	7
3350	presurvey_arm_1	2	177	2	1	12	7
3351	presurvey_arm_1	2	177	3	1	13	7
3352	presurvey_arm_1	2	177	3	1	13	7
3353	presurvey_arm_1	2	177	3	1	12	7
3354	presurvey_arm_1	2	177	3	1	12	7
3355	presurvey_arm_1	2	177	2	1	12	7
3356	presurvey_arm_1	2	177	2	1	12	7
3357	presurvey_arm_1	2	177	3	1	10	6
3358	presurvey_arm_1	2	177	3	1	12	7
3359	presurvey_arm_1	2	177	2	1	13	7
3360	presurvey_arm_1	3	178	2	1	14	8
3361	presurvey_arm_1	3	178	3	1	14	8
3362	presurvey_arm_1	3	178	2	1	14	8
3363	presurvey_arm_1	3	178	3	1	14	8
3364	presurvey_arm_1	3	178	2	1	14	8
3365	presurvey_arm_1	3	178	3	1	13	8
3366	presurvey_arm_1	3	178	2	1	14	8
3367	presurvey_arm_1	3	178	2	1	14	8
3368	presurvey_arm_1	3	178	2	1	14	8
3369	presurvey_arm_1	3	178	3	1	14	8
3370	presurvey_arm_1	3	179	2	1	14	8
3371	presurvey_arm_1	3	179	2	1	14	8
3372	presurvey_arm_1	3	179	3	1	14	8
3373	presurvey_arm_1	3	179	3	1	14	8
3374	presurvey_arm_1	3	179	3	1	14	8
3375	presurvey_arm_1	3	179	2	1	13	8
3376	presurvey_arm_1	3	179	3	1	14	8
3377	presurvey_arm_1	3	179	3	1	16	8
3378	presurvey_arm_1	3	179	3	1	14	8
3379	presurvey_arm_1	3	179	3	1	14	8
3380	presurvey_arm_1	3	179	2	1	14	8

3407	presurvey_arm_1	1	155	2	1	13	6
3443	presurvey_arm_1	2	157	2	1	13	7
3456	presurvey_arm_1	3	158	2	1	14	8
3674	presurvey_arm_1	1	20	1	1	11	6
3675	presurvey_arm_1	1	20	1	1	11	6
3676	presurvey_arm_1	1	20	1	1	12	6
3677	presurvey_arm_1	1	20	1	1	12	6
3678	presurvey_arm_1	1	20	1	1	12	6
3679	presurvey_arm_1	1	20	1	1	11	6
3680	presurvey_arm_1	1	20	1	1	11	6
3681	presurvey_arm_1	1	20	1	1	11	6
3682	presurvey_arm_1	1	20	1	1	11	6
3683	presurvey_arm_1	1	20	1	1	12	6
3684	presurvey_arm_1	1	20	1	1	11	6
3685	presurvey_arm_1	1	20	1	1	11	6
3686	presurvey_arm_1	1	20	1	1	12	6
3687	presurvey_arm_1	1	20	1	1	11	6
3688	presurvey_arm_1	1	20	1	1	11	6
3689	presurvey_arm_1	1	21	1	1	11	6
3690	presurvey_arm_1	1	21	1	1	12	6
3691	presurvey_arm_1	1	21	1	1	10	6
3692	presurvey_arm_1	1	21	1	1	11	6
3693	presurvey_arm_1	1	21	1	1	13	6
3694	presurvey_arm_1	1	21	1	1	12	6
3695	presurvey_arm_1	1	21	1	1	12	6
3696	presurvey_arm_1	1	21	1	1	99	6
3697	presurvey_arm_1	1	21	1	1	11	6
3698	presurvey_arm_1	1	21	1	1	11	6
3699	presurvey_arm_1	1	21	1	1	12	6
3700	presurvey_arm_1	1	21	1	1	11	6
3701	presurvey_arm_1	1	21	1	1	11	6
3702	presurvey_arm_1	2	22	1	1	13	7
3703	presurvey_arm_1	2	22	1	1	13	7
3704	presurvey_arm_1	2	22	1	1	12	7
3705	presurvey_arm_1	2	22	1	1	12	7
3706	presurvey_arm_1	2	22	1	1	12	7
3707	presurvey_arm_1	2	22	1	1	12	7
3708	presurvey_arm_1	2	22	1	1	12	7
3709	presurvey_arm_1	2	22	1	1	12	7
3710	presurvey_arm_1	2	22	1	1	12	7
3711	presurvey_arm_1	2	22	1	1	13	7
3712	presurvey_arm_1	2	22	1	1	12	7
3713	presurvey_arm_1	2	22	1	1	12	7
3714	presurvey_arm_1	2	22	1	1	12	7
3715	presurvey_arm_1	2	23	1	1	12	7
3716	presurvey_arm_1	2	23	1	1	12	7
3717	presurvey_arm_1	2	23	1	1	12	7
3718	presurvey_arm_1	2	23	1	1	12	7

3719	presurvey_arm_1	2	23	1	1	12	7
3720	presurvey_arm_1	2	23	1	1	12	7
3721	presurvey_arm_1	2	23	1	1	12	7
3722	presurvey_arm_1	2	23	1	1	12	7
3723	presurvey_arm_1	2	23	1	1	12	7
3724	presurvey_arm_1	2	23	1	1	12	7
3725	presurvey_arm_1	2	23	1	1	12	7
3726	presurvey_arm_1	2	23	1	1	12	7
3727	presurvey_arm_1	2	23	1	1	12	7
3728	presurvey_arm_1	2	23	1	1	12	7
3729	presurvey_arm_1	2	23	1	1	12	7
3730	presurvey_arm_1	2	23	1	1	12	7
3731	presurvey_arm_1	2	23	1	1	12	7
3732	presurvey_arm_1	3	24	1	1	14	8
3733	presurvey_arm_1	3	24	1	1	13	8
3734	presurvey_arm_1	3	24	1	1	13	8
3735	presurvey_arm_1	3	24	1	1	13	8
3736	presurvey_arm_1	3	24	1	1	13	8
3737	presurvey_arm_1	3	24	1	1	13	8
3738	presurvey_arm_1	3	24	1	1	13	8
3739	presurvey_arm_1	3	24	1	1	14	8
3740	presurvey_arm_1	3	24	1	1	13	8
3741	presurvey_arm_1	3	24	1	1	13	8
3742	presurvey_arm_1	3	24	1	1	14	8
3743	presurvey_arm_1	3	24	1	1	13	8
3744	presurvey_arm_1	3	24	1	1	13	8
3745	presurvey_arm_1	3	24	1	1	13	8
3746	presurvey_arm_1	3	24	1	1	13	8
3747	presurvey_arm_1	3	25	1	1	13	8
3748	presurvey_arm_1	3	25	1	1	13	8
3749	presurvey_arm_1	3	25	1	1	13	8
3750	presurvey_arm_1	3	25	1	1	13	8
3751	presurvey_arm_1	3	25	1	1	13	8
3752	presurvey_arm_1	3	25	1	1	13	8
3753	presurvey_arm_1	3	25	1	1	13	8
3754	presurvey_arm_1	3	25	1	1	13	8
3755	presurvey_arm_1	3	25	1	1	13	8
3756	presurvey_arm_1	3	25	1	1	13	8
3757	presurvey_arm_1	3	25	1	1	13	8
3758	presurvey_arm_1	3	25	1	1	13	8
3759	presurvey_arm_1	3	25	1	1	15	8
3760	presurvey_arm_1	3	25	1	1	14	8
3761	presurvey_arm_1	3	25	1	1	13	99
3933	presurvey_arm_1	1	30	1	1	12	6
3934	presurvey_arm_1	1	30	1	1	11	6
3935	presurvey_arm_1	1	30	1	1	12	6
3936	presurvey_arm_1	1	30	1	1	11	6
3937	presurvey_arm_1	1	30	1	1	12	6

3938	presurvey_arm_1	1	30	1	1	11	6
3939	presurvey_arm_1	1	30	1	1	11	6
3940	presurvey_arm_1	1	30	1	1	11	6
3941	presurvey_arm_1	1	30	1	1	12	6
3942	presurvey_arm_1	1	30	1	1	13	6
3943	presurvey_arm_1	1	30	1	1	12	6
3944	presurvey_arm_1	1	30	1	1	12	6
3945	presurvey_arm_1	1	30	1	1	11	6
3946	presurvey_arm_1	1	30	1	1	11	6
3947	presurvey_arm_1	1	30	1	1	11	6
3948	presurvey_arm_1	1	30	1	1	11	6
3949	presurvey_arm_1	1	30	1	1	12	6
3950	presurvey_arm_1	1	31	1	1	11	6
3951	presurvey_arm_1	1	31	1	1	11	6
3952	presurvey_arm_1	1	31	1	1	11	6
3953	presurvey_arm_1	1	31	1	1	12	6
3954	presurvey_arm_1	1	31	1	1	11	6
3955	presurvey_arm_1	1	31	1	1	11	6
3956	presurvey_arm_1	1	31	1	1	12	6
3957	presurvey_arm_1	1	31	1	1	11	6
3958	presurvey_arm_1	1	31	1	1	11	6
3959	presurvey_arm_1	1	31	1	1	11	6
3960	presurvey_arm_1	1	31	1	1	13	6
3961	presurvey_arm_1	1	31	1	1	12	6
3962	presurvey_arm_1	2	32	1	1	12	7
3963	presurvey_arm_1	2	32	1	1	13	7
3964	presurvey_arm_1	2	32	1	1	12	7
3965	presurvey_arm_1	2	32	1	1	12	7
3966	presurvey_arm_1	2	32	1	1	12	7
3967	presurvey_arm_1	2	32	1	1	13	7
3968	presurvey_arm_1	2	32	1	1	12	7
3969	presurvey_arm_1	2	32	1	1	13	7
3970	presurvey_arm_1	2	32	1	1	13	7
3971	presurvey_arm_1	2	32	1	1	12	7
3972	presurvey_arm_1	2	32	1	1	12	7
3973	presurvey_arm_1	2	33	1	1	12	7
3974	presurvey_arm_1	2	33	1	1	12	7
3975	presurvey_arm_1	2	33	1	1	13	7
3976	presurvey_arm_1	2	33	1	1	12	7
3977	presurvey_arm_1	2	33	1	1	12	7
3978	presurvey_arm_1	2	33	1	1	12	7
3979	presurvey_arm_1	2	33	1	1	13	7
3980	presurvey_arm_1	2	33	1	1	12	7
3981	presurvey_arm_1	2	33	1	1	13	7
3982	presurvey_arm_1	2	33	1	1	13	7
3983	presurvey_arm_1	2	33	1	1	12	7
3984	presurvey_arm_1	2	33	1	1	13	7
3985	presurvey_arm_1	2	33	1	1	12	7

3986	presurvey_arm_1	2	33	1	1	12	7
3987	presurvey_arm_1	2	33	1	1	14	7
3988	presurvey_arm_1	2	33	1	1	13	7
3989	presurvey_arm_1	3	34	1	1	13	8
3990	presurvey_arm_1	3	34	1	1	13	8
3991	presurvey_arm_1	3	34	1	1	13	8
3992	presurvey_arm_1	3	34	1	1	13	8
3993	presurvey_arm_1	3	34	1	1	14	8
3994	presurvey_arm_1	3	34	1	1	12	8
3995	presurvey_arm_1	3	34	1	1	14	8
3996	presurvey_arm_1	3	34	1	1	13	8
3997	presurvey_arm_1	3	34	1	1	13	8
3998	presurvey_arm_1	3	34	1	1	12	8
3999	presurvey_arm_1	3	34	1	1	13	8
4000	presurvey_arm_1	3	34	1	1	13	8
4001	presurvey_arm_1	3	34	1	1	13	8
4002	presurvey_arm_1	3	34	1	1	13	8
4003	presurvey_arm_1	3	34	1	1	13	8
4004	presurvey_arm_1	3	34	1	1	14	8
4005	presurvey_arm_1	3	35	1	1	14	8
4006	presurvey_arm_1	3	35	1	1	13	8
4007	presurvey_arm_1	3	35	1	1	14	8
4008	presurvey_arm_1	3	35	1	1	14	8
4009	presurvey_arm_1	3	35	1	1	13	8
4010	presurvey_arm_1	3	35	1	1	13	8
4011	presurvey_arm_1	3	35	1	1	14	8
4012	presurvey_arm_1	3	35	1	1	13	8
4013	presurvey_arm_1	3	35	1	1	14	8
4014	presurvey_arm_1	3	35	1	1	13	8
4015	presurvey_arm_1	3	35	1	1	13	8
4016	presurvey_arm_1	3	35	1	1	14	8
4017	presurvey_arm_1	3	35	1	1	13	8
4018	presurvey_arm_1	3	35	1	1	14	8
4019	presurvey_arm_1	3	35	1	1	13	8
4020	presurvey_arm_1	1	36	1	1	12	6
4021	presurvey_arm_1	1	36	1	1	11	6
4022	presurvey_arm_1	1	36	1	1	11	6
4023	presurvey_arm_1	1	36	1	1	11	6
4024	presurvey_arm_1	1	36	1	1	11	6
4025	presurvey_arm_1	1	36	1	1	11	6
4026	presurvey_arm_1	1	36	1	1	11	6
4027	presurvey_arm_1	1	36	1	1	12	6
4028	presurvey_arm_1	1	36	1	1	11	6
4029	presurvey_arm_1	1	36	1	1	11	6
4030	presurvey_arm_1	1	36	1	1	11	6
4031	presurvey_arm_1	1	36	1	1	12	6
4032	presurvey_arm_1	1	36	1	1	11	6
4033	presurvey_arm_1	1	36	1	1	12	6

4034	presurvey_arm_1	1	37	1	1	11	6
4035	presurvey_arm_1	1	37	1	1	11	6
4036	presurvey_arm_1	1	37	1	1	11	6
4037	presurvey_arm_1	1	37	1	1	12	6
4038	presurvey_arm_1	1	37	1	1	12	6
4039	presurvey_arm_1	1	37	1	1	11	6
4040	presurvey_arm_1	1	37	1	1	11	6
4041	presurvey_arm_1	1	37	1	1	11	7
4042	presurvey_arm_1	1	37	1	1	11	6
4043	presurvey_arm_1	1	37	1	1	11	6
4044	presurvey_arm_1	1	37	1	1	11	6
4045	presurvey_arm_1	1	37	1	1	11	6
4046	presurvey_arm_1	1	37	1	1	11	6
4047	presurvey_arm_1	1	37	1	1	13	6
4048	presurvey_arm_1	2	38	1	1	13	7
4049	presurvey_arm_1	2	38	1	1	13	7
4050	presurvey_arm_1	2	38	1	1	12	7
4051	presurvey_arm_1	2	38	1	1	12	7
4052	presurvey_arm_1	2	38	1	1	12	7
4053	presurvey_arm_1	2	38	1	1	12	7
4054	presurvey_arm_1	2	38	1	1	12	7
4055	presurvey_arm_1	2	38	1	1	12	7
4056	presurvey_arm_1	2	38	1	1	12	7
4057	presurvey_arm_1	2	38	1	1	13	7
4058	presurvey_arm_1	2	38	1	1	13	7
4059	presurvey_arm_1	2	38	1	1	12	7
4060	presurvey_arm_1	2	38	1	1	12	7
4061	presurvey_arm_1	2	38	1	1	12	7
4062	presurvey_arm_1	2	38	1	1	13	7
4063	presurvey_arm_1	2	38	1	1	12	7
4064	presurvey_arm_1	2	38	1	1	12	7
4065	presurvey_arm_1	2	39	1	1	13	7
4066	presurvey_arm_1	2	39	1	1	12	7
4067	presurvey_arm_1	2	39	1	1	12	7
4068	presurvey_arm_1	2	39	1	1	12	7
4069	presurvey_arm_1	2	39	1	1	12	8
4070	presurvey_arm_1	2	39	1	1	13	7
4071	presurvey_arm_1	2	39	1	1	12	7
4072	presurvey_arm_1	2	39	1	1	12	7
4073	presurvey_arm_1	2	39	1	1	12	7
4074	presurvey_arm_1	2	39	1	1	12	7
4075	presurvey_arm_1	2	39	1	1	13	7
4076	presurvey_arm_1	3	40	1	1	14	8
4077	presurvey_arm_1	3	40	1	1	14	8
4078	presurvey_arm_1	3	40	1	1	13	8
4079	presurvey_arm_1	3	40	1	1	13	8
4080	presurvey_arm_1	3	40	1	1	13	8
4081	presurvey_arm_1	3	40	1	1	13	8

4082	presurvey_arm_1	3	40	1	1	13	8
4083	presurvey_arm_1	3	40	1	1	13	8
4084	presurvey_arm_1	3	40	1	1	13	8
4085	presurvey_arm_1	3	40	1	1	14	8
4086	presurvey_arm_1	3	40	1	1	13	8
4087	presurvey_arm_1	3	40	1	1	13	8
4088	presurvey_arm_1	3	40	1	1	13	8
4089	presurvey_arm_1	3	41	1	1	14	8
4090	presurvey_arm_1	3	41	1	1	13	8
4091	presurvey_arm_1	3	41	1	1	14	8
4092	presurvey_arm_1	3	41	1	1	13	8
4093	presurvey_arm_1	3	41	1	1	15	8
4094	presurvey_arm_1	3	41	1	1	14	8
4095	presurvey_arm_1	3	41	1	1	13	8
4096	presurvey_arm_1	3	41	1	1	13	8
4097	presurvey_arm_1	3	41	1	1	13	8
4098	presurvey_arm_1	3	41	1	1	13	8
4099	presurvey_arm_1	3	41	1	1	13	8
4100	presurvey_arm_1	3	41	1	1	14	8
4101	presurvey_arm_1	3	41	1	1	14	8
4102	presurvey_arm_1	3	41	1	1	13	8
4103	presurvey_arm_1	3	41	1	1	14	8
4104	presurvey_arm_1	3	41	1	1	13	8
4105	presurvey_arm_1	3	41	1	1	13	8
4186	presurvey_arm_1	1	36	1	1	12	6
4187	presurvey_arm_1	1	36	1	1	11	6
4188	presurvey_arm_1	2	39	1	1	15	7
4189	presurvey_arm_1	1	46	1	1	11	6
4190	presurvey_arm_1	1	46	1	1	11	6
4191	presurvey_arm_1	1	46	1	1	11	6
4192	presurvey_arm_1	1	46	1	1	11	6
4193	presurvey_arm_1	1	46	1	1	12	6
4194	presurvey_arm_1	1	46	1	1	12	6
4195	presurvey_arm_1	1	46	1	1	12	6
4196	presurvey_arm_1	1	46	1	1	10	6
4197	presurvey_arm_1	1	46	1	1	12	6
4198	presurvey_arm_1	1	46	1	1	12	6
4199	presurvey_arm_1	1	46	1	1	11	6
4200	presurvey_arm_1	1	46	1	1	12	6
4201	presurvey_arm_1	1	46	1	1	11	6
4202	presurvey_arm_1	1	46	1	1	11	6
4203	presurvey_arm_1	1	47	1	1	12	6
4204	presurvey_arm_1	1	47	1	1	10	6
4205	presurvey_arm_1	1	47	1	1	11	6
4206	presurvey_arm_1	1	47	1	1	11	6
4207	presurvey_arm_1	1	47	1	1	12	6
4208	presurvey_arm_1	1	47	1	1	12	6
4209	presurvey_arm_1	1	47	1	1	11	6

4210	presurvey_arm_1	1	47	1	1	12	6
4211	presurvey_arm_1	1	47	1	1	11	6
4212	presurvey_arm_1	1	47	1	1	12	6
4213	presurvey_arm_1	1	47	1	1	11	6
4214	presurvey_arm_1	1	47	1	1	11	6
4215	presurvey_arm_1	1	47	1	1	11	6
4216	presurvey_arm_1	1	47	1	1	11	6
4217	presurvey_arm_1	2	48	1	1	13	7
4218	presurvey_arm_1	2	48	1	1	12	7
4219	presurvey_arm_1	2	48	1	1	12	7
4220	presurvey_arm_1	2	48	1	1	13	7
4221	presurvey_arm_1	2	48	1	1	12	7
4222	presurvey_arm_1	2	48	1	1	13	7
4223	presurvey_arm_1	2	48	1	1	13	7
4224	presurvey_arm_1	2	48	1	1	13	7
4225	presurvey_arm_1	2	48	1	1	13	7
4226	presurvey_arm_1	2	48	1	1	12	7
4227	presurvey_arm_1	2	48	1	1	13	7
4228	presurvey_arm_1	2	49	1	1	12	7
4229	presurvey_arm_1	2	49	1	1	12	7
4230	presurvey_arm_1	2	49	1	1	13	7
4231	presurvey_arm_1	2	49	1	1	13	7
4232	presurvey_arm_1	2	49	1	1	12	7
4233	presurvey_arm_1	2	49	1	1	12	7
4234	presurvey_arm_1	2	49	1	1	12	7
4235	presurvey_arm_1	2	49	1	1	12	7
4236	presurvey_arm_1	2	49	1	1	12	7
4237	presurvey_arm_1	2	49	1	1	12	7
4238	presurvey_arm_1	2	49	1	1	12	7
4239	presurvey_arm_1	2	49	1	1	12	7
4240	presurvey_arm_1	2	49	1	1	12	7
4241	presurvey_arm_1	2	49	1	1	12	7
4242	presurvey_arm_1	2	49	1	1	13	7
4243	presurvey_arm_1	2	49	1	1	12	7
4244	presurvey_arm_1	2	49	1	1	12	7
4245	presurvey_arm_1	2	49	1	1	12	7
4246	presurvey_arm_1	3	50	1	1	13	8
4247	presurvey_arm_1	3	50	1	1	14	8
4248	presurvey_arm_1	3	50	1	1	14	8
4249	presurvey_arm_1	3	50	1	1	14	8
4250	presurvey_arm_1	3	50	1	1	13	8
4251	presurvey_arm_1	3	50	1	1	14	8
4252	presurvey_arm_1	3	50	1	1	13	8
4253	presurvey_arm_1	3	50	1	1	13	8
4254	presurvey_arm_1	3	50	1	1	14	8
4255	presurvey_arm_1	3	50	1	1	13	8
4256	presurvey_arm_1	3	50	1	1	13	8
4257	presurvey_arm_1	3	50	1	1	13	8

4258	presurvey_arm_1	3	50	1	1	13	8
4259	presurvey_arm_1	3	50	1	1	14	8
4260	presurvey_arm_1	3	51	1	1	13	8
4261	presurvey_arm_1	3	51	1	1	13	8
4262	presurvey_arm_1	3	51	1	1	13	8
4263	presurvey_arm_1	3	51	1	1	14	8
4264	presurvey_arm_1	3	51	1	1	13	8
4265	presurvey_arm_1	3	51	1	1	14	8
4266	presurvey_arm_1	3	51	1	1	13	8
4267	presurvey_arm_1	3	51	1	1	14	8
4268	presurvey_arm_1	3	51	1	1	13	8
4269	presurvey_arm_1	3	51	1	1	14	8
4270	presurvey_arm_1	3	51	1	1	13	8
4271	presurvey_arm_1	3	51	1	1	13	8
4272	presurvey_arm_1	3	51	1	1	14	8
4273	presurvey_arm_1	3	51	1	1	13	8
4274	presurvey_arm_1	3	51	1	1	14	8
4275	presurvey_arm_1	3	51	1	1	13	8
4276	presurvey_arm_1	3	51	1	1	13	8
4358	presurvey_arm_1	1	52	2	1	11	6
4359	presurvey_arm_1	1	52	2	1	11	6
4360	presurvey_arm_1	1	52	2	1	12	6
4361	presurvey_arm_1	1	52	2	1	11	6
4362	presurvey_arm_1	1	52	2	1	12	6
4363	presurvey_arm_1	1	52	2	1	11	6
4364	presurvey_arm_1	1	52	2	1	12	6
4365	presurvey_arm_1	1	52	2	1	11	6
4366	presurvey_arm_1	1	52	2	1	12	6
4367	presurvey_arm_1	1	52	2	1	12	6
4368	presurvey_arm_1	1	52	2	1	12	6
4369	presurvey_arm_1	1	52	2	1	11	6
4370	presurvey_arm_1	1	52	2	1	11	6
4371	presurvey_arm_1	1	52	2	1	12	6
4372	presurvey_arm_1	1	52	2	1	11	6
4373	presurvey_arm_1	1	52	2	1	12	6
4374	presurvey_arm_1	1	53	2	1	11	6
4375	presurvey_arm_1	1	53	2	1	11	6
4376	presurvey_arm_1	1	53	2	1	12	6
4377	presurvey_arm_1	1	53	2	1	12	6
4378	presurvey_arm_1	1	53	2	1	11	6
4379	presurvey_arm_1	1	53	2	1	12	6
4380	presurvey_arm_1	1	53	2	1	12	6
4381	presurvey_arm_1	1	53	2	1	12	6
4382	presurvey_arm_1	1	53	2	1	12	6
4383	presurvey_arm_1	1	53	2	1	12	6
4384	presurvey_arm_1	1	53	2	1	12	6
4385	presurvey_arm_1	1	53	2	1	11	6
4386	presurvey_arm_1	1	53	2	1	11	6

4387	presurvey_arm_1	2	54	2	1	13	7
4388	presurvey_arm_1	2	54	2	1	13	7
4389	presurvey_arm_1	2	54	2	1	12	7
4390	presurvey_arm_1	2	54	2	1	13	7
4391	presurvey_arm_1	2	54	2	1	12	7
4392	presurvey_arm_1	2	54	2	1	12	7
4393	presurvey_arm_1	2	54	2	1	13	7
4394	presurvey_arm_1	2	54	2	1	13	7
4395	presurvey_arm_1	2	54	2	1	13	7
4396	presurvey_arm_1	2	54	2	1	12	7
4397	presurvey_arm_1	2	54	2	1	13	7
4398	presurvey_arm_1	2	55	2	1	13	7
4399	presurvey_arm_1	2	55	2	1	13	7
4400	presurvey_arm_1	2	55	2	1	13	7
4401	presurvey_arm_1	2	55	2	1	13	7
4402	presurvey_arm_1	2	55	2	1	13	7
4403	presurvey_arm_1	2	55	2	1	12	7
4404	presurvey_arm_1	2	55	2	1	13	7
4405	presurvey_arm_1	2	55	2	1	12	7
4406	presurvey_arm_1	2	55	2	1	13	7
4407	presurvey_arm_1	2	55	2	1	13	7
4408	presurvey_arm_1	2	55	2	1	13	7
4409	presurvey_arm_1	2	55	2	1	13	7
4410	presurvey_arm_1	2	55	2	1	13	7
4411	presurvey_arm_1	2	55	2	1	12	7
4412	presurvey_arm_1	2	55	2	1	13	7
4413	presurvey_arm_1	2	55	2	1	12	7
4414	presurvey_arm_1	3	56	2	1	14	8
4415	presurvey_arm_1	3	56	2	1	13	8
4416	presurvey_arm_1	3	56	2	1	13	8
4417	presurvey_arm_1	3	56	2	1	15	8
4418	presurvey_arm_1	3	56	2	1	13	8
4419	presurvey_arm_1	3	56	2	1	14	8
4420	presurvey_arm_1	3	56	2	1	13	8
4421	presurvey_arm_1	3	56	2	1	14	8
4422	presurvey_arm_1	3	56	2	1	13	8
4423	presurvey_arm_1	3	56	2	1	13	8
4424	presurvey_arm_1	3	56	2	1	14	8
4425	presurvey_arm_1	3	56	2	1	14	8
4426	presurvey_arm_1	3	56	2	1	14	8
4427	presurvey_arm_1	3	57	2	1	14	8
4428	presurvey_arm_1	3	57	2	1	13	8
4429	presurvey_arm_1	3	57	2	1	13	8
4430	presurvey_arm_1	3	57	2	1	13	8
4431	presurvey_arm_1	3	57	2	1	13	8
4432	presurvey_arm_1	3	57	2	1	13	8
4433	presurvey_arm_1	3	57	2	1	13	8
4434	presurvey_arm_1	3	57	2	1	13	8

4435	presurvey_arm_1	3	57	2	1	13	8
4436	presurvey_arm_1	3	57	2	1	14	8
4437	presurvey_arm_1	3	57	2	1	13	8
4438	presurvey_arm_1	3	57	2	1	13	8
4439	presurvey_arm_1	3	57	2	1	13	8
4440	presurvey_arm_1	3	57	2	1	13	8
4441	presurvey_arm_1	3	57	2	1	13	8
4442	presurvey_arm_1	1	53	2	1	13	6
4443	presurvey_arm_1	2	55	2	1	13	7
4444	presurvey_arm_1	1	58	2	1	11	6
4445	presurvey_arm_1	1	58	2	1	12	6
4446	presurvey_arm_1	1	58	2	1	11	6
4447	presurvey_arm_1	1	58	2	1	11	6
4448	presurvey_arm_1	1	58	2	1	11	6
4449	presurvey_arm_1	1	58	2	1	11	6
4450	presurvey_arm_1	1	58	2	1	12	6
4451	presurvey_arm_1	1	58	2	1	12	6
4452	presurvey_arm_1	1	58	2	1	12	6
4453	presurvey_arm_1	1	58	2	1	11	6
4454	presurvey_arm_1	1	58	2	1	11	6
4455	presurvey_arm_1	1	58	2	1	11	6
4456	presurvey_arm_1	1	58	2	1	11	6
4457	presurvey_arm_1	1	58	2	1	12	6
4458	presurvey_arm_1	1	58	2	1	12	6
4459	presurvey_arm_1	1	59	2	1	12	6
4460	presurvey_arm_1	1	59	2	1	11	6
4461	presurvey_arm_1	1	59	2	1	11	6
4462	presurvey_arm_1	1	59	2	1	11	6
4463	presurvey_arm_1	1	59	2	1	12	6
4464	presurvey_arm_1	1	59	2	1	11	99
4465	presurvey_arm_1	1	59	2	1	12	6
4466	presurvey_arm_1	1	59	2	1	11	6
4467	presurvey_arm_1	1	59	2	1	11	6
4468	presurvey_arm_1	1	59	2	1	12	6
4469	presurvey_arm_1	1	59	2	1	11	6
4470	presurvey_arm_1	1	59	2	1	12	6
4471	presurvey_arm_1	1	59	2	1	12	6
4472	presurvey_arm_1	2	60	2	1	13	7
4473	presurvey_arm_1	2	60	2	1	12	7
4474	presurvey_arm_1	2	60	2	1	13	7
4475	presurvey_arm_1	2	60	2	1	13	7
4476	presurvey_arm_1	2	60	2	1	12	7
4477	presurvey_arm_1	2	60	2	1	12	7
4478	presurvey_arm_1	2	60	2	1	13	7
4479	presurvey_arm_1	2	60	2	1	13	7
4480	presurvey_arm_1	2	60	2	1	12	7
4481	presurvey_arm_1	2	61	2	1	13	7
4482	presurvey_arm_1	2	61	2	1	12	7

4483	presurvey_arm_1	2	61	2	1	12	7
4484	presurvey_arm_1	2	61	2	1	13	7
4485	presurvey_arm_1	2	61	2	1	13	7
4486	presurvey_arm_1	2	61	2	1	12	7
4487	presurvey_arm_1	2	61	2	1	12	7
4488	presurvey_arm_1	2	61	2	1	13	7
4489	presurvey_arm_1	2	61	2	1	13	7
4490	presurvey_arm_1	2	61	2	1	13	7
4491	presurvey_arm_1	2	61	2	1	13	7
4492	presurvey_arm_1	2	61	2	1	12	7
4493	presurvey_arm_1	2	61	2	1	13	7
4494	presurvey_arm_1	2	61	2	1	13	7
4495	presurvey_arm_1	3	62	2	1	14	8
4496	presurvey_arm_1	3	62	2	1	14	8
4497	presurvey_arm_1	3	62	2	1	14	8
4498	presurvey_arm_1	3	62	2	1	14	8
4499	presurvey_arm_1	3	62	2	1	14	8
4500	presurvey_arm_1	3	62	2	1	14	8
4501	presurvey_arm_1	3	62	2	1	13	8
4502	presurvey_arm_1	3	62	2	1	14	8
4503	presurvey_arm_1	3	62	2	1	14	8
4504	presurvey_arm_1	3	62	2	1	13	8
4505	presurvey_arm_1	3	63	2	1	14	8
4506	presurvey_arm_1	3	63	2	1	14	8
4507	presurvey_arm_1	3	63	2	1	13	8
4508	presurvey_arm_1	3	63	2	1	14	8
4509	presurvey_arm_1	3	63	2	1	15	8
4510	presurvey_arm_1	3	63	2	1	14	8
4511	presurvey_arm_1	3	63	2	1	14	8
4512	presurvey_arm_1	3	63	2	1	14	8
4513	presurvey_arm_1	3	63	2	1	14	8
4514	presurvey_arm_1	3	63	2	1	13	8
4515	presurvey_arm_1	3	63	2	1	14	8
4516	presurvey_arm_1	3	63	2	1	13	8
4517	presurvey_arm_1	3	63	2	1	13	8
4518	presurvey_arm_1	3	63	2	1	14	8
4519	presurvey_arm_1	3	63	2	1	13	8
4520	presurvey_arm_1	1	72	2	1	12	7
4521	presurvey_arm_1	1	72	2	1	12	6
4522	presurvey_arm_1	1	72	2	1	12	6
4523	presurvey_arm_1	1	72	2	1	12	6
4524	presurvey_arm_1	1	72	2	1	12	6
4525	presurvey_arm_1	1	72	2	1	12	6
4526	presurvey_arm_1	1	72	2	1	12	6
4527	presurvey_arm_1	1	72	2	1	12	6
4528	presurvey_arm_1	1	72	2	1	12	6
4529	presurvey_arm_1	1	72	2	1	12	6
4530	presurvey_arm_1	1	72	2	1	12	6

4531	presurvey_arm_1	1	72	2	1	12	6
4532	presurvey_arm_1	1	72	2	1	12	7
4533	presurvey_arm_1	1	72	2	1	12	6
4534	presurvey_arm_1	1	72	2	1	13	6
4535	presurvey_arm_1	1	73	2	1	11	6
4536	presurvey_arm_1	1	73	2	1	11	6
4537	presurvey_arm_1	1	73	2	1	12	6
4538	presurvey_arm_1	1	73	2	1	11	6
4539	presurvey_arm_1	1	73	2	1	12	6
4540	presurvey_arm_1	1	73	2	1	11	6
4541	presurvey_arm_1	1	73	2	1	12	6
4542	presurvey_arm_1	1	73	2	1	11	6
4543	presurvey_arm_1	1	73	2	1	13	6
4544	presurvey_arm_1	1	73	2	1	12	6
4545	presurvey_arm_1	1	73	2	1	11	6
4546	presurvey_arm_1	1	73	2	1	13	6
4547	presurvey_arm_1	2	74	2	1	12	7
4548	presurvey_arm_1	2	74	2	1	13	7
4549	presurvey_arm_1	2	74	2	1	12	7
4550	presurvey_arm_1	2	74	2	1	12	7
4551	presurvey_arm_1	2	74	2	1	13	7
4552	presurvey_arm_1	2	74	2	1	12	7
4553	presurvey_arm_1	2	74	2	1	13	7
4554	presurvey_arm_1	2	74	2	1	13	7
4555	presurvey_arm_1	2	74	2	1	13	7
4556	presurvey_arm_1	2	75	2	1	14	7
4557	presurvey_arm_1	2	75	2	1	13	7
4558	presurvey_arm_1	2	75	2	1	12	7
4559	presurvey_arm_1	2	75	2	1	13	7
4560	presurvey_arm_1	2	75	2	1	13	7
4561	presurvey_arm_1	2	75	2	1	13	7
4562	presurvey_arm_1	2	75	2	1	13	7
4563	presurvey_arm_1	2	75	2	1	13	7
4564	presurvey_arm_1	2	75	2	1	13	7
4565	presurvey_arm_1	2	75	2	1	13	7
4566	presurvey_arm_1	2	75	2	1	13	7
4567	presurvey_arm_1	2	75	2	1	13	7
4568	presurvey_arm_1	2	75	2	1	13	7
4569	presurvey_arm_1	2	75	2	1	13	7
4570	presurvey_arm_1	2	75	2	1	13	7
4571	presurvey_arm_1	3	76	2	1	13	8
4572	presurvey_arm_1	3	76	2	1	13	8
4573	presurvey_arm_1	3	76	2	1	14	8
4574	presurvey_arm_1	3	76	2	1	13	8
4575	presurvey_arm_1	3	76	2	1	14	8
4576	presurvey_arm_1	3	76	2	1	14	8
4577	presurvey_arm_1	3	76	2	1	13	8
4578	presurvey_arm_1	3	76	2	1	14	8

4579	presurvey_arm_1	3	76	2	1	14	8
4580	presurvey_arm_1	3	77	2	1	13	8
4581	presurvey_arm_1	3	77	2	1	14	8
4582	presurvey_arm_1	3	77	2	1	14	8
4583	presurvey_arm_1	3	77	2	1	13	8
4584	presurvey_arm_1	3	77	2	1	14	8
4585	presurvey_arm_1	3	77	2	1	14	8
4586	presurvey_arm_1	3	77	2	1	14	8
4587	presurvey_arm_1	3	77	2	1	13	8
4588	presurvey_arm_1	3	77	2	1	14	8
4589	presurvey_arm_1	3	77	2	1	15	8
4590	presurvey_arm_1	3	77	2	1	14	8
4591	presurvey_arm_1	3	77	2	1	14	8
4592	presurvey_arm_1	3	77	2	1	13	8
4593	presurvey_arm_1	3	77	2	1	14	8
4594	presurvey_arm_1	3	77	2	1	13	8
4595	presurvey_arm_1	3	77	2	1	14	8
4596	presurvey_arm_1	3	77	2	1	13	8
4597	presurvey_arm_1	3	77	2	1	14	8
4598	presurvey_arm_1	1	78	2	1	12	6
4599	presurvey_arm_1	1	78	2	1	12	6
4600	presurvey_arm_1	1	78	2	1	12	6
4601	presurvey_arm_1	1	78	2	1	12	6
4602	presurvey_arm_1	1	78	2	1	12	6
4603	presurvey_arm_1	1	78	2	1	12	6
4604	presurvey_arm_1	1	78	2	1	12	6
4605	presurvey_arm_1	1	78	2	1	11	6
4606	presurvey_arm_1	1	78	2	1	12	6
4607	presurvey_arm_1	1	78	2	1	12	6
4608	presurvey_arm_1	1	78	2	1	12	6
4609	presurvey_arm_1	1	78	2	1	11	6
4610	presurvey_arm_1	1	78	2	1	12	6
4611	presurvey_arm_1	1	78	2	1	12	6
4612	presurvey_arm_1	1	78	2	1	11	6
4613	presurvey_arm_1	1	79	2	1	12	6
4614	presurvey_arm_1	1	79	2	1	12	6
4615	presurvey_arm_1	1	79	2	1	11	6
4616	presurvey_arm_1	1	79	2	1	12	6
4617	presurvey_arm_1	1	79	2	1	12	6
4618	presurvey_arm_1	1	79	2	1	12	6
4619	presurvey_arm_1	1	79	2	1	12	6
4620	presurvey_arm_1	1	79	2	1	12	6
4621	presurvey_arm_1	1	79	2	1	12	6
4622	presurvey_arm_1	1	79	2	1	11	6
4623	presurvey_arm_1	1	79	2	1	12	6
4624	presurvey_arm_1	1	79	2	1	11	6
4625	presurvey_arm_1	1	79	2	1	12	6
4626	presurvey_arm_1	1	79	2	1	12	6

4627	presurvey_arm_1	2	80	2	1	12	7
4628	presurvey_arm_1	2	80	2	1	13	7
4629	presurvey_arm_1	2	80	2	1	13	7
4630	presurvey_arm_1	2	80	2	1	13	7
4631	presurvey_arm_1	2	80	2	1	14	7
4632	presurvey_arm_1	2	80	2	1	13	7
4633	presurvey_arm_1	2	80	2	1	13	7
4634	presurvey_arm_1	2	80	2	1	13	7
4635	presurvey_arm_1	2	80	2	1	12	7
4636	presurvey_arm_1	2	81	2	1	12	7
4637	presurvey_arm_1	2	81	2	1	13	7
4638	presurvey_arm_1	2	81	2	1	13	7
4639	presurvey_arm_1	2	81	2	1	13	7
4640	presurvey_arm_1	2	81	2	1	13	7
4641	presurvey_arm_1	2	81	2	1	99	7
4642	presurvey_arm_1	2	81	2	1	13	7
4643	presurvey_arm_1	2	81	2	1	13	7
4644	presurvey_arm_1	2	81	2	1	12	7
4645	presurvey_arm_1	2	81	2	1	12	7
4646	presurvey_arm_1	2	81	2	1	13	7
4647	presurvey_arm_1	2	81	2	1	13	7
4648	presurvey_arm_1	2	81	2	1	13	7
4649	presurvey_arm_1	2	81	2	1	13	7
4650	presurvey_arm_1	3	82	2	1	14	8
4651	presurvey_arm_1	3	82	2	1	15	8
4652	presurvey_arm_1	3	82	2	1	14	8
4653	presurvey_arm_1	3	82	2	1	15	8
4654	presurvey_arm_1	3	82	2	1	15	8
4655	presurvey_arm_1	3	82	2	1	13	8
4656	presurvey_arm_1	3	82	2	1	14	8
4657	presurvey_arm_1	3	82	2	1	13	8
4658	presurvey_arm_1	3	82	2	1	14	8
4659	presurvey_arm_1	3	82	2	1	13	8
4660	presurvey_arm_1	3	82	2	1	14	8
4661	presurvey_arm_1	3	82	2	1	14	8
4662	presurvey_arm_1	3	82	2	1	14	8
4663	presurvey_arm_1	3	82	2	1	14	8
4664	presurvey_arm_1	3	83	2	1	14	8
4665	presurvey_arm_1	3	83	2	1	14	8
4666	presurvey_arm_1	3	83	2	1	14	8
4667	presurvey_arm_1	3	83	2	1	14	8
4668	presurvey_arm_1	3	83	2	1	15	8
4669	presurvey_arm_1	3	83	2	1	15	8
4670	presurvey_arm_1	3	83	2	1	14	8
4671	presurvey_arm_1	3	83	2	1	14	8
4672	presurvey_arm_1	3	83	2	1	13	8
4673	presurvey_arm_1	3	83	2	1	14	8
4674	presurvey_arm_1	3	83	2	1	14	8

4675	presurvey_arm_1	3	83	2	1	13	8
4676	presurvey_arm_1	3	83	2	1	15	8
4677	presurvey_arm_1	3	83	2	1	14	8
4985	presurvey_arm_1	#NULL!	#NULL!	5	1	12	7
4986	presurvey_arm_1	#NULL!	#NULL!	5	1	13	8
4987	presurvey_arm_1	#NULL!	#NULL!	5	1	13	8
4988	presurvey_arm_1	#NULL!	#NULL!	5	1	13	8
4989	presurvey_arm_1	#NULL!	#NULL!	5	1	13	8
4990	presurvey_arm_1	#NULL!	#NULL!	5	1	14	8
4991	presurvey_arm_1	#NULL!	#NULL!	5	1	14	8
4992	presurvey_arm_1	#NULL!	#NULL!	5	1	13	8
4993	presurvey_arm_1	#NULL!	#NULL!	5	1	13	8
4994	presurvey_arm_1	#NULL!	#NULL!	5	1	13	8
4995	presurvey_arm_1	#NULL!	#NULL!	5	1	14	8
4996	presurvey_arm_1	#NULL!	#NULL!	5	1	13	8
4997	presurvey_arm_1	#NULL!	#NULL!	5	1	14	8
4998	presurvey_arm_1	#NULL!	#NULL!	5	1	13	8
4999	presurvey_arm_1	#NULL!	#NULL!	5	1	13	8
5000	presurvey_arm_1	#NULL!	#NULL!	5	1	13	8
5001	presurvey_arm_1	#NULL!	#NULL!	5	1	14	8
5002	presurvey_arm_1	#NULL!	#NULL!	5	1	13	8
5003	presurvey_arm_1	#NULL!	#NULL!	5	1	13	8
5004	presurvey_arm_1	#NULL!	#NULL!	5	1	13	8
5005	presurvey_arm_1	#NULL!	#NULL!	5	1	13	8
5006	presurvey_arm_1	#NULL!	#NULL!	5	1	14	8
5007	presurvey_arm_1	#NULL!	#NULL!	5	1	13	8
5008	presurvey_arm_1	#NULL!	#NULL!	5	1	13	8
5009	presurvey_arm_1	#NULL!	#NULL!	5	1	13	8
5010	presurvey_arm_1	#NULL!	#NULL!	5	1	13	8
5011	presurvey_arm_1	#NULL!	#NULL!	5	#NULL!	13	8
5012	presurvey_arm_1	#NULL!	#NULL!	5	1	13	8
5013	presurvey_arm_1	#NULL!	#NULL!	5	1	13	8
5014	presurvey_arm_1	#NULL!	#NULL!	5	1	13	8
5015	presurvey_arm_1	#NULL!	#NULL!	5	1	13	8
5016	presurvey_arm_1	#NULL!	#NULL!	5	1	13	8
5017	presurvey_arm_1	#NULL!	#NULL!	5	1	14	8
5018	presurvey_arm_1	#NULL!	#NULL!	5	1	13	8
5019	presurvey_arm_1	#NULL!	#NULL!	5	1	13	8
5020	presurvey_arm_1	#NULL!	#NULL!	5	1	13	8
5021	presurvey_arm_1	#NULL!	#NULL!	5	1	14	8
5022	presurvey_arm_1	#NULL!	#NULL!	5	1	13	8
5023	presurvey_arm_1	#NULL!	#NULL!	5	1	12	7
5024	presurvey_arm_1	#NULL!	#NULL!	5	1	13	7
5025	presurvey_arm_1	#NULL!	#NULL!	5	1	12	7
5026	presurvey_arm_1	#NULL!	#NULL!	5	1	13	7
5027	presurvey_arm_1	#NULL!	#NULL!	5	1	12	7
5028	presurvey_arm_1	#NULL!	#NULL!	5	1	13	8
5029	presurvey_arm_1	#NULL!	#NULL!	5	1	13	7

5030	presurvey_arm_1	#NULL!	#NULL!	5	1	12	7
5031	presurvey_arm_1	#NULL!	#NULL!	5	1	12	7
5032	presurvey_arm_1	#NULL!	#NULL!	5	1	13	7
5033	presurvey_arm_1	#NULL!	#NULL!	5	1	11	7
5034	presurvey_arm_1	#NULL!	#NULL!	5	1	13	7
5035	presurvey_arm_1	#NULL!	#NULL!	5	1	12	7
5036	presurvey_arm_1	#NULL!	#NULL!	5	1	12	7
5037	presurvey_arm_1	#NULL!	#NULL!	5	1	12	7
5038	presurvey_arm_1	#NULL!	#NULL!	999	#NULL!	12	7
5039	presurvey_arm_1	#NULL!	#NULL!	5	1	12	7
5040	presurvey_arm_1	#NULL!	#NULL!	999	#NULL!	12	7
5041	presurvey_arm_1	#NULL!	#NULL!	5	1	12	7
5042	presurvey_arm_1	#NULL!	#NULL!	5	1	12	7
5043	presurvey_arm_1	#NULL!	#NULL!	999	#NULL!	12	7
5044	presurvey_arm_1	#NULL!	#NULL!	5	1	12	7
5045	presurvey_arm_1	#NULL!	#NULL!	5	1	12	8
5046	presurvey_arm_1	#NULL!	#NULL!	5	1	12	7
5047	presurvey_arm_1	#NULL!	#NULL!	5	1	12	7
5048	presurvey_arm_1	#NULL!	#NULL!	5	1	12	7
5049	presurvey_arm_1	#NULL!	#NULL!	5	#NULL!	12	7
5050	presurvey_arm_1	#NULL!	#NULL!	999	1	12	7
5051	presurvey_arm_1	#NULL!	#NULL!	5	1	13	7
5052	presurvey_arm_1	#NULL!	#NULL!	999	1	12	7
5053	presurvey_arm_1	#NULL!	#NULL!	5	1	12	7
5054	presurvey_arm_1	#NULL!	#NULL!	999	#NULL!	13	8
5055	presurvey_arm_1	#NULL!	#NULL!	5	1	13	8
5056	presurvey_arm_1	#NULL!	#NULL!	5	1	13	8
5057	presurvey_arm_1	#NULL!	#NULL!	5	1	11	6
5058	presurvey_arm_1	#NULL!	#NULL!	5	1	11	6
5059	presurvey_arm_1	#NULL!	#NULL!	999	#NULL!	11	6
5060	presurvey_arm_1	#NULL!	#NULL!	5	1	11	6
5061	presurvey_arm_1	#NULL!	#NULL!	5	1	11	6
5062	presurvey_arm_1	#NULL!	#NULL!	5	1	12	6
5063	presurvey_arm_1	#NULL!	#NULL!	5	1	11	6
5064	presurvey_arm_1	#NULL!	#NULL!	5	1	11	6
5065	presurvey_arm_1	#NULL!	#NULL!	999	1	11	6
5066	presurvey_arm_1	#NULL!	#NULL!	5	1	11	6
5067	presurvey_arm_1	#NULL!	#NULL!	5	1	11	6
5068	presurvey_arm_1	#NULL!	#NULL!	5	1	11	6
5069	presurvey_arm_1	#NULL!	#NULL!	5	1	12	6
5070	presurvey_arm_1	#NULL!	#NULL!	5	1	11	6
5071	presurvey_arm_1	#NULL!	#NULL!	5	1	11	6
5072	presurvey_arm_1	#NULL!	#NULL!	5	1	11	6
5073	presurvey_arm_1	#NULL!	#NULL!	5	1	11	6
5074	presurvey_arm_1	#NULL!	#NULL!	5	1	11	6
5075	presurvey_arm_1	#NULL!	#NULL!	5	1	13	8
5076	presurvey_arm_1	#NULL!	#NULL!	5	1	11	6
5077	presurvey_arm_1	#NULL!	#NULL!	5	1	12	6

5078	presurvey_arm_1	#NULL!	#NULL!	5	1	12	6
5079	presurvey_arm_1	#NULL!	#NULL!	5	1	11	6
5080	presurvey_arm_1	#NULL!	#NULL!	5	1	11	6
5081	presurvey_arm_1	#NULL!	#NULL!	5	#NULL!	11	6
5082	presurvey_arm_1	#NULL!	#NULL!	5	1	11	6
5083	presurvey_arm_1	#NULL!	#NULL!	5	1	11	6
5084	presurvey_arm_1	#NULL!	#NULL!	5	1	12	6
5085	presurvey_arm_1	#NULL!	#NULL!	5	1	11	6
5086	presurvey_arm_1	#NULL!	#NULL!	5	1	12	6
5087	presurvey_arm_1	#NULL!	#NULL!	5	#NULL!	11	6
5088	presurvey_arm_1	#NULL!	#NULL!	5	1	12	6
5089	presurvey_arm_1	#NULL!	#NULL!	5	1	11	6
5090	presurvey_arm_1	#NULL!	#NULL!	5	1	11	6
5091	presurvey_arm_1	#NULL!	#NULL!	5	1	11	6
5092	presurvey_arm_1	#NULL!	#NULL!	5	1	13	8
5093	presurvey_arm_1	#NULL!	#NULL!	5	1	11	6
5094	presurvey_arm_1	#NULL!	#NULL!	999	1	13	8
5095	presurvey_arm_1	#NULL!	#NULL!	5	1	12	7
5096	presurvey_arm_1	#NULL!	#NULL!	5	1	13	8
5097	presurvey_arm_1	#NULL!	#NULL!	5	1	11	6
5098	presurvey_arm_1	#NULL!	#NULL!	5	1	11	6

gender_pre	sexorient_pre___1	sexorient_pre___2	sexorient_pre___3	sexorient_pre___4
1	1	0	0	0
0	1	0	0	0
1	0	0	0	0
0	0	0	0	1
0	1	0	0	0
0	1	0	0	0
0	1	0	0	0
0	1	0	0	0
0	1	0	0	0
1	1	0	0	0
0	1	0	0	0
1	1	0	0	0
0	1	0	0	0
1	1	0	0	0
1	1	0	0	0
0	1	0	0	0
0	1	0	0	0
0	1	0	0	0
0	1	0	0	0
0	1	0	0	0
0	1	0	0	0
1	1	0	0	0
0	1	0	0	0
0	1	0	0	0
1	1	0	0	0
1	1	0	0	0
1	1	0	0	0
0	1	0	0	0
1	1	0	0	0
1	1	0	0	0
1	1	0	0	0
0	1	0	0	0
0	1	0	0	0
1	1	0	0	0
1	1	0	0	0
1	1	0	0	0
0	1	0	0	0
1	1	0	0	0
1	1	0	0	0
1	1	0	0	0
1	1	0	0	0
0	1	0	0	0
0	1	0	0	0
1	0	0	0	0
1	1	0	0	0
1	1	0	0	0
0	1	0	0	0

0	1	0	0	0
1	1	0	0	0
1	1	0	0	0
1	0	0	0	1
0	1	0	0	0
1	1	0	0	0
1	1	0	0	0
1	1	0	0	0
1	1	0	0	0
0	1	0	0	0
0	1	0	0	0
0	0	0	0	0
0	0	0	0	0
1	1	0	0	0
0	1	0	0	0
1	1	0	0	0
1	1	0	0	0
1	1	0	0	0
1	0	0	0	1
0	1	0	0	0
0	1	0	0	0
1	1	0	0	0
1	1	0	0	0
0	1	0	0	0
1	1	0	0	0
1	1	0	0	0
0	1	0	0	0
0	1	0	0	0
1	1	0	0	0
1	1	0	0	0
0	1	0	0	0
1	1	0	0	0
1	1	0	0	0
1	1	0	0	0
1	0	0	0	1
1	1	0	0	0
1	1	0	0	0
1	1	0	0	0
0	1	0	0	0
0	1	0	0	0
1	1	0	0	0
0	1	0	0	0
0	1	0	0	0
0	1	0	0	0
1	1	0	0	0
0	1	0	0	0
1	0	0	0	0
1	1	0	0	0

0	1	0	0	0
0	1	0	0	0
0	1	0	0	0
1	1	0	0	0
1	0	0	0	0
0	0	0	0	0
1	0	0	0	1
1	1	0	0	0
1	1	0	0	0
1	1	0	0	0
0	1	0	0	0
0	1	0	0	0
0	1	0	0	0
0	1	0	0	0
1	1	0	0	0
1	0	0	0	1
1	0	0	0	1
1	1	0	0	0
0	1	0	0	0
0	1	0	0	0
0	1	0	0	0
1	1	0	0	0
0	1	0	0	0
3	0	1	0	0
1	1	0	0	0
0	1	0	0	0
0	1	0	0	0
0	1	0	0	0
0	1	0	0	0
1	1	0	0	0
1	1	0	0	0
0	1	0	0	0
0	1	0	0	0
1	1	0	0	0
1	1	0	0	0
0	1	0	0	0
0	0	0	0	0
1	0	1	0	0
1	1	0	0	0
1	1	0	0	0
1	1	0	0	0
0	1	0	0	0
1	1	0	0	0
0	0	0	0	0
0	1	0	0	0
0	1	0	0	0
1	1	0	0	0
1	1	0	0	0

0	1	0	0	0
0	1	0	0	0
1	0	0	0	0
1	1	0	0	0
1	0	0	0	1
1	0	0	0	1
0	0	0	0	0
0	1	0	0	0
0	1	0	0	0
0	1	0	0	0
1	1	0	0	0
0	1	0	0	0
0	0	0	0	0
0	1	0	0	0
0	0	0	0	0
0	1	0	0	0
0	1	0	0	0
0	1	0	0	0
0	1	0	0	0
1	1	0	0	0
1	1	0	0	0
1	1	0	0	0
1	1	0	0	0
1	1	0	0	0
0	0	0	0	0
0	1	0	0	0
1	1	0	0	0
0	1	0	0	0
1	1	0	0	0
0	1	0	0	0
0	1	0	0	0
0	1	0	0	0
0	1	0	0	0
1	1	0	0	0
0	1	0	0	0
0	0	0	0	0
1	1	0	0	0
0	1	0	0	0
1	1	0	0	0
1	1	0	0	0
1	1	0	0	0
1	1	0	0	0
0	1	0	0	0
1	0	0	0	1
1	0	0	0	1
1	1	0	0	0
1	1	0	0	0
1	1	0	0	0

1	1	0	0	0
1	0	0	0	0
0	1	0	0	0
0	1	0	0	0
1	1	0	0	0
0	1	0	0	0
0	1	0	0	0
1	1	0	0	0
0	1	0	0	0
1	1	0	0	0
0	1	0	0	0
0	1	0	0	0
1	1	0	0	0
1	0	0	0	1
1	1	0	0	0
1	1	0	0	0
1	0	0	0	0
1	1	0	0	0
1	1	0	0	0
1	1	0	0	0
0	1	0	0	0
1	1	0	0	0
0	1	0	0	0
0	1	0	0	0
0	1	0	0	0
1	0	0	0	1
1	1	0	0	0
1	1	0	0	0
0	1	0	0	0
0	1	0	0	0
1	1	0	0	0
1	1	0	0	0
0	1	0	0	0
99	0	0	0	0
1	1	0	0	0
1	0	0	0	0
0	1	0	0	0
0	1	0	0	0
0	1	0	0	0
0	1	0	0	0
0	1	0	0	0
0	1	0	0	0
1	1	0	0	0
0	1	0	0	0
1	0	0	0	1
1	1	0	0	0
0	1	0	0	0
1	1	0	0	0

0	0	0	0	1
0	1	0	0	0
1	0	0	0	0
0	1	0	0	0
0	1	0	0	0
1	1	0	0	0
0	0	0	0	0
0	0	0	0	0
0	1	0	0	0
1	0	0	0	0
0	1	0	0	0
1	0	0	0	0
0	1	0	0	0
0	1	0	0	0
0	1	0	0	0
1	0	0	0	0
0	1	0	0	0
0	1	0	0	0
0	1	0	0	0
0	0	0	1	1
0	1	0	0	0
1	1	0	0	0
0	1	0	0	0
1	0	0	0	0
0	1	0	0	0
0	1	0	0	0
1	1	0	0	0
0	0	0	0	0
1	0	1	0	0
1	1	0	0	0
1	1	0	0	0
0	1	0	0	0
1	1	0	0	0
1	1	0	0	0
0	1	0	0	0
0	1	0	0	0
1	0	0	0	0
0	1	0	0	0
1	1	0	0	0
1	1	0	0	0
1	0	0	0	1
0	1	0	0	0
0	1	0	0	0
0	1	0	0	0
1	1	0	0	0
1	0	0	0	1
0	1	0	0	0
1	0	0	0	0

1	1	0	0	0
1	1	0	0	0
1	0	0	0	0
1	0	0	0	1
0	1	0	0	0
0	1	0	0	0
1	1	0	0	0
0	1	0	0	0
1	1	0	0	0
0	1	0	0	0
1	1	0	0	0
0	1	0	0	0
1	1	0	0	0
0	1	0	0	0
0	1	0	0	0
0	1	0	0	0
0	1	0	0	0
1	1	0	0	0
1	1	0	0	0
1	1	0	0	0
1	0	0	0	0
1	0	0	0	1
0	1	0	0	0
1	1	0	0	0
0	1	0	0	0
0	1	0	0	0
0	1	0	0	0
1	0	0	0	1
0	1	0	0	0
0	1	0	0	0
1	0	0	0	1
0	1	0	0	0
1	0	0	0	1
0	1	0	0	0
0	0	0	0	0
0	1	0	0	0
0	1	0	0	0
1	1	0	0	0
0	1	0	0	0
1	1	0	0	0
1	1	0	0	0
1	1	0	0	0
0	1	0	0	0
1	1	0	0	0
1	1	0	0	0
1	1	0	0	0
0	0	0	0	1
0	1	0	0	0

1	1	0	0	0
1	1	0	0	0
1	1	0	0	0
0	1	0	0	0
0	1	0	0	0
0	1	0	0	0
1	1	0	0	0
0	1	0	0	0
0	1	0	0	0
1	1	0	0	0
1	1	0	0	0
1	1	0	0	0
0	0	0	0	1
1	1	0	0	0
1	0	0	0	0
1	1	0	0	0
1	1	0	0	0
0	1	0	0	0
1	1	0	0	0
1	1	0	0	0
1	1	0	0	0
3	0	0	1	0
1	0	0	0	1
0	1	0	0	0
0	1	0	0	0
0	1	0	0	0
0	1	0	0	0
0	1	0	0	0
1	1	0	0	0
0	1	0	0	0
1	0	0	0	1
1	1	0	0	0
0	1	0	0	0
1	0	0	0	1
0	1	0	0	0
1	0	0	0	0
0	1	0	0	0
0	1	0	0	0
0	1	0	0	0
0	0	0	0	1
0	1	0	0	0
1	1	0	0	0
0	1	0	0	0
1	0	0	0	1
0	0	0	1	0
0	1	0	0	0
0	1	0	0	0
1	1	0	0	0

1	1	0	0	0
1	1	0	0	0
0	1	0	0	0
1	0	0	0	1
0	0	0	0	0
0	1	0	0	0
0	1	0	0	0
1	1	0	0	0
1	1	0	0	0
0	0	0	1	0
0	1	0	0	0
1	0	0	0	1
0	1	0	0	0
0	1	0	0	0
1	1	0	0	0
1	1	0	0	0
1	1	0	0	0
1	0	0	0	0
0	1	0	0	0
0	0	0	0	0
0	1	0	0	0
0	1	0	0	0
0	1	0	0	0
0	0	0	0	0
0	0	0	0	0
0	1	0	0	0
1	1	0	0	0
1	0	0	0	0
1	0	0	0	0
0	1	0	0	0
1	0	0	0	0
1	1	0	0	0
0	1	0	0	0
0	1	0	0	0
1	0	0	0	0
0	1	0	0	0
0	1	0	0	0
1	0	0	0	1
0	1	0	0	0
0	1	0	0	0
1	1	0	0	0
1	1	0	0	0
0	1	0	0	0
0	1	0	0	0
1	0	1	0	0
1	1	0	0	0
1	1	0	0	0
0	1	0	0	0

0	1	0	0	0
1	1	0	0	0
0	1	0	0	0
0	0	0	0	0
1	1	0	0	0
0	1	0	0	0
0	1	0	0	0
1	1	0	0	0
0	1	0	0	0
1	1	0	0	0
1	1	0	0	0
0	1	0	0	0
0	1	0	0	0
1	1	0	0	0
1	1	0	0	0
1	1	0	0	0
0	1	0	0	0
1	1	0	0	0
1	0	0	0	1
1	1	0	0	0
0	1	0	0	0
1	1	0	0	0
1	1	0	0	0
0	1	0	0	0
1	1	0	0	0
0	1	0	0	0
0	0	0	0	0
0	1	0	0	0
0	1	0	0	0
1	1	0	0	0
1	1	0	0	0
0	1	0	0	0
1	1	0	0	0
1	1	0	0	0
1	0	0	0	1
0	1	0	0	0
1	1	0	0	0
1	1	0	0	0
1	1	0	0	0
0	1	0	0	0
1	0	1	0	0
0	1	0	0	0
0	1	0	0	0
0	1	0	0	0
0	1	0	0	0
1	0	0	0	0
0	1	0	0	0
0	1	0	0	0

1	0	0	0	0
1	0	0	0	0
0	1	0	0	0
0	0	0	0	0
1	1	0	0	0
1	0	0	0	1
1	1	0	0	0
0	1	0	0	0
1	1	0	0	0
0	1	0	0	0
0	0	0	0	0
0	1	0	0	0
1	1	0	0	0
0	1	0	0	0
0	1	0	0	0
0	1	0	0	0
0	1	0	0	0
1	1	0	0	0
1	1	0	0	0
1	1	0	0	0
1	1	0	0	0
0	1	0	0	0
0	1	0	0	0
1	1	0	0	0
1	0	0	0	0
1	1	0	0	0
1	1	0	0	0
1	1	0	0	0
1	1	0	0	0
0	1	0	0	0
1	0	0	0	1
1	1	0	0	0
1	1	0	0	0
1	0	0	0	0
1	0	0	0	0
0	1	0	0	0
0	1	0	0	0
0	1	1	0	0
0	1	0	0	0
0	1	0	0	0
0	1	0	0	0
0	1	0	0	0
0	1	0	0	0
0	1	0	0	0
1	0	0	0	1
0	1	0	0	0
1	1	0	0	0
0	1	0	0	0

1	1	0	0	0
1	1	0	0	0
1	1	0	0	0
1	1	0	0	0
0	1	0	0	0
0	0	0	0	0
0	1	0	0	0
0	1	0	0	0
0	1	0	0	0
0	0	0	0	0
1	1	0	0	0
0	1	0	0	0
1	1	0	0	0
1	1	0	0	0
1	1	0	0	0
0	1	0	0	0
0	1	0	0	0
0	1	0	0	0
0	1	0	0	0
1	1	0	0	0
0	1	0	0	0
1	0	0	0	1
0	1	0	0	0
3	0	0	0	0
1	0	0	0	1
1	1	0	0	0
1	0	0	0	1
0	0	0	0	1
0	1	0	0	0
0	1	0	0	0
1	0	1	0	0
0	1	0	0	0
1	1	0	0	0
1	0	0	0	1
1	1	0	0	0
0	1	0	0	0
1	1	0	0	0
1	0	0	0	0
0	1	0	0	0
1	1	0	0	0
0	1	0	0	0
1	1	0	0	0
0	1	0	0	0
0	1	0	0	0
0	1	0	0	0
0	0	0	0	0
0	1	0	0	0
0	1	0	0	0

0	1	0	0	0
1	1	0	0	0
1	1	0	0	0
0	1	0	0	0
0	1	0	0	0
1	1	0	0	0
0	1	0	0	0
0	1	0	0	0
1	1	0	0	0
0	1	0	0	0
0	1	0	0	0
0	0	0	0	0
1	1	0	0	0
0	1	0	0	0
0	1	0	0	0
0	0	0	0	0
1	1	0	0	0
1	1	0	0	0
0	1	0	0	0
0	1	0	0	0
1	0	1	0	0
1	1	0	0	0
0	1	0	0	0
1	1	0	0	0
0	1	0	0	0
1	1	0	0	0
0	0	0	0	0
1	1	0	0	0
0	0	0	0	0
1	1	0	0	0
99	0	0	0	0
1	1	0	0	0
1	1	0	0	0
1	1	0	0	0
0	1	0	0	0
0	1	0	0	0
0	0	0	0	0
0	0	0	0	0
0	1	0	0	0
1	1	0	0	0
0	0	0	0	0
1	1	0	0	0
0	1	0	0	0
0	1	0	0	0
0	1	0	0	0
1	1	0	0	0
0	0	0	0	0
1	1	0	0	0

1	0	0	0	0
0	1	0	0	0
1	1	0	0	0
0	0	0	0	0
1	1	0	0	0
0	0	0	0	0
0	1	0	0	0
1	1	0	0	0
1	1	0	0	0
1	1	0	0	0
0	1	0	0	0
1	1	0	0	0
1	0	1	0	0
1	1	0	0	0
0	1	0	0	0
0	1	0	0	0
0	1	0	0	0
0	1	0	0	0
1	1	0	0	0
0	1	0	0	0
1	0	0	0	1
0	1	0	0	0
0	1	0	0	0
0	1	0	0	0
1	1	0	0	0
1	1	0	0	0
0	1	0	0	0
1	1	0	0	0
0	1	0	0	0
0	1	0	0	0
1	0	0	0	0
1	1	0	0	0
0	1	0	0	0
0	1	0	0	0
1	1	0	0	0
0	1	0	0	0
0	1	0	0	0
1	1	0	0	0
1	1	0	0	0
0	0	0	0	0
1	0	0	0	1
1	1	0	0	0
0	1	0	0	0
0	1	0	0	0
0	1	0	0	0
1	1	0	0	0
0	1	0	0	0
1	1	0	0	0

0	1	0	0	0
0	1	0	0	0
0	1	0	0	0
1	1	0	0	0
0	1	0	0	0
1	1	0	0	0
0	0	0	0	0
0	1	0	0	0
0	1	0	0	0
1	1	0	0	0
0	1	0	0	0
1	1	0	0	0
1	1	0	0	0
0	1	0	0	0
1	1	0	0	0
1	1	0	0	0
1	1	0	0	0
1	0	0	0	0
1	0	0	0	1
1	1	0	0	0
1	1	0	0	0
1	1	0	0	0
99	1	0	0	0
0	1	0	0	0
1	1	0	0	0
0	1	0	0	0
1	1	0	0	0
0	0	0	0	0
1	0	0	0	1
0	0	0	0	0
0	1	0	0	0
0	1	0	0	0
0	0	0	0	0
0	0	0	0	1
1	0	0	0	0
0	1	0	0	0
1	1	0	0	0
0	1	0	0	0
0	1	0	0	0
1	1	0	0	0
1	0	0	0	0
0	1	0	0	0
0	1	0	0	0
0	1	0	0	0
1	1	0	0	0
1	0	0	0	1
0	0	0	0	0
1	1	0	0	0

0	0	0	0	0
1	0	0	0	0
1	1	0	0	0
0	0	0	0	0
0	0	0	0	0
0	1	0	0	0
1	1	0	0	0
0	1	0	0	0
0	1	0	0	0
0	0	0	0	0
1	1	0	0	0
0	1	0	0	1
1	0	0	0	0
1	0	0	0	1
1	1	0	0	0
0	1	0	0	0
0	1	0	0	0
1	0	0	0	0
1	0	0	0	0
99	0	0	0	0
1	1	0	0	0
0	1	0	0	0
0	1	0	0	0
1	1	0	0	0
1	1	0	0	0
1	0	0	0	1
1	1	0	0	0
0	1	0	0	0
1	1	0	0	0
1	0	0	1	0
1	1	0	0	0
1	1	0	0	0
1	1	0	0	0
1	0	0	0	0
0	1	0	0	0
1	1	0	0	0
0	1	0	0	0
1	0	0	0	0
0	0	0	0	0
0	0	0	0	0
1	1	0	0	0
1	1	0	0	0
0	1	0	0	0
1	0	0	0	0
1	1	0	0	0
1	1	0	0	0
0	1	0	0	0
1	1	0	0	0

0	1	0	0	0
0	1	0	0	0
1	0	0	0	0
1	1	0	0	0
1	0	0	0	1
1	1	0	0	0
1	1	0	0	0
0	1	0	0	0
0	0	0	0	0
1	1	0	0	0
0	1	0	0	0
0	1	0	0	0
0	1	0	0	0
1	1	0	0	0
0	1	0	0	0
1	1	0	0	0
1	0	0	0	1
1	1	0	0	0
0	1	0	0	0
1	1	0	0	0
1	1	0	0	0
1	1	0	0	0
1	1	0	0	0
1	1	0	0	0
1	0	0	0	1
0	1	0	0	0
0	0	0	0	0
0	0	0	0	0
0	1	0	0	0
0	1	0	0	0
1	0	0	0	1
0	1	0	0	0
0	1	0	0	0
1	1	0	0	0
0	1	0	0	0
1	0	0	0	0
0	1	0	0	0
0	1	0	0	0
1	0	0	0	1
0	1	0	0	0
1	0	0	0	0
0	0	0	0	0
1	1	0	0	0
0	1	0	0	0
0	1	0	0	0
1	0	0	0	0
0	1	0	0	0
0	0	0	0	0

1	1	0	0	0
0	1	0	0	0
0	1	0	0	0
0	1	0	0	0
0	1	0	0	0
0	1	0	0	0
1	1	0	0	0
0	1	0	0	0
1	1	0	0	0
0	1	0	0	0
1	1	0	0	0
1	1	0	0	0
1	0	0	0	0
1	0	0	0	0
1	1	0	0	0
0	1	0	0	0
1	1	0	0	0
0	1	0	0	0
0	1	0	0	0
1	1	0	0	0
1	1	0	0	0
3	0	0	0	0
0	1	0	0	0
1	1	0	0	0
1	0	0	0	1
0	1	0	0	0
0	0	0	0	0
1	0	0	0	1
0	1	0	0	0
1	1	0	0	0
1	0	0	0	1
0	0	0	0	0
1	0	0	0	0
1	1	0	0	0
0	1	0	0	0
1	1	0	0	0
0	1	0	0	0
0	1	0	0	0
3	1	0	0	0
1	0	0	0	1
0	1	0	0	0
0	1	0	0	0
1	1	0	0	0
0	1	0	0	0
1	1	0	0	0
0	1	0	0	0
0	1	0	0	0
0	1	0	0	0

1	1	0	0	0
0	1	0	0	0
1	0	0	0	0
1	1	0	0	0
0	1	0	0	0
1	1	0	0	0
1	1	0	0	0
0	1	0	0	0
0	1	0	0	0
0	1	0	0	0
0	1	0	0	0
1	1	0	0	0
0	1	0	0	0
0	0	0	0	1
1	1	0	0	0
1	1	0	0	0
1	1	0	0	0
1	1	0	0	0
0	0	0	0	0
1	1	0	0	0
1	1	0	0	0
0	0	0	0	0
0	0	0	0	0
0	0	0	0	1
0	0	0	0	0
1	1	0	0	0
0	1	0	0	0
1	1	0	0	0
0	0	0	0	0
0	1	0	0	0
1	0	0	0	0
0	1	0	0	0
1	1	0	0	0
0	1	0	0	0
1	1	0	0	0
1	1	0	0	0
0	0	0	0	0
1	1	0	0	0
0	1	0	0	0
0	1	0	0	0
2	0	0	0	0
1	1	0	0	0
0	1	0	0	0
0	1	0	0	0
1	1	0	0	0
3	1	0	0	0
1	1	0	0	0
1	1	0	0	0

1	1	0	0	0
1	0	0	0	0
0	1	0	0	0
0	1	0	0	0
0	0	0	1	0
1	1	0	0	0
0	0	0	0	1
0	0	0	0	0
0	0	0	0	0
0	0	0	0	0
0	1	0	0	0
0	0	0	0	0
0	1	0	0	0
0	1	0	0	0
0	0	0	0	0
0	1	0	0	0
1	0	0	0	0
1	0	0	0	1
0	1	0	0	0
1	1	0	0	0
0	1	0	0	0
0	1	0	0	0
1	1	0	0	0
0	1	0	0	0
0	1	0	0	0
1	0	0	0	0
0	1	0	0	0
1	1	0	0	0
1	1	0	0	0
0	1	0	0	0
0	1	0	0	0
0	0	0	0	0
0	1	0	0	0
0	1	0	0	0
1	0	0	0	0
0	1	0	0	0
0	1	0	0	0
1	1	0	0	0
0	1	0	0	0
3	1	0	0	0
1	0	0	0	1
0	0	0	0	0
0	1	0	0	0
1	1	0	0	0
0	1	0	0	0
0	1	0	0	0
1	1	0	0	0
0	1	0	0	0

1	1	0	0	0
0	1	0	0	0
0	1	0	0	0
1	1	0	0	0
0	1	0	0	0
1	1	0	0	0
1	1	0	0	0
1	0	0	0	1
1	0	0	0	0
1	1	0	0	0
1	1	0	0	0
1	0	0	0	1
0	1	0	0	0
1	0	0	0	1
1	0	0	0	0
0	0	0	0	0
1	1	0	0	0
0	0	0	0	0
1	1	0	0	0
1	1	0	0	0
0	1	0	0	0
0	1	0	0	0
1	1	0	0	0
1	0	0	0	0
0	1	0	0	0
1	1	0	0	0
0	1	0	0	0
1	1	0	0	0
1	1	0	0	0
0	1	0	0	0
0	1	0	0	0
0	1	0	0	0
1	1	0	0	0
0	0	0	0	0
1	1	0	0	0
0	0	0	0	0
0	1	0	0	0
1	1	0	0	0
0	1	0	0	0
1	1	0	0	0
0	1	0	0	0
0	1	0	0	0
1	1	0	0	0
0	0	0	0	0
1	1	0	0	0
1	1	0	0	0
1	0	0	0	1
1	1	0	0	0

0	1	0	0	0
0	1	0	0	0
1	1	0	0	0
1	0	0	0	1
0	1	0	0	0
0	1	0	0	0
0	1	0	0	0
0	1	0	0	0
1	1	0	0	0
1	1	0	0	0
1	1	0	0	0
1	1	0	0	0
1	1	0	0	0
0	1	0	0	0
0	1	0	0	0
1	1	0	0	0
1	1	0	0	0
1	1	0	0	0
0	1	0	0	0
0	1	0	0	0
0	1	0	0	0
99	1	0	0	0
0	1	0	0	0
1	1	0	0	0
2	0	0	0	0
1	0	0	0	0
0	1	0	0	0
0	1	0	0	0
1	0	0	0	1
0	1	0	0	0
0	0	0	0	0
1	1	0	0	0
1	0	0	0	1
0	1	0	0	0
1	1	0	0	0
0	1	0	0	0
1	1	0	0	0
1	1	0	0	0
1	1	0	0	0
1	1	0	0	0
0	1	0	0	0
99	0	0	1	0
1	0	0	0	1
0	1	0	0	0
1	1	0	0	0
1	0	0	0	1
1	0	0	0	1
0	1	0	0	0

1	1	0	0	1
0	0	0	0	0
1	1	0	0	0
0	1	0	0	0
1	1	0	0	0
1	1	0	0	0
0	1	0	0	0
1	1	0	0	0
0	1	0	0	0
1	1	0	0	0
1	1	0	0	0
1	1	0	0	0
0	1	0	0	0
0	1	0	0	0
1	0	0	0	0
0	1	0	0	0
0	1	0	0	0
99	0	0	0	1
1	1	0	0	0
0	1	0	0	0
1	1	0	0	0
0	1	0	0	0
0	1	0	0	0
1	1	0	0	0
0	1	0	0	0
1	1	0	0	0
0	0	0	0	0
1	1	0	0	0
0	1	0	0	0
1	1	0	0	0
1	1	0	0	0
1	0	0	0	0
0	1	0	0	0
1	1	0	0	0
0	1	0	0	0
1	0	0	0	1
1	1	0	0	0
1	1	0	0	0
0	1	0	0	0
1	1	0	0	0
1	1	0	0	0
1	1	0	0	0
0	1	0	0	0
0	1	0	0	0
1	1	0	0	0
0	1	0	0	0
1	1	0	0	0
0	1	0	0	0

1	1	0	0	0
0	1	0	0	0
1	0	0	0	1
0	1	0	0	0
3	0	0	0	1
0	1	0	0	0
1	1	0	0	0
0	1	0	0	0
3	0	0	0	0
0	1	0	0	0
2	0	0	0	0
1	1	0	0	0
1	1	0	0	0
1	0	0	0	0
1	1	0	0	0
0	1	0	0	0
1	1	0	0	0
1	1	0	0	0
1	0	0	0	0
0	1	0	0	0
0	1	0	0	0
1	0	1	1	0
0	1	0	0	0
0	1	0	0	0
0	1	0	0	0
0	1	0	0	0
0	1	0	0	0
0	1	0	0	0
1	1	0	0	0
0	1	0	0	0
0	1	0	0	0
1	1	0	0	0
1	0	0	0	1
1	1	0	0	0
0	1	0	0	0
1	1	0	0	0
3	0	0	0	0
0	1	0	0	0
0	1	0	0	0
0	1	0	0	0
0	1	0	0	0
0	1	0	0	0
1	1	0	0	0
0	1	0	0	0
1	1	0	0	0
1	1	0	0	0
0	1	0	0	0
1	1	0	0	0

0	1	0	0	0
0	1	0	0	0
0	1	0	0	0
1	1	0	0	0
3	0	1	0	0
3	0	0	0	1
0	1	0	0	0
0	0	1	0	0
0	1	0	0	0
0	1	0	0	0
1	1	0	0	0
1	1	0	0	0
0	1	0	0	0
1	1	0	0	0
1	0	0	0	0
1	1	0	0	0
1	1	0	0	0
0	1	0	0	0
0	1	0	0	0
1	1	0	0	0
0	1	0	0	0
1	1	0	0	0
0	1	0	0	0
1	1	0	0	0
1	0	0	0	0
1	1	0	0	0
0	1	0	0	0
1	1	0	0	0
0	1	0	0	0
0	0	0	0	0
1	1	0	0	0
0	0	0	0	0
1	0	0	0	0
1	1	0	0	0
0	1	0	0	0
0	1	0	0	0
1	0	0	0	0
0	0	0	0	0
1	1	0	0	0
1	0	0	0	0
3	1	0	0	0
1	1	0	0	0
0	0	0	0	0
1	1	0	0	0
1	1	0	0	0
1	1	0	0	0
0	1	0	0	0
1	1	0	0	0

1	0	0	0	0
0	1	0	0	0
0	1	0	0	0
1	1	0	0	0
0	1	0	0	0
0	0	0	0	0
0	0	0	0	0
1	1	0	0	0
3	1	1	0	0
0	1	0	0	0
1	1	0	0	0
1	0	0	0	0
0	1	0	0	0
0	1	0	0	0
1	1	0	0	0
1	0	0	0	0
0	1	0	0	0
0	1	0	0	0
1	1	0	0	0
1	0	0	0	1
0	1	0	0	0
1	1	0	0	0
0	1	0	0	0
1	1	0	0	0
0	1	0	0	0
0	1	0	0	0
0	1	0	0	0
1	1	0	0	0
1	0	1	0	0
0	1	0	0	0
0	1	0	0	0
0	1	0	0	0
1	1	0	0	0
0	1	0	0	0
1	0	0	0	1
0	1	0	0	0
0	1	0	0	0
1	0	0	0	1
1	1	0	0	0
1	1	0	0	0
1	1	0	0	0
0	1	0	0	0
1	1	0	0	0
0	1	0	0	0
0	0	0	0	0
0	1	0	0	0
0	1	0	0	0
1	0	0	0	0

1	1	0	0	0
0	1	0	0	0
0	1	0	0	0
0	1	0	0	0
1	1	0	0	0
1	1	0	0	0
3	1	1	1	1
0	1	0	0	0
0	1	0	0	0
0	1	0	0	0
1	1	0	0	0
1	1	0	0	0
1	1	0	0	0
1	1	0	0	0
1	1	0	0	0
1	1	0	0	0
0	0	0	0	0
1	1	0	0	0
1	1	0	0	0
1	1	0	0	0
0	1	0	0	0
1	1	0	0	0
0	1	0	0	0
1	1	0	0	0
1	0	0	0	0
1	1	0	0	1
0	1	0	0	0
0	1	0	0	0
0	0	0	0	0
0	1	0	0	0
0	1	0	0	0
0	1	0	0	0
3	0	0	0	0
0	1	0	0	0
0	1	0	0	0
0	1	0	0	0
0	1	0	0	0
1	1	0	0	0
1	1	0	0	0
0	0	0	0	0
1	1	0	0	0
0	1	0	0	0
1	0	0	0	0
1	0	0	0	0
0	1	0	0	0
1	0	0	0	0
1	1	0	0	0
0	1	0	0	0

0	1	0	0	0
1	1	0	0	0
0	0	0	0	0
1	1	0	0	0
1	1	0	0	0
0	0	0	0	0
1	1	0	0	0
0	1	0	0	0
0	0	0	0	0
1	1	0	0	0
0	1	0	0	0
1	1	0	0	0
0	0	0	0	0
1	0	0	0	0
0	1	0	0	0
1	1	0	0	0
1	1	0	0	0
1	1	0	0	0
1	1	0	0	0
0	1	0	0	0
0	1	0	0	0
1	1	0	0	0
1	0	0	0	0
1	1	0	0	0
0	0	0	0	0
1	1	0	0	0
0	1	0	0	0
0	1	0	0	0
1	1	0	0	0
0	1	0	0	0
0	0	0	0	0
0	1	0	0	0
1	1	0	0	0
1	1	0	0	0
1	1	0	0	0
1	1	0	0	0
0	1	0	0	0
0	1	0	0	0
0	1	0	0	0
0	1	0	0	0
0	0	0	0	0
0	1	0	0	0
0	1	0	0	0
1	1	0	0	0
1	1	0	0	0
0	1	0	0	0
1	0	0	0	0
0	1	0	0	0

0	1	0	0	0
1	1	0	0	0
0	1	0	0	0
1	0	0	0	0
1	1	0	0	0
1	1	0	0	0
1	0	0	0	0
0	1	0	0	0
0	1	0	0	0
0	0	0	0	0
1	1	0	0	0
1	1	0	0	0
0	0	0	0	0
0	1	0	0	0
0	1	0	0	0
1	1	0	0	0
1	1	0	0	0
1	1	0	0	0
0	1	0	0	0
0	1	0	0	0
0	1	0	0	0
1	1	0	0	0
1	0	0	0	0
1	1	0	0	0
0	0	0	0	0
1	1	0	0	0
0	1	0	0	0
0	1	0	0	0
1	1	0	0	0
0	1	0	0	0
1	1	0	0	0
0	1	0	0	0
0	1	0	0	0
0	1	0	0	0
1	1	0	0	0
3	1	0	0	0
1	1	0	0	0
0	1	0	0	0
1	1	0	0	0
1	0	0	0	1
1	1	0	0	0
0	1	0	0	0
1	1	0	0	0
0	1	0	0	0
1	1	0	0	0
0	1	0	0	0
0	1	0	0	0
1	1	0	0	0

0	0	0	0	0
0	1	0	0	0
0	1	0	0	0
0	1	0	0	0
0	0	0	0	0
0	1	0	0	0
0	1	0	0	0
0	1	0	0	0
0	1	0	0	0
0	1	0	0	0
0	0	0	0	0
1	0	0	0	0
1	1	0	0	0
1	1	0	0	0
0	1	0	0	0
1	1	0	0	0
1	1	0	0	0
0	0	0	0	0
1	1	0	0	0
0	0	0	0	0
1	1	0	0	0
0	1	0	0	0
1	1	0	0	0
1	0	0	0	0
1	1	0	0	0
1	1	0	0	0
1	1	0	0	0
3	0	0	0	0
0	1	0	0	0
0	0	0	0	0
1	1	0	0	0
1	0	0	0	0
1	0	0	0	0
0	1	0	0	0
1	1	0	0	0
1	1	0	0	0
0	1	0	0	0
1	1	0	0	0
0	0	0	0	0
1	1	0	0	0
0	1	0	0	0
1	1	0	0	0
0	1	0	0	0
1	1	0	0	0
1	1	0	0	0
1	1	0	0	0
0	1	0	0	0
0	1	0	0	0

1	1	0	0	0
1	1	0	0	0
1	1	0	0	0
0	1	0	0	0
0	1	0	0	0
99	1	0	0	0
1	1	0	0	0
1	0	0	0	1
0	1	0	0	0
0	1	0	0	0
3	0	0	0	1
0	1	0	0	0
1	1	0	0	0
0	1	0	0	0
0	0	0	0	0
1	1	0	0	0
0	1	0	0	0
1	0	0	0	0
0	1	0	0	0
0	1	0	0	0
1	1	0	0	0
1	1	0	0	0
0	1	0	0	0
0	1	0	0	0
1	1	0	0	0
0	1	0	0	0
1	1	0	0	0
1	1	0	0	0
1	1	0	0	0
0	1	0	0	0
1	1	0	0	0
1	1	0	0	0
0	1	0	0	0
1	1	0	0	0
0	1	0	0	0
1	1	0	0	0
0	1	0	0	0
1	0	0	0	0
0	1	0	0	0
1	1	0	0	0
1	1	0	0	0
0	1	0	0	0
0	1	0	0	0
1	1	0	0	0
1	1	0	0	0
1	0	0	0	0
0	1	0	0	0
1	1	0	0	0

1	1	0	0	0
0	1	0	0	0
0	1	0	0	0
0	1	0	0	0
1	1	0	0	0
0	1	0	0	0
1	1	0	0	0
0	1	0	0	0
0	1	0	0	0
1	1	0	0	0
0	1	0	0	0
0	1	0	0	0
0	1	0	0	0
0	1	0	0	0
1	1	0	0	0
0	0	0	0	0
0	0	0	0	0
0	1	0	0	0
1	1	0	0	0
0	1	0	0	0
0	0	0	0	0
0	1	0	0	0
1	1	0	0	0
0	1	0	0	0
0	1	0	0	0
0	0	0	0	0
1	1	0	0	0
0	0	0	0	0
1	0	0	0	0
0	1	0	0	0
1	1	0	0	0
1	0	0	0	1
1	1	0	0	0
0	0	0	0	0
1	0	0	0	0
1	1	0	0	0
1	1	0	0	0
0	1	0	0	0
1	1	0	0	0
1	0	0	0	0
1	0	0	0	0
99	0	0	0	0
1	1	0	0	0
1	1	0	0	0
1	0	0	0	0
0	1	0	0	0
1	0	0	0	0
1	1	0	0	0

1	1	0	0	0
0	1	0	0	0
1	1	0	0	0
0	0	0	0	0
1	0	0	0	0
1	0	0	0	0
0	1	0	0	0
1	0	0	0	1
0	1	0	0	0
1	1	0	0	0
0	1	0	0	0
1	1	0	0	0
2	0	0	1	0
1	0	0	0	0
1	0	0	0	0
0	1	0	0	0
0	0	0	0	0
1	1	0	0	0
1	1	0	0	0
0	1	0	0	0
0	1	0	0	0
1	1	0	0	0
0	1	0	0	0
1	1	0	0	0
0	1	0	0	0
1	1	0	0	0
1	0	0	0	0
0	1	0	0	0
1	0	1	0	0
0	1	0	0	0
0	1	0	0	0
0	1	0	0	0
1	1	0	0	0
0	1	0	0	0
3	1	0	0	0
0	1	0	0	0
0	1	0	0	0
1	1	0	0	0
1	1	0	0	0
0	0	0	0	0
0	1	0	0	0
0	1	0	0	0
0	1	0	0	0
0	0	0	0	0
0	1	0	0	0
1	0	0	0	1
0	1	0	0	0
1	1	0	0	0

0	1	0	0	0
0	1	0	0	0
0	1	0	0	0
0	1	0	0	0
1	1	0	0	0
3	0	0	0	0
0	1	0	0	0
1	1	0	0	0
0	1	0	0	0
0	1	0	0	0
0	1	0	0	0
0	1	0	0	0
1	1	0	0	0
1	1	0	0	0
0	1	0	0	0
1	1	0	0	0
0	1	0	0	0
0	1	0	0	0
0	1	0	0	0
0	1	0	0	0
1	1	0	0	0
1	1	0	0	0
1	1	0	0	0
0	1	0	0	0
1	1	0	0	0
0	1	0	0	0
0	1	0	0	0
0	1	0	0	0
0	0	0	0	0
0	1	0	0	0
0	1	0	0	0
1	0	0	0	0
0	0	0	0	0
1	1	0	0	0
0	1	0	0	0
0	1	0	0	0
1	0	0	0	0
0	1	0	0	0
1	1	0	0	0
0	1	0	0	0
0	1	0	0	0
1	0	0	0	0
0	1	0	0	0
0	0	0	0	0
0	0	0	0	0
1	0	0	0	0
1	1	0	0	0
0	0	0	0	0

0	0	0	0	0
1	1	0	0	0
0	1	0	0	0
0	1	0	0	0
1	1	0	0	0
1	1	0	0	0
0	0	0	0	0
0	0	0	0	0
1	1	0	0	0
1	1	0	0	0
0	1	0	0	0
99	0	0	0	0
1	1	0	0	0
1	1	0	0	0
0	0	0	0	0
0	1	0	0	0
1	1	0	0	0
0	0	0	0	0
0	0	0	0	0
0	1	0	0	0
0	1	0	0	0
0	1	0	0	0
0	1	0	0	0
0	0	0	0	0
0	0	0	0	0
0	1	0	0	0
1	0	0	0	0
0	1	0	0	0
0	1	0	0	0
1	1	0	0	0
0	0	0	0	0
1	0	0	0	0
0	1	0	0	0
1	0	0	0	0
0	1	0	0	0
0	1	0	0	0
0	1	0	0	0
1	1	0	0	0
0	1	0	0	0
1	1	0	0	0
0	1	0	0	0
1	1	0	0	0
0	0	0	0	0
1	1	0	0	0
0	0	0	0	0
0	0	0	0	0
1	1	0	0	0
1	1	0	0	0

0	1	0	0	0
1	1	0	0	0
0	1	0	0	0
0	1	0	0	0
1	1	0	0	0
1	1	0	0	0
1	1	0	0	0
0	1	0	0	0
1	1	0	0	0
1	1	0	0	0
3	1	0	0	0
0	1	0	0	0
1	1	0	0	0
99	1	0	0	0
1	1	0	0	0
0	1	0	0	0
0	1	0	0	0
0	1	0	0	0
0	1	0	0	0
1	1	0	0	0
0	1	0	0	0
1	1	0	0	0
0	1	0	0	0
1	1	0	0	0
1	1	0	0	0
1	1	0	0	0
0	1	0	0	0
1	1	0	0	0
0	1	0	0	0
0	1	0	0	0
1	1	0	0	0
0	1	0	0	0
1	1	0	0	0
0	1	0	0	0
0	1	0	0	0
1	1	0	0	0
0	1	0	0	0
1	1	0	0	0
0	1	0	0	0
0	1	0	0	0
1	0	0	1	0
0	0	0	0	0
1	1	0	0	0
0	0	0	0	0
0	1	0	0	0
0	1	0	0	0
0	0	0	0	0
0	0	0	0	0

0	0	0	0	0
1	0	0	0	0
0	1	0	0	0
0	1	0	0	0
1	0	0	0	0
0	0	0	0	1
1	1	0	0	0
0	1	0	0	0
1	0	0	0	0
0	1	0	0	0
0	1	0	0	0
1	1	0	0	0
1	1	0	0	0
1	1	0	0	0
1	1	0	0	0
0	1	0	0	0
1	1	0	0	0
0	0	0	0	0
1	1	0	0	0
0	1	0	0	0
0	1	0	0	0
1	0	0	1	0
1	0	0	0	0
1	0	0	0	0
0	1	0	0	0
1	1	0	0	0
0	1	0	0	0
1	1	0	0	0
1	1	0	0	0
1	1	0	0	0
0	1	0	0	0
0	1	0	0	0
1	0	0	0	0
1	1	0	0	0
0	1	0	0	0
1	1	0	0	0
0	1	0	0	0
1	1	0	0	0
1	1	0	0	0
1	1	0	0	0
0	1	0	0	0
1	1	0	0	0
0	1	0	0	0
1	1	0	0	0
0	1	0	0	0
1	1	0	0	0
0	1	0	0	0
1	0	0	0	0

0	1	0	0	0
1	1	0	0	0
1	1	0	0	0
0	1	0	0	0
1	1	0	0	0
0	1	0	0	0
1	0	0	0	0
1	1	0	0	0
0	1	0	0	0
1	1	0	0	0
0	1	0	0	0
1	1	0	0	0
0	1	0	0	0
1	1	0	0	0
1	1	0	0	0
0	0	0	0	0
0	1	0	0	0
1	1	0	0	0
0	1	0	0	0
1	0	0	0	0
0	0	0	0	0
1	1	0	0	0
0	1	0	0	0
1	0	0	0	1
0	1	0	0	0
0	1	0	0	0
0	1	0	0	0
1	1	0	0	0
0	1	0	0	0
0	1	0	0	0
0	1	0	0	0
1	1	0	0	0
3	0	0	0	1
1	1	0	0	0
1	1	0	0	0
1	1	0	0	0
0	1	0	0	0
1	1	0	0	0
2	0	0	0	0
3	0	0	0	0
1	1	0	0	0
3	0	0	1	0
0	1	0	0	0
1	1	0	0	0
1	1	0	0	0
1	1	0	0	0
0	1	0	0	0
0	1	0	0	0

1	1	0	0	0
1	1	0	0	0
1	1	0	0	0
0	0	0	0	0
1	1	0	0	0
1	1	0	0	0
0	0	0	0	0
1	1	0	0	0
0	1	0	0	0
0	1	0	0	0
0	0	0	0	0
0	0	0	0	0
0	1	0	0	0
0	1	0	0	0
0	1	0	0	0
0	1	0	0	0
1	1	0	0	0
1	0	0	0	0
1	1	0	0	0
1	1	0	0	0
1	1	0	0	0
1	1	0	0	0
0	1	0	0	0
0	1	0	0	0
1	1	0	0	0
0	1	0	0	0
1	1	0	0	0
1	1	0	0	0
0	1	0	0	0
1	0	0	0	1
1	1	0	0	0
0	1	0	0	0
0	0	0	0	0
1	1	0	0	0
1	1	0	0	0
1	0	0	0	0
0	0	0	0	0
1	0	0	0	0
1	1	0	0	0
1	0	0	0	0
1	1	0	0	0
0	1	0	0	0
0	1	0	0	0
1	1	0	0	0
0	0	0	0	1
1	1	0	0	0
0	1	0	0	0
1	1	0	0	0

1	1	0	0	0
0	1	0	0	0
1	0	0	0	1
1	1	0	0	0
0	1	0	0	0
1	1	0	0	0
0	1	0	0	0
1	1	0	0	0
0	1	0	0	0
1	1	0	0	0
1	0	0	0	0
0	1	0	0	0
1	1	0	0	0
0	1	0	0	0
1	1	0	0	0
1	1	0	0	0
1	0	0	0	0
1	0	0	0	0
0	0	0	0	0
1	1	0	0	0
0	1	0	0	0
0	1	0	0	0
1	1	0	0	0
1	1	0	0	0
3	0	0	0	0
0	1	0	0	0
1	1	0	0	0
0	1	0	0	0
0	1	0	0	0
1	1	0	0	0
0	1	0	0	0
1	1	0	0	0
1	1	0	0	0
1	1	0	0	0
0	1	0	0	0
0	1	0	0	0
1	1	0	0	0
1	0	0	0	0
0	1	0	0	0
0	1	0	0	0
0	1	0	0	0
0	1	0	0	0
0	1	0	0	0
0	1	0	0	0
0	0	0	1	0
0	1	0	0	0
1	1	0	0	0
1	1	0	0	0

0	1	0	0	0
1	1	0	0	0
1	1	0	0	0
0	0	0	0	0
0	0	0	0	0
1	0	0	0	0
0	1	0	0	0
1	1	0	0	0
0	1	0	0	0
1	0	0	0	0
0	1	0	0	0
0	0	0	0	0
1	1	0	0	0
1	1	0	0	0
0	0	0	0	0
0	1	0	0	0
0	1	0	0	0
0	0	0	0	0
1	1	0	0	0
1	1	0	0	0
0	0	0	0	0
1	0	0	0	1
0	1	0	0	0
1	1	0	0	0
0	1	0	0	0
1	1	0	0	0
0	0	0	0	0
1	0	0	0	0
1	0	0	0	0
1	1	0	0	0
1	0	0	0	0
0	1	0	0	0
1	1	0	0	0
1	1	0	0	0
0	1	0	0	0
1	0	0	0	0
0	1	0	0	0
1	0	0	0	0
1	0	0	0	0
1	1	0	0	0
0	1	0	0	0
0	0	0	0	0
0	1	0	0	0
0	1	0	0	0
0	1	0	0	0
1	1	0	0	0
0	1	0	0	0
1	1	0	0	0

0	1	0	0	0
1	1	0	0	0
0	0	0	0	0
0	1	0	0	0
1	1	0	0	0
0	0	0	0	0
0	1	0	0	0
1	1	0	0	0
1	1	0	0	0
0	1	0	0	0
0	1	0	0	0
1	1	0	0	0
0	1	0	0	0
0	1	0	0	0
0	1	0	0	0
0	1	0	0	0
0	1	0	0	0
1	1	0	0	0
1	1	0	0	0
1	1	0	0	0
1	1	0	0	0
0	0	0	0	0
1	0	0	0	0
1	1	0	0	0
1	1	0	0	0
1	0	0	0	0
0	0	0	0	0
1	1	0	0	0
1	0	0	0	1
1	1	0	0	0
1	1	0	0	0
1	0	0	0	1
0	1	0	0	0
1	1	0	0	0
1	1	0	0	0
0	1	0	0	0
1	0	0	0	0
3	1	0	0	0
0	1	0	0	0
0	1	0	0	0
0	1	0	0	0
0	1	0	0	0
0	1	0	0	0
0	1	0	0	0
0	1	0	0	0
1	1	0	0	0
1	1	0	0	0
0	1	0	0	0

1	1	0	0	0
0	1	0	0	0
0	1	0	0	0
1	1	0	0	0
0	1	0	0	0
1	0	0	0	0
0	0	0	0	0
0	1	0	0	0
1	0	0	0	0
0	1	0	0	0
0	1	0	0	0
0	1	0	0	0
1	1	0	0	0
1	1	0	0	0
1	1	0	0	0
1	1	0	0	0
1	1	0	0	0
0	1	0	0	0
1	0	0	0	0
0	1	0	0	0
1	0	0	0	0
1	1	0	0	0
1	0	0	0	1
0	1	0	0	0
1	1	0	0	0
0	1	0	0	0
0	1	0	0	0
0	1	0	0	0
1	1	0	0	0
0	1	0	0	0
0	0	0	0	0
1	1	0	0	0
1	1	0	0	0
1	1	0	0	0
0	1	0	0	0
1	1	0	0	0
0	1	0	0	0
0	0	0	0	0
0	0	0	0	0
1	1	0	0	0
0	1	0	0	0
1	1	0	0	0
1	1	0	0	0
0	0	0	0	0
3	1	0	0	0
1	1	0	0	0
0	1	0	0	0
1	0	0	0	0

0	0	0	0	0
0	0	0	0	0
0	1	0	0	0
0	1	0	0	0
0	1	0	0	0
1	0	0	1	0
1	0	0	0	0
0	1	0	0	0
0	1	0	0	0
0	1	0	0	0
1	1	0	0	0
1	1	0	0	0
0	1	0	0	0
0	1	0	0	0
1	1	0	0	0
1	1	0	0	0
1	1	0	0	0
0	1	0	0	0
0	1	0	0	0
1	1	0	0	0
1	0	0	0	0
1	0	0	0	0
0	1	0	0	0
0	1	0	0	0
1	1	0	0	0
0	1	0	0	0
0	1	0	0	0
0	1	0	0	0
1	1	0	0	0
0	1	0	0	0
1	1	0	0	0
1	1	0	0	0
1	0	0	0	0
0	1	0	0	0
1	1	0	0	0
0	1	0	0	0
1	1	0	0	0
0	1	0	0	0
1	0	0	0	0
0	1	0	0	0
1	0	0	0	0
0	1	0	0	0
0	1	0	0	0
0	0	0	0	0
1	1	0	0	0
3	0	0	0	0
1	0	0	0	0
3	1	0	0	0

1	0	0	0	0
1	1	0	0	0
0	0	0	0	0
0	1	0	0	0
1	1	0	0	0
0	0	0	0	0
1	1	0	0	0
1	1	0	0	0
0	1	0	0	0
1	1	0	0	0
1	1	0	0	0
0	1	0	0	0
1	1	0	0	0
0	1	0	0	0
1	1	0	0	0
0	1	0	0	0
0	1	0	0	0
1	0	0	0	0
0	0	0	0	0
1	1	0	0	0
1	1	0	0	0
0	1	0	0	0
1	1	0	0	0
0	0	0	0	0
1	1	0	0	0
3	1	0	0	0
0	1	0	0	0
0	1	0	0	0
0	1	0	0	0
0	1	0	0	0
0	1	0	0	0
0	1	0	0	0
1	1	0	0	0
1	1	0	0	0
0	1	0	0	0
0	1	0	0	0
1	0	0	0	0
1	1	0	0	0
1	0	0	0	0
1	0	0	0	0
1	1	0	0	0
1	1	0	0	0
0	1	0	0	0
1	0	0	0	0
1	0	0	0	0
1	1	0	0	0
0	1	0	0	0
0	1	0	0	0

0	0	0	0	0
0	1	0	0	0
0	1	0	0	0
0	1	0	0	0
1	0	0	0	1
1	1	0	0	0
1	1	0	0	0
1	1	0	0	0
1	1	0	0	0
0	1	0	0	0
3	0	0	0	1
0	1	0	0	0
0	1	0	0	0
0	1	0	0	0
0	1	0	0	0
3	1	0	0	0
1	0	0	0	0
1	1	0	0	0
0	1	0	0	0
0	1	0	0	0
1	0	0	0	1
1	1	0	0	0
0	0	0	0	0
0	1	0	0	0
0	1	0	0	0
0	1	0	0	0
0	0	0	0	0
1	1	0	0	0
1	0	0	0	0
1	0	0	0	0
0	1	0	0	0
1	0	0	0	0
1	1	0	0	0
0	1	0	0	0
0	0	0	0	0
0	0	0	0	0
0	0	0	0	0
0	1	0	0	0
0	0	0	0	0
1	1	0	0	0
0	1	0	0	0
1	1	0	0	0
0	1	0	0	0
1	1	0	0	0
1	1	0	0	0
1	1	0	0	0
0	1	0	0	0
1	1	0	0	0

0	0	0	0	0
1	1	0	0	0
1	1	0	0	0
1	0	0	0	0
0	0	0	0	0
1	0	0	0	0
1	0	0	0	0
1	0	0	0	0
1	0	0	0	0
0	1	0	0	0
1	1	0	0	0
0	0	0	0	0
1	1	0	0	0
0	1	0	0	0
1	1	0	0	0
1	1	0	0	0
0	1	0	0	0
0	1	0	0	0
0	1	0	0	0
1	1	0	0	0
0	1	0	0	0
1	1	0	0	0
1	1	0	0	0
0	1	0	0	0
1	1	0	0	0
1	1	0	0	0
0	1	0	0	0
1	1	0	0	0
0	1	0	0	0
1	1	0	0	0
0	1	0	0	0
1	1	0	0	0
1	1	0	0	0
0	1	0	0	0
1	0	0	0	0
1	1	0	0	0
1	0	0	0	0
0	1	0	0	0
1	1	0	0	0
0	1	0	0	0
1	1	0	0	0
0	1	0	0	0
1	1	0	0	0
1	1	0	0	0
0	1	0	0	0
1	1	0	0	0
1	1	0	0	0
0	1	0	0	0

0	1	0	0	0
0	1	0	0	0
0	1	0	0	0
1	1	0	0	0
1	1	0	0	0
1	1	0	0	0
1	1	0	0	0
1	0	0	0	1
0	0	0	0	0
0	1	0	0	0
1	0	0	0	0
1	1	0	0	0
1	1	0	0	0
0	1	0	0	0
0	0	0	0	0
0	1	0	0	0
0	1	0	0	0
0	1	0	0	0
0	0	0	0	0
0	1	0	0	0
1	1	0	0	0
0	0	0	0	0
0	0	0	0	0
1	1	0	0	0
0	1	0	0	0
0	1	0	0	0
1	1	0	0	0
1	0	0	0	0
0	0	0	0	0
0	0	0	0	0
0	0	0	0	0
1	1	0	0	0
0	0	0	0	0
0	1	0	0	0
1	1	0	0	0
0	1	0	0	0
1	1	0	0	0
0	0	0	0	0
1	1	0	0	0
1	1	0	0	0
0	1	0	0	0
1	0	0	1	0
0	1	0	0	0
1	0	0	0	1
1	1	0	0	0
0	1	0	0	0
1	0	0	0	0
1	0	0	1	0

1	1	0	0	0
0	0	0	0	0
1	0	0	0	0
0	1	0	0	0
0	1	0	0	0
1	0	0	0	0
0	1	0	0	0
0	1	0	0	0
1	1	0	0	0
1	1	0	0	0
0	1	0	0	0
1	1	0	0	0
0	0	0	0	0
1	1	0	0	0
0	1	0	0	0
0	1	0	0	0
1	1	0	0	0
0	0	0	0	0
1	0	0	0	0
1	0	0	0	1
0	1	0	0	0
1	1	0	0	0
0	1	0	0	0
3	0	0	0	1
0	1	0	0	0
1	0	0	0	0
0	0	0	0	0
1	1	0	0	0
0	1	0	0	0
1	1	0	0	0
1	1	0	0	0
1	1	0	0	0
0	0	0	0	0
0	1	0	0	0
1	1	0	0	0
1	1	0	0	0
1	1	0	0	0
1	0	0	0	0
1	1	0	0	0
0	1	0	0	0
0	1	0	0	0
0	1	0	0	0
0	1	0	0	0
1	1	0	0	0
1	1	0	0	0
0	0	0	0	0
1	1	0	0	0
1	1	0	0	0

0	1	0	0	0
1	1	0	0	0
1	1	0	0	0
1	1	0	0	0
0	1	0	0	0
0	1	0	0	0
0	0	0	0	0
1	1	0	0	0
1	1	0	0	0
1	1	0	0	0
3	0	0	0	1
0	0	0	0	0
0	1	0	0	0
1	0	0	0	0
0	1	0	0	0
1	0	0	0	0
0	1	0	0	0
1	1	0	0	0
0	0	0	0	0
0	1	0	0	0
0	0	0	0	0
0	0	0	0	0
1	1	0	0	0
0	0	0	0	0
1	1	0	0	0
1	1	0	0	0
0	1	0	0	0
1	1	0	0	0
1	1	0	0	0
0	1	0	0	0
1	0	0	1	0
1	0	0	0	0
0	0	0	0	0
0	1	0	0	0
0	1	0	0	0
1	1	0	0	0
1	1	0	0	0
1	1	0	0	0
0	1	0	0	0
0	1	0	0	0
0	1	0	0	0
0	1	0	0	0
0	0	0	0	0
1	1	0	0	0
1	0	0	0	0
0	1	0	0	0
1	0	0	0	0
0	1	0	0	0

1	1	0	0	0
0	1	0	0	0
1	1	0	0	0
0	0	0	0	0
1	1	0	0	0
1	1	0	0	0
0	1	0	0	0
1	0	0	0	1
0	1	0	0	0
0	1	0	0	0
1	1	0	0	0
0	1	0	0	0
1	1	0	0	0
0	1	0	0	0
1	0	0	0	1
0	1	0	0	0
0	1	0	0	0
0	1	0	0	0
0	1	0	0	0
1	1	0	0	0
1	1	0	0	0
1	1	0	0	0
1	1	0	0	0
1	1	0	0	0
1	0	0	0	1
0	1	0	0	0
0	1	0	0	0
0	1	0	0	0
1	0	0	0	1
0	1	0	0	0
0	1	0	0	0
1	1	0	0	0
1	1	0	0	0
1	1	0	0	0
1	1	0	0	0
0	1	0	0	0
0	0	0	0	0
0	1	0	0	0
0	1	0	0	0
3	0	0	0	0
1	0	0	0	0
0	1	0	0	0
0	1	0	0	0
1	1	0	0	0
1	1	0	0	0
0	1	0	0	0
1	1	0	0	0
0	1	0	0	0

0	1	0	0	0
0	1	0	0	0
0	0	0	0	0
0	1	0	0	0
1	1	0	0	0
0	0	0	0	0
1	1	0	0	0
1	1	0	0	0
1	1	0	0	0
1	1	0	0	0
0	0	0	0	0
0	0	0	0	0
0	1	0	0	1
0	0	0	0	0
1	1	0	0	0
0	1	0	0	0
0	1	0	0	0
1	1	0	0	0
0	1	0	0	0
0	1	0	0	0
0	1	0	0	0
0	1	0	0	0
1	1	0	0	0
1	1	0	0	0
0	1	0	0	0
0	1	0	0	0
1	0	0	0	0
0	1	0	0	0
0	0	1	0	0
1	1	0	0	0
1	1	0	0	1
0	1	0	0	0
1	1	0	0	0
0	0	0	0	0
0	1	0	0	0
1	1	0	0	0
0	1	0	0	0
1	1	0	0	0
0	0	0	0	0
1	1	0	0	0
0	1	0	0	0
0	1	0	0	0
0	1	0	0	0
1	1	0	0	0
1	1	0	0	0
1	1	0	0	0
1	1	0	0	0
1	1	0	0	0

0	1	0	0	0
1	0	0	0	1
0	1	0	0	0
0	1	0	0	0
0	1	0	0	0
0	1	0	0	0
1	0	0	0	1
1	1	0	0	0
1	1	0	0	0
1	1	0	0	0
1	0	0	0	1
0	1	0	0	0
1	1	0	0	0
0	1	0	0	0
1	0	0	0	0
0	1	0	0	0
0	1	0	0	0
1	1	0	0	0
1	1	0	0	0
1	1	0	0	0
0	1	0	0	0
1	1	0	0	0
1	0	0	0	1
0	1	0	0	0
1	1	0	0	0
0	1	0	0	0
1	1	0	0	0
0	1	0	0	0
0	1	0	0	0
0	1	0	0	0
1	1	0	0	0
0	1	0	0	0
0	1	0	0	0
1	0	0	0	0
0	1	0	0	0
1	0	0	0	0
1	0	0	0	0
1	1	0	0	0
0	1	0	0	0
0	1	0	0	0
1	0	0	0	1
1	1	0	0	0
0	1	0	0	0
1	1	0	0	0
1	0	0	0	0
1	1	0	0	0
1	1	0	0	0
0	0	0	0	0

1	1	0	0	0
0	1	0	0	0
1	1	0	0	0
0	1	0	0	0
0	1	0	0	0
1	1	0	0	0
0	0	0	0	0
0	1	0	0	0
1	1	0	0	0
1	1	0	0	0
0	0	0	0	0
0	1	0	0	0
1	1	0	0	0
0	1	0	0	0
99	1	0	0	0
0	1	0	0	0
1	1	0	0	0
1	0	0	0	0
1	0	0	0	0
0	1	0	0	0
0	1	0	0	0
1	1	0	0	0
0	0	0	0	0
1	0	0	0	0
0	0	0	0	0
1	1	0	0	0
0	1	0	0	0
1	1	0	0	0
1	1	0	0	0
0	1	0	0	0
1	1	0	0	0
0	1	0	0	0
0	1	0	0	0
0	1	0	0	0
1	1	0	0	0
1	1	0	0	0
0	1	0	0	0
0	1	0	0	0
1	1	0	0	0
1	1	0	0	0
0	1	0	0	0
0	1	0	0	0
1	1	0	0	0
1	1	0	0	0
0	1	0	0	0
1	1	0	0	0
0	1	0	0	0
0	1	0	0	0

1	1	0	0	0
0	1	0	0	0
0	1	0	0	0
1	1	0	0	0
1	1	0	0	0
0	0	0	0	0
0	1	0	0	0
3	1	1	1	1
0	1	0	0	0
0	0	0	0	0
1	0	0	0	0
0	1	0	0	0
0	1	0	0	0
1	1	0	0	0
1	1	0	0	0
0	1	0	0	0
1	1	0	0	0
0	1	0	0	0
1	1	0	0	0
1	1	0	0	0
#NULL!	0	0	0	1
0	1	0	0	0
0	1	0	0	0
0	1	0	0	0
0	1	0	0	0
1	1	0	0	0
0	1	0	0	0
0	1	0	0	0
0	1	0	0	0
0	1	0	0	0
0	1	0	0	0
0	1	0	0	0
0	0	0	0	1
0	1	0	0	0
1	1	0	0	0
1	0	0	0	1
0	1	0	0	0
0	1	0	0	0
1	0	0	0	1
0	1	0	0	0
1	1	0	0	0
0	1	0	0	0
3	0	0	1	0
0	1	0	0	0
0	1	0	0	0
1	1	0	0	0
1	1	0	0	0
0	1	0	0	0

0	1	0	0	0
0	1	0	0	0
1	0	0	0	0
1	0	0	0	0
0	0	0	0	1
0	1	0	0	0
3	0	0	1	1
1	1	0	0	0
1	0	0	0	0
1	1	0	0	0
1	0	0	0	0
3	1	0	0	0
0	1	0	0	0
1	0	0	0	0
0	1	0	0	0
1	1	0	0	0
0	1	0	0	0
1	1	0	0	0
1	1	0	0	0
0	1	0	0	0
0	1	0	0	0
0	1	0	0	0
1	1	0	0	0
1	1	0	0	0
3	0	0	0	0
0	0	0	0	0
1	0	1	0	0
0	0	0	0	0
1	1	0	0	0
0	0	0	0	0
1	1	0	0	0
1	1	0	0	0
1	0	0	0	0
0	0	0	0	0
1	1	0	0	0
1	1	0	0	0
0	1	0	0	0
1	1	0	0	0
1	1	0	0	0
0	0	0	0	0
1	1	0	0	0
0	1	0	0	0
0	1	0	0	0
1	1	0	0	0
1	0	0	0	1
1	0	0	0	1
0	1	0	0	0
1	1	0	0	0

0	1	0	0	0
0	1	0	0	0
1	1	0	0	0
0	1	0	0	0
1	1	0	0	0
1	0	0	0	1
0	0	0	0	0
0	1	0	0	0
0	1	0	0	0
1	1	0	0	0
0	1	0	0	0
0	1	0	0	0
0	1	0	0	0
1	1	0	0	0
1	1	0	0	0
1	1	0	0	0
0	1	0	0	0
1	1	0	0	0
1	1	0	0	0
0	1	0	0	0
1	1	0	0	0

sexorient_pre___5	sexorient_pre___98	sexorient_pre___99	ethnicity_pre	race_pre___1	race_pre___2
0	0	0	1	0	0
0	0	0	1	1	0
0	1	0	1	0	0
0	0	0	0	0	0
0	0	0	0	0	0
0	0	0	99	0	0
0	0	0	1	0	0
0	0	0	1	0	0
0	0	0	1	0	0
0	0	0	0	0	0
0	0	0	0	0	0
0	0	0	0	0	0
0	0	0	0	0	0
0	0	0	0	0	0
0	0	0	0	0	0
0	0	0	1	0	0
0	0	0	1	0	0
0	0	0	1	0	0
0	0	0	1	0	0
0	0	0	0	1	0
0	0	0	1	0	0
0	0	0	0	0	0
0	0	0	1	0	0
0	0	0	1	0	0
0	0	0	1	0	0
0	0	0	1	0	0
0	0	0	1	0	0
0	0	0	0	1	0
0	0	0	1	0	0
0	0	0	1	0	0
0	0	0	1	0	0
0	0	0	0	0	0
0	0	0	1	0	0
0	0	0	1	0	0
0	0	0	1	0	0
0	0	0	1	0	0
0	0	0	1	0	0
0	0	0	1	0	0
0	0	0	99	0	0
0	0	0	1	0	0
0	0	0	1	0	0
0	0	0	0	0	0
0	0	0	0	1	0
0	1	0	0	0	0
0	0	0	0	0	0
0	0	0	0	0	0
0	0	0	0	0	0

0	0	0	1	0	0
0	0	0	0	0	0
0	0	0	1	0	0
0	0	0	99	0	0
0	0	0	1	0	1
0	0	0	0	0	0
0	0	0	0	0	0
0	0	0	1	0	0
0	0	0	1	0	0
0	0	0	0	0	0
0	0	0	1	0	0
1	0	0	0	0	0
0	1	0	1	0	0
1	0	0	0	0	0
0	0	0	0	0	0
0	0	0	0	1	0
0	0	0	0	0	0
0	0	0	1	0	0
0	0	0	0	0	0
0	0	0	1	0	0
0	0	0	1	0	0
0	0	0	1	0	0
0	0	0	0	0	0
0	0	0	0	0	0
0	0	0	0	0	0
0	0	0	1	0	0
0	0	0	0	0	0
0	0	0	1	0	0
0	0	0	1	0	0
0	0	0	1	0	0
0	0	0	0	0	0
0	0	0	1	0	0
0	0	0	0	0	0
0	0	0	0	0	0
0	0	0	1	0	0
0	0	0	1	0	0
0	0	0	1	0	0
0	0	0	1	0	0
0	0	0	1	0	0
0	0	0	0	0	0
0	0	0	1	0	0
0	0	0	1	0	0
0	0	0	1	0	0
0	0	0	1	0	0
0	0	0	1	0	0
0	0	0	0	0	0
0	1	0	0	0	0
0	0	0	1	0	0

0	0	0	99	0	0
0	0	0	1	0	0
0	0	0	1	0	0
0	0	0	0	0	0
1	0	0	1	0	0
0	0	1	0	0	0
0	0	0	99	0	0
0	0	0	0	0	0
0	0	0	1	0	0
0	0	0	0	0	0
0	0	0	1	0	0
0	0	0	0	0	0
0	0	0	0	0	0
0	0	0	0	0	0
0	0	0	1	0	0
0	0	0	1	0	0
0	0	0	0	0	0
0	0	0	1	0	0
0	0	0	1	0	0
0	0	0	1	0	0
0	0	0	1	0	0
0	0	0	0	0	0
0	0	0	99	0	0
0	0	0	0	0	0
0	0	0	1	0	0
0	0	0	1	0	0
0	0	0	1	0	0
0	0	0	0	0	0
0	0	0	1	0	0
0	0	0	1	0	0
0	0	0	99	0	0
0	0	0	0	0	0
0	0	0	1	0	0
0	0	0	0	0	0
0	0	0	1	0	0
0	0	0	1	0	0
0	1	0	1	0	0
0	0	0	1	1	0
0	0	0	0	0	0
0	0	0	0	0	0
0	0	0	0	0	0
0	0	0	0	0	0
0	0	0	1	1	0
0	1	0	0	0	0
0	0	0	0	0	0
0	0	0	0	0	0
0	0	0	0	0	0
0	0	0	1	0	0

0	0	0	1	0	0
0	0	0	1	0	0
0	1	0	0	0	0
0	0	0	0	0	0
0	0	0	0	0	0
0	0	0	0	0	0
0	0	1	1	1	0
0	0	0	1	0	0
0	1	0	1	0	0
0	0	0	1	0	0
0	0	0	1	0	0
0	0	0	0	0	0
0	1	0	1	0	0
0	0	0	0	0	0
0	1	0	1	0	0
0	0	0	1	0	0
0	0	0	0	0	0
0	0	0	1	1	0
0	0	0	1	0	0
0	0	0	1	0	0
0	0	0	1	0	0
0	0	0	1	0	0
0	0	0	0	0	0
0	0	0	0	0	0
0	1	0	1	0	0
0	0	0	0	0	0
0	0	0	0	0	0
0	0	0	0	0	0
0	0	0	0	0	0
0	0	0	0	1	0
0	0	0	1	1	0
1	0	0	1	0	0
0	0	0	0	0	0
0	0	0	1	0	0
0	0	0	0	0	0
0	1	0	1	1	0
0	0	0	0	0	0
0	0	0	1	0	0
0	0	0	0	0	0
0	0	0	0	0	0
0	0	0	1	0	0
0	0	0	1	0	0
0	0	0	0	1	0
0	0	0	0	1	0
0	0	0	0	0	0
0	0	0	0	0	0
0	0	0	1	0	0
0	0	0	0	0	0

0	0	0	1	0	0
0	0	1	0	0	0
0	0	0	1	0	0
0	0	0	1	0	0
0	0	0	0	0	0
0	0	0	0	0	0
0	0	0	1	0	0
0	0	0	0	0	0
0	0	0	1	0	0
0	0	0	0	0	0
0	0	0	1	0	0
0	0	0	1	1	0
0	0	0	1	0	0
0	0	0	0	0	0
0	0	0	1	0	0
0	0	0	0	0	0
1	0	0	0	0	0
0	0	0	1	0	0
0	0	0	0	0	0
0	0	0	1	0	0
0	0	0	0	1	0
0	0	0	1	0	0
0	0	0	1	0	0
0	0	0	1	1	0
0	0	0	0	0	0
0	0	0	0	0	0
0	0	0	0	0	0
0	0	0	1	0	0
0	0	0	1	1	0
0	0	0	1	0	0
0	0	0	1	0	0
0	0	0	1	0	0
0	0	0	1	0	0
0	0	1	1	0	0
0	0	0	0	0	0
0	0	1	0	0	0
0	0	0	1	0	0
0	0	0	1	0	0
0	0	0	1	0	0
0	0	0	1	0	0
0	0	0	0	0	1
0	0	0	0	0	1
0	0	0	0	0	0
0	0	0	1	0	0
0	0	0	0	1	0
0	0	0	0	0	0
0	0	0	0	0	0
0	0	0	1	0	0

0	0	0	0	0	0
0	0	0	1	0	0
0	1	0	0	0	0
0	0	0	1	0	0
0	0	0	0	0	0
0	0	0	1	0	0
0	1	0	0	0	0
0	0	1	1	0	0
0	0	0	0	0	0
0	1	0	1	0	0
0	0	0	0	0	0
0	0	1	0	0	0
0	0	0	1	0	0
0	0	0	1	0	0
0	0	0	1	0	0
0	1	0	1	0	1
0	0	0	1	0	0
0	0	0	99	0	1
0	0	0	99	0	0
0	0	0	0	0	0
0	0	0	1	0	0
1	0	0	0	1	0
0	0	0	1	0	0
0	1	0	99	0	0
0	0	0	0	0	0
0	0	0	0	0	0
0	0	0	0	0	0
0	0	1	1	0	0
0	0	0	0	0	0
0	0	0	1	0	0
0	0	0	1	0	0
0	0	0	1	0	0
0	0	0	1	0	0
0	0	0	1	0	0
0	0	0	0	0	0
0	0	0	1	0	0
1	0	0	1	0	0
0	0	0	0	0	0
0	0	0	0	0	0
0	0	0	1	0	0
0	0	0	1	0	0
0	0	0	1	0	0
0	0	0	1	0	0
0	0	0	1	0	0
0	0	0	1	0	0
0	0	0	1	1	0
0	0	0	0	1	0
1	0	0	1	0	0

0	0	0	1	0	0
0	0	0	99	0	0
0	0	1	0	0	0
0	0	0	99	0	0
0	0	0	0	0	0
0	0	0	1	1	0
0	0	0	0	0	0
0	0	0	0	0	0
0	0	0	1	0	0
0	0	0	1	0	0
0	0	0	0	0	0
0	0	0	1	0	0
0	0	0	1	0	0
0	0	0	0	0	0
0	0	0	0	0	0
0	0	0	0	0	0
0	0	0	1	0	0
0	0	0	1	0	0
0	0	0	1	0	0
0	0	0	0	0	0
0	0	1	0	0	0
0	0	0	0	0	0
0	0	0	1	0	0
0	0	0	0	0	0
0	0	0	1	0	0
0	0	0	1	0	0
0	0	0	0	0	0
0	0	0	1	0	0
0	0	0	0	0	0
0	0	0	1	0	0
0	0	0	0	0	0
0	0	0	1	0	0
0	0	0	0	0	0
0	0	0	1	0	0
0	0	1	99	1	0
0	0	0	0	0	0
0	0	0	1	0	0
0	0	0	1	0	0
0	0	0	1	0	0
0	0	0	1	0	0
0	0	0	1	0	0
0	0	0	1	0	0
0	0	0	1	0	0
0	0	0	1	0	0
0	0	0	0	0	0
0	0	0	1	0	0
1	0	0	1	0	0
0	0	0	1	0	0

0	0	0	1	0	0
0	0	0	1	0	0
0	0	0	1	0	0
0	0	0	0	0	0
0	0	0	1	0	0
0	0	0	99	0	0
0	0	0	1	0	0
0	0	0	0	0	0
0	0	0	0	0	0
0	0	0	1	0	0
0	0	0	0	0	0
0	0	0	1	0	0
0	0	0	0	0	0
0	0	0	0	1	1
1	0	0	1	0	0
0	0	0	1	0	0
0	0	0	1	0	0
0	0	0	1	0	1
0	0	0	1	0	0
0	0	0	1	0	0
0	0	0	1	0	0
1	0	0	1	0	0
0	0	0	0	0	0
0	0	0	0	0	0
0	0	0	1	0	0
0	0	0	0	0	0
0	0	0	0	0	0
0	0	0	99	0	0
0	0	0	1	0	0
0	0	0	0	0	0
0	0	0	0	0	0
0	0	0	0	0	0
0	0	0	1	0	0
0	0	0	0	0	0
0	0	0	0	0	0
0	1	0	1	0	0
0	0	0	0	0	0
0	0	0	0	0	0
0	0	0	1	0	0
0	0	0	0	0	0
0	0	0	0	0	0
0	0	0	1	0	0
0	0	0	0	0	0
0	0	0	1	0	0
0	0	0	0	1	0
0	0	0	0	0	0
0	0	0	0	0	0
0	0	0	1	0	0

0	0	0	0	0	0
0	0	0	1	0	0
0	0	0	1	0	0
0	0	0	0	0	0
0	1	0	0	0	0
0	0	0	0	0	0
0	0	0	99	0	0
0	0	0	0	0	0
0	0	0	1	0	0
0	0	0	0	0	0
0	0	0	0	0	0
0	0	0	0	0	0
0	0	0	1	0	0
0	0	0	1	0	0
0	0	0	0	0	0
0	0	0	0	0	0
0	0	0	0	0	0
1	0	0	0	0	0
0	0	0	1	0	0
0	1	0	0	0	0
0	0	0	1	0	0
0	0	0	0	0	0
0	0	0	1	0	0
0	1	0	99	1	0
0	1	0	1	0	0
0	0	0	0	0	0
0	0	0	1	0	0
0	1	0	0	0	0
0	0	1	1	0	0
0	0	0	1	0	0
1	0	0	1	0	0
0	0	0	1	0	0
0	0	0	0	0	0
0	0	0	1	0	0
0	0	1	1	0	0
0	0	0	1	0	0
0	0	0	0	0	0
0	0	0	0	1	0
0	0	0	1	0	0
0	0	0	1	0	0
0	0	0	0	1	0
0	0	0	1	0	0
0	0	0	1	0	0
0	0	0	0	0	0
0	0	0	0	0	0
0	0	0	1	0	0
0	0	0	1	1	0
0	0	0	1	0	0

0	0	0	1	0	0
0	0	0	0	0	0
0	0	0	1	0	0
0	1	0	1	0	0
0	0	0	1	0	0
0	0	0	1	0	0
0	0	0	0	1	0
0	0	0	1	0	0
0	0	0	0	0	0
0	0	0	1	0	0
0	0	0	0	0	0
0	0	0	1	1	0
0	0	0	1	0	0
0	0	0	1	0	0
0	0	0	1	0	0
0	0	0	0	0	0
0	0	0	1	0	0
0	0	0	0	0	0
0	0	0	0	0	0
0	0	0	1	0	0
0	0	0	0	0	0
0	0	0	0	0	0
0	0	0	1	0	0
0	0	0	1	0	0
0	0	0	1	0	0
0	0	0	1	1	0
0	1	0	0	1	0
0	0	0	1	0	0
0	0	0	1	1	0
0	0	0	1	0	0
0	0	0	1	0	0
0	0	0	1	1	0
0	0	0	1	0	0
0	0	0	0	0	0
0	0	0	0	0	0
0	0	0	1	0	0
0	0	0	1	0	0
0	0	0	1	0	0
0	0	0	1	0	0
0	0	0	0	0	0
0	0	0	1	0	0
0	0	0	1	1	0
0	0	0	1	0	0
0	0	0	99	1	0
0	0	0	0	0	0
1	0	0	0	1	0
0	0	0	1	0	0
0	0	0	1	0	0

0	1	0	1	0	0
1	0	0	1	0	0
0	0	0	1	0	0
0	1	0	99	0	0
0	0	0	0	0	0
0	0	0	0	0	0
0	0	0	99	0	0
0	0	0	1	0	0
0	0	0	0	0	0
0	0	0	0	0	0
0	1	0	0	0	0
0	0	0	0	0	0
0	0	0	1	0	0
0	0	0	0	0	0
0	0	0	1	0	0
0	0	0	1	0	0
0	0	0	1	0	0
0	0	0	1	0	0
0	0	0	1	0	0
0	0	0	1	0	0
0	0	0	1	0	0
0	0	0	1	0	0
0	0	0	1	1	0
0	0	0	1	0	0
0	0	1	99	0	0
0	0	0	0	0	0
0	0	0	99	0	0
0	0	0	1	0	0
0	0	0	0	0	0
0	0	0	0	0	0
0	0	0	1	1	0
0	0	0	0	0	0
0	0	0	0	0	0
0	1	0	1	1	0
0	0	1	1	0	0
0	0	0	1	0	0
0	0	0	1	0	0
0	0	0	1	0	0
0	0	0	1	0	0
0	0	0	1	0	0
0	0	0	0	1	0
0	0	0	1	0	0
0	0	0	0	0	0
0	0	0	1	0	0
0	0	0	0	0	0
0	0	0	0	0	0
0	0	0	1	0	0
0	0	0	1	0	0

0	0	0	1	0	0
0	0	0	1	0	0
0	0	0	1	0	0
0	0	0	0	0	0
0	0	0	0	0	0
0	1	0	0	0	0
0	0	0	0	0	0
0	0	0	1	0	0
0	0	0	1	0	0
0	1	0	0	0	0
0	0	0	1	0	0
0	0	0	1	0	0
0	0	0	0	0	0
0	0	0	0	0	0
0	0	0	1	0	0
0	0	0	1	0	0
0	0	0	1	0	0
0	0	0	0	0	0
0	0	0	1	0	0
0	0	0	1	0	0
0	0	0	0	0	0
0	0	0	1	0	0
0	0	0	0	0	0
0	1	0	1	0	0
0	0	0	0	0	0
0	0	0	1	0	0
0	0	0	1	1	0
0	0	0	1	0	0
0	0	0	1	1	0
0	0	0	0	0	0
0	0	0	1	0	0
0	0	0	1	0	0
0	0	0	0	0	0
0	0	0	0	0	0
0	0	0	1	0	0
0	0	0	1	0	0
0	0	0	1	0	0
0	1	0	0	0	0
0	0	0	1	0	0
0	0	0	1	0	0
0	0	0	99	0	0
0	0	0	0	0	0
0	0	0	1	0	0
0	0	0	0	0	0
0	0	0	1	0	0
0	1	0	1	0	0
0	0	0	1	0	0
0	0	0	1	0	0

0	0	0	1	0	0
0	0	0	0	0	0
0	0	0	1	0	0
0	0	0	1	0	0
0	0	0	1	1	0
0	0	0	1	0	0
0	0	0	1	0	0
0	0	0	1	0	0
0	0	0	1	0	0
0	0	0	1	0	0
0	0	0	0	0	0
0	1	0	0	0	0
0	0	0	1	0	0
0	0	0	0	0	0
0	0	0	1	0	0
0	1	0	0	0	0
0	0	0	99	0	0
0	0	0	0	0	0
0	0	0	0	0	0
0	0	0	0	0	0
0	0	0	0	0	1
0	0	0	1	0	0
0	0	0	1	0	0
0	0	0	0	0	0
0	0	0	1	1	0
0	0	0	1	0	0
0	0	1	0	0	0
0	0	0	1	0	0
0	1	0	0	0	0
0	0	0	0	0	0
0	0	1	1	0	0
0	0	0	0	0	0
0	0	0	1	0	0
0	0	0	1	0	0
0	0	0	99	0	0
0	0	0	1	0	0
0	1	0	1	1	0
0	1	0	0	1	0
0	0	0	1	1	0
0	0	0	0	0	0
0	0	1	1	0	0
0	0	0	1	0	0
0	0	0	0	0	0
0	0	0	0	0	0
0	0	0	1	0	0
0	0	0	0	0	0
0	1	0	1	0	0
0	0	0	1	0	0

0	1	0	1	0	0
0	0	0	1	0	0
0	0	0	1	0	0
0	1	0	1	0	0
0	0	0	0	0	0
0	1	0	0	0	0
0	0	0	1	0	0
0	0	0	0	0	0
0	0	0	0	0	0
0	0	0	1	0	0
0	0	0	1	0	0
0	0	0	0	0	0
0	0	0	0	1	0
0	0	0	0	0	0
0	0	0	0	0	0
0	0	0	0	1	0
0	0	0	0	0	0
0	0	0	0	1	0
0	0	0	1	0	0
0	0	0	0	0	0
0	0	0	0	0	0
0	0	0	1	0	0
0	0	0	1	0	0
0	0	0	1	0	0
0	0	0	1	0	0
0	0	0	1	0	0
0	0	0	0	0	0
0	0	0	1	0	0
0	0	0	1	0	0
0	0	0	0	0	0
0	0	1	1	0	0
0	0	0	0	0	0
0	0	0	1	1	0
0	0	0	1	0	0
0	0	0	1	0	0
0	0	0	1	0	0
0	0	0	0	0	0
0	0	0	0	0	0
0	0	0	0	0	0
1	0	0	1	0	0
0	0	0	99	0	0
0	0	0	1	0	0
0	0	0	0	0	0
0	0	0	0	0	0
0	0	0	1	0	0
0	0	0	1	0	0
0	0	0	1	0	0
0	0	0	0	0	0

0	0	0	1	0	0
0	0	0	1	0	0
0	0	0	1	0	0
0	0	0	1	0	0
0	0	0	0	0	0
0	0	0	1	1	0
1	0	0	0	1	0
0	0	0	0	0	0
0	0	0	1	0	0
0	0	0	0	0	0
0	0	0	0	0	0
0	0	0	1	0	0
0	0	0	1	0	0
0	0	0	1	1	0
0	0	0	1	0	0
0	0	0	0	0	0
0	0	0	1	0	0
0	1	0	0	1	0
0	0	0	0	0	0
0	0	0	1	0	0
0	0	0	1	0	0
0	0	0	0	0	0
0	0	0	0	0	0
0	0	0	0	1	0
0	0	0	1	0	1
0	0	0	0	0	0
0	0	0	1	0	0
1	0	0	1	0	0
0	0	0	1	0	0
0	1	0	0	0	0
0	0	0	1	0	0
0	0	0	1	0	0
0	1	0	1	0	0
0	0	0	0	0	0
0	1	0	1	0	0
0	0	0	99	0	0
0	0	0	1	0	0
0	0	0	99	0	0
0	0	0	0	0	0
0	0	0	0	0	0
0	1	0	99	0	0
0	0	0	0	0	0
0	0	0	0	0	0
0	0	0	1	0	0
0	0	0	1	0	0
0	0	0	0	0	0
0	1	0	1	0	0
0	0	0	1	0	0

0	1	0	0	0	0
0	0	1	1	0	0
0	0	0	0	0	0
0	1	0	1	0	0
0	1	0	1	0	0
0	0	0	0	0	0
0	0	0	0	0	0
0	0	0	0	0	0
0	0	0	1	0	0
0	1	0	0	0	0
0	0	0	1	0	0
0	0	0	0	1	0
0	1	0	1	0	0
0	0	0	0	0	0
0	0	0	1	0	0
0	0	0	0	0	0
0	0	0	0	0	0
0	0	1	1	0	0
0	1	0	0	0	0
0	1	0	1	0	0
0	0	0	0	0	0
0	0	0	1	0	0
0	0	0	0	0	0
0	0	0	0	0	0
0	0	0	1	0	0
0	0	0	0	0	0
0	0	0	1	0	0
0	0	0	0	0	0
0	0	0	1	0	0
0	0	0	0	0	0
0	0	0	1	0	0
0	0	0	1	0	0
0	0	0	1	0	0
0	1	0	1	0	0
0	0	0	0	0	0
0	0	0	1	0	0
0	0	0	1	0	0
1	0	0	1	0	0
0	1	0	0	0	0
0	1	0	1	0	0
0	0	0	1	0	0
0	0	0	1	0	0
0	0	0	0	1	0
0	1	0	1	0	0
0	0	0	1	0	0
0	0	0	1	0	0
0	0	0	1	0	0
0	0	0	1	0	0

0	0	0	1	0	0
0	0	0	1	0	0
0	1	0	0	0	0
0	0	0	1	0	0
0	0	0	1	0	0
0	0	0	1	0	0
0	0	0	1	0	0
0	0	0	0	0	0
0	0	1	0	0	0
0	0	0	1	0	0
0	0	0	0	0	0
0	0	0	0	0	0
0	0	0	1	0	0
0	0	0	1	0	0
0	0	0	0	0	0
0	0	0	0	1	0
0	0	0	1	0	0
0	0	0	1	0	0
0	0	0	1	0	0
0	0	0	1	0	0
0	0	0	1	0	0
0	0	0	0	0	0
0	0	0	1	0	0
0	0	0	1	0	0
0	0	0	0	0	0
0	0	0	0	0	0
0	0	1	1	0	0
0	1	0	1	0	0
0	0	0	0	1	0
0	0	0	1	0	0
0	0	0	1	0	0
0	0	0	1	0	0
0	0	0	1	0	0
0	0	0	1	0	0
0	0	0	0	1	0
1	0	0	1	0	0
0	0	0	99	0	0
0	0	0	1	0	0
0	0	0	99	0	0
0	0	0	0	0	0
0	1	0	0	0	0
0	1	0	1	0	0
0	0	0	1	0	0
0	0	0	1	1	0
0	0	0	0	1	0
1	0	0	1	1	0
0	0	0	1	1	0
0	1	0	1	0	0

0	0	0	1	0	0
0	0	0	0	0	0
0	0	0	0	0	0
0	0	0	0	0	0
0	0	0	0	0	0
0	0	0	1	0	0
0	0	0	1	0	0
0	0	0	1	0	0
0	0	0	0	1	0
0	0	0	1	0	0
0	0	0	0	0	0
0	0	0	0	0	0
1	0	0	1	0	0
0	1	0	0	0	0
0	0	0	1	0	0
0	0	0	1	0	0
0	0	0	1	1	0
0	0	0	1	0	0
0	0	0	1	0	0
0	0	0	1	0	0
0	0	0	1	0	0
0	0	1	1	1	0
0	0	0	0	0	0
0	0	0	1	0	0
0	0	0	1	0	0
0	0	0	1	0	0
0	0	1	1	0	0
0	0	0	0	0	0
0	0	0	0	0	0
0	0	0	0	0	0
0	0	0	0	0	0
0	1	0	1	0	0
0	0	1	99	0	0
0	0	0	1	0	0
0	0	0	0	0	0
0	0	0	0	0	0
0	0	0	1	0	0
0	0	0	1	0	0
0	0	0	0	0	0
0	0	0	0	0	0
0	0	0	1	0	0
0	0	0	1	0	0
0	0	0	1	0	0
0	0	0	1	0	0
0	0	0	1	0	0
0	0	0	1	0	0
0	0	0	1	0	0
0	0	0	1	0	0

0	0	0	0	0	0
0	0	0	1	0	0
0	1	0	1	0	0
0	0	0	0	0	0
0	0	0	1	0	0
0	0	0	1	0	0
0	0	0	1	0	0
0	0	0	1	0	0
0	0	0	0	0	0
0	0	0	1	0	0
0	0	0	0	0	0
0	0	0	1	1	0
0	0	0	0	0	0
0	0	0	0	0	0
0	0	0	0	0	0
0	0	0	0	1	0
0	0	0	1	0	0
0	0	0	1	0	0
0	1	0	0	0	0
0	0	0	1	0	0
0	0	0	1	0	0
0	1	0	1	1	0
0	1	0	1	0	0
0	0	0	1	0	0
0	1	0	1	1	0
0	0	0	0	0	0
0	0	0	1	0	0
0	0	0	0	0	0
0	0	1	1	0	0
0	0	0	0	0	0
1	0	0	0	0	0
0	0	0	1	0	0
0	0	0	0	0	0
0	0	0	1	0	0
0	0	0	0	0	0
0	0	0	0	0	0
0	0	1	1	1	0
0	0	0	1	0	0
0	0	0	0	0	0
0	0	0	1	0	0
0	0	1	1	0	0
0	0	0	1	0	0
0	0	0	0	0	0
0	0	0	1	0	0
0	0	0	1	0	0
0	0	0	99	1	1
0	0	0	1	0	0
0	0	0	1	0	0

0	0	0	0	1	0
0	1	0	1	1	0
0	0	0	1	0	0
0	0	0	0	0	0
0	0	0	0	0	0
0	0	0	0	0	0
0	0	0	0	0	0
0	0	1	0	0	0
0	0	1	0	0	0
0	1	0	0	0	0
0	0	0	1	0	0
0	1	0	0	0	0
0	0	0	1	0	0
0	0	0	1	0	0
0	0	1	1	0	0
0	0	0	0	0	0
1	0	0	1	0	0
0	0	0	1	0	0
0	0	0	1	0	0
0	0	0	1	0	0
0	0	0	0	0	0
0	0	0	1	0	0
0	0	0	99	0	0
0	0	0	0	0	0
0	0	0	0	0	0
0	0	1	0	0	0
0	0	0	99	0	0
0	0	0	1	1	0
0	0	0	0	0	0
0	0	0	1	0	0
0	0	0	1	0	0
0	1	0	1	0	0
0	0	0	1	0	0
0	0	0	1	0	0
0	1	0	1	0	0
0	0	0	1	0	0
0	0	0	0	0	0
0	0	0	0	0	0
0	0	0	1	0	0
0	0	0	1	0	0
0	0	0	0	0	0
1	0	0	1	0	0
0	0	0	0	1	0
0	0	0	1	0	0
0	0	0	1	0	0
0	0	0	0	0	0
0	0	0	1	0	0
0	0	0	99	0	0

0	0	0	0	0	0
0	0	0	1	0	0
0	0	0	0	0	0
0	0	0	0	0	0
0	0	0	1	0	0
0	0	0	1	0	0
0	0	0	1	0	0
0	0	0	0	0	0
0	1	0	0	0	0
0	0	0	1	0	0
0	0	0	0	1	0
0	0	0	1	0	0
0	0	0	0	0	0
0	0	0	0	0	0
0	1	0	0	0	0
0	1	0	0	1	0
0	0	0	1	0	0
0	1	0	0	0	0
0	0	0	1	1	0
0	0	0	1	0	0
0	0	0	0	0	0
0	0	0	0	0	0
0	0	0	1	0	0
1	0	0	0	0	0
0	0	0	1	0	0
0	0	0	1	0	0
0	0	0	0	0	0
0	0	0	1	0	0
0	0	0	1	0	0
0	0	0	0	0	0
0	0	0	1	0	0
0	0	0	0	0	0
0	0	0	1	0	0
0	1	0	0	0	0
0	0	0	1	1	0
0	1	0	1	0	0
0	0	0	1	0	1
0	0	0	0	0	1
0	0	0	1	0	0
0	0	0	1	0	0
0	0	0	1	1	0
0	0	0	1	1	0
0	0	0	1	0	0
1	0	0	1	0	0
0	0	0	1	0	0
0	0	0	1	0	0
1	0	0	1	0	0
0	0	0	0	0	0

0	0	0	0	0	0
0	0	0	1	0	0
0	0	0	1	0	0
0	0	0	1	0	0
0	0	0	0	0	0
0	0	0	1	0	0
0	0	0	1	0	0
0	0	0	0	0	0
0	0	0	1	0	0
0	0	0	1	0	0
0	0	0	1	0	0
0	0	0	1	0	0
0	0	0	1	0	0
0	0	0	0	0	0
0	0	0	0	0	0
0	0	0	1	1	0
0	0	0	0	0	0
0	0	0	0	0	0
0	0	0	0	0	0
0	0	0	0	0	0
0	0	0	1	0	0
0	0	0	1	1	0
0	0	0	0	0	0
0	0	0	0	0	0
1	0	0	0	0	0
1	0	0	0	0	0
0	0	0	1	0	0
0	0	0	1	0	0
0	0	0	0	0	0
0	0	0	1	0	0
0	0	1	1	0	0
0	0	0	1	1	0
1	0	0	0	0	0
0	0	0	0	0	0
0	0	0	1	0	0
0	0	0	0	0	0
0	0	0	0	0	0
0	0	0	1	0	0
0	0	0	0	0	0
0	0	0	0	0	0
0	0	0	0	0	0
0	0	0	0	0	0
0	0	0	0	0	0
0	0	0	1	0	0
0	0	0	0	1	0
0	0	0	0	0	0
0	0	0	0	0	0
0	0	0	1	0	0

1	0	0	1	0	0
0	1	0	1	0	0
0	0	0	1	0	0
0	0	0	1	1	0
0	0	0	1	0	0
0	0	0	1	0	0
0	0	0	0	1	0
0	0	0	1	0	0
0	0	0	0	0	0
0	0	0	0	1	0
0	0	0	1	0	0
0	0	0	1	0	0
0	0	0	1	0	0
0	0	0	1	0	0
0	0	1	1	0	0
0	0	0	1	1	0
0	0	0	0	0	0
0	0	0	0	0	0
0	0	0	1	0	0
0	0	0	1	0	0
0	0	0	0	0	0
0	0	0	0	0	0
0	0	0	1	0	0
0	0	0	0	1	0
0	0	0	0	0	0
0	0	0	0	1	0
0	1	0	1	1	0
0	0	0	0	0	0
0	0	0	1	0	0
0	0	0	0	0	0
0	0	0	1	0	0
1	0	0	1	0	0
0	0	0	1	0	0
0	0	0	0	0	0
0	0	0	1	0	0
1	0	0	1	0	0
0	0	0	0	0	0
0	0	0	1	0	0
0	0	0	0	1	0
0	0	0	0	0	0
0	0	0	1	0	0
0	0	0	0	0	0
0	0	0	1	0	0
0	0	0	0	0	0
0	0	0	0	0	0
0	0	0	1	0	0
0	0	0	1	0	0
0	0	0	1	0	0

0	0	0	1	0	0
0	0	0	1	0	0
0	0	0	1	0	0
0	0	0	0	0	0
0	0	0	0	0	0
0	0	0	1	0	0
0	0	0	1	0	0
0	0	0	1	0	0
1	0	0	1	0	0
0	0	0	0	1	0
0	0	1	1	0	0
0	0	0	1	0	0
0	0	0	1	0	0
0	1	0	1	0	0
0	0	0	0	0	0
0	0	0	1	0	0
0	0	0	0	0	0
0	0	0	1	0	0
1	0	0	0	0	0
0	0	0	0	0	0
0	0	0	0	0	0
0	0	0	1	0	0
0	0	0	0	0	0
0	0	0	1	0	0
0	0	0	0	0	0
0	0	0	1	0	0
0	0	0	1	0	0
0	0	0	1	0	0
0	0	0	1	0	0
0	0	0	1	0	0
0	0	0	1	0	0
0	0	0	1	0	0
0	0	0	0	0	0
0	0	0	0	0	0
0	0	0	0	0	0
0	0	0	1	0	0
0	0	1	0	0	0
0	0	0	1	0	0
0	0	0	1	0	0
0	0	0	0	0	0
0	0	0	1	0	0
0	0	0	1	0	0
0	0	0	0	0	0
0	0	0	0	0	0
0	0	0	1	0	0
0	0	0	1	0	0
0	0	0	0	0	0
0	0	0	1	0	0

0	0	0	0	0	0
0	0	0	0	0	0
0	0	0	0	0	0
0	0	0	1	0	0
0	0	0	0	0	0
0	0	0	0	1	0
0	0	0	1	0	0
0	0	0	1	0	0
0	0	0	0	0	0
0	0	0	1	0	0
0	0	0	1	0	0
0	0	0	0	0	0
0	0	0	1	0	0
0	0	0	0	0	0
0	1	0	0	0	0
0	0	0	1	0	0
0	0	0	0	0	0
0	0	0	0	1	0
0	0	0	1	0	0
0	0	0	0	0	0
0	0	0	0	1	0
0	0	0	0	0	0
0	0	0	1	0	0
0	0	0	1	0	0
1	0	0	0	0	0
0	0	0	0	0	0
0	0	0	1	1	0
0	0	0	1	0	0
0	0	0	0	0	0
0	0	1	1	0	0
0	0	0	1	1	0
0	1	0	1	0	0
1	0	0	0	0	0
0	0	0	0	0	0
0	0	0	0	0	0
0	0	0	1	0	0
0	1	0	0	0	0
1	0	0	1	0	0
0	0	0	0	0	0
0	1	0	1	0	0
0	0	0	0	0	0
0	0	0	0	0	0
0	1	0	1	0	0
0	0	0	1	0	0
0	0	0	1	0	0
0	0	0	0	0	0
0	0	0	1	0	0
0	0	0	1	0	0

0	0	1	0	0	0
0	0	0	0	0	0
0	0	0	1	0	0
0	0	0	0	0	0
0	0	0	0	0	0
0	1	0	1	0	0
0	0	1	1	0	0
0	0	0	1	0	0
0	0	0	0	1	0
0	0	0	1	1	0
0	0	0	0	0	0
0	1	0	0	0	0
0	0	0	1	0	0
0	0	0	0	0	0
0	0	0	0	0	0
1	0	0	0	0	0
0	0	0	1	1	0
0	0	0	1	0	0
0	0	0	0	0	0
0	0	0	0	0	0
0	0	0	1	0	0
0	0	0	1	0	0
0	0	0	0	0	0
0	0	0	1	0	0
0	0	0	1	0	0
0	0	0	0	0	0
0	0	0	1	0	0
0	0	0	1	0	0
0	0	0	1	0	0
0	0	0	1	0	0
0	0	0	0	0	0
0	0	0	0	0	0
0	0	0	0	0	0
0	0	0	1	1	0
0	0	0	0	0	0
0	0	0	1	0	0
0	0	0	0	0	0
0	0	0	0	0	0
0	0	0	1	0	0
0	0	0	1	0	0
0	0	0	0	0	0
0	0	0	0	0	0
0	0	0	1	0	0
0	0	0	0	0	0
0	0	1	1	0	0
0	0	0	1	0	0
0	0	0	0	0	0
0	1	0	0	0	0

0	0	0	1	0	0
0	0	0	1	0	0
0	0	0	1	0	0
0	0	0	1	0	0
0	0	0	0	0	0
0	0	0	0	0	0
1	1	0	0	0	0
0	0	0	1	0	0
0	0	0	0	0	0
0	0	0	0	1	0
0	0	0	1	0	0
0	0	0	0	0	0
0	0	0	1	0	0
0	0	0	1	0	0
0	0	0	1	0	0
0	0	0	1	0	0
1	0	0	0	0	0
0	0	0	1	0	0
0	0	0	0	0	0
0	0	0	0	0	0
0	0	0	1	0	0
0	0	0	1	0	0
0	0	0	0	0	0
0	0	0	0	0	0
1	0	0	0	0	0
0	0	0	0	0	0
0	0	0	0	1	0
0	0	0	1	0	0
1	0	0	99	0	0
0	0	0	0	0	0
0	0	0	0	0	0
0	0	0	0	0	0
0	1	0	0	0	0
0	0	0	0	1	0
0	0	0	0	0	0
0	0	0	1	0	0
0	0	0	99	0	0
0	0	0	99	0	0
0	0	0	0	1	0
0	1	0	0	0	0
0	0	0	1	0	0
0	0	0	0	0	0
1	0	0	0	0	0
0	1	0	1	0	0
0	0	0	1	0	0
1	0	0	1	0	0
0	0	0	0	0	0
0	0	0	0	1	0

0	0	0	0	0	0
0	0	0	0	0	0
0	0	1	0	0	0
0	0	0	0	0	0
0	0	0	0	0	0
1	0	0	0	0	0
0	0	0	1	0	0
0	0	0	1	0	0
0	1	0	1	1	0
0	0	0	0	0	0
0	0	0	1	0	0
0	0	0	0	0	0
0	0	1	1	0	0
0	1	0	0	0	0
0	0	0	1	0	0
0	0	0	1	0	0
0	0	0	0	0	0
0	0	0	0	0	0
0	0	0	0	0	0
0	0	0	1	0	0
0	0	0	0	0	0
0	0	0	0	0	0
0	1	0	0	0	0
0	0	0	1	1	0
0	0	1	1	0	0
0	0	0	1	0	0
0	0	0	1	0	0
0	0	0	1	0	0
0	0	0	1	0	0
0	0	0	99	0	0
1	0	0	1	0	0
0	0	0	0	0	0
0	0	0	0	0	0
0	0	0	1	0	0
0	0	0	1	0	0
0	0	0	0	0	0
0	0	0	0	0	0
0	0	0	1	0	0
0	1	0	1	1	0
0	0	0	1	0	0
0	1	0	0	0	0
0	0	0	0	0	0
0	0	0	0	0	0
0	0	0	0	0	0
0	0	0	0	0	0
0	0	0	1	0	0
1	0	0	0	0	0
0	0	0	1	0	0

0	0	0	0	0	0
0	0	0	0	0	0
0	0	0	1	0	0
0	0	1	0	0	0
0	0	0	0	0	0
0	0	0	0	0	0
0	0	1	0	0	0
0	0	0	0	0	0
0	0	0	0	0	0
0	1	0	1	0	0
0	0	0	1	0	0
0	0	0	1	0	0
0	1	0	0	0	0
0	0	0	0	0	0
0	0	0	0	0	0
0	0	0	0	0	0
0	0	0	0	0	0
0	0	0	0	0	0
0	0	0	0	0	0
0	0	0	0	0	0
0	0	0	0	0	0
0	0	0	99	0	0
0	1	0	0	0	0
0	0	0	0	0	0
0	0	1	99	0	0
0	0	0	1	0	0
0	0	0	1	0	0
0	0	0	1	0	0
0	0	0	1	0	0
0	0	0	1	0	0
0	0	0	0	0	0
0	0	0	0	0	0
0	0	0	1	0	0
0	0	0	0	0	0
0	0	0	1	0	0
0	0	0	1	0	0
0	0	0	0	0	0
0	0	0	1	0	0
0	0	0	0	0	0
0	0	0	1	0	0
0	0	0	1	0	0
0	0	0	0	0	0
0	0	0	1	0	0
0	0	0	0	0	0
0	0	0	0	0	0
0	0	0	1	0	0
0	0	0	1	0	0
0	0	0	1	0	0

1	0	0	1	0	0
0	0	0	1	0	0
0	0	0	0	0	0
0	0	0	1	0	0
0	1	0	0	0	0
0	0	0	1	0	0
0	0	0	1	0	0
0	0	0	0	0	0
0	0	0	1	0	0
0	0	0	0	1	0
0	1	0	0	0	0
0	0	1	0	0	0
0	0	0	1	0	0
0	0	0	0	0	0
0	0	0	1	1	0
0	0	0	0	0	0
0	0	0	1	0	1
0	0	1	0	0	0
0	0	0	0	0	0
0	1	0	0	0	0
0	1	0	0	0	0
0	0	0	0	0	0
0	0	0	1	0	0
0	1	0	0	0	0
0	0	0	0	0	0
0	0	0	0	0	0
0	0	0	0	0	0
1	0	0	0	0	0
0	0	0	1	0	0
0	1	0	1	1	0
0	0	0	0	0	0
1	0	0	0	0	0
0	1	0	0	0	0
0	0	0	1	0	0
0	0	0	0	0	0
0	0	0	0	0	0
0	0	0	0	0	0
0	0	0	0	0	0
0	1	0	1	0	0
0	0	0	0	0	0
0	0	0	1	0	0
0	0	0	0	0	0
0	0	0	0	0	0
0	0	0	0	1	0
0	0	0	1	0	0
0	0	0	1	0	0
0	0	0	0	0	0
0	0	0	99	0	0

0	0	0	0	0	0
0	0	0	0	0	0
0	0	0	1	0	0
0	0	0	0	1	0
0	0	0	0	0	0
0	0	0	1	0	0
0	0	0	0	0	0
0	0	0	1	0	0
0	0	0	0	0	0
0	0	0	1	0	0
0	0	0	1	0	0
0	0	0	1	0	0
0	0	0	0	0	0
0	0	0	1	0	0
0	1	0	0	1	0
0	0	0	1	0	0
0	0	0	1	0	0
0	1	0	1	0	0
0	0	0	0	0	0
0	0	0	0	0	0
0	0	0	1	1	0
0	0	0	0	0	0
0	0	0	0	0	0
0	0	0	1	0	0
0	0	0	0	0	0
0	0	0	1	0	0
0	0	0	1	0	0
0	0	0	0	0	0
0	0	0	0	0	0
0	0	0	0	0	0
0	0	0	0	0	0
0	0	0	0	0	0
0	0	0	1	0	0
0	0	0	1	0	0
0	0	0	0	0	0
0	0	0	1	0	0
0	0	0	0	0	0
1	0	0	0	0	0
0	0	0	0	0	0
0	0	0	0	0	0
0	0	0	0	0	0
0	0	0	0	0	0
0	0	0	1	0	0
0	0	0	0	0	0
0	0	0	0	0	0
0	0	1	0	0	0
0	0	0	0	0	0
0	0	0	0	0	0

0	0	0	0	0	0
0	0	0	0	0	0
0	0	0	1	0	0
0	0	0	0	0	0
0	0	0	0	0	0
0	0	0	1	0	0
0	0	0	1	0	0
0	0	0	0	0	0
0	0	0	0	0	0
0	0	0	0	0	0
0	0	0	1	0	0
0	0	0	0	0	0
0	0	0	0	0	0
0	0	0	1	0	0
0	0	0	0	0	0
1	0	0	1	0	0
0	1	0	1	0	0
0	0	0	1	1	0
0	0	0	0	1	0
0	0	0	1	0	0
0	1	0	1	0	0
0	1	0	1	0	0
0	0	0	0	0	0
0	0	0	1	0	0
0	0	0	1	0	0
1	0	0	1	0	0
0	0	0	1	0	0
1	0	0	0	0	0
1	0	0	0	0	0
0	0	0	1	1	0
0	0	0	1	0	0
0	0	0	0	0	0
0	0	0	1	0	0
1	0	0	0	0	0
0	1	0	99	0	0
0	0	0	0	0	0
0	0	0	0	0	0
0	0	0	1	0	0
0	0	0	0	1	0
1	0	0	0	0	0
0	1	0	1	0	0
0	1	0	1	0	0
0	0	0	0	0	0
0	0	0	0	0	0
0	1	0	0	0	0
0	0	0	0	0	0
0	1	0	0	0	0
0	0	0	0	0	0

0	0	0	0	0	0
0	0	0	0	0	0
0	0	0	0	0	0
0	1	0	1	0	0
0	1	0	0	0	0
0	1	0	0	0	0
0	0	0	1	0	0
1	0	0	1	0	0
0	0	0	0	0	0
0	0	0	0	0	0
0	0	0	1	0	0
0	0	0	1	0	0
0	0	0	0	0	0
1	0	0	1	0	0
0	1	0	1	0	0
0	0	0	1	0	0
0	0	1	0	0	0
0	0	0	0	0	0
0	0	0	1	0	0
0	0	0	1	0	0
0	0	0	1	0	0
0	0	0	1	0	0
0	0	0	1	0	0
0	0	0	0	0	0
0	0	0	1	1	0
0	0	0	0	0	0
0	1	0	1	1	0
0	0	0	1	1	0
0	0	0	1	0	0
0	0	0	0	0	0
0	0	0	0	0	0
0	0	0	1	0	0
0	0	0	1	0	0
0	0	0	0	0	0
0	0	0	1	0	0
0	0	0	1	1	0
0	0	0	0	0	0
0	0	0	0	0	0
0	0	0	1	0	0
0	1	0	1	0	0
0	0	0	1	0	0
0	0	0	0	0	0
0	0	0	1	0	0
0	0	1	0	0	0
0	0	0	0	0	0
0	0	0	0	0	0
0	0	0	0	0	0
0	0	0	1	0	0

0	0	0	0	0	0
0	0	0	0	0	0
0	0	0	1	1	0
0	0	0	0	0	0
0	0	0	0	0	0
1	0	0	1	0	0
0	0	0	1	0	0
0	0	0	0	0	0
0	0	0	1	0	0
0	0	0	1	0	0
0	0	0	1	0	0
0	0	0	1	1	0
0	0	0	0	0	0
0	0	0	1	0	0
0	0	0	0	0	0
0	0	0	0	0	0
0	0	0	1	0	0
0	0	0	1	1	0
0	0	0	0	0	0
0	0	0	0	0	0
0	0	0	1	1	0
0	0	0	0	0	0
0	0	0	0	0	0
0	0	0	1	0	0
0	0	0	1	0	0
0	0	0	0	0	0
0	0	0	99	0	0
0	0	0	1	0	0
0	1	0	0	0	0
0	0	0	1	0	0
0	0	0	0	0	0
0	1	0	0	0	0
0	1	0	0	0	0
0	0	0	1	0	0
0	0	0	99	0	0
0	0	0	0	0	0
1	0	0	0	0	0
0	0	0	0	0	0
0	0	0	1	0	0
0	0	0	0	0	0
0	0	0	0	0	0
1	0	0	0	0	0
0	0	0	0	0	0
1	0	0	0	0	0
0	0	1	99	0	0
0	0	1	0	0	0
0	0	0	1	0	0
0	1	0	0	0	0

0	1	0	0	0	0
0	0	0	1	0	0
0	0	0	1	0	0
0	0	0	1	0	0
0	0	0	1	0	0
0	0	0	0	0	0
0	1	0	0	0	0
0	1	0	1	0	0
0	0	0	0	1	0
0	0	0	1	1	0
0	0	0	0	0	0
0	0	1	99	0	0
0	0	0	0	0	0
0	0	0	1	0	0
0	1	0	0	1	0
0	0	0	1	0	0
0	0	0	1	0	0
1	0	0	1	0	0
1	0	0	1	0	0
0	0	0	1	0	0
0	0	0	0	0	1
0	1	0	0	0	0
0	0	0	1	0	0
0	1	0	1	0	0
0	0	1	0	0	0
0	0	0	1	0	0
0	1	0	1	0	0
0	0	0	0	0	0
0	0	0	1	0	1
0	0	0	1	0	0
0	1	0	0	0	0
0	0	1	0	0	0
0	0	0	0	0	0
1	0	0	1	0	0
0	0	0	1	0	0
0	0	0	0	0	0
0	0	0	0	0	0
0	0	0	1	1	0
0	0	0	0	0	0
0	0	0	0	0	0
0	0	0	0	0	0
0	0	0	1	0	0
0	1	0	1	0	0
0	0	0	1	1	0
0	1	0	1	0	0
0	1	0	1	0	0
0	0	0	1	0	0
0	0	0	0	0	0

0	0	0	0	0	0
0	0	0	1	1	0
0	0	0	0	0	0
0	0	0	0	0	0
0	0	0	1	0	0
0	0	0	1	0	0
0	0	0	1	0	0
0	0	0	1	0	0
0	0	0	1	0	0
0	0	0	0	0	0
0	0	0	0	0	0
0	0	0	0	1	0
0	0	0	1	1	0
0	0	0	0	0	0
0	0	0	1	0	0
0	0	0	1	0	0
0	0	0	0	1	0
0	0	0	1	0	0
0	0	0	0	1	0
0	0	0	1	0	0
0	0	0	0	0	0
0	0	0	1	0	0
0	0	0	0	0	0
0	0	0	0	0	0
0	0	0	0	0	0
0	0	0	0	0	0
0	0	0	0	0	0
0	0	0	1	0	0
0	0	0	1	0	0
0	0	0	1	0	0
0	0	0	1	0	0
0	0	0	0	0	0
0	0	0	0	0	0
0	0	0	0	1	0
0	0	0	1	0	0
0	0	0	0	0	0
0	0	0	1	0	0
0	0	0	0	0	0
0	0	0	1	0	0
0	0	0	0	0	0
0	0	0	0	0	0
0	1	0	0	0	0
0	0	0	1	0	0
0	1	0	0	0	0
0	0	0	0	0	0
0	0	0	0	0	0
0	0	1	0	0	0
0	1	0	1	0	0

0	0	1	0	0	0
0	1	0	0	0	0
0	0	0	1	0	0
0	0	0	0	0	0
0	1	0	0	0	0
0	0	0	0	0	0
0	0	0	0	0	0
0	0	0	0	1	0
0	1	0	0	0	0
0	0	0	0	0	0
0	0	0	0	0	0
0	0	0	1	0	0
0	0	0	1	0	0
0	0	0	0	0	0
0	0	0	0	0	0
0	0	0	0	0	0
0	0	0	0	0	0
1	0	0	0	0	0
0	0	0	0	0	0
0	0	0	0	0	0
0	0	0	1	0	0
0	0	0	1	0	0
1	0	0	1	0	0
1	0	0	1	0	0
0	0	0	1	0	0
0	0	0	1	0	0
0	0	0	1	0	0
0	0	0	99	0	0
0	0	0	0	0	0
0	0	0	1	1	0
0	0	0	0	0	0
0	0	0	0	0	0
1	0	0	1	0	0
0	0	0	1	0	0
0	0	0	0	0	0
0	0	0	1	0	0
0	0	0	1	0	0
0	0	0	0	0	0
0	0	0	0	0	0
0	0	0	1	0	0
0	0	0	0	0	0
0	0	0	0	0	0
0	0	0	0	0	0
0	0	0	0	0	0
0	0	0	0	0	0
0	1	0	1	0	0
0	0	0	1	0	0
0	0	1	1	0	0

0	0	0	0	0	0
0	0	0	0	0	0
0	0	0	0	0	0
0	0	0	1	0	0
0	0	0	0	0	0
0	0	0	0	1	0
0	0	1	0	0	0
0	0	0	0	0	0
0	0	0	1	0	0
0	0	0	0	0	0
0	0	0	1	1	0
0	0	0	0	0	0
0	0	0	1	0	0
0	0	0	0	0	0
0	0	0	0	0	0
0	0	1	1	0	0
0	0	0	1	0	0
0	0	0	0	0	0
0	0	0	0	0	0
0	0	1	0	0	0
0	1	0	1	0	0
0	0	0	0	0	0
0	0	0	0	0	0
0	0	0	0	0	0
0	0	0	0	0	0
0	0	0	99	1	0
0	0	0	0	0	0
0	0	0	0	0	0
0	0	0	0	0	0
0	0	0	0	0	0
0	0	0	0	0	0
0	0	0	1	0	0
0	0	0	1	0	0
0	0	0	1	0	0
0	0	0	1	0	0
0	0	0	1	0	0
0	0	0	0	0	0
0	0	0	0	0	0
0	0	1	0	0	0
0	1	0	1	0	0
0	0	0	1	1	0
0	0	0	1	0	0
0	0	0	1	0	0
0	0	0	1	0	0
0	0	0	1	0	0
0	0	0	0	0	0
0	0	0	0	0	0
0	0	0	1	0	0

0	0	0	0	0	0
0	0	0	0	0	0
0	0	0	0	0	0
0	0	1	1	0	0
0	0	0	0	0	0
0	0	0	0	0	0
0	1	0	0	0	0
0	0	0	1	0	0
0	0	0	1	1	0
0	0	0	0	0	0
0	0	1	1	0	0
0	1	0	0	0	0
0	0	0	1	0	0
0	0	0	0	0	0
0	0	0	1	0	0
0	0	0	0	0	0
0	0	0	0	0	0
0	1	0	0	0	0
0	0	0	99	0	0
0	0	0	0	0	0
0	0	0	1	0	0
0	0	0	0	0	0
0	0	0	1	0	0
0	0	0	0	0	0
0	0	0	1	0	0
0	0	0	0	0	0
0	0	0	1	0	0
0	0	0	0	0	0
0	0	0	1	0	0
0	0	0	1	0	0
0	0	0	1	0	0
0	0	0	0	0	0
0	1	0	0	1	0
0	0	0	0	0	0
0	0	0	1	1	1
0	0	1	0	0	0
0	0	1	99	0	0
0	1	0	0	0	0
0	0	0	0	0	0
0	1	0	0	0	0
0	0	0	1	0	0
0	0	0	0	0	0
0	0	0	0	0	0
0	0	0	1	0	0
0	0	0	1	0	0
0	0	0	0	0	0
0	0	0	1	0	0
0	0	0	0	0	0

0	0	1	0	0	0
0	0	0	1	0	0
0	0	0	0	1	0
0	0	0	0	0	0
0	0	0	1	1	0
0	0	0	0	0	0
0	0	0	0	0	0
0	0	0	0	0	0
0	0	0	0	0	0
0	0	0	0	0	0
1	0	0	1	0	0
0	0	0	0	0	0
0	0	0	1	0	0
0	0	0	0	0	0
0	0	0	0	0	0
0	0	0	0	0	0
0	1	0	1	0	0
0	0	1	0	0	0
0	0	1	0	0	0
0	0	0	0	0	0
0	0	0	0	0	0
0	0	0	1	0	0
0	0	0	0	0	0
0	0	0	0	0	0
0	1	0	0	0	0
0	0	0	1	0	0
0	0	0	1	0	0
0	0	0	1	0	0
0	0	0	1	0	0
0	0	0	1	0	0
0	0	0	1	0	0
0	0	0	0	0	0
0	0	0	1	0	0
0	0	0	0	0	0
0	0	0	1	0	0
0	0	0	1	0	0
0	0	0	0	0	0
0	1	0	1	0	0
0	0	0	0	0	0
0	0	0	0	0	0
0	0	0	1	0	0
0	0	0	1	1	1
0	0	0	1	0	0
0	0	0	1	0	0
0	0	0	0	0	0
0	0	0	0	0	0
0	0	0	0	0	0
0	0	0	1	1	0

0	0	0	1	0	0
0	0	0	0	0	0
0	0	0	0	0	0
1	0	0	1	0	0
0	1	0	1	0	0
0	0	1	0	0	0
0	0	0	0	0	0
0	0	0	0	0	0
0	0	0	1	0	0
0	0	1	0	0	0
0	0	0	1	0	0
0	0	1	1	0	0
0	0	0	99	0	0
0	0	0	0	0	0
0	1	0	99	0	0
0	0	0	1	0	0
0	0	0	99	0	0
0	0	1	0	0	0
0	0	0	1	0	0
0	0	0	1	0	0
0	1	0	1	0	0
0	0	0	1	0	0
0	0	0	1	0	0
0	0	0	1	1	0
0	0	0	1	0	0
0	0	0	0	0	0
0	1	0	99	0	0
0	1	0	1	0	0
0	1	0	1	0	0
0	0	0	0	0	0
0	0	1	1	0	0
0	0	0	1	0	0
0	0	0	0	0	0
0	0	0	0	0	0
0	0	0	0	0	0
1	0	0	0	0	0
0	0	0	0	0	0
0	1	0	0	0	0
0	0	1	0	0	0
0	0	0	1	0	0
0	0	0	1	0	0
0	0	1	0	1	0
0	0	0	1	0	0
0	0	0	1	1	0
0	0	0	1	0	0
0	0	0	1	0	0
0	0	0	1	0	0
0	0	0	0	0	0

0	0	0	1	0	0
0	0	0	1	0	0
0	1	0	0	0	0
0	0	0	0	0	0
0	0	0	0	0	0
0	0	1	0	0	0
0	0	0	99	0	0
0	0	0	1	0	0
0	0	0	1	0	0
0	0	0	1	0	0
0	0	0	99	0	0
0	0	0	1	0	0
0	0	0	0	0	0
0	0	0	0	0	0
0	0	0	1	0	0
0	0	0	0	0	0
0	0	0	0	0	0
0	0	0	1	0	0
0	0	0	0	0	0
0	0	0	1	0	0
0	0	0	1	0	0
0	1	0	0	0	0
0	0	1	0	0	0
0	0	0	0	0	0
0	0	0	0	0	0
0	1	0	0	0	0
0	1	0	0	0	0
0	0	0	0	0	0
0	0	0	1	0	0
0	0	0	1	0	0
0	0	0	99	0	0
0	0	0	1	0	0
0	0	0	1	0	0
0	0	0	0	0	0
0	0	0	0	0	0
0	0	0	1	0	0
0	0	1	99	0	0
0	0	0	0	0	0
0	0	0	1	0	0
0	0	0	0	0	0
0	0	0	0	0	0
0	0	0	0	0	0
0	0	0	0	0	0
0	0	0	1	0	0
0	0	0	0	0	0
0	0	0	0	0	0
0	0	0	1	1	0
0	0	0	1	0	0

0	0	0	0	0	0
0	0	0	0	0	0
0	0	0	1	0	0
0	0	0	0	0	0
0	0	0	1	0	0
0	0	1	0	0	0
0	1	0	0	0	0
0	0	0	1	0	0
0	1	0	0	0	0
0	0	0	0	0	0
0	0	0	0	0	0
0	0	0	1	0	0
0	0	0	1	0	0
0	0	0	0	0	0
0	0	0	1	0	0
0	0	0	0	0	0
0	0	0	0	0	0
0	0	0	99	0	0
0	1	0	1	0	0
0	0	0	0	0	0
1	0	0	0	0	0
0	0	0	1	0	0
0	0	0	1	0	0
0	0	0	0	0	0
0	0	0	0	0	0
0	0	0	1	0	0
0	0	0	1	0	0
0	0	0	99	0	0
0	0	0	0	0	0
0	0	0	0	0	0
0	1	0	0	0	0
0	0	0	0	0	0
0	0	0	0	0	0
0	0	0	0	0	0
0	0	0	1	1	0
0	0	0	1	0	0
0	0	0	1	0	0
0	0	1	99	0	0
0	0	1	0	0	0
0	0	0	1	0	0
0	0	0	0	0	0
0	0	0	0	0	0
0	0	0	1	0	0
0	0	1	0	0	0
0	0	0	1	0	0
0	0	0	0	0	0
0	0	0	1	1	0
1	0	0	0	0	0

0	0	1	99	0	0
0	0	1	0	0	0
0	0	0	1	0	0
0	0	0	99	0	0
0	0	0	0	0	0
0	0	0	0	0	0
0	0	1	0	0	0
0	0	0	0	0	0
0	0	0	0	0	0
0	0	0	0	0	0
0	0	0	0	0	0
0	0	0	0	0	0
0	0	0	0	0	0
0	0	0	0	0	0
0	0	0	0	0	0
0	0	0	1	0	0
0	0	0	1	0	0
0	0	0	1	0	0
0	0	0	0	0	0
0	0	0	1	0	0
0	1	0	0	0	0
0	1	0	1	0	0
0	0	0	1	0	0
0	0	0	1	0	0
0	0	0	1	0	0
0	0	0	1	0	0
0	0	0	1	0	0
0	0	0	0	0	0
0	0	0	1	0	0
0	0	0	0	0	0
0	0	0	1	0	0
0	0	0	1	0	0
0	1	0	1	0	0
0	0	0	1	0	0
0	0	0	1	1	0
0	0	0	1	0	0
0	0	0	1	0	0
0	0	0	0	0	0
0	1	0	1	0	0
0	0	0	1	0	1
0	0	1	1	1	0
0	0	0	1	0	0
0	0	0	0	0	0
1	0	0	1	0	0
0	0	0	0	0	0
0	0	1	1	0	0
0	1	0	0	0	0
0	0	0	1	0	0

0	1	0	1	0	0
0	0	0	1	0	0
0	1	0	1	0	0
0	0	0	0	1	0
0	0	0	1	0	0
0	1	0	1	0	0
0	0	0	1	0	0
0	0	0	1	0	0
0	0	0	0	0	0
0	0	0	0	0	0
0	0	0	1	0	0
0	0	0	99	0	0
0	0	0	0	0	0
0	0	0	1	0	0
0	0	0	1	0	0
0	0	0	1	0	0
0	0	0	0	0	0
0	1	0	0	0	0
0	1	0	0	0	0
0	0	0	0	0	0
0	0	0	0	0	0
0	0	0	0	0	0
0	0	0	0	0	0
0	1	0	0	0	0
0	0	0	0	0	0
0	0	0	1	0	0
0	0	0	1	0	0
0	0	0	1	0	0
0	0	0	0	0	0
0	0	0	0	0	0
0	0	0	1	1	0
0	0	0	0	0	0
0	0	0	1	0	0
0	0	0	1	0	0
0	0	0	1	0	0
0	0	0	1	0	0
0	0	1	1	0	0
0	0	0	0	0	0
0	1	0	99	0	0
0	0	1	1	0	0
0	0	0	1	0	0
0	0	0	0	0	0
0	0	0	1	0	0
1	0	0	0	0	0
0	1	0	0	0	0
0	0	0	0	0	0
0	0	0	1	0	0
0	0	0	0	0	0

0	0	1	0	0	0
0	0	0	0	0	0
0	0	0	0	0	0
0	0	0	1	0	0
0	0	0	1	0	0
0	0	0	1	0	0
0	0	0	1	0	0
0	1	0	1	0	0
0	0	0	0	0	0
0	0	0	1	0	0
0	0	0	0	0	0
0	0	0	0	0	0
0	0	0	1	0	0
0	0	0	0	0	0
0	0	0	0	0	0
0	0	0	1	1	0
0	1	0	0	0	0
0	0	0	1	0	0
0	0	0	1	0	0
0	0	0	1	0	0
0	0	0	1	0	0
0	0	0	0	0	0
0	1	0	0	0	0
0	0	0	1	0	0
0	0	0	1	0	0
0	0	0	0	0	0
0	1	0	0	0	0
0	0	0	99	0	1
0	0	1	0	0	0
0	1	0	1	0	0
0	0	0	99	0	0
1	0	0	0	0	0
0	0	0	1	0	0
0	0	0	99	0	0
0	1	0	99	0	0
0	0	1	99	0	0
0	1	0	1	0	0
0	0	0	0	0	0
0	1	0	0	0	0
0	0	0	99	0	0
0	0	0	0	0	0
0	0	0	1	0	0
0	0	0	0	0	0
0	0	0	0	0	0
0	0	0	0	0	0
0	0	0	1	0	0
0	0	0	0	0	0
0	0	0	0	0	0

0	0	1	0	0	0
0	0	0	1	0	0
0	0	0	0	0	0
1	0	0	1	0	0
0	1	0	0	0	0
0	1	0	1	0	0
0	1	0	0	0	0
0	1	0	1	0	0
0	1	0	99	0	0
0	0	0	0	0	0
0	0	0	0	0	0
0	1	0	1	0	0
0	0	0	1	0	0
0	0	0	0	0	0
0	0	0	0	0	0
0	0	0	0	0	0
0	0	0	0	0	0
0	0	0	1	0	0
0	0	0	0	0	0
0	0	0	1	0	0
0	0	0	1	0	0
0	0	0	99	0	0
0	0	0	0	1	0
0	0	0	1	0	0
0	0	0	1	0	0
0	0	0	99	0	0
0	0	0	0	0	0
0	0	0	1	0	0
0	0	0	0	0	0
0	0	0	0	0	0
0	0	0	0	0	0
0	0	0	1	0	0
0	0	0	1	0	0
0	0	0	1	1	0
0	1	0	1	0	0
0	0	0	1	0	0
0	1	0	99	0	0
0	0	0	0	0	0
0	0	0	0	0	0
0	0	0	0	0	0
0	0	0	0	0	0
0	0	0	1	0	0
0	0	0	1	0	0
0	0	0	0	0	0
0	0	0	1	1	0
0	0	0	0	0	0
0	0	0	0	0	0
0	0	0	1	0	0

0	0	0	1	0	0
0	0	0	1	0	0
0	0	0	0	0	0
0	0	0	1	0	0
0	0	0	1	0	0
0	0	0	1	0	0
0	0	0	1	0	0
0	0	0	1	0	0
0	0	1	0	0	0
0	0	0	0	0	0
0	0	1	0	0	0
0	0	0	1	0	0
0	0	0	0	0	0
0	0	0	0	0	0
0	1	0	1	0	0
0	0	0	1	0	0
0	0	0	0	0	0
0	0	0	0	0	0
0	0	1	0	0	0
0	0	0	0	0	0
0	0	0	0	1	0
1	0	0	1	0	0
0	1	0	99	0	0
0	0	0	0	1	0
0	0	0	0	0	0
0	0	0	99	0	0
0	0	0	0	0	0
0	1	0	1	0	0
0	0	1	99	0	0
0	1	0	1	1	0
0	0	1	1	1	0
0	0	0	0	0	0
0	1	0	0	0	0
0	0	0	0	0	0
0	0	0	0	0	0
0	0	0	0	0	0
0	0	0	0	0	0
0	1	0	0	0	0
0	0	0	99	0	0
0	0	0	0	0	0
0	0	0	0	0	0
0	0	0	0	0	0
0	0	0	1	0	0
0	0	0	1	0	0
0	0	0	99	0	0
0	0	0	1	0	0
1	0	0	1	0	0
0	0	0	0	0	0

0	0	0	0	0	0
0	0	1	0	0	0
0	1	0	1	0	0
0	0	0	0	0	0
0	0	0	0	0	0
0	0	1	0	0	0
0	0	0	0	0	0
0	0	0	1	0	0
0	0	0	0	0	0
0	0	0	0	0	0
0	0	0	1	0	0
0	0	0	1	0	0
0	0	1	0	0	0
0	0	0	0	0	0
0	0	0	1	0	0
0	0	0	1	0	0
0	0	0	0	0	0
0	0	1	0	0	0
0	1	0	0	0	0
0	0	0	1	0	0
0	0	0	1	1	0
0	0	0	0	0	0
0	0	0	99	0	0
0	0	0	1	0	0
0	0	0	1	0	0
0	0	1	1	0	0
0	0	1	0	0	0
0	0	0	0	0	0
0	0	0	1	1	0
0	0	0	0	0	0
0	0	0	0	0	0
0	0	0	0	0	0
1	0	0	0	0	0
0	0	0	1	0	0
0	0	0	1	0	0
0	0	0	0	0	0
0	0	0	0	0	0
0	0	1	0	0	0
0	0	0	0	0	0
0	0	0	0	0	0
0	0	0	0	0	0
0	0	0	0	0	0
0	0	0	0	0	0
0	0	0	0	0	0
0	0	0	0	0	0
0	1	0	1	0	0
0	0	0	99	0	0
0	0	0	0	0	0

0	0	0	1	0	0
0	0	0	0	0	0
0	0	0	1	0	0
0	0	0	1	0	0
0	0	0	0	0	0
0	0	0	0	0	0
0	0	1	0	0	0
0	0	0	1	0	0
0	0	0	1	0	0
0	0	0	1	0	0
0	0	0	1	0	0
0	1	0	1	0	0
0	0	0	1	0	0
0	1	0	0	0	0
0	0	0	0	0	0
0	1	0	1	0	0
0	0	0	0	0	0
0	0	0	0	0	0
0	0	1	99	0	0
0	0	0	0	0	0
0	1	0	0	0	0
0	1	0	99	0	0
0	0	0	0	0	0
0	0	1	0	0	0
0	0	0	1	0	0
0	0	0	0	0	0
0	0	0	1	1	0
0	0	0	1	1	0
0	0	0	0	0	0
0	0	0	0	1	0
0	0	0	1	0	0
0	1	0	0	0	0
0	1	0	0	0	0
0	0	0	1	1	0
0	0	0	1	1	0
0	0	0	1	0	0
0	0	0	1	0	1
0	0	0	0	0	0
0	0	0	0	0	0
0	0	0	1	0	0
0	0	0	0	1	0
0	0	0	1	0	0
0	1	0	0	0	0
0	0	0	0	0	0
0	1	0	0	1	0
0	0	0	0	0	0
0	0	1	0	0	0
0	0	0	0	0	0

0	0	0	99	0	0
0	0	0	0	0	0
0	0	0	1	0	0
0	1	0	0	0	0
0	0	0	0	0	0
0	0	0	0	0	0
0	0	0	0	0	0
1	0	0	1	0	0
0	0	0	1	0	0
0	0	0	1	0	0
0	0	0	0	0	0
0	0	0	0	0	0
0	0	0	0	0	0
0	0	0	0	0	0
0	0	0	1	0	0
0	0	0	1	1	0
0	0	0	1	1	0
0	0	0	0	0	0
0	0	0	0	1	0
0	0	0	0	0	0
0	0	0	0	0	0
0	0	0	0	0	0
0	0	0	0	0	0
0	0	0	0	0	0
0	0	0	1	0	0
0	0	0	0	0	0
0	0	0	0	0	0
0	0	0	1	1	0
0	0	0	1	0	0
0	0	0	0	0	0
0	0	0	1	0	0
0	0	0	1	0	0
0	0	0	0	0	0
0	0	0	1	0	0
0	0	0	1	0	0
0	0	0	0	0	0
0	0	1	0	0	0
0	0	0	1	0	0
0	0	0	1	0	0
1	0	0	1	0	0
0	1	0	0	0	0
0	0	0	1	0	0
0	0	0	1	0	0
0	0	0	0	0	0
0	0	0	1	0	0
0	0	0	1	0	0
0	0	0	0	0	0
0	0	0	0	0	0

0	0	0	1	0	0
0	0	0	0	1	0
0	0	1	99	0	0
0	0	0	1	1	0
0	0	0	0	0	0
0	1	0	0	0	0
0	0	0	1	0	0
0	0	0	0	1	0
0	0	0	1	0	0
0	0	0	0	0	0
0	1	0	99	0	0
0	1	0	1	1	0
0	0	0	1	0	0
0	1	0	0	1	0
0	0	0	0	0	0
0	0	0	1	0	1
0	0	0	1	0	0
0	0	0	0	0	0
0	0	0	1	0	0
0	0	0	1	0	0
0	0	0	1	0	0
0	0	0	0	0	0
0	0	0	1	0	0
0	0	0	0	0	0
0	0	0	0	0	0
0	0	0	1	0	0
1	0	0	0	0	0
0	1	0	1	0	0
0	0	0	0	0	0
0	0	0	1	1	0
0	0	0	1	0	0
0	0	0	0	0	0
0	0	0	1	0	0
0	1	0	0	0	0
0	0	0	1	0	0
0	0	0	0	0	0
0	0	0	0	0	0
0	0	0	1	0	0
1	0	0	1	0	0
0	0	0	1	1	0
0	0	0	1	0	0
0	0	0	1	0	0
0	0	0	1	0	0
0	0	0	0	0	0
0	0	0	1	0	0
0	0	0	99	0	0
0	0	0	0	0	0
0	0	0	0	0	0

0	0	0	1	0	0
0	0	0	0	0	0
0	0	0	1	0	0
0	0	0	1	0	0
0	0	0	0	0	0
0	0	0	0	0	0
0	0	0	1	0	0
0	0	0	1	0	0
0	0	0	1	0	0
0	0	0	1	0	0
0	0	0	0	0	0
0	0	0	1	0	0
0	0	0	1	0	0
0	0	0	1	0	0
1	0	0	0	0	0
0	0	0	0	0	0
0	0	0	1	0	0
0	0	0	0	0	0
0	0	0	1	0	0
0	0	0	0	0	0
0	0	0	0	0	0
0	0	0	0	0	0
0	0	0	0	0	0
0	0	0	1	0	0
0	0	0	0	0	0
0	0	0	1	1	0
0	0	0	1	0	0
0	0	0	1	1	0
0	0	0	0	1	0
0	0	0	1	0	0
0	0	0	0	0	0
0	0	0	0	0	0
0	0	0	1	0	0
0	1	0	1	0	0
0	0	0	1	0	0
0	0	1	0	0	0
1	0	0	1	1	0
0	0	0	1	0	0
0	0	0	0	0	0
0	0	0	1	0	0
0	0	0	1	1	0
0	0	0	0	0	0
0	0	0	0	0	0
0	0	0	1	0	0
0	1	0	0	0	0
0	0	0	0	0	0
0	0	0	1	1	0
0	1	0	1	0	0

0	0	0	1	0	0
0	0	0	0	0	0
0	0	0	0	0	0
0	0	0	1	0	0
0	0	0	1	0	0
0	0	0	1	0	0
1	0	0	0	0	0
0	0	0	0	0	0
0	0	0	0	0	0
0	0	0	0	0	0
0	1	0	1	0	0
0	0	0	1	0	0
0	0	0	1	0	0
0	0	0	0	0	0
0	0	0	1	0	0
0	0	0	0	0	0
0	0	0	1	0	0
0	1	0	0	0	0
0	0	1	0	0	0
0	0	0	0	0	0
0	0	0	0	0	0
0	0	0	1	0	0
0	1	0	0	0	0
0	1	0	0	0	0
0	1	0	0	0	0
0	0	0	0	0	0
0	0	0	0	0	0
0	0	0	0	0	0
0	0	0	0	0	0
0	0	0	0	0	0
0	0	0	1	0	0
0	0	0	1	0	0
0	0	0	1	0	0
0	0	0	1	0	0
0	0	0	0	0	0
0	0	0	1	0	0
0	0	0	0	0	0
0	0	0	99	0	0
0	0	0	1	0	0
0	0	0	1	0	0
0	0	0	1	0	0
0	0	0	99	0	0
0	0	0	0	0	0
0	0	0	1	0	0
0	0	0	1	0	0
0	0	0	0	0	0
0	0	0	1	0	0
0	0	0	1	0	0

0	0	0	1	0	0
0	0	0	0	0	0
0	0	0	0	0	0
0	0	#NULL!	1	1	0
0	0	#NULL!	0	0	0
1	0	#NULL!	0	0	0
0	0	#NULL!	1	0	0
0	0	#NULL!	0	0	0
0	0	#NULL!	0	0	0
0	0	#NULL!	0	0	0
0	1	#NULL!	1	0	0
0	0	#NULL!	0	0	0
0	0	#NULL!	0	0	0
0	0	#NULL!	0	0	0
0	0	#NULL!	0	0	0
0	0	#NULL!	0	0	0
0	0	#NULL!	1	0	0
0	0	#NULL!	1	0	0
0	0	#NULL!	0	0	0
0	0	#NULL!	1	1	0
0	0	#NULL!	1	0	0
0	0	#NULL!	0	0	0
0	0	#NULL!	0	0	0
0	0	#NULL!	0	0	0
0	0	#NULL!	1	0	0
0	0	#NULL!	1	0	0
0	0	#NULL!	0	1	0
0	0	#NULL!	1	0	0
0	0	#NULL!	0	0	0
0	0	#NULL!	1	1	0
0	0	#NULL!	1	0	0
0	0	#NULL!	1	0	0
1	0	#NULL!	1	0	0
0	0	#NULL!	0	0	0
0	0	#NULL!	0	0	0
0	0	#NULL!	0	0	0
0	0	#NULL!	1	0	0
0	0	#NULL!	0	0	0
0	0	#NULL!	1	0	0
0	0	#NULL!	1	0	0
0	0	#NULL!	0	0	0
0	0	#NULL!	1	0	0
0	0	#NULL!	0	0	0
0	0	#NULL!	1	0	0
0	0	#NULL!	0	1	0
0	0	#NULL!	1	0	0
0	0	#NULL!	0	0	0
0	0	#NULL!	1	0	0

0	0	#NULL!	1	0	0
0	0	#NULL!	0	0	0
0	1	#NULL!	0	0	0
0	1	#NULL!	0	0	0
0	0	#NULL!	0	1	0
0	0	#NULL!	0	1	0
0	0	#NULL!	0	0	0
0	0	#NULL!	0	0	0
0	1	#NULL!	0	0	0
0	0	#NULL!	1	0	0
0	1	#NULL!	0	0	0
0	0	#NULL!	1	0	0
0	0	#NULL!	1	0	0
0	1	#NULL!	0	0	0
0	0	#NULL!	0	0	0
0	0	#NULL!	1	0	0
0	0	#NULL!	1	0	0
0	0	#NULL!	1	0	0
0	0	#NULL!	1	0	0
0	0	#NULL!	1	0	0
0	0	#NULL!	1	0	0
0	0	#NULL!	1	0	0
0	0	#NULL!	1	0	0
0	0	#NULL!	1	0	0
0	1	#NULL!	1	0	0
0	1	#NULL!	1	0	0
0	0	#NULL!	0	0	0
0	1	#NULL!	0	0	0
0	0	#NULL!	1	0	0
0	1	#NULL!	1	0	0
0	0	#NULL!	1	0	0
0	0	#NULL!	0	0	0
0	1	#NULL!	0	0	0
0	1	#NULL!	1	0	0
0	0	#NULL!	0	0	0
0	0	#NULL!	1	0	0
0	0	#NULL!	0	0	0
0	0	#NULL!	1	0	0
0	0	#NULL!	1	0	0
0	1	#NULL!	0	0	0
0	0	#NULL!	1	0	0
0	0	#NULL!	1	0	0
0	0	#NULL!	1	0	0
0	0	#NULL!	0	0	0
0	0	#NULL!	0	0	0
0	0	#NULL!	0	0	0
0	0	#NULL!	1	0	0
0	0	#NULL!	1	0	0

0	0	#NULL!	0	0	0
0	0	#NULL!	1	0	0
0	0	#NULL!	0	0	0
0	0	#NULL!	1	0	0
0	0	#NULL!	1	0	0
0	0	#NULL!	1	0	0
0	1	#NULL!	1	0	0
0	0	#NULL!	1	0	0
0	0	#NULL!	1	0	0
0	0	#NULL!	0	0	0
0	0	#NULL!	0	0	0
0	0	#NULL!	#NULL!	0	0
0	0	#NULL!	1	0	0
0	0	#NULL!	1	0	0
0	0	#NULL!	1	0	0
0	0	#NULL!	1	0	0
0	0	#NULL!	0	0	0
0	0	#NULL!	0	0	0
0	0	#NULL!	0	1	0
0	0	#NULL!	1	0	0
0	0	#NULL!	1	0	0

race_pre___3	race_pre___4	race_pre___5	race_pre___6	race_pre___99	race_other_pre
0	0	0	1	0	
0	0	0	0	0	
0	1	0	0	0	
0	1	0	0	0	
0	1	0	0	0	
0	0	0	0	1	
0	0	0	1	0	
0	0	0	1	0	
0	0	0	0	1	
0	1	1	0	0	
0	1	0	0	0	
0	1	0	0	0	
0	1	0	0	0	
0	0	0	1	0	
0	1	0	0	0	
0	0	0	1	0	
0	0	0	1	0	
0	0	0	0	1	
0	0	0	1	0	
0	1	0	0	0	
0	0	0	0	1	
0	0	0	1	0	
0	0	0	0	1	
0	0	0	1	0	
0	0	0	0	1	
0	0	0	0	1	
0	0	0	0	1	
0	1	0	1	0	
0	0	0	1	0	
0	0	0	1	0	
0	0	0	1	0	
0	0	1	0	0	
0	0	0	1	0	
0	0	0	0	1	
0	0	0	0	1	
0	0	0	0	1	
0	0	0	1	0	
0	0	0	0	1	
0	0	0	1	0	
0	1	0	1	0	
0	0	0	1	0	
0	1	0	0	0	
0	1	0	1	0	
0	1	0	0	0	
0	1	0	0	0	
0	1	0	0	0	
0	1	0	0	0	

0	0	0	1	0
0	1	0	0	0
0	0	0	1	0
0	1	0	0	0
0	0	0	0	0
0	1	0	0	0
0	1	0	0	0
0	0	0	1	0
0	0	0	1	0
0	1	0	0	0
0	1	0	0	0
0	1	0	0	0
0	0	0	1	0
0	1	0	0	0
0	0	1	0	0
0	1	0	0	0
0	1	0	0	0
0	0	0	1	0
0	1	0	0	0
0	1	0	0	0
0	0	0	1	0
0	0	0	1	0
0	1	0	0	0
0	1	0	0	0
0	1	0	0	0
0	0	0	1	0
0	1	0	0	0
0	0	0	1	0
0	0	0	1	0
0	0	0	1	0
0	1	0	0	0
0	1	0	0	0
0	1	0	0	0
1	1	0	0	0
0	0	0	1	0
0	0	0	1	0
0	0	0	0	1
0	0	0	1	0
0	0	0	1	0
0	1	0	0	0
0	0	0	0	1
0	0	0	1	0
0	0	0	1	0
0	0	0	1	0
0	0	0	1	0
0	1	0	0	0
0	1	0	0	0
0	0	0	0	1

0	1	0	0	0
0	0	0	1	0
0	0	0	1	0
1	0	0	0	0
0	0	0	0	1
0	1	0	0	0
0	1	0	1	0
0	1	0	0	0
0	0	0	1	0
0	1	0	0	0
0	0	0	1	0
0	1	0	0	0
0	1	0	1	0
0	1	0	0	0
0	1	0	0	0
0	0	0	0	1
0	1	0	0	0
0	0	0	1	0
0	0	0	0	1
0	0	0	1	0
0	0	0	1	0
0	1	0	0	0
0	0	0	0	1
0	1	0	0	0
0	0	0	1	0
0	0	1	0	0
0	0	0	1	0
0	1	0	0	0
0	0	0	1	0
0	0	0	1	0
0	0	0	1	0
0	0	1	0	0
0	0	0	1	0
0	1	0	0	0
0	0	0	1	0
0	0	0	1	0
0	0	0	1	0
0	0	0	0	0
1	0	0	0	0
0	1	0	0	0
0	1	0	0	0
0	1	0	0	0
0	0	0	0	0
0	1	0	0	0
0	1	0	0	0
0	1	0	0	0
0	1	0	0	0
0	0	0	0	1

0	0	0	1	0
0	0	0	1	0
0	1	0	0	0
0	1	0	0	0
0	0	0	1	0
0	1	0	0	0
0	0	0	0	0
0	0	1	0	0
0	0	0	1	0
0	0	0	1	0
0	0	0	0	1
0	0	0	0	1
0	1	0	0	0
0	1	0	0	0
0	0	0	1	0
0	0	0	1	0
0	1	0	1	0
0	0	0	0	0
0	0	0	1	0
0	0	0	1	0
0	0	0	1	0
0	0	0	1	0
0	1	0	0	0
0	1	0	0	0
0	0	0	1	0
0	1	0	0	0
0	1	0	0	0
0	1	0	0	0
0	0	0	1	0
0	1	0	0	0
0	0	0	0	0
0	0	0	0	1
0	0	0	1	0
0	0	0	1	0
0	1	0	0	0
0	1	0	0	0
0	1	0	0	0
0	0	0	1	0
0	1	0	0	0
0	1	0	0	0
0	0	0	1	0
0	0	0	1	0
0	1	0	0	0
0	1	0	0	0
0	1	0	0	0
0	1	0	0	0
0	1	0	0	0
0	1	0	0	0

0	0	0	0	1
0	1	0	0	0
0	1	0	0	0
0	0	0	1	0
0	1	0	0	0
0	1	0	0	0
0	1	0	1	0
0	1	0	0	0
0	0	0	1	0
0	1	0	0	0
0	1	0	0	0
0	0	0	0	0
0	1	0	0	0
0	1	0	0	0
0	0	0	1	0
0	1	0	0	0
0	1	0	0	0
0	0	0	1	0
0	1	0	0	0
0	0	0	1	0
0	0	0	0	0
0	0	0	1	0
0	0	0	1	0
0	0	0	0	0
0	1	0	0	0
0	0	0	1	0
0	0	1	0	0
0	1	0	0	0
0	0	0	0	0
0	0	0	0	1
0	0	0	1	0
0	0	0	1	0
0	0	0	1	0
0	0	0	1	0
0	1	0	0	0
0	1	0	0	0
0	0	0	1	0
0	0	0	1	0
0	0	0	1	0
0	0	0	1	0
0	1	0	0	0
0	1	0	0	0
0	1	0	0	0
0	0	0	1	0
0	0	0	0	0
0	1	0	0	0
0	1	0	0	0
0	0	0	0	1

0	1	0	0	0
0	0	0	1	0
0	1	0	0	0
0	0	0	1	0
0	1	0	0	0
0	0	0	1	0
0	1	0	0	0
0	0	0	0	1
0	1	0	0	0
0	0	0	1	0
0	1	0	0	0
0	0	0	0	1
0	0	0	1	0
0	0	0	1	0
0	0	0	1	0
0	0	0	0	0
0	0	0	1	0
0	1	0	0	0
0	0	0	0	1
0	1	0	0	0
0	0	0	1	0
0	0	0	0	0
0	0	0	1	0
0	0	0	0	1
0	0	0	0	1
0	1	0	0	0
0	1	0	0	0
0	0	0	1	0
0	1	0	0	0
0	0	0	1	0
0	0	0	1	0
0	0	0	1	0
0	0	0	1	0
0	0	0	1	0
0	0	0	1	0
0	0	1	0	0
0	0	0	1	0
0	1	0	0	0
0	1	0	0	0
0	0	0	1	0
0	0	0	1	0
0	0	0	1	0
0	0	0	1	0
0	0	1	0	0
0	0	0	1	0
0	0	0	0	0
0	1	0	0	0
0	1	0	1	0

0	0	0	1	0
0	1	0	0	0
0	1	0	0	0
0	0	0	0	1
0	1	0	0	0
0	0	0	0	0
0	1	0	0	0
0	1	0	0	0
0	0	0	1	0
0	0	0	1	0
0	1	0	0	0
0	0	0	1	0
0	0	0	0	1
1	1	0	0	0
0	1	0	0	0
0	1	0	0	0
0	0	0	1	0
0	0	0	1	0
0	0	0	1	0
0	1	0	0	0
0	1	0	0	0
0	1	0	0	0
0	0	0	1	0
0	1	0	1	0
0	0	0	1	0
0	0	0	0	1
0	1	0	0	0
0	1	0	1	0
0	1	0	0	0
0	0	0	0	1
0	0	1	0	0
0	0	0	0	1
0	1	0	0	0
0	0	0	0	1
0	0	0	0	0
0	1	0	0	0
0	0	0	0	1
0	0	0	1	0
0	0	0	1	0
0	0	0	1	0
0	0	0	0	1
0	0	0	1	0
0	0	0	1	0
0	0	0	1	0
0	1	0	0	0
0	0	0	1	0
0	0	0	1	0
0	0	0	0	1

0	0	0	1	0
0	0	0	1	0
0	0	0	1	0
0	1	0	0	0
0	0	0	1	0
0	0	0	0	1
0	0	0	1	0
0	1	0	0	0
0	1	0	0	0
0	0	0	0	1
0	0	1	0	0
0	0	0	1	0
0	1	0	0	0
0	0	0	0	0
0	0	0	1	0
0	0	0	1	0
0	0	0	1	0
0	0	0	0	0
0	0	0	1	0
0	0	0	1	0
0	0	0	0	1
0	0	0	1	0
0	1	0	0	0
0	0	1	0	0
0	0	0	1	0
0	1	0	0	0
0	1	0	0	0
0	0	0	0	1
0	1	0	1	0
0	1	0	0	0
0	1	0	0	0
0	1	0	0	0
0	0	0	1	0
0	1	0	0	0
0	1	0	0	0
0	0	0	1	0
0	1	0	0	0
0	1	0	0	0
0	0	0	0	1
0	1	0	0	0
0	1	0	0	0
0	0	0	1	0
0	1	0	0	0
0	0	0	1	0
0	0	0	0	0
0	1	0	0	0
0	1	0	0	0
0	0	0	1	0

0	1	0	0	0
0	1	0	0	0
0	0	0	1	0
0	0	0	1	0
0	1	0	0	0
0	1	0	0	0
0	1	0	0	0
0	1	0	0	0
0	0	0	1	0
0	1	0	0	0
0	1	0	0	0
0	1	0	0	0
0	0	0	0	1
0	0	0	1	0
0	1	0	0	0
0	1	0	0	0
0	1	0	0	0
0	1	0	0	0
0	0	0	1	0
0	1	0	0	0
0	0	0	1	0
0	1	0	0	0
0	0	0	1	0
0	0	0	0	0
0	0	0	1	0
0	1	0	0	0
0	0	0	1	0
0	1	0	0	0
0	0	0	1	0
0	0	0	1	0
0	0	0	0	1
0	0	0	1	0
0	1	0	0	0
0	1	0	0	0
0	0	0	1	0
0	0	0	0	1
0	1	0	0	0
0	1	0	0	0
0	0	0	1	0
0	0	0	1	0
0	1	0	0	0
0	1	0	1	0
0	0	0	1	0
0	1	0	0	0
0	1	0	0	0
0	1	0	1	0
0	1	0	0	0
0	0	1	0	0

0	0	0	1	0
0	1	0	0	0
0	0	0	1	0
0	0	0	1	0
0	0	0	0	1
0	0	0	1	0
0	0	0	0	0
0	0	0	1	0
0	1	0	0	0
0	0	0	0	1
0	1	0	0	0
0	0	0	0	0
0	1	0	0	0
0	0	0	0	1
0	0	0	1	0
0	1	0	0	0
0	0	0	1	0
0	1	0	0	0
0	1	0	0	0
0	0	0	0	1
0	1	0	0	0
0	0	0	1	0
0	1	0	0	0
0	0	0	0	1
0	0	0	1	0
0	0	0	0	0
0	1	0	0	0
0	1	0	0	0
0	0	0	0	0
0	0	0	1	0
0	0	0	1	0
0	0	0	0	0
0	0	1	0	0
0	1	0	0	0
0	1	0	0	0
0	0	0	1	0
0	1	0	1	0
0	0	0	1	0
0	1	0	0	0
0	1	0	0	0
0	0	0	0	1
0	0	0	0	0
0	0	0	1	0
0	0	0	0	0
0	1	0	0	0
0	0	0	0	0
0	0	0	0	1
0	0	0	1	0

0	0	0	0	1
0	0	0	1	0
0	0	0	1	0
0	0	0	1	0
0	1	0	0	0
0	1	0	0	0
0	1	0	0	0
0	0	1	0	0
0	1	0	0	0
0	1	0	0	0
0	0	1	0	0
0	0	1	0	0
0	0	0	1	0
0	1	0	0	0
0	0	0	0	1
0	0	0	1	0
0	0	0	1	0
0	0	0	0	1
0	0	0	1	0
0	0	0	1	0
0	0	0	1	0
0	0	0	1	0
0	0	0	0	0
0	0	0	1	0
0	0	0	0	1
0	1	0	0	0
0	1	1	1	0
0	0	0	1	0
0	1	0	0	0
0	0	0	1	0
0	0	0	0	0
0	1	0	0	0
0	1	0	0	0
0	0	0	0	0
0	0	0	1	0
0	0	0	1	0
0	0	0	1	0
0	0	0	1	0
0	0	0	1	0
0	0	0	0	1
0	0	0	0	0
0	0	0	1	0
0	1	0	0	0
0	0	0	1	0
0	1	1	0	0
0	1	0	0	0
0	0	0	0	1
0	0	0	1	0

0	0	0	1	0
0	0	0	1	0
0	0	0	1	0
0	1	0	0	0
0	1	0	0	0
0	1	0	0	0
0	1	0	0	0
0	0	0	1	0
0	1	1	0	0
0	1	0	0	0
0	0	0	1	0
0	0	0	1	0
0	1	0	0	0
0	1	0	0	0
0	0	0	1	0
0	0	0	1	0
0	0	0	0	1
0	1	0	0	0
0	0	0	1	0
0	0	0	1	0
0	1	0	0	0
0	0	0	1	0
0	1	0	0	0
0	1	0	0	0
0	1	1	0	0
0	0	0	1	0
0	0	0	0	0
0	0	0	0	1
0	0	0	0	0
0	1	0	0	0
0	0	0	1	0
0	0	0	0	1
0	0	1	0	0
0	1	0	0	0
0	0	0	0	1
0	0	0	1	0
0	0	0	0	1
0	1	0	0	0
0	0	0	0	1
0	0	0	1	0
0	1	0	0	0
0	1	0	0	0
0	0	0	1	0
0	1	0	0	0
0	1	0	0	0
0	0	0	1	0
0	0	0	0	1
0	0	0	1	0

0	0	0	0	1
0	1	0	0	0
0	0	0	1	0
0	0	0	1	0
0	0	0	1	0
0	0	0	0	1
0	0	0	1	0
0	0	0	0	1
0	0	0	0	1
0	0	0	1	0
0	1	0	0	0
0	1	0	0	0
0	0	0	1	0
0	1	0	0	0
0	1	0	0	0
0	1	0	0	0
0	1	0	0	0
0	1	0	0	0
0	1	0	0	0
0	1	0	0	0
0	0	0	0	0
0	0	0	1	0
0	0	0	0	1
0	1	0	0	0
0	0	0	0	0
0	0	0	0	1
0	0	0	1	0
0	0	0	0	1
0	1	0	0	0
0	1	0	0	0
0	0	0	0	1
0	1	0	0	0
0	0	0	0	1
0	0	0	0	1
0	0	0	0	1
0	0	0	1	0
0	0	0	0	0
0	1	0	0	0
0	0	0	0	0
0	1	0	0	0
0	0	0	1	0
0	0	1	0	0
0	0	1	0	0
0	1	0	0	0
0	0	0	1	0
0	1	0	0	0
0	1	0	0	0
0	0	0	1	0

0	0	0	1	0
0	0	0	1	0
0	0	0	1	0
0	0	0	1	0
0	1	0	0	0
0	0	0	1	0
0	0	0	1	0
0	1	0	0	0
0	1	0	0	0
0	1	0	0	0
0	1	0	0	0
1	0	0	0	0
0	1	0	0	0
0	1	0	0	0
0	0	1	0	0
0	1	0	0	0
0	1	0	0	0
0	0	0	0	0
0	1	0	1	0
0	1	0	0	0
0	1	0	0	0
0	0	0	1	0
0	0	0	0	1
0	0	0	1	0
0	0	0	1	0
0	0	0	1	0
0	1	0	0	0
0	0	0	0	1
0	1	0	1	0
0	1	0	0	0
0	0	0	0	1
0	1	0	0	0
0	0	0	0	0
0	0	0	1	0
0	0	0	1	0
0	0	0	0	1
0	1	0	0	0
0	1	0	0	0
1	0	0	0	0
0	1	0	0	0
0	1	0	0	0
0	1	0	0	0
0	1	0	0	0
0	1	0	0	0
0	0	0	0	1
0	0	0	1	0
0	0	0	1	0
0	1	0	0	0

0	0	0	1	0
0	0	0	1	0
0	0	0	1	0
0	0	0	1	0
0	1	0	0	0
0	0	0	0	0
0	1	0	0	0
0	1	0	0	0
0	0	0	0	1
0	1	0	0	0
0	1	0	0	0
0	0	0	1	0
0	0	0	1	0
0	0	0	0	0
0	0	0	0	1
0	1	0	0	0
0	0	0	0	1
0	1	0	0	0
0	1	0	0	0
0	1	0	0	0
0	0	0	0	1
0	1	0	1	0
0	1	0	0	0
0	1	0	0	0
0	0	0	0	0
0	0	0	0	1
0	0	0	0	1
0	0	1	0	0
0	0	0	1	0
0	1	0	0	0
0	0	0	0	1
0	0	0	0	1
0	0	0	0	1
0	1	0	0	0
0	0	0	1	0
0	1	0	0	0
0	0	0	1	0
0	0	0	0	1
0	1	0	0	0
0	0	0	0	1
0	1	0	0	0
0	1	0	0	0
0	0	0	0	1
0	0	0	1	0
0	0	0	0	1
0	1	0	0	0
0	0	0	0	1
0	1	0	0	0

0	1	0	0	0
0	0	0	1	0
0	1	0	0	0
0	0	0	0	1
0	0	0	0	1
0	1	0	0	0
0	1	0	0	0
0	0	1	0	0
0	0	0	1	0
0	0	0	1	0
0	0	0	0	1
0	1	0	0	0
0	0	0	1	0
0	1	0	0	0
0	0	0	0	1
0	1	0	0	0
0	1	0	0	0
0	0	0	0	1
0	1	0	0	0
0	0	0	0	1
0	1	0	0	0
0	0	0	0	1
0	0	0	0	1
0	0	1	0	0
0	0	0	0	1
0	1	0	0	0
0	0	0	0	1
0	1	0	0	0
0	0	0	0	1
0	1	0	0	0
0	0	0	0	1
0	0	0	0	1
0	0	0	0	1
0	0	0	0	1
0	1	0	0	0
0	0	0	0	1
0	0	0	0	1
0	0	0	0	1
0	0	0	0	1
0	0	0	0	1
0	0	0	0	1
0	0	0	0	1
0	0	0	0	0
0	0	0	0	1
0	0	0	0	1
0	0	0	1	0
0	0	0	0	1
0	0	0	1	0

0	0	0	1	0
0	0	0	0	1
0	1	0	0	0
0	0	0	0	1
0	1	0	0	0
0	0	0	0	1
0	0	0	0	1
0	1	0	0	0
0	1	0	0	0
0	0	0	0	1
0	1	0	0	0
0	1	0	0	0
0	0	0	1	0
0	0	0	1	0
0	1	0	0	0
0	1	0	0	0
0	0	0	1	0
0	0	0	1	0
0	1	0	0	0
0	0	0	0	1
0	0	0	0	1
0	1	0	0	0
0	0	0	0	1
0	0	0	0	1
0	1	0	0	0
0	1	1	0	0
0	1	0	0	0
0	0	0	1	0
0	0	0	0	0
0	0	0	0	1
1	0	0	0	0
0	0	0	1	0
0	1	0	0	0
0	0	0	1	0
0	1	0	0	0
0	0	0	1	0
0	0	0	1	0
0	0	0	1	0
0	0	1	0	0
0	0	1	0	0
0	1	0	0	0
0	0	0	1	0
0	0	0	0	1
0	0	0	0	0
0	1	1	0	0
0	0	0	0	0
0	0	0	0	0
0	0	0	1	0

0	0	0	1	0
0	1	0	0	0
0	1	0	0	0
0	1	0	0	0
0	1	0	0	0
0	0	0	1	0
0	0	0	0	1
0	0	0	1	0
0	0	0	0	0
0	0	0	1	0
0	1	0	0	0
0	1	0	1	0
0	1	0	1	0
0	1	0	0	0
0	0	0	0	1
0	0	0	1	0
0	0	0	0	0
0	0	0	1	0
0	0	0	1	0
0	0	0	0	1
0	0	0	0	1
0	1	0	0	0
0	1	0	0	0
0	0	0	0	1
0	0	0	0	1
0	0	0	0	1
0	0	0	0	1
0	0	0	0	1
0	1	0	0	0
0	1	0	0	0
0	0	0	0	1
0	0	0	0	1
0	0	0	0	1
0	1	0	1	0
0	1	0	0	0
0	1	0	0	0
0	0	0	0	1
0	0	0	1	0
0	1	0	0	0
0	1	0	0	0
0	0	0	0	1
0	0	0	0	1
0	1	0	0	0
0	0	0	0	1
0	0	0	0	1
0	0	0	1	0
0	0	0	0	1
0	0	0	1	0

0	1	0	0	0
0	0	0	1	0
0	0	0	0	1
0	1	0	0	0
0	1	0	0	0
0	0	0	0	1
0	0	0	0	1
0	0	0	1	0
0	1	0	0	0
0	0	0	0	1
0	1	0	0	0
0	0	0	0	0
0	1	0	0	0
0	1	0	0	0
0	1	1	0	0
0	0	0	0	0
0	0	0	0	1
0	0	0	1	0
0	1	0	0	0
0	0	0	0	1
0	0	0	0	1
0	0	0	0	0
0	0	0	0	1
0	0	0	1	0
0	0	0	0	0
0	1	0	0	0
0	1	0	0	0
0	1	0	0	0
0	0	0	0	1
0	1	0	0	0
0	1	0	0	0
0	0	0	1	0
0	1	0	0	0
0	0	0	0	1
0	1	0	0	0
0	1	0	0	0
0	0	0	0	0
0	0	0	0	1
0	1	0	0	0
0	1	0	0	0
0	0	1	0	0
0	0	0	0	1
0	1	0	0	0
0	0	0	0	1
0	0	0	0	1
1	1	1	1	0
0	0	0	1	0
0	0	0	0	1

0	0	0	0	0
0	0	0	0	0
0	0	0	1	0
0	1	0	0	0
0	0	1	0	0
0	1	0	0	0
0	1	0	0	0
0	1	0	0	0
0	0	0	1	0
0	1	0	0	0
0	0	0	0	1
0	1	0	0	0
0	0	0	0	1
0	0	1	0	0
0	0	0	0	1
0	0	0	1	0
0	0	0	0	1
0	1	0	0	0
0	0	0	1	0
0	0	0	1	0
0	1	0	0	0
0	0	0	1	0
0	1	0	0	0
0	1	0	0	0
0	1	0	0	0
0	1	0	0	0
0	0	0	0	1
0	0	0	0	0
0	1	0	0	0
0	0	0	0	1
0	0	0	0	1
0	0	1	0	0
0	0	0	0	1
0	0	0	1	0
0	0	0	1	0
0	0	0	1	0
0	1	0	0	0
0	1	0	0	0
0	0	0	0	1
0	0	0	1	0
0	1	0	0	0
0	0	0	0	1
0	1	0	0	0
0	0	0	0	1
0	0	0	0	1
0	0	0	0	1
0	0	0	0	1
0	0	0	0	1

0	1	0	0	0
0	0	0	0	1
0	1	0	0	0
0	1	0	0	0
0	0	0	0	1
0	0	0	0	1
0	0	0	0	1
0	0	0	0	1
0	0	0	1	0
0	0	0	1	0
0	0	0	0	0
0	1	0	0	0
0	1	0	0	0
0	1	0	0	0
0	1	0	0	0
0	1	0	0	0
0	1	0	1	0
0	1	0	0	0
0	0	0	0	0
0	0	0	0	1
0	1	0	0	0
0	1	0	0	0
0	0	0	0	1
0	1	0	1	0
0	0	0	0	1
0	0	0	0	1
0	1	0	0	0
0	0	0	1	0
0	1	0	0	0
0	1	0	0	0
0	0	0	0	1
0	1	0	0	0
0	0	0	1	0
0	0	0	1	0
0	0	0	0	0
0	0	0	0	1
0	0	0	0	0
0	1	0	0	0
0	0	0	1	0
0	0	0	1	0
0	0	0	0	0
0	0	0	0	0
0	0	0	1	0
0	0	0	0	1
0	1	0	0	0
0	0	0	1	0
0	0	0	1	0
0	1	0	0	0

0	1	0	1	0
0	0	0	1	0
0	0	1	0	0
0	0	0	0	1
0	1	0	0	0
0	1	0	0	0
0	0	0	0	1
0	1	0	0	0
0	0	0	0	1
0	0	0	0	1
0	0	0	0	1
0	0	0	0	1
0	1	0	1	0
0	1	0	0	0
0	1	0	0	0
0	0	0	0	0
0	1	0	0	0
0	1	0	0	0
0	0	1	0	0
0	1	0	0	0
0	0	0	0	1
0	0	0	0	0
0	1	0	0	0
0	1	0	0	0
0	1	0	0	0
0	1	0	0	0
0	1	1	0	0
0	0	0	0	1
0	1	0	0	0
0	0	0	0	1
0	0	0	1	0
0	0	0	0	0
0	1	0	0	0
0	1	0	0	0
0	0	0	0	1
0	1	0	0	0
0	1	0	0	0
0	0	0	0	1
0	1	0	0	0
0	1	0	0	0
0	1	0	0	0
0	1	0	0	0
0	1	0	0	0
0	0	0	0	1
0	0	0	0	0
0	1	0	0	0
0	1	0	0	0
0	1	0	0	0

0	0	0	1	0
0	0	0	1	0
0	0	0	0	1
0	0	0	0	0
0	1	0	0	0
0	0	0	0	1
0	1	1	0	0
0	0	0	1	0
0	1	0	0	0
0	0	0	0	0
0	0	0	0	1
0	1	0	0	0
0	0	0	1	0
0	0	0	0	1
0	0	0	0	1
0	0	0	0	0
1	0	0	0	0
0	1	0	0	0
0	0	0	0	1
0	0	0	1	0
0	1	0	0	0
0	1	0	0	0
0	0	0	1	0
0	0	1	0	0
0	1	0	0	0
0	1	0	0	0
0	0	0	0	0
0	1	0	0	0
0	0	0	1	0
0	1	0	0	0
0	0	0	1	0
0	0	0	1	0
0	0	0	1	0
0	1	0	0	0
0	0	0	1	0
0	0	0	1	0
0	1	0	0	0
0	0	0	0	1
0	1	0	0	0
0	1	0	0	0
0	0	0	1	0
0	0	0	1	0
0	0	0	0	1
0	1	0	0	0
1	0	0	0	0
0	0	0	1	0
0	0	1	0	0
0	0	0	1	0

0	0	0	1	0
0	0	0	1	0
0	0	0	0	1
0	1	0	0	0
0	0	0	1	0
0	0	0	1	0
0	1	0	0	0
0	1	0	0	0
0	0	0	0	1
0	1	0	0	0
0	0	1	0	0
0	0	0	1	0
0	0	0	1	0
0	0	0	1	0
0	0	0	1	0
0	0	0	0	1
0	1	0	0	0
0	0	0	0	1
0	1	0	0	0
0	0	0	1	0
0	1	0	0	0
0	1	0	0	0
0	1	0	0	0
0	0	0	0	1
0	1	0	0	0
0	0	0	0	1
0	0	0	1	0
0	0	0	0	1
0	1	0	0	0
0	0	0	0	1
0	0	0	0	1
0	0	0	0	1
0	1	0	0	0
0	1	0	0	0
0	1	0	0	0
0	0	0	0	1
0	1	0	0	0
0	0	0	0	1
0	0	0	0	1
0	1	0	0	0
0	0	0	0	1
0	0	0	0	1
0	1	0	0	0
0	1	0	0	0
0	0	0	1	0
0	0	0	0	1
0	1	0	0	0
0	0	0	0	1

0	1	0	0	0
0	1	0	0	0
0	1	0	0	0
0	0	0	0	1
0	1	0	0	0
0	0	0	0	0
0	0	0	0	1
0	0	1	0	0
0	1	0	0	0
0	0	0	0	1
0	0	0	1	0
1	1	0	0	0
0	0	0	1	0
0	1	0	0	0
0	1	0	0	0
0	1	0	0	0
0	1	0	0	0
0	1	0	0	0
0	0	0	1	0
0	1	0	0	0
0	1	0	0	0
0	0	0	0	1
0	0	0	1	0
0	0	0	1	0
0	1	0	0	0
0	1	0	0	0
0	1	0	0	0
0	0	0	1	0
0	1	0	0	0
0	1	0	0	0
0	0	0	1	0
0	0	0	0	1
0	1	1	1	0
0	1	0	0	0
0	1	0	0	0
0	1	1	0	0
0	1	0	0	0
0	1	0	0	0
0	1	0	0	0
0	0	0	1	0
0	1	0	0	0
0	1	0	0	0
0	0	0	1	0
0	0	0	1	0
0	1	0	0	0
0	1	0	0	0
0	0	0	1	0
0	0	0	1	0

0	1	0	0	0
0	1	0	0	0
0	0	0	1	0
0	1	0	0	0
0	1	0	0	0
0	0	0	0	1
0	0	0	1	0
0	0	0	1	0
0	1	0	0	0
0	0	0	0	0
0	1	0	0	0
0	1	0	0	0
0	0	0	1	0
0	1	0	0	0
0	1	0	0	0
0	1	0	0	0
0	0	0	0	0
0	0	0	1	0
0	0	1	0	0
0	1	0	0	0
0	0	0	1	0
0	1	0	0	0
0	1	0	0	0
0	0	0	1	0
0	0	0	1	0
0	1	0	0	0
0	0	0	1	0
0	0	0	1	0
0	0	0	1	0
0	0	0	1	0
0	1	0	0	0
0	1	0	1	0
0	1	0	0	0
0	1	0	1	0
0	1	0	0	0
0	1	0	0	0
0	1	0	0	0
0	1	0	0	0
0	0	0	0	1
0	0	0	0	1
0	1	0	0	0
0	0	1	0	0
0	0	0	1	0
0	1	0	0	0
0	0	0	1	0
0	0	0	0	1
0	1	0	0	0
0	1	0	0	0

0	0	0	1	0
0	0	1	0	0
0	0	0	0	1
0	1	0	0	0
0	1	0	0	0
0	1	0	0	0
0	1	0	0	0
0	0	0	0	1
0	1	0	0	0
0	1	0	0	0
0	0	0	1	0
0	1	0	0	0
0	0	0	0	1
0	0	0	0	1
0	0	0	0	1
0	0	0	0	1
0	1	0	0	0
0	0	0	0	1
0	1	0	0	0
0	1	0	0	0
0	1	0	0	0
0	0	0	0	1
0	1	0	0	0
0	1	0	0	0
0	0	1	0	0
0	1	0	0	0
0	1	0	0	0
0	0	0	1	0
0	0	0	0	1
0	0	0	0	1
0	0	0	0	1
0	0	0	0	1
0	1	0	0	0
0	0	0	0	0
0	0	0	1	0
0	1	0	0	0
0	0	0	0	1
0	0	0	0	1
0	0	0	0	0
0	0	0	1	0
0	1	0	0	0
0	0	0	0	1
0	0	0	0	1
0	1	0	0	0
0	1	0	0	0
0	1	0	0	0
0	0	0	0	1
0	0	0	0	0

0	0	0	0	1
0	1	0	1	0
0	0	0	0	1
0	0	0	0	1
0	0	0	0	1
0	0	0	0	1
0	1	0	0	0
0	1	0	0	0
0	0	0	0	0
0	0	0	0	1
0	1	0	0	0
0	0	0	0	1
0	0	1	0	0
0	0	0	0	1
0	1	0	0	0
0	0	0	1	0
0	0	0	1	0
0	0	0	0	1
0	0	0	0	1
0	1	0	0	0
0	0	0	0	1
0	0	0	0	1
0	0	0	0	1
0	0	0	0	0
0	0	0	0	1
0	1	0	0	0
0	1	0	0	0
0	1	0	0	0
0	1	0	0	0
0	1	0	0	0
0	1	0	0	0
0	0	0	1	0
0	0	0	1	0
0	1	0	0	0
0	1	0	0	0
0	0	0	0	1
0	0	0	0	1
0	1	0	0	0
0	0	0	0	0
0	1	0	0	0
0	0	0	0	1
0	0	0	0	1
0	0	0	0	1
0	0	0	0	1
0	0	0	1	0
0	1	0	0	0
0	0	0	0	1
0	1	0	0	0

0	0	0	0	1
0	0	0	0	1
0	1	0	0	0
0	0	0	0	1
0	0	0	0	1
0	0	0	0	1
0	0	0	0	1
0	0	0	1	0
0	0	0	1	0
0	1	0	0	0
0	1	0	0	0
0	1	0	0	0
0	0	0	0	1
0	0	0	1	0
0	0	0	0	1
0	0	0	0	1
0	0	0	1	0
0	0	0	0	1
0	1	0	0	0
0	0	0	0	1
0	0	0	0	1
0	1	0	0	0
0	0	0	0	1
0	1	0	0	0
0	0	0	0	1
0	1	0	0	0
0	1	0	0	0
0	1	0	0	0
0	1	0	0	0
0	1	0	0	0
0	0	0	0	1
0	0	1	0	0
0	1	0	0	0
0	0	0	0	1
0	1	0	1	0
0	0	0	1	0
0	0	0	1	0
0	1	0	0	0
0	0	0	1	0
0	1	0	0	0
0	1	0	0	0
0	0	0	0	1
0	1	0	0	0
0	0	0	0	1
0	0	0	0	1
0	1	0	0	0
0	0	1	0	0
0	1	0	0	0

0	0	0	0	1
0	1	0	0	0
0	0	0	1	0
0	1	0	0	0
0	0	0	0	1
0	1	0	0	0
0	1	1	0	0
0	0	0	1	0
0	1	0	0	0
0	0	0	0	0
0	0	0	0	1
0	0	0	0	1
0	0	0	1	0
0	1	0	0	0
0	1	0	0	0
0	0	0	0	1
0	0	0	0	0
0	0	0	0	1
0	0	0	0	1
0	0	0	0	1
0	0	0	0	1
0	0	0	1	0
0	1	0	0	0
0	0	0	0	1
0	0	0	0	1
0	1	0	1	0
0	0	0	0	1
0	0	0	0	1
0	1	0	0	0
0	0	0	0	0
0	0	0	0	1
0	0	0	0	1
0	0	0	0	1
0	1	0	0	0
0	0	0	0	1
0	0	0	0	1
0	0	0	0	1
0	1	0	0	0
0	1	0	0	0
0	0	0	0	1
0	1	0	0	0
0	0	0	0	1
0	0	0	0	1
0	0	0	0	0
0	1	0	0	0
0	1	0	0	0
0	0	0	0	1
0	0	0	0	1

0	0	0	0	1
0	0	0	1	0
0	1	0	0	0
0	0	0	0	0
0	0	0	0	1
0	1	0	0	0
0	0	0	0	1
0	1	0	0	0
0	1	1	0	0
0	1	0	0	0
0	1	0	0	0
0	1	0	0	0
0	0	0	0	1
0	1	0	0	0
0	0	0	0	0
0	1	0	0	0
0	1	0	0	0
0	1	0	0	0
0	1	0	0	0
0	0	0	0	1
0	1	0	0	0
0	0	0	0	1
0	0	0	0	1
0	1	0	0	0
0	0	0	1	0
0	1	0	0	0
0	1	0	0	0
0	0	0	0	1
0	0	0	0	1
0	0	0	0	1
0	0	0	1	0
0	0	0	0	1
0	1	0	0	0
0	1	0	0	0
0	0	0	0	1
0	1	0	0	0
0	0	0	1	0
0	0	0	0	1
0	0	0	0	1
0	1	0	0	0
0	0	0	0	1
0	0	0	0	1
0	1	0	0	0
0	1	0	0	0
0	0	0	1	0
0	0	0	1	0
0	0	0	0	1
0	1	0	0	0

0	0	0	0	1
0	0	0	1	0
0	0	1	0	0
0	0	0	0	1
0	0	0	1	0
0	1	0	0	0
0	1	0	0	0
0	0	0	0	1
0	0	0	1	1
0	1	0	0	0
0	1	0	0	0
0	0	0	0	1
0	0	0	0	1
0	1	0	0	0
0	0	0	0	1
0	1	0	0	0
0	0	0	0	1
0	0	0	0	0
0	1	0	1	0
0	1	0	0	0
0	1	0	0	0
0	1	0	0	0
0	0	0	0	1
0	0	0	1	0
0	1	0	0	0
0	1	0	0	0
0	1	0	0	0
0	0	0	1	0
0	0	0	1	0
0	1	0	0	0
0	1	0	0	0
0	1	0	1	0
0	1	0	0	0
0	0	0	0	1
0	1	0	0	0
0	0	0	1	0
0	1	0	0	0
0	1	0	0	0
0	0	0	0	0
0	0	0	1	0
0	1	0	0	0
0	0	0	0	1
0	0	0	0	1
0	0	0	1	0
0	0	0	0	1
0	0	0	1	0
0	0	0	0	1
0	0	0	1	0

0	0	0	0	1
0	0	0	1	0
0	0	0	1	0
0	1	0	0	0
0	0	0	0	1
0	0	1	0	0
0	1	0	0	0
0	1	0	0	0
0	0	0	0	1
0	0	0	0	1
0	0	0	0	1
0	1	0	0	0
0	0	0	0	1
0	1	0	0	0
0	1	0	0	0
0	1	0	0	0
0	0	0	0	1
0	0	0	0	1
0	1	0	0	0
0	1	0	0	0
0	1	0	0	0
0	1	0	0	0
0	1	0	0	0
0	0	0	1	0
0	1	0	0	0
0	0	0	0	1
0	1	0	0	0
0	1	0	0	0
0	1	0	0	0
0	1	0	0	0
0	0	0	0	1
0	1	0	0	0
0	1	0	0	0
0	0	0	0	1
0	1	0	0	0
0	0	0	0	0
0	0	0	0	1
0	1	0	0	0
0	0	0	1	0
0	0	1	0	0
0	1	0	0	0
0	0	0	0	1
0	1	0	0	0
0	0	0	0	1
0	1	0	1	0
0	0	0	1	0
0	1	0	0	0
0	1	0	0	0

0	0	0	1	0
0	0	0	1	0
0	0	0	1	0
0	0	0	0	1
0	1	0	1	0
0	1	0	0	0
0	1	0	0	0
0	0	0	0	1
0	1	0	0	0
0	1	0	0	0
0	1	0	0	0
0	1	0	0	0
0	0	0	1	0
0	1	0	0	0
0	0	0	1	0
0	0	0	0	1
0	1	0	0	0
0	1	0	1	0
0	0	0	0	1
0	0	0	1	0
0	1	0	0	0
0	1	0	0	0
0	0	0	1	0
0	1	0	0	0
0	1	0	0	0
0	0	0	1	0
0	0	0	0	1
0	1	0	1	0
0	0	0	1	0
0	0	0	0	1
0	0	0	0	1
0	0	0	0	1
0	0	0	0	1
0	1	0	0	0
0	1	0	0	0
0	0	0	1	0
0	0	0	0	1
0	0	0	0	1
0	1	0	0	0
0	0	0	0	1
0	0	0	0	1
0	0	0	0	1
0	0	0	0	1
0	1	0	0	0
0	0	0	0	1
0	0	0	0	1
0	1	0	0	0
0	1	0	1	0

0	0	0	0	1
0	0	1	0	0
0	1	0	0	0
0	1	0	0	0
0	1	0	0	0
0	0	0	0	1
0	0	1	0	0
0	1	0	0	0
0	0	0	0	0
0	1	0	0	0
0	0	1	0	0
0	0	0	0	1
0	0	0	0	1
0	1	0	0	0
0	0	0	0	0
0	1	0	0	0
0	1	0	0	0
0	1	0	0	0
0	1	0	0	0
0	1	0	0	0
0	0	0	0	0
0	0	0	0	1
0	1	0	0	0
0	1	0	0	0
0	0	0	0	1
0	1	0	0	0
0	1	0	0	0
0	0	0	0	1
0	0	0	0	0
0	1	0	0	0
0	0	0	0	1
0	0	0	0	1
0	0	0	1	0
0	1	0	0	0
0	1	0	0	0
0	0	0	1	0
0	0	0	0	1
0	0	0	0	0
0	0	0	0	1
0	0	0	0	1
0	0	0	0	1
0	1	0	0	0
0	1	0	0	0
0	0	0	0	0
0	1	0	0	0
0	1	0	0	0
0	1	0	0	0
0	0	0	0	1

0	0	0	1	0
0	0	0	0	0
0	0	0	0	1
0	1	0	0	0
0	1	0	0	0
0	1	0	0	0
0	1	0	0	0
0	1	0	0	0
0	1	0	0	0
0	0	0	0	1
0	0	0	0	1
0	0	0	0	0
0	1	0	0	0
0	0	0	0	1
0	1	0	0	0
0	1	0	0	0
0	0	0	0	0
0	1	0	0	0
0	0	1	0	0
0	1	0	0	0
0	0	0	0	1
0	1	0	0	0
0	0	0	0	1
0	0	0	1	0
0	0	0	1	0
0	0	0	0	1
0	0	0	0	1
0	1	0	0	0
0	1	0	0	0
0	1	0	0	0
0	1	0	0	0
0	0	0	1	0
0	0	0	0	1
0	0	0	0	0
0	1	0	0	0
0	0	0	1	0
0	1	0	0	0
0	1	0	1	0
0	1	0	0	0
0	0	0	0	1
0	0	0	1	0
0	0	0	0	1
0	1	0	0	0
0	0	0	0	1
0	0	0	1	0
0	0	0	0	1
0	0	0	0	1
0	1	0	0	0

0	0	0	0	1
0	0	0	0	1
0	0	0	1	0
0	1	0	0	0
0	0	0	0	1
0	0	0	0	1
0	0	0	0	1
0	0	1	0	0
0	0	0	0	1
0	0	0	1	0
0	0	0	1	0
0	1	0	0	0
0	1	0	1	0
0	0	0	0	1
0	0	0	0	1
0	0	0	0	1
0	0	0	0	1
0	0	0	0	1
0	0	0	0	1
0	0	0	0	1
0	1	0	0	0
0	1	0	0	0
0	0	0	1	0
0	1	0	0	0
0	1	0	0	0
0	1	0	0	0
0	1	0	0	0
0	1	0	0	0
0	0	0	0	1
0	0	0	0	0
0	0	0	0	1
0	0	0	0	1
0	1	0	0	0
0	1	1	0	0
0	0	1	1	0
0	1	0	0	0
0	1	0	0	0
0	0	0	0	1
0	0	0	0	1
0	1	0	0	0
0	0	0	0	1
0	0	0	1	0
0	0	0	0	1
0	0	0	0	1
0	0	0	0	1
0	1	0	0	0
0	1	0	0	0
0	1	0	0	0

1	0	0	0	0
0	0	0	0	1
0	0	0	1	0
0	1	0	0	0
0	0	0	0	1
0	0	0	0	0
0	0	0	0	1
0	0	0	0	1
0	1	0	0	0
0	1	0	0	0
0	0	0	0	0
0	0	0	0	1
0	1	0	0	0
0	0	0	1	0
0	0	0	0	1
0	1	0	0	0
0	1	0	0	0
0	0	0	0	1
0	0	0	0	1
0	0	0	0	1
0	0	0	1	0
0	0	0	0	1
0	0	0	0	1
0	0	0	0	1
0	0	0	1	0
0	0	0	0	0
0	0	0	0	1
0	0	0	0	1
0	0	0	0	1
0	0	0	1	0
0	0	0	1	0
0	1	0	0	0
0	1	0	0	0
0	1	0	0	0
0	1	0	0	0
0	1	0	0	0
0	0	0	0	1
0	0	0	1	0
0	0	0	0	1
0	0	0	1	0
0	0	0	0	0
0	0	1	0	0
0	1	0	0	0
0	1	0	0	0
0	1	0	0	0
0	0	0	0	1
0	0	0	0	1
0	1	0	0	0

0	1	0	0	0
0	0	0	0	1
0	0	0	0	1
0	1	0	0	0
0	1	0	0	0
0	1	0	0	0
0	0	0	0	1
0	1	0	0	0
0	1	0	0	0
0	0	0	0	1
0	0	0	0	1
0	0	0	0	1
0	1	0	0	0
0	0	0	0	1
0	1	0	0	0
0	0	0	1	0
0	0	0	0	1
0	0	0	0	1
0	0	0	1	0
0	0	0	0	1
0	1	0	0	0
0	0	0	1	0
0	1	0	0	0
0	0	0	0	1
0	1	0	0	0
0	1	0	0	0
0	1	0	0	0
0	0	0	0	1
0	1	0	0	0
0	1	0	0	0
0	1	0	0	0
0	0	0	0	1
0	0	0	0	0
0	0	0	0	1
0	1	0	0	0
0	0	0	1	0
0	0	0	0	1
0	0	0	0	1
0	0	0	0	1
0	0	0	0	1
0	0	1	0	0
0	0	0	0	1
0	0	0	0	1
0	1	0	0	0
0	1	0	0	0
0	1	0	0	0
0	1	0	0	0
0	0	0	0	1

0	0	0	0	1
0	1	0	0	0
0	0	0	0	0
0	1	0	0	0
0	0	0	0	0
0	1	0	0	0
0	0	0	0	1
0	0	0	0	1
0	1	0	1	0
0	0	0	0	1
0	1	0	0	0
0	0	0	0	1
0	0	0	0	1
0	0	0	0	1
0	1	0	1	0
0	0	0	0	1
0	1	0	0	0
0	0	0	0	1
0	0	0	0	1
0	0	0	1	0
0	1	0	0	0
0	1	0	0	0
0	1	0	0	0
0	0	0	0	1
0	0	1	0	0
0	1	0	0	0
0	1	0	0	0
0	1	0	0	0
0	1	0	0	0
0	1	0	0	0
0	1	0	0	0
0	0	0	0	1
0	1	0	0	0
0	0	0	0	1
0	1	0	0	0
0	1	0	0	0
0	0	0	1	0
0	1	0	0	0
0	0	0	1	0
0	0	0	1	0
0	0	0	1	0
1	1	1	1	0
0	1	0	0	0
0	1	0	0	0
0	0	0	1	0
0	0	0	1	0
0	0	0	0	1
0	0	0	0	0

0	1	0	0	0	
0	0	0	0	1	
0	0	0	0	1	
0	1	0	0	0	
0	1	0	0	0	
0	0	0	0	1	
0	0	0	1	0	hispanic
0	0	0	0	1	
0	1	0	0	0	
0	0	0	1	0	latino
0	1	0	0	0	
0	1	0	0	0	
0	0	0	1	0	Indan
0	0	0	1	0	Hispanic
0	1	0	0	0	
0	1	0	0	0	
0	1	0	0	0	
0	1	0	0	0	
0	1	0	0	0	
0	1	0	0	0	
0	1	0	0	0	
0	1	0	0	0	
0	1	0	0	0	
0	0	0	0	0	
0	1	0	0	0	
0	0	0	0	1	
0	0	0	0	1	
0	1	0	0	0	
0	1	0	0	0	
0	1	0	0	0	
0	0	0	0	1	
0	1	0	0	0	
0	0	0	0	1	
0	0	0	0	1	
0	0	0	1	0	latino
0	0	0	1	0	hispanic
0	0	0	0	1	
0	0	0	1	0	
0	0	0	0	1	
0	1	0	0	0	
0	1	0	0	0	
0	0	0	0	0	
0	1	0	0	0	
0	0	0	0	0	
0	1	0	0	0	
0	1	0	0	0	
0	1	0	0	0	
0	0	0	1	0	Hispanic

0	1	1	0	0	
0	1	0	0	0	
0	0	0	1	0	No answer
0	0	0	1	0	hispanic
0	0	0	0	1	
0	0	0	1	0	
0	1	0	0	0	
0	1	0	0	0	
0	1	0	0	0	
0	1	0	0	0	
0	0	0	1	0	half white and hispanic
0	1	0	0	0	
0	0	0	0	1	
0	0	0	1	0	latino
0	1	0	0	0	
0	0	0	0	1	
0	0	0	0	1	
0	1	0	0	0	
0	1	0	1	0	hispanic
0	1	0	0	0	
0	1	0	0	0	
0	0	0	0	1	
0	1	0	0	0	
0	1	0	0	0	
0	1	0	0	0	
0	1	0	0	0	
0	1	0	0	0	
0	0	0	1	0	hispanic/latino
0	1	0	0	0	
0	1	0	0	0	
0	1	0	0	0	
0	1	0	0	0	
0	1	0	0	0	
0	0	0	1	0	hispanic
0	1	0	0	0	
0	1	0	0	0	
0	0	0	0	1	
0	1	0	0	0	
0	1	0	0	0	
0	0	0	0	1	
0	0	0	1	0	hispanic
0	0	0	1	0	mexican
0	0	0	1	0	hispanic
0	1	0	0	0	
0	1	1	0	0	
0	0	0	0	1	
0	0	0	0	0	
0	1	0	0	0	

0	0	0	0	1	
0	0	0	0	1	
0	1	0	0	0	
0	0	0	1	0	no answer given
0	1	0	0	0	
0	0	0	0	1	
0	0	0	0	1	
0	1	0	0	0	
0	0	0	0	1	
0	0	0	0	1	
0	0	0	0	1	
0	1	0	0	0	
0	1	0	0	0	
0	1	0	0	0	
0	1	0	0	0	
0	0	0	0	1	
0	0	0	0	1	
0	0	0	0	1	
0	1	0	0	0	
0	0	0	0	1	
0	0	0	0	1	
0	1	0	0	0	
0	1	0	0	0	
0	0	0	0	1	
0	0	0	0	1	
0	1	0	0	0	
0	1	0	0	0	
0	1	0	0	0	
0	0	0	0	1	
0	0	0	0	1	
0	0	0	0	1	
0	1	0	1	0	hispanic
0	0	0	1	0	no answer given
0	0	0	0	1	
0	0	0	0	0	
0	1	0	0	0	
0	0	0	0	1	
0	0	0	0	1	
0	0	0	0	1	
0	1	0	0	0	
0	0	0	1	0	mexican
0	0	0	0	1	
0	1	0	0	0	
0	0	0	0	1	
0	1	0	0	0	
0	1	0	0	0	
0	0	0	0	0	
0	0	0	1	0	no answer given

0	0	0	0	1	
0	0	0	0	1	
0	1	0	0	0	
0	0	0	1	0	American
0	0	0	1	0	no answer given
0	0	0	1	0	no answer given
0	0	0	1	0	hispanic
0	0	0	1	0	mexican
0	0	0	1	0	Mexicano
0	0	0	1	0	no answer given
0	0	0	1	0	hispanic
0	0	0	1	0	Hispanic
0	0	0	1	0	no answer given
0	0	0	1	0	White hispanic
0	0	0	1	0	hispanic
0	1	0	0	0	
0	1	0	0	0	
0	1	0	0	0	
0	0	1	0	0	
0	1	0	0	0	
0	0	0	1	0	white/hispanic
0	1	0	0	0	
0	1	0	0	0	
0	1	0	0	0	
0	1	0	0	0	
0	1	0	0	0	
0	1	0	0	0	
0	1	0	0	0	
0	1	0	0	0	
0	0	0	1	0	Hispanic
0	1	0	0	0	
0	1	0	0	0	
0	0	0	1	0	no answer given
0	0	1	0	0	
0	1	0	0	0	
0	1	0	0	0	
0	1	1	1	0	By racial
0	0	0	0	1	
0	1	0	0	0	
0	0	1	0	0	
0	1	0	0	0	
0	1	0	0	0	
0	0	0	0	1	
0	1	0	0	0	
0	0	0	1	0	hispanic
1	0	0	0	0	
0	0	0	0	1	
0	1	0	0	0	

0	1	0	0	0	
0	1	0	0	0	
0	1	0	0	0	
0	0	0	0	0	
0	1	0	0	0	
0	1	0	0	0	
0	1	0	0	0	
0	1	0	0	0	
0	0	0	0	1	
0	0	0	0	1	
0	1	0	0	0	
0	0	0	0	1	
0	1	0	0	0	
0	1	0	0	0	
0	1	0	0	0	
0	1	0	0	0	
0	0	0	0	1	
0	0	0	0	1	
0	0	0	0	1	
0	0	0	0	1	
0	0	0	1	0	latina/hispanic
0	0	0	0	1	
0	0	0	0	1	
0	0	0	0	1	
0	0	0	1	0	spainurd
0	1	0	0	0	
0	1	0	0	0	
0	1	0	0	0	
0	0	0	0	1	
0	0	0	0	1	
0	1	0	0	0	
0	0	0	1	0	latino
0	1	0	0	0	
0	1	0	0	0	
0	1	0	0	0	
0	1	0	0	0	
1	0	0	0	0	
0	0	0	0	1	
0	0	0	1	0	Hispanic/White
0	1	0	0	0	
0	1	0	0	0	
0	0	0	0	1	
0	1	0	0	0	
0	0	0	0	1	
0	0	0	1	0	Mexican
0	0	0	0	1	
0	1	0	0	0	
0	0	0	0	1	

0	0	0	0	1	
0	1	0	0	0	
0	0	0	0	1	
0	1	0	0	0	
0	1	0	0	0	
0	1	0	0	0	
0	1	0	0	0	
0	1	0	1	0	American
0	0	0	0	1	
0	1	0	0	0	
0	0	0	1	0	white/native american
0	0	0	0	1	
0	1	0	0	0	
0	0	0	1	0	no answer given
0	0	0	0	1	
0	0	0	0	0	
0	0	0	0	1	
0	1	0	0	0	
0	0	0	1	0	italian, black, cherricy indian
0	1	0	0	0	
0	1	0	1	0	and Indian
0	0	0	0	1	
0	0	0	0	1	
0	1	0	0	0	
0	1	0	0	0	
0	0	0	0	1	
0	0	0	0	1	
0	1	0	1	0	Mexican
0	0	0	0	1	
0	1	0	0	0	
0	1	0	0	0	
0	0	0	1	0	American/Mexico
0	1	0	0	0	
0	0	0	0	1	
0	0	0	0	1	
0	0	0	0	1	
0	1	0	0	0	
0	0	0	0	1	
0	0	0	1	0	Latino
0	0	0	0	1	
0	0	0	0	1	
0	1	0	0	0	
0	1	0	0	0	
0	0	0	0	1	
0	0	0	0	1	
0	1	0	0	0	
0	0	0	0	1	
0	0	0	0	1	

0	0	0	0	1	
0	1	0	0	0	
0	0	0	0	1	
0	0	0	0	1	
0	0	0	0	1	
0	1	0	0	0	
0	0	1	0	0	
0	1	0	0	0	
0	0	0	0	1	
0	0	0	0	1	
0	0	0	0	1	
0	1	0	0	0	
0	1	0	0	0	
0	1	0	0	0	
0	0	0	0	1	
0	0	0	0	1	
0	1	0	0	0	
0	1	0	0	0	
0	0	0	0	1	
0	1	0	0	0	
0	1	0	0	0	
0	1	0	0	0	
0	1	1	1	0	German/Irish
0	1	0	0	0	
0	1	0	0	0	
0	0	0	0	1	
0	0	0	0	1	
0	1	0	0	0	
0	1	0	0	0	
0	1	0	1	0	Mexican
0	0	0	1	0	Hispanic
0	1	0	0	0	
0	1	0	0	0	
0	1	0	0	0	
0	1	0	0	0	
0	1	0	0	0	
0	0	0	0	1	
0	0	0	0	1	
0	0	0	0	1	
0	0	0	0	1	
0	0	0	0	1	
0	1	0	0	0	
0	1	0	0	0	
0	0	0	0	1	
0	0	0	0	0	
0	0	0	0	1	
0	1	0	0	0	
0	1	0	0	0	

0	1	0	0	0	
0	1	0	0	0	
0	0	0	1	0	White Hispanic
0	0	1	0	0	
0	1	0	0	0	
0	1	0	0	0	
0	1	1	0	0	
0	1	0	0	0	
0	0	0	0	1	
0	0	0	0	1	
0	0	0	0	1	
0	1	0	0	0	
0	0	0	1	0	Hispanic
0	0	0	0	1	
0	1	0	1	0	German
0	1	0	0	0	
0	0	0	0	1	
0	0	0	0	1	
0	0	0	0	1	
0	0	0	0	1	
0	1	0	0	0	
0	1	0	0	0	
0	0	0	0	1	
0	0	0	0	0	
0	0	0	0	1	
0	0	0	0	1	
0	0	0	1	0	Half American Half Mexican
0	1	0	0	0	
0	0	0	0	1	
0	0	0	0	0	
0	0	0	0	0	
0	0	0	0	1	
0	0	0	0	1	
0	0	0	0	1	
0	0	0	0	1	
0	0	0	0	1	
0	0	0	0	1	
0	0	0	0	1	
0	0	0	1	0	am spanish
0	0	0	0	1	
0	1	0	0	0	
0	0	0	0	1	
0	1	0	0	0	
0	1	0	0	0	
0	1	0	0	0	
0	1	0	0	0	
0	1	0	0	0	
0	0	0	0	1	

0	1	0	0	0	
0	0	0	0	1	
0	0	0	0	1	
0	0	0	0	1	
0	0	0	0	1	
0	0	0	0	1	
0	1	0	0	0	
0	1	0	0	0	
0	1	0	0	0	
0	0	0	1	0	mexican
0	1	0	0	0	
0	1	0	0	0	
0	0	0	0	1	
0	0	0	0	1	
0	1	0	0	0	
0	1	0	0	0	
0	1	0	0	0	
0	0	0	0	1	
0	0	0	0	1	
0	1	0	0	0	
0	0	0	0	0	
0	0	0	0	1	
0	0	0	0	1	
0	1	0	0	0	
0	1	0	0	0	
1	1	1	0	0	
0	0	0	0	1	
0	0	0	1	0	no answer given
0	1	0	0	0	
0	0	0	0	1	
0	0	0	1	0	hispanic
0	0	0	0	1	
0	0	0	0	1	
0	1	0	0	0	
0	1	0	0	0	
0	0	0	0	1	
0	0	0	1	0	no answer given
0	0	0	1	0	hispanic
0	0	0	0	1	
0	0	0	0	1	
0	0	0	0	1	
0	0	0	0	1	
0	0	0	0	1	
0	0	0	1	0	hispanic
0	0	0	0	1	
0	1	0	0	0	
0	1	0	0	0	
0	0	0	1	0	no answer given

0	1	0	0	0	
0	0	0	0	1	
0	1	0	0	0	
0	1	0	0	0	
0	1	0	0	0	
0	0	0	0	1	
0	0	0	0	1	
0	1	0	0	0	
0	1	0	0	0	
0	1	0	0	0	
0	0	0	0	1	
0	1	0	0	0	
0	0	1	0	0	
0	0	0	0	1	
0	0	0	0	1	
0	1	0	0	0	
0	0	0	0	1	
0	0	0	1	0	Hispanic
0	0	0	0	1	
0	0	0	0	1	
0	0	0	0	1	
0	1	0	0	0	
0	0	0	0	1	
0	0	0	0	1	
0	1	0	0	0	
0	0	0	1	0	No Response
0	0	0	0	0	
0	0	0	0	0	
0	0	0	0	1	
0	0	0	0	0	
0	1	0	0	0	
0	0	0	0	1	
0	1	0	1	0	Mexican
0	0	0	0	0	
0	1	0	0	0	
0	1	0	0	0	
0	0	0	0	0	
0	1	0	0	0	
0	0	0	0	1	
0	1	0	0	0	
0	0	0	0	0	
0	1	0	0	0	
0	0	0	0	1	
0	0	0	1	0	No Response
0	0	0	0	0	
0	0	0	0	1	
0	0	0	0	1	
0	0	0	0	1	

0	1	0	0	0	
0	0	0	1	0	Hispanic
0	1	0	0	0	
0	0	0	0	1	
0	0	0	0	1	
0	0	0	0	1	
0	0	0	0	1	
0	1	0	0	0	
0	1	0	0	0	
0	1	0	0	0	
0	0	0	0	1	
0	0	0	0	1	
0	0	0	0	1	
0	0	0	0	1	
0	1	0	1	0	American
0	0	0	0	0	
0	1	0	0	0	
0	0	0	0	1	
0	0	0	0	0	
0	0	0	1	0	Hispanic
0	0	0	0	1	
0	0	0	0	1	
0	0	0	1	0	Mexicana and Americana
0	0	0	1	0	Hispanic/Latina
0	1	0	0	0	
0	0	0	0	1	
0	0	0	0	1	
1	1	0	0	0	
0	1	0	0	0	
0	0	0	0	1	
0	1	0	0	0	
0	1	1	0	0	
0	1	0	0	0	
0	0	1	0	0	
0	1	0	0	0	
0	0	0	0	1	
0	0	0	0	1	
0	1	0	0	0	
0	1	0	0	0	
0	1	0	0	0	
0	0	0	0	1	
0	1	0	0	0	
0	1	0	0	0	
0	0	0	0	1	
0	1	0	0	0	
0	1	0	0	0	
0	0	0	0	1	
0	1	0	0	0	

0	1	0	0	0	
0	0	0	0	0	
0	1	0	0	0	
0	1	0	0	0	
0	0	0	0	1	
0	0	0	0	1	
0	1	0	0	0	
0	0	0	0	0	
0	1	0	0	0	
0	0	0	0	1	
0	0	0	0	1	
0	0	0	0	0	
0	0	0	0	1	
0	0	0	0	0	
0	0	0	0	1	
0	1	0	0	0	
0	1	0	0	0	
0	1	0	1	0	Hispanic
0	1	0	0	0	
0	1	0	1	0	pordarican
0	0	0	0	1	
0	0	0	0	1	
0	1	0	0	0	
0	0	0	0	1	
0	0	0	0	1	
0	1	0	0	0	
0	0	0	0	1	
0	0	1	0	0	
0	0	1	0	0	
0	1	0	0	0	
0	1	0	0	0	
0	0	0	0	1	
0	1	0	0	0	
0	0	0	0	1	
0	1	0	0	0	
0	0	0	0	1	
0	0	0	1	0	Dominican
0	1	0	0	0	
0	1	0	0	0	
0	1	0	0	0	
0	0	0	1	0	Mexican American
0	1	0	0	0	
0	1	0	0	0	
0	0	0	0	1	
0	1	0	0	0	
0	0	0	1	0	Idk
0	0	0	0	1	
0	0	0	0	1	

0	1	0	0	0	
0	1	0	1	0	Hispanic
0	1	0	0	0	
0	1	0	0	0	
0	0	0	0	1	
0	0	0	0	1	
0	1	0	0	0	
0	1	0	0	0	
0	1	0	0	0	
0	1	0	0	0	
0	0	0	0	1	
0	1	0	0	0	
0	1	0	0	0	
0	1	0	0	0	
0	0	0	1	0	Hispanic/White
0	0	0	1	0	Hispanic
0	1	0	0	0	
0	0	0	0	1	
0	1	0	0	0	
0	0	0	0	1	
0	0	0	0	1	
0	0	0	0	1	
0	0	0	0	1	
0	1	0	0	0	
0	0	0	0	1	
0	0	0	0	0	
0	1	0	0	0	
0	1	0	0	0	
0	0	0	0	0	
0	1	0	0	0	
0	0	0	0	1	
0	0	0	0	1	
0	1	0	0	0	
0	0	0	0	1	
0	0	0	0	1	
0	0	0	0	1	
0	0	0	0	0	
0	1	0	0	0	
0	0	0	1	0	Hispanic
0	1	0	0	0	
0	0	0	0	0	
0	0	0	0	1	
0	1	0	0	0	
0	1	0	0	0	
0	0	0	0	1	
0	0	0	1	0	Latino
0	0	0	0	0	
0	1	0	0	0	

0	1	0	0	0	
0	0	0	1	0	Half white and hispanic
0	0	0	0	1	
0	1	0	1	0	No Response
0	1	0	0	0	
1	1	0	0	0	
0	0	0	1	0	No Response
0	0	0	0	1	
0	0	0	0	1	
0	0	0	0	1	
0	1	0	0	0	
0	1	1	0	0	
0	1	0	0	0	
0	0	0	0	1	
0	0	0	1	0	Toaster
0	0	0	0	1	
0	1	0	0	0	
0	0	0	1	0	Hispanic
0	0	0	0	1	
0	0	0	0	1	
0	0	0	0	1	
0	1	0	0	0	
0	0	0	0	1	
0	0	0	0	1	
0	0	0	1	0	No Response
0	0	0	1	0	Hispanic
0	0	0	0	1	
0	0	0	0	1	
0	0	0	0	1	
0	0	0	0	1	
0	1	0	0	0	
0	1	0	0	0	
0	1	0	0	0	
0	1	0	0	0	
0	0	0	0	1	
0	1	0	0	0	
0	0	0	0	1	
0	1	0	0	0	
0	1	0	0	0	
0	1	0	0	0	
0	1	0	0	0	
0	1	0	0	0	
0	0	0	1	0	Response
0	1	0	0	0	
0	1	0	0	0	
0	0	0	0	1	
0	1	0	0	0	
0	1	0	0	0	

0	1	0	0		0	
0	0	0	1		0	Mexican
0	0	0	0		1	
0	0	0	0	#NULL!		
0	1	0	0	#NULL!		
0	1	0	0	#NULL!		
0	0	0	0	#NULL!		
0	1	0	0	#NULL!		
0	1	0	0	#NULL!		
0	0	0	0	#NULL!		
0	0	0	1	#NULL!		
0	1	0	0	#NULL!		
0	1	0	0	#NULL!		
0	1	0	0	#NULL!		
0	1	0	0	#NULL!		
0	1	0	0	#NULL!		
0	0	0	1	#NULL!		Mexican
0	0	0	1	#NULL!		Hispanic
0	1	0	0	#NULL!		
0	1	0	0	#NULL!		
0	0	0	1	#NULL!		bi-racial
0	1	0	0	#NULL!		
0	1	0	0	#NULL!		
0	0	0	1	#NULL!		
0	0	0	1	#NULL!		hispanic
0	0	0	1	#NULL!		mexican
0	1	0	0	#NULL!		
0	0	0	1	#NULL!		Hispanic
0	1	0	0	#NULL!		
0	0	0	0	#NULL!		
0	0	0	1	#NULL!		Mexican
0	0	0	1	#NULL!		100% mexican
0	0	0	1	#NULL!		Hispanic
0	1	0	0	#NULL!		
0	1	0	0	#NULL!		
0	1	0	0	#NULL!		
0	0	0	1	#NULL!		hispaanic
0	1	0	0	#NULL!		
0	0	0	1	#NULL!		latino
0	0	0	1	#NULL!		I'm Hispanic
0	1	0	0	#NULL!		
0	0	0	1	#NULL!		hispanic
0	1	0	0	#NULL!		
0	0	0	1	#NULL!		mexican
0	1	0	0	#NULL!		
0	1	0	0	#NULL!		
0	1	0	0	#NULL!		
0	0	0	1	#NULL!		hispanic

0	0	0	1	#NULL!	
0	1	0	0	#NULL!	
0	1	0	0	#NULL!	
0	1	0	0	#NULL!	
0	1	0	1	#NULL!	native Irish and american.
0	1	0	0	#NULL!	
0	1	0	0	#NULL!	
0	1	0	0	#NULL!	
0	1	0	0	#NULL!	
0	1	0	0	#NULL!	
0	0	0	1	#NULL!	
0	0	0	1	#NULL!	
0	0	0	1	#NULL!	hispanic
0	1	0	0	#NULL!	
0	1	0	0	#NULL!	
0	0	0	1	#NULL!	hispanic
0	1	0	0	#NULL!	
0	0	0	1	#NULL!	hispanic
0	0	0	1	#NULL!	hispanic
0	0	0	0	#NULL!	
0	0	0	0	#NULL!	
0	0	0	1	#NULL!	hispanic
0	0	0	1	#NULL!	mexican
0	0	0	1	#NULL!	Hispanic
0	0	0	1	#NULL!	fish
0	1	0	0	#NULL!	
0	1	0	0	#NULL!	
0	1	0	0	#NULL!	
0	0	0	1	#NULL!	
0	1	0	0	#NULL!	
0	0	0	1	#NULL!	Hispanic
0	1	0	0	#NULL!	
0	1	0	0	#NULL!	
0	0	0	1	#NULL!	Hispanic
0	1	0	0	#NULL!	
0	0	0	1	#NULL!	other
0	1	0	0	#NULL!	
0	0	0	1	#NULL!	Hispanic
0	0	0	1	#NULL!	mexica
0	0	0	1	#NULL!	American
0	1	0	0	#NULL!	
0	0	0	1	#NULL!	Hispanic
0	0	0	1	#NULL!	hispanic
0	1	0	0	#NULL!	
0	1	0	0	#NULL!	
0	1	0	0	#NULL!	
0	0	0	1	#NULL!	Hispanic
0	0	0	1	#NULL!	hispanic

0	1	0	0	#NULL!	
0	0	0	1	#NULL!	Hispanic
0	1	0	0	#NULL!	
0	0	0	1	#NULL!	Hispanic
0	0	0	1	#NULL!	hispanic
0	0	0	1	#NULL!	Hispanic
0	0	0	1	#NULL!	Hispanic
0	0	0	1	#NULL!	hispanic
0	0	0	1	#NULL!	Hispanic
0	1	0	0	#NULL!	
0	1	0	0	#NULL!	
0	1	0	0	#NULL!	
0	0	0	1	#NULL!	hispanic
0	0	0	1	#NULL!	I'm not any of those
0	1	0	0	#NULL!	
0	0	0	0	#NULL!	
0	0	0	1	#NULL!	
0	1	0	0	#NULL!	
0	1	0	0	#NULL!	
0	1	0	0	#NULL!	
0	0	0	1	#NULL!	Hispanic

living_pre	living_other_pre	ace_div_pre	ace_die_pre	ace_jail_pre	ace_ill_pre	ace_drug_pre
1	#NULL!	0	0	0	0	0
1	#NULL!	0	0	0	0	0
7	#NULL!	0	0	0	0	0
2	#NULL!	0	1	1	0	99
2	#NULL!	0	0	0	0	0
1	#NULL!	99	99	0	99	1
7	#NULL!	0	0	0	0	1
1	#NULL!	0	0	0	0	1
1	#NULL!	0	0	1	0	0
5	#NULL!	0	0	1	1	1
4	#NULL!	1	0	1	0	1
1	#NULL!	0	0	1	0	0
1	#NULL!	1	0	1	0	0
1	#NULL!	0	0	0	0	0
5	#NULL!	0	0	0	0	0
2	#NULL!	1	0	1	0	0
1	#NULL!	0	0	0	0	0
4	#NULL!	1	0	99	0	99
4	#NULL!	1	99	99	99	99
4	#NULL!	0	0	1	1	0
2	#NULL!	0	1	1	99	99
1	#NULL!	99	99	99	99	99
1	#NULL!	99	99	99	99	99
2	#NULL!	0	0	0	0	0
7	#NULL!	0	0	0	0	0
1	#NULL!	0	0	0	0	0
4	#NULL!	1	0	0	0	0
4	#NULL!	1	1	0	0	1
1	#NULL!	0	0	0	0	0
4	#NULL!	1	0	1	0	0
1	#NULL!	0	0	1	0	1
2	#NULL!	1	0	1	0	1
2	#NULL!	0	0	1	1	0
1	#NULL!	0	0	0	0	0
99	#NULL!	0	0	0	99	0
1	#NULL!	0	0	0	1	0
7	#NULL!	1	0	0	1	0
1	#NULL!	0	0	0	0	0
7	#NULL!	1	0	1	1	1
2	#NULL!	1	0	1	0	1
1	#NULL!	0	99	0	0	99
1	#NULL!	0	0	0	0	0
1	#NULL!	0	0	0	0	0
2	#NULL!	1	0	0	0	0
1	#NULL!	0	0	1	0	0
1	#NULL!	1	0	0	0	0
4	#NULL!	1	0	1	1	1

1	#NULL!	0	1	0	0	0
1	#NULL!	0	0	0	1	0
1	#NULL!	0	0	1	0	0
4	#NULL!	1	0	1	0	0
1	#NULL!	0	0	1	0	1
2	#NULL!	0	0	1	0	0
1	#NULL!	0	0	1	0	1
3	#NULL!	1	1	1	1	1
2	#NULL!	0	0	0	0	0
4	#NULL!	1	0	1	0	0
1	#NULL!	0	0	0	0	0
1	#NULL!	0	0	0	0	0
1	#NULL!	0	0	0	0	0
5	#NULL!	1	0	1	1	1
2	#NULL!	1	0	1	0	0
2	#NULL!	0	0	1	0	1
2	#NULL!	1	0	0	0	1
1	#NULL!	1	0	1	0	1
4	#NULL!	1	0	0	1	1
4	#NULL!	1	0	0	0	0
4	#NULL!	1	0	1	0	0
1	#NULL!	0	0	0	0	0
4	#NULL!	1	0	0	0	1
4	#NULL!	1	0	0	0	1
1	#NULL!	0	0	0	0	0
1	#NULL!	0	1	0	0	0
1	#NULL!	1	0	0	0	1
1	#NULL!	0	0	0	0	1
1	#NULL!	0	0	0	0	0
1	#NULL!	0	0	0	0	0
4	#NULL!	1	0	0	0	0
1	#NULL!	0	0	0	0	0
2	#NULL!	1	0	1	1	0
5	#NULL!	0	0	1	0	0
4	#NULL!	0	0	0	0	0
2	#NULL!	1	0	1	0	0
1	#NULL!	0	0	0	0	0
1	#NULL!	0	0	1	1	0
1	#NULL!	0	0	0	0	0
1	#NULL!	0	0	0	0	0
1	#NULL!	0	0	0	0	0
1	#NULL!	0	0	0	0	0
4	#NULL!	1	1	1	1	1
1	#NULL!	0	0	99	0	0
1	#NULL!	0	0	0	0	0
5	#NULL!	0	0	1	0	0
4	#NULL!	1	0	1	0	1
4	#NULL!	1	0	1	1	1

1	#NULL!	0	1	0	0	0
5	#NULL!	1	0	1	0	1
2	#NULL!	0	0	1	1	0
1	#NULL!	0	0	0	0	0
1	#NULL!	0	0	0	0	0
1	#NULL!	0	0	0	0	0
2	#NULL!	1	0	1	1	1
1	#NULL!	0	0	0	0	0
1	#NULL!	0	0	0	0	0
4	#NULL!	1	1	1	1	1
1	#NULL!	1	0	1	0	0
7	#NULL!	0	0	0	1	1
4	#NULL!	1	1	1	1	1
1	#NULL!	0	0	1	0	0
1	#NULL!	0	0	0	0	0
4	#NULL!	1	0	1	0	1
1	#NULL!	0	0	0	1	1
1	#NULL!	0	0	0	0	1
1	#NULL!	0	0	0	0	0
1	#NULL!	0	0	0	0	0
2	#NULL!	1	1	1	0	1
4	#NULL!	0	0	1	0	1
1	#NULL!	1	1	1	1	1
2	#NULL!	1	0	0	0	0
1	#NULL!	1	0	0	0	0
2	#NULL!	0	0	99	0	0
1	#NULL!	0	0	99	0	1
1	#NULL!	0	0	0	0	0
1	#NULL!	0	0	0	0	0
1	#NULL!	0	0	0	0	0
1	#NULL!	0	0	0	0	0
1	#NULL!	1	0	0	0	1
1	#NULL!	0	0	0	0	1
1	#NULL!	0	0	0	0	0
4	#NULL!	0	0	0	0	0
4	#NULL!	0	0	0	0	0
2	#NULL!	1	0	0	0	0
4	#NULL!	0	0	1	0	1
1	#NULL!	0	0	0	0	0
7	#NULL!	0	0	0	0	0
2	#NULL!	1	0	0	0	0
1	#NULL!	0	0	0	0	0
2	#NULL!	1	0	0	0	0
1	#NULL!	0	0	1	0	0
2	#NULL!	0	0	1	0	0
1	#NULL!	0	0	0	0	0
5	#NULL!	0	1	1	1	1
4	#NULL!	1	0	0	0	0

4	#NULL!	99	99	1	99	99
1	#NULL!	0	0	0	0	0
7	#NULL!	0	1	1	0	0
4	#NULL!	1	0	1	99	1
7	#NULL!	0	99	1	0	1
4	#NULL!	0	0	1	0	0
1	#NULL!	0	0	1	99	0
1	#NULL!	0	0	0	0	0
1	#NULL!	0	0	0	0	0
2	#NULL!	0	0	1	0	1
1	#NULL!	0	0	0	0	0
99	#NULL!	0	0	0	0	0
1	#NULL!	0	0	0	0	0
1	#NULL!	0	0	0	0	0
2	#NULL!	0	0	0	0	0
1	#NULL!	0	0	0	0	0
1	#NULL!	0	0	0	1	1
4	#NULL!	1	0	0	0	0
2	#NULL!	1	0	1	0	0
1	#NULL!	0	0	0	0	1
2	#NULL!	1	0	0	0	0
1	#NULL!	0	0	0	0	0
5	#NULL!	1	0	1	0	1
2	#NULL!	1	0	0	0	0
1	#NULL!	0	0	0	0	0
4	#NULL!	1	0	0	0	0
1	#NULL!	1	0	1	0	0
1	#NULL!	0	0	99	0	0
3	#NULL!	1	0	0	0	0
4	#NULL!	0	0	0	0	0
1	#NULL!	0	0	0	0	0
1	#NULL!	0	0	0	0	0
1	#NULL!	1	1	1	1	1
1	#NULL!	0	0	0	0	0
5	#NULL!	0	0	1	0	1
1	#NULL!	1	1	0	1	0
7	#NULL!	1	0	0	0	0
1	#NULL!	0	0	0	0	0
3	#NULL!	1	1	0	0	0
2	#NULL!	1	0	0	0	0
1	#NULL!	0	0	1	1	1
1	#NULL!	0	0	0	0	0
3	#NULL!	1	0	0	0	0
4	#NULL!	1	0	1	1	1
1	#NULL!	0	0	0	0	0
4	#NULL!	1	0	1	0	1
6	#NULL!	0	0	1	0	1
5	#NULL!	0	1	1	0	0

2	#NULL!	1	0	0	0	0
1	#NULL!	1	0	1	0	0
1	#NULL!	1	0	1	0	1
99	#NULL!	0	0	0	0	0
4	#NULL!	1	0	0	0	0
1	#NULL!	0	0	0	0	0
2	#NULL!	1	0	0	0	0
1	#NULL!	0	0	1	1	1
1	#NULL!	0	1	0	0	0
5	#NULL!	0	0	1	1	1
4	#NULL!	1	1	1	1	1
1	#NULL!	0	0	0	0	0
2	#NULL!	1	0	1	1	1
2	#NULL!	1	1	0	1	1
1	#NULL!	0	0	0	0	0
1	#NULL!	0	0	1	1	0
4	#NULL!	1	0	0	0	1
2	#NULL!	1	1	1	0	0
1	#NULL!	0	0	0	0	0
3	#NULL!	0	0	1	0	1
1	#NULL!	0	0	0	0	1
1	#NULL!	0	0	0	0	0
2	#NULL!	1	0	0	0	0
1	#NULL!	0	0	0	0	0
5	#NULL!	1	0	1	1	1
4	#NULL!	1	1	1	1	0
1	#NULL!	0	0	1	0	1
4	#NULL!	1	0	0	0	0
1	#NULL!	0	0	0	0	0
1	#NULL!	0	0	1	0	0
1	#NULL!	0	0	0	1	0
4	#NULL!	1	0	1	1	1
1	#NULL!	0	0	1	0	0
7	#NULL!	0	1	1	1	1
4	#NULL!	0	0	1	1	1
4	#NULL!	1	0	1	1	1
5	#NULL!	0	0	0	0	0
2	#NULL!	1	1	0	0	0
1	#NULL!	0	0	0	0	0
1	#NULL!	1	1	0	0	0
1	#NULL!	99	99	99	99	99
4	#NULL!	1	0	1	0	1
4	#NULL!	1	0	1	0	1
1	#NULL!	1	0	0	0	0
4	#NULL!	1	1	1	1	1
2	#NULL!	1	0	1	0	0
1	#NULL!	0	0	0	1	0
1	#NULL!	0	0	0	0	0

6	#NULL!	1	0	1	0	1
1	#NULL!	0	0	0	0	0
1	#NULL!	0	0	0	1	0
1	#NULL!	0	0	1	0	0
1	#NULL!	0	0	0	0	1
1	#NULL!	0	0	0	0	0
1	#NULL!	0	0	0	0	0
2	#NULL!	1	0	1	1	0
1	#NULL!	0	0	0	0	0
4	#NULL!	1	0	0	0	0
1	#NULL!	0	0	0	0	0
1	#NULL!	0	0	0	0	0
1	#NULL!	0	0	0	0	0
1	#NULL!	0	0	1	0	1
1	#NULL!	0	0	0	0	0
4	#NULL!	0	0	1	1	1
2	#NULL!	1	0	1	1	1
1	#NULL!	0	0	0	0	1
4	#NULL!	1	0	1	99	1
1	#NULL!	1	0	0	0	0
1	#NULL!	0	0	0	0	0
1	#NULL!	1	0	0	99	0
1	#NULL!	0	0	1	0	0
1	#NULL!	0	1	0	0	0
5	#NULL!	1	1	0	0	1
1	#NULL!	0	0	0	0	0
1	#NULL!	0	0	0	0	0
1	#NULL!	0	0	1	0	0
7	#NULL!	0	0	1	1	1
2	#NULL!	1	0	0	0	0
3	#NULL!	1	0	0	0	0
1	#NULL!	0	0	0	0	1
4	#NULL!	1	0	1	0	0
1	#NULL!	0	0	1	0	0
2	#NULL!	1	0	0	0	0
5	#NULL!	0	0	0	0	0
1	#NULL!	0	0	0	0	0
1	#NULL!	0	0	1	0	0
1	#NULL!	0	0	0	0	0
4	#NULL!	0	0	1	0	1
4	#NULL!	1	0	1	1	1
1	#NULL!	0	0	0	0	0
1	#NULL!	0	0	0	0	1
1	#NULL!	0	0	1	0	0
1	#NULL!	0	0	0	0	0
1	#NULL!	0	0	1	0	1
4	#NULL!	1	0	99	1	0
4	#NULL!	1	0	1	1	0

1	#NULL!	0	0	0	0	0
4	#NULL!	1	0	0	0	0
5	#NULL!	0	0	1	0	1
4	#NULL!	1	0	1	0	0
4	#NULL!	0	0	0	0	1
1	#NULL!	0	0	0	0	0
4	#NULL!	1	0	1	0	1
1	#NULL!	0	0	0	0	0
2	#NULL!	0	0	1	0	0
1	#NULL!	0	0	0	0	0
7	#NULL!	0	0	0	0	0
1	#NULL!	0	0	0	0	0
1	#NULL!	0	0	0	0	0
5	#NULL!	1	0	1	0	0
1	#NULL!	1	1	0	1	0
1	#NULL!	0	0	1	0	0
1	#NULL!	0	0	0	0	0
1	#NULL!	0	0	0	0	0
4	#NULL!	1	0	0	0	0
1	#NULL!	0	0	1	0	1
1	#NULL!	0	0	0	0	0
3	#NULL!	1	0	1	1	1
7	#NULL!	0	0	1	0	1
4	#NULL!	1	0	1	99	1
2	#NULL!	0	0	0	0	1
1	#NULL!	0	0	0	0	0
4	#NULL!	1	0	1	0	1
7	#NULL!	1	0	1	0	1
5	#NULL!	1	0	1	0	1
2	#NULL!	0	0	0	0	0
1	#NULL!	1	0	1	0	0
1	#NULL!	0	1	0	0	0
5	#NULL!	0	0	1	1	1
2	#NULL!	1	0	0	0	0
1	#NULL!	0	0	0	0	0
2	#NULL!	1	0	1	1	1
1	#NULL!	0	0	1	0	0
1	#NULL!	0	0	0	0	0
1	#NULL!	0	0	1	0	0
1	#NULL!	0	0	0	0	0
1	#NULL!	0	0	0	0	0
4	#NULL!	0	0	1	0	0
5	#NULL!	0	0	1	99	0
2	#NULL!	1	0	0	1	1
1	#NULL!	0	0	0	0	0
1	#NULL!	0	0	99	0	0
3	#NULL!	1	0	1	0	0
1	#NULL!	0	0	0	0	0

1	#NULL!	0	0	0	0	0
2	#NULL!	1	0	1	0	1
1	#NULL!	0	1	1	0	0
3	#NULL!	1	0	1	1	1
1	#NULL!	0	0	0	0	0
99	#NULL!	0	0	0	0	0
4	#NULL!	1	0	1	0	1
4	#NULL!	0	0	1	0	1
4	#NULL!	1	1	1	0	1
1	#NULL!	0	0	0	0	0
2	#NULL!	0	0	1	0	0
1	#NULL!	0	0	0	0	0
2	#NULL!	1	0	1	1	1
2	#NULL!	1	0	1	1	0
4	#NULL!	1	0	0	0	1
1	#NULL!	0	0	0	0	1
1	#NULL!	0	0	1	0	1
2	#NULL!	1	0	0	0	0
1	#NULL!	0	0	0	0	0
1	#NULL!	0	0	0	0	0
1	#NULL!	0	0	0	0	0
99	#NULL!	1	0	1	1	1
2	#NULL!	1	0	0	1	0
1	#NULL!	1	0	1	0	0
99	#NULL!	0	0	1	0	0
1	#NULL!	0	0	0	0	0
1	#NULL!	0	0	0	0	0
99	#NULL!	0	0	0	0	0
1	#NULL!	0	0	0	0	0
2	#NULL!	1	0	0	0	0
1	#NULL!	0	0	0	0	0
1	#NULL!	0	0	0	0	0
1	#NULL!	0	0	0	0	0
5	#NULL!	0	0	1	0	1
2	#NULL!	0	0	99	0	0
4	#NULL!	1	0	0	0	0
1	#NULL!	0	0	0	0	0
1	#NULL!	0	0	0	1	0
1	#NULL!	0	0	0	0	0
1	#NULL!	0	0	0	0	0
1	#NULL!	1	0	1	0	1
1	#NULL!	0	0	0	0	0
1	#NULL!	0	0	0	1	1
1	#NULL!	0	0	0	1	1
1	#NULL!	0	0	0	1	0
2	#NULL!	1	0	1	0	1
1	#NULL!	0	0	0	0	0
1	#NULL!	1	1	1	0	0

1	#NULL!	0	0	0	0	0
1	#NULL!	0	0	0	0	0
4	#NULL!	1	0	0	0	0
4	#NULL!	0	0	1	0	0
1	#NULL!	0	0	1	0	0
1	#NULL!	1	0	1	99	1
1	#NULL!	0	0	0	0	0
2	#NULL!	1	1	1	1	1
1	#NULL!	1	0	99	0	0
1	#NULL!	0	0	1	0	1
1	#NULL!	0	0	0	1	0
5	#NULL!	1	0	1	1	0
1	#NULL!	0	0	0	0	0
1	#NULL!	0	0	0	0	0
1	#NULL!	0	0	0	0	0
1	#NULL!	0	0	0	0	0
5	#NULL!	1	1	1	1	1
1	#NULL!	0	0	1	1	1
1	#NULL!	0	0	0	0	0
1	#NULL!	1	0	0	0	1
1	#NULL!	0	0	0	1	0
3	#NULL!	0	0	0	0	0
99	#NULL!	1	0	0	0	1
1	#NULL!	99	0	0	0	0
4	#NULL!	1	0	0	0	0
3	#NULL!	1	0	1	1	0
1	#NULL!	0	0	0	0	1
1	#NULL!	0	0	0	0	0
7	#NULL!	1	0	0	0	0
1	#NULL!	99	99	99	99	99
1	#NULL!	0	0	0	1	0
1	#NULL!	0	1	0	0	0
2	#NULL!	1	0	0	0	0
4	#NULL!	1	99	1	1	1
2	#NULL!	1	0	0	0	0
1	#NULL!	0	0	0	0	0
4	#NULL!	1	0	1	1	99
1	#NULL!	1	0	0	1	0
1	#NULL!	0	0	0	0	0
1	#NULL!	0	0	0	0	0
4	#NULL!	1	0	0	1	0
99	#NULL!	0	1	1	1	0
1	#NULL!	0	1	0	0	0
1	#NULL!	99	99	99	99	99
7	#NULL!	0	0	1	1	1
4	#NULL!	1	0	1	1	1
4	#NULL!	0	0	1	0	1
2	#NULL!	0	0	99	0	0

1	#NULL!	0	0	0	0	0
1	#NULL!	0	0	0	99	0
1	#NULL!	0	0	0	0	0
1	#NULL!	0	0	0	0	0
1	#NULL!	1	0	0	0	0
1	#NULL!	0	0	0	1	1
4	#NULL!	1	0	1	0	0
1	#NULL!	0	0	1	0	0
1	#NULL!	1	1	0	1	1
1	#NULL!	0	0	0	0	0
2	#NULL!	1	0	1	1	1
1	#NULL!	0	0	1	0	1
2	#NULL!	1	0	1	0	0
1	#NULL!	0	1	1	0	0
1	#NULL!	0	0	1	1	0
4	#NULL!	1	0	1	0	1
1	#NULL!	0	0	0	0	0
4	#NULL!	1	0	1	0	0
2	#NULL!	1	1	1	0	1
1	#NULL!	0	1	1	0	1
1	#NULL!	0	0	0	0	0
1	#NULL!	1	1	1	1	1
1	#NULL!	0	0	1	0	1
2	#NULL!	0	0	1	0	0
1	#NULL!	0	0	1	0	1
2	#NULL!	1	0	0	0	0
6	#NULL!	1	1	1	1	1
2	#NULL!	1	0	0	1	1
2	#NULL!	1	0	0	0	0
1	#NULL!	0	1	1	1	1
1	#NULL!	0	0	0	0	0
2	#NULL!	0	0	1	0	1
1	#NULL!	0	0	0	0	0
4	#NULL!	0	0	0	0	0
4	#NULL!	1	0	0	1	1
1	#NULL!	0	0	0	0	0
4	#NULL!	1	1	1	0	1
1	#NULL!	0	0	0	0	0
1	#NULL!	0	0	0	0	0
4	#NULL!	1	0	1	0	1
1	#NULL!	0	0	0	0	1
1	#NULL!	0	0	0	0	99
3	#NULL!	0	1	0	0	0
4	#NULL!	1	0	1	0	0
1	#NULL!	0	0	0	0	0
1	#NULL!	0	0	0	0	0
1	#NULL!	0	0	0	0	0
1	#NULL!	0	0	0	1	1

7	#NULL!	0	0	0	0	0
1	#NULL!	0	0	0	0	0
1	#NULL!	0	0	0	0	0
1	#NULL!	0	0	99	0	0
5	#NULL!	1	1	1	1	1
1	#NULL!	99	1	99	1	0
1	#NULL!	0	0	0	0	99
1	#NULL!	0	0	1	0	1
4	#NULL!	0	0	1	1	1
4	#NULL!	0	0	0	0	0
4	#NULL!	0	1	0	0	0
2	#NULL!	1	0	1	0	0
1	#NULL!	0	0	0	1	0
1	#NULL!	0	0	0	0	0
1	#NULL!	0	0	0	0	0
1	#NULL!	0	0	0	0	0
3	#NULL!	0	1	0	0	0
99	#NULL!	1	1	0	0	0
2	#NULL!	1	1	0	0	0
4	#NULL!	0	1	0	0	0
1	#NULL!	0	0	0	1	0
4	#NULL!	1	0	0	0	0
1	#NULL!	0	0	0	0	0
1	#NULL!	0	0	0	1	0
99	#NULL!	99	99	99	99	99
1	#NULL!	99	0	0	0	1
1	#NULL!	0	0	0	0	0
1	#NULL!	0	0	0	0	0
4	#NULL!	1	1	1	0	0
7	#NULL!	0	0	0	0	0
4	#NULL!	0	0	1	1	1
1	#NULL!	0	0	0	0	0
4	#NULL!	1	0	1	1	0
5	#NULL!	0	0	0	0	0
2	#NULL!	0	0	0	0	0
1	#NULL!	0	0	0	0	0
1	#NULL!	0	0	1	1	1
5	#NULL!	1	0	0	0	1
1	#NULL!	0	0	0	0	0
1	#NULL!	0	0	0	0	0
2	#NULL!	1	0	0	1	1
1	#NULL!	0	0	0	0	0
1	#NULL!	0	0	0	0	0
1	#NULL!	0	0	0	0	0
4	#NULL!	0	0	1	1	1
1	#NULL!	1	0	0	0	0
2	#NULL!	0	0	0	0	0
1	#NULL!	0	0	0	0	0

1	#NULL!	0	0	0	0	0
1	#NULL!	0	0	0	1	1
1	#NULL!	0	0	0	0	0
2	#NULL!	0	0	1	0	1
4	#NULL!	1	0	1	1	1
1	#NULL!	0	0	0	0	0
4	#NULL!	1	0	0	0	0
5	#NULL!	0	0	0	0	0
1	#NULL!	1	0	0	0	0
1	#NULL!	0	0	0	0	0
1	#NULL!	0	0	1	0	1
1	#NULL!	0	0	0	0	0
4	#NULL!	1	0	1	0	1
1	#NULL!	0	0	1	0	0
2	#NULL!	1	0	0	0	1
2	#NULL!	1	0	0	0	0
1	#NULL!	0	0	0	0	0
1	#NULL!	0	0	0	0	0
1	#NULL!	0	0	0	0	0
2	#NULL!	0	0	1	0	0
1	#NULL!	0	0	0	0	0
1	#NULL!	0	0	0	0	0
3	#NULL!	0	0	0	0	1
3	#NULL!	1	0	1	0	0
4	#NULL!	1	0	1	1	1
1	#NULL!	0	0	0	0	0
1	#NULL!	0	0	0	0	0
4	#NULL!	1	0	1	0	0
1	#NULL!	0	0	1	0	1
1	#NULL!	0	0	0	0	0
2	#NULL!	1	0	1	1	1
1	#NULL!	0	0	0	0	0
4	#NULL!	1	0	1	1	1
5	#NULL!	0	0	1	0	1
1	#NULL!	0	0	0	0	0
4	#NULL!	1	1	0	1	1
1	#NULL!	0	0	0	0	0
1	#NULL!	0	0	0	0	0
7	#NULL!	99	99	99	99	99
4	#NULL!	0	1	1	0	0
1	#NULL!	0	0	0	0	0
4	#NULL!	1	1	1	0	1
1	#NULL!	1	0	1	0	0
1	#NULL!	0	0	0	0	0
1	#NULL!	0	0	1	0	0
1	#NULL!	0	0	0	0	0
2	#NULL!	1	0	0	0	0
1	#NULL!	0	0	0	0	0

4	#NULL!	0	1	0	0	0
1	#NULL!	0	0	1	0	0
2	#NULL!	1	0	1	1	1
4	#NULL!	1	0	1	1	1
4	#NULL!	0	0	0	0	0
2	#NULL!	1	0	0	0	0
1	#NULL!	0	0	0	0	0
1	#NULL!	0	0	0	0	0
1	#NULL!	0	0	0	0	0
1	#NULL!	0	0	1	0	0
1	#NULL!	1	0	1	0	0
2	#NULL!	1	1	0	0	0
4	#NULL!	1	0	0	0	0
1	#NULL!	1	0	1	0	1
99	#NULL!	0	0	1	0	1
1	#NULL!	0	0	1	0	1
4	#NULL!	1	0	1	0	1
1	#NULL!	0	0	0	0	0
4	#NULL!	1	0	1	1	0
1	#NULL!	1	0	0	0	0
6	#NULL!	0	0	1	99	0
4	#NULL!	1	0	0	0	0
2	#NULL!	0	0	0	0	0
1	#NULL!	0	0	0	0	0
1	#NULL!	1	0	1	0	0
1	#NULL!	0	0	0	0	0
3	#NULL!	0	0	1	0	1
1	#NULL!	0	0	0	0	0
2	#NULL!	0	0	1	1	1
2	#NULL!	0	0	0	0	0
99	#NULL!	99	99	99	99	0
4	#NULL!	0	0	0	0	0
1	#NULL!	0	0	0	0	0
1	#NULL!	0	0	0	0	0
1	#NULL!	0	0	0	0	0
1	#NULL!	0	0	0	0	99
1	#NULL!	0	0	0	0	0
4	#NULL!	1	0	1	0	1
1	#NULL!	1	0	1	0	0
1	#NULL!	0	0	0	0	0
1	#NULL!	0	0	0	0	0
2	#NULL!	1	0	0	0	0
1	#NULL!	0	0	1	0	0
7	#NULL!	1	0	0	0	0
3	#NULL!	1	0	1	1	0
4	#NULL!	1	0	1	0	0
1	#NULL!	0	0	0	0	0
1	#NULL!	0	1	0	0	0

1	#NULL!	0	0	0	0	0
1	#NULL!	0	0	0	0	0
1	#NULL!	0	0	0	0	0
1	#NULL!	0	0	0	0	0
1	#NULL!	0	0	1	0	0
1	#NULL!	0	0	0	0	0
1	#NULL!	0	0	0	0	0
2	#NULL!	0	1	0	1	0
2	#NULL!	1	0	0	0	0
1	#NULL!	0	0	0	0	0
1	#NULL!	0	0	0	0	0
1	#NULL!	0	0	0	0	0
2	#NULL!	1	0	0	1	0
2	#NULL!	0	99	99	1	0
2	#NULL!	1	0	0	1	0
1	#NULL!	0	0	1	0	0
3	#NULL!	0	0	99	0	1
3	#NULL!	1	0	1	0	0
2	#NULL!	0	0	1	1	1
5	#NULL!	1	1	1	1	1
4	#NULL!	1	0	1	1	0
2	#NULL!	0	0	0	0	0
5	#NULL!	0	0	0	0	0
1	#NULL!	0	0	0	0	0
4	#NULL!	0	1	1	0	1
1	#NULL!	0	0	0	0	0
4	#NULL!	0	0	1	0	0
1	#NULL!	0	0	0	0	0
4	#NULL!	1	1	0	1	1
7	#NULL!	1	0	0	0	0
1	#NULL!	0	0	99	99	99
4	#NULL!	0	0	0	0	0
1	#NULL!	0	0	0	0	0
1	#NULL!	0	0	1	0	0
1	#NULL!	0	0	0	0	0
5	#NULL!	0	0	1	0	0
1	#NULL!	0	1	0	0	0
4	#NULL!	0	0	1	0	0
1	#NULL!	0	0	0	0	0
4	#NULL!	1	1	1	1	0
7	#NULL!	1	1	1	1	1
1	#NULL!	0	0	0	0	0
5	#NULL!	0	0	1	0	1
1	#NULL!	0	0	0	0	0
1	#NULL!	0	0	1	0	1
1	#NULL!	0	0	0	0	0
1	#NULL!	0	0	0	0	0
4	#NULL!	1	0	1	99	1

1	#NULL!	0	0	0	0	0
1	#NULL!	1	0	0	0	0
4	#NULL!	1	0	0	1	0
1	#NULL!	0	0	0	0	0
1	#NULL!	0	0	0	0	0
1	#NULL!	0	0	0	1	1
2	#NULL!	1	0	1	1	0
3	#NULL!	1	0	1	1	0
1	#NULL!	0	0	0	0	0
4	#NULL!	1	0	1	0	0
1	#NULL!	0	0	1	0	1
2	#NULL!	0	1	1	1	1
2	#NULL!	0	0	1	0	0
7	#NULL!	1	0	1	0	1
1	#NULL!	0	0	0	0	0
1	#NULL!	0	0	0	0	0
1	#NULL!	0	0	0	0	0
4	#NULL!	1	0	0	1	1
2	#NULL!	1	0	0	1	1
4	#NULL!	0	0	0	0	0
2	#NULL!	1	0	1	0	0
2	#NULL!	1	0	1	0	0
1	#NULL!	0	0	1	0	1
1	#NULL!	0	0	1	0	1
4	#NULL!	1	0	1	0	0
1	#NULL!	0	0	1	0	1
4	#NULL!	1	0	1	0	1
4	#NULL!	1	0	1	0	1
4	#NULL!	0	0	1	1	0
3	#NULL!	1	0	0	0	0
1	#NULL!	0	0	0	0	0
2	#NULL!	1	0	0	0	1
4	#NULL!	0	1	0	0	0
1	#NULL!	0	0	1	0	0
1	#NULL!	0	0	0	0	0
1	#NULL!	0	0	0	0	0
1	#NULL!	0	0	0	0	0
1	#NULL!	1	1	1	0	1
1	#NULL!	1	1	0	0	1
1	#NULL!	0	0	1	0	0
1	#NULL!	0	0	0	0	0
4	#NULL!	1	0	0	0	0
5	#NULL!	0	0	1	1	0
1	#NULL!	0	0	0	0	0
1	#NULL!	0	0	0	0	0
3	#NULL!	1	0	0	1	1
1	#NULL!	0	0	0	0	0
1	#NULL!	0	0	0	0	0

1	#NULL!	0	0	1	0	0
1	#NULL!	0	0	0	0	0
4	#NULL!	1	0	0	1	1
1	#NULL!	0	0	1	1	0
1	#NULL!	0	0	1	0	0
4	#NULL!	1	1	1	0	0
7	#NULL!	0	0	1	1	1
2	#NULL!	1	0	1	1	1
1	#NULL!	0	0	0	0	0
1	#NULL!	0	0	0	0	0
1	#NULL!	0	0	0	0	0
4	#NULL!	1	0	1	0	1
5	#NULL!	0	0	1	0	0
7	#NULL!	1	0	1	1	1
4	#NULL!	1	0	1	0	1
4	#NULL!	1	0	0	0	0
1	#NULL!	0	0	1	0	1
1	#NULL!	0	0	0	0	99
4	#NULL!	1	0	0	0	1
1	#NULL!	0	0	0	0	0
4	#NULL!	0	0	0	1	0
1	#NULL!	0	0	0	0	0
1	#NULL!	1	0	1	0	1
1	#NULL!	1	0	1	0	1
1	#NULL!	0	0	0	0	0
1	#NULL!	1	0	1	0	1
1	#NULL!	0	0	0	0	0
1	#NULL!	0	0	0	0	1
1	#NULL!	0	0	0	0	0
99	#NULL!	0	0	1	1	1
7	#NULL!	1	0	1	0	0
1	#NULL!	0	0	99	1	0
4	#NULL!	1	1	1	99	99
1	#NULL!	0	0	0	0	0
1	#NULL!	0	0	1	1	1
1	#NULL!	0	0	0	0	0
4	#NULL!	0	0	0	0	0
1	#NULL!	0	0	0	0	0
1	#NULL!	0	0	0	0	0
1	#NULL!	0	0	1	0	1
2	#NULL!	1	0	1	0	1
4	#NULL!	1	0	0	0	0
2	#NULL!	1	0	1	0	0
1	#NULL!	0	0	0	0	0
7	#NULL!	0	0	0	0	1
1	#NULL!	0	0	0	0	0
1	#NULL!	0	0	0	0	0
1	#NULL!	0	0	1	1	1

1	#NULL!	0	0	0	0	0
2	#NULL!	1	0	0	0	0
1	#NULL!	0	0	0	0	0
4	#NULL!	1	0	0	0	0
4	#NULL!	1	0	1	1	0
2	#NULL!	0	0	0	0	0
1	#NULL!	0	0	0	0	0
2	#NULL!	0	0	1	1	1
1	#NULL!	0	0	0	0	0
1	#NULL!	0	0	0	0	0
1	#NULL!	1	0	0	1	1
4	#NULL!	1	0	1	0	0
1	#NULL!	0	0	99	0	0
1	#NULL!	0	0	0	0	0
2	#NULL!	1	0	1	0	1
4	#NULL!	1	0	1	1	0
1	#NULL!	0	0	1	0	1
1	#NULL!	0	0	1	0	0
4	#NULL!	1	0	0	0	1
1	#NULL!	0	0	0	0	1
1	#NULL!	0	0	0	0	0
2	#NULL!	1	0	0	0	0
1	#NULL!	0	1	0	0	0
4	#NULL!	1	0	1	0	0
5	#NULL!	0	0	0	0	0
2	#NULL!	1	0	0	0	1
3	#NULL!	1	0	1	0	1
1	#NULL!	0	0	0	0	1
2	#NULL!	0	0	0	0	0
1	#NULL!	0	0	0	0	0
4	#NULL!	0	0	1	1	0
1	#NULL!	0	0	0	0	0
1	#NULL!	0	0	0	0	1
1	#NULL!	0	0	1	1	1
4	#NULL!	1	0	1	0	0
1	#NULL!	0	0	0	0	0
4	#NULL!	1	1	1	0	1
3	#NULL!	1	0	0	0	1
1	#NULL!	1	1	1	0	0
2	#NULL!	0	1	1	0	0
4	#NULL!	1	0	0	0	1
1	#NULL!	0	0	0	0	0
3	#NULL!	1	0	0	1	1
1	#NULL!	1	1	1	0	0
1	#NULL!	1	0	1	1	1
1	#NULL!	0	0	0	0	0
1	#NULL!	0	0	0	0	0
4	#NULL!	0	0	1	0	1

2	#NULL!	0	0	0	0	0
1	#NULL!	0	0	0	0	0
2	#NULL!	1	0	1	0	0
1	#NULL!	0	0	0	1	0
1	#NULL!	0	0	0	0	0
1	#NULL!	0	1	0	0	1
1	#NULL!	0	0	0	0	0
5	#NULL!	0	0	0	0	0
1	#NULL!	0	0	0	0	0
1	#NULL!	0	0	0	0	0
1	#NULL!	0	0	0	0	0
5	#NULL!	0	0	1	0	1
1	#NULL!	1	0	1	1	1
1	#NULL!	0	1	0	0	0
1	#NULL!	0	0	0	0	0
1	#NULL!	0	0	0	1	0
4	#NULL!	0	1	1	0	1
1	#NULL!	0	0	0	0	1
1	#NULL!	0	1	0	0	1
1	#NULL!	0	0	1	0	1
1	#NULL!	0	1	0	0	0
4	#NULL!	0	0	1	0	1
4	#NULL!	1	0	0	0	1
1	#NULL!	0	0	1	0	0
4	#NULL!	1	0	0	1	1
1	#NULL!	0	0	0	0	0
1	#NULL!	0	0	0	0	0
4	#NULL!	1	0	0	0	0
2	#NULL!	0	0	1	1	0
1	#NULL!	0	0	0	0	0
2	#NULL!	0	0	0	0	0
1	#NULL!	0	0	1	1	0
1	#NULL!	0	0	0	99	0
4	#NULL!	0	0	1	0	1
4	#NULL!	1	0	1	0	1
1	#NULL!	0	0	0	0	0
1	#NULL!	0	0	1	0	0
1	#NULL!	0	1	0	0	0
4	#NULL!	1	0	1	0	0
1	#NULL!	0	0	0	1	0
1	#NULL!	0	0	99	0	0
4	#NULL!	0	0	0	0	0
2	#NULL!	0	0	1	1	1
1	#NULL!	0	0	0	0	0
4	#NULL!	1	0	1	0	1
1	#NULL!	0	0	1	0	0
6	#NULL!	0	0	1	1	1
1	#NULL!	0	0	1	0	0

5	#NULL!	0	0	1	0	1
1	#NULL!	0	1	0	1	0
1	#NULL!	0	0	0	0	0
2	#NULL!	1	0	0	0	0
3	#NULL!	1	0	1	0	1
2	#NULL!	1	0	1	0	0
1	#NULL!	0	0	0	0	0
2	#NULL!	1	0	1	0	1
1	#NULL!	0	0	0	0	0
2	#NULL!	1	0	1	1	0
1	#NULL!	0	0	1	0	0
1	#NULL!	0	0	0	0	0
1	#NULL!	0	0	0	0	0
1	#NULL!	0	0	0	0	0
4	#NULL!	1	0	0	0	1
1	#NULL!	0	0	0	0	0
1	#NULL!	1	0	1	0	0
1	#NULL!	0	0	0	0	0
1	#NULL!	0	0	0	0	1
1	#NULL!	0	0	0	0	0
4	#NULL!	1	0	0	0	0
1	#NULL!	0	0	1	0	1
1	#NULL!	0	1	0	0	0
1	#NULL!	1	0	0	0	0
3	#NULL!	0	0	1	0	0
2	#NULL!	0	1	1	0	0
1	#NULL!	0	0	0	0	0
99	#NULL!	1	1	1	1	0
1	#NULL!	0	0	0	0	0
1	#NULL!	1	0	1	0	0
5	#NULL!	0	0	1	1	1
1	#NULL!	0	0	0	1	0
1	#NULL!	0	0	0	0	0
1	#NULL!	0	0	0	0	0
2	#NULL!	1	0	1	0	1
1	#NULL!	0	0	0	1	0
1	#NULL!	0	0	0	0	0
1	#NULL!	0	0	0	0	0
4	#NULL!	0	0	0	0	0
2	#NULL!	1	0	0	0	0
1	#NULL!	0	0	0	0	0
2	#NULL!	1	1	1	1	0
1	#NULL!	0	0	1	0	1
1	#NULL!	0	0	0	0	0
1	#NULL!	0	0	0	0	0
1	#NULL!	0	0	1	1	1
2	#NULL!	1	0	1	0	0
4	#NULL!	0	0	0	0	0

7	#NULL!	1	0	1	0	1
1	#NULL!	0	0	0	0	0
1	#NULL!	0	0	0	0	0
1	#NULL!	1	0	1	0	1
99	#NULL!	99	99	99	99	99
1	#NULL!	1	0	0	1	0
2	#NULL!	1	0	1	0	0
1	#NULL!	0	0	0	0	0
7	#NULL!	1	0	0	0	0
1	#NULL!	0	0	0	0	0
7	#NULL!	1	0	0	1	0
1	#NULL!	0	0	0	0	0
2	#NULL!	1	0	1	0	1
1	#NULL!	0	1	0	0	0
1	#NULL!	0	0	1	0	1
1	#NULL!	1	0	1	0	0
2	#NULL!	1	0	0	0	0
1	#NULL!	1	0	0	1	0
1	#NULL!	0	0	0	1	0
4	#NULL!	1	1	0	0	0
1	#NULL!	0	1	1	0	0
1	#NULL!	0	0	0	0	0
1	#NULL!	0	0	0	0	0
7	#NULL!	1	1	1	0	1
4	#NULL!	1	0	0	0	0
1	#NULL!	0	0	0	0	0
1	#NULL!	0	0	0	0	0
1	#NULL!	0	0	0	0	0
5	#NULL!	0	0	1	0	1
2	#NULL!	0	0	1	0	0
1	#NULL!	0	0	0	0	1
2	#NULL!	1	0	1	1	1
1	#NULL!	0	0	1	0	0
5	#NULL!	1	0	1	0	0
1	#NULL!	0	0	0	1	0
99	#NULL!	1	0	1	0	1
4	#NULL!	1	0	0	1	1
2	#NULL!	0	1	1	0	99
1	#NULL!	0	0	0	0	0
1	#NULL!	0	0	0	1	1
4	#NULL!	1	0	1	0	0
1	#NULL!	0	0	0	0	0
4	#NULL!	1	0	0	0	0
5	#NULL!	0	0	0	0	0
2	#NULL!	1	0	0	0	0
4	#NULL!	1	99	99	99	1
1	#NULL!	0	0	0	0	0
1	#NULL!	0	0	1	0	0

4	#NULL!	0	0	1	0	0
4	#NULL!	0	0	1	0	0
1	#NULL!	0	0	0	1	1
1	#NULL!	0	0	1	0	0
1	#NULL!	0	0	1	1	0
1	#NULL!	0	1	0	0	0
2	#NULL!	0	0	1	0	1
7	#NULL!	0	0	99	0	0
5	#NULL!	1	1	0	0	0
1	#NULL!	0	0	0	0	0
1	#NULL!	1	0	0	0	0
4	#NULL!	1	1	0	1	0
4	#NULL!	1	1	1	0	1
5	#NULL!	0	0	1	0	1
1	#NULL!	1	0	1	0	0
1	#NULL!	0	0	0	0	0
1	#NULL!	0	0	0	0	0
1	#NULL!	1	0	0	99	0
1	#NULL!	0	0	0	0	0
1	#NULL!	0	0	0	0	0
1	#NULL!	0	0	0	0	0
7	#NULL!	1	99	1	99	99
1	#NULL!	0	0	0	0	0
4	#NULL!	1	1	1	1	1
1	#NULL!	1	0	0	1	0
1	#NULL!	0	0	0	0	0
1	#NULL!	1	0	1	0	0
4	#NULL!	1	0	1	0	1
1	#NULL!	0	0	1	0	1
1	#NULL!	0	0	0	0	0
1	#NULL!	0	0	0	0	0
4	#NULL!	1	0	1	0	0
1	#NULL!	0	0	1	0	0
7	#NULL!	1	1	1	0	0
1	#NULL!	0	0	0	0	0
4	#NULL!	1	0	0	0	0
1	#NULL!	0	0	0	0	0
2	#NULL!	1	0	1	1	1
1	#NULL!	0	0	0	0	0
1	#NULL!	0	0	0	0	0
4	#NULL!	0	0	0	0	0
4	#NULL!	1	0	1	0	1
2	#NULL!	1	0	0	1	0
1	#NULL!	0	0	0	0	0
1	#NULL!	0	0	0	0	0
1	#NULL!	0	0	1	0	0
1	#NULL!	0	0	0	0	1
4	#NULL!	1	0	0	0	0

1	#NULL!	0	0	0	0	0
7	#NULL!	0	0	0	0	0
1	#NULL!	0	0	0	0	0
7	#NULL!	0	1	1	1	99
1	#NULL!	0	0	0	0	0
1	#NULL!	1	0	0	0	0
1	#NULL!	0	0	0	0	0
99	#NULL!	0	0	0	0	0
1	#NULL!	0	0	0	0	0
1	#NULL!	0	0	1	1	0
2	#NULL!	1	0	99	1	0
1	#NULL!	0	0	0	0	1
1	#NULL!	0	0	0	0	0
3	#NULL!	1	1	1	1	1
5	#NULL!	1	1	1	0	0
2	#NULL!	0	0	0	1	0
1	#NULL!	0	0	0	0	0
4	#NULL!	1	0	0	0	0
99	#NULL!	0	0	0	1	1
2	#NULL!	1	0	0	0	0
1	#NULL!	0	0	0	0	0
1	#NULL!	0	0	1	0	1
2	#NULL!	0	0	0	0	0
5	#NULL!	0	0	1	0	1
2	#NULL!	1	0	1	1	1
4	#NULL!	1	0	1	1	1
1	#NULL!	0	0	0	0	0
4	#NULL!	1	0	0	0	0
2	#NULL!	1	0	1	0	0
1	#NULL!	0	0	0	0	1
5	#NULL!	1	0	0	0	1
1	#NULL!	0	0	0	0	0
1	#NULL!	0	1	0	1	0
1	#NULL!	0	0	0	0	0
1	#NULL!	0	0	0	1	0
2	#NULL!	0	0	1	0	0
4	#NULL!	1	0	1	0	1
4	#NULL!	0	0	0	0	0
1	#NULL!	0	0	0	0	1
4	#NULL!	0	0	1	0	0
1	#NULL!	0	0	0	0	0
1	#NULL!	0	0	0	0	0
4	#NULL!	1	0	0	0	0
1	#NULL!	1	0	1	0	0
2	#NULL!	0	0	99	0	0
2	#NULL!	0	0	0	0	0
4	#NULL!	0	0	0	1	0
1	#NULL!	0	0	1	0	0

2	#NULL!	1	0	1	0	1
5	#NULL!	0	0	0	0	0
4	#NULL!	1	0	0	0	0
1	#NULL!	1	0	1	0	0
4	#NULL!	1	0	0	0	0
1	#NULL!	0	0	0	1	1
1	#NULL!	1	0	1	0	1
7	#NULL!	0	0	1	0	1
7	#NULL!	1	0	1	0	1
2	#NULL!	1	0	0	0	0
1	#NULL!	0	0	0	0	0
4	#NULL!	1	0	0	0	0
1	#NULL!	0	0	1	0	1
1	#NULL!	0	0	1	0	0
3	#NULL!	1	0	0	0	0
1	#NULL!	0	0	0	0	0
1	#NULL!	0	0	0	0	1
5	#NULL!	0	0	0	0	0
1	#NULL!	0	0	0	0	0
1	#NULL!	0	0	0	0	0
7	#NULL!	1	0	1	1	1
1	#NULL!	0	0	0	0	0
1	#NULL!	0	0	0	0	0
5	#NULL!	0	0	0	0	0
4	#NULL!	0	0	1	0	0
99	#NULL!	1	0	99	0	0
1	#NULL!	0	0	0	0	0
1	#NULL!	0	0	0	0	0
1	#NULL!	0	0	0	0	0
7	#NULL!	1	0	0	0	0
1	#NULL!	0	0	0	0	0
2	#NULL!	1	0	1	0	0
1	#NULL!	0	0	1	0	0
1	#NULL!	0	0	0	0	0
1	#NULL!	0	0	0	0	1
4	#NULL!	1	0	0	0	1
1	#NULL!	0	0	0	0	0
1	#NULL!	0	1	1	1	1
1	#NULL!	0	0	0	0	0
1	#NULL!	0	0	1	1	1
1	#NULL!	0	0	0	0	0
3	#NULL!	1	1	0	1	1
2	#NULL!	99	99	99	99	99
5	#NULL!	0	0	1	0	1
1	#NULL!	0	0	0	0	0
1	#NULL!	0	0	0	0	0
1	#NULL!	0	0	0	0	0
4	#NULL!	1	1	1	1	1

1	#NULL!	0	0	1	0	1
1	#NULL!	0	0	0	0	1
1	#NULL!	0	1	1	0	0
1	#NULL!	1	0	0	1	1
4	#NULL!	1	0	1	1	0
1	#NULL!	0	0	0	0	1
2	#NULL!	0	1	0	0	0
4	#NULL!	1	0	1	0	0
1	#NULL!	0	0	0	0	0
3	#NULL!	0	1	0	0	1
7	#NULL!	0	0	0	1	0
1	#NULL!	0	0	1	0	1
4	#NULL!	1	0	1	1	1
3	#NULL!	1	0	1	0	1
1	#NULL!	0	0	0	0	0
1	#NULL!	0	0	0	0	0
5	#NULL!	1	1	1	0	1
4	#NULL!	0	0	0	0	0
7	#NULL!	1	0	1	1	1
5	#NULL!	1	0	1	0	1
2	#NULL!	0	0	0	0	0
7	#NULL!	1	0	1	1	1
1	#NULL!	0	0	0	0	1
1	#NULL!	0	0	0	0	0
7	#NULL!	1	0	1	1	1
1	#NULL!	0	0	0	0	0
1	#NULL!	0	0	0	0	0
1	#NULL!	0	0	99	0	1
99	#NULL!	1	0	1	1	0
1	#NULL!	0	0	0	0	0
1	#NULL!	0	0	0	0	0
1	#NULL!	0	0	0	0	0
5	#NULL!	0	0	0	0	0
1	#NULL!	0	0	1	0	1
2	#NULL!	1	0	1	1	1
1	#NULL!	0	0	0	0	0
7	#NULL!	1	0	0	1	1
1	#NULL!	0	0	0	0	0
4	#NULL!	1	1	1	0	1
3	#NULL!	1	1	0	0	0
1	#NULL!	0	0	0	0	0
1	#NULL!	0	0	0	0	0
2	#NULL!	1	0	0	0	0
4	#NULL!	1	0	1	1	1
2	#NULL!	1	0	1	0	0
4	#NULL!	1	0	1	0	1
1	#NULL!	0	0	1	0	0
1	#NULL!	0	0	0	0	0

1	#NULL!	0	0	1	1	1
7	#NULL!	1	0	1	0	0
4	#NULL!	1	0	1	0	1
1	#NULL!	99	99	99	99	99
1	#NULL!	0	0	1	1	0
4	#NULL!	1	0	1	1	1
1	#NULL!	0	0	0	0	0
1	#NULL!	0	0	1	0	0
4	#NULL!	0	0	0	0	0
1	#NULL!	0	1	0	0	1
1	#NULL!	0	0	1	0	1
7	#NULL!	1	1	1	0	1
1	#NULL!	0	0	0	0	1
4	#NULL!	1	0	1	0	1
1	#NULL!	99	0	1	0	0
5	#NULL!	1	1	1	0	1
5	#NULL!	0	1	0	1	0
7	#NULL!	1	0	1	0	0
7	#NULL!	0	0	0	0	0
1	#NULL!	0	0	0	0	1
99	#NULL!	1	1	0	0	0
1	#NULL!	0	0	1	0	0
2	#NULL!	1	0	0	0	0
2	#NULL!	0	0	0	1	0
4	#NULL!	1	0	1	1	1
5	#NULL!	1	0	1	1	1
1	#NULL!	0	0	0	0	0
1	#NULL!	1	0	0	0	0
3	#NULL!	0	0	1	1	1
4	#NULL!	1	0	1	0	1
2	#NULL!	1	0	1	1	0
1	#NULL!	0	0	0	0	0
2	#NULL!	1	1	1	1	1
1	#NULL!	0	0	0	0	0
4	#NULL!	1	0	1	0	1
2	#NULL!	0	0	1	1	0
2	#NULL!	1	0	1	0	0
1	#NULL!	0	0	0	0	0
1	#NULL!	0	0	0	1	0
2	#NULL!	1	1	1	1	0
1	#NULL!	0	0	0	0	0
1	#NULL!	0	0	0	0	0
1	#NULL!	0	0	0	0	0
4	#NULL!	1	1	1	1	1
1	#NULL!	0	0	0	0	0
1	#NULL!	0	99	0	99	0
1	#NULL!	0	0	0	1	0
1	#NULL!	0	0	1	0	0

4	#NULL!	1	0	1	1	1
7	#NULL!	1	0	1	1	1
2	#NULL!	1	0	0	0	0
1	#NULL!	1	0	0	0	0
5	#NULL!	0	0	1	0	1
2	#NULL!	1	0	0	0	0
1	#NULL!	0	0	1	0	0
2	#NULL!	1	0	1	0	1
7	#NULL!	1	1	1	1	1
2	#NULL!	0	0	0	0	0
1	#NULL!	0	0	1	1	1
4	#NULL!	0	0	0	0	0
1	#NULL!	0	0	1	0	0
1	#NULL!	0	0	0	0	0
1	#NULL!	0	0	0	0	0
2	#NULL!	1	0	1	0	0
4	#NULL!	1	0	1	0	0
1	#NULL!	0	0	0	0	0
2	#NULL!	1	0	1	1	1
2	#NULL!	1	0	0	1	1
7	#NULL!	0	0	0	1	1
4	#NULL!	1	0	1	0	1
1	#NULL!	1	0	0	0	1
2	#NULL!	0	0	0	0	0
2	#NULL!	1	0	1	0	0
1	#NULL!	1	0	0	0	0
4	#NULL!	0	1	0	0	0
2	#NULL!	1	0	1	0	0
1	#NULL!	0	0	0	0	0
1	#NULL!	0	0	0	0	0
1	#NULL!	0	0	0	0	0
1	#NULL!	1	0	1	0	1
4	#NULL!	99	0	1	0	0
2	#NULL!	1	0	0	0	0
2	#NULL!	1	0	1	1	0
4	#NULL!	1	0	0	0	1
1	#NULL!	0	0	0	0	0
5	#NULL!	1	1	0	1	1
1	#NULL!	0	0	0	0	0
1	#NULL!	0	1	0	1	1
2	#NULL!	1	0	1	1	1
2	#NULL!	0	0	1	1	0
1	#NULL!	0	0	0	0	0
2	#NULL!	0	0	0	0	0
1	#NULL!	0	99	0	0	0
1	#NULL!	0	0	0	0	0
5	#NULL!	0	0	1	1	1
2	#NULL!	1	0	1	0	1

1	#NULL!	0	0	0	0	0
4	#NULL!	0	0	0	0	0
1	#NULL!	0	0	0	0	0
1	#NULL!	0	0	1	1	1
1	#NULL!	0	0	0	0	0
4	#NULL!	1	0	1	1	1
6	#NULL!	1	0	1	1	1
1	#NULL!	0	0	0	0	0
1	#NULL!	0	0	0	0	0
1	#NULL!	0	0	0	0	0
1	#NULL!	0	0	1	0	0
1	#NULL!	0	0	0	0	0
1	#NULL!	1	0	0	1	0
1	#NULL!	0	0	0	1	0
1	#NULL!	0	0	0	0	0
2	#NULL!	0	0	0	0	0
1	#NULL!	0	0	0	0	0
4	#NULL!	1	1	0	1	0
1	#NULL!	1	0	0	1	1
4	#NULL!	1	0	1	0	1
4	#NULL!	1	0	1	0	1
1	#NULL!	0	0	0	0	0
1	#NULL!	0	0	0	0	0
3	#NULL!	1	0	1	0	0
2	#NULL!	1	0	1	0	0
1	#NULL!	0	0	0	1	0
2	#NULL!	1	0	1	1	0
1	#NULL!	0	0	0	1	0
1	#NULL!	0	0	0	99	0
4	#NULL!	1	0	1	0	1
1	#NULL!	0	0	0	0	0
2	#NULL!	1	0	1	0	0
4	#NULL!	0	1	1	0	99
1	#NULL!	0	0	1	0	0
1	#NULL!	0	1	1	0	1
4	#NULL!	1	0	0	0	0
2	#NULL!	0	0	0	0	0
1	#NULL!	0	0	0	0	0
2	#NULL!	0	1	0	1	0
7	#NULL!	0	0	0	0	0
1	#NULL!	0	0	0	0	0
1	#NULL!	0	0	0	0	0
1	#NULL!	0	0	0	0	0
4	#NULL!	1	0	1	0	99
2	#NULL!	0	0	0	1	0
2	#NULL!	1	0	0	0	0
2	#NULL!	99	0	0	0	0
2	#NULL!	1	0	0	0	0

1	#NULL!	0	0	0	0	0
1	#NULL!	0	0	0	0	0
1	#NULL!	0	0	0	0	0
2	#NULL!	0	0	0	0	0
1	#NULL!	0	0	0	0	0
4	#NULL!	1	0	0	0	0
1	#NULL!	99	99	99	99	99
4	#NULL!	1	0	1	0	0
1	#NULL!	0	0	0	0	0
1	#NULL!	1	0	0	0	1
1	#NULL!	0	0	0	0	0
1	#NULL!	1	0	0	0	0
5	#NULL!	1	0	0	0	0
7	#NULL!	99	0	1	0	0
1	#NULL!	0	0	0	0	0
1	#NULL!	0	0	0	0	0
1	#NULL!	0	0	1	0	0
4	#NULL!	99	99	99	99	99
2	#NULL!	0	0	0	0	0
3	#NULL!	1	1	0	0	0
1	#NULL!	0	0	0	0	0
1	#NULL!	0	1	0	0	0
2	#NULL!	1	0	1	0	0
1	#NULL!	0	0	0	0	0
1	#NULL!	0	0	0	0	0
5	#NULL!	0	0	1	99	1
1	#NULL!	0	0	0	0	0
1	#NULL!	0	0	0	0	0
4	#NULL!	1	99	1	99	99
1	#NULL!	99	0	0	99	0
4	#NULL!	0	0	0	0	0
1	#NULL!	0	0	1	0	1
4	#NULL!	1	1	1	0	1
7	#NULL!	0	1	0	0	1
4	#NULL!	1	0	0	0	0
1	#NULL!	0	0	0	0	0
1	#NULL!	0	0	0	0	0
1	#NULL!	0	0	0	0	0
6	#NULL!	1	1	1	1	1
1	#NULL!	0	0	0	0	0
1	#NULL!	0	0	0	0	0
1	#NULL!	0	0	0	0	0
1	#NULL!	0	0	0	0	0
4	#NULL!	1	0	1	0	0
1	#NULL!	0	0	0	0	1
2	#NULL!	0	0	1	0	0
1	#NULL!	0	0	0	1	0
7	#NULL!	0	0	1	0	1

1	#NULL!	0	0	0	0	0
1	#NULL!	0	0	0	0	0
1	#NULL!	0	0	0	0	0
1	#NULL!	0	0	0	0	0
2	#NULL!	1	0	1	0	0
1	#NULL!	0	0	1	0	0
1	#NULL!	0	0	0	0	0
2	#NULL!	1	0	0	0	0
1	#NULL!	0	0	1	0	0
4	#NULL!	1	1	1	1	1
2	#NULL!	1	0	1	1	1
1	#NULL!	0	0	0	0	0
2	#NULL!	1	0	0	0	0
1	#NULL!	1	0	1	0	1
1	#NULL!	0	0	0	0	0
1	#NULL!	0	0	0	0	0
4	#NULL!	1	0	1	0	1
1	#NULL!	0	0	0	0	0
4	#NULL!	0	0	1	0	0
1	#NULL!	0	0	0	0	0
1	#NULL!	0	0	0	0	0
2	#NULL!	0	0	1	0	0
1	#NULL!	0	0	0	0	0
2	#NULL!	1	0	0	0	0
2	#NULL!	0	0	0	0	0
4	#NULL!	1	0	1	0	1
1	#NULL!	0	0	0	0	0
1	#NULL!	0	0	0	0	0
1	#NULL!	0	0	0	0	0
1	#NULL!	1	0	0	0	0
2	#NULL!	1	0	0	0	0
2	#NULL!	1	0	0	0	0
5	#NULL!	0	0	0	0	0
1	#NULL!	0	0	0	0	0
7	#NULL!	1	0	1	1	1
1	#NULL!	0	0	0	0	0
1	#NULL!	0	0	0	0	0
2	#NULL!	1	0	0	0	0
1	#NULL!	0	0	0	0	0
5	#NULL!	0	0	1	0	1
1	#NULL!	1	0	0	0	0
2	#NULL!	1	0	0	0	0
1	#NULL!	0	0	1	1	1
1	#NULL!	0	0	0	0	0
1	#NULL!	0	0	0	0	0
1	#NULL!	0	0	0	0	0
1	#NULL!	0	0	0	0	0
1	#NULL!	0	0	1	0	0

1	#NULL!	0	0	0	0	0
1	#NULL!	0	0	0	0	0
1	#NULL!	0	0	99	0	1
4	#NULL!	0	0	0	0	0
2	#NULL!	0	1	0	0	0
1	#NULL!	0	0	0	0	0
2	#NULL!	1	0	1	0	0
1	#NULL!	0	0	0	0	0
1	#NULL!	0	0	0	0	0
1	#NULL!	1	0	0	1	0
1	#NULL!	0	0	0	0	0
2	#NULL!	0	0	0	0	0
2	#NULL!	1	0	0	0	0
2	#NULL!	1	0	0	1	1
1	#NULL!	0	0	0	0	0
2	#NULL!	0	1	1	0	0
5	#NULL!	1	1	1	1	1
1	#NULL!	0	0	0	0	0
1	#NULL!	0	1	1	1	0
2	#NULL!	0	0	1	0	0
1	#NULL!	0	0	0	0	0
1	#NULL!	0	0	1	0	1
1	#NULL!	0	0	1	0	0
1	#NULL!	0	0	0	0	0
2	#NULL!	1	0	99	0	0
4	#NULL!	0	0	0	0	0
1	#NULL!	0	0	0	0	0
1	#NULL!	0	0	0	0	0
5	#NULL!	0	0	1	0	1
1	#NULL!	0	0	0	0	0
2	#NULL!	1	0	1	0	0
1	#NULL!	0	0	0	0	0
1	#NULL!	1	1	1	0	0
1	#NULL!	0	0	0	0	0
4	#NULL!	1	0	0	0	0
2	#NULL!	0	0	0	1	0
1	#NULL!	0	1	0	0	0
1	#NULL!	0	0	0	0	0
5	#NULL!	1	0	0	0	0
1	#NULL!	0	0	0	0	0
2	#NULL!	1	0	1	1	1
1	#NULL!	0	0	99	0	1
1	#NULL!	0	0	0	1	0
2	#NULL!	1	0	0	0	0
4	#NULL!	0	1	0	0	0
1	#NULL!	0	0	0	0	0
1	#NULL!	0	0	1	0	1
99	#NULL!	99	99	99	99	99

1	#NULL!	0	0	1	0	1
1	#NULL!	0	0	0	0	0
1	#NULL!	0	0	0	0	0
7	#NULL!	1	0	1	1	1
4	#NULL!	1	0	0	0	0
4	#NULL!	0	1	1	0	0
1	#NULL!	0	0	0	0	0
2	#NULL!	0	0	0	1	1
1	#NULL!	0	0	0	0	0
4	#NULL!	1	0	0	0	0
2	#NULL!	1	0	0	1	1
1	#NULL!	0	0	0	0	0
1	#NULL!	0	0	0	0	0
99	#NULL!	0	0	1	0	1
1	#NULL!	0	0	1	0	1
7	#NULL!	1	0	1	0	1
2	#NULL!	0	0	0	0	0
5	#NULL!	1	0	1	1	1
1	#NULL!	1	0	1	0	1
1	#NULL!	0	0	0	0	0
6	#NULL!	1	1	1	0	1
4	#NULL!	1	0	1	1	1
1	#NULL!	0	0	0	0	0
2	#NULL!	1	1	1	1	0
7	#NULL!	0	0	1	0	0
1	#NULL!	0	0	0	0	0
1	#NULL!	0	0	1	1	1
1	#NULL!	0	0	0	0	0
4	#NULL!	0	0	0	0	0
2	#NULL!	1	0	0	0	0
4	#NULL!	1	0	0	0	0
2	#NULL!	0	0	0	0	0
1	#NULL!	0	0	0	0	0
5	#NULL!	0	1	0	0	0
1	#NULL!	0	0	0	0	0
5	#NULL!	0	0	1	0	1
1	#NULL!	0	0	0	0	0
2	#NULL!	1	0	0	0	0
1	#NULL!	0	0	0	0	0
1	#NULL!	0	0	0	0	0
1	#NULL!	0	0	1	1	1
1	#NULL!	1	0	0	0	0
4	#NULL!	0	0	0	0	0
4	#NULL!	1	0	0	0	0
2	#NULL!	1	0	0	0	1
4	#NULL!	1	0	0	0	0
1	#NULL!	1	0	1	0	0
5	#NULL!	0	0	0	0	1

1	#NULL!	0	0	0	0	0
4	#NULL!	1	0	1	0	0
4	#NULL!	1	0	0	0	0
1	#NULL!	0	0	0	0	0
1	#NULL!	0	0	0	0	1
4	#NULL!	1	0	0	0	0
2	#NULL!	1	0	0	0	1
1	#NULL!	0	0	0	0	0
5	#NULL!	1	99	1	1	0
2	#NULL!	1	0	0	0	1
4	#NULL!	1	0	0	0	0
1	#NULL!	0	0	0	0	0
1	#NULL!	0	0	0	0	0
4	#NULL!	1	0	0	0	0
1	#NULL!	0	0	0	0	0
1	#NULL!	1	0	0	0	1
1	#NULL!	0	0	0	0	0
3	#NULL!	1	0	0	0	0
4	#NULL!	0	0	1	0	1
1	#NULL!	1	0	0	0	0
1	#NULL!	1	0	99	99	0
2	#NULL!	1	0	0	0	1
4	#NULL!	1	0	99	0	0
7	#NULL!	0	0	1	0	1
4	#NULL!	1	0	1	0	0
1	#NULL!	0	0	0	0	0
1	#NULL!	0	0	1	1	1
1	#NULL!	0	0	1	1	1
1	#NULL!	0	0	0	0	1
3	#NULL!	1	0	1	0	99
2	#NULL!	1	1	0	0	0
4	#NULL!	1	0	99	1	1
1	#NULL!	1	0	0	0	0
1	#NULL!	0	0	0	0	0
4	#NULL!	0	0	0	0	0
1	#NULL!	0	0	1	1	1
1	#NULL!	0	0	0	1	0
1	#NULL!	0	0	0	0	0
1	#NULL!	0	0	0	0	0
2	#NULL!	1	0	1	0	0
2	#NULL!	1	0	0	0	0
4	#NULL!	0	0	0	0	0
1	#NULL!	0	0	0	0	0
1	#NULL!	0	0	0	0	0
1	#NULL!	0	0	0	0	0
1	#NULL!	0	0	1	0	0
1	#NULL!	0	0	0	1	0
1	#NULL!	0	0	0	0	1

1	#NULL!	0	0	0	0	0
1	#NULL!	0	0	0	0	1
2	#NULL!	1	0	0	0	1
1	#NULL!	0	0	0	0	0
1	#NULL!	0	0	0	0	0
1	#NULL!	1	0	0	0	0
1	#NULL!	0	0	0	0	0
1	#NULL!	0	0	0	0	0
1	#NULL!	0	0	1	0	0
99	#NULL!	0	0	1	1	0
2	#NULL!	1	0	1	0	0
2	#NULL!	1	0	0	0	0
1	#NULL!	0	0	99	1	0
5	#NULL!	1	1	0	0	1
1	#NULL!	1	0	1	1	1
1	#NULL!	0	0	0	0	0
1	#NULL!	0	0	0	0	0
1	#NULL!	0	0	0	1	0
1	#NULL!	0	0	0	0	0
1	#NULL!	1	0	0	0	0
1	#NULL!	0	0	0	0	1
1	#NULL!	0	0	0	0	0
6	#NULL!	0	0	1	0	1
4	#NULL!	0	1	1	1	1
1	#NULL!	0	0	1	0	0
1	#NULL!	0	0	0	0	1
4	#NULL!	1	0	0	1	0
7	#NULL!	0	0	0	0	0
5	#NULL!	0	0	0	0	0
2	#NULL!	1	0	99	0	0
1	#NULL!	0	0	0	0	0
99	#NULL!	0	0	0	0	0
2	#NULL!	1	0	1	0	1
1	#NULL!	0	0	0	0	0
7	#NULL!	1	0	1	1	0
4	#NULL!	1	0	1	0	99
1	#NULL!	0	0	0	0	0
4	#NULL!	99	99	1	1	1
3	#NULL!	0	0	0	0	0
1	#NULL!	0	0	1	0	1
5	#NULL!	0	0	1	1	1
1	#NULL!	99	99	99	99	99
1	#NULL!	0	0	1	0	1
1	#NULL!	0	0	0	0	0
4	#NULL!	1	0	1	0	1
4	#NULL!	1	1	0	1	0
4	#NULL!	0	0	1	0	0
1	#NULL!	0	0	1	0	0

1	#NULL!	0	0	0	0	1
1	#NULL!	0	0	0	0	0
4	#NULL!	1	0	0	0	0
1	#NULL!	0	0	0	0	0
1	#NULL!	0	0	1	0	0
3	#NULL!	1	0	0	0	0
4	#NULL!	1	0	1	0	1
1	#NULL!	0	0	1	0	0
5	#NULL!	1	0	0	0	0
1	#NULL!	0	0	0	0	0
7	#NULL!	1	99	99	99	99
4	#NULL!	1	0	1	0	0
7	#NULL!	0	0	1	0	0
2	#NULL!	0	0	0	0	0
3	#NULL!	1	0	1	0	0
1	#NULL!	0	0	0	0	0
2	#NULL!	1	0	0	0	0
1	#NULL!	0	0	0	0	0
1	#NULL!	0	0	0	0	0
1	#NULL!	1	0	0	1	1
5	#NULL!	1	1	1	1	1
4	#NULL!	1	0	0	0	0
1	#NULL!	0	0	0	0	0
1	#NULL!	0	0	0	0	0
1	#NULL!	0	0	0	0	1
1	#NULL!	0	0	1	1	0
1	#NULL!	99	99	99	99	99
3	#NULL!	1	1	0	0	0
1	#NULL!	0	0	0	0	0
4	#NULL!	0	0	0	0	0
1	#NULL!	0	0	1	0	0
1	#NULL!	0	0	0	0	0
2	#NULL!	1	0	0	0	0
7	#NULL!	1	0	1	0	0
5	#NULL!	1	1	1	0	1
1	#NULL!	0	0	0	0	1
5	#NULL!	0	1	1	0	0
1	#NULL!	0	0	0	0	0
4	#NULL!	1	0	0	0	0
1	#NULL!	0	0	0	0	0
2	#NULL!	1	1	1	99	0
1	#NULL!	0	0	0	0	0
2	#NULL!	1	0	0	0	0
4	#NULL!	1	1	99	99	0
1	#NULL!	0	0	0	0	0
1	#NULL!	1	1	1	1	1
5	#NULL!	0	0	0	0	0
99	#NULL!	1	1	99	1	1

1	#NULL!	0	0	0	0	0
1	#NULL!	1	0	0	0	1
4	#NULL!	1	0	1	0	0
1	#NULL!	1	0	0	0	0
1	#NULL!	0	0	0	0	0
1	#NULL!	0	0	0	0	0
1	#NULL!	0	0	0	0	0
2	#NULL!	0	0	1	0	1
1	#NULL!	0	0	0	0	0
4	#NULL!	1	0	1	0	1
2	#NULL!	0	0	1	0	1
99	#NULL!	99	0	0	99	99
2	#NULL!	0	0	1	1	1
1	#NULL!	1	0	0	0	0
1	#NULL!	0	0	0	99	0
4	#NULL!	1	1	1	0	1
1	#NULL!	0	0	0	1	1
1	#NULL!	1	0	1	0	0
7	#NULL!	1	99	0	1	0
1	#NULL!	0	0	0	0	0
1	#NULL!	0	0	0	0	0
4	#NULL!	0	1	0	0	0
2	#NULL!	1	1	0	0	0
1	#NULL!	0	0	0	0	0
1	#NULL!	0	0	0	0	0
2	#NULL!	0	0	1	0	1
1	#NULL!	0	0	0	0	0
1	#NULL!	0	0	0	0	0
1	#NULL!	0	0	0	0	0
1	#NULL!	0	0	0	1	0
1	#NULL!	1	0	99	0	0
1	#NULL!	99	99	99	99	99
2	#NULL!	1	0	0	0	0
2	#NULL!	1	0	1	1	0
1	#NULL!	0	0	0	0	0
4	#NULL!	1	0	1	0	1
1	#NULL!	0	0	0	0	0
1	#NULL!	0	0	1	0	1
1	#NULL!	0	0	0	0	0
2	#NULL!	1	1	1	1	1
3	#NULL!	1	0	0	0	0
5	#NULL!	1	1	1	1	1
2	#NULL!	1	1	1	0	0
1	#NULL!	0	0	0	0	0
1	#NULL!	0	0	0	0	0
1	#NULL!	0	1	0	1	0
4	#NULL!	0	0	0	0	0
1	#NULL!	0	0	1	0	1

1	#NULL!	0	0	0	0	0
4	#NULL!	1	1	1	1	0
2	#NULL!	1	0	0	0	0
4	#NULL!	0	0	1	0	1
2	#NULL!	1	0	0	0	0
4	#NULL!	1	0	1	0	0
1	#NULL!	0	0	1	0	0
1	#NULL!	1	0	0	0	0
5	#NULL!	1	0	1	0	0
1	#NULL!	1	0	1	1	1
4	#NULL!	1	0	1	1	0
1	#NULL!	0	0	0	0	0
7	#NULL!	1	0	1	0	1
1	#NULL!	0	0	0	0	1
1	#NULL!	0	0	1	0	0
1	#NULL!	0	0	1	1	1
4	#NULL!	1	1	1	1	1
2	#NULL!	1	1	1	1	1
4	#NULL!	0	0	1	1	0
2	#NULL!	1	0	0	0	0
1	#NULL!	0	0	0	0	0
2	#NULL!	1	0	1	0	1
1	#NULL!	0	0	0	0	0
1	#NULL!	0	0	0	0	0
1	#NULL!	99	99	99	99	1
1	#NULL!	0	0	0	0	1
2	#NULL!	1	0	0	0	0
4	#NULL!	1	0	0	0	0
1	#NULL!	0	0	0	0	0
4	#NULL!	1	0	1	1	1
1	#NULL!	0	0	0	1	0
1	#NULL!	0	0	0	0	0
2	#NULL!	0	0	1	0	1
7	#NULL!	0	0	1	0	0
1	#NULL!	0	0	0	0	0
2	#NULL!	1	0	0	0	0
5	#NULL!	1	1	1	0	1
1	#NULL!	0	0	0	0	0
1	#NULL!	1	0	99	0	0
1	#NULL!	0	0	0	0	0
1	#NULL!	0	0	1	1	0
1	#NULL!	0	0	0	0	0
2	#NULL!	0	0	1	0	0
2	#NULL!	1	0	0	0	0
1	#NULL!	0	0	0	0	0
1	#NULL!	1	0	0	0	0
1	#NULL!	0	0	0	0	0
1	#NULL!	0	0	0	0	0

3	#NULL!	1	0	0	0	0
1	#NULL!	0	0	0	0	1
1	#NULL!	99	99	99	1	99
4	#NULL!	1	0	1	1	0
3	#NULL!	1	0	0	0	1
1	#NULL!	0	0	1	1	0
1	#NULL!	0	1	99	1	99
2	#NULL!	1	1	1	1	1
1	#NULL!	0	0	0	0	0
7	#NULL!	0	0	0	0	0
1	#NULL!	0	0	1	0	0
4	#NULL!	1	0	1	0	1
4	#NULL!	1	1	1	1	0
1	#NULL!	1	0	0	0	0
1	#NULL!	0	0	0	0	0
1	#NULL!	0	0	0	99	0
1	#NULL!	0	0	0	1	1
4	#NULL!	1	0	1	0	0
1	#NULL!	0	1	99	0	0
5	#NULL!	99	99	99	99	99
3	#NULL!	1	0	0	0	0
1	#NULL!	0	0	1	0	1
1	#NULL!	1	0	1	1	1
4	#NULL!	1	0	0	1	1
1	#NULL!	0	0	0	0	0
1	#NULL!	0	0	0	0	0
4	#NULL!	0	0	0	0	0
1	#NULL!	0	0	0	0	0
4	#NULL!	0	0	0	0	0
1	#NULL!	1	0	1	0	1
5	#NULL!	0	0	0	0	0
99	#NULL!	1	0	0	0	0
4	#NULL!	1	0	1	0	1
4	#NULL!	0	0	0	0	1
4	#NULL!	0	0	0	0	0
4	#NULL!	1	0	1	0	1
2	#NULL!	1	0	1	0	0
2	#NULL!	99	0	1	0	0
1	#NULL!	0	0	0	0	0
5	#NULL!	1	1	1	1	1
1	#NULL!	0	0	0	0	0
1	#NULL!	0	99	1	99	0
4	#NULL!	1	0	0	99	1
1	#NULL!	0	0	0	0	0
1	#NULL!	0	0	1	0	1
1	#NULL!	0	0	0	0	0
2	#NULL!	1	0	1	99	1
7	#NULL!	1	0	1	0	0

1	#NULL!	0	0	0	0	0
1	#NULL!	0	0	0	0	0
4	#NULL!	0	0	0	0	0
2	#NULL!	1	1	0	0	1
1	#NULL!	0	0	0	0	0
1	#NULL!	0	0	1	0	1
1	#NULL!	0	0	1	0	1
1	#NULL!	0	1	0	0	0
1	#NULL!	0	0	1	0	99
2	#NULL!	1	0	0	0	1
2	#NULL!	1	0	0	0	1
1	#NULL!	0	0	0	0	0
3	#NULL!	1	0	1	0	0
4	#NULL!	1	0	1	0	0
1	#NULL!	0	0	0	0	0
1	#NULL!	1	0	1	1	1
1	#NULL!	0	0	0	0	0
1	#NULL!	0	0	0	1	0
1	#NULL!	0	0	0	0	0
4	#NULL!	0	0	0	0	0
1	#NULL!	0	0	0	0	0
1	#NULL!	0	0	0	0	0
1	#NULL!	0	0	0	0	0
1	#NULL!	0	0	1	0	99
1	#NULL!	0	0	0	0	0
1	#NULL!	0	0	1	0	1
3	#NULL!	1	0	0	0	0
2	#NULL!	1	0	0	0	0
2	#NULL!	0	0	0	0	0
1	#NULL!	0	0	0	0	0
1	#NULL!	0	0	0	0	0
1	#NULL!	0	0	0	0	0
5	#NULL!	0	0	1	0	1
1	#NULL!	0	0	0	0	0
5	#NULL!	1	0	1	0	1
4	#NULL!	1	0	1	0	1
1	#NULL!	0	0	0	0	0
3	#NULL!	1	0	0	0	0
1	#NULL!	0	0	0	0	1
1	#NULL!	0	0	0	0	0
1	#NULL!	1	1	0	1	0
7	#NULL!	1	1	1	1	1
4	#NULL!	1	0	0	0	0
1	#NULL!	1	0	0	0	1
1	#NULL!	0	0	0	1	0
2	#NULL!	1	0	0	0	0
1	#NULL!	0	0	99	0	0
1	#NULL!	0	0	0	0	0

1	#NULL!	1	0	1	0	1
2	#NULL!	1	0	1	0	0
4	#NULL!	0	0	0	0	0
1	#NULL!	0	0	0	0	0
1	#NULL!	0	0	0	0	0
1	#NULL!	0	0	0	0	0
1	#NULL!	0	0	0	0	0
4	#NULL!	1	0	0	0	1
1	#NULL!	0	0	1	0	0
2	#NULL!	0	0	0	0	0
1	#NULL!	0	0	0	0	0
2	#NULL!	1	1	0	0	0
1	#NULL!	1	0	0	0	0
4	#NULL!	1	0	1	0	0
1	#NULL!	1	1	0	0	0
99	#NULL!	1	0	0	0	0
1	#NULL!	0	0	0	0	0
2	#NULL!	1	0	0	1	0
2	#NULL!	0	0	1	1	99
2	#NULL!	1	0	1	0	0
1	#NULL!	0	0	0	0	0
4	#NULL!	1	0	0	0	0
4	#NULL!	1	0	1	0	1
1	#NULL!	1	1	1	1	0
4	#NULL!	0	1	0	0	0
4	#NULL!	0	0	1	0	1
1	#NULL!	0	0	1	0	0
1	#NULL!	0	0	0	1	1
1	#NULL!	0	0	0	0	0
6	#NULL!	1	0	0	0	1
5	#NULL!	0	0	1	0	1
1	#NULL!	0	0	0	0	0
3	#NULL!	0	1	0	0	0
4	#NULL!	0	1	1	0	1
2	#NULL!	1	0	1	1	1
5	#NULL!	0	0	1	0	0
2	#NULL!	1	0	0	0	0
1	#NULL!	0	0	0	0	0
1	#NULL!	0	0	0	0	0
1	#NULL!	0	0	0	0	0
1	#NULL!	0	0	1	0	1
1	#NULL!	1	0	0	0	0
1	#NULL!	0	0	0	0	0
1	#NULL!	0	0	0	0	0
1	#NULL!	0	0	0	0	0
2	#NULL!	1	0	1	0	1
1	#NULL!	0	0	0	0	0
1	#NULL!	0	0	0	1	0

1	#NULL!	1	0	0	1	0
1	#NULL!	0	0	0	0	0
2	#NULL!	0	0	0	1	1
7	#NULL!	1	0	0	0	0
1	#NULL!	0	0	0	0	0
4	#NULL!	1	0	0	1	0
1	#NULL!	0	0	0	0	0
4	#NULL!	0	0	0	1	0
3	#NULL!	1	0	1	0	0
1	#NULL!	0	0	0	0	0
4	#NULL!	1	0	1	0	0
1	#NULL!	0	0	0	0	0
2	#NULL!	0	0	0	0	0
1	#NULL!	0	0	0	0	0
2	#NULL!	1	0	0	1	0
2	#NULL!	1	0	0	0	0
5	#NULL!	0	0	0	0	0
1	#NULL!	0	0	0	0	0
1	#NULL!	0	1	0	0	0
1	#NULL!	0	0	0	0	0
1	#NULL!	0	0	0	0	0
4	#NULL!	0	0	0	0	1
1	#NULL!	0	0	0	0	0
2	#NULL!	0	1	0	0	0
6	#NULL!	0	1	1	0	1
4	#NULL!	0	0	1	0	0
4	#NULL!	1	0	1	0	1
1	#NULL!	0	0	0	0	0
1	#NULL!	0	0	1	0	0
2	#NULL!	1	0	0	1	1
1	#NULL!	0	0	0	0	0
1	#NULL!	1	0	0	0	0
4	#NULL!	1	0	0	0	0
1	#NULL!	0	0	0	0	1
1	#NULL!	1	0	0	0	0
4	#NULL!	1	0	0	0	0
2	#NULL!	0	0	0	0	0
1	#NULL!	0	0	0	0	0
1	#NULL!	0	0	0	0	0
1	#NULL!	1	0	0	0	0
1	#NULL!	0	0	0	0	0
1	#NULL!	99	99	99	99	99
4	#NULL!	0	1	0	0	0
7	#NULL!	1	0	0	0	0
2	#NULL!	0	0	0	0	0
1	#NULL!	0	0	0	0	0
1	#NULL!	0	0	1	0	0
2	#NULL!	0	0	1	0	0

1	#NULL!	0	0	0	0	0
1	#NULL!	1	0	1	1	1
1	#NULL!	0	0	1	0	1
1	#NULL!	0	0	0	0	1
1	#NULL!	1	0	0	0	0
1	#NULL!	0	0	99	0	0
2	#NULL!	1	0	1	0	1
1	#NULL!	0	0	0	0	0
1	#NULL!	0	1	1	0	1
2	#NULL!	1	1	1	0	0
1	#NULL!	0	0	0	0	0
1	#NULL!	0	0	0	0	0
1	#NULL!	1	0	1	1	0
1	#NULL!	1	0	99	0	0
1	#NULL!	0	0	0	0	0
2	#NULL!	1	0	1	0	1
2	#NULL!	1	1	0	1	1
4	#NULL!	1	0	1	0	0
4	#NULL!	0	0	0	0	0
4	#NULL!	0	0	1	99	0
1	#NULL!	0	0	0	0	0
2	#NULL!	1	0	0	1	0
99	#NULL!	1	0	0	0	0
4	#NULL!	0	0	1	0	0
4	#NULL!	1	0	0	0	0
1	#NULL!	0	0	0	0	0
1	#NULL!	0	0	0	0	1
1	#NULL!	0	0	0	0	0
1	#NULL!	0	0	1	1	0
1	#NULL!	0	0	0	0	0
1	#NULL!	0	0	0	0	0
2	#NULL!	0	0	0	0	0
1	#NULL!	0	0	0	0	0
1	#NULL!	0	0	0	0	0
1	#NULL!	0	0	0	0	0
1	#NULL!	0	0	0	0	0
2	#NULL!	1	0	0	0	0
2	#NULL!	1	0	0	0	0
1	#NULL!	0	0	0	0	0
1	#NULL!	0	0	0	0	0
1	#NULL!	0	0	0	0	0
2	#NULL!	1	0	0	0	0
7	#NULL!	0	1	1	0	0
1	#NULL!	0	0	0	0	0
1	#NULL!	1	0	0	0	0
1	#NULL!	1	0	0	0	0
2	#NULL!	1	1	1	0	0
1	#NULL!	0	0	0	0	0

2	#NULL!	1	0	0	0	1
1	#NULL!	0	0	0	1	0
1	#NULL!	0	0	0	0	0
2	#NULL!	0	0	0	0	0
1	#NULL!	0	0	0	0	0
2	#NULL!	0	0	0	0	0
1	#NULL!	0	0	1	1	0
1	#NULL!	0	0	0	0	0
4	#NULL!	1	0	0	0	0
1	#NULL!	0	0	0	0	0
2	#NULL!	0	0	0	0	0
1	#NULL!	0	0	1	0	0
3	#NULL!	1	0	0	0	0
1	#NULL!	0	0	0	0	0
1	#NULL!	0	0	0	0	0
2	#NULL!	1	0	1	1	0
4	#NULL!	1	0	0	0	0
4	#NULL!	1	0	0	0	0
2	#NULL!	1	1	0	1	1
1	#NULL!	1	0	0	1	1
1	#NULL!	0	0	0	0	0
1	#NULL!	0	0	0	0	0
1	#NULL!	0	0	0	0	0
1	#NULL!	0	0	0	0	0
2	#NULL!	0	1	0	0	0
2	#NULL!	0	1	0	0	0
1	#NULL!	0	0	1	0	0
1	#NULL!	0	0	0	0	0
4	#NULL!	1	0	0	1	1
1	#NULL!	0	0	1	99	0
1	#NULL!	1	0	0	1	1
4	#NULL!	1	1	1	1	1
2	#NULL!	1	0	0	0	0
2	#NULL!	99	0	0	0	0
4	#NULL!	0	0	99	0	0
1	#NULL!	0	0	0	0	0
1	#NULL!	0	0	0	99	0
1	#NULL!	0	0	1	0	0
3	#NULL!	0	0	1	0	1
2	#NULL!	1	0	0	0	0
1	#NULL!	0	0	1	1	0
4	#NULL!	1	0	0	0	0
1	#NULL!	0	0	0	0	0
1	#NULL!	0	1	0	0	0
99	#NULL!	0	0	1	0	0
4	#NULL!	1	0	1	0	0
1	#NULL!	0	0	0	0	0
4	#NULL!	0	0	0	0	0

1	#NULL!	0	0	0	0	0
1	#NULL!	0	0	0	99	0
5	#NULL!	99	0	1	0	1
2	#NULL!	0	0	0	0	0
2	#NULL!	1	0	99	0	0
2	#NULL!	99	0	0	0	0
1	#NULL!	0	1	0	0	0
1	#NULL!	0	0	0	0	0
1	#NULL!	0	0	0	1	0
1	#NULL!	0	0	0	0	0
1	#NULL!	0	0	0	0	0
1	#NULL!	0	0	0	0	0
5	#NULL!	1	1	0	1	1
1	#NULL!	0	0	0	0	0
1	#NULL!	0	0	0	0	0
1	#NULL!	0	0	0	0	0
2	#NULL!	1	0	0	0	0
6	#NULL!	1	0	1	0	1
1	#NULL!	1	0	0	0	0
1	#NULL!	0	0	0	0	0
1	#NULL!	0	0	0	0	0
5	#NULL!	1	0	0	0	0
2	#NULL!	1	0	1	0	99
1	#NULL!	0	0	0	0	0
1	#NULL!	99	99	1	99	1
1	#NULL!	0	0	0	0	0
99	#NULL!	0	0	0	0	0
4	#NULL!	1	0	1	0	1
1	#NULL!	0	0	0	0	0
2	#NULL!	1	0	0	0	0
2	#NULL!	1	0	0	0	0
4	#NULL!	1	0	0	0	0
4	#NULL!	0	0	0	0	0
1	#NULL!	1	0	0	0	0
1	#NULL!	0	0	1	0	1
2	#NULL!	99	0	99	1	1
7	#NULL!	1	0	0	0	0
99	#NULL!	99	99	99	99	99
4	#NULL!	1	0	1	0	0
2	#NULL!	1	0	0	0	1
1	#NULL!	0	0	0	0	0
2	#NULL!	1	0	0	0	0
1	#NULL!	0	0	0	99	0
1	#NULL!	0	0	0	0	0
1	#NULL!	1	99	1	99	1
1	#NULL!	1	0	1	0	0
5	#NULL!	1	0	1	0	1
1	#NULL!	0	0	0	0	0

1	#NULL!	0	0	0	0	0
1	#NULL!	1	0	0	0	0
1	#NULL!	0	0	0	0	0
2	#NULL!	0	0	0	0	99
1	#NULL!	0	0	0	0	0
1	#NULL!	0	0	99	0	99
1	#NULL!	0	0	99	0	0
2	#NULL!	0	0	1	0	0
3	#NULL!	0	0	0	0	0
1	#NULL!	0	1	0	0	0
1	#NULL!	0	0	0	1	1
1	#NULL!	0	0	0	0	0
1	#NULL!	0	0	0	0	0
4	#NULL!	0	1	99	0	0
1	#NULL!	0	0	0	0	0
3	#NULL!	1	0	0	0	1
1	#NULL!	0	0	0	0	0
99	#NULL!	1	0	0	0	1
2	#NULL!	1	0	1	0	1
3	#NULL!	0	0	1	1	0
1	#NULL!	0	0	1	0	1
2	#NULL!	1	0	0	0	0
3	#NULL!	1	0	0	99	99
4	#NULL!	1	0	1	0	0
1	#NULL!	0	0	1	1	1
2	#NULL!	0	0	99	0	0
1	#NULL!	0	0	0	0	0
2	#NULL!	1	0	0	0	0
4	#NULL!	1	0	1	0	1
1	#NULL!	0	0	1	0	0
1	#NULL!	0	0	1	0	1
4	#NULL!	1	0	0	0	1
1	#NULL!	0	0	0	0	1
4	#NULL!	1	99	1	0	1
1	#NULL!	0	0	0	0	0
3	#NULL!	1	1	0	0	0
1	#NULL!	0	0	0	0	0
1	#NULL!	0	0	99	99	99
5	#NULL!	0	0	0	0	0
5	#NULL!	1	0	1	0	1
2	#NULL!	1	0	0	0	0
3	#NULL!	1	0	1	0	0
1	#NULL!	0	0	0	0	0
1	#NULL!	0	0	0	0	0
2	#NULL!	0	0	0	0	0
1	#NULL!	0	1	0	0	0
1	#NULL!	0	0	0	0	0
4	#NULL!	1	1	1	1	1

5	#NULL!	1	1	1	0	1
3	#NULL!	1	0	1	0	1
2	#NULL!	1	0	1	0	0
4	#NULL!	1	0	0	0	0
4	#NULL!	1	0	1	0	1
1	#NULL!	0	0	0	0	0
1	#NULL!	0	0	0	0	0
4	#NULL!	1	0	0	1	0
1	#NULL!	1	0	0	0	0
1	#NULL!	0	0	0	0	0
1	#NULL!	0	0	0	0	0
2	#NULL!	1	0	1	0	0
2	#NULL!	1	0	1	0	0
1	#NULL!	0	0	0	0	0
4	#NULL!	0	0	1	0	0
7	#NULL!	1	0	0	0	0
1	#NULL!	0	0	0	0	0
2	#NULL!	1	0	0	0	0
1	#NULL!	0	0	0	0	0
1	#NULL!	0	0	0	0	0
2	#NULL!	99	0	0	0	1
2	#NULL!	1	0	99	0	0
1	#NULL!	0	0	1	0	0
1	#NULL!	0	0	0	0	0
6	#NULL!	1	1	0	0	0
4	#NULL!	1	1	1	0	1
1	#NULL!	0	0	0	0	0
2	#NULL!	1	0	0	0	0
1	#NULL!	99	99	99	99	99
1	#NULL!	0	0	0	0	0
5	#NULL!	1	0	1	0	0
1	#NULL!	0	0	1	0	0
1	#NULL!	0	0	0	0	0
7	#NULL!	1	0	1	0	0
1	#NULL!	0	0	0	0	0
1	#NULL!	0	0	1	0	0
1	#NULL!	1	0	0	0	0
1	#NULL!	0	0	0	0	0
2	#NULL!	1	0	0	0	0
1	#NULL!	0	0	99	0	0
1	#NULL!	0	0	0	0	0
2	#NULL!	0	0	1	1	1
1	#NULL!	0	0	0	0	0
1	#NULL!	0	0	0	0	0
1	#NULL!	0	0	0	0	0
4	#NULL!	1	0	0	0	0
1	#NULL!	0	0	0	0	0
1	#NULL!	0	0	0	0	0

1	#NULL!	0	0	0	0	1
4	#NULL!	0	1	0	0	0
1	#NULL!	0	0	0	0	0
4	#NULL!	0	1	1	0	1
4	#NULL!	1	1	0	0	0
1	#NULL!	0	0	0	0	0
1	#NULL!	0	0	0	0	0
2	#NULL!	99	0	1	0	0
2	#NULL!	99	0	0	0	0
1	#NULL!	1	99	99	99	1
4	#NULL!	1	0	1	1	1
1	#NULL!	0	0	0	0	0
5	#NULL!	1	0	0	0	0
1	#NULL!	0	0	1	0	0
1	#NULL!	0	0	0	0	0
2	#NULL!	0	0	1	1	1
1	#NULL!	0	0	0	1	0
2	#NULL!	1	0	1	0	1
4	#NULL!	1	1	1	1	1
4	#NULL!	0	1	1	0	0
7	#NULL!	1	0	1	1	1
1	#NULL!	0	0	0	0	0
1	#NULL!	0	1	1	0	1
1	#NULL!	0	0	1	0	0
2	#NULL!	0	0	0	0	0
2	#NULL!	1	0	1	0	1
2	#NULL!	0	0	0	0	0
1	#NULL!	1	99	99	99	99
1	#NULL!	0	0	0	1	0
4	#NULL!	1	0	0	99	0
7	#NULL!	0	99	99	99	99
1	#NULL!	0	0	0	0	0
5	#NULL!	1	0	1	1	1
1	#NULL!	0	0	0	0	0
1	#NULL!	1	0	0	99	0
1	#NULL!	0	0	0	0	0
1	#NULL!	0	0	1	0	0
1	#NULL!	0	0	0	0	0
1	#NULL!	0	0	0	0	0
4	#NULL!	1	0	0	0	0
1	#NULL!	0	0	0	0	0
5	#NULL!	1	0	0	0	0
2	#NULL!	1	0	0	0	0
1	#NULL!	0	0	0	0	0
1	#NULL!	0	0	0	0	0
4	#NULL!	1	0	99	0	0
1	#NULL!	0	0	0	0	0
1	#NULL!	1	0	1	0	0

1	#NULL!	0	0	0	0	0
1	#NULL!	0	0	1	0	1
1	#NULL!	0	0	0	0	0
1	#NULL!	1	0	0	0	0
1	#NULL!	0	0	0	0	0
4	#NULL!	1	0	1	1	0
1	#NULL!	1	0	0	0	0
1	#NULL!	0	0	0	0	1
1	#NULL!	0	0	1	0	0
1	#NULL!	0	0	0	0	0
4	#NULL!	1	0	0	0	1
4	#NULL!	1	0	0	0	0
2	#NULL!	1	0	99	1	0
2	#NULL!	1	0	0	0	1
4	#NULL!	0	0	0	0	0
1	#NULL!	0	0	0	0	0
4	#NULL!	0	0	0	0	0
2	#NULL!	1	0	99	0	0
2	#NULL!	99	0	0	0	0
99	#NULL!	1	0	0	0	0
2	#NULL!	1	0	1	0	1
2	#NULL!	1	0	0	0	0
1	#NULL!	1	0	1	1	1
1	#NULL!	0	0	0	0	0
5	#NULL!	1	0	1	0	1
1	#NULL!	0	0	0	0	0
1	#NULL!	0	0	0	0	0
4	#NULL!	1	1	1	1	1
5	#NULL!	1	0	0	0	0
1	#NULL!	0	0	1	0	0
2	#NULL!	1	0	0	0	0
4	#NULL!	0	0	0	0	0
1	#NULL!	0	0	0	0	0
2	#NULL!	0	0	1	0	0
4	#NULL!	1	0	0	0	0
5	#NULL!	99	99	99	99	1
3	#NULL!	0	1	0	0	0
1	#NULL!	0	0	1	0	1
1	#NULL!	0	0	0	0	0
1	#NULL!	0	0	0	0	0
1	#NULL!	0	0	1	0	0
1	#NULL!	0	0	0	0	0
1	#NULL!	1	0	0	0	0
4	#NULL!	0	0	1	0	1
1	#NULL!	0	0	1	1	0
4	#NULL!	1	0	1	1	1
1	#NULL!	0	0	0	0	0
1	#NULL!	99	99	99	99	99

2	#NULL!	1	0	0	0	0
6	#NULL!	0	0	0	0	0
4	#NULL!	1	0	1	0	1
1	#NULL!	0	0	0	0	0
4	#NULL!	1	0	1	0	1
4	#NULL!	1	0	0	1	1
4	#NULL!	0	1	1	0	0
4	#NULL!	0	1	1	1	1
4	#NULL!	1	0	1	0	1
5	#NULL!	1	0	0	0	0
1	#NULL!	0	0	0	0	0
4	#NULL!	1	0	0	0	1
4	#NULL!	1	0	1	0	0
1	#NULL!	0	0	0	0	0
7	#NULL!	1	0	0	0	1
2	#NULL!	0	0	1	0	0
2	#NULL!	1	0	1	0	0
1	#NULL!	0	0	0	0	0
1	#NULL!	0	0	99	99	99
5	#NULL!	0	1	0	0	0
2	#NULL!	0	0	1	1	1
2	#NULL!	1	0	0	1	0
1	#NULL!	0	0	0	0	0
2	#NULL!	0	0	0	0	0
1	#NULL!	0	0	0	0	0
2	#NULL!	0	0	1	0	0
4	#NULL!	0	1	0	0	1
1	#NULL!	0	0	0	0	0
4	#NULL!	99	0	1	99	99
1	#NULL!	1	1	1	0	1
4	#NULL!	1	1	99	0	0
1	#NULL!	0	0	0	0	0
1	#NULL!	0	0	0	0	0
1	#NULL!	0	0	0	0	0
2	#NULL!	0	0	0	0	0
1	#NULL!	0	0	0	0	0
1	#NULL!	0	0	0	0	1
4	#NULL!	0	1	1	0	0
1	#NULL!	0	1	0	0	0
1	#NULL!	0	0	0	0	1
2	#NULL!	0	0	0	0	0
1	#NULL!	0	0	0	0	0
4	#NULL!	0	0	1	0	1
2	#NULL!	0	0	0	0	1
2	#NULL!	1	0	0	1	1
1	#NULL!	0	0	1	0	1
1	#NULL!	0	0	0	0	0
1	#NULL!	0	0	0	1	1

1	#NULL!	0	0	0	0	0
1	#NULL!	0	0	0	0	0
2	#NULL!	99	0	1	0	0
1	#NULL!	0	0	0	0	0
1	#NULL!	0	0	0	0	0
1	#NULL!	0	0	0	99	0
4	#NULL!	1	0	0	0	1
1	#NULL!	1	0	0	0	0
2	#NULL!	1	0	1	0	0
1	#NULL!	0	0	1	0	0
1	#NULL!	0	0	0	0	0
7	#NULL!	0	0	1	0	1
1	#NULL!	0	0	0	0	0
2	#NULL!	1	0	1	0	1
1	#NULL!	0	0	0	0	0
1	#NULL!	0	0	0	0	0
5	#NULL!	1	0	1	0	1
1	#NULL!	0	0	99	0	0
1	#NULL!	99	99	99	99	99
4	#NULL!	0	0	1	0	1
4	#NULL!	1	0	0	0	1
1	#NULL!	0	0	0	0	0
2	#NULL!	0	0	1	1	0
1	#NULL!	0	0	0	0	0
1	#NULL!	1	0	1	0	0
2	#NULL!	0	0	1	0	1
1	#NULL!	0	0	0	0	0
1	#NULL!	0	0	1	0	0
3	#NULL!	1	0	0	0	99
4	#NULL!	1	1	0	1	1
1	#NULL!	0	0	0	1	0
99	#NULL!	99	99	99	99	99
1	#NULL!	0	0	0	0	1
1	#NULL!	0	1	0	0	0
4	#NULL!	0	0	0	0	0
1	#NULL!	0	0	1	0	0
1	#NULL!	0	0	0	0	0
2	#NULL!	1	0	0	0	0
1	#NULL!	0	0	1	0	1
4	#NULL!	1	0	0	0	0
1	#NULL!	1	0	0	0	0
1	#NULL!	0	0	1	0	1
4	#NULL!	0	0	1	0	1
1	#NULL!	0	0	0	0	0
3	#NULL!	1	0	1	0	1
1	#NULL!	0	0	1	0	0
2	#NULL!	1	0	1	0	1
1	#NULL!	0	0	0	1	0

1	#NULL!	0	0	0	0	0
1	#NULL!	0	0	0	0	0
2	#NULL!	1	0	1	0	0
4	#NULL!	0	0	0	0	0
2	#NULL!	1	0	1	1	1
4	#NULL!	1	0	1	0	1
5	#NULL!	0	0	0	0	0
2	#NULL!	1	0	99	0	0
4	#NULL!	0	0	0	0	0
4	#NULL!	1	0	0	0	0
4	#NULL!	1	0	0	0	1
4	#NULL!	1	0	1	0	0
1	#NULL!	1	0	0	0	0
1	#NULL!	0	0	0	0	0
1	#NULL!	0	0	0	0	0
7	#NULL!	0	0	0	0	0
1	#NULL!	0	0	0	0	0
99	#NULL!	99	0	1	0	0
1	#NULL!	0	0	0	0	0
1	#NULL!	0	0	0	0	0
1	#NULL!	0	0	1	0	0
1	#NULL!	1	1	1	1	1
1	#NULL!	0	0	0	0	0
1	#NULL!	99	99	99	99	99
5	#NULL!	1	0	1	0	0
1	#NULL!	0	0	0	0	0
1	#NULL!	1	0	0	0	0
1	#NULL!	0	0	1	0	0
1	#NULL!	0	0	0	0	0
2	#NULL!	1	0	0	0	0
1	#NULL!	0	0	1	0	1
1	#NULL!	0	0	0	0	0
5	#NULL!	0	0	0	0	0
4	#NULL!	1	0	1	0	0
4	#NULL!	1	0	1	0	0
1	#NULL!	0	0	0	0	0
2	#NULL!	1	0	1	0	0
7	#NULL!	0	0	1	0	0
1	#NULL!	0	0	1	0	1
5	#NULL!	1	0	0	0	0
1	#NULL!	0	0	0	0	0
1	#NULL!	0	0	0	0	0
1	#NULL!	0	0	0	0	0
4	#NULL!	1	0	0	0	1
1	#NULL!	0	0	0	0	0
1	#NULL!	0	0	0	0	0
4	#NULL!	1	0	0	0	0
7	#NULL!	0	1	1	0	0

1	#NULL!	0	0	1	0	0
1	#NULL!	0	0	0	0	0
1	#NULL!	1	0	0	0	0
1	#NULL!	0	0	0	0	0
1	#NULL!	0	0	0	0	0
1	#NULL!	0	0	0	0	0
1	#NULL!	0	0	0	0	0
99	#NULL!	0	0	1	1	0
1	#NULL!	0	0	0	0	0
1	#NULL!	0	0	0	0	0
4	#NULL!	1	0	0	1	0
1	#NULL!	0	0	0	0	0
1	#NULL!	0	0	99	0	0
1	#NULL!	0	0	0	0	0
7	#NULL!	0	0	99	1	0
1	#NULL!	0	0	0	0	0
2	#NULL!	1	0	0	1	0
1	#NULL!	0	0	0	0	0
2	#NULL!	0	0	0	0	1
4	#NULL!	1	0	0	1	1
1	#NULL!	0	0	0	0	0
1	#NULL!	0	0	0	0	0
5	#NULL!	0	0	0	0	0
1	#NULL!	0	0	0	0	0
2	#NULL!	1	0	0	0	0
1	#NULL!	0	0	0	0	0
4	#NULL!	1	0	0	0	1
2	#NULL!	1	0	0	0	0
2	#NULL!	0	0	1	0	1
1	#NULL!	0	0	0	0	0
1	#NULL!	0	0	1	0	0
2	#NULL!	0	0	0	0	1
2	#NULL!	0	0	0	0	0
1	#NULL!	0	0	1	0	0
1	#NULL!	0	0	0	0	0
4	#NULL!	1	0	1	0	0
5	#NULL!	0	0	0	0	0
1	#NULL!	0	0	0	0	0
1	#NULL!	1	0	0	0	0
1	#NULL!	0	0	1	0	1
1	#NULL!	0	1	0	0	1
1	#NULL!	1	99	99	99	99
1	#NULL!	0	0	0	0	0
2	#NULL!	0	0	1	1	0
4	#NULL!	99	99	99	99	99
1	#NULL!	1	1	1	1	1
4	#NULL!	1	0	1	0	0
4	#NULL!	1	1	0	0	0

1	#NULL!	0	0	1	0	0
1	#NULL!	0	0	1	0	0
2	#NULL!	0	0	1	0	0
99	#NULL!	0	1	1	0	0
4	#NULL!	1	0	0	0	0
1	#NULL!	0	0	0	0	0
1	#NULL!	0	0	0	0	0
1	#NULL!	0	0	0	0	0
7	#NULL!	0	1	0	0	0
4	#NULL!	1	0	1	0	0
2	#NULL!	99	99	0	99	99
1	#NULL!	1	0	1	0	1
1	#NULL!	0	1	0	0	0
1	#NULL!	0	0	0	0	0
1	#NULL!	0	0	0	0	0
4	#NULL!	1	0	0	0	0
1	#NULL!	0	0	0	0	0
4	#NULL!	1	0	1	0	1
3	#NULL!	1	1	1	0	1
4	#NULL!	1	0	0	0	0
2	#NULL!	1	0	0	0	0
1	#NULL!	0	0	0	0	0
1	#NULL!	0	0	0	0	0
1	#NULL!	0	0	0	0	0
1	#NULL!	1	0	0	0	0
1	#NULL!	0	0	0	0	0
1	#NULL!	1	0	0	0	0
1	#NULL!	0	0	1	0	1
2	#NULL!	1	0	0	0	0
5	#NULL!	1	1	0	1	1
1	#NULL!	1	0	0	0	0
1	#NULL!	0	0	0	0	0
4	#NULL!	0	0	1	1	1
1	#NULL!	0	0	0	0	1
7	#NULL!	1	99	99	99	99
4	#NULL!	0	0	0	1	0
1	#NULL!	0	0	0	0	1
4	#NULL!	1	0	0	0	1
2	#NULL!	1	0	0	0	0
5	#NULL!	0	1	1	0	1
1	#NULL!	0	0	0	0	0
4	#NULL!	0	0	1	0	1
1	#NULL!	1	0	0	1	1
1	#NULL!	0	0	0	0	0
1	#NULL!	0	0	1	0	1
1	#NULL!	0	0	0	0	0
4	#NULL!	0	0	0	0	0
1	#NULL!	0	0	0	0	0

1	#NULL!	1	0	0	0	0
4	#NULL!	1	0	0	1	1
1	#NULL!	0	0	0	0	0
4	#NULL!	1	0	0	0	0
1	#NULL!	0	0	0	0	0
2	#NULL!	1	0	0	0	0
5	#NULL!	0	1	1	0	1
7	#NULL!	1	0	0	0	0
1	#NULL!	0	0	0	1	0
4	#NULL!	0	0	1	0	0
4	#NULL!	0	0	0	1	0
1	#NULL!	0	0	1	0	0
1	#NULL!	0	0	1	0	0
1	#NULL!	0	0	0	0	0
2	#NULL!	1	0	0	0	0
2	#NULL!	0	0	0	0	0
1	#NULL!	0	0	0	0	0
2	#NULL!	0	1	0	0	0
4	#NULL!	1	0	0	1	0
1	#NULL!	0	0	0	0	0
4	#NULL!	1	0	0	0	1
1	#NULL!	0	0	0	1	0
1	#NULL!	0	0	1	0	0
1	#NULL!	0	0	0	0	0
1	#NULL!	0	0	0	0	0
4	#NULL!	0	0	0	0	0
2	#NULL!	1	0	0	0	0
1	#NULL!	1	99	99	99	99
1	#NULL!	0	0	0	1	0
1	#NULL!	0	0	0	0	0
4	#NULL!	1	0	1	1	1
1	#NULL!	0	0	0	0	0
1	#NULL!	0	0	0	0	0
5	#NULL!	1	0	0	99	1
2	#NULL!	1	0	0	0	0
1	#NULL!	0	0	0	0	0
1	#NULL!	1	0	0	0	0
4	#NULL!	1	0	0	0	0
1	#NULL!	0	0	1	0	0
1	#NULL!	0	0	0	0	0
4	#NULL!	0	1	99	1	99
1	#NULL!	0	0	0	0	0
3	#NULL!	1	0	99	1	0
4	#NULL!	0	0	0	0	0
1	#NULL!	0	0	0	0	0
2	#NULL!	0	0	1	0	0
5	#NULL!	0	0	1	0	1
1	#NULL!	99	0	99	0	1

1	#NULL!	0	0	1	0	0
2	#NULL!	1	0	0	1	0
1	#NULL!	0	0	1	1	0
1	#NULL!	0	0	99	99	0
7	#NULL!	0	0	0	0	0
1	#NULL!	0	0	0	1	1
1	#NULL!	0	0	0	0	0
1	#NULL!	0	0	1	0	0
4	#NULL!	0	0	1	0	0
1	#NULL!	1	0	0	0	0
2	#NULL!	0	0	0	0	0
5	#NULL!	1	99	1	0	1
4	#NULL!	1	0	1	0	1
1	#NULL!	0	0	0	0	0
1	#NULL!	0	0	0	0	0
2	#NULL!	1	0	1	0	0
2	#NULL!	1	1	1	1	1
1	#NULL!	0	0	0	0	0
2	#NULL!	1	0	0	99	0
1	#NULL!	0	0	0	0	0
1	#NULL!	1	0	0	0	0
7	#NULL!	1	0	0	0	0
1	#NULL!	0	0	0	0	0
2	#NULL!	1	0	1	0	1
1	#NULL!	0	0	0	0	0
1	#NULL!	1	0	1	1	1
4	#NULL!	1	0	0	0	0
1	#NULL!	0	0	0	0	0
1	#NULL!	0	0	0	0	0
2	#NULL!	1	0	1	0	0
1	#NULL!	0	0	0	0	1
2	#NULL!	1	0	0	0	0
1	#NULL!	1	0	1	0	0
3	#NULL!	1	0	1	1	1
1	#NULL!	1	0	0	0	0
2	#NULL!	1	0	0	1	1
1	#NULL!	0	0	0	0	0
3	#NULL!	1	0	0	0	0
4	#NULL!	1	0	0	0	0
1	#NULL!	0	0	0	0	0
1	#NULL!	0	1	1	0	1
4	#NULL!	0	0	1	0	1
1	#NULL!	0	0	0	0	0
1	#NULL!	0	0	0	0	0
2	#NULL!	0	0	1	1	1
4	#NULL!	1	99	0	0	0
1	#NULL!	1	0	0	0	0
1	#NULL!	0	0	0	0	0

4	#NULL!	0	0	0	1	0
1	#NULL!	1	0	0	1	0
1	#NULL!	0	0	0	0	0
1	#NULL!	0	0	0	0	0
1	#NULL!	0	0	1	0	1
1	#NULL!	0	0	1	0	0
1	#NULL!	0	0	0	0	0
4	#NULL!	1	0	1	1	1
4	#NULL!	1	0	1	1	1
4	#NULL!	1	0	1	1	1
1	#NULL!	0	0	0	0	0
4	#NULL!	1	0	1	1	1
1	#NULL!	0	0	0	0	0
1	#NULL!	0	0	0	0	0
1	#NULL!	0	0	0	0	0
4	#NULL!	1	0	0	1	1
1	#NULL!	0	0	0	0	0
1	#NULL!	0	0	0	0	0
1	#NULL!	1	0	0	0	0
4	#NULL!	0	0	1	0	1
4	#NULL!	1	1	1	1	0
1	#NULL!	1	0	0	0	0
1	#NULL!	0	0	0	0	1
1	#NULL!	0	0	0	0	0
1	#NULL!	0	0	0	0	0
1	#NULL!	0	0	0	0	0
2	#NULL!	0	0	1	0	0
1	#NULL!	0	0	0	0	0
5	#NULL!	0	0	0	1	0
2	#NULL!	1	0	1	0	0
1	#NULL!	0	0	0	0	0
1	#NULL!	0	0	0	0	0
4	#NULL!	1	0	0	0	0
4	#NULL!	1	0	0	1	0
4	#NULL!	1	0	0	0	0
5	#NULL!	1	0	1	0	1
1	#NULL!	0	0	0	0	0
1	#NULL!	0	0	1	0	0
4	#NULL!	0	0	1	1	1
1	#NULL!	0	0	0	0	0
4	#NULL!	1	0	0	0	0
1	#NULL!	0	0	0	0	0
1	#NULL!	0	0	0	1	0
1	#NULL!	0	0	0	0	0
7	#NULL!	0	0	0	1	1
5	#NULL!	0	0	0	1	0
1	#NULL!	0	0	0	1	0
4	#NULL!	1	0	0	0	0

1	#NULL!	0	0	0	0	0
5	#NULL!	0	0	1	0	1
1	#NULL!	0	0	0	1	0
1	#NULL!	1	0	0	0	0
5	#NULL!	0	0	0	0	0
1	#NULL!	1	0	0	0	0
4	#NULL!	0	0	0	1	0
1	#NULL!	1	0	0	0	0
1	#NULL!	0	0	0	0	1
2	#NULL!	1	0	1	0	1
7	#NULL!	0	0	0	0	0
5	#NULL!	1	1	1	1	1
2	#NULL!	0	0	1	0	0
2	#NULL!	0	0	1	1	0
1	#NULL!	0	0	0	0	0
1	#NULL!	0	0	0	0	0
1	#NULL!	0	0	0	0	0
1	#NULL!	0	0	0	0	0
1	#NULL!	0	0	0	0	0
1	#NULL!	0	0	0	0	0
1	#NULL!	0	0	0	0	0
1	#NULL!	0	0	0	0	0
1	#NULL!	0	0	0	0	0
2	#NULL!	0	0	0	0	0
7	#NULL!	1	0	0	0	0
1	#NULL!	0	0	0	0	0
2	#NULL!	1	0	0	0	1
1	#NULL!	1	1	0	0	1
1	#NULL!	0	0	0	0	0
1	#NULL!	0	0	0	0	0
1	#NULL!	0	0	0	0	0
5	#NULL!	1	0	0	0	0
2	#NULL!	0	0	1	0	0
1	#NULL!	0	0	0	0	0
5	#NULL!	0	0	0	0	0
2	#NULL!	0	0	0	0	1
2	#NULL!	1	0	1	0	0
4	#NULL!	1	0	0	0	0
1	#NULL!	0	0	0	0	0
1	#NULL!	0	0	0	0	0
2	#NULL!	1	0	0	0	0
7	#NULL!	1	0	0	0	0
1	#NULL!	0	0	0	0	0
1	#NULL!	1	0	0	0	0
4	#NULL!	1	0	1	0	1
3	#NULL!	1	0	1	1	1
2	#NULL!	0	0	1	0	0
1	#NULL!	0	1	0	0	0

4	#NULL!	1	0	1	1	1
1	#NULL!	0	0	1	0	0
4	#NULL!	1	0	0	1	0
1	#NULL!	0	0	0	0	0
2	#NULL!	1	0	1	0	0
1	#NULL!	0	0	0	1	0
3	#NULL!	0	0	0	0	0
2	#NULL!	1	0	0	0	0
1	#NULL!	0	0	0	0	0
2	#NULL!	0	0	1	0	0
1	#NULL!	0	0	0	0	0
1	#NULL!	0	0	0	0	0
1	#NULL!	0	0	0	0	0
1	#NULL!	0	0	0	0	0
1	#NULL!	0	0	1	1	0
1	#NULL!	0	0	0	0	0
1	#NULL!	0	#NULL!	#NULL!	#NULL!	#NULL!
2	#NULL!	1	0	1	1	0
2	#NULL!	0	0	1	1	1
2	#NULL!	1	0	0	0	0
1	#NULL!	0	0	0	0	0

ace_home_pre	ace_neighbor_pre	ace_econ_pre	language_pre___1	language_pre___2	language_pre___3
0	0	0	0	1	0
0	0	0	0	1	0
0	0	0	1	1	0
99	0	1	1	0	0
0	0	0	1	0	0
99	99	99	0	1	0
0	1	0	1	1	0
1	0	0	1	1	0
0	0	0	0	1	0
1	0	0	1	0	0
1	1	0	1	0	0
1	0	0	1	0	0
0	0	1	1	0	0
0	0	0	1	0	0
0	0	0	1	0	0
0	0	0	0	1	0
0	0	0	1	1	0
1	0	0	1	0	0
99	99	99	1	0	0
1	1	1	1	0	0
0	0	99	1	1	0
99	99	99	0	0	0
99	99	99	0	1	0
0	1	0	1	1	0
0	0	0	1	1	0
0	0	0	1	1	0
0	0	0	1	1	0
0	0	1	1	0	0
0	0	0	1	1	0
1	0	1	1	1	0
0	0	1	0	0	0
0	0	0	1	0	0
0	1	1	1	1	0
0	0	0	1	1	0
99	0	0	1	1	0
0	0	1	1	1	0
1	0	0	0	1	0
0	0	0	1	1	0
1	0	1	1	0	0
0	0	0	1	0	0
0	1	99	1	1	0
0	1	0	1	0	0
0	0	0	1	0	0
0	0	1	1	0	0
0	0	0	1	0	0
0	1	0	1	0	0
0	0	0	1	0	0

0	0	0	1	1	0
0	0	0	1	0	0
0	0	0	1	1	0
0	0	0	1	0	0
0	0	0	0	1	0
0	0	1	1	0	0
0	0	0	1	0	0
1	1	0	1	1	0
0	0	1	0	1	0
0	0	0	1	0	0
0	0	0	0	1	0
0	0	0	1	0	0
1	0	0	1	1	0
1	0	1	1	0	0
0	99	0	1	0	0
1	0	1	1	0	0
0	1	1	1	0	0
1	0	0	1	1	0
1	0	1	1	0	0
0	0	0	1	0	0
0	0	0	1	1	0
0	0	0	1	1	0
1	0	0	1	0	0
0	0	0	1	0	0
0	0	0	1	0	0
0	0	0	1	1	0
0	0	0	1	0	0
0	0	0	1	1	0
0	0	0	1	1	0
0	0	0	0	1	0
0	0	0	1	0	0
0	0	0	0	1	0
0	0	0	1	0	0
0	0	0	1	0	0
0	0	0	1	1	0
0	0	1	0	1	0
0	0	0	1	1	0
1	0	1	1	1	0
0	1	1	1	1	0
0	0	0	1	0	0
0	0	0	1	1	0
0	0	0	1	1	0
1	99	0	1	1	0
0	0	0	1	1	0
0	0	0	1	1	0
0	1	0	1	0	0
0	0	0	1	0	0
1	99	0	1	1	0

0	1	1	1	0	0
1	1	0	0	1	0
0	0	0	0	1	0
0	0	0	1	0	1
0	0	1	0	1	0
0	0	0	1	0	0
0	0	0	1	1	0
0	0	0	1	0	0
0	0	0	0	1	0
1	1	1	1	0	0
0	0	0	1	0	0
0	1	1	0	0	0
0	1	1	1	0	0
0	0	0	1	0	0
0	0	0	1	1	0
0	0	0	1	1	0
0	0	1	1	0	0
1	0	0	1	1	0
0	0	0	1	1	0
0	0	0	1	1	0
1	0	0	0	1	0
1	0	0	1	0	0
1	1	1	0	0	0
1	0	1	1	0	0
0	0	0	0	1	0
0	1	0	0	1	0
1	0	0	1	1	0
0	0	0	1	0	0
0	0	0	1	1	0
0	0	0	1	1	0
0	0	0	1	1	0
1	1	99	1	0	0
1	0	0	1	1	0
0	0	0	1	0	0
0	0	0	1	1	0
0	0	1	0	1	0
0	0	1	1	1	0
0	0	1	1	1	0
0	0	0	1	0	1
0	0	0	1	0	0
0	0	0	1	0	0
0	1	0	1	0	0
0	0	0	1	1	0
0	0	0	1	0	0
0	0	0	1	0	0
0	0	0	1	0	0
1	1	1	1	0	0
1	1	0	1	1	0

99	99	99	1	0	0
0	0	0	1	1	0
1	0	1	1	0	0
0	0	1	1	0	0
0	0	1	1	0	0
0	1	0	1	0	0
99	0	0	0	1	0
0	0	0	1	1	0
0	0	0	0	1	0
0	0	0	1	0	0
0	0	0	1	1	0
0	0	0	0	0	0
0	0	0	1	1	0
0	0	0	1	0	0
0	0	1	0	1	0
0	0	0	1	1	0
1	1	0	1	0	0
0	0	0	1	1	0
0	0	0	0	1	0
0	0	0	0	1	0
0	99	1	0	1	0
0	0	0	1	1	0
1	1	1	1	0	0
0	0	0	1	0	0
0	0	0	1	1	0
0	0	0	1	0	0
1	0	0	1	0	0
0	0	0	1	0	0
0	0	0	1	0	0
0	0	0	1	0	0
0	0	0	0	1	0
0	0	0	1	0	0
1	1	1	1	1	0
0	0	0	1	1	0
1	1	0	1	0	0
1	0	1	1	0	0
0	0	0	1	0	0
1	0	0	1	1	0
0	0	0	1	0	0
0	0	0	1	0	0
0	0	99	1	1	0
0	0	1	1	1	0
0	0	1	1	0	0
0	0	0	1	0	0
0	0	1	1	0	0
1	1	1	1	0	0
0	0	0	1	0	0
0	0	1	1	0	0

1	0	99	1	1	0
0	0	1	1	0	0
0	1	99	1	0	0
0	0	0	1	1	0
1	1	0	1	0	0
0	1	0	1	0	0
0	0	0	1	0	0
1	1	0	1	0	0
0	0	0	0	1	0
0	0	0	1	0	0
0	0	0	1	1	0
0	0	0	0	1	0
1	0	1	1	0	0
1	0	0	1	0	0
0	0	0	1	1	0
1	0	1	1	0	0
0	0	0	1	0	0
1	1	1	1	0	0
0	1	0	1	0	0
1	0	0	0	1	0
1	1	0	1	0	0
0	0	0	1	1	0
0	0	0	1	1	0
0	0	0	1	1	0
1	1	1	1	0	0
0	0	0	1	0	0
1	0	1	1	0	0
1	0	0	1	1	0
0	1	0	0	1	0
1	1	0	1	0	0
0	0	1	1	1	0
0	0	1	1	1	0
0	0	0	1	1	0
0	0	99	1	1	0
0	0	0	1	0	0
1	1	1	1	0	0
0	0	0	1	1	0
0	0	0	0	1	0
0	0	0	0	1	0
0	0	1	0	1	0
1	1	99	1	0	0
0	1	0	1	0	0
0	0	0	1	0	0
1	0	0	1	1	0
0	1	0	1	0	0
0	0	0	1	0	0
0	0	0	1	0	0
0	0	0	1	1	0

0	0	1	1	0	0
0	0	0	1	1	0
0	0	0	1	0	0
0	1	0	1	1	0
1	0	1	1	0	0
0	0	0	1	1	0
0	1	1	1	0	0
1	0	1	1	1	0
0	1	1	1	0	0
0	0	0	1	1	0
0	0	0	1	0	0
0	0	0	0	1	0
1	0	0	1	1	0
1	0	0	1	1	0
0	0	0	0	1	0
1	1	1	1	0	0
1	1	1	1	1	0
1	1	0	1	0	0
1	1	1	1	0	0
0	1	0	1	0	0
0	0	0	1	1	0
99	99	99	1	0	0
0	0	0	0	1	0
0	1	1	1	0	0
0	0	1	1	0	0
0	0	1	1	0	0
0	0	0	1	0	0
0	0	0	1	1	0
1	0	1	1	0	0
0	0	1	1	1	0
0	0	0	1	1	0
0	1	0	0	1	0
1	0	0	1	1	0
0	0	0	1	1	0
0	0	0	1	0	0
0	0	1	0	1	0
0	0	0	0	1	0
0	0	0	1	0	0
0	0	0	1	0	0
1	0	1	1	1	0
1	1	0	0	1	0
0	0	0	1	1	0
1	0	0	1	1	0
0	0	0	1	1	0
0	0	0	0	1	0
1	0	1	0	1	0
0	0	0	1	0	0
1	1	0	1	1	0

0	0	0	0	1	0
0	0	0	1	0	0
1	0	0	1	0	0
0	0	1	1	1	0
0	0	0	1	0	0
0	0	0	0	1	0
0	0	1	1	0	0
0	0	0	1	0	0
0	0	1	1	1	0
0	0	0	0	1	0
0	0	0	1	0	0
0	0	0	1	1	0
0	0	0	1	1	0
0	1	0	1	0	0
1	1	1	1	0	0
0	0	0	1	0	0
0	0	0	1	1	0
1	0	0	0	1	0
0	0	0	0	1	0
0	0	0	1	0	0
0	0	0	1	0	0
1	0	1	1	0	0
0	0	0	1	1	0
1	1	1	0	0	0
0	0	0	1	1	0
0	0	0	1	1	0
1	1	1	1	0	0
0	0	0	1	1	0
1	1	0	1	0	0
0	0	0	1	0	0
0	0	0	1	0	0
0	0	0	0	1	0
1	0	0	1	0	0
0	0	0	0	1	0
0	0	0	1	0	0
1	1	1	1	0	0
1	0	0	0	1	0
0	0	0	1	1	0
0	0	0	1	1	0
0	0	0	1	1	0
0	0	0	1	1	0
0	0	0	1	1	0
0	0	0	0	1	0
1	0	1	1	1	0
0	0	0	1	0	0
0	0	0	1	1	0
0	0	1	1	1	0
0	0	0	1	1	0

0	0	0	1	1	0
0	0	0	1	1	0
0	0	0	0	0	0
1	1	0	1	0	0
0	0	0	0	1	0
0	0	99	0	1	0
1	0	0	1	1	0
1	0	0	1	0	0
0	0	0	1	0	0
0	0	0	1	1	0
0	0	1	0	1	0
0	0	0	0	1	0
1	1	1	1	0	0
1	0	0	1	0	0
0	0	0	1	1	0
0	0	0	1	1	0
0	0	0	0	1	0
0	0	0	0	1	0
0	0	0	1	1	0
0	0	0	1	1	0
0	0	0	0	1	0
1	99	0	1	1	0
0	0	0	1	0	0
0	0	0	1	0	0
0	0	0	1	1	0
0	0	0	1	0	0
0	0	0	1	0	0
0	0	0	1	0	0
0	0	0	1	1	0
0	0	0	1	0	0
0	0	0	1	0	0
0	0	0	1	0	0
0	0	0	1	1	0
0	0	0	1	0	0
0	0	1	1	0	0
0	0	99	1	1	0
0	0	0	1	0	0
0	0	1	1	0	0
0	0	0	1	1	0
0	0	0	1	0	0
0	1	0	1	0	0
0	0	0	1	1	0
1	1	1	1	0	0
0	1	1	1	1	0
0	0	0	1	0	0
0	1	0	1	0	1
0	0	0	1	0	0
0	0	0	0	1	0

0	0	0	1	0	0
0	0	0	1	1	0
0	0	0	1	0	0
0	1	0	1	1	0
1	0	0	1	0	0
0	1	99	1	0	0
0	0	0	1	0	0
1	1	0	1	0	0
1	0	1	0	1	0
0	1	1	1	0	0
0	1	0	1	0	0
1	0	0	1	0	0
0	0	0	0	1	0
0	0	0	1	1	0
0	0	0	1	0	0
0	0	0	1	0	0
1	1	1	1	0	0
0	0	0	1	0	0
0	0	0	1	1	0
1	0	1	1	0	0
0	1	0	1	1	0
1	0	0	0	0	0
1	0	0	0	1	0
99	99	99	0	1	1
0	0	0	1	0	0
1	0	0	1	0	0
0	1	0	1	1	0
0	0	0	1	0	0
0	0	0	0	1	0
99	99	99	0	1	0
0	1	0	1	1	0
0	0	0	1	1	0
0	1	0	1	0	0
1	0	0	1	1	0
0	0	0	0	1	0
0	0	0	0	1	0
0	0	1	1	0	0
0	1	99	1	0	0
0	1	0	0	1	0
0	0	0	1	1	0
0	0	0	1	0	0
0	0	0	1	1	0
0	0	0	0	1	0
99	99	99	1	0	0
1	0	1	1	0	0
0	0	0	1	1	0
0	0	0	1	0	0
0	1	1	1	1	0

0	1	0	1	1	0
0	0	0	1	0	0
0	0	0	0	1	0
0	0	1	0	1	0
0	0	0	1	1	0
1	0	0	1	1	0
0	0	0	1	0	0
0	0	0	1	1	0
1	1	1	1	0	0
0	0	0	1	1	0
0	0	1	1	0	0
1	0	0	1	1	0
0	1	0	1	0	0
1	0	0	1	1	0
0	0	0	1	1	0
0	0	0	1	0	0
0	0	0	1	1	0
0	0	0	1	0	0
0	0	0	1	0	0
0	0	0	1	1	0
0	0	0	1	0	0
1	1	1	1	1	0
0	0	0	1	0	0
0	1	0	0	1	0
1	1	1	1	0	0
0	0	0	0	1	0
1	0	0	1	0	0
1	0	1	1	1	0
0	0	0	0	1	0
1	0	0	1	1	0
0	0	0	1	1	0
0	0	0	1	1	0
0	0	0	0	1	0
1	1	99	1	0	0
0	1	0	1	0	0
0	0	0	1	1	0
0	1	1	1	1	0
1	1	0	1	1	0
0	0	0	0	1	0
1	0	1	1	0	0
0	0	0	1	1	0
0	0	0	1	0	0
0	0	0	0	1	0
1	0	1	1	0	0
0	0	0	1	0	0
0	0	0	0	1	0
0	0	0	1	1	0
1	1	0	1	1	0

1	0	0	0	1	0
0	0	0	1	0	0
0	0	0	1	1	0
0	0	0	0	1	0
1	1	1	1	0	0
0	0	1	1	0	0
0	0	0	1	0	0
1	1	0	0	1	0
1	0	1	1	0	0
0	0	0	1	0	0
0	0	0	1	0	0
0	0	1	1	0	0
0	0	0	1	1	0
0	0	0	1	0	0
0	0	0	0	1	0
0	0	0	1	1	0
0	0	0	0	1	0
0	0	0	0	1	0
1	0	0	1	1	0
1	0	0	1	1	0
0	0	0	1	1	0
0	0	0	1	0	0
0	0	0	1	1	0
1	0	0	1	1	0
99	99	99	1	1	0
99	0	0	1	0	0
0	0	0	1	0	0
0	0	0	0	1	0
0	0	1	1	0	0
0	0	0	0	0	1
1	1	0	1	0	0
0	0	0	1	0	0
1	1	0	1	0	0
0	0	1	0	1	0
0	0	0	0	1	0
0	0	0	0	1	0
1	1	1	0	1	0
0	0	0	1	1	0
0	0	0	1	1	0
0	0	0	0	1	0
0	1	0	1	0	0
0	0	0	0	1	0
0	0	0	1	0	0
0	0	0	0	1	0
1	1	1	1	0	0
0	0	0	1	0	0
0	0	0	1	1	0
0	0	0	1	1	0

0	0	0	1	1	0
0	0	0	1	1	0
0	0	0	1	1	0
0	0	0	1	0	0
1	1	1	1	0	0
0	0	0	1	0	0
0	0	0	1	0	0
0	0	0	1	1	0
0	0	0	1	0	0
0	0	0	1	0	0
0	0	1	0	1	0
0	0	0	0	1	0
1	0	0	1	0	0
0	0	0	1	0	0
1	0	1	1	1	0
0	0	0	0	1	0
0	0	0	0	1	0
0	0	0	1	0	0
0	0	0	0	1	0
0	0	0	1	0	0
0	0	0	1	0	0
0	0	0	1	1	0
0	0	0	1	0	0
1	0	0	1	0	0
1	1	1	1	0	0
0	0	0	1	1	0
0	1	1	1	1	0
0	0	0	1	1	0
0	0	0	1	1	0
0	0	0	1	0	0
0	0	1	1	0	0
0	0	0	1	1	0
1	1	1	1	0	0
0	0	1	1	0	0
0	0	0	0	1	0
0	0	0	0	1	0
0	0	0	1	1	0
0	0	0	1	0	0
1	99	99	1	0	0
1	0	0	1	1	0
1	1	0	1	0	0
0	0	0	1	0	0
0	0	0	1	1	0
0	0	0	1	0	0
0	0	0	1	1	0
0	0	0	1	1	0
0	0	0	0	1	0
0	0	0	0	1	0

0	0	0	0	1	0
0	0	0	1	0	0
1	0	0	0	1	0
1	0	1	1	1	0
0	0	0	1	0	0
0	0	0	1	1	0
0	0	0	0	0	0
0	0	0	1	1	0
0	0	0	1	1	0
0	0	0	1	1	0
0	1	1	1	0	0
1	0	0	1	0	0
1	0	0	1	1	0
0	0	0	1	1	0
1	0	0	1	0	0
0	0	0	1	0	0
0	0	0	1	0	0
0	0	0	1	0	0
1	0	0	1	0	0
0	0	0	1	0	0
0	0	1	0	1	0
0	0	0	1	1	0
0	0	0	1	1	0
0	0	0	1	0	0
1	1	0	1	0	0
0	0	0	1	1	0
0	0	0	0	0	0
0	0	0	0	1	0
0	0	0	1	1	0
0	0	0	1	0	0
1	99	99	0	1	0
0	0	0	1	0	0
0	1	0	1	1	0
0	0	1	1	1	0
0	0	0	1	0	0
0	0	0	0	1	0
0	0	0	1	1	0
1	99	1	1	1	0
0	0	0	0	1	0
0	0	0	1	0	0
0	0	0	1	1	0
0	0	0	1	0	0
0	0	0	0	1	0
0	0	0	1	0	0
0	1	0	1	0	0
0	0	0	1	0	0
0	0	0	1	1	0
0	0	0	1	1	0

0	0	0	0	1	0
0	0	0	0	1	0
0	0	0	1	1	0
0	0	0	0	1	0
0	0	0	1	0	0
0	0	0	1	0	0
0	0	0	1	1	0
0	0	0	1	0	0
0	0	0	1	0	0
0	0	99	1	1	0
0	0	0	0	1	0
0	0	0	1	0	1
1	1	1	1	0	0
0	0	0	1	0	0
0	0	0	1	0	0
0	1	0	1	0	0
1	0	0	1	0	0
0	1	0	1	0	0
1	0	1	1	1	0
1	0	0	1	0	0
1	1	0	1	0	0
0	0	0	0	1	0
0	0	0	1	1	0
0	0	0	1	1	0
1	0	0	1	1	0
0	0	0	1	1	0
0	0	0	1	0	0
0	0	0	0	1	0
0	1	0	1	1	0
0	0	0	1	0	0
99	99	99	1	1	0
0	0	0	1	0	0
0	0	0	1	1	0
0	0	0	1	1	0
0	0	0	1	1	0
0	0	0	0	1	0
0	0	0	1	0	0
0	0	0	1	0	0
0	0	0	1	0	0
0	0	0	1	1	0
1	1	1	1	1	0
0	0	0	0	1	0
1	0	0	1	0	0
0	0	0	1	0	0
1	0	0	1	1	0
0	1	0	1	1	0
0	0	0	1	1	0
0	1	0	1	0	0

0	0	0	1	1	0
0	0	0	0	1	0
0	0	0	1	1	0
0	0	0	1	1	0
0	0	0	1	0	0
0	0	0	1	1	0
0	0	0	1	0	0
0	1	0	1	0	0
0	0	0	1	1	0
0	0	0	1	1	0
0	0	0	1	0	0
1	0	0	0	1	0
1	0	0	0	1	0
0	0	0	1	0	0
0	0	0	1	1	0
0	0	0	1	0	0
1	0	0	1	1	0
1	1	0	1	0	0
1	1	1	1	0	0
0	0	0	1	1	0
0	0	0	1	1	0
0	1	0	1	0	0
1	0	0	1	0	0
0	0	0	1	0	0
1	0	1	1	1	0
1	1	0	1	0	0
0	0	0	1	1	0
1	1	0	1	0	0
1	0	1	1	0	0
1	0	1	1	0	0
0	0	0	1	1	0
0	0	0	1	1	0
0	0	0	0	1	0
1	0	0	1	0	0
0	0	0	1	1	0
0	0	0	1	0	0
0	0	0	1	1	0
1	1	0	1	1	0
1	0	0	1	0	0
1	0	0	0	1	0
0	0	0	1	0	0
1	0	0	1	1	0
1	1	1	1	0	0
0	0	0	1	1	0
0	0	0	1	1	0
1	0	1	1	0	0
0	0	0	0	1	0
0	0	0	0	1	0

0	1	0	1	0	0
0	0	0	1	1	0
0	0	0	1	0	0
0	0	1	1	1	0
0	1	1	1	1	0
1	0	1	1	0	0
0	0	0	1	0	0
1	0	0	1	0	0
0	0	0	1	1	0
1	0	0	1	0	0
0	0	0	1	1	0
1	99	1	1	0	0
0	0	1	1	0	0
1	1	1	1	0	0
0	1	1	1	1	0
0	0	0	1	0	0
0	1	0	1	0	0
0	0	99	1	1	0
1	0	0	1	0	0
0	0	99	0	1	0
0	1	0	1	0	0
0	0	0	1	1	0
1	1	1	1	0	0
1	1	1	1	0	0
0	0	0	1	1	0
1	0	0	1	0	0
0	0	0	1	1	0
1	0	0	1	0	0
0	0	0	1	1	0
1	1	0	1	0	0
0	0	1	1	1	0
99	1	1	1	1	0
1	99	99	1	1	0
0	0	0	0	1	0
1	1	1	1	0	0
0	0	0	1	1	0
0	0	0	1	1	0
0	0	0	1	1	0
0	0	0	1	1	0
0	0	0	1	1	0
1	0	1	1	1	0
0	0	0	1	1	0
0	0	0	1	0	0
0	0	0	1	1	0
0	0	0	1	1	0
0	0	0	1	1	0
0	0	0	1	1	0
0	1	0	1	1	0

0	0	0	1	1	0
0	99	99	1	1	0
0	0	0	1	0	0
0	0	0	1	1	0
1	0	0	1	0	0
0	0	1	1	1	0
0	0	0	1	1	0
0	1	1	1	0	0
0	0	0	1	0	0
0	0	0	1	1	0
0	0	0	1	0	0
0	99	99	1	0	0
0	0	99	1	1	0
0	0	0	1	1	0
0	0	0	1	0	0
0	0	0	1	0	0
0	0	1	1	1	0
0	0	0	1	1	0
1	1	0	1	0	0
0	0	0	1	1	0
0	0	0	1	1	0
0	0	0	1	0	0
0	0	0	1	1	0
0	1	0	1	0	0
0	0	1	1	0	0
1	0	1	1	0	0
0	0	0	1	1	0
0	0	0	0	1	0
0	0	0	1	0	0
0	0	0	1	1	0
1	0	1	1	1	0
1	1	0	1	1	0
0	0	0	1	1	0
1	0	0	1	1	0
0	0	0	1	1	0
0	0	0	1	1	0
0	1	0	1	1	0
0	0	0	1	1	0
0	0	0	1	0	0
0	0	0	1	0	0
1	0	1	1	1	0
0	0	0	1	1	0
0	1	0	1	1	0
0	0	0	1	1	0
0	0	0	1	0	0
0	0	0	1	1	0
0	0	0	0	1	0
0	0	0	1	0	0

0	0	0	1	1	0
0	0	0	1	0	0
0	0	1	1	0	0
0	0	0	1	0	0
0	0	0	1	0	0
0	1	0	1	1	0
0	0	0	1	1	0
0	0	1	0	1	0
0	0	0	1	0	0
0	0	0	1	1	0
0	0	1	1	0	0
0	0	0	1	0	0
0	0	1	1	1	0
0	0	0	1	0	0
0	0	0	1	1	0
0	0	0	1	1	0
0	0	0	0	1	0
0	0	0	1	1	0
0	0	1	1	1	0
0	99	1	1	1	0
1	1	0	1	1	0
1	1	0	1	1	0
1	0	1	1	0	0
0	0	0	1	1	0
0	1	0	0	1	0
0	0	0	1	1	0
0	0	0	1	1	0
0	0	0	1	0	0
0	1	1	1	0	0
0	0	0	1	0	0
0	1	1	1	0	0
1	99	0	0	1	0
0	99	0	1	1	0
1	0	0	1	1	0
0	0	1	1	0	0
0	0	0	1	0	0
0	0	0	1	1	0
0	0	0	0	1	0
0	0	0	1	0	0
0	0	0	1	0	0
0	0	0	0	1	0
0	0	0	1	1	0
1	0	0	1	0	0
0	0	1	1	1	0
0	0	1	1	1	0
0	0	0	1	1	0
1	1	0	1	1	0
0	0	0	1	1	0

0	0	0	1	0	0
0	0	0	1	1	0
0	0	0	1	1	0
0	0	0	1	0	0
1	1	0	1	0	0
0	0	0	1	1	0
0	0	0	1	1	0
1	1	0	0	0	0
0	0	0	1	0	0
1	0	0	1	1	0
0	0	0	1	0	0
0	0	0	0	1	0
0	0	0	1	0	0
0	0	0	1	0	0
0	0	0	1	0	0
0	0	0	1	0	0
0	0	0	1	1	0
0	0	0	0	1	0
0	0	0	1	0	0
0	0	0	1	1	0
0	0	0	1	1	0
0	0	0	1	1	0
0	0	0	0	1	0
0	0	1	0	1	0
0	0	1	1	1	0
0	0	0	1	0	0
0	0	0	1	0	0
1	1	1	1	0	0
0	0	0	1	1	0
0	0	0	1	0	0
0	1	0	1	0	0
0	0	0	0	1	0
0	0	0	1	0	0
0	0	0	1	1	0
0	0	0	1	0	0
0	0	0	1	0	0
0	0	0	0	0	0
0	0	0	1	1	0
0	0	0	1	0	0
0	0	0	1	1	0
0	0	1	0	1	0
1	1	1	1	1	0
0	0	0	1	0	0
0	0	0	0	1	0
0	0	0	1	1	0
1	1	1	1	0	0
0	0	0	1	1	0
0	0	0	1	1	0

0	0	0	1	0	0
0	0	1	1	1	0
0	0	0	1	1	0
0	0	0	1	0	0
99	99	99	0	0	1
0	0	1	1	0	0
0	0	1	1	0	0
0	0	0	1	0	0
0	0	0	1	1	0
0	0	0	1	0	0
0	0	1	1	1	0
0	0	0	1	0	0
1	0	1	0	1	0
0	0	0	1	1	0
1	0	0	0	1	0
1	1	0	1	0	0
0	0	1	1	1	0
0	0	0	1	1	0
0	0	1	0	1	0
1	0	0	1	1	0
0	0	0	1	0	0
0	0	0	1	1	0
0	0	0	0	1	0
0	0	0	1	0	0
0	0	0	1	0	0
0	0	0	1	0	0
0	1	0	1	1	0
0	0	0	1	1	0
1	1	1	1	0	0
1	0	0	1	1	0
0	0	0	1	1	0
0	1	1	1	1	1
0	0	0	0	1	0
0	0	0	1	1	0
0	0	0	1	1	0
1	1	0	0	1	0
0	0	0	1	0	0
0	0	0	1	0	0
0	0	0	1	0	0
0	1	1	0	1	0
0	1	1	1	0	0
0	0	0	0	1	0
0	0	1	1	0	0
0	1	0	1	1	0
0	0	0	1	1	0
1	1	99	1	0	0
0	0	0	1	1	0
0	0	1	0	1	0

1	0	0	1	0	0
0	1	0	1	1	0
1	1	1	1	0	0
0	1	0	1	0	0
0	0	0	1	1	0
0	0	1	1	1	0
1	0	1	1	1	0
0	1	0	1	0	0
0	0	1	0	1	0
0	0	0	1	1	0
0	0	0	1	0	0
0	0	1	1	0	0
1	1	1	1	0	0
0	1	1	1	0	0
0	0	0	1	0	0
0	0	0	1	0	0
0	0	0	1	1	0
1	0	0	1	0	0
0	0	0	1	1	0
0	0	0	1	1	0
0	1	0	1	0	0
1	1	1	1	1	0
0	0	0	1	1	0
1	1	1	1	0	0
0	0	0	1	1	0
0	0	0	1	1	0
1	1	1	1	0	0
0	0	1	1	1	0
0	0	0	1	1	0
0	0	0	1	1	0
0	0	0	1	1	0
1	1	0	1	0	0
0	0	0	1	1	0
0	0	1	1	0	0
0	0	0	0	1	0
0	0	0	1	1	0
0	0	0	1	1	0
1	1	0	1	0	0
0	0	0	0	1	0
0	0	0	1	1	0
0	0	0	1	0	0
0	0	0	0	1	0
0	0	0	1	1	0
0	0	0	1	0	0
1	0	0	1	1	0
0	0	0	1	1	0
0	0	0	1	1	0
0	0	0	1	0	0

0	0	1	1	0	0
1	0	1	1	1	0
0	0	0	0	1	0
0	0	1	1	1	0
0	0	0	1	0	0
0	0	0	1	1	0
0	0	0	0	1	0
0	0	0	1	0	0
0	0	0	1	1	0
1	0	0	1	1	0
1	0	0	0	1	0
0	0	0	1	1	0
0	0	0	0	1	0
1	1	0	1	0	0
0	0	0	1	0	0
0	0	0	1	1	0
0	0	0	1	0	0
1	0	0	1	0	0
1	0	1	1	1	0
0	0	0	1	0	0
0	0	0	1	1	0
0	0	0	1	1	0
0	0	0	1	0	0
1	0	0	1	0	0
1	1	1	1	0	0
1	1	1	1	0	0
0	0	0	1	0	0
0	0	0	1	1	0
0	0	1	1	0	0
1	1	0	1	1	0
0	0	0	1	1	0
0	0	0	1	1	0
0	0	0	1	0	0
0	0	0	1	0	0
0	0	0	1	1	0
0	0	1	1	0	0
0	0	0	1	0	0
0	0	0	1	1	0
0	1	0	1	0	0
0	0	0	1	0	0
0	0	0	1	0	0
0	0	0	1	0	0
0	0	0	1	0	0
0	0	0	0	1	0
1	1	1	1	0	0
1	0	0	1	0	0
0	0	1	1	0	0
0	0	0	1	1	0

0	0	0	1	1	0
0	0	0	0	1	0
0	0	0	1	1	0
1	0	0	1	1	0
0	0	0	0	1	0
0	0	0	1	1	0
0	1	0	1	0	0
1	0	0	1	1	0
0	0	0	1	0	0
0	0	1	1	0	0
0	0	0	1	1	0
1	0	0	1	1	0
1	1	0	1	1	0
1	1	0	1	1	0
0	0	0	1	1	0
1	0	0	0	1	0
1	0	1	1	0	0
0	0	0	1	0	0
1	99	0	1	1	0
0	0	0	0	1	0
0	0	0	1	0	0
0	0	0	1	0	0
0	1	1	1	1	0
0	0	0	1	0	0
0	0	0	1	1	0
1	0	1	1	0	0
0	0	0	1	1	0
0	0	0	1	0	0
0	0	0	1	1	0
0	0	0	1	0	0
0	0	0	1	1	0
1	0	1	1	1	0
0	0	1	0	1	0
0	0	0	1	0	0
1	1	0	1	1	0
0	0	0	0	1	0
1	1	1	1	0	0
1	0	0	1	1	0
0	0	0	1	0	0
1	1	1	1	0	0
0	0	0	0	1	0
0	0	0	1	0	0
99	1	99	0	1	0
0	1	1	1	1	0
0	0	0	1	0	1
1	0	0	1	1	0
0	0	0	1	1	0
1	1	1	1	1	0

0	0	1	1	1	0
0	1	0	1	1	0
0	0	0	1	1	0
1	1	0	1	0	0
0	0	1	1	0	0
0	0	0	1	1	0
0	0	1	0	1	0
0	0	0	1	0	0
0	0	0	1	1	0
0	0	0	1	0	0
0	0	0	0	0	0
0	0	0	0	1	0
1	0	0	1	1	0
0	1	0	1	1	0
0	0	0	1	0	0
0	0	0	1	1	0
1	0	0	1	0	0
0	0	0	1	1	0
1	1	0	1	0	0
1	99	1	1	0	0
0	0	0	1	0	0
1	1	1	1	0	0
0	0	0	1	0	0
0	0	0	1	1	0
0	1	0	1	0	0
0	0	0	0	1	0
0	0	0	1	1	0
0	0	0	1	1	0
1	1	1	1	0	0
0	1	0	1	1	0
0	0	0	1	1	0
0	0	0	0	0	0
0	0	0	1	0	0
1	0	1	1	0	0
1	0	0	1	0	0
0	0	0	1	1	0
0	0	0	1	0	0
0	0	0	1	1	0
0	0	0	1	1	0
0	0	0	1	0	0
0	0	0	1	1	0
0	0	0	0	1	0
0	0	0	1	0	0
0	0	1	1	0	0
0	0	0	0	1	0
1	1	0	1	1	0
0	0	0	1	0	0
0	1	0	1	1	0

1	0	0	1	0	0
0	0	0	1	0	0
1	0	1	1	0	0
99	99	99	0	0	0
1	0	1	1	0	0
0	1	0	1	0	0
0	0	0	1	1	0
0	0	0	1	1	0
0	0	0	1	0	0
0	0	0	1	1	0
0	0	0	1	1	0
1	0	1	1	0	0
1	0	0	1	1	0
1	1	1	1	1	0
0	0	0	1	0	0
0	0	1	1	0	0
1	1	1	1	0	0
0	0	0	1	0	0
0	0	0	0	1	0
0	0	0	1	0	0
0	0	0	1	0	0
0	1	0	1	0	0
0	0	0	1	1	0
0	0	0	1	1	0
1	0	1	1	0	0
1	0	1	1	0	0
0	0	0	1	1	0
0	0	0	1	1	0
0	0	1	1	0	0
0	0	0	1	0	0
0	0	1	1	1	0
0	0	0	1	1	0
1	1	1	1	0	0
0	0	0	1	0	0
0	1	1	1	0	0
0	0	0	1	0	0
1	0	1	1	0	0
0	0	0	1	0	0
1	0	99	1	0	0
1	1	1	1	1	0
1	1	0	1	1	1
0	1	0	1	0	0
0	0	1	1	1	0
1	0	0	1	1	0
0	0	0	1	1	0
0	0	0	1	0	0
1	0	1	0	0	0
0	0	0	1	1	0

1	1	1	1	0	0
1	1	1	1	0	0
0	0	0	0	1	0
0	0	0	1	0	0
1	1	0	1	0	0
0	0	0	1	1	0
0	0	1	0	1	0
1	0	0	1	1	0
1	0	1	1	0	0
0	1	0	1	1	0
0	0	1	1	0	0
0	0	0	1	0	0
0	1	0	0	1	0
0	0	0	1	0	0
0	1	0	1	0	0
0	0	0	1	0	0
0	0	0	1	0	0
0	0	0	1	1	0
1	1	1	1	0	0
0	0	0	1	1	0
1	0	0	1	1	0
0	0	0	1	0	0
1	0	1	0	0	0
0	0	0	1	1	0
0	0	0	1	1	0
0	0	0	1	1	0
0	0	0	1	1	0
1	1	0	1	1	0
0	0	0	1	1	0
0	0	0	0	1	0
0	0	0	1	0	0
0	0	0	1	0	0
0	1	1	1	0	0
0	0	0	1	0	0
0	0	0	1	0	0
0	1	1	1	1	0
0	0	0	1	0	0
0	0	0	1	0	0
0	0	0	1	1	0
0	0	1	1	0	0
1	0	1	1	0	0
1	0	1	1	0	0
0	0	0	0	1	0
0	0	0	1	0	0
0	0	0	0	1	0
0	0	0	0	1	0
99	1	1	1	0	0
0	0	0	1	0	0

0	0	0	1	1	0
0	0	0	0	1	0
0	0	0	0	1	0
0	0	0	1	1	0
0	0	0	1	0	0
0	0	0	1	0	0
0	0	0	0	0	0
0	0	0	0	1	0
0	0	0	1	0	0
0	0	0	1	0	0
0	0	0	1	1	0
0	0	0	1	0	0
0	1	0	1	1	0
0	0	0	1	1	0
0	0	0	1	1	0
0	0	0	1	1	0
0	0	0	1	1	0
0	0	0	1	1	0
1	0	0	1	0	0
1	1	0	1	0	0
0	1	0	1	0	0
0	0	0	0	1	0
0	0	0	1	0	0
0	0	0	1	0	0
0	0	0	1	0	0
0	0	0	1	0	0
0	0	0	1	0	0
0	0	0	1	1	0
1	99	0	1	1	0
0	0	0	0	1	0
0	0	0	1	1	0
0	0	0	0	1	0
0	0	1	1	1	0
1	0	0	0	1	0
0	0	0	1	1	0
0	0	0	1	0	0
0	0	0	0	1	0
0	0	0	1	1	0
1	0	1	0	1	0
0	1	0	0	1	0
0	0	0	1	0	0
0	0	0	1	1	0
0	0	0	0	1	0
0	0	0	1	0	0
0	1	1	1	0	0
0	0	0	1	1	0
0	0	0	1	1	0
0	0	1	1	0	0

0	0	0	0	1	0
0	0	0	1	1	0
0	0	0	1	1	0
0	0	0	1	1	0
0	0	0	1	1	0
0	0	0	1	0	0
99	99	99	1	0	0
0	1	0	1	0	0
0	0	1	1	1	0
0	0	0	1	1	0
0	0	0	1	0	0
0	0	0	1	0	0
0	0	0	1	0	0
0	0	0	0	0	0
0	0	0	1	0	0
0	0	0	1	1	0
0	0	1	1	1	0
99	99	0	1	1	0
1	0	0	1	1	0
0	0	0	1	0	0
0	0	0	0	1	0
0	0	0	1	1	0
1	1	1	1	1	0
1	0	1	1	1	0
0	0	0	0	1	0
1	0	0	1	0	0
0	0	0	1	0	0
0	0	0	1	0	0
99	99	99	1	0	0
0	1	0	1	0	0
0	0	0	0	0	0
0	1	1	1	1	0
1	0	0	1	1	0
0	0	0	1	0	0
0	1	0	1	0	0
0	0	0	1	1	0
0	0	0	1	1	0
0	0	0	1	0	0
1	1	1	1	1	0
0	0	0	1	0	0
0	0	0	1	1	0
0	0	0	1	1	0
0	0	0	1	1	0
0	1	0	1	1	0
1	1	0	1	1	0
0	0	0	1	0	0
0	0	0	0	1	0
0	0	0	1	0	0

0	0	0	1	1	0
0	99	99	1	0	0
0	1	0	1	0	0
0	0	0	1	1	0
0	0	0	1	1	0
0	0	0	1	1	0
0	0	0	1	1	0
0	0	0	1	1	0
0	0	0	0	1	0
1	1	1	1	0	0
1	1	99	0	0	0
0	0	0	1	0	0
0	0	0	1	1	0
0	1	0	0	1	0
0	0	0	0	1	0
0	0	0	1	1	0
0	0	0	1	1	0
99	0	0	1	1	0
0	0	0	1	0	0
1	0	0	0	1	0
0	0	0	0	1	0
0	1	0	1	0	0
0	0	0	0	1	0
0	0	1	1	1	0
0	0	0	1	1	0
0	0	0	1	0	0
0	0	0	1	0	0
0	0	0	1	0	0
0	0	0	1	0	0
0	0	0	1	0	0
1	0	1	0	1	0
0	0	0	1	1	0
0	0	0	1	0	0
0	0	0	1	1	0
0	1	0	1	0	0
0	0	0	0	0	0
0	0	1	1	1	0
0	0	0	1	0	0
0	0	0	1	1	0
1	0	1	1	0	0
0	0	0	1	0	0
0	0	0	1	1	0
1	1	0	1	0	0
0	0	0	1	1	0
0	0	0	1	1	0
0	0	0	1	0	0
0	0	0	1	0	0
0	0	0	1	0	0

0	0	0	1	0	0
0	0	0	1	0	0
1	1	0	1	1	0
0	0	0	1	0	0
1	0	0	1	1	0
1	1	0	1	0	0
0	0	1	1	0	0
0	0	0	1	1	0
0	0	0	1	0	0
1	0	0	1	0	0
0	0	0	1	1	0
0	0	0	0	1	0
0	0	0	1	1	0
0	0	1	1	1	0
0	0	0	1	0	0
1	1	1	1	0	0
1	0	1	1	0	0
0	0	0	0	1	0
1	0	0	1	1	0
0	0	0	1	1	0
0	0	0	1	1	0
1	0	1	1	1	0
0	0	1	1	0	0
0	0	1	1	1	0
0	0	0	1	1	0
0	0	1	1	1	0
0	0	0	1	1	0
0	0	0	1	1	0
1	0	0	1	0	0
0	0	0	1	0	0
0	0	0	1	1	0
0	0	0	1	1	0
1	99	0	1	1	0
0	0	0	1	0	0
0	0	0	0	1	0
0	0	0	1	1	0
0	0	1	1	1	0
0	0	0	1	1	0
0	0	0	1	0	0
0	0	0	1	1	0
0	0	0	1	0	0
1	1	1	1	1	0
0	0	0	1	1	0
0	0	0	0	1	0
1	0	0	1	0	0
0	0	0	1	0	0
0	0	0	0	1	0
99	99	99	0	0	0

0	1	0	1	1	0
0	0	0	1	1	0
0	0	1	1	0	0
1	1	0	1	1	0
0	0	0	0	1	0
0	1	0	1	0	0
1	0	0	1	1	0
0	0	0	1	0	0
0	0	0	1	0	0
0	0	0	1	0	0
1	1	1	1	0	0
0	0	0	1	0	0
0	0	0	1	1	0
1	0	1	1	0	0
0	0	1	1	1	0
0	1	0	1	0	0
0	0	0	1	0	0
1	1	0	1	0	0
1	1	0	1	1	0
0	0	0	0	1	0
1	1	1	1	1	0
1	0	1	1	1	0
0	0	0	0	1	0
0	0	0	1	0	0
1	0	1	1	1	0
0	0	0	1	0	0
1	0	1	1	0	0
0	0	0	1	1	0
0	0	0	1	1	0
99	1	99	1	0	0
0	0	0	1	0	0
0	0	0	1	1	0
0	0	0	1	0	0
0	0	0	1	0	0
0	0	0	1	1	0
0	0	0	1	0	0
0	0	0	1	1	0
0	0	0	0	1	0
0	1	0	1	0	0
0	0	0	1	0	0
1	1	1	1	1	0
0	0	0	1	1	0
0	0	0	1	0	0
0	0	0	1	1	0
0	0	0	1	1	0
0	0	0	1	1	0
0	0	0	1	1	0
0	0	0	1	0	0

0	0	0	1	1	0
0	0	99	1	1	0
1	0	0	1	0	0
0	0	0	1	1	0
0	0	0	0	1	0
0	0	0	1	0	0
0	1	0	1	0	0
0	0	0	1	1	0
1	1	0	1	0	0
0	0	0	1	1	0
0	0	0	1	0	0
1	0	0	1	1	0
0	0	0	0	1	0
0	0	0	1	0	0
0	0	0	1	1	0
1	0	1	1	0	0
0	0	0	1	0	0
0	0	0	1	0	0
0	1	0	1	1	0
0	0	0	1	0	0
0	0	99	1	0	0
1	0	0	1	0	0
0	0	0	1	0	0
1	1	1	1	0	0
0	0	0	1	0	0
0	0	0	1	0	0
1	0	0	1	0	0
1	0	1	1	1	0
0	0	0	0	1	0
99	1	0	1	0	0
0	0	0	1	0	0
0	1	1	1	0	0
0	1	1	1	0	0
0	0	0	1	0	0
0	0	0	1	0	0
1	1	1	1	1	0
1	0	0	1	1	0
0	0	0	1	0	0
0	0	0	1	1	0
1	0	1	0	1	0
0	1	1	1	0	0
0	0	99	1	1	0
0	0	0	1	1	0
0	0	0	0	1	0
0	0	0	1	1	0
0	0	0	1	1	0
0	0	1	0	1	0
0	0	1	1	1	0

0	0	0	1	1	0
1	0	0	1	1	0
0	0	0	1	1	0
0	0	0	1	0	0
0	0	0	0	1	0
1	0	0	1	1	0
0	0	0	1	0	0
0	0	0	1	0	0
0	0	0	1	1	0
1	1	0	1	1	0
0	0	0	1	0	0
0	0	0	1	0	0
0	0	99	1	1	0
0	0	0	1	0	0
1	1	1	1	1	0
0	0	0	1	0	0
0	0	0	1	1	0
0	0	0	1	0	0
1	1	0	1	0	0
0	0	1	1	0	0
0	0	0	1	0	0
0	1	0	1	0	0
0	0	1	1	0	0
1	1	1	1	1	0
0	0	0	1	0	0
0	0	1	1	1	0
0	0	0	1	0	0
0	0	0	1	0	0
0	0	1	1	0	0
0	0	1	1	1	0
0	0	0	1	1	0
0	0	1	1	0	0
0	1	0	1	0	0
0	0	0	0	1	0
1	0	1	1	0	0
0	1	0	1	0	0
0	0	0	1	1	0
1	1	0	1	1	0
0	0	0	1	0	0
1	0	0	0	0	0
0	0	1	1	0	0
99	99	99	1	1	0
0	0	0	1	0	0
0	0	0	1	1	0
1	1	0	1	1	0
1	0	1	1	1	0
1	0	1	1	0	0
0	0	0	1	0	0

0	0	0	1	1	0
1	0	0	1	1	0
0	0	0	1	0	0
0	0	0	1	1	0
1	0	0	1	0	0
0	1	1	0	0	1
0	0	0	1	0	0
0	0	0	1	1	0
0	0	0	1	1	0
0	0	1	1	0	0
99	99	99	1	0	0
0	0	1	1	0	0
0	0	0	1	1	0
0	0	0	1	0	0
1	1	1	1	1	0
0	0	0	1	1	0
0	0	1	1	0	0
0	0	0	1	0	0
0	0	0	1	1	0
0	0	1	0	1	0
1	0	0	1	0	0
0	0	0	1	0	0
0	0	0	1	1	0
0	0	0	1	0	0
0	0	0	1	0	0
0	1	0	1	1	0
99	99	99	0	1	0
0	0	0	1	0	0
0	0	0	1	1	0
0	0	0	0	1	0
0	1	1	1	1	0
0	0	99	1	1	0
0	0	0	0	1	0
1	0	0	1	0	0
1	0	1	1	0	0
1	1	0	1	1	0
0	0	0	1	0	0
0	0	0	1	1	0
0	0	0	1	0	0
0	0	0	1	1	0
1	1	0	1	1	0
1	0	0	1	1	0
99	0	0	1	1	0
0	0	0	1	1	0
0	0	0	1	1	0
1	1	1	1	1	0
0	0	0	1	0	0
1	0	0	1	1	0

0	0	0	1	1	0
0	1	0	1	0	0
0	0	1	1	0	0
0	0	0	1	0	0
0	0	0	1	0	0
0	0	0	1	1	0
0	0	0	0	1	0
0	1	0	1	0	0
0	0	0	1	1	0
0	0	0	1	0	0
0	1	0	1	0	0
99	99	99	0	0	0
0	0	0	1	1	0
0	0	0	1	0	0
0	0	0	1	1	0
1	1	0	1	0	0
0	0	1	1	0	0
0	1	1	1	0	0
1	0	1	1	1	0
0	0	0	1	0	0
0	0	0	1	1	0
0	0	0	1	1	0
0	0	1	1	0	0
0	0	0	1	0	0
0	0	0	1	1	0
0	0	1	1	0	0
0	1	0	1	0	0
0	1	0	1	1	0
0	1	0	1	0	1
0	0	0	1	0	0
0	0	0	0	1	0
99	99	99	0	0	0
0	0	0	1	0	0
1	1	0	1	0	0
0	0	0	1	0	0
0	0	1	1	1	0
0	0	0	0	1	0
0	1	0	1	0	0
0	0	0	1	1	0
1	1	1	1	1	0
0	0	0	1	1	0
0	0	0	1	0	0
0	0	0	1	0	0
0	0	0	1	0	0
0	0	0	1	0	0
1	0	1	1	0	0
0	0	0	1	0	0
0	0	0	1	1	0

0	0	0	0	1	0
1	1	1	1	0	0
0	0	0	1	1	0
1	1	0	1	1	0
0	0	0	1	1	0
0	0	0	1	1	0
0	0	0	1	0	0
0	0	0	1	0	0
1	1	1	1	0	0
1	1	0	1	1	0
99	0	0	1	0	0
0	0	0	0	1	0
1	1	1	1	0	0
0	0	1	0	1	0
0	0	1	1	0	0
1	0	0	1	0	0
1	1	1	1	0	0
0	1	1	1	0	0
1	0	1	1	1	0
0	0	0	1	0	0
0	0	0	1	1	0
0	1	1	1	0	0
0	0	0	0	1	0
0	0	0	1	1	0
99	99	99	0	1	0
0	0	0	1	0	0
0	0	0	1	0	0
1	1	0	1	0	0
0	0	0	1	0	0
1	0	0	1	0	0
0	0	0	1	0	0
0	0	0	1	1	0
0	0	0	0	1	0
0	0	0	1	1	0
0	0	0	1	0	0
1	0	1	0	1	0
0	0	0	1	0	0
0	0	0	1	0	0
0	99	0	1	0	0
0	0	0	1	1	0
0	0	0	1	1	0
0	0	0	0	1	0
0	1	1	1	0	0
0	0	0	0	1	0
0	0	0	0	0	0
0	0	1	1	1	0
0	0	0	1	1	0
0	0	0	1	0	0

0	0	0	1	1	0
0	1	0	1	1	0
1	1	1	1	0	0
0	1	0	1	0	0
99	0	0	0	1	0
1	0	0	1	1	0
99	0	0	0	1	0
0	1	1	1	0	0
0	0	0	1	1	0
0	1	1	1	1	0
0	1	0	1	1	0
0	1	0	1	0	0
0	1	1	1	0	0
0	1	1	1	1	0
0	0	0	1	0	0
99	99	99	1	1	0
0	0	0	1	1	0
0	0	0	1	1	0
0	0	0	1	1	0
99	99	99	0	0	0
0	0	0	1	0	0
0	1	0	1	0	0
0	1	0	1	0	0
1	0	0	1	0	0
0	0	0	1	0	0
0	0	0	1	0	0
0	0	0	1	1	0
0	0	0	1	0	0
0	0	0	1	1	0
0	0	0	1	0	0
0	0	0	1	1	0
0	0	0	1	1	0
0	0	1	1	0	0
0	0	0	1	0	0
0	0	0	0	1	0
0	0	0	1	0	0
0	0	0	1	0	0
0	0	0	1	1	0
0	0	0	1	1	0
1	0	0	1	0	0
0	0	0	1	1	0
0	0	0	1	1	0
0	1	0	1	1	0
0	0	0	1	1	0
0	0	0	1	1	0
0	0	0	1	0	0
1	1	1	1	1	0
0	0	0	1	0	0

0	0	1	0	1	0
0	0	0	0	1	0
0	0	0	1	1	0
0	1	1	1	0	0
0	0	0	1	1	0
0	0	0	1	1	0
0	0	0	1	1	0
0	0	0	1	1	0
1	0	0	1	0	0
0	0	0	1	0	0
0	0	0	1	0	0
0	0	0	0	1	0
0	1	0	1	0	0
0	0	1	1	0	0
0	0	0	0	1	0
0	1	0	1	0	0
0	0	0	1	0	0
0	0	1	1	1	0
0	0	0	1	1	0
0	0	0	1	1	0
0	0	0	1	0	0
0	0	0	1	1	0
0	0	0	1	1	0
0	1	1	1	1	0
0	0	0	1	1	0
1	0	0	1	0	0
0	1	0	1	1	0
0	0	0	0	1	0
0	0	0	1	1	0
0	0	0	0	1	0
0	0	0	0	1	0
0	0	0	1	0	0
1	0	1	1	0	0
0	0	0	1	0	0
0	0	0	1	0	0
1	1	0	1	0	0
0	0	0	1	1	0
0	0	0	1	0	0
1	0	0	1	1	0
0	0	0	1	0	0
0	0	0	1	0	0
1	1	1	0	0	1
0	0	0	1	0	0
0	0	0	1	0	0
0	0	0	1	0	0
0	0	0	1	1	0
0	0	0	0	1	0
0	0	0	1	0	0

1	0	0	1	0	0
1	0	1	1	1	0
1	0	1	1	0	0
0	0	0	1	0	0
0	0	0	0	1	0
0	0	0	0	1	0
0	0	0	1	1	0
1	1	1	1	0	0
0	1	0	1	0	0
0	0	0	1	1	0
0	1	0	1	0	0
1	0	0	1	1	0
0	1	0	1	0	0
0	0	0	1	1	0
1	0	1	1	0	0
0	0	0	1	1	0
0	0	0	1	1	0
0	0	0	1	1	0
1	0	0	1	0	0
0	0	0	1	1	0
0	0	0	1	0	0
1	1	0	1	1	0
0	0	0	1	0	0
0	1	0	1	1	0
0	0	0	1	0	0
1	0	1	1	0	0
0	0	1	1	0	0
1	1	1	1	1	0
0	0	0	1	0	0
1	1	1	1	0	0
0	0	1	1	0	0
0	1	1	1	1	0
0	0	0	1	0	0
1	0	0	1	1	0
1	1	0	1	0	0
0	0	0	1	1	0
0	0	0	1	1	0
0	0	0	1	1	0
0	0	0	1	1	0
0	0	0	1	1	0
1	0	0	1	0	0
0	1	0	1	1	0
0	1	0	1	1	0
0	1	0	1	0	0
0	0	0	1	0	0
1	0	1	1	1	0
0	0	0	1	0	0
0	0	0	1	1	0

0	1	0	1	1	0
0	0	0	1	0	0
1	1	0	1	1	0
1	1	1	1	0	0
0	0	0	1	0	0
0	0	1	1	1	0
0	0	0	1	1	0
1	1	0	0	1	0
0	1	0	1	0	0
0	0	0	1	1	0
0	0	1	1	0	0
0	0	0	0	1	0
0	0	0	1	0	0
0	0	0	1	1	0
1	1	0	1	1	0
1	0	0	1	1	0
0	0	0	1	0	0
0	0	0	1	1	0
0	0	1	0	1	0
0	0	0	1	1	0
0	0	0	1	1	0
1	0	0	1	0	0
0	0	0	1	1	0
1	0	0	0	1	0
1	0	0	0	0	1
0	0	0	1	0	0
1	0	0	1	0	0
0	0	0	1	0	0
1	1	0	1	0	0
1	1	0	1	0	0
0	0	0	0	0	0
0	0	0	1	1	0
0	0	0	1	0	0
0	0	0	1	1	0
0	1	0	1	0	0
0	0	0	1	0	0
0	0	0	1	1	0
0	0	1	1	0	0
0	0	0	1	1	0
0	0	0	1	1	0
0	0	0	1	0	0
99	99	99	1	0	0
0	0	0	1	0	0
0	0	1	1	0	0
0	0	0	1	1	0
0	0	0	1	1	0
0	0	0	0	1	0
0	0	0	1	0	0

0	0	0	1	0	0
1	1	0	1	1	0
0	0	0	1	1	0
0	0	0	1	0	0
0	0	0	1	0	0
0	99	0	1	1	0
1	1	0	1	1	0
1	0	0	1	1	0
0	0	0	1	0	0
0	0	0	1	1	0
0	0	0	1	0	0
0	0	0	1	0	0
1	0	1	1	0	0
99	99	0	1	1	0
0	0	0	1	0	0
0	0	1	1	0	0
1	1	0	1	0	0
1	1	0	1	0	0
0	0	0	1	0	0
1	1	1	1	0	0
0	0	1	1	0	0
0	99	1	1	0	0
0	1	0	1	0	0
0	0	1	1	0	0
0	0	0	1	0	0
0	0	0	1	1	0
0	0	0	1	0	0
0	0	0	1	0	0
0	0	1	1	0	0
0	0	0	1	1	0
0	0	0	1	0	0
0	0	0	1	0	0
0	0	0	0	1	0
1	1	1	0	1	0
0	0	0	1	0	0
0	0	1	1	1	0
0	0	0	0	1	0
0	0	0	1	1	0
0	0	0	0	1	0
0	0	0	1	0	0
0	0	0	1	0	0
0	0	0	1	1	0
0	0	0	1	0	0
0	0	0	1	0	0
0	0	0	1	0	0
0	0	0	1	0	0
1	1	0	1	0	0
0	0	0	0	1	0

1	0	0	1	0	0
0	0	0	1	0	0
0	0	0	0	1	0
0	0	0	0	1	0
0	0	0	1	1	0
0	0	0	0	1	0
0	1	1	1	0	0
0	0	0	1	0	0
0	0	0	1	0	0
0	0	0	1	0	0
1	0	0	1	0	0
1	0	0	1	0	0
0	0	0	1	1	0
0	0	0	0	1	0
0	0	0	1	0	0
0	1	1	1	0	0
0	0	0	1	1	0
0	0	0	1	0	0
1	0	0	1	1	0
0	0	0	1	0	0
0	0	0	1	0	0
0	0	0	1	1	0
0	1	0	1	1	0
1	1	0	1	1	0
0	0	0	1	1	0
0	0	1	0	1	0
0	0	0	0	1	0
0	0	0	1	1	0
0	0	0	1	0	0
0	0	0	1	0	0
0	1	0	1	0	0
1	1	0	1	0	0
0	0	1	1	0	0
0	0	0	1	1	0
99	99	0	1	0	0
0	0	0	1	0	0
99	99	99	1	0	0
0	0	0	1	0	0
0	0	0	1	0	0
0	0	0	1	0	0
0	0	1	0	1	0
0	0	0	1	0	0
0	1	0	0	1	0
0	0	0	1	0	0
0	0	99	1	0	0
0	0	0	1	1	0
1	0	1	1	1	0
0	0	0	1	1	0

0	0	0	1	1	0
0	0	0	1	1	0
1	1	0	1	0	0
0	0	0	1	1	0
99	0	0	1	0	0
0	0	0	1	1	0
0	0	0	0	1	0
0	1	0	1	0	0
0	0	0	1	1	0
0	0	0	1	1	0
0	0	0	1	1	0
0	0	0	1	0	0
0	0	0	1	0	0
0	0	0	1	0	0
0	0	0	1	0	0
0	0	0	1	1	0
0	0	0	1	1	0
1	1	1	1	1	0
0	0	1	1	0	0
0	0	99	0	1	0
0	0	0	0	1	0
0	0	0	1	0	0
0	1	1	1	0	0
0	1	0	1	1	0
1	99	99	1	0	0
0	0	0	1	0	0
0	0	0	1	0	0
99	99	99	1	0	0
0	0	0	1	1	0
0	0	0	0	1	0
0	0	0	1	1	0
0	0	0	1	1	0
0	0	0	0	1	0
0	0	0	1	1	0
0	0	1	1	0	0
1	1	1	1	0	0
0	0	0	1	0	0
99	99	99	0	0	0
99	1	99	1	1	0
0	1	1	1	0	0
0	0	0	1	1	0
1	0	1	1	1	0
0	0	0	1	0	0
0	0	0	1	1	0
1	99	99	1	0	0
0	0	1	1	0	0
1	0	1	1	0	0
0	0	0	1	1	0

0	0	0	1	0	0
0	0	0	0	1	0
0	0	0	1	0	0
0	0	0	1	0	0
0	0	0	1	1	0
0	0	0	1	1	0
0	0	0	1	1	0
1	0	0	1	1	0
1	0	0	0	1	0
0	0	0	1	1	0
0	0	1	1	1	0
0	0	0	1	1	0
0	0	0	0	1	0
0	0	0	1	1	0
0	0	0	1	1	0
1	0	0	0	1	0
0	0	0	1	0	0
0	1	0	1	0	0
0	1	0	1	0	0
0	0	0	1	0	0
1	1	0	1	1	0
0	0	0	1	0	0
99	99	99	1	0	0
99	0	0	1	0	0
1	0	1	1	0	0
0	0	1	1	0	0
0	0	1	1	0	0
0	0	0	1	1	0
0	0	1	1	0	0
0	0	0	1	1	0
0	0	0	1	0	0
0	0	0	1	0	0
0	1	0	1	0	0
1	1	1	1	0	0
0	0	0	1	0	0
0	1	0	1	0	0
0	0	0	1	0	0
0	0	0	1	1	0
0	0	0	1	0	0
1	1	1	1	0	0
0	0	0	1	0	0
0	0	0	1	0	0
0	1	0	1	1	0
0	0	0	1	0	0
0	0	1	1	1	0
0	0	0	1	0	1
0	0	0	1	1	0
0	1	0	1	0	0

0	0	1	1	0	0
1	0	0	1	0	0
0	1	0	1	0	0
0	0	0	1	1	0
0	0	0	1	0	0
0	0	0	1	0	0
0	0	0	1	0	0
0	0	0	1	0	0
0	1	0	1	1	0
0	1	0	1	1	0
0	0	0	1	0	0
0	0	1	1	1	0
0	0	1	1	0	0
0	0	0	1	0	0
1	0	0	1	0	0
0	0	0	1	0	0
0	0	0	1	1	0
99	0	0	1	1	0
0	0	0	1	1	0
0	0	0	1	1	0
0	0	0	1	1	0
99	0	0	1	1	0
0	0	0	1	1	0
0	0	99	1	1	0
0	0	0	1	1	0
0	1	1	1	0	0
0	0	0	1	0	0
0	0	0	1	0	0
99	99	99	1	1	0
0	0	0	1	1	0
0	0	0	1	0	0
0	0	1	1	1	0
0	0	99	1	0	0
1	1	0	1	0	0
0	0	0	1	0	0
0	0	99	1	0	0
0	0	0	1	0	0
0	0	0	1	1	0
0	0	0	1	1	0
0	1	0	1	0	0
0	0	0	1	0	0
1	1	1	1	1	0
0	0	0	1	0	0
0	0	0	0	1	0
0	0	0	0	1	0
1	1	1	1	1	0
0	0	0	1	0	0
0	99	0	1	1	0

0	0	0	0	1	0
0	0	0	1	1	0
0	0	0	1	1	0
0	0	0	1	0	0
0	0	1	1	0	0
0	0	0	1	0	0
0	0	0	1	0	0
99	1	0	1	0	0
1	99	1	1	1	0
99	99	99	1	0	0
1	1	1	1	0	0
0	0	0	1	1	0
0	0	0	1	0	0
0	0	0	1	1	0
0	0	0	1	0	0
1	0	1	1	0	0
0	0	0	1	1	0
1	1	1	1	0	0
1	1	0	1	0	0
0	0	0	1	0	0
1	1	0	1	0	0
0	0	0	1	1	0
99	99	0	1	1	0
0	1	0	1	0	0
0	0	0	1	0	0
0	0	0	1	0	0
0	0	0	1	1	0
99	99	99	1	0	0
0	0	0	1	1	0
0	0	0	1	0	0
99	99	99	1	0	0
0	0	0	1	1	0
1	1	0	1	0	0
0	0	0	1	1	0
0	0	99	0	1	0
0	0	0	1	1	0
1	1	0	1	0	0
0	0	0	1	1	0
0	0	0	1	1	0
99	0	0	1	1	0
99	0	0	0	1	0
1	1	1	1	0	0
0	0	0	1	1	0
0	0	0	1	1	0
0	0	0	1	1	0
0	0	0	1	0	0
0	0	0	0	1	0
0	1	0	1	1	0

0	0	0	1	1	0
1	1	1	1	0	0
0	0	0	0	1	0
0	0	0	1	1	0
0	0	0	1	1	0
1	0	1	1	0	0
0	0	0	0	1	0
1	1	1	1	0	0
0	0	0	1	1	0
0	0	0	1	1	0
1	0	1	1	1	0
0	0	0	1	0	0
0	0	0	1	0	0
1	1	1	1	0	0
0	0	0	1	1	0
0	0	0	1	1	0
1	0	0	1	0	0
1	99	99	1	0	0
0	0	0	0	1	0
0	1	0	1	0	0
0	0	1	1	0	0
0	0	0	1	0	0
0	1	1	1	1	0
0	1	0	1	0	0
0	0	0	1	0	0
0	0	1	1	1	0
0	0	0	1	1	0
1	0	0	1	0	0
0	0	0	1	1	0
0	0	1	1	0	0
0	1	0	1	1	0
0	0	0	1	0	0
0	0	0	1	0	0
0	1	99	1	0	0
0	0	0	1	0	0
99	99	99	1	0	0
0	0	0	0	1	0
0	1	0	1	0	0
0	0	0	1	1	0
0	0	0	1	1	0
0	0	0	1	1	0
0	0	0	1	0	0
1	1	1	1	0	0
1	1	0	1	1	0
0	1	1	1	0	0
1	0	0	1	1	0
0	0	0	1	1	0
99	99	99	1	0	0

0	0	0	1	0	0
0	0	1	1	0	0
0	0	0	1	0	0
0	0	0	1	0	0
1	1	0	1	0	0
1	0	0	1	0	0
0	0	0	1	0	0
0	0	0	1	0	0
0	0	0	1	0	0
0	0	0	1	1	0
0	0	0	1	1	0
0	0	1	1	0	0
1	1	0	1	1	0
0	0	0	1	1	0
0	0	0	1	0	0
0	0	0	1	0	0
0	0	0	1	1	0
0	0	0	1	1	0
99	0	99	0	1	0
0	0	0	0	1	0
1	1	1	1	1	0
1	0	0	1	0	0
0	0	0	1	1	0
0	0	0	1	1	0
0	0	0	1	1	0
0	99	0	1	1	0
1	0	0	1	1	0
0	0	0	1	0	0
0	1	1	0	0	0
1	0	1	1	0	0
0	0	0	1	0	0
0	99	0	1	1	0
0	0	0	0	1	0
0	0	0	1	1	0
0	0	0	1	1	0
0	0	0	0	1	0
0	0	0	1	1	0
0	0	0	1	1	0
0	0	0	1	1	0
1	0	0	1	1	0
0	0	0	1	1	0
0	0	0	1	0	0
0	1	1	1	0	0
0	0	99	1	0	0
0	1	0	1	0	0
1	1	0	1	0	0
0	0	0	1	0	0
1	0	1	1	1	0

99	0	0	1	0	0
0	0	0	1	1	0
0	0	0	1	0	0
0	1	0	1	1	0
0	0	0	1	1	0
99	0	99	0	1	0
0	0	0	1	0	0
0	0	0	1	0	0
0	0	0	1	1	0
0	0	0	1	1	0
0	0	0	1	0	0
1	0	1	1	0	0
0	0	0	1	1	0
1	1	1	1	1	0
0	0	0	1	0	0
0	0	0	1	0	0
1	0	1	1	0	0
0	0	0	1	1	0
99	99	99	1	1	0
0	0	0	1	1	0
1	0	0	1	0	0
0	0	0	1	1	0
0	1	0	1	0	0
0	0	0	1	0	0
0	0	0	1	0	0
1	1	1	1	0	0
0	0	0	1	1	0
0	0	1	0	1	0
0	0	99	1	0	0
0	1	0	1	1	0
1	1	1	1	1	0
1	1	99	1	1	0
0	1	0	1	1	0
0	0	0	1	0	0
1	0	0	1	0	0
0	0	0	1	1	0
0	0	0	1	1	0
0	0	0	1	1	0
0	0	0	1	1	0
0	0	1	0	1	0
0	0	0	1	1	0
1	1	0	0	1	0
1	0	0	1	0	0
0	0	0	1	1	0
1	0	1	1	1	0
0	0	0	1	0	0
1	0	0	1	0	0
0	0	0	1	1	0

0	0	0	1	0	0
1	0	0	1	1	0
1	0	1	1	0	0
0	1	0	1	0	0
1	1	0	0	0	0
1	0	0	0	1	0
0	0	0	1	1	0
0	0	0	1	0	0
0	0	0	1	0	0
0	0	0	1	0	0
0	0	1	1	0	0
1	0	0	1	0	0
0	1	0	1	0	0
0	0	0	0	1	0
0	0	0	0	1	0
0	0	0	1	0	0
0	0	0	1	1	0
0	1	0	1	1	0
0	0	0	1	1	0
1	0	0	0	1	0
0	0	0	0	1	0
0	1	0	1	0	0
0	0	0	1	1	0
99	99	99	1	1	0
0	0	1	1	0	0
0	0	0	1	1	0
0	0	0	1	0	0
99	99	0	1	0	0
0	0	0	1	1	0
0	0	0	1	0	0
0	1	1	1	0	0
0	0	0	0	1	0
0	0	1	0	1	0
1	1	0	1	0	0
0	0	0	1	0	0
0	0	0	1	0	0
1	1	1	1	1	0
1	0	0	1	1	0
0	0	1	1	1	0
0	0	0	1	0	0
0	0	0	1	0	0
0	1	0	1	0	0
0	0	0	1	1	0
0	0	0	1	1	0
0	0	0	1	1	0
0	0	0	1	1	0
0	0	0	1	1	0
0	0	0	1	1	0

0	0	0	1	0	0
0	0	0	1	1	0
0	0	0	1	0	0
0	0	0	0	1	0
0	0	0	1	1	0
0	0	0	1	1	0
0	0	0	1	1	0
0	1	99	1	0	0
0	0	0	1	0	0
0	0	1	1	0	0
0	1	1	1	1	0
0	0	0	1	1	0
0	0	0	0	1	0
0	0	0	1	1	0
1	1	0	1	0	0
0	0	0	1	0	0
1	1	1	1	0	0
0	0	0	1	1	0
0	0	0	1	1	0
1	0	0	1	1	0
0	0	0	1	1	0
0	0	0	1	1	0
0	0	0	0	1	0
1	0	1	1	1	0
0	1	0	1	0	0
0	0	0	1	1	0
0	1	0	1	1	0
0	0	0	1	0	0
1	0	0	1	0	0
0	1	0	1	1	0
0	0	0	1	0	0
0	0	0	1	0	0
0	0	0	1	1	0
1	0	0	1	0	0
0	0	0	1	0	0
1	0	0	1	1	0
0	0	0	0	1	0
0	0	0	1	0	0
0	0	0	1	0	0
0	0	0	1	0	0
0	0	0	1	1	0
99	1	99	1	0	0
0	0	0	1	0	0
0	1	0	1	1	0
99	1	99	1	0	0
0	0	0	1	0	0
1	0	99	1	1	0
1	0	0	1	1	0

0	0	1	1	0	0
0	0	0	0	1	0
0	0	0	1	0	0
0	1	0	1	0	0
0	0	0	1	0	0
0	0	0	1	1	0
0	0	0	1	0	0
0	0	0	1	1	0
0	0	0	1	0	0
0	0	0	1	1	0
99	99	99	1	1	1
0	1	0	1	0	0
1	0	99	1	0	0
0	0	0	1	1	0
0	1	0	1	1	0
1	1	0	1	0	0
0	0	0	1	0	0
1	1	1	1	0	0
0	0	0	1	0	0
0	0	0	1	0	0
0	0	0	1	0	0
1	0	0	1	1	0
0	0	0	1	0	0
0	0	0	1	1	0
0	0	0	1	1	0
0	0	0	1	0	0
0	0	0	1	1	0
0	0	0	1	0	0
0	0	0	1	1	0
0	0	0	1	0	0
0	0	1	1	0	0
0	0	0	1	1	0
1	1	0	1	0	0
0	1	99	1	1	0
99	99	99	1	0	0
1	0	1	1	1	0
0	0	0	0	1	0
1	1	0	1	0	0
0	0	0	1	0	0
0	0	0	1	0	0
0	0	0	1	1	0
0	0	0	1	0	0
1	1	1	1	0	0
0	0	0	0	1	0
1	1	0	1	0	0
0	0	0	1	1	0
0	0	0	1	1	0
1	0	1	1	1	0

0	0	0	1	0	0
0	0	1	1	0	0
0	0	0	1	0	0
0	0	0	1	0	0
0	0	0	1	1	0
0	0	1	0	1	0
1	0	1	1	0	0
0	0	0	1	0	0
0	0	0	1	0	0
0	0	0	1	0	0
0	1	0	1	1	0
0	1	0	1	0	0
0	0	0	1	0	0
0	0	0	1	0	0
1	0	0	1	1	0
0	0	0	1	0	0
0	0	0	1	0	0
0	0	0	1	1	0
0	0	0	1	0	0
0	0	0	1	1	0
0	0	0	1	1	0
1	0	1	1	1	0
0	0	0	1	1	0
0	0	0	1	0	0
0	0	0	1	1	0
1	1	1	1	0	0
0	1	0	1	0	0
99	1	99	1	0	0
0	0	0	1	1	0
0	0	0	1	0	0
0	0	0	1	1	0
0	0	1	1	1	0
0	0	0	1	0	0
0	0	99	1	0	0
0	1	0	1	0	0
0	0	0	1	1	0
0	0	1	0	1	0
0	0	0	1	0	0
0	0	0	1	1	0
0	0	0	1	0	0
0	1	1	1	0	0
0	0	0	1	1	0
0	1	0	1	0	0
1	1	0	1	0	0
0	0	0	1	1	0
1	1	1	0	1	0
1	0	0	1	0	0
0	0	0	1	0	0

0	0	0	1	0	0
0	1	99	1	0	0
0	0	0	1	1	0
0	0	0	1	0	0
0	0	0	1	0	0
0	1	1	1	0	0
0	0	0	1	1	0
0	0	0	1	1	0
1	0	0	1	1	0
0	1	0	1	1	0
99	0	99	1	0	0
1	99	1	1	0	0
0	0	0	1	0	0
0	0	0	1	1	0
0	1	0	0	0	0
0	0	0	0	1	0
0	1	1	1	0	0
0	1	0	1	1	0
0	0	99	0	1	0
0	0	0	1	1	0
1	1	0	1	1	0
0	0	0	1	0	0
0	0	0	1	1	0
0	0	1	1	1	0
0	0	0	1	1	0
0	0	0	1	1	0
0	1	0	0	1	0
0	0	0	0	1	0
0	0	0	1	1	0
1	0	0	1	1	0
0	1	0	1	0	0
0	0	0	1	0	0
1	0	0	1	0	0
1	0	0	1	0	0
0	1	0	1	1	0
0	0	0	1	0	0
0	0	0	1	1	0
0	0	0	1	0	0
0	1	1	1	0	0
0	0	0	1	0	0
0	0	0	1	0	0
1	1	1	1	0	0
0	0	0	1	1	0
0	0	0	1	0	0
1	1	0	1	0	0
0	0	0	1	1	0
0	0	0	1	0	0
0	0	0	1	0	0

0	0	0	1	0	0
1	0	0	1	1	0
0	0	0	1	1	0
0	0	0	1	1	0
0	0	0	1	0	0
0	0	0	1	0	0
0	0	0	1	1	0
1	0	1	1	0	0
1	1	0	1	0	0
1	1	0	0	0	0
0	0	0	1	1	0
0	0	0	1	0	0
0	0	0	1	0	0
0	0	0	1	0	0
0	0	0	1	0	0
0	0	0	1	0	0
0	0	0	1	1	0
0	0	0	1	1	0
0	0	0	1	0	0
0	0	0	1	0	0
0	0	0	1	1	0
0	0	0	1	0	0
0	1	0	1	0	0
0	0	0	1	0	0
0	0	0	1	1	0
0	0	0	1	1	0
0	0	0	1	0	0
0	0	0	1	1	0
1	1	0	1	0	0
0	0	0	1	1	0
0	0	0	1	1	0
0	0	0	1	1	0
0	0	0	1	1	0
1	0	0	1	1	0
0	0	0	1	0	0
1	0	1	1	0	0
0	0	0	0	1	0
0	0	0	1	0	0
0	0	1	1	1	0
0	0	0	1	1	0
0	0	0	0	0	1
0	0	0	0	1	0
0	0	0	1	0	0
0	0	0	0	1	0
0	0	#NULL!	1	0	0
0	0	0	1	0	0
0	0	0	1	0	0
0	0	0	0	1	0

0	0	0	1	1	0
1	0	0	1	0	0
0	0	0	1	0	0
1	0	1	1	0	0
0	0	0	1	0	0
0	0	0	1	0	0
0	1	1	1	0	0
0	0	0	1	0	0
0	0	0	1	1	0
0	0	0	1	0	0
0	0	0	0	1	0
1	1	1	1	0	0
#NULL!	#NULL!	#NULL!	0	0	0
1	0	0	1	0	0
0	0	0	1	0	0
0	0	#NULL!	1	1	0
0	0	0	1	0	0
0	0	0	1	1	0
0	0	0	0	1	0
0	0	0	1	1	0
0	0	#NULL!	1	1	0
0	0	0	1	1	0
0	0	0	0	1	0
0	0	0	1	1	0
0	0	0	0	0	0
0	0	0	1	1	0
0	0	0	1	0	0
1	0	0	1	0	0
0	0	0	1	1	0
0	0	0	1	1	0
0	0	0	1	1	0
0	0	#NULL!	1	0	0
0	1	0	1	0	0
0	0	0	1	1	0
0	0	0	1	0	0
0	0	#NULL!	0	1	0
0	0	#NULL!	1	0	0
0	0	0	1	1	0
0	0	0	1	1	0
0	1	0	1	0	0
0	0	0	0	1	0
0	0	0	1	1	0
0	0	0	0	1	0
0	0	0	1	0	0
0	1	1	1	0	0
0	0	1	1	0	0
0	0	0	1	1	0
0	0	0	1	1	0

0	1	0	1	0	0
0	1	1	1	1	0
0	0	1	1	0	0
0	0	0	0	0	0
0	1	0	1	1	0
0	0	0	1	1	0
0	1	0	1	1	0
0	0	0	1	1	0
0	0	0	1	1	0
0	0	0	1	0	0
0	1	0	1	0	0
0	0	0	1	0	0
1	0	0	1	0	0
0	0	0	0	1	0
0	0	0	1	1	0
0	0	0	1	1	0
#NULL!	#NULL!	#NULL!	0	0	0
1	0	1	1	0	0
0	1	0	1	0	0
0	0	0	1	1	0
0	0	0	1	1	0

language_pre___4	language_pre___5	other_language_pre	langa_pre	langb_pre	subusea_pre	subuseb_pre
0	0	#NULL!	3	2	0	0
0	0	#NULL!	3	3	0	0
0	0	#NULL!	3	1	0	0
0	0	#NULL!	1	1	0	0
0	0	#NULL!	1	1	0	0
0	0	#NULL!	4	1	0	0
0	0	#NULL!	2	1	3	4
0	0	#NULL!	3	3	0	99
0	0	#NULL!	3	3	99	99
0	0	#NULL!	1	1	0	1
0	0	#NULL!	1	1	0	0
0	0	#NULL!	1	1	1	0
0	0	#NULL!	1	1	0	1
0	0	#NULL!	1	1	0	0
0	0	#NULL!	1	2	0	0
0	0	#NULL!	5	2	0	0
0	0	#NULL!	3	1	0	0
0	0	#NULL!	2	3	0	99
0	0	#NULL!	3	1	0	0
0	0	#NULL!	1	2	2	1
0	0	#NULL!	3	2	0	99
0	1	#NULL!	99	99	0	0
0	0	#NULL!	3	3	0	0
0	0	#NULL!	4	2	0	0
0	0	#NULL!	3	1	0	0
0	0	#NULL!	4	1	0	0
0	0	#NULL!	3	2	0	0
0	0	#NULL!	1	1	0	0
0	0	#NULL!	2	1	0	0
0	0	#NULL!	3	2	0	0
1	0	#NULL!	3	3	0	0
0	0	#NULL!	1	1	1	5
0	0	#NULL!	2	2	0	0
0	0	#NULL!	3	3	0	0
0	0	#NULL!	1	99	0	0
0	0	#NULL!	3	1	0	0
0	0	#NULL!	3	1	0	0
0	0	#NULL!	3	1	0	0
1	0	#NULL!	3	1	2	0
0	0	#NULL!	2	2	2	3
0	0	#NULL!	3	2	0	0
0	0	#NULL!	1	1	0	0
0	0	#NULL!	1	1	0	0
0	0	#NULL!	1	1	0	0
0	0	#NULL!	1	1	0	0
0	0	#NULL!	1	1	0	0
0	0	#NULL!	1	2	1	1

0	0	#NULL!	3	3	2	3
0	0	#NULL!	1	1	0	0
0	0	#NULL!	3	2	0	0
0	0	#NULL!	1	1	0	1
0	0	#NULL!	3	2	1	0
0	0	#NULL!	1	2	0	0
0	0	#NULL!	1	1	0	0
0	0	#NULL!	4	4	0	1
0	0	#NULL!	4	3	0	3
1	0	#NULL!	4	1	1	1
0	0	#NULL!	3	3	0	0
0	0	#NULL!	1	1	0	0
0	0	#NULL!	3	3	0	0
1	0	#NULL!	2	1	3	4
0	0	#NULL!	2	2	0	1
1	0	#NULL!	2	2	0	0
0	0	#NULL!	1	1	0	0
0	0	#NULL!	3	3	5	5
0	0	#NULL!	1	1	0	0
0	0	#NULL!	1	2	0	1
0	0	#NULL!	3	3	0	5
0	0	#NULL!	3	2	0	0
0	0	#NULL!	1	1	0	0
0	0	#NULL!	1	2	1	0
0	0	#NULL!	1	1	0	0
0	0	#NULL!	3	1	0	0
0	0	#NULL!	1	1	0	0
0	0	#NULL!	3	3	0	5
0	0	#NULL!	3	2	5	5
0	0	#NULL!	3	2	0	0
0	0	#NULL!	1	2	0	0
0	0	#NULL!	3	2	0	1
0	0	#NULL!	1	1	0	0
0	0	#NULL!	1	1	0	0
0	0	#NULL!	3	2	0	2
0	0	#NULL!	3	3	0	0
0	0	#NULL!	2	4	0	0
0	0	#NULL!	3	3	1	1
0	0	#NULL!	4	3	0	2
0	0	#NULL!	1	1	0	0
0	0	#NULL!	4	3	0	2
0	0	#NULL!	3	2	3	5
0	0	#NULL!	3	3	2	3
0	0	#NULL!	3	3	0	0
0	0	#NULL!	3	3	0	0
0	0	#NULL!	1	1	1	1
0	0	#NULL!	1	1	0	0
0	0	#NULL!	3	3	0	0

0	0	#NULL!	1	1	0	0
0	0	#NULL!	3	2	4	4
0	0	#NULL!	4	2	0	1
0	0	#NULL!	2	1	0	0
0	0	#NULL!	2	2	0	0
0	0	#NULL!	1	1	0	0
0	0	#NULL!	2	2	1	5
0	0	#NULL!	2	1	0	0
0	0	#NULL!	4	2	0	1
0	0	#NULL!	1	1	2	2
0	0	#NULL!	2	2	1	1
1	0	#NULL!	3	3	0	0
0	0	#NULL!	1	1	1	2
0	0	#NULL!	1	2	3	3
0	0	#NULL!	2	1	0	0
0	0	#NULL!	4	3	0	5
0	0	#NULL!	1	1	0	0
0	0	#NULL!	3	2	0	0
0	0	#NULL!	3	2	0	0
0	0	#NULL!	3	3	0	0
0	0	#NULL!	5	3	1	2
0	0	#NULL!	1	1	0	2
0	1	#NULL!	1	5	0	0
0	0	#NULL!	1	1	0	0
0	0	#NULL!	3	1	0	0
0	0	#NULL!	4	2	1	4
0	0	#NULL!	4	1	0	0
0	0	#NULL!	1	1	0	0
0	0	#NULL!	3	3	0	0
0	0	#NULL!	3	2	0	0
0	0	#NULL!	3	2	0	0
0	0	#NULL!	1	1	0	0
0	0	#NULL!	3	3	0	0
0	0	#NULL!	1	1	0	0
0	0	#NULL!	2	2	0	0
0	0	#NULL!	3	2	0	0
0	0	#NULL!	3	3	0	0
0	0	#NULL!	3	3	0	0
0	0	#NULL!	3	1	0	0
0	0	#NULL!	1	1	0	0
0	0	#NULL!	1	1	0	0
0	0	#NULL!	2	1	0	0
0	0	#NULL!	3	2	0	1
0	0	#NULL!	1	1	0	0
0	0	#NULL!	1	1	0	0
0	0	#NULL!	1	1	1	1
0	0	#NULL!	1	1	0	0
0	0	#NULL!	2	4	0	0

0	0	#NULL!	1	1	0	0
0	0	#NULL!	3	3	0	1
0	0	#NULL!	1	1	0	0
0	0	#NULL!	1	1	1	1
0	0	#NULL!	1	1	1	2
0	0	#NULL!	1	1	0	0
0	0	#NULL!	4	4	0	0
0	0	#NULL!	3	2	0	1
0	0	#NULL!	5	3	0	0
0	0	#NULL!	1	1	0	0
0	0	#NULL!	2	3	0	0
0	1	#NULL!	5	5	0	0
0	0	#NULL!	3	1	0	0
0	0	#NULL!	1	1	0	0
0	0	#NULL!	5	4	0	0
0	0	#NULL!	3	2	0	0
0	0	#NULL!	1	1	0	0
0	0	#NULL!	3	1	0	0
0	0	#NULL!	3	3	0	0
0	0	#NULL!	3	3	0	0
0	0	#NULL!	4	1	0	0
0	0	#NULL!	4	1	0	0
0	0	#NULL!	1	1	0	0
0	0	#NULL!	1	1	0	0
0	0	#NULL!	3	1	0	0
0	0	#NULL!	1	1	0	0
0	0	#NULL!	2	2	0	0
0	0	#NULL!	1	1	0	0
0	0	#NULL!	1	1	0	0
0	0	#NULL!	1	1	0	0
0	0	#NULL!	1	1	0	0
0	0	#NULL!	2	1	0	0
0	0	#NULL!	2	1	0	0
0	0	#NULL!	4	1	0	0
0	0	#NULL!	1	1	1	1
0	0	#NULL!	1	2	0	0
0	0	#NULL!	1	1	0	0
0	0	#NULL!	99	1	0	0
0	0	#NULL!	1	1	0	0
0	0	#NULL!	1	1	0	0
0	0	#NULL!	2	2	0	0
0	0	#NULL!	5	3	0	0
0	0	#NULL!	1	1	0	0
0	0	#NULL!	1	2	0	0
0	0	#NULL!	1	1	0	0
0	0	#NULL!	1	1	0	0
0	0	#NULL!	1	1	0	0
0	0	#NULL!	1	1	0	0

0	0	#NULL!	4	2	0	0
0	0	#NULL!	1	1	0	0
0	0	#NULL!	1	1	0	0
0	0	#NULL!	4	2	0	2
0	0	#NULL!	1	1	0	1
0	0	#NULL!	1	1	0	0
0	0	#NULL!	1	1	0	0
0	0	#NULL!	1	1	2	2
0	0	#NULL!	3	3	0	0
0	0	#NULL!	1	1	0	0
0	0	#NULL!	2	3	0	0
0	0	#NULL!	4	2	2	2
0	0	#NULL!	1	1	0	0
0	0	#NULL!	1	1	2	1
0	0	#NULL!	3	2	0	0
0	0	#NULL!	1	1	0	2
0	0	#NULL!	1	1	0	0
0	0	#NULL!	2	2	1	1
0	0	#NULL!	1	1	0	0
0	0	#NULL!	4	4	0	0
0	0	#NULL!	1	1	0	0
0	0	#NULL!	3	3	0	0
0	0	#NULL!	3	4	0	0
0	0	#NULL!	3	3	0	0
0	0	#NULL!	1	2	2	99
0	0	#NULL!	1	1	1	0
0	0	#NULL!	1	1	0	0
0	0	#NULL!	2	1	1	0
0	0	#NULL!	3	2	2	2
0	0	#NULL!	2	1	0	0
0	0	#NULL!	3	3	0	0
0	0	#NULL!	4	1	0	1
0	0	#NULL!	4	4	0	3
0	0	#NULL!	3	1	0	0
0	0	#NULL!	2	1	2	2
0	0	#NULL!	1	1	0	2
0	0	#NULL!	3	3	1	0
0	0	#NULL!	3	2	0	1
0	0	#NULL!	3	2	0	0
0	0	#NULL!	4	3	0	0
0	0	#NULL!	1	99	1	3
0	0	#NULL!	2	2	0	0
0	0	#NULL!	1	1	0	0
0	0	#NULL!	1	1	0	0
0	0	#NULL!	1	1	0	0
0	0	#NULL!	1	1	1	0
0	0	#NULL!	1	1	0	0
0	0	#NULL!	3	1	0	0

0	0	#NULL!	1	3	0	0
0	0	#NULL!	3	3	0	0
0	0	#NULL!	1	1	0	0
0	0	#NULL!	4	2	0	0
1	0	#NULL!	99	99	1	2
0	0	#NULL!	3	3	0	0
0	0	#NULL!	3	3	0	0
0	0	#NULL!	1	1	0	0
0	0	#NULL!	1	3	0	0
0	0	#NULL!	3	3	0	0
0	0	#NULL!	1	1	0	0
0	0	#NULL!	1	99	0	0
0	0	#NULL!	3	2	0	0
0	0	#NULL!	99	1	0	2
0	0	#NULL!	3	1	0	0
0	0	#NULL!	2	1	1	2
0	0	#NULL!	2	2	0	0
0	0	#NULL!	1	1	0	0
0	0	#NULL!	1	1	0	0
0	0	#NULL!	1	1	0	0
0	0	#NULL!	3	3	1	0
0	0	#NULL!	1	1	0	0
0	0	#NULL!	5	2	0	1
0	0	#NULL!	1	1	0	0
0	0	#NULL!	1	3	0	0
0	0	#NULL!	1	1	0	0
0	0	#NULL!	1	2	0	0
0	0	#NULL!	3	3	0	0
0	0	#NULL!	1	1	0	0
0	0	#NULL!	3	2	0	0
0	0	#NULL!	3	3	0	1
0	0	#NULL!	4	3	0	1
0	0	#NULL!	3	2	0	0
0	0	#NULL!	5	2	0	0
0	0	#NULL!	1	1	1	5
0	0	#NULL!	4	1	1	5
0	0	#NULL!	3	2	0	0
0	0	#NULL!	1	1	0	0
0	0	#NULL!	1	1	0	0
0	0	#NULL!	3	2	1	0
0	0	#NULL!	3	2	3	2
0	0	#NULL!	3	2	0	0
0	0	#NULL!	3	3	0	0
0	0	#NULL!	3	3	0	0
0	0	#NULL!	3	3	0	0
0	0	#NULL!	2	2	0	0
0	0	#NULL!	1	1	0	0
0	0	#NULL!	3	2	0	0

0	0	#NULL!	2	2	0	0
0	0	#NULL!	1	1	0	0
0	0	#NULL!	1	1	0	0
0	0	#NULL!	3	2	0	0
0	0	#NULL!	1	1	0	0
0	0	#NULL!	2	1	0	0
0	0	#NULL!	1	1	2	0
0	0	#NULL!	1	1	0	0
0	0	#NULL!	2	2	3	4
0	0	#NULL!	4	2	0	3
0	0	#NULL!	1	1	1	1
0	0	#NULL!	3	4	0	1
0	0	#NULL!	3	2	0	0
0	0	#NULL!	2	2	0	0
0	0	#NULL!	1	2	0	1
0	0	#NULL!	1	1	0	0
0	0	#NULL!	3	3	0	2
0	0	#NULL!	4	3	0	0
0	0	#NULL!	4	3	0	0
0	0	#NULL!	1	1	0	0
0	0	#NULL!	1	1	0	0
0	0	#NULL!	1	1	5	5
0	0	#NULL!	3	3	0	2
1	0	#NULL!	3	1	1	4
0	0	#NULL!	4	2	0	0
0	0	#NULL!	3	3	0	0
0	0	#NULL!	1	2	5	5
0	0	#NULL!	3	4	0	0
0	0	#NULL!	1	1	0	0
0	0	#NULL!	2	2	0	0
0	0	#NULL!	1	1	0	0
0	0	#NULL!	1	2	0	0
0	0	#NULL!	1	1	1	1
0	0	#NULL!	3	2	99	99
0	0	#NULL!	1	1	0	0
0	0	#NULL!	1	1	1	5
0	0	#NULL!	3	3	1	3
0	0	#NULL!	3	3	0	0
0	0	#NULL!	3	3	0	0
0	0	#NULL!	3	3	0	0
0	0	#NULL!	4	3	3	5
0	0	#NULL!	3	2	0	2
0	0	#NULL!	4	2	1	1
0	0	#NULL!	4	4	0	0
0	0	#NULL!	1	1	0	0
0	0	#NULL!	3	3	2	2
0	0	#NULL!	3	3	1	1
0	0	#NULL!	3	2	0	0

0	0	#NULL!	3	3	0	0
0	0	#NULL!	3	2	0	1
1	0	#NULL!	3	3	0	0
0	0	#NULL!	1	2	0	0
0	0	#NULL!	3	3	0	1
0	0	#NULL!	1	1	0	0
0	0	#NULL!	2	2	2	5
0	0	#NULL!	1	1	1	4
0	0	#NULL!	1	1	2	5
0	0	#NULL!	2	3	0	4
0	0	#NULL!	1	1	1	0
0	0	#NULL!	5	3	0	0
0	0	#NULL!	1	1	4	2
0	0	#NULL!	1	2	0	3
0	0	#NULL!	3	3	0	0
0	0	#NULL!	3	3	1	2
1	0	#NULL!	4	2	0	0
0	0	#NULL!	4	2	0	0
0	0	#NULL!	3	2	0	1
0	0	#NULL!	3	3	0	2
0	0	#NULL!	3	2	0	0
0	0	#NULL!	2	1	0	0
0	0	#NULL!	1	1	0	0
0	0	#NULL!	2	1	3	3
0	0	#NULL!	3	1	0	0
0	0	#NULL!	1	1	0	0
0	0	#NULL!	1	1	0	0
0	0	#NULL!	1	1	0	0
0	0	#NULL!	3	3	0	0
0	0	#NULL!	1	2	2	2
0	0	#NULL!	1	1	0	99
0	0	#NULL!	1	1	0	0
0	0	#NULL!	3	2	0	0
0	0	#NULL!	1	1	0	0
1	0	#NULL!	4	1	0	0
0	0	#NULL!	4	2	0	0
0	0	#NULL!	1	1	0	0
0	0	#NULL!	1	1	0	0
0	0	#NULL!	3	3	0	0
0	0	#NULL!	1	1	0	0
0	0	#NULL!	1	2	0	99
0	0	#NULL!	3	2	0	1
0	0	#NULL!	2	1	0	0
0	0	#NULL!	3	3	0	1
0	0	#NULL!	1	1	0	0
0	0	#NULL!	1	1	0	0
0	0	#NULL!	1	1	0	0
0	0	#NULL!	4	1	0	0

0	0	#NULL!	1	1	0	0
0	0	#NULL!	3	3	0	0
0	0	#NULL!	3	3	0	1
0	0	#NULL!	2	1	1	2
0	0	#NULL!	1	1	0	0
0	0	#NULL!	1	1	3	4
0	0	#NULL!	1	1	0	0
0	0	#NULL!	1	1	3	4
0	0	#NULL!	4	2	0	0
0	0	#NULL!	1	4	0	0
0	0	#NULL!	1	1	0	0
0	0	#NULL!	1	1	0	1
0	0	#NULL!	4	2	0	2
0	0	#NULL!	3	2	0	0
0	0	#NULL!	1	1	0	0
0	0	#NULL!	1	1	0	0
0	0	#NULL!	1	3	5	5
0	0	#NULL!	1	1	0	0
0	0	#NULL!	3	2	0	0
0	0	#NULL!	1	1	0	0
0	0	#NULL!	3	2	0	1
0	1	#NULL!	2	2	0	0
0	0	#NULL!	4	2	1	4
0	0	#NULL!	4	2	0	0
0	0	#NULL!	3	2	0	0
0	0	#NULL!	1	1	2	1
0	0	#NULL!	4	3	0	1
0	0	#NULL!	1	1	0	0
0	0	#NULL!	4	3	0	0
0	0	#NULL!	5	5	0	0
0	0	#NULL!	3	3	1	1
0	0	#NULL!	3	1	0	0
1	0	#NULL!	2	1	0	0
0	0	#NULL!	3	3	0	0
0	0	#NULL!	5	5	0	0
0	0	#NULL!	4	3	0	0
0	0	#NULL!	1	1	0	0
1	0	#NULL!	1	1	0	1
0	0	#NULL!	4	4	0	0
1	0	#NULL!	4	3	0	0
0	0	#NULL!	1	1	0	0
0	0	#NULL!	3	2	2	0
0	0	#NULL!	3	3	0	2
0	0	#NULL!	1	1	0	1
0	0	#NULL!	1	1	0	1
0	0	#NULL!	3	2	1	0
0	0	#NULL!	1	1	0	1
0	0	#NULL!	2	2	3	2

0	0	#NULL!	4	2	0	0
0	0	#NULL!	1	1	0	0
0	0	#NULL!	4	3	0	0
0	0	#NULL!	4	1	0	0
0	0	#NULL!	3	2	0	0
0	0	#NULL!	3	2	1	1
0	0	#NULL!	2	1	3	5
0	0	#NULL!	3	2	1	2
0	0	#NULL!	1	1	2	3
0	0	#NULL!	3	3	0	3
0	0	#NULL!	1	1	1	1
0	0	#NULL!	3	3	5	5
0	0	#NULL!	1	1	0	0
0	0	#NULL!	2	3	0	0
0	0	#NULL!	3	3	0	0
0	0	#NULL!	1	1	5	3
0	0	#NULL!	3	1	0	1
0	0	#NULL!	1	3	0	0
0	0	#NULL!	1	1	0	0
0	0	#NULL!	3	3	0	0
0	0	#NULL!	1	1	0	0
0	0	#NULL!	2	3	5	5
0	0	#NULL!	2	3	2	3
0	0	#NULL!	4	3	0	4
0	0	#NULL!	1	1	1	3
0	0	#NULL!	4	2	0	3
0	0	#NULL!	1	1	5	5
0	0	#NULL!	3	2	0	0
0	0	#NULL!	3	2	0	0
0	0	#NULL!	3	3	4	5
0	0	#NULL!	3	2	0	0
0	0	#NULL!	4	2	3	3
0	0	#NULL!	3	3	1	1
0	0	#NULL!	1	1	0	0
0	0	#NULL!	2	2	0	2
0	0	#NULL!	4	3	0	0
0	0	#NULL!	3	2	0	1
0	0	#NULL!	4	2	0	0
0	0	#NULL!	3	3	0	0
0	0	#NULL!	1	1	0	0
0	0	#NULL!	3	2	0	0
0	0	#NULL!	1	1	0	0
0	0	#NULL!	5	5	0	0
0	0	#NULL!	1	1	2	2
0	0	#NULL!	1	1	0	0
0	0	#NULL!	4	99	0	0
0	0	#NULL!	3	2	0	1
0	0	#NULL!	99	1	0	0

0	0	#NULL!	4	3	0	0
0	0	#NULL!	2	3	0	0
0	0	#NULL!	2	2	0	0
0	0	#NULL!	4	2	0	0
0	0	#NULL!	1	2	5	3
0	0	#NULL!	1	1	0	0
0	0	#NULL!	1	3	0	0
0	0	#NULL!	1	2	0	2
0	0	#NULL!	1	2	1	1
0	0	#NULL!	1	1	2	2
0	0	#NULL!	2	2	0	0
0	0	#NULL!	1	1	0	0
0	0	#NULL!	3	2	0	0
0	0	#NULL!	1	1	0	0
0	0	#NULL!	3	2	0	0
0	0	#NULL!	3	2	0	0
0	0	#NULL!	4	1	0	0
0	0	#NULL!	3	1	0	0
0	0	#NULL!	2	2	0	0
0	0	#NULL!	3	3	0	1
0	0	#NULL!	4	2	0	1
0	0	#NULL!	3	1	0	1
0	0	#NULL!	2	1	0	0
0	0	#NULL!	4	3	0	2
0	0	#NULL!	99	1	99	99
0	0	#NULL!	1	1	0	1
0	0	#NULL!	2	1	0	0
0	0	#NULL!	5	4	0	0
0	0	#NULL!	1	1	0	0
0	0	#NULL!	4	1	0	1
0	0	#NULL!	1	2	1	0
0	0	#NULL!	99	99	0	0
0	0	#NULL!	1	1	0	0
0	0	#NULL!	5	3	0	0
0	0	#NULL!	99	99	0	0
0	0	#NULL!	5	3	0	0
0	0	#NULL!	4	1	1	2
0	0	#NULL!	3	99	0	0
0	0	#NULL!	4	2	0	0
0	0	#NULL!	5	3	0	0
0	0	#NULL!	1	1	0	0
0	0	#NULL!	5	3	0	0
0	0	#NULL!	1	1	0	0
0	0	#NULL!	4	3	0	0
0	0	#NULL!	1	1	1	1
0	0	#NULL!	1	1	0	0
0	0	#NULL!	3	2	0	0
0	0	#NULL!	3	2	0	0

0	0	#NULL!	3	2	0	0
0	0	#NULL!	4	3	0	1
0	0	#NULL!	3	3	0	2
0	0	#NULL!	1	1	0	1
0	0	#NULL!	1	1	1	0
0	0	#NULL!	1	1	0	0
0	0	#NULL!	1	1	0	0
0	0	#NULL!	2	2	0	0
0	0	#NULL!	1	1	0	0
0	0	#NULL!	1	1	0	0
0	0	#NULL!	3	2	0	0
0	0	#NULL!	3	4	0	0
0	0	#NULL!	1	1	0	0
0	0	#NULL!	1	1	1	1
0	0	#NULL!	3	2	0	1
0	0	#NULL!	4	3	0	1
0	0	#NULL!	4	2	0	0
0	0	#NULL!	1	2	0	0
0	0	#NULL!	4	3	0	0
0	0	#NULL!	3	2	0	0
0	0	#NULL!	1	1	0	0
0	0	#NULL!	3	2	0	0
0	0	#NULL!	1	1	0	0
0	0	#NULL!	1	2	1	0
0	0	#NULL!	1	1	1	2
0	0	#NULL!	3	2	0	0
0	0	#NULL!	3	2	0	0
0	0	#NULL!	3	3	0	1
0	0	#NULL!	3	2	0	0
0	0	#NULL!	1	1	0	0
0	0	#NULL!	2	1	0	0
0	0	#NULL!	3	2	0	5
0	0	#NULL!	2	2	0	0
0	0	#NULL!	1	1	1	2
0	0	#NULL!	3	3	0	0
0	0	#NULL!	5	4	0	0
0	0	#NULL!	3	2	0	0
0	0	#NULL!	1	1	0	0
0	0	#NULL!	2	2	0	0
0	0	#NULL!	4	2	0	2
0	0	#NULL!	1	1	0	0
0	0	#NULL!	1	1	0	0
0	0	#NULL!	3	3	0	2
0	0	#NULL!	1	99	0	0
0	0	#NULL!	3	2	0	0
0	0	#NULL!	1	1	0	0
0	0	#NULL!	3	2	0	0
0	0	#NULL!	4	1	0	0

0	0	#NULL!	2	2	0	2
0	0	#NULL!	99	99	0	0
0	0	#NULL!	5	3	1	3
0	0	#NULL!	3	2	0	2
0	0	#NULL!	1	1	0	0
0	0	#NULL!	3	2	0	0
0	1	#NULL!	3	3	0	0
0	0	#NULL!	3	2	0	0
0	0	#NULL!	3	4	0	1
0	0	#NULL!	4	2	0	0
0	0	#NULL!	1	1	1	3
0	0	#NULL!	1	2	0	0
0	0	#NULL!	1	2	0	0
0	0	#NULL!	3	1	0	0
0	0	#NULL!	1	1	0	0
0	0	#NULL!	1	1	0	0
0	0	#NULL!	1	1	0	0
0	0	#NULL!	1	1	0	0
0	0	#NULL!	1	1	0	0
0	0	#NULL!	1	1	0	0
0	0	#NULL!	3	1	0	0
0	0	#NULL!	3	3	0	0
0	0	#NULL!	3	1	0	0
0	0	#NULL!	1	1	0	0
0	0	#NULL!	2	2	0	0
0	0	#NULL!	2	2	0	0
0	1	#NULL!	1	1	0	0
0	0	#NULL!	3	1	0	0
0	0	#NULL!	3	3	0	0
0	0	#NULL!	1	1	0	0
0	0	#NULL!	99	99	99	0
0	0	#NULL!	1	1	0	0
0	0	#NULL!	5	1	0	0
0	0	#NULL!	3	3	0	0
0	0	#NULL!	1	1	0	0
0	0	#NULL!	3	3	0	0
0	0	#NULL!	3	3	0	0
0	0	#NULL!	3	2	0	0
0	0	#NULL!	3	3	0	0
0	0	#NULL!	1	1	0	0
0	0	#NULL!	3	3	0	0
0	0	#NULL!	1	1	0	0
0	0	#NULL!	3	3	0	0
0	0	#NULL!	1	1	0	0
0	0	#NULL!	2	2	0	0
0	0	#NULL!	1	1	0	0
0	0	#NULL!	3	3	0	0
0	0	#NULL!	3	2	0	0

0	0	#NULL!	3	1	0	0
0	0	#NULL!	4	3	0	0
0	0	#NULL!	3	1	0	0
0	0	#NULL!	3	3	0	0
0	0	#NULL!	1	1	0	0
0	0	#NULL!	1	1	0	0
0	0	#NULL!	3	3	0	0
0	0	#NULL!	1	1	0	0
0	0	#NULL!	1	1	0	0
0	0	#NULL!	2	1	0	0
0	0	#NULL!	2	2	0	0
0	0	#NULL!	3	1	0	0
0	0	#NULL!	1	1	0	0
0	0	#NULL!	1	2	0	0
0	0	#NULL!	1	1	0	0
0	0	#NULL!	1	1	1	0
0	0	#NULL!	1	2	1	1
0	0	#NULL!	1	1	0	0
0	0	#NULL!	2	1	1	1
0	0	#NULL!	1	1	2	2
0	0	#NULL!	1	2	1	2
0	0	#NULL!	4	3	1	3
0	0	#NULL!	3	3	2	0
0	0	#NULL!	3	3	0	0
0	0	#NULL!	3	2	0	1
0	0	#NULL!	4	1	0	0
0	0	#NULL!	1	1	1	1
0	0	#NULL!	4	99	0	0
0	0	#NULL!	3	3	0	1
0	0	#NULL!	1	1	0	0
0	0	#NULL!	2	2	0	0
0	0	#NULL!	1	1	0	0
0	0	#NULL!	3	3	0	5
0	0	#NULL!	3	2	0	0
0	0	#NULL!	4	3	0	0
0	0	#NULL!	3	2	0	0
0	0	#NULL!	1	1	0	0
0	0	#NULL!	1	1	0	0
0	0	#NULL!	2	1	0	0
0	0	#NULL!	3	3	0	0
0	0	#NULL!	2	3	3	4
0	0	#NULL!	4	1	0	0
0	0	#NULL!	1	1	0	0
0	0	#NULL!	1	1	0	0
0	0	#NULL!	3	1	0	0
0	0	#NULL!	3	2	0	0
0	0	#NULL!	3	3	0	0
0	0	#NULL!	1	1	0	0

0	0	#NULL!	4	2	0	0
0	0	#NULL!	3	3	0	0
0	0	#NULL!	4	3	0	0
0	0	#NULL!	3	2	0	0
0	0	#NULL!	1	1	0	0
0	0	#NULL!	3	2	0	1
0	0	#NULL!	1	1	0	0
0	0	#NULL!	1	1	2	1
0	0	#NULL!	4	3	99	99
0	0	#NULL!	3	3	0	0
0	0	#NULL!	1	1	5	5
0	0	#NULL!	3	3	2	5
0	0	#NULL!	4	2	0	0
0	0	#NULL!	2	1	2	3
0	0	#NULL!	2	1	0	1
0	0	#NULL!	1	1	0	0
0	0	#NULL!	3	3	0	1
1	0	#NULL!	2	2	0	0
0	0	#NULL!	1	1	1	1
0	0	#NULL!	3	1	0	0
0	0	#NULL!	3	2	0	1
0	0	#NULL!	2	2	0	0
0	0	#NULL!	1	1	0	0
0	0	#NULL!	2	1	0	0
0	0	#NULL!	3	3	0	0
0	0	#NULL!	1	1	0	0
0	0	#NULL!	3	3	0	1
0	0	#NULL!	1	1	0	0
0	0	#NULL!	1	2	0	0
0	0	#NULL!	1	1	0	0
0	0	#NULL!	4	2	0	0
0	0	#NULL!	3	2	0	0
0	0	#NULL!	3	3	99	99
0	0	#NULL!	1	1	0	0
0	0	#NULL!	3	2	0	0
0	0	#NULL!	1	1	0	0
0	0	#NULL!	3	3	0	0
0	0	#NULL!	1	1	99	0
0	0	#NULL!	1	1	0	0
0	0	#NULL!	2	1	0	0
0	0	#NULL!	1	1	0	0
0	0	#NULL!	2	2	0	0
0	0	#NULL!	1	1	0	0
0	0	#NULL!	4	3	0	0
0	0	#NULL!	4	4	0	0
0	0	#NULL!	1	2	1	0
0	0	#NULL!	5	2	0	0
0	0	#NULL!	4	3	0	0

0	0	#NULL!	1	1	0	0
0	0	#NULL!	3	2	0	0
0	0	#NULL!	1	1	0	0
0	0	#NULL!	3	1	0	0
0	0	#NULL!	4	1	0	0
0	0	#NULL!	1	1	0	0
0	0	#NULL!	1	1	0	0
0	0	#NULL!	1	1	0	0
0	0	#NULL!	3	3	0	0
1	0	#NULL!	3	3	0	0
0	0	#NULL!	3	2	0	0
0	0	#NULL!	1	1	0	1
0	0	#NULL!	2	2	0	0
0	0	#NULL!	1	1	0	0
0	0	#NULL!	3	2	0	0
1	0	#NULL!	1	99	0	0
0	0	#NULL!	1	1	0	0
0	0	#NULL!	3	2	99	99
0	0	#NULL!	1	1	0	0
0	0	#NULL!	3	3	0	0
0	0	#NULL!	1	1	0	0
0	0	#NULL!	3	2	0	0
0	0	#NULL!	1	1	0	0
0	0	#NULL!	1	1	0	0
0	0	#NULL!	4	2	0	0
0	0	#NULL!	1	1	1	1
0	0	#NULL!	3	2	0	0
0	0	#NULL!	1	1	1	2
0	0	#NULL!	2	2	0	0
0	0	#NULL!	1	1	1	5
0	0	#NULL!	3	2	0	0
0	0	#NULL!	3	2	0	0
0	0	#NULL!	2	1	0	0
0	0	#NULL!	3	2	0	0
0	0	#NULL!	1	3	0	0
0	0	#NULL!	3	3	0	0
0	0	#NULL!	3	1	0	0
0	0	#NULL!	2	2	0	0
0	0	#NULL!	1	1	0	0
0	0	#NULL!	3	2	0	0
0	0	#NULL!	3	3	0	0
0	0	#NULL!	3	1	0	0
0	0	#NULL!	1	1	0	0
0	0	#NULL!	3	3	0	1
0	0	#NULL!	3	2	1	0
0	0	#NULL!	3	3	1	1
0	0	#NULL!	3	3	0	1
0	0	#NULL!	3	2	1	3

0	0	#NULL!	4	3	0	0
0	0	#NULL!	3	3	0	0
0	0	#NULL!	1	1	0	0
0	0	#NULL!	4	3	0	0
0	0	#NULL!	1	1	0	0
0	0	#NULL!	4	3	0	1
0	0	#NULL!	3	2	0	0
0	0	#NULL!	1	1	0	0
0	0	#NULL!	1	2	0	0
0	0	#NULL!	3	3	1	4
0	0	#NULL!	1	1	0	0
0	0	#NULL!	1	1	5	4
0	0	#NULL!	3	2	0	99
0	0	#NULL!	3	3	0	0
0	0	#NULL!	1	1	0	0
0	0	#NULL!	1	1	0	0
0	0	#NULL!	3	3	0	0
0	0	#NULL!	2	3	0	0
0	0	#NULL!	2	2	1	1
0	0	#NULL!	4	3	0	1
0	0	#NULL!	2	1	0	0
0	0	#NULL!	1	1	1	1
0	0	#NULL!	2	1	0	2
0	0	#NULL!	2	2	0	3
0	0	#NULL!	2	2	0	1
0	0	#NULL!	1	1	1	1
0	0	#NULL!	3	2	4	4
0	0	#NULL!	99	1	0	0
0	0	#NULL!	1	1	0	0
0	0	#NULL!	99	1	0	0
1	0	#NULL!	3	2	2	2
0	0	#NULL!	3	2	0	0
0	0	#NULL!	2	2	1	1
0	0	#NULL!	4	3	0	0
0	0	#NULL!	3	3	0	1
0	0	#NULL!	3	3	0	0
0	0	#NULL!	3	4	0	3
0	0	#NULL!	3	2	0	2
0	0	#NULL!	1	1	0	0
0	0	#NULL!	1	1	0	0
0	0	#NULL!	2	1	0	0
0	0	#NULL!	3	2	0	99
1	0	#NULL!	3	3	0	0
0	0	#NULL!	3	3	0	0
0	0	#NULL!	1	2	0	0
0	0	#NULL!	3	3	0	0
0	0	#NULL!	4	2	0	0
0	0	#NULL!	2	2	0	0

0	0	#NULL!	3	2	0	0
0	0	#NULL!	2	1	0	3
0	0	#NULL!	1	1	0	0
0	0	#NULL!	1	1	0	0
0	0	#NULL!	1	1	0	0
0	0	#NULL!	99	99	0	0
0	0	#NULL!	3	1	0	0
0	0	#NULL!	4	1	0	0
0	0	#NULL!	1	1	0	0
0	0	#NULL!	4	3	0	0
0	0	#NULL!	1	1	0	0
0	0	#NULL!	1	1	0	0
0	0	#NULL!	2	2	0	0
0	0	#NULL!	1	1	0	0
0	0	#NULL!	3	2	0	0
0	0	#NULL!	3	3	1	1
0	0	#NULL!	4	2	0	1
0	0	#NULL!	3	3	0	0
0	0	#NULL!	3	99	1	4
0	0	#NULL!	99	99	0	0
0	0	#NULL!	3	3	0	2
0	0	#NULL!	2	1	0	0
0	0	#NULL!	1	1	1	4
0	0	#NULL!	3	3	0	1
0	0	#NULL!	5	3	0	1
0	0	#NULL!	4	2	0	1
0	0	#NULL!	3	3	0	0
0	0	#NULL!	1	1	0	0
0	0	#NULL!	3	1	0	0
1	0	#NULL!	2	1	0	0
0	0	#NULL!	1	1	0	0
0	0	#NULL!	3	3	0	1
0	0	#NULL!	2	1	0	0
0	0	#NULL!	3	2	0	0
0	0	#NULL!	1	1	1	1
0	0	#NULL!	1	1	0	0
0	0	#NULL!	3	3	1	2
0	0	#NULL!	4	3	0	0
0	0	#NULL!	2	2	0	0
0	0	#NULL!	1	1	0	0
0	0	#NULL!	5	4	0	0
0	0	#NULL!	3	2	0	0
0	0	#NULL!	1	1	0	0
0	0	#NULL!	3	3	0	1
0	0	#NULL!	3	2	1	4
0	0	#NULL!	3	3	0	0
0	0	#NULL!	4	3	2	4
0	0	#NULL!	4	4	0	2

0	0	#NULL!	1	1	0	0
1	0	#NULL!	4	3	1	3
0	0	#NULL!	4	3	0	0
0	0	#NULL!	2	1	0	0
0	0	#NULL!	1	1	0	0
0	0	#NULL!	4	3	0	1
0	0	#NULL!	3	2	0	0
1	0	#NULL!	2	2	0	0
0	0	#NULL!	1	1	0	0
0	0	#NULL!	3	2	0	0
0	0	#NULL!	1	1	0	1
0	0	#NULL!	4	1	0	0
0	0	#NULL!	1	1	0	1
0	0	#NULL!	1	1	0	0
0	0	#NULL!	1	1	1	2
0	0	#NULL!	1	1	0	0
0	0	#NULL!	3	3	0	3
0	0	#NULL!	3	3	0	1
0	0	#NULL!	1	1	1	1
0	0	#NULL!	2	1	0	0
0	0	#NULL!	3	3	0	0
0	0	#NULL!	3	3	0	0
0	0	#NULL!	4	2	0	0
0	0	#NULL!	5	5	0	0
0	0	#NULL!	3	3	0	1
0	0	#NULL!	1	1	0	0
0	0	#NULL!	1	1	0	0
0	0	#NULL!	1	1	0	0
0	0	#NULL!	4	3	0	0
0	0	#NULL!	1	1	0	0
0	0	#NULL!	2	1	1	3
0	0	#NULL!	3	1	0	0
0	0	#NULL!	1	1	0	0
0	0	#NULL!	4	2	0	0
0	0	#NULL!	1	1	0	0
0	0	#NULL!	1	1	0	0
0	1	#NULL!	4	2	0	0
0	0	#NULL!	3	2	0	0
0	0	#NULL!	1	1	0	0
0	0	#NULL!	2	2	0	0
0	0	#NULL!	4	3	0	0
0	0	#NULL!	2	2	0	0
0	0	#NULL!	1	1	2	5
0	0	#NULL!	3	3	0	0
0	0	#NULL!	3	3	0	0
1	0	#NULL!	2	1	2	4
0	0	#NULL!	3	2	0	1
0	0	#NULL!	3	3	0	0

0	0	#NULL!	1	1	0	0
0	0	#NULL!	3	1	0	0
0	0	#NULL!	3	2	0	0
0	0	#NULL!	1	1	0	0
0	0	#NULL!	99	99	5	5
0	0	#NULL!	3	2	1	1
0	0	#NULL!	1	1	0	0
0	0	#NULL!	1	1	0	0
0	0	#NULL!	3	2	0	0
0	0	#NULL!	1	1	0	0
0	0	#NULL!	3	2	0	1
0	0	#NULL!	1	1	0	0
0	0	#NULL!	4	4	0	0
0	0	#NULL!	3	1	0	0
0	0	#NULL!	3	3	0	0
0	0	#NULL!	4	5	1	3
0	0	#NULL!	3	3	0	0
0	0	#NULL!	3	3	0	0
0	0	#NULL!	4	2	0	2
0	0	#NULL!	2	1	0	0
0	0	#NULL!	1	1	0	0
0	0	#NULL!	3	3	0	0
0	0	#NULL!	4	3	0	0
0	0	#NULL!	1	1	0	0
0	0	#NULL!	1	2	1	0
0	0	#NULL!	1	1	0	0
0	0	#NULL!	3	3	1	1
0	0	#NULL!	2	2	0	0
0	0	#NULL!	1	1	0	0
0	0	#NULL!	3	3	0	0
0	0	#NULL!	3	1	0	0
0	0	#NULL!	3	2	5	1
0	0	#NULL!	4	3	0	0
0	0	#NULL!	3	3	0	0
0	0	#NULL!	3	3	0	0
0	0	#NULL!	3	1	0	0
0	0	#NULL!	1	2	1	2
0	0	#NULL!	1	1	0	0
1	0	#NULL!	3	2	0	0
0	0	#NULL!	5	5	0	0
0	0	#NULL!	1	1	0	0
0	0	#NULL!	5	1	0	0
0	0	#NULL!	1	2	0	0
0	0	#NULL!	3	2	0	0
0	0	#NULL!	2	2	0	0
0	0	#NULL!	1	1	0	0
0	0	#NULL!	3	2	0	0
0	0	#NULL!	3	3	0	0

0	0	#NULL!	1	1	0	0
0	0	#NULL!	3	3	0	0
0	0	#NULL!	1	2	0	0
0	0	#NULL!	1	1	0	0
0	0	#NULL!	3	1	0	1
0	0	#NULL!	3	3	0	0
0	0	#NULL!	3	3	0	0
0	0	#NULL!	1	1	0	0
0	0	#NULL!	3	3	0	2
0	0	#NULL!	3	3	0	0
0	0	#NULL!	1	1	0	0
0	0	#NULL!	1	2	0	0
0	0	#NULL!	1	1	0	1
0	0	#NULL!	1	1	2	4
0	0	#NULL!	1	2	0	0
0	0	#NULL!	1	2	0	0
0	0	#NULL!	3	3	0	0
0	0	#NULL!	1	1	0	0
0	0	#NULL!	3	2	0	0
0	0	#NULL!	3	2	0	0
0	0	#NULL!	1	1	0	1
0	0	#NULL!	1	2	0	0
0	0	#NULL!	3	1	0	0
0	0	#NULL!	1	1	1	2
0	0	#NULL!	4	3	2	4
0	0	#NULL!	3	2	0	0
0	0	#NULL!	1	1	0	3
0	0	#NULL!	3	2	0	0
0	0	#NULL!	2	1	0	1
0	0	#NULL!	2	1	0	0
0	0	#NULL!	3	2	0	0
0	0	#NULL!	1	1	0	0
0	0	#NULL!	3	2	1	1
0	0	#NULL!	1	1	0	0
0	0	#NULL!	4	3	0	0
0	0	#NULL!	5	1	0	2
0	0	#NULL!	3	3	0	1
0	0	#NULL!	1	1	0	2
0	0	#NULL!	4	2	0	0
0	0	#NULL!	3	2	1	3
0	0	#NULL!	1	1	0	0
0	0	#NULL!	3	3	0	1
0	0	#NULL!	3	3	0	0
0	0	#NULL!	2	3	0	0
0	0	#NULL!	3	3	0	0
0	0	#NULL!	3	3	0	0
0	0	#NULL!	4	1	0	0
0	0	#NULL!	1	1	0	0

0	0	#NULL!	2	1	0	1
0	0	#NULL!	3	2	0	1
0	0	#NULL!	3	3	0	99
0	0	#NULL!	2	1	0	0
0	0	#NULL!	1	1	0	0
0	0	#NULL!	2	2	0	0
0	0	#NULL!	5	1	0	0
0	0	#NULL!	1	1	0	0
0	0	#NULL!	3	3	0	0
0	0	#NULL!	4	3	3	4
0	0	#NULL!	4	1	0	0
0	0	#NULL!	3	2	0	3
0	0	#NULL!	4	2	0	0
1	0	#NULL!	2	99	0	1
0	0	#NULL!	1	1	0	0
0	0	#NULL!	4	3	0	1
0	0	#NULL!	1	1	0	0
0	0	#NULL!	1	1	0	0
0	0	#NULL!	1	2	5	5
0	0	#NULL!	1	1	0	0
0	0	#NULL!	3	3	1	1
1	0	#NULL!	3	2	0	0
0	0	#NULL!	1	1	0	0
0	0	#NULL!	1	1	0	0
0	0	#NULL!	1	1	0	0
0	0	#NULL!	2	1	2	4
0	0	#NULL!	2	2	0	0
0	0	#NULL!	3	3	0	0
0	0	#NULL!	1	1	0	0
0	0	#NULL!	4	99	2	3
0	0	#NULL!	3	2	0	0
0	0	#NULL!	3	3	0	0
1	0	#NULL!	2	1	0	0
0	0	#NULL!	1	1	0	0
0	0	#NULL!	3	2	0	0
0	0	#NULL!	1	1	0	0
0	0	#NULL!	1	1	0	0
0	0	#NULL!	4	3	0	0
0	0	#NULL!	1	1	0	0
0	0	#NULL!	1	1	0	0
0	0	#NULL!	1	1	0	0
0	0	#NULL!	1	1	0	0
0	0	#NULL!	1	1	0	1
0	0	#NULL!	3	2	0	0
0	0	#NULL!	1	1	0	0
0	0	#NULL!	1	1	4	2
0	0	#NULL!	1	4	1	1
0	0	#NULL!	4	3	0	0

0	0	#NULL!	2	1	1	1
0	0	#NULL!	5	1	0	0
0	0	#NULL!	3	2	0	0
0	0	#NULL!	4	3	0	99
0	0	#NULL!	3	2	0	0
0	0	#NULL!	3	2	0	0
0	0	#NULL!	3	3	0	0
0	0	#NULL!	2	1	0	0
0	0	#NULL!	1	1	0	0
0	0	#NULL!	1	2	0	1
0	0	#NULL!	3	3	0	0
0	0	#NULL!	99	3	0	0
0	0	#NULL!	3	3	0	5
0	0	#NULL!	3	2	0	1
0	0	#NULL!	3	3	0	0
0	0	#NULL!	3	3	0	0
0	0	#NULL!	1	1	0	0
0	0	#NULL!	1	1	0	0
0	0	#NULL!	4	2	0	0
0	0	#NULL!	5	4	0	0
0	0	#NULL!	1	1	1	1
0	0	#NULL!	1	1	0	0
0	0	#NULL!	4	2	0	3
0	0	#NULL!	3	2	0	0
0	0	#NULL!	2	3	0	0
0	0	#NULL!	3	2	0	0
0	0	#NULL!	3	3	0	1
0	0	#NULL!	1	1	0	0
0	0	#NULL!	4	3	0	0
0	0	#NULL!	1	1	0	0
0	0	#NULL!	3	3	0	1
1	0	#NULL!	3	2	0	0
0	0	#NULL!	3	2	0	0
0	0	#NULL!	1	1	0	0
0	0	#NULL!	3	2	0	1
0	0	#NULL!	3	3	0	0
0	0	#NULL!	2	1	0	1
0	0	#NULL!	3	3	0	0
0	0	#NULL!	1	1	0	3
0	0	#NULL!	1	1	0	2
0	0	#NULL!	2	1	0	0
0	0	#NULL!	1	1	0	0
0	0	#NULL!	4	3	99	99
0	0	#NULL!	3	2	0	3
0	0	#NULL!	2	1	0	0
0	0	#NULL!	3	1	0	1
0	0	#NULL!	3	3	0	1
0	0	#NULL!	3	3	1	5

0	0	#NULL!	3	3	0	1
0	0	#NULL!	4	3	0	2
0	0	#NULL!	3	3	0	0
0	0	#NULL!	1	1	0	0
0	0	#NULL!	1	1	2	5
0	0	#NULL!	3	2	2	5
0	0	#NULL!	4	2	0	1
0	0	#NULL!	1	1	0	0
0	0	#NULL!	3	2	0	0
0	0	#NULL!	1	1	0	0
1	0	#NULL!	4	5	5	0
0	0	#NULL!	5	3	0	2
0	0	#NULL!	3	2	0	0
0	0	#NULL!	4	4	0	1
0	0	#NULL!	1	1	0	2
0	0	#NULL!	3	3	0	1
0	0	#NULL!	1	1	1	0
0	0	#NULL!	2	1	0	0
0	0	#NULL!	2	1	2	3
0	0	#NULL!	1	1	0	5
0	0	#NULL!	1	1	1	0
0	0	#NULL!	1	1	1	1
0	0	#NULL!	1	2	3	99
0	0	#NULL!	4	3	0	0
0	0	#NULL!	1	1	1	5
0	0	#NULL!	4	2	0	0
0	0	#NULL!	3	2	0	0
0	0	#NULL!	3	2	0	1
0	0	#NULL!	2	1	0	1
0	0	#NULL!	3	3	0	0
0	0	#NULL!	3	2	0	0
0	1	#NULL!	3	3	0	0
0	0	#NULL!	1	1	0	0
0	0	#NULL!	1	1	4	4
0	0	#NULL!	1	1	5	5
0	0	#NULL!	4	3	0	1
0	0	#NULL!	1	2	0	0
0	0	#NULL!	2	2	0	0
0	0	#NULL!	2	2	5	3
0	0	#NULL!	1	99	1	1
0	0	#NULL!	3	2	0	0
0	0	#NULL!	4	3	0	0
0	0	#NULL!	1	1	0	1
0	0	#NULL!	1	1	0	0
0	0	#NULL!	3	2	0	0
0	0	#NULL!	3	2	1	1
0	0	#NULL!	1	1	1	1
0	0	#NULL!	3	3	0	1

0	0	#NULL!	1	1	0	0
0	0	#NULL!	1	1	2	5
0	0	#NULL!	1	1	0	0
0	1	#NULL!	99	99	0	0
1	0	#NULL!	3	1	1	0
0	0	#NULL!	1	2	3	3
0	0	#NULL!	3	3	0	0
0	0	#NULL!	3	3	0	99
0	0	#NULL!	2	1	2	2
0	0	#NULL!	3	4	0	2
0	0	#NULL!	3	2	1	2
0	0	#NULL!	1	1	1	1
0	0	#NULL!	3	2	0	0
0	0	#NULL!	3	1	0	5
0	0	#NULL!	1	1	0	0
0	0	#NULL!	1	1	0	0
0	0	#NULL!	1	99	1	1
0	0	#NULL!	1	2	0	0
0	0	#NULL!	3	3	0	0
0	0	#NULL!	1	2	0	0
0	0	#NULL!	1	1	1	1
0	0	#NULL!	1	1	0	0
0	0	#NULL!	99	99	0	0
0	0	#NULL!	3	2	1	1
0	0	#NULL!	1	1	0	0
1	0	#NULL!	2	2	1	2
0	0	#NULL!	3	3	0	0
0	0	#NULL!	3	2	0	0
0	0	#NULL!	1	1	0	0
0	0	#NULL!	1	1	0	0
0	0	#NULL!	3	2	0	0
0	0	#NULL!	3	2	0	0
0	0	#NULL!	1	1	1	0
0	0	#NULL!	1	3	0	0
0	0	#NULL!	1	2	2	5
0	0	#NULL!	1	1	0	1
0	0	#NULL!	1	1	0	0
0	0	#NULL!	3	3	0	0
0	0	#NULL!	1	99	0	0
0	0	#NULL!	3	2	0	0
1	0	#NULL!	99	99	0	1
0	0	#NULL!	1	1	0	0
0	0	#NULL!	3	2	0	0
0	0	#NULL!	3	3	3	4
0	0	#NULL!	3	3	0	0
0	0	#NULL!	1	1	0	0
0	1	#NULL!	4	99	3	4
0	0	#NULL!	3	2	0	0

0	0	#NULL!	1	1	0	0
0	0	#NULL!	1	1	2	5
0	0	#NULL!	4	2	0	0
0	0	#NULL!	1	1	0	0
0	0	#NULL!	1	1	0	0
0	0	#NULL!	3	3	0	0
0	0	#NULL!	3	3	0	0
0	0	#NULL!	2	1	0	5
0	0	#NULL!	99	1	3	5
0	0	#NULL!	4	2	0	2
0	0	#NULL!	1	1	0	0
0	0	#NULL!	1	1	0	0
0	0	#NULL!	4	2	0	0
0	0	#NULL!	1	1	0	2
0	0	#NULL!	1	1	0	0
0	0	#NULL!	1	1	0	0
0	0	#NULL!	1	1	0	0
0	0	#NULL!	2	1	0	0
0	0	#NULL!	2	3	0	0
0	0	#NULL!	2	2	0	2
0	0	#NULL!	2	2	0	1
0	0	#NULL!	1	1	3	4
0	1	#NULL!	1	1	0	0
0	0	#NULL!	3	2	1	1
0	0	#NULL!	3	2	0	0
0	0	#NULL!	1	1	0	0
0	0	#NULL!	3	2	0	0
0	0	#NULL!	2	2	0	0
0	0	#NULL!	3	3	0	0
0	0	#NULL!	3	3	0	0
0	0	#NULL!	1	1	0	0
0	0	#NULL!	1	1	0	0
0	0	#NULL!	1	1	0	0
0	0	#NULL!	1	1	0	0
0	0	#NULL!	2	1	0	0
0	0	#NULL!	3	2	1	2
0	0	#NULL!	1	1	0	0
1	0	#NULL!	4	1	3	2
0	0	#NULL!	4	2	0	0
0	0	#NULL!	1	3	0	1
0	0	#NULL!	1	1	2	4
0	0	#NULL!	1	1	0	0
0	0	#NULL!	4	3	0	1
0	0	#NULL!	1	1	0	0
0	0	#NULL!	5	5	0	0
0	0	#NULL!	5	3	0	0
0	0	#NULL!	1	1	0	0
0	0	#NULL!	1	1	0	0

0	0	#NULL!	3	1	0	0
0	0	#NULL!	4	3	0	0
0	0	#NULL!	3	3	1	2
0	0	#NULL!	3	1	1	1
0	0	#NULL!	1	1	0	1
0	0	#NULL!	1	1	0	0
0	1	#NULL!	1	1	5	5
0	0	#NULL!	5	2	0	0
0	0	#NULL!	1	1	0	1
0	0	#NULL!	2	2	0	0
0	0	#NULL!	2	1	0	0
0	0	#NULL!	1	1	0	0
0	0	#NULL!	4	3	0	1
0	0	#NULL!	3	3	0	0
0	0	#NULL!	4	2	0	0
0	0	#NULL!	3	3	0	1
0	0	#NULL!	1	1	0	0
0	0	#NULL!	2	1	0	1
0	0	#NULL!	1	1	2	2
0	0	#NULL!	1	1	0	0
0	0	#NULL!	1	1	0	0
0	0	#NULL!	3	2	0	0
0	0	#NULL!	1	1	0	0
0	0	#NULL!	1	1	0	0
0	0	#NULL!	1	1	0	0
0	0	#NULL!	1	2	0	0
0	0	#NULL!	1	1	0	0
0	0	#NULL!	5	2	0	3
0	0	#NULL!	3	1	0	0
0	0	#NULL!	3	2	0	0
0	0	#NULL!	3	2	0	0
0	0	#NULL!	3	1	0	0
0	0	#NULL!	2	1	0	0
0	0	#NULL!	3	2	0	0
0	0	#NULL!	3	3	0	2
0	0	#NULL!	1	1	0	0
0	0	#NULL!	99	99	0	0
0	0	#NULL!	3	2	0	1
0	0	#NULL!	4	3	0	0
0	0	#NULL!	3	2	0	0
0	0	#NULL!	1	1	0	0
0	0	#NULL!	3	3	0	0
0	0	#NULL!	5	3	0	0
0	0	#NULL!	1	1	0	0
0	0	#NULL!	1	1	0	0
0	0	#NULL!	2	1	0	0
0	0	#NULL!	4	2	0	0
0	0	#NULL!	1	1	1	0

0	0	#NULL!	3	2	0	0
0	0	#NULL!	3	2	0	0
0	0	#NULL!	4	2	0	0
0	0	#NULL!	2	1	0	0
0	0	#NULL!	3	3	0	0
0	0	#NULL!	1	1	0	0
0	0	#NULL!	1	1	0	0
0	0	#NULL!	1	1	0	0
0	0	#NULL!	1	99	0	0
0	0	#NULL!	3	2	0	0
0	0	#NULL!	1	1	1	0
0	0	#NULL!	2	1	0	0
0	0	#NULL!	1	1	0	0
1	0	#NULL!	3	1	0	0
0	0	#NULL!	1	1	0	0
0	0	#NULL!	3	3	0	0
0	0	#NULL!	1	1	0	0
0	0	#NULL!	5	2	0	0
0	0	#NULL!	5	3	1	0
0	0	#NULL!	1	1	0	0
0	0	#NULL!	3	1	0	0
0	0	#NULL!	4	3	0	0
0	0	#NULL!	3	2	0	0
0	0	#NULL!	3	1	0	0
0	0	#NULL!	5	5	0	0
0	0	#NULL!	1	1	0	0
0	0	#NULL!	1	1	0	0
0	0	#NULL!	1	1	0	0
0	0	#NULL!	1	1	0	0
0	0	#NULL!	1	1	0	0
0	1	#NULL!	1	1	0	0
0	0	#NULL!	2	3	99	99
0	0	#NULL!	4	2	0	0
0	0	#NULL!	1	1	0	0
0	0	#NULL!	1	1	0	0
0	0	#NULL!	3	2	0	0
0	0	#NULL!	5	3	0	0
0	0	#NULL!	1	1	0	0
0	0	#NULL!	2	3	0	0
0	0	#NULL!	1	1	0	0
0	0	#NULL!	3	2	0	0
0	0	#NULL!	3	2	0	0
0	0	#NULL!	4	2	0	0
0	0	#NULL!	2	3	0	1
0	0	#NULL!	3	2	0	0
0	0	#NULL!	1	1	0	0
0	0	#NULL!	5	3	0	0
0	0	#NULL!	1	1	0	0

0	0	#NULL!	4	2	0	0
0	0	#NULL!	2	1	0	0
0	0	#NULL!	1	1	0	0
0	0	#NULL!	3	2	0	0
0	0	#NULL!	3	2	0	0
0	0	#NULL!	3	3	0	0
0	0	#NULL!	3	2	0	0
0	0	#NULL!	1	1	0	0
0	0	#NULL!	3	3	1	2
0	0	#NULL!	1	1	0	0
1	0	#NULL!	5	1	0	0
0	0	#NULL!	1	1	0	0
0	0	#NULL!	3	3	0	0
0	0	#NULL!	4	4	0	0
0	0	#NULL!	3	3	0	0
0	0	#NULL!	3	2	0	0
0	0	#NULL!	3	4	0	0
0	0	#NULL!	3	2	0	1
0	0	#NULL!	1	2	0	0
0	0	#NULL!	3	2	0	1
0	0	#NULL!	3	3	0	3
0	0	#NULL!	1	1	0	0
0	0	#NULL!	5	3	0	0
0	0	#NULL!	3	3	0	0
0	0	#NULL!	99	1	0	0
0	0	#NULL!	1	1	0	0
0	0	#NULL!	1	1	0	0
0	0	#NULL!	1	1	0	0
0	0	#NULL!	2	1	0	0
0	0	#NULL!	1	1	0	0
0	0	#NULL!	3	99	0	0
0	0	#NULL!	3	3	0	1
0	0	#NULL!	1	1	0	0
0	0	#NULL!	4	2	0	0
0	0	#NULL!	2	1	0	0
1	0	#NULL!	1	1	0	0
0	0	#NULL!	4	2	0	1
0	0	#NULL!	1	1	0	0
0	0	#NULL!	3	2	0	0
0	0	#NULL!	1	1	0	0
0	0	#NULL!	1	1	0	0
0	0	#NULL!	4	2	0	0
0	0	#NULL!	1	1	1	0
0	0	#NULL!	3	2	1	0
0	0	#NULL!	4	99	0	0
0	0	#NULL!	1	1	0	1
0	0	#NULL!	1	1	0	0
0	0	#NULL!	1	1	0	0

0	0	#NULL!	1	1	0	0
0	0	#NULL!	1	1	0	0
0	0	#NULL!	3	3	0	0
0	0	#NULL!	1	1	0	0
0	0	#NULL!	99	99	0	1
0	0	#NULL!	1	1	0	1
0	0	#NULL!	1	1	0	0
0	0	#NULL!	3	3	0	0
0	0	#NULL!	1	1	0	0
0	0	#NULL!	1	1	0	0
0	0	#NULL!	4	2	0	0
0	0	#NULL!	2	3	0	0
0	0	#NULL!	2	2	0	0
0	0	#NULL!	3	1	0	0
1	0	#NULL!	1	1	0	0
0	0	#NULL!	2	2	0	0
0	0	#NULL!	3	1	0	5
0	0	#NULL!	3	1	0	0
0	0	#NULL!	2	2	0	0
0	0	#NULL!	3	2	0	0
1	0	#NULL!	4	1	0	0
0	0	#NULL!	2	2	0	0
0	0	#NULL!	1	1	0	0
0	0	#NULL!	4	3	0	0
0	0	#NULL!	3	1	0	0
0	0	#NULL!	3	1	0	0
0	0	#NULL!	3	3	0	0
0	0	#NULL!	2	1	0	0
0	0	#NULL!	1	1	0	0
0	0	#NULL!	1	3	0	0
0	0	#NULL!	3	3	0	0
0	0	#NULL!	2	2	0	0
0	0	#NULL!	2	1	0	99
0	0	#NULL!	1	1	0	1
0	0	#NULL!	3	1	0	0
0	0	#NULL!	3	3	99	0
0	0	#NULL!	2	2	0	0
0	0	#NULL!	3	2	0	0
0	0	#NULL!	1	1	0	0
0	0	#NULL!	2	2	0	0
0	0	#NULL!	1	1	0	0
0	0	#NULL!	3	2	0	0
0	0	#NULL!	3	1	0	0
0	0	#NULL!	4	4	0	0
0	0	#NULL!	1	1	0	0
0	0	#NULL!	1	1	0	0
0	0	#NULL!	5	4	0	1
0	1	#NULL!	99	99	0	1

0	0	#NULL!	5	3	1	5
0	0	#NULL!	3	3	0	0
0	0	#NULL!	2	2	0	0
0	0	#NULL!	2	1	2	99
0	0	#NULL!	4	3	0	0
0	0	#NULL!	1	1	0	0
0	0	#NULL!	3	3	0	0
0	0	#NULL!	2	2	0	0
0	0	#NULL!	2	2	0	0
0	0	#NULL!	1	1	1	1
0	0	#NULL!	1	1	0	0
0	0	#NULL!	1	1	0	0
0	0	#NULL!	3	2	0	0
0	0	#NULL!	1	3	1	1
0	0	#NULL!	3	2	0	0
0	0	#NULL!	1	1	1	2
0	0	#NULL!	1	1	0	0
0	0	#NULL!	1	1	0	1
0	0	#NULL!	2	2	0	99
0	0	#NULL!	4	3	1	1
0	0	#NULL!	3	2	0	0
0	0	#NULL!	3	3	0	0
0	0	#NULL!	4	2	0	0
0	0	#NULL!	1	1	0	0
0	0	#NULL!	3	2	0	0
0	0	#NULL!	1	1	0	0
0	0	#NULL!	1	1	5	2
0	0	#NULL!	3	2	0	0
0	0	#NULL!	3	2	1	3
0	0	#NULL!	2	1	1	1
0	0	#NULL!	2	2	1	1
0	0	#NULL!	3	3	0	0
0	0	#NULL!	1	1	0	0
0	0	#NULL!	1	1	0	0
0	0	#NULL!	3	3	0	0
0	0	#NULL!	1	1	0	2
0	0	#NULL!	2	2	0	0
0	0	#NULL!	4	2	0	1
0	0	#NULL!	2	1	0	0
0	0	#NULL!	1	1	0	0
0	0	#NULL!	2	2	1	1
0	0	#NULL!	2	2	0	0
0	0	#NULL!	1	1	0	0
0	0	#NULL!	3	3	0	3
0	0	#NULL!	2	3	0	0
0	0	#NULL!	3	3	0	0
0	0	#NULL!	2	1	0	1
0	0	#NULL!	1	1	0	0

0	0	#NULL!	3	3	0	0
0	0	#NULL!	4	3	0	0
0	0	#NULL!	1	1	0	0
0	0	#NULL!	3	2	0	0
0	0	#NULL!	4	2	0	0
0	0	#NULL!	1	1	0	0
0	0	#NULL!	1	2	0	0
0	0	#NULL!	3	2	0	0
0	0	#NULL!	1	1	0	1
0	0	#NULL!	2	3	0	0
0	0	#NULL!	2	2	0	1
0	0	#NULL!	3	4	0	0
0	0	#NULL!	4	3	0	0
0	0	#NULL!	1	1	0	0
0	0	#NULL!	4	3	1	4
0	0	#NULL!	1	1	0	0
0	0	#NULL!	1	1	0	0
0	0	#NULL!	1	1	0	0
0	0	#NULL!	1	1	0	0
0	0	#NULL!	1	1	0	0
0	0	#NULL!	1	1	0	99
0	0	#NULL!	1	1	0	0
0	0	#NULL!	1	1	0	0
0	0	#NULL!	1	3	0	1
0	0	#NULL!	1	2	0	0
0	0	#NULL!	1	1	0	0
0	0	#NULL!	1	1	0	0
0	0	#NULL!	3	3	2	3
0	0	#NULL!	4	2	0	0
0	0	#NULL!	1	1	0	0
0	0	#NULL!	1	1	0	0
0	0	#NULL!	2	2	0	0
0	0	#NULL!	1	2	0	0
0	0	#NULL!	2	1	0	0
0	0	#NULL!	1	1	0	0
0	0	#NULL!	3	3	0	1
0	0	#NULL!	4	3	0	0
0	0	#NULL!	1	1	0	0
0	0	#NULL!	3	1	0	0
0	0	#NULL!	5	3	0	0
0	0	#NULL!	1	1	0	0
0	0	#NULL!	1	2	0	0
0	0	#NULL!	3	3	0	0
0	0	#NULL!	2	2	0	0
0	0	#NULL!	3	1	0	0
0	0	#NULL!	3	3	0	0
0	0	#NULL!	4	2	0	0
0	0	#NULL!	3	3	0	0

0	0	#NULL!	3	2	0	0
0	0	#NULL!	4	3	0	1
0	0	#NULL!	3	1	1	1
0	0	#NULL!	1	1	0	0
0	0	#NULL!	3	3	0	0
0	0	#NULL!	3	1	0	0
0	0	#NULL!	1	1	0	0
0	0	#NULL!	1	1	0	0
0	0	#NULL!	4	3	0	0
0	0	#NULL!	4	2	0	1
0	0	#NULL!	1	1	0	0
0	0	#NULL!	1	1	0	0
0	0	#NULL!	2	2	0	1
0	0	#NULL!	1	1	0	0
0	0	#NULL!	2	2	1	0
0	0	#NULL!	1	1	0	0
0	0	#NULL!	2	1	0	0
0	0	#NULL!	2	1	0	0
0	0	#NULL!	1	1	0	0
0	0	#NULL!	1	1	0	0
0	0	#NULL!	1	1	0	1
0	0	#NULL!	1	1	0	0
0	0	#NULL!	1	1	5	0
0	0	#NULL!	3	2	0	0
0	0	#NULL!	2	1	0	0
0	0	#NULL!	3	1	0	1
1	0	#NULL!	2	2	0	0
0	0	#NULL!	1	1	0	0
0	0	#NULL!	1	2	0	0
0	0	#NULL!	3	2	0	0
0	0	#NULL!	3	2	0	0
0	0	#NULL!	2	2	1	0
0	0	#NULL!	1	1	0	0
0	0	#NULL!	3	3	0	1
1	0	#NULL!	3	3	0	0
0	0	#NULL!	1	1	0	0
0	0	#NULL!	5	3	0	0
0	0	#NULL!	2	1	1	2
0	0	#NULL!	1	1	0	1
0	1	#NULL!	2	1	0	0
0	0	#NULL!	1	1	0	0
0	0	#NULL!	3	3	0	0
0	0	#NULL!	2	1	5	5
0	0	#NULL!	4	3	0	0
0	0	#NULL!	2	1	1	2
0	0	#NULL!	4	2	3	2
0	0	#NULL!	2	2	0	0
0	0	#NULL!	1	99	0	1

0	0	#NULL!	3	2	0	0
0	0	#NULL!	3	1	0	0
0	0	#NULL!	2	3	0	0
0	0	#NULL!	3	3	0	1
0	0	#NULL!	1	1	0	1
0	0	#NULL!	3	1	0	3
0	0	#NULL!	1	1	0	0
0	0	#NULL!	2	1	0	0
0	0	#NULL!	4	1	0	0
0	0	#NULL!	1	1	0	0
0	0	#NULL!	1	2	0	0
0	0	#NULL!	1	1	0	0
0	0	#NULL!	4	3	0	3
0	0	#NULL!	1	1	0	0
0	0	#NULL!	5	99	2	2
0	0	#NULL!	3	3	0	0
0	0	#NULL!	1	1	0	0
0	0	#NULL!	1	1	0	0
0	0	#NULL!	3	3	0	0
0	0	#NULL!	4	3	0	0
0	0	#NULL!	1	1	0	1
0	0	#NULL!	2	2	0	0
0	0	#NULL!	3	3	1	1
0	0	#NULL!	1	1	0	0
0	0	#NULL!	1	1	1	2
0	0	#NULL!	2	2	0	1
0	0	#NULL!	3	3	99	1
0	0	#NULL!	1	1	0	0
0	0	#NULL!	2	2	0	0
0	0	#NULL!	3	3	0	0
0	0	#NULL!	4	1	0	0
0	0	#NULL!	3	1	0	99
0	0	#NULL!	3	1	0	0
0	0	#NULL!	1	1	1	1
0	0	#NULL!	1	1	0	0
0	0	#NULL!	3	2	0	0
0	0	#NULL!	2	1	0	0
0	0	#NULL!	5	4	0	0
0	0	#NULL!	1	1	0	0
0	0	#NULL!	3	2	0	0
0	0	#NULL!	3	3	0	1
0	0	#NULL!	3	3	0	0
0	0	#NULL!	3	3	0	0
0	0	#NULL!	2	3	0	0
0	0	#NULL!	3	1	0	0
0	0	#NULL!	3	3	0	0
0	0	#NULL!	1	1	0	0
0	0	#NULL!	3	2	0	0

0	0	#NULL!	3	1	0	0
0	0	#NULL!	1	2	0	0
0	0	#NULL!	1	1	0	0
0	0	#NULL!	1	1	0	0
0	0	#NULL!	1	1	0	0
0	0	#NULL!	4	2	0	0
0	0	#NULL!	5	2	0	0
0	0	#NULL!	1	3	0	0
0	0	#NULL!	3	2	0	0
0	0	#NULL!	1	1	0	0
0	0	#NULL!	1	1	0	1
0	1	#NULL!	99	99	0	1
0	0	#NULL!	3	3	0	0
0	0	#NULL!	1	1	0	0
0	0	#NULL!	4	2	0	0
0	0	#NULL!	1	1	0	2
0	0	#NULL!	1	2	0	0
0	0	#NULL!	2	2	0	0
0	0	#NULL!	3	3	0	0
0	0	#NULL!	1	1	0	0
0	0	#NULL!	3	2	0	0
0	0	#NULL!	3	1	0	0
0	0	#NULL!	3	2	0	0
0	0	#NULL!	1	1	0	0
0	0	#NULL!	3	2	0	0
0	0	#NULL!	1	2	0	0
0	0	#NULL!	1	1	0	0
0	0	#NULL!	4	2	0	0
0	0	#NULL!	3	1	0	0
0	0	#NULL!	1	1	0	0
0	0	#NULL!	5	1	0	0
0	1	#NULL!	99	99	99	99
0	0	#NULL!	1	1	0	0
0	0	#NULL!	1	1	0	0
0	0	#NULL!	1	1	0	0
0	0	#NULL!	2	2	3	2
0	0	#NULL!	5	2	0	0
0	0	#NULL!	1	1	0	0
0	0	#NULL!	3	3	0	0
0	0	#NULL!	4	3	0	0
0	0	#NULL!	2	2	0	0
1	0	#NULL!	4	1	0	0
0	0	#NULL!	1	1	0	0
0	0	#NULL!	1	1	0	0
0	0	#NULL!	1	1	0	0
0	0	#NULL!	1	1	0	0
0	0	#NULL!	1	1	0	0
0	0	#NULL!	3	2	0	0

0	0	#NULL!	4	3	0	0
0	0	#NULL!	1	1	0	0
0	0	#NULL!	2	2	0	0
0	0	#NULL!	2	3	1	2
0	0	#NULL!	2	2	0	0
0	0	#NULL!	2	3	0	2
0	0	#NULL!	1	1	0	0
0	0	#NULL!	1	1	0	0
0	0	#NULL!	1	1	1	1
0	0	#NULL!	3	3	0	2
1	0	#NULL!	2	1	1	3
0	0	#NULL!	4	2	0	0
0	0	#NULL!	1	1	0	1
0	0	#NULL!	3	2	99	99
0	0	#NULL!	1	1	0	1
0	0	#NULL!	1	99	1	1
0	0	#NULL!	3	2	3	3
0	0	#NULL!	2	1	2	2
0	0	#NULL!	1	1	0	1
0	0	#NULL!	1	1	1	2
0	0	#NULL!	3	3	0	0
0	0	#NULL!	1	1	0	0
0	0	#NULL!	3	3	0	99
0	0	#NULL!	3	2	0	0
0	0	#NULL!	2	3	0	0
0	0	#NULL!	3	2	0	0
0	0	#NULL!	3	3	0	0
0	0	#NULL!	1	1	0	0
0	0	#NULL!	1	1	0	0
0	0	#NULL!	1	1	0	0
0	0	#NULL!	1	1	0	1
0	0	#NULL!	2	2	0	0
0	0	#NULL!	3	2	0	0
0	0	#NULL!	3	2	0	0
0	0	#NULL!	1	1	1	0
0	0	#NULL!	2	1	0	0
0	0	#NULL!	1	1	0	0
0	0	#NULL!	1	1	0	0
0	0	#NULL!	1	1	0	0
0	0	#NULL!	3	3	0	1
0	0	#NULL!	2	2	0	0
0	0	#NULL!	5	2	0	0
0	0	#NULL!	2	1	0	0
0	0	#NULL!	3	3	0	0
1	0	#NULL!	3	3	0	0
0	0	#NULL!	3	1	0	0
0	0	#NULL!	3	2	0	0
0	0	#NULL!	1	1	0	0

1	0	#NULL!	3	4	0	0
0	0	#NULL!	3	3	0	1
0	0	#NULL!	2	3	1	0
0	0	#NULL!	1	1	0	0
0	0	#NULL!	4	4	0	0
0	0	#NULL!	3	3	0	0
0	0	#NULL!	4	3	0	0
0	0	#NULL!	1	1	0	0
0	0	#NULL!	3	2	0	0
0	0	#NULL!	4	3	0	0
0	0	#NULL!	3	2	1	0
0	0	#NULL!	1	1	0	0
0	0	#NULL!	1	1	0	0
0	0	#NULL!	3	1	0	0
0	0	#NULL!	1	1	99	99
0	0	#NULL!	3	3	0	0
0	0	#NULL!	3	3	1	5
0	0	#NULL!	3	4	0	0
0	0	#NULL!	4	4	0	0
0	1	#NULL!	99	99	99	99
0	0	#NULL!	2	3	0	2
0	0	#NULL!	1	1	0	3
0	0	#NULL!	1	1	1	1
0	0	#NULL!	1	1	1	2
0	0	#NULL!	1	1	0	0
0	0	#NULL!	1	1	0	0
0	0	#NULL!	2	2	0	2
0	0	#NULL!	1	1	0	0
0	0	#NULL!	4	3	0	0
0	0	#NULL!	1	1	0	0
0	0	#NULL!	3	3	0	0
0	0	#NULL!	4	2	0	0
0	0	#NULL!	1	1	0	0
0	0	#NULL!	1	2	0	0
0	0	#NULL!	4	3	0	1
0	0	#NULL!	1	1	1	2
0	0	#NULL!	1	1	3	1
0	0	#NULL!	3	2	0	1
0	0	#NULL!	4	2	0	0
0	0	#NULL!	1	1	0	0
0	0	#NULL!	3	3	0	0
0	0	#NULL!	99	2	0	1
0	0	#NULL!	4	3	0	0
0	0	#NULL!	3	1	0	0
0	0	#NULL!	3	5	0	0
0	0	#NULL!	1	1	0	0
0	0	#NULL!	1	1	0	0
0	0	#NULL!	2	4	1	1

0	0	#NULL!	5	3	0	0
0	0	#NULL!	3	1	0	0
0	0	#NULL!	3	2	0	0
0	0	#NULL!	1	1	0	0
0	0	#NULL!	4	2	0	0
1	0	#NULL!	3	2	0	0
0	0	#NULL!	2	99	0	0
0	0	#NULL!	3	2	0	0
0	0	#NULL!	1	1	0	0
0	0	#NULL!	1	1	2	3
0	0	#NULL!	1	1	5	5
0	0	#NULL!	5	3	0	0
0	0	#NULL!	1	2	2	0
0	0	#NULL!	3	3	0	0
0	0	#NULL!	5	3	0	3
0	0	#NULL!	1	1	0	1
0	0	#NULL!	1	1	0	0
0	0	#NULL!	3	3	0	0
0	0	#NULL!	3	2	0	1
0	0	#NULL!	3	3	0	0
0	0	#NULL!	1	1	0	0
0	0	#NULL!	3	2	0	0
0	0	#NULL!	1	1	0	0
0	0	#NULL!	3	3	0	0
0	0	#NULL!	3	3	0	0
0	0	#NULL!	1	1	0	0
0	0	#NULL!	3	3	0	0
0	0	#NULL!	3	3	0	0
0	0	#NULL!	4	2	0	0
0	0	#NULL!	2	2	1	1
0	0	#NULL!	3	2	1	2
0	0	#NULL!	1	1	0	0
0	0	#NULL!	1	1	1	0
0	0	#NULL!	1	1	0	0
0	0	#NULL!	1	1	0	0
0	0	#NULL!	1	2	4	3
0	0	#NULL!	3	2	0	0
0	0	#NULL!	1	1	0	0
0	0	#NULL!	3	3	1	2
0	0	#NULL!	1	2	5	5
0	0	#NULL!	1	1	0	0
0	0	#NULL!	5	1	5	5
0	0	#NULL!	1	1	0	0
0	0	#NULL!	1	1	0	0
0	0	#NULL!	1	1	0	0
0	0	#NULL!	3	3	0	0
0	0	#NULL!	3	3	0	0
0	0	#NULL!	1	1	0	0

0	0	#NULL!	1	1	2	2
0	0	#NULL!	4	3	0	0
1	0	#NULL!	3	1	0	0
0	0	#NULL!	1	1	0	0
0	0	#NULL!	4	2	0	0
0	0	#NULL!	3	3	0	0
0	0	#NULL!	3	3	0	0
0	0	#NULL!	1	1	0	0
0	0	#NULL!	1	1	0	1
0	0	#NULL!	3	2	0	3
0	0	#NULL!	1	1	0	0
0	0	#NULL!	2	2	0	0
0	0	#NULL!	1	1	0	0
0	0	#NULL!	2	1	0	0
0	0	#NULL!	2	1	0	0
0	0	#NULL!	3	3	0	0
0	0	#NULL!	3	3	0	0
0	0	#NULL!	3	1	0	0
0	0	#NULL!	1	1	0	0
0	0	#NULL!	4	2	0	1
0	0	#NULL!	1	1	0	0
0	0	#NULL!	3	3	0	0
0	0	#NULL!	1	1	0	0
0	0	#NULL!	99	99	0	0
0	0	#NULL!	1	1	0	0
0	0	#NULL!	1	3	0	0
0	0	#NULL!	2	1	0	0
0	0	#NULL!	3	3	0	0
0	0	#NULL!	1	1	0	0
0	0	#NULL!	1	1	0	0
0	0	#NULL!	1	1	0	1
0	0	#NULL!	3	3	1	1
0	0	#NULL!	1	2	0	0
0	0	#NULL!	3	2	0	1
0	0	#NULL!	1	1	3	4
0	0	#NULL!	3	3	1	0
0	0	#NULL!	3	1	0	0
0	0	#NULL!	4	3	0	0
0	0	#NULL!	3	2	1	2
0	0	#NULL!	3	2	0	0
0	0	#NULL!	1	1	0	0
0	0	#NULL!	3	3	0	1
0	0	#NULL!	4	3	0	0
0	0	#NULL!	1	1	0	0
0	0	#NULL!	1	1	0	0
0	0	#NULL!	2	1	1	2
0	0	#NULL!	1	1	0	0
0	0	#NULL!	3	1	0	0

0	0	#NULL!	4	2	1	2
0	0	#NULL!	1	1	0	0
0	0	#NULL!	2	3	4	4
0	0	#NULL!	1	1	0	0
0	0	#NULL!	1	2	0	1
0	0	#NULL!	2	1	0	0
0	0	#NULL!	3	1	0	0
0	0	#NULL!	4	4	1	4
0	0	#NULL!	2	2	0	5
0	0	#NULL!	3	2	0	0
0	0	#NULL!	1	1	0	0
0	0	#NULL!	4	3	0	0
0	0	#NULL!	1	1	1	1
0	0	#NULL!	3	2	0	0
0	0	#NULL!	3	3	0	0
0	0	#NULL!	3	2	0	0
0	0	#NULL!	1	1	0	0
0	0	#NULL!	2	1	0	0
0	0	#NULL!	3	2	0	0
0	0	#NULL!	3	3	0	0
0	0	#NULL!	2	2	0	0
0	0	#NULL!	1	2	0	0
0	0	#NULL!	3	2	0	0
0	0	#NULL!	4	3	0	0
0	0	#NULL!	5	5	5	5
0	0	#NULL!	1	1	0	0
0	0	#NULL!	1	1	0	0
0	0	#NULL!	1	1	0	2
0	0	#NULL!	1	1	0	0
0	0	#NULL!	1	1	0	0
0	1	#NULL!	1	1	0	0
0	0	#NULL!	3	2	0	0
0	0	#NULL!	1	1	0	0
0	0	#NULL!	5	3	0	0
0	0	#NULL!	1	1	0	0
0	0	#NULL!	1	1	0	1
0	0	#NULL!	3	2	0	99
0	0	#NULL!	1	1	0	0
0	0	#NULL!	3	3	0	0
0	0	#NULL!	2	2	0	0
0	0	#NULL!	1	1	0	0
1	0	#NULL!	1	1	0	0
0	0	#NULL!	2	2	0	0
0	0	#NULL!	1	1	0	0
0	0	#NULL!	3	3	0	1
0	0	#NULL!	3	3	2	0
0	0	#NULL!	4	2	0	1
0	0	#NULL!	1	1	0	1

0	0	#NULL!	1	1	0	0
0	0	#NULL!	3	3	0	2
0	0	#NULL!	3	2	0	0
0	0	#NULL!	1	1	0	0
0	0	#NULL!	1	1	0	0
0	0	#NULL!	3	1	0	0
0	0	#NULL!	2	2	0	0
0	0	#NULL!	3	2	0	1
0	0	#NULL!	1	1	0	0
0	0	#NULL!	1	3	0	0
0	0	#NULL!	1	1	0	0
0	0	#NULL!	1	1	0	0
0	0	#NULL!	1	1	0	0
0	0	#NULL!	3	3	0	0
0	0	#NULL!	1	1	0	0
0	0	#NULL!	1	1	0	0
0	0	#NULL!	1	1	0	0
0	0	#NULL!	1	1	0	0
0	0	#NULL!	1	1	0	0
0	0	#NULL!	1	1	0	0
0	0	#NULL!	1	1	0	0
0	0	#NULL!	1	1	0	0
0	0	#NULL!	1	1	0	0
0	0	#NULL!	1	3	0	0
0	0	#NULL!	1	2	0	0
0	0	#NULL!	3	3	0	0
0	0	#NULL!	1	1	0	0
0	0	#NULL!	1	1	0	0
0	0	#NULL!	1	1	0	0
0	0	#NULL!	3	1	0	0
0	0	#NULL!	1	1	0	0
0	0	#NULL!	1	1	0	0
0	0	#NULL!	3	2	0	0
0	0	#NULL!	5	3	0	0
0	0	#NULL!	3	3	0	0
0	0	#NULL!	3	3	0	0
0	0	#NULL!	3	2	0	0
0	0	#NULL!	2	1	0	0
0	0	#NULL!	3	3	0	0
0	0	#NULL!	1	1	0	0
0	0	#NULL!	1	1	0	0
0	0	#NULL!	2	1	0	0
0	0	#NULL!	1	1	0	0
0	0	#NULL!	1	1	0	0
0	0	#NULL!	1	1	0	0
0	0	#NULL!	1	1	0	0
0	0	#NULL!	1	1	0	1
0	0	#NULL!	4	2	0	0

0	0	#NULL!	1	1	0	0
0	0	#NULL!	1	1	0	0
0	0	#NULL!	3	2	0	0
0	0	#NULL!	4	2	0	0
0	0	#NULL!	3	3	0	0
0	0	#NULL!	4	1	0	0
0	0	#NULL!	1	1	0	0
0	0	#NULL!	1	1	0	0
0	0	#NULL!	1	1	0	0
0	0	#NULL!	1	1	0	0
0	0	#NULL!	1	1	0	0
0	0	#NULL!	1	1	0	0
0	0	#NULL!	1	1	0	0
0	0	#NULL!	5	1	0	0
0	0	#NULL!	1	1	0	0
0	0	#NULL!	2	5	0	0
0	0	#NULL!	4	1	0	0
0	0	#NULL!	1	1	0	0
0	0	#NULL!	3	2	0	0
0	0	#NULL!	1	1	0	1
0	0	#NULL!	1	1	0	0
0	0	#NULL!	1	1	0	0
0	0	#NULL!	3	2	0	0
0	0	#NULL!	3	3	0	0
0	0	#NULL!	3	2	0	0
0	0	#NULL!	5	5	0	0
0	0	#NULL!	4	2	0	0
0	0	#NULL!	3	2	0	0
0	0	#NULL!	1	1	0	0
0	0	#NULL!	1	1	0	0
0	0	#NULL!	1	1	0	0
0	0	#NULL!	1	2	0	1
0	0	#NULL!	1	1	0	0
0	0	#NULL!	3	1	0	0
0	0	#NULL!	2	1	0	0
0	0	#NULL!	1	1	0	0
0	0	#NULL!	1	1	99	99
0	0	#NULL!	1	1	0	0
0	0	#NULL!	1	1	0	0
0	0	#NULL!	2	2	0	0
0	0	#NULL!	3	2	0	0
0	0	#NULL!	1	1	0	1
0	0	#NULL!	3	1	0	0
0	0	#NULL!	1	1	0	0
0	0	#NULL!	1	1	0	0
0	0	#NULL!	2	3	0	0
0	0	#NULL!	3	2	0	0
0	0	#NULL!	3	2	0	0

0	0	#NULL!	3	2	0	0
0	0	#NULL!	3	2	0	0
0	0	#NULL!	1	1	0	0
0	0	#NULL!	3	3	0	0
0	0	#NULL!	1	1	0	0
0	0	#NULL!	3	3	0	0
0	0	#NULL!	3	3	0	0
0	0	#NULL!	1	1	0	0
0	0	#NULL!	3	3	0	0
0	0	#NULL!	5	1	0	0
0	0	#NULL!	4	1	0	0
0	0	#NULL!	1	1	0	0
0	0	#NULL!	1	1	0	0
0	0	#NULL!	1	1	0	0
0	0	#NULL!	1	1	0	0
0	0	#NULL!	3	3	0	0
0	0	#NULL!	3	3	0	0
0	0	#NULL!	2	2	0	0
0	0	#NULL!	1	1	0	0
0	0	#NULL!	4	3	0	0
0	0	#NULL!	3	1	0	0
0	0	#NULL!	1	2	0	0
0	0	#NULL!	1	1	0	0
1	0	#NULL!	99	99	0	0
0	0	#NULL!	3	2	0	0
0	0	#NULL!	1	1	0	0
0	0	#NULL!	1	1	0	0
0	0	#NULL!	1	1	1	2
0	0	#NULL!	3	2	0	0
0	0	#NULL!	99	1	0	0
0	0	#NULL!	3	2	0	0
0	0	#NULL!	2	2	0	0
0	0	#NULL!	3	3	0	0
0	0	#NULL!	4	1	0	0
0	0	#NULL!	1	1	0	0
0	0	#NULL!	1	1	0	0
0	0	#NULL!	1	1	0	0
0	1	#NULL!	99	99	99	99
0	0	#NULL!	3	3	0	1
0	0	#NULL!	1	1	0	0
0	0	#NULL!	3	2	0	0
0	0	#NULL!	3	1	0	0
0	0	#NULL!	1	1	0	0
0	0	#NULL!	3	2	0	0
0	0	#NULL!	1	1	0	0
0	0	#NULL!	2	1	0	0
0	0	#NULL!	1	1	0	1
0	0	#NULL!	4	3	0	0

0	0	#NULL!	1	1	0	0
0	0	#NULL!	5	3	0	0
0	0	#NULL!	1	1	0	0
0	0	#NULL!	1	1	0	0
0	0	#NULL!	4	3	0	0
0	0	#NULL!	3	3	0	0
0	0	#NULL!	3	3	0	0
0	0	#NULL!	3	3	0	0
0	0	#NULL!	4	3	0	0
0	0	#NULL!	4	2	0	0
0	0	#NULL!	3	2	0	1
0	0	#NULL!	3	2	0	0
0	0	#NULL!	4	3	0	0
0	0	#NULL!	2	1	0	1
0	0	#NULL!	3	2	0	0
0	0	#NULL!	1	1	0	0
0	0	#NULL!	1	1	0	0
0	0	#NULL!	2	1	0	0
0	0	#NULL!	3	1	0	0
0	0	#NULL!	1	2	0	0
0	0	#NULL!	3	1	0	0
0	0	#NULL!	1	2	0	0
0	0	#NULL!	2	2	99	99
0	0	#NULL!	1	1	0	0
0	0	#NULL!	1	2	0	0
0	0	#NULL!	1	1	0	0
0	0	#NULL!	1	1	0	0
0	0	#NULL!	3	2	0	0
0	0	#NULL!	1	1	0	0
0	0	#NULL!	3	1	0	0
0	0	#NULL!	1	1	0	0
0	0	#NULL!	1	1	0	0
0	0	#NULL!	1	1	0	0
0	0	#NULL!	1	1	0	0
0	0	#NULL!	1	1	0	0
0	0	#NULL!	2	1	0	0
0	0	#NULL!	1	1	0	0
0	0	#NULL!	3	2	0	0
0	0	#NULL!	1	1	0	0
0	0	#NULL!	2	3	0	0
0	0	#NULL!	1	2	0	0
0	0	#NULL!	1	1	0	0
0	0	#NULL!	3	3	0	0
0	0	#NULL!	1	1	0	0
0	0	#NULL!	3	3	0	0
0	0	#NULL!	3	1	0	0
0	0	#NULL!	3	3	0	0
0	0	#NULL!	2	2	0	0

0	0	#NULL!	1	1	0	0
0	0	#NULL!	1	1	0	1
0	0	#NULL!	1	1	0	0
0	0	#NULL!	2	1	0	0
0	0	#NULL!	1	1	0	0
0	0	#NULL!	1	2	0	0
0	0	#NULL!	1	1	0	0
0	0	#NULL!	1	1	0	0
0	0	#NULL!	2	1	0	0
0	0	#NULL!	3	1	0	0
1	0	#NULL!	2	1	0	0
0	0	#NULL!	3	1	0	0
0	0	#NULL!	1	1	0	0
0	0	#NULL!	1	1	0	0
0	0	#NULL!	1	1	0	0
0	0	#NULL!	1	1	0	0
0	0	#NULL!	3	3	0	1
0	0	#NULL!	3	3	0	0
0	0	#NULL!	3	3	0	0
0	0	#NULL!	2	2	0	0
0	0	#NULL!	4	2	0	0
0	0	#NULL!	3	1	0	0
0	0	#NULL!	3	3	0	0
0	0	#NULL!	4	3	0	0
0	0	#NULL!	3	4	0	0
0	0	#NULL!	2	1	0	0
0	0	#NULL!	1	2	0	0
0	0	#NULL!	1	1	0	0
0	0	#NULL!	3	3	99	99
0	0	#NULL!	3	2	1	0
0	0	#NULL!	1	1	0	0
0	0	#NULL!	3	2	0	0
0	0	#NULL!	1	1	0	0
0	0	#NULL!	1	1	0	0
0	0	#NULL!	1	1	0	0
0	0	#NULL!	1	1	0	0
0	0	#NULL!	1	1	0	0
0	0	#NULL!	3	3	5	5
0	0	#NULL!	2	2	0	0
0	0	#NULL!	1	1	0	0
0	0	#NULL!	1	1	0	0
0	0	#NULL!	2	2	0	0
0	0	#NULL!	1	1	0	0
0	0	#NULL!	5	5	0	1
0	0	#NULL!	3	1	0	0
0	0	#NULL!	4	2	0	0
0	0	#NULL!	1	1	0	0
0	0	#NULL!	4	4	1	1

0	0	#NULL!	3	3	2	5
0	0	#NULL!	3	2	1	5
0	0	#NULL!	3	2	0	0
0	0	#NULL!	1	1	0	0
0	0	#NULL!	1	1	0	1
0	0	#NULL!	1	1	0	0
0	0	#NULL!	1	1	0	0
0	0	#NULL!	1	1	1	0
0	0	#NULL!	5	1	0	0
0	0	#NULL!	1	1	0	0
0	0	#NULL!	2	2	1	1
0	0	#NULL!	3	3	0	0
0	0	#NULL!	1	2	0	2
0	0	#NULL!	3	2	0	1
0	0	#NULL!	3	3	3	3
0	0	#NULL!	1	2	0	1
0	0	#NULL!	3	1	0	0
0	0	#NULL!	1	1	0	2
0	0	#NULL!	2	1	2	0
0	0	#NULL!	1	1	0	0
0	0	#NULL!	1	2	3	3
0	0	#NULL!	3	2	0	0
0	0	#NULL!	2	2	0	99
0	0	#NULL!	1	1	0	1
0	0	#NULL!	1	1	0	0
0	0	#NULL!	1	2	99	99
0	0	#NULL!	3	2	0	0
0	0	#NULL!	2	1	0	0
0	0	#NULL!	3	2	0	0
1	0	#NULL!	3	2	0	0
0	0	#NULL!	2	1	0	2
0	0	#NULL!	2	1	0	0
0	0	#NULL!	1	1	0	0
0	0	#NULL!	3	2	0	0
0	0	#NULL!	3	3	0	99
0	0	#NULL!	3	2	0	0
0	0	#NULL!	1	1	0	0
0	0	#NULL!	3	2	0	0
0	0	#NULL!	3	3	0	0
0	0	#NULL!	1	3	0	1
0	0	#NULL!	3	3	0	1
0	0	#NULL!	1	1	0	0
0	0	#NULL!	2	3	0	0
0	0	#NULL!	3	1	0	0
0	0	#NULL!	3	2	0	0
0	0	#NULL!	1	1	0	0
0	0	#NULL!	4	2	0	0
0	0	#NULL!	2	1	0	0

0	0	#NULL!	4	2	0	0
0	0	#NULL!	1	1	0	1
0	0	#NULL!	4	3	0	0
0	0	#NULL!	3	3	0	0
0	0	#NULL!	3	2	0	0
0	0	#NULL!	1	1	0	0
0	0	#NULL!	5	5	0	0
0	0	#NULL!	1	1	0	0
0	0	#NULL!	3	3	0	0
0	0	#NULL!	4	3	0	1
0	0	#NULL!	3	2	1	0
0	0	#NULL!	1	2	0	0
0	0	#NULL!	1	1	0	0
0	0	#NULL!	2	1	1	3
0	0	#NULL!	3	3	0	0
0	0	#NULL!	3	2	0	0
0	0	#NULL!	1	1	0	0
0	0	#NULL!	1	1	0	0
0	0	#NULL!	4	2	0	0
0	0	#NULL!	1	1	0	0
0	0	#NULL!	2	1	1	1
0	0	#NULL!	1	1	1	3
0	0	#NULL!	3	3	0	0
0	0	#NULL!	1	1	0	0
0	0	#NULL!	1	1	0	0
0	0	#NULL!	3	2	0	0
0	0	#NULL!	3	2	0	0
0	0	#NULL!	1	1	3	99
0	0	#NULL!	3	1	0	0
0	0	#NULL!	1	1	0	0
0	0	#NULL!	5	3	0	0
0	0	#NULL!	1	1	0	0
0	0	#NULL!	1	1	0	0
0	0	#NULL!	1	1	0	0
0	0	#NULL!	1	1	0	0
0	0	#NULL!	1	1	0	0
0	0	#NULL!	3	1	0	0
0	0	#NULL!	1	2	1	1
0	0	#NULL!	3	2	0	0
0	0	#NULL!	4	2	0	0
0	0	#NULL!	3	2	0	0
0	0	#NULL!	1	1	0	2
0	0	#NULL!	1	1	0	0
0	0	#NULL!	3	2	0	0
0	0	#NULL!	2	2	0	1
0	0	#NULL!	4	2	1	0
0	0	#NULL!	2	1	0	0
0	0	#NULL!	1	1	99	99

0	0	#NULL!	1	1	0	0
0	0	#NULL!	1	1	0	0
0	0	#NULL!	1	1	0	0
0	0	#NULL!	1	1	0	0
0	0	#NULL!	1	1	0	0
0	0	#NULL!	1	1	0	0
0	0	#NULL!	1	1	0	0
1	0	#NULL!	3	3	0	1
0	0	#NULL!	2	1	0	0
0	0	#NULL!	3	2	0	1
0	0	#NULL!	3	1	0	0
0	0	#NULL!	1	1	1	1
0	0	#NULL!	3	3	0	0
0	0	#NULL!	3	3	0	0
0	0	#NULL!	1	1	0	0
0	0	#NULL!	1	1	2	1
0	0	#NULL!	2	2	0	0
0	0	#NULL!	3	2	0	2
0	0	#NULL!	5	3	99	99
0	0	#NULL!	5	5	99	99
0	0	#NULL!	3	1	1	1
0	0	#NULL!	2	2	1	0
0	0	#NULL!	2	1	0	0
0	0	#NULL!	3	3	0	0
0	0	#NULL!	5	3	0	0
0	0	#NULL!	2	1	0	0
0	0	#NULL!	3	2	0	0
0	0	#NULL!	1	1	0	0
0	1	#NULL!	2	1	0	0
0	0	#NULL!	2	2	0	0
0	0	#NULL!	1	1	0	0
0	0	#NULL!	3	3	0	0
0	0	#NULL!	4	2	0	0
0	0	#NULL!	3	1	0	0
0	0	#NULL!	4	1	0	0
0	0	#NULL!	3	3	0	1
0	0	#NULL!	4	3	0	0
0	0	#NULL!	4	3	0	0
0	0	#NULL!	2	1	0	0
0	0	#NULL!	3	2	0	0
0	0	#NULL!	2	1	0	0
0	0	#NULL!	3	3	0	0
0	0	#NULL!	1	2	0	0
0	0	#NULL!	1	1	0	0
0	0	#NULL!	1	1	0	0
0	0	#NULL!	1	1	0	5
0	0	#NULL!	1	1	0	0
0	0	#NULL!	3	3	0	1

0	0	#NULL!	1	1	0	0
0	0	#NULL!	2	1	0	0
0	0	#NULL!	1	1	0	0
0	0	#NULL!	2	1	0	1
0	0	#NULL!	3	3	1	2
0	0	#NULL!	5	3	0	0
0	0	#NULL!	1	1	0	2
0	0	#NULL!	1	1	0	0
0	0	#NULL!	3	2	0	0
0	0	#NULL!	2	1	0	0
0	0	#NULL!	1	1	0	0
0	0	#NULL!	1	1	0	0
0	0	#NULL!	5	3	0	0
0	0	#NULL!	3	3	0	0
0	0	#NULL!	1	1	0	0
0	0	#NULL!	1	1	1	0
0	0	#NULL!	1	1	0	0
0	0	#NULL!	4	2	0	99
0	0	#NULL!	3	2	0	0
0	0	#NULL!	2	2	3	2
0	0	#NULL!	2	2	0	0
0	0	#NULL!	3	1	0	0
0	0	#NULL!	1	1	0	1
0	0	#NULL!	1	1	1	1
0	0	#NULL!	1	1	0	0
0	0	#NULL!	2	2	1	1
0	0	#NULL!	3	3	0	1
0	0	#NULL!	5	3	0	1
0	0	#NULL!	1	3	0	0
0	0	#NULL!	2	2	2	2
0	0	#NULL!	2	2	0	1
0	0	#NULL!	3	2	0	2
0	0	#NULL!	3	2	0	1
0	0	#NULL!	1	1	0	0
0	0	#NULL!	1	1	0	0
0	0	#NULL!	3	2	0	1
0	0	#NULL!	3	3	0	1
0	0	#NULL!	3	3	0	1
1	0	#NULL!	3	2	0	0
0	0	#NULL!	3	3	0	0
0	0	#NULL!	2	1	0	0
0	0	#NULL!	3	2	1	2
0	0	#NULL!	2	1	1	3
0	0	#NULL!	3	3	0	0
0	0	#NULL!	3	2	0	1
0	0	#NULL!	1	1	0	0
0	0	#NULL!	2	3	1	2
1	0	#NULL!	3	2	0	0

0	0	#NULL!	1	1	0	0
0	0	#NULL!	4	3	0	0
0	0	#NULL!	1	1	0	0
0	0	#NULL!	1	1	0	0
0	1	#NULL!	1	1	0	1
0	0	#NULL!	5	2	0	1
0	0	#NULL!	3	3	0	0
0	0	#NULL!	1	1	0	0
0	0	#NULL!	1	1	0	0
0	0	#NULL!	1	1	0	0
0	0	#NULL!	1	3	0	0
0	0	#NULL!	1	1	0	0
0	0	#NULL!	1	3	0	0
0	0	#NULL!	2	1	0	0
0	0	#NULL!	5	4	0	2
0	0	#NULL!	1	1	0	0
0	0	#NULL!	3	3	0	0
0	0	#NULL!	2	2	0	0
0	0	#NULL!	3	3	0	0
0	0	#NULL!	3	3	0	0
0	0	#NULL!	5	2	0	0
0	0	#NULL!	1	1	0	0
0	0	#NULL!	4	2	0	0
0	0	#NULL!	2	2	99	1
0	0	#NULL!	1	1	0	0
0	0	#NULL!	3	1	0	0
0	0	#NULL!	1	1	0	0
0	0	#NULL!	2	2	0	0
0	0	#NULL!	3	1	0	0
0	0	#NULL!	1	1	0	0
0	0	#NULL!	1	1	0	0
0	0	#NULL!	4	3	0	99
0	0	#NULL!	3	1	0	5
0	0	#NULL!	1	1	0	0
0	0	#NULL!	1	1	0	0
0	0	#NULL!	1	1	0	0
0	0	#NULL!	3	2	0	0
0	0	#NULL!	2	3	0	0
0	0	#NULL!	3	3	0	0
0	0	#NULL!	1	1	0	0
0	0	#NULL!	2	1	0	0
0	0	#NULL!	1	1	0	0
0	0	#NULL!	3	3	0	0
0	0	#NULL!	3	3	0	0
0	0	#NULL!	3	2	0	0
0	0	#NULL!	3	3	0	1
0	0	#NULL!	3	3	0	0
0	0	#NULL!	3	2	0	2

0	0	#NULL!	1	1	0	0
0	0	#NULL!	3	2	0	0
0	0	#NULL!	1	1	0	0
0	0	#NULL!	5	1	0	0
0	0	#NULL!	3	3	0	0
0	0	#NULL!	3	2	0	0
0	0	#NULL!	4	1	0	0
0	0	#NULL!	1	1	0	0
0	0	#NULL!	1	1	0	0
0	0	#NULL!	2	2	0	0
0	0	#NULL!	3	2	4	5
0	0	#NULL!	3	3	0	0
0	0	#NULL!	4	3	0	0
0	0	#NULL!	4	2	0	0
0	0	#NULL!	1	1	5	0
0	0	#NULL!	2	2	0	0
0	0	#NULL!	3	1	0	0
0	0	#NULL!	3	3	0	0
1	0	#NULL!	3	1	0	0
0	0	#NULL!	3	2	0	0
0	0	#NULL!	3	2	0	0
0	0	#NULL!	3	2	0	0
0	0	#NULL!	3	3	0	1
0	0	#NULL!	3	3	0	1
0	0	#NULL!	1	1	0	0
0	0	#NULL!	3	2	0	1
0	0	#NULL!	2	2	2	1
0	0	#NULL!	1	1	0	0
0	0	#NULL!	1	1	0	0
0	0	#NULL!	4	2	0	1
0	0	#NULL!	1	1	0	0
0	0	#NULL!	1	1	0	0
0	0	#NULL!	2	1	0	1
0	0	#NULL!	2	2	0	0
0	0	#NULL!	1	1	0	0
0	0	#NULL!	3	3	0	0
0	0	#NULL!	3	1	0	0
0	0	#NULL!	1	1	0	0
0	0	#NULL!	1	1	0	0
0	0	#NULL!	1	2	1	1
0	0	#NULL!	3	1	0	0
0	0	#NULL!	2	2	0	0
0	0	#NULL!	1	1	0	0
0	0	#NULL!	3	1	0	0
0	0	#NULL!	1	1	0	0
0	0	#NULL!	1	2	0	0
0	0	#NULL!	99	1	0	0
0	0	#NULL!	3	3	0	0

0	0	#NULL!	1	1	0	0
0	0	#NULL!	5	1	0	0
0	0	#NULL!	1	1	0	0
0	0	#NULL!	3	1	0	0
0	0	#NULL!	2	1	0	0
0	0	#NULL!	4	2	0	0
0	0	#NULL!	1	1	0	0
0	0	#NULL!	3	1	0	0
0	0	#NULL!	1	1	0	0
0	0	#NULL!	3	2	0	0
0	0	#NULL!	3	1	0	99
0	0	#NULL!	1	1	0	0
0	0	#NULL!	1	1	0	0
0	0	#NULL!	3	1	0	0
0	0	#NULL!	3	3	0	0
1	0	#NULL!	1	1	4	1
0	0	#NULL!	1	1	0	0
0	0	#NULL!	2	1	0	0
0	0	#NULL!	1	1	0	0
0	0	#NULL!	1	1	0	0
0	0	#NULL!	1	1	0	0
0	0	#NULL!	5	2	0	0
0	0	#NULL!	1	1	0	0
0	0	#NULL!	4	3	0	0
0	0	#NULL!	2	2	0	0
0	0	#NULL!	1	1	0	0
0	0	#NULL!	3	2	0	0
0	0	#NULL!	1	1	0	0
0	0	#NULL!	3	3	0	0
0	0	#NULL!	1	1	0	0
0	0	#NULL!	1	1	0	0
0	0	#NULL!	3	2	0	99
0	0	#NULL!	1	1	0	0
0	0	#NULL!	3	3	0	0
0	0	#NULL!	1	1	0	0
0	0	#NULL!	3	2	1	1
0	0	#NULL!	4	3	0	0
0	0	#NULL!	1	1	0	0
0	0	#NULL!	1	1	0	0
0	0	#NULL!	1	1	0	0
0	0	#NULL!	3	2	0	0
0	0	#NULL!	2	2	0	1
0	0	#NULL!	1	1	4	3
0	0	#NULL!	3	1	0	0
0	0	#NULL!	1	1	0	0
0	0	#NULL!	3	3	0	0
0	0	#NULL!	2	99	0	0
0	0	#NULL!	3	2	0	0

0	0	#NULL!	1	1	0	0
1	0	#NULL!	1	2	1	5
0	0	#NULL!	1	1	0	0
0	0	#NULL!	1	1	0	0
0	0	#NULL!	3	3	0	0
0	0	#NULL!	3	3	0	0
0	0	#NULL!	1	1	0	0
0	0	#NULL!	1	1	0	0
0	0	#NULL!	1	1	0	0
0	0	#NULL!	1	1	0	0
0	0	#NULL!	4	2	1	1
0	0	#NULL!	1	1	4	0
0	0	#NULL!	1	1	0	0
0	0	#NULL!	1	1	0	1
0	0	#NULL!	3	2	0	0
0	0	#NULL!	3	2	0	0
0	0	#NULL!	1	1	0	0
0	0	#NULL!	3	3	0	1
0	0	#NULL!	1	1	0	0
0	0	#NULL!	3	2	0	0
0	0	#NULL!	3	1	0	0
0	0	#NULL!	3	3	0	0
0	0	#NULL!	3	3	0	0
0	0	#NULL!	1	1	0	0
0	0	#NULL!	3	2	0	0
0	0	#NULL!	1	1	0	0
0	0	#NULL!	1	1	0	0
0	0	#NULL!	1	1	0	0
0	0	#NULL!	3	1	0	0
0	0	#NULL!	1	1	0	0
0	0	#NULL!	3	3	0	0
0	0	#NULL!	3	2	0	0
0	0	#NULL!	1	1	0	0
0	0	#NULL!	1	1	0	0
0	0	#NULL!	3	1	0	3
0	0	#NULL!	4	3	0	0
0	0	#NULL!	5	5	0	1
0	0	#NULL!	1	1	0	0
0	0	#NULL!	5	3	0	2
0	0	#NULL!	1	1	0	0
0	0	#NULL!	1	2	1	0
0	0	#NULL!	3	4	0	0
0	0	#NULL!	3	1	2	3
0	0	#NULL!	1	1	0	0
0	0	#NULL!	3	1	0	0
0	0	#NULL!	5	2	0	0
0	0	#NULL!	1	1	0	0
0	0	#NULL!	2	2	0	0

0	0	#NULL!	1	1	0	0
0	0	#NULL!	2	1	2	2
0	0	#NULL!	3	2	1	4
0	0	#NULL!	1	1	0	0
0	0	#NULL!	1	1	0	0
0	0	#NULL!	1	2	0	0
0	0	#NULL!	3	3	0	0
0	0	#NULL!	3	3	0	0
0	0	#NULL!	3	2	0	0
0	0	#NULL!	3	3	0	0
1	0	#NULL!	2	2	0	0
0	0	#NULL!	1	1	0	0
0	0	#NULL!	1	1	0	0
0	0	#NULL!	3	2	0	1
1	0	#NULL!	5	1	0	0
0	0	#NULL!	4	3	0	0
0	0	#NULL!	1	1	5	99
0	0	#NULL!	3	2	0	0
0	0	#NULL!	5	3	0	0
0	0	#NULL!	4	1	0	1
1	0	#NULL!	2	2	0	0
0	0	#NULL!	1	1	0	0
0	0	#NULL!	4	2	0	0
0	0	#NULL!	3	2	0	0
0	0	#NULL!	4	1	0	0
0	0	#NULL!	3	3	2	1
0	0	#NULL!	4	3	0	0
0	0	#NULL!	3	3	0	0
0	0	#NULL!	3	2	0	0
0	0	#NULL!	3	2	0	0
0	0	#NULL!	1	1	0	0
0	0	#NULL!	1	1	0	0
0	0	#NULL!	1	1	0	1
0	0	#NULL!	1	1	0	0
0	0	#NULL!	3	2	1	2
0	0	#NULL!	1	99	0	0
0	0	#NULL!	4	2	0	0
0	0	#NULL!	1	3	0	0
0	0	#NULL!	1	1	1	1
0	0	#NULL!	1	1	0	0
0	0	#NULL!	1	1	0	0
0	0	#NULL!	1	1	0	0
0	0	#NULL!	4	3	0	0
0	0	#NULL!	1	1	0	0
0	0	#NULL!	1	1	0	0
0	0	#NULL!	4	3	0	2
0	0	#NULL!	2	1	0	0
0	0	#NULL!	1	1	0	0

0	0	#NULL!	1	1	0	0
0	0	#NULL!	3	3	1	2
0	0	#NULL!	3	2	0	0
0	0	#NULL!	3	2	0	0
0	0	#NULL!	1	1	0	0
0	0	#NULL!	1	1	0	0
0	0	#NULL!	4	3	0	0
0	0	#NULL!	1	1	0	0
0	0	#NULL!	1	1	1	0
0	0	#NULL!	1	1	1	0
0	0	#NULL!	3	2	0	0
1	0	#NULL!	2	#NULL!	0	0
0	0	#NULL!	1	1	0	0
0	0	#NULL!	1	1	0	0
0	0	#NULL!	1	1	0	0
0	0	#NULL!	1	1	0	0
0	0	#NULL!	4	2	0	0
0	0	#NULL!	4	2	0	0
0	0	#NULL!	1	1	0	0
0	0	#NULL!	1	1	0	0
0	0	#NULL!	3	2	0	0
0	0	#NULL!	1	1	0	0
0	0	#NULL!	1	1	0	0
0	0	#NULL!	1	1	0	0
0	0	#NULL!	3	2	0	0
0	0	#NULL!	3	2	0	0
0	0	#NULL!	1	1	0	0
0	0	#NULL!	3	2	0	0
0	0	#NULL!	1	1	0	0
0	0	#NULL!	2	2	2	2
0	0	#NULL!	2	2	0	0
0	0	#NULL!	4	4	0	5
0	0	#NULL!	4	2	0	1
0	0	#NULL!	1	2	0	0
0	0	#NULL!	1	1	0	0
0	0	#NULL!	1	1	5	5
0	0	#NULL!	3	1	0	0
0	0	#NULL!	1	1	0	0
0	0	#NULL!	3	3	0	0
0	0	#NULL!	5	5	0	0
0	0	#NULL!	1	1	0	0
0	0	#NULL!	3	3	0	0
0	0	#NULL!	1	1	0	4
0	0	#NULL!	5	1	0	0
0	0	#NULL!	1	1	0	5
0	0	#NULL!	1	1	0	0
0	0	#NULL!	1	1	0	0
0	0	#NULL!	5	3	0	0

0	0	#NULL!	2	1	0	0
0	0	#NULL!	1	1	0	0
0	0	#NULL!	1	1	0	0
0	0	#NULL!	1	1	0	0
0	0	#NULL!	1	2	0	0
0	0	#NULL!	1	1	0	0
0	0	#NULL!	1	1	1	2
0	0	#NULL!	1	1	0	0
0	0	#NULL!	5	#NULL!	0	0
0	0	#NULL!	1	1	5	4
0	0	#NULL!	5	1	0	0
0	0	#NULL!	1	2	2	2
0	0	#NULL!	#NULL!	#NULL!	#NULL!	#NULL!
0	0	#NULL!	1	1	0	0
0	0	#NULL!	1	1	0	0
0	0	#NULL!	3	3	0	0
0	0	#NULL!	1	1	0	0
0	0	#NULL!	3	3	0	0
0	0	#NULL!	3	3	0	1
0	0	#NULL!	3	2	0	0
0	0	#NULL!	3	2	0	1
0	0	#NULL!	5	2	1	3
0	0	#NULL!	3	3	0	0
0	0	#NULL!	4	1	0	0
1	0	#NULL!	5	5	0	0
0	0	#NULL!	3	1	0	0
0	0	#NULL!	1	1	1	1
0	0	#NULL!	1	1	0	0
0	0	#NULL!	2	2	0	0
0	0	#NULL!	3	1	0	0
0	0	#NULL!	3	2	0	0
0	0	#NULL!	3	2	0	0
0	0	#NULL!	1	1	0	0
0	0	#NULL!	3	1	0	0
0	0	#NULL!	1	1	0	0
0	0	#NULL!	4	2	0	0
0	0	#NULL!	1	1	0	0
0	0	#NULL!	4	1	0	0
0	0	#NULL!	3	2	0	0
0	0	#NULL!	1	1	0	0
0	0	#NULL!	3	2	0	0
0	0	#NULL!	3	1	0	0
0	0	#NULL!	3	3	0	0
0	0	#NULL!	1	1	0	0
0	0	#NULL!	1	1	1	5
0	0	#NULL!	1	2	0	0
0	0	#NULL!	4	2	0	0
0	0	#NULL!	3	3	0	0

0	0	#NULL!	1	1	0	0
0	0	#NULL!	3	2	0	0
0	0	#NULL!	1	1	0	0
0	0	#NULL!	#NULL!	#NULL!	0	0
0	0	#NULL!	3	3	0	0
0	0	#NULL!	3	2	0	0
0	0	#NULL!	3	2	0	0
0	0	#NULL!	3	3	0	0
0	0	#NULL!	3	2	0	0
0	0	#NULL!	1	1	0	0
0	0	#NULL!	1	1	0	0
0	0	#NULL!	1	1	0	0
0	0	#NULL!	3	1	0	0
0	0	#NULL!	4	2	0	0
0	0	#NULL!	4	2	0	0
0	0	#NULL!	4	2	0	0
0	0	#NULL!	#NULL!	#NULL!	#NULL!	#NULL!
0	0	#NULL!	1	1	0	0
0	0	#NULL!	1	1	0	1
0	0	#NULL!	3	3	0	0
0	0	#NULL!	2	1	0	0

subusec_pre	subused_pre	subusee_pre	subusef_pre	subuseg_pre	subuseh_pre	eversex_pre	sexpart_pre
0	0	0	0	0	0	0	0
0	0	0	0	0	0	0	0
0	0	0	0	0	0	0	0
0	0	0	0	0	0	0	0
1	0	0	0	0	0	0	0
0	0	0	0	0	0	99	99
1	0	1	0	0	0	1	1
2	0	0	0	1	0	0	7
99	99	99	99	99	99	2	0
0	0	0	0	1	0	2	0
0	0	0	0	1	0	0	0
1	0	0	0	0	0	0	0
1	0	0	0	0	0	0	0
0	0	0	0	0	0	2	0
0	0	0	0	0	0	0	0
1	0	0	0	0	0	0	0
0	0	0	0	0	0	0	0
99	0	0	0	0	0	2	7
0	0	0	0	0	99	0	0
2	0	0	3	99	99	1	1
0	0	0	0	0	0	0	99
0	0	0	0	0	0	99	99
0	0	0	0	0	0	0	0
1	0	0	0	0	0	0	0
0	0	0	0	0	0	0	0
0	0	0	0	0	0	0	0
1	0	0	0	0	0	0	0
0	0	0	0	0	0	0	0
0	0	0	0	0	0	0	0
0	0	0	0	0	0	0	0
0	0	0	0	0	0	0	0
99	5	5	0	0	0	1	4
1	0	0	0	0	0	0	0
0	0	0	0	0	0	0	0
0	0	0	0	0	0	0	99
0	0	0	0	0	0	0	0
0	0	0	0	0	0	0	0
0	0	0	0	0	0	0	0
1	0	0	0	0	0	0	0
2	4	0	0	2	0	0	0
0	0	0	0	0	0	0	0
0	0	0	0	0	0	0	0
0	0	0	0	0	0	0	0
0	0	0	0	0	0	0	0
0	0	0	0	0	0	0	0
0	0	0	0	0	0	0	0
0	0	0	0	0	0	1	1

5	0	0	0	2	0	0	0
1	0	0	0	0	0	0	0
1	0	0	0	0	0	0	0
0	0	0	0	0	0	0	0
0	0	0	0	0	0	0	0
0	0	0	0	0	0	0	0
0	0	0	0	0	0	0	0
3	2	1	0	0	0	2	7
1	0	0	0	0	0	0	0
0	0	0	0	0	0	0	0
0	0	0	0	0	0	0	0
0	0	0	0	0	0	0	0
0	0	0	0	0	0	0	0
2	4	0	0	0	0	0	0
1	1	0	0	0	0	0	0
0	0	0	0	0	0	0	1
1	1	0	0	0	0	0	0
5	5	5	5	5	0	1	0
1	0	0	1	0	0	0	0
1	0	0	0	0	0	0	0
2	4	0	0	0	0	99	6
0	0	0	0	0	0	0	0
0	0	0	0	0	0	0	0
0	0	0	0	0	0	0	0
0	0	0	0	0	0	0	0
0	0	0	0	0	0	0	0
0	0	0	0	0	5	0	7
1	2	1	0	5	0	2	1
5	5	5	5	5	0	0	0
0	0	0	0	0	0	0	0
0	0	0	0	0	0	0	0
1	0	0	0	0	0	0	0
0	0	0	0	0	0	0	0
2	0	0	0	0	0	0	0
1	0	0	0	0	0	0	0
0	0	0	1	0	0	0	0
3	0	0	1	0	0	2	0
0	0	0	0	1	0	0	0
2	0	0	0	0	0	0	0
0	0	0	0	0	0	0	0
2	0	0	0	0	0	0	0
5	5	0	5	0	0	1	1
3	3	0	0	0	0	0	0
0	0	0	0	0	0	0	0
0	0	0	0	0	0	0	0
0	0	0	0	0	0	0	0
0	0	0	0	0	0	0	0
4	0	0	0	0	0	0	0

0	0	0	0	0	0	0	0
5	3	0	0	0	0	0	0
2	1	0	0	0	0	0	0
0	0	0	0	0	0	1	0
0	0	0	0	0	0	0	0
1	0	0	0	0	0	0	0
2	4	4	2	1	0	0	0
2	0	0	0	0	0	0	0
1	0	0	0	0	0	1	1
2	0	0	0	0	0	0	0
1	1	0	0	0	0	1	1
2	0	1	0	0	0	99	0
0	0	0	2	0	0	0	0
0	0	0	0	0	0	0	0
1	0	0	0	0	0	0	0
5	4	5	0	0	0	1	1
0	0	0	0	0	0	0	0
0	0	0	2	2	0	0	0
0	0	0	0	0	0	0	0
0	0	0	0	0	0	0	0
2	0	0	0	1	0	0	0
1	0	0	0	0	0	0	0
0	0	0	0	0	0	1	1
0	0	0	0	0	0	0	0
0	0	0	0	0	0	0	0
1	5	5	1	1	0	1	6
0	0	0	0	0	0	2	0
0	0	0	0	0	0	0	0
3	0	0	0	0	0	0	0
0	0	0	0	0	0	0	0
0	0	0	0	0	0	0	0
0	0	0	0	0	0	0	0
0	0	0	0	0	0	0	0
0	0	0	0	0	0	0	0
0	0	0	0	0	0	0	0
0	0	0	0	0	0	0	0
0	0	0	0	0	0	0	0
1	0	0	0	2	0	0	0
0	0	0	0	0	0	0	0
0	0	0	0	0	0	0	0
1	0	0	0	0	0	0	0
1	0	0	0	0	0	0	0
1	0	0	0	0	0	0	0
0	0	0	0	0	0	0	0
0	0	0	0	0	0	0	0
0	0	0	0	0	0	0	0
1	0	0	5	5	0	0	0
1	0	0	0	0	0	0	0

0	0	0	0	0	0	0	0
1	0	0	0	0	0	0	0
0	0	0	0	0	0	0	0
1	0	0	4	0	0	0	0
1	2	0	0	0	0	2	7
0	0	0	0	0	0	0	0
0	0	0	0	0	0	0	6
0	0	0	0	0	0	0	0
0	0	0	0	0	0	0	0
1	0	0	0	0	0	0	0
1	0	0	0	0	0	0	0
0	0	0	0	0	0	0	0
0	0	0	0	0	0	0	0
0	0	0	0	0	0	0	7
0	0	0	0	0	0	0	0
0	0	0	0	0	0	0	0
0	0	0	0	0	0	0	0
0	0	0	0	0	0	2	0
0	0	0	0	0	0	0	0
0	0	0	0	0	0	0	0
0	0	99	0	1	0	2	0
0	0	0	0	0	0	0	0
0	0	0	0	0	0	0	0
0	0	0	0	0	0	0	0
0	0	0	0	0	0	0	0
0	0	0	0	0	0	0	0
0	0	0	0	0	0	0	0
0	0	0	0	0	0	0	0
0	0	0	0	0	0	0	0
0	0	0	0	0	0	0	7
0	0	0	0	0	0	0	0
0	0	0	0	0	0	0	0
0	0	0	0	0	0	0	0
0	0	0	0	0	0	0	0
0	0	0	0	0	0	0	0
0	0	0	0	0	0	0	0
0	0	0	0	0	0	1	1
0	0	0	0	0	0	0	0
0	0	0	0	0	0	0	0
0	0	0	0	0	0	0	0
0	0	0	0	0	0	0	0
0	0	0	0	0	0	0	99
0	0	0	0	0	0	0	0
0	0	0	0	0	0	0	0
0	0	0	0	0	0	0	0
0	0	0	0	0	0	0	0
0	0	0	0	0	0	0	0
0	0	0	0	0	0	0	0

0	0	0	0	0	0	0	0
0	0	0	0	0	0	0	0
0	0	0	0	0	0	2	7
2	0	0	0	0	0	2	7
2	0	0	0	0	0	0	0
0	0	0	0	0	0	0	0
1	5	0	0	0	0	0	0
1	2	0	0	0	0	0	0
0	0	0	0	0	0	0	0
0	0	0	0	0	0	0	0
1	0	0	0	0	0	0	0
2	0	0	0	0	0	0	0
0	0	0	0	0	0	0	0
1	0	0	0	1	0	1	1
1	0	0	0	0	0	0	0
1	0	0	0	0	0	0	0
0	0	0	0	0	0	0	0
3	1	0	1	1	0	0	0
2	0	0	0	0	0	0	0
5	0	0	0	0	0	0	0
2	0	0	0	0	0	0	0
0	0	0	0	0	0	0	0
0	0	0	0	0	0	2	7
0	0	0	0	0	0	0	0
5	5	0	0	0	0	0	0
2	0	0	0	0	0	0	0
1	0	0	0	0	0	0	0
1	0	0	0	1	0	0	0
5	4	3	0	0	0	0	0
1	0	0	0	0	0	0	0
0	0	0	0	0	0	0	0
1	0	0	0	0	0	2	7
2	1	0	0	0	0	0	0
0	0	0	0	0	0	0	0
2	1	0	1	0	0	0	99
1	0	0	0	0	0	0	0
1	0	0	0	0	0	0	0
0	0	0	0	0	0	0	0
1	0	0	0	0	0	0	0
0	0	0	0	0	0	0	0
0	1	0	0	0	0	0	0
2	0	0	0	2	0	1	1
0	0	0	0	0	0	0	0
0	0	0	0	0	0	0	0
0	0	0	0	0	0	0	0
0	0	0	0	0	0	0	0
0	0	0	0	0	0	0	0
0	99	99	0	1	0	2	0

99	0	0	0	0	0	0	0
0	0	0	0	0	0	0	0
0	0	0	0	1	0	0	0
0	0	0	0	0	0	0	0
0	0	0	0	0	0	0	0
0	0	0	0	0	0	0	0
0	0	0	0	0	0	0	0
0	0	0	0	0	0	0	7
0	0	0	0	0	0	2	0
0	0	0	0	0	0	0	0
0	0	0	0	0	0	0	0
0	0	0	0	0	0	0	7
1	0	0	0	0	0	0	0
2	0	0	0	1	0	2	7
1	0	0	0	0	0	0	0
2	0	0	0	2	0	0	0
0	0	0	0	1	0	0	0
0	0	0	0	0	0	0	0
0	0	0	0	0	0	0	0
1	0	0	0	0	0	0	0
1	0	0	0	0	0	0	0
0	0	0	0	0	0	0	0
0	0	0	0	0	0	0	0
0	0	0	0	0	0	99	99
0	0	0	0	0	0	0	0
1	0	0	0	0	0	0	0
0	0	0	0	0	0	0	0
0	0	0	0	0	0	0	0
1	0	0	0	0	0	0	0
0	0	0	0	0	0	0	0
1	0	0	0	0	0	0	0
1	0	0	0	0	0	0	0
1	0	0	0	1	0	0	0
0	0	0	0	0	0	2	0
1	5	0	0	1	0	2	7
2	5	4	3	4	2	1	4
0	0	0	0	0	0	0	0
0	0	0	0	1	0	2	7
0	0	0	0	0	0	0	0
1	0	0	0	1	0	2	2
3	3	3	0	1	0	0	0
0	0	0	0	0	0	0	0
3	0	0	0	0	0	0	0
0	0	0	0	0	0	0	0
1	0	0	0	0	0	0	0
0	99	0	0	0	0	0	0
0	0	0	0	0	0	0	0
0	0	0	0	0	0	0	0

0	0	0	0	0	0	0	0
0	0	0	0	0	0	0	0
0	0	0	0	0	0	0	0
0	0	99	0	0	0	99	0
0	0	0	0	0	0	0	0
0	0	0	0	0	0	0	0
1	0	0	0	0	0	0	0
0	0	0	0	0	0	0	0
2	5	0	0	0	0	0	0
3	99	99	0	0	0	0	0
0	0	0	0	0	0	0	0
0	0	0	0	0	0	0	0
0	0	0	0	0	0	0	0
1	0	0	0	0	0	0	0
1	0	0	0	0	0	1	6
0	0	0	0	0	0	0	0
4	0	0	0	2	0	0	0
2	0	0	0	0	0	0	0
1	0	0	0	0	0	0	0
0	0	0	0	0	0	0	0
0	0	0	0	0	0	0	0
4	4	4	4	0	0	0	0
2	1	2	0	0	0	1	1
2	1	1	2	2	1	1	2
1	0	0	0	0	0	2	7
0	0	0	0	0	0	0	0
1	5	0	0	1	0	0	1
0	0	0	0	1	0	0	0
0	0	0	0	0	0	0	0
0	0	0	0	0	0	0	0
0	0	0	0	0	0	0	0
0	0	0	0	0	0	0	0
0	2	2	0	0	0	2	7
1	0	0	0	0	0	1	1
0	0	0	0	0	0	0	0
3	4	0	1	1	0	1	3
2	2	0	0	0	0	1	3
0	0	0	0	0	0	0	0
0	0	0	0	0	0	0	0
1	0	0	0	0	0	0	0
5	4	1	0	0	0	0	0
3	2	0	0	0	0	0	0
0	0	0	0	0	0	0	0
5	0	0	0	0	0	0	0
0	0	0	0	0	0	0	0
0	0	0	0	0	0	0	0
1	0	0	0	0	0	0	0
0	0	0	0	0	0	0	0

0	0	0	0	0	0	0	0
0	0	0	0	0	0	0	0
1	0	0	0	0	0	0	0
1	2	0	0	0	0	0	0
1	0	0	0	0	0	0	7
0	0	0	0	0	0	0	99
5	3	3	5	1	2	2	7
2	3	1	99	0	0	0	0
1	3	0	0	0	0	0	0
4	1	0	99	0	0	2	0
1	0	0	0	0	0	0	0
0	0	0	0	0	0	0	0
4	5	2	1	0	0	1	6
3	5	4	0	0	0	1	1
1	0	0	0	0	0	0	0
1	1	1	0	1	0	0	0
0	0	0	0	0	0	0	0
0	0	0	0	0	0	0	0
1	0	0	0	0	0	0	0
0	0	0	0	0	0	0	0
0	0	0	0	0	0	0	0
1	0	0	0	0	0	0	0
1	0	0	0	0	0	0	0
2	0	0	0	1	0	0	0
1	0	0	0	0	0	0	0
0	0	0	0	0	0	0	0
0	0	0	0	0	0	0	0
0	0	0	0	0	0	2	7
0	0	0	0	0	0	0	0
1	0	0	0	0	0	0	0
0	0	0	0	0	0	0	0
0	0	0	0	0	0	0	0
0	0	0	0	0	0	0	0
0	0	0	0	0	0	0	0
0	0	0	0	0	0	0	0
0	0	0	0	0	0	0	0
1	0	0	0	0	0	0	0
0	0	0	0	0	0	1	1
1	0	0	0	0	0	0	0
0	0	0	0	4	0	0	0
0	99	0	0	0	0	0	0
1	0	0	0	1	0	0	0
0	0	0	0	0	0	0	0
1	0	0	0	0	0	0	0
0	0	0	0	0	0	0	0
0	0	0	0	0	0	0	0
0	0	0	0	0	0	0	0
0	0	0	0	0	0	0	0

0	0	0	0	0	0	0	0
0	0	0	0	0	0	0	0
1	0	0	0	2	0	0	0
2	0	0	0	0	0	0	0
0	0	0	0	0	0	2	0
2	1	1	0	4	0	0	0
0	0	0	0	0	0	0	0
1	2	1	2	5	0	0	0
0	0	0	0	0	0	0	0
0	0	0	0	0	0	0	0
0	0	0	0	0	0	0	0
0	0	0	0	0	0	0	0
2	0	0	0	0	0	0	0
0	0	0	0	0	0	0	0
0	0	0	0	0	0	0	0
0	0	0	0	0	0	0	0
5	5	5	3	5	1	1	3
0	0	0	0	0	0	0	0
0	0	0	0	0	0	0	0
0	0	0	0	0	0	0	0
0	0	0	0	0	0	0	0
0	0	99	1	0	0	0	0
1	5	4	1	0	0	0	1
0	0	0	0	0	0	2	0
2	0	0	0	0	0	0	7
2	0	1	0	0	0	0	0
2	0	0	0	0	0	0	0
0	0	0	0	0	0	0	0
0	0	0	0	0	0	2	7
0	0	0	0	0	0	2	0
1	0	0	0	0	0	0	0
0	0	0	0	0	0	0	0
0	0	0	0	0	0	0	7
0	0	0	0	0	0	0	0
0	0	0	0	0	0	0	0
0	0	0	0	0	0	0	0
0	0	0	0	0	0	1	99
1	0	0	0	1	0	0	0
0	0	0	0	0	0	0	0
0	0	0	0	0	0	0	0
0	0	0	0	0	0	0	0
1	0	0	0	0	0	0	0
3	2	1	2	0	0	1	6
2	0	0	0	3	0	0	0
0	1	0	0	0	0	0	0
0	0	0	0	1	0	0	0
0	0	0	0	0	0	0	0
1	2	0	0	0	0	0	0

1	0	0	0	0	0	0	0
1	0	0	0	1	0	0	0
0	0	0	0	0	0	0	0
0	0	0	0	0	0	0	0
0	0	0	0	0	0	0	0
2	0	0	0	2	0	0	0
2	4	1	0	0	0	0	0
2	0	0	0	0	0	0	0
3	2	1	1	0	0	1	1
3	0	0	0	0	0	0	0
0	1	1	0	0	0	1	0
4	3	3	1	0	0	0	0
0	5	0	0	0	0	0	0
4	0	0	0	1	0	0	0
1	0	0	0	0	0	0	0
1	2	2	0	0	0	0	0
0	0	0	0	0	0	0	0
0	0	0	0	0	0	0	0
0	0	0	0	0	0	0	0
0	0	0	0	0	0	0	0
0	0	0	0	0	0	0	0
4	4	3	1	0	0	0	0
1	3	1	0	0	0	0	0
3	2	2	1	1	0	1	2
4	4	4	0	1	0	0	0
0	0	0	0	0	0	0	0
5	5	0	0	0	0	2	7
0	0	0	0	0	0	0	0
0	0	0	0	0	0	0	0
5	5	5	0	0	0	1	2
0	0	0	0	0	0	0	0
3	5	3	0	0	0	2	7
1	0	0	0	0	0	0	0
1	5	0	0	0	0	0	0
2	0	0	0	0	0	0	0
1	0	0	0	0	0	0	0
2	0	0	0	0	0	0	0
2	0	0	0	0	0	0	0
0	0	0	0	0	0	0	0
0	0	0	0	0	0	0	0
0	0	0	0	0	0	0	0
0	0	0	0	0	0	0	0
0	0	0	0	0	0	0	0
0	0	0	0	0	0	2	7
0	0	0	0	0	0	0	7
0	0	0	0	0	0	1	1
2	0	0	0	0	0	0	0
1	0	0	0	0	0	0	0

0	0	0	0	0	0	0	0
0	0	0	0	0	0	0	0
1	0	0	0	0	0	0	0
0	0	0	0	0	0	0	0
2	5	5	3	2	0	2	7
0	0	0	0	0	0	0	0
0	0	0	0	0	0	2	7
2	0	0	0	4	0	0	0
1	0	0	1	0	0	1	2
2	2	2	2	2	2	1	1
0	0	0	0	0	0	0	0
0	0	0	0	0	0	0	7
0	0	0	0	0	0	0	0
1	0	0	0	2	0	0	0
0	0	0	0	0	0	0	0
0	0	0	0	0	0	0	0
0	0	0	0	0	0	0	0
0	0	0	0	0	0	0	0
0	0	0	0	0	0	0	0
0	0	0	0	0	0	1	1
1	0	0	0	0	0	0	0
1	0	0	0	0	0	0	0
0	0	0	0	0	0	0	0
2	0	1	0	0	0	0	0
99	99	99	99	99	99	99	99
2	0	0	0	0	0	0	0
1	0	0	0	1	0	0	0
1	0	0	0	0	0	0	0
0	0	0	0	0	0	0	0
0	0	0	0	0	0	2	0
2	1	1	2	0	0	0	0
0	0	0	0	0	0	0	0
0	0	0	0	0	0	0	0
0	0	0	0	0	0	0	0
0	0	0	0	0	0	0	0
5	0	0	1	1	0	0	0
0	0	0	0	0	0	0	0
0	0	0	0	0	0	0	0
0	0	0	0	0	0	0	0
2	0	0	0	0	0	0	0
1	0	0	0	0	0	0	0
0	0	0	0	0	0	2	7
0	0	0	0	0	0	0	0
0	0	0	0	0	0	0	0
1	0	0	0	0	0	0	0
0	0	0	0	0	0	0	0
0	0	0	0	0	0	1	0
0	0	0	0	0	0	0	0

0	0	0	0	0	0	0	0
2	0	0	0	1	0	0	0
2	99	99	0	99	0	99	99
1	1	0	0	0	0	0	0
2	0	0	0	0	0	0	0
0	0	0	0	0	0	0	0
0	0	0	0	0	0	0	0
1	0	0	0	0	0	0	0
1	0	0	0	0	0	0	0
0	0	0	0	0	0	2	7
0	0	0	0	0	0	0	0
0	0	0	0	0	0	0	0
0	0	0	0	0	0	0	0
1	2	2	0	0	0	0	0
0	0	0	1	0	0	0	0
0	0	0	0	0	0	0	0
0	0	0	0	0	0	0	0
0	0	0	0	0	0	0	0
1	0	0	0	0	0	0	0
1	0	0	1	0	0	0	0
0	0	0	0	0	0	0	0
0	0	0	0	0	0	0	0
0	0	0	0	0	0	0	0
4	3	4	3	2	0	2	7
4	5	3	1	0	0	0	0
99	0	0	0	0	0	0	0
0	0	0	0	0	0	0	0
1	1	0	0	0	0	0	0
1	0	0	0	0	0	0	0
0	0	0	0	0	0	0	0
0	0	99	0	0	0	0	0
5	5	0	0	0	0	1	7
1	0	0	1	0	0	0	0
2	2	2	1	0	0	1	1
0	0	0	0	0	0	0	0
1	0	0	0	0	0	0	0
0	0	0	0	0	0	0	0
0	0	0	0	0	0	0	0
0	0	0	0	0	0	0	0
2	0	0	0	0	0	0	0
0	0	0	0	0	0	0	0
0	0	0	0	0	0	0	0
5	1	0	0	0	0	1	1
0	0	0	0	0	0	0	0
0	0	0	0	0	0	0	0
0	0	0	0	0	0	99	99
0	0	0	0	0	0	99	99
0	0	0	0	0	0	0	0

1	3	0	0	0	0	0	0
0	0	0	0	0	0	0	0
3	2	0	0	0	0	0	0
4	0	0	0	0	0	1	1
0	0	0	0	0	0	0	0
0	0	0	0	0	0	0	0
2	0	0	0	0	0	0	0
0	0	0	0	0	0	0	0
0	0	0	0	0	0	0	0
0	0	0	0	0	0	0	0
1	5	0	1	0	0	1	3
0	0	0	0	0	0	0	7
0	0	0	0	0	0	0	0
0	0	0	0	0	0	0	0
0	0	0	0	0	0	0	0
0	0	0	0	0	0	2	7
0	0	0	0	0	0	0	0
0	0	0	0	0	0	0	0
0	0	0	0	0	0	2	7
0	0	0	0	0	0	0	0
0	0	0	0	0	0	2	7
0	0	0	0	0	0	0	0
0	0	0	0	0	0	0	0
0	0	0	0	0	0	0	0
1	0	0	0	0	0	0	0
0	0	0	0	0	0	0	0
0	0	0	0	0	0	0	0
0	0	0	0	0	0	0	0
0	0	0	0	0	0	99	0
0	0	0	0	0	0	0	0
99	99	99	99	99	99	99	99
0	0	0	0	0	0	0	7
0	0	0	0	0	0	0	0
0	0	0	0	0	0	99	0
0	0	0	0	0	0	0	0
1	0	0	0	0	0	0	0
1	0	0	0	0	0	99	7
1	0	0	0	0	0	0	0
0	0	0	0	0	0	0	0
0	0	0	0	0	0	0	0
0	0	0	0	0	0	0	0
0	0	0	0	0	0	0	0
0	0	0	0	0	0	0	0
0	0	0	0	0	0	0	0
4	0	0	0	0	0	0	0
0	0	0	0	0	0	0	0
0	0	0	0	0	0	0	0
0	0	0	0	0	0	0	0

0	0	0	0	0	0	2	7
1	0	0	0	0	0	0	0
0	0	0	0	0	0	0	0
0	0	0	0	0	0	1	3
0	0	0	0	0	0	0	0
0	0	0	0	0	0	0	0
0	0	0	0	0	0	0	0
0	0	0	0	0	0	0	0
0	0	0	0	0	0	0	0
0	0	0	0	0	0	2	7
0	0	0	0	0	0	0	0
0	0	0	0	0	0	0	0
0	0	0	0	0	0	0	0
0	0	0	0	0	0	0	0
0	0	0	0	0	0	0	0
1	0	0	0	0	0	0	0
0	0	0	0	0	0	2	7
0	0	0	0	0	0	0	0
1	0	0	0	0	0	0	0
2	2	0	0	1	0	0	0
1	0	0	2	2	0	1	1
2	2	0	0	1	0	0	0
2	0	0	0	0	0	0	0
0	0	0	0	0	0	0	0
2	0	0	0	0	0	0	0
0	0	0	0	0	0	0	0
0	0	0	0	1	0	0	0
0	0	0	0	0	0	0	0
0	0	0	0	0	0	0	0
0	0	0	1	1	0	0	0
99	0	99	99	99	99	0	0
0	0	0	0	0	0	0	0
5	0	0	1	0	0	0	0
0	0	0	0	0	0	0	0
0	0	0	0	0	0	0	0
0	0	0	0	0	0	0	0
0	0	0	0	0	0	0	0
0	0	0	0	0	0	0	0
0	0	0	0	0	0	0	0
1	0	0	0	0	0	0	0
5	1	1	2	1	0	1	1
0	0	0	0	0	0	0	0
1	0	0	0	0	0	0	0
0	0	0	0	0	0	0	0
1	0	0	0	0	0	0	0
0	0	0	0	0	0	0	0
0	0	0	0	0	0	0	0
1	0	0	0	0	0	0	0

0	0	0	0	0	0	0	0
0	0	0	0	0	0	0	0
0	0	0	0	0	0	99	99
0	0	0	0	0	0	0	0
0	0	0	0	0	0	0	0
1	0	0	1	1	0	0	0
0	0	0	0	1	0	0	0
1	2	0	0	0	0	0	0
99	99	99	99	99	99	99	99
0	0	0	0	0	0	0	0
5	5	5	0	0	0	2	7
2	4	1	0	0	0	0	0
1	0	0	0	0	0	0	0
2	1	1	0	0	0	2	7
0	0	0	0	0	0	0	0
0	0	0	0	0	0	0	0
1	0	0	0	0	0	0	0
5	0	0	4	0	0	2	7
2	0	0	1	3	0	0	0
1	0	0	0	0	0	0	0
1	0	0	0	0	0	0	0
1	0	0	0	0	0	0	0
0	0	0	0	0	0	0	0
0	0	0	0	0	0	0	0
0	0	0	0	0	0	0	0
0	0	0	0	0	0	0	0
1	0	0	0	0	0	0	0
0	0	0	0	0	0	0	0
0	0	0	0	0	0	0	0
0	0	0	0	0	0	0	0
0	0	0	0	0	0	0	0
0	0	0	0	0	0	0	0
0	0	0	0	0	0	0	0
0	0	0	0	0	0	0	0
0	0	0	0	0	0	0	0
1	0	0	0	0	0	0	0
0	0	0	0	0	0	0	0
99	0	0	99	99	0	0	99
1	0	0	0	0	0	0	0
0	0	0	0	0	0	0	0
0	0	0	0	0	0	99	99
0	0	0	0	0	0	2	7
0	0	0	0	0	0	0	0
1	0	0	0	1	0	0	0
1	0	0	0	0	0	0	0
1	0	0	0	0	0	0	0
0	0	0	0	0	0	0	0
1	0	0	0	3	0	0	0

0	0	0	0	0	0	0	0
0	0	0	0	0	0	0	0
0	0	0	0	0	0	0	0
0	0	0	0	0	0	0	0
0	0	0	0	0	0	2	7
0	0	0	0	0	0	0	0
1	0	0	0	0	0	0	0
0	0	0	0	0	0	0	0
0	0	0	0	0	0	0	0
0	0	0	0	0	0	0	0
0	0	0	0	0	0	0	0
0	0	0	0	0	0	0	0
0	0	0	0	0	0	0	0
0	0	0	0	0	0	0	0
0	0	0	0	0	0	0	0
1	0	0	0	0	0	0	0
1	0	0	0	99	0	0	0
0	0	0	99	0	0	0	0
1	0	0	1	0	0	0	0
0	0	0	0	0	0	0	0
0	0	0	0	0	0	0	0
1	0	0	0	0	0	0	0
0	0	0	0	0	0	0	0
0	0	0	0	0	0	0	0
1	0	0	0	0	0	0	0
2	1	0	0	1	0	0	0
0	0	0	0	0	0	0	0
3	1	0	0	0	0	0	0
0	0	0	0	0	0	0	0
5	3	1	3	1	0	0	0
0	0	0	0	0	0	0	0
0	0	0	0	0	0	0	0
0	0	0	0	0	0	0	0
0	0	0	0	0	0	0	0
0	0	0	0	0	0	0	0
0	0	0	0	0	0	0	0
0	0	0	0	0	0	0	0
3	0	0	1	0	0	2	0
0	0	0	0	0	0	2	7
0	0	0	0	0	0	0	0
1	0	0	0	0	0	0	0
1	0	0	0	0	0	0	0
0	0	0	0	0	0	0	0
0	0	0	0	0	0	0	0
0	0	0	0	1	0	0	0
3	0	0	0	1	0	0	0
1	0	0	0	0	0	0	0
1	0	0	0	0	0	0	0

0	0	0	0	0	0	0	0
0	0	0	0	0	0	0	0
0	0	0	0	0	0	0	0
0	0	0	0	0	0	0	0
1	0	0	0	0	0	0	0
1	0	0	0	0	0	0	0
0	0	0	0	0	0	0	0
0	0	0	0	0	0	0	0
0	0	0	0	0	0	0	0
3	0	0	1	1	0	0	0
1	0	0	0	0	0	0	0
1	0	0	0	0	0	0	0
99	99	99	99	99	99	99	99
1	0	0	0	1	0	0	0
0	0	0	0	0	0	0	0
0	0	0	1	0	0	0	0
1	0	0	0	0	0	0	0
1	0	0	0	0	0	0	0
1	1	0	0	1	0	0	0
2	0	0	0	0	0	0	0
0	0	0	0	0	0	0	0
1	1	0	0	0	0	0	0
1	0	0	0	2	0	0	0
5	3	0	0	0	0	0	0
0	0	0	0	0	0	0	0
1	0	0	0	0	0	0	99
3	4	0	0	0	0	1	1
0	0	0	0	0	0	0	0
0	0	0	0	0	0	0	1
0	0	0	0	0	0	0	0
1	1	0	1	0	0	2	1
0	0	0	0	0	0	0	0
1	0	0	0	0	0	0	0
2	0	0	1	2	0	0	0
1	0	0	0	0	0	0	0
0	0	0	0	0	0	0	0
1	0	99	0	2	0	0	0
1	0	0	0	0	0	0	0
0	0	0	0	0	0	0	0
0	0	0	0	0	0	0	0
0	0	0	0	0	0	0	0
0	99	99	0	0	0	2	7
0	0	0	0	0	0	0	0
0	0	0	0	0	0	0	0
0	0	0	0	0	0	0	0
0	0	0	0	0	0	2	0
0	0	0	0	0	0	0	0
0	0	0	0	0	0	0	0

0	0	0	0	0	0	0	0
1	0	0	0	0	0	0	0
0	0	0	0	0	0	0	7
0	0	0	0	0	0	0	0
0	0	0	0	0	0	0	0
1	0	0	0	0	0	2	7
0	0	0	0	0	0	0	0
99	0	0	0	0	0	0	0
0	0	0	0	0	0	0	0
0	0	0	0	0	0	0	0
0	0	0	0	0	0	0	0
0	0	0	0	0	0	0	0
0	0	0	0	0	0	0	0
0	0	0	0	0	0	0	0
0	0	0	0	0	0	0	0
0	0	0	0	0	0	0	0
0	0	0	0	0	0	0	0
0	0	0	0	0	0	0	0
5	1	0	0	5	0	1	2
0	99	99	0	1	0	2	7
1	0	0	0	1	0	0	0
4	2	2	0	5	0	1	1
3	1	0	0	0	0	0	0
1	0	0	0	1	0	0	0
1	0	0	0	0	0	0	0
3	0	0	0	0	0	0	0
0	0	0	0	0	0	0	0
0	0	0	0	0	0	0	0
0	0	0	0	0	0	0	0
0	0	0	0	0	0	0	0
0	0	0	0	0	0	0	0
0	0	0	0	0	0	2	7
0	0	0	0	0	0	0	0
1	0	0	0	0	0	0	0
1	0	0	0	0	0	0	0
0	0	0	0	0	0	0	0
1	0	0	0	0	0	0	0
0	0	0	0	0	0	0	0
2	0	0	0	2	0	2	7
0	0	0	0	0	0	0	0
0	0	0	0	0	0	0	0
1	0	0	0	0	0	0	0
0	0	0	0	0	0	0	0
1	0	0	0	0	0	0	0
2	1	1	0	0	0	0	0
0	0	0	0	0	0	0	0
4	5	0	2	1	0	1	1
2	0	2	0	0	0	0	0

0	0	0	0	0	0	0	0
5	2	2	1	0	1	0	0
0	0	0	0	0	0	2	0
1	0	0	0	0	0	0	0
0	0	0	2	0	0	0	0
0	0	0	0	0	0	0	0
0	0	0	0	0	0	0	0
0	0	0	0	0	0	0	0
0	0	0	0	0	0	0	0
0	0	0	0	0	0	0	99
1	0	0	0	0	0	0	0
0	0	0	0	0	0	0	0
2	0	0	0	0	0	0	0
0	0	0	0	0	0	0	0
1	0	0	0	0	0	0	0
0	0	0	0	0	0	0	0
5	0	0	0	0	0	0	0
0	1	0	0	0	0	0	0
0	1	0	0	0	0	0	0
0	0	0	0	0	0	0	0
0	0	0	0	0	0	0	0
1	0	0	0	1	0	0	0
2	0	0	0	0	0	0	0
0	0	0	0	0	0	2	7
0	0	0	0	0	0	0	0
0	0	0	0	0	0	0	0
0	0	0	0	0	0	0	0
0	0	0	0	0	0	0	0
0	0	0	0	0	0	1	3
0	0	0	0	0	0	0	0
0	1	0	0	0	0	0	0
0	0	0	0	0	0	0	0
0	0	0	1	0	0	0	0
0	0	0	0	0	0	0	0
0	0	0	0	0	0	0	0
1	0	0	0	0	0	0	0
0	0	0	0	0	0	0	0
1	0	0	0	0	0	0	0
0	0	0	0	0	0	0	0
0	0	0	0	0	0	0	0
1	0	0	0	0	0	1	6
0	0	0	0	0	0	0	0
2	4	0	2	0	0	1	6
0	0	0	0	0	0	1	6
0	0	0	0	0	0	0	0
5	1	0	0	3	0	1	1
1	0	0	0	0	0	0	0
0	0	0	0	0	0	0	0

0	0	0	0	0	0	0	0
0	0	0	0	0	0	0	0
1	0	0	0	0	0	0	0
0	0	0	0	0	0	0	0
5	5	2	5	1	3	1	6
2	99	0	0	0	0	0	0
1	0	0	1	0	0	0	0
0	0	0	0	0	0	0	0
99	0	0	0	0	0	0	0
0	0	0	0	0	0	0	0
1	0	0	0	0	0	0	0
0	0	0	0	0	0	0	0
1	0	0	0	0	0	0	0
0	0	0	0	0	0	0	0
0	0	0	0	0	0	0	0
4	0	0	0	1	0	1	4
0	0	0	0	0	0	0	0
0	0	0	0	0	0	0	0
1	1	1	0	0	0	0	0
0	0	0	0	0	0	0	0
0	0	0	0	0	0	0	0
0	0	0	0	0	0	0	0
0	0	0	0	0	0	0	0
0	0	0	0	0	0	0	0
0	0	0	0	0	0	0	0
0	0	0	0	0	0	0	0
1	1	0	0	0	0	1	1
0	0	0	0	0	0	0	0
0	0	0	3	0	0	2	0
0	0	0	0	0	0	0	0
1	0	0	0	0	0	1	1
3	5	3	1	0	0	2	6
0	0	0	0	0	0	0	0
0	0	0	0	0	0	0	0
0	0	0	0	0	0	0	0
0	0	0	0	0	0	0	0
0	1	0	0	1	0	0	0
1	0	0	0	2	0	0	0
0	0	0	0	0	0	0	0
0	0	0	1	0	0	0	0
1	0	0	0	0	0	0	0
0	0	0	0	0	0	0	0
0	0	0	0	0	0	0	0
0	0	0	0	0	0	0	0
0	0	0	0	0	0	0	0
0	0	0	0	0	0	0	0
0	0	0	0	0	0	0	0
0	0	0	0	0	0	0	0

0	0	0	0	0	0	0	0
1	0	0	0	0	0	2	7
0	0	0	0	0	0	0	0
0	0	0	0	0	0	0	0
1	0	0	0	0	0	1	1
0	0	0	0	0	0	2	7
0	0	0	1	0	0	0	0
0	0	0	0	0	0	0	0
0	0	0	1	1	1	1	0
0	0	0	0	0	0	0	0
0	0	0	0	0	0	0	0
1	0	0	0	0	0	0	0
0	0	0	2	0	0	0	0
2	2	1	3	0	0	0	0
0	0	0	0	0	0	0	0
1	0	0	0	0	0	0	0
1	0	0	0	0	0	0	0
0	0	0	0	0	0	2	0
0	0	0	0	0	0	0	0
0	0	0	0	0	0	0	0
2	0	0	0	0	0	0	0
0	1	0	0	0	0	0	0
1	0	0	1	0	0	0	7
1	0	0	0	0	0	0	0
4	5	3	1	0	1	0	0
0	0	0	0	0	0	0	0
1	0	0	0	0	0	0	0
0	0	0	0	0	0	0	0
0	0	0	0	0	0	0	0
0	0	0	0	0	0	0	0
0	0	0	0	0	0	0	0
0	0	0	0	0	0	0	0
0	0	0	0	0	0	0	0
0	0	0	0	0	0	0	0
0	0	0	0	0	0	0	0
0	0	0	0	0	0	0	0
0	0	0	0	0	0	0	0
1	0	0	0	0	0	0	0
0	0	0	0	0	0	0	0
1	2	0	0	0	0	0	0
0	0	0	0	0	0	0	0
2	0	0	0	0	0	0	0
1	0	0	0	0	0	0	0
0	0	0	0	0	0	0	0
0	0	0	0	1	0	0	0
0	0	0	0	0	0	0	0
0	0	0	0	0	0	0	0
0	0	0	0	0	0	0	0

1	0	0	1	0	0	0	0
0	0	0	0	0	0	0	0
99	0	0	0	0	0	0	0
0	0	0	0	0	0	0	0
0	0	0	0	0	0	0	0
0	0	0	0	0	0	0	0
0	0	0	0	0	0	0	0
0	0	0	0	0	0	0	7
2	0	0	1	1	0	0	0
2	1	0	0	0	0	0	0
0	0	0	0	0	0	0	0
1	0	0	0	0	0	0	0
0	0	0	0	0	0	0	0
2	1	0	0	0	0	0	0
0	0	0	0	0	0	0	0
1	0	0	0	0	0	0	0
0	0	0	0	0	0	0	0
0	0	0	0	0	0	0	0
5	5	3	2	1	4	1	6
0	0	0	0	0	0	0	0
0	0	0	0	0	0	2	7
2	0	0	0	0	0	2	7
0	0	0	0	0	0	0	0
0	0	0	0	0	0	0	0
1	0	0	0	0	0	0	0
2	0	0	0	5	0	0	0
0	0	0	0	0	0	0	0
99	0	0	0	0	0	0	0
0	0	0	0	0	0	0	0
3	1	0	0	0	0	0	0
2	1	0	0	0	0	1	1
0	0	0	0	0	0	0	0
1	0	0	1	0	0	0	0
0	0	0	0	0	0	0	0
0	0	1	0	0	0	0	0
0	0	0	0	0	0	0	0
0	0	0	0	0	0	0	0
0	0	0	0	0	0	0	0
0	0	0	0	1	0	0	0
1	0	0	2	0	0	0	0
0	0	0	0	0	0	0	0
0	0	0	0	0	0	0	0
1	0	0	0	0	0	0	0
0	0	0	0	1	0	0	0
1	0	0	0	0	0	2	7
1	1	0	0	0	0	1	1
0	0	0	1	1	0	2	7
0	0	0	0	0	0	0	0

1	1	0	0	2	0	0	0
99	0	99	0	0	0	0	0
1	0	0	0	0	0	0	0
0	0	99	0	0	0	0	0
0	0	0	0	0	0	0	0
2	0	0	0	0	0	0	0
1	0	0	0	0	0	0	0
0	0	0	0	1	0	0	0
0	0	0	0	0	0	0	0
0	0	0	0	0	0	0	0
0	0	0	0	0	0	0	0
1	0	0	0	0	0	0	0
2	0	0	0	0	0	0	0
1	0	0	0	0	0	0	7
1	0	0	0	0	0	0	0
0	0	0	0	0	0	0	0
1	0	0	0	0	0	0	0
0	0	0	0	0	0	2	99
0	0	0	0	0	0	0	0
0	0	0	0	0	0	0	0
2	0	0	0	0	0	0	0
0	0	0	0	0	0	0	0
1	0	0	1	0	0	0	0
0	0	0	0	1	0	0	0
0	0	0	0	0	0	2	7
2	0	0	0	0	0	0	0
1	0	0	0	1	0	0	0
0	0	0	0	0	0	0	0
0	0	0	0	0	0	0	0
0	0	0	0	0	0	0	0
1	0	0	0	0	0	0	0
0	0	0	0	0	0	0	0
0	0	0	0	0	0	0	0
1	0	0	0	0	0	0	0
1	0	0	0	0	0	0	0
1	0	0	0	0	0	0	0
1	0	0	0	0	0	0	0
2	0	0	0	0	0	0	0
1	0	0	0	0	0	0	0
1	0	0	0	0	0	0	0
1	0	0	1	0	0	0	0
1	0	0	1	0	0	0	0
99	99	99	99	99	99	99	99
0	1	0	4	5	0	2	7
0	0	0	0	0	0	0	0
0	0	0	0	0	0	0	0
0	0	0	0	0	0	0	0
2	1	0	0	0	0	0	0

2	0	0	0	0	0	0	0
2	0	0	0	0	0	0	0
0	0	0	0	0	0	0	0
2	0	0	0	0	0	0	0
3	3	0	0	0	0	0	0
5	3	0	0	0	0	2	7
3	2	0	0	0	0	1	1
0	0	0	0	0	0	0	0
0	0	0	0	0	0	0	0
0	0	0	0	0	0	0	0
2	0	0	0	5	0	0	0
2	0	0	0	2	0	0	0
4	0	0	0	0	0	0	0
5	0	0	0	0	0	0	0
2	2	2	2	0	0	0	0
1	0	0	0	0	0	2	7
0	0	0	0	1	0	0	0
1	0	0	0	0	0	0	0
5	4	2	1	0	1	0	0
3	5	0	3	0	0	1	1
3	3	1	0	0	0	0	0
1	1	0	0	0	0	1	3
1	0	0	0	0	0	0	0
5	0	0	0	0	0	0	0
2	0	0	0	1	0	0	0
3	0	0	0	0	0	0	0
5	0	0	0	0	0	0	0
1	0	0	0	0	0	0	0
1	0	0	0	0	0	0	0
0	0	0	0	0	0	0	0
1	0	0	0	0	0	0	0
1	0	0	0	0	0	0	0
0	0	0	0	0	0	0	0
3	4	0	0	2	0	1	3
5	5	5	5	0	2	1	3
2	0	0	0	1	0	0	0
0	0	0	0	0	0	0	0
0	0	0	0	0	0	0	0
4	5	0	2	0	0	0	0
2	0	0	0	0	0	0	0
0	0	0	0	0	0	0	7
0	0	0	0	0	0	0	0
1	0	0	0	0	0	0	0
0	0	0	0	0	0	0	0
0	0	0	0	0	0	0	0
2	0	0	0	0	0	0	0
1	0	0	0	0	0	0	0
3	1	0	0	0	0	0	0

0	0	0	0	0	0	0	0
3	4	0	0	5	0	1	1
1	0	0	0	0	0	0	0
0	0	0	0	0	0	0	0
0	0	0	2	0	0	0	0
5	5	99	2	0	0	1	1
5	0	0	0	0	0	0	0
1	3	3	2	0	0	1	6
3	5	0	0	0	0	1	6
3	0	0	0	0	0	0	0
1	0	0	0	0	0	0	0
2	0	0	0	0	0	0	0
0	0	0	0	0	0	0	0
1	0	0	0	0	0	0	0
0	0	0	0	0	0	0	0
0	0	0	1	0	0	0	0
1	0	0	0	1	0	0	0
0	0	0	0	0	0	0	0
0	0	0	0	0	0	0	0
0	0	0	0	0	0	0	0
1	0	0	0	0	0	0	0
0	0	0	0	0	0	0	0
0	0	0	0	0	0	0	0
3	0	0	1	0	0	0	0
1	0	0	1	0	0	0	0
1	0	0	0	0	0	0	0
1	0	0	0	0	0	0	0
0	0	0	0	0	0	0	0
0	0	0	0	0	0	2	0
0	0	0	0	0	0	0	0
2	0	0	0	0	0	0	0
0	0	0	0	0	0	0	0
2	3	0	1	0	0	0	0
0	0	0	0	0	0	0	0
1	1	0	0	0	0	0	0
0	0	0	0	0	0	0	0
0	0	0	0	0	0	0	0
0	0	0	0	0	0	0	0
0	0	0	0	0	0	0	0
1	0	0	0	0	0	0	0
1	0	0	0	1	0	2	0
0	0	0	0	0	0	0	0
0	0	0	0	0	0	0	0
5	5	3	0	4	0	0	0
0	0	0	0	0	0	0	0
0	0	0	0	1	0	0	0
5	2	2	0	2	0	0	0
1	0	0	0	0	0	0	0

1	0	0	0	0	0	0	0
4	5	5	0	0	0	1	1
1	0	0	0	0	0	0	0
0	0	0	0	0	0	0	0
0	0	0	0	0	0	0	0
0	0	0	0	0	0	0	0
0	0	0	0	0	0	2	7
4	0	0	5	3	0	0	0
1	4	0	0	0	0	1	1
2	0	0	0	0	0	0	0
0	0	0	0	0	0	0	0
0	0	0	0	0	0	0	0
0	0	0	0	0	0	0	0
2	0	0	0	0	0	0	0
1	0	0	0	0	0	0	0
1	0	0	0	0	0	0	0
0	0	0	0	0	0	0	0
0	0	0	0	0	0	0	0
2	0	0	0	0	0	0	0
1	2	0	1	1	0	0	0
1	0	0	0	1	0	0	0
1	4	0	0	0	0	0	0
0	0	0	2	0	0	0	0
2	0	0	2	0	0	0	0
0	0	0	0	0	0	0	0
0	0	0	0	0	0	0	7
0	0	0	0	0	0	0	0
3	0	0	0	0	0	0	0
0	0	0	0	0	0	0	0
0	0	0	0	0	0	0	0
0	0	0	0	0	0	0	0
0	0	0	0	0	0	1	6
1	0	0	0	0	0	0	0
0	5	0	0	0	0	0	0
0	0	0	0	0	0	0	0
1	2	0	1	1	0	1	1
0	0	0	0	0	0	0	0
3	4	0	0	0	0	0	0
1	0	0	0	0	0	2	7
1	0	0	0	0	0	2	0
4	2	2	1	2	0	0	0
0	5	2	0	0	0	1	6
1	0	0	0	0	0	0	0
1	0	0	0	0	0	0	0
0	0	0	0	0	0	99	0
0	0	0	0	0	0	2	7
1	0	0	1	0	0	0	0
0	0	0	0	0	0	0	0

0	0	0	0	0	0	0	0
0	0	0	0	0	0	2	7
2	2	2	0	0	0	0	0
1	1	1	1	0	0	1	3
1	1	0	0	0	0	0	0
0	0	0	0	0	0	0	0
5	5	5	5	5	5	1	6
0	0	0	0	0	0	0	0
0	0	0	0	0	0	0	0
0	0	0	0	0	0	0	0
0	0	0	0	1	0	0	0
0	0	0	0	0	0	0	0
1	0	0	0	0	0	0	0
0	0	0	0	0	0	0	0
1	0	0	0	0	0	0	0
2	0	0	0	0	0	0	0
0	0	0	0	0	0	1	6
0	0	0	0	0	0	0	0
4	0	0	1	0	0	0	0
3	0	0	0	0	0	0	0
1	0	0	0	0	0	0	0
0	0	0	0	0	0	0	0
0	0	0	0	0	0	0	0
0	0	0	0	0	0	0	0
0	0	0	0	0	0	0	0
0	0	0	0	0	0	0	0
0	0	0	0	0	0	0	0
0	0	0	0	0	0	0	0
0	0	0	0	0	0	2	0
1	0	0	0	0	0	0	0
1	0	0	0	0	0	0	0
0	0	0	0	0	0	0	0
1	0	0	0	0	0	0	0
0	0	0	0	0	0	0	0
1	0	0	0	0	0	0	7
0	0	0	0	0	0	0	0
0	0	0	0	0	0	0	0
0	0	0	99	0	0	0	0
1	0	0	0	0	0	0	0
0	0	0	0	0	0	2	0
0	0	0	0	0	0	0	0
1	0	0	0	0	0	0	0
0	0	0	0	0	0	0	0
0	0	0	0	0	0	0	0
99	1	99	0	0	0	0	0
0	0	0	0	0	0	0	0
0	0	0	0	0	0	99	99
1	0	0	0	0	0	0	0

0	0	0	0	0	0	0	0
0	0	0	0	0	0	0	0
0	0	0	0	0	0	0	0
0	0	0	0	0	0	0	0
0	0	0	0	0	0	0	0
0	0	0	0	0	0	0	0
0	0	0	0	0	0	0	0
0	0	0	0	0	0	0	0
0	0	0	1	0	0	0	0
1	0	0	0	0	0	0	0
0	0	0	0	0	0	0	0
0	0	0	0	0	0	0	0
0	0	0	0	0	0	0	0
0	0	0	0	0	0	2	7
0	0	0	0	0	0	0	0
0	0	0	0	0	0	0	0
1	0	0	0	0	0	0	0
0	0	0	0	0	0	0	0
2	0	0	0	0	0	2	7
0	0	0	0	0	0	0	0
0	0	0	0	0	0	0	0
1	0	0	0	0	0	0	0
0	0	0	0	0	0	0	0
0	0	0	1	2	1	0	0
0	0	0	0	0	0	0	0
0	0	0	0	0	0	0	0
1	0	0	0	0	0	0	0
0	0	0	0	0	0	0	0
0	0	0	0	0	0	0	0
0	0	0	0	0	0	0	0
0	0	0	0	0	0	0	0
99	2	0	0	0	0	1	1
1	0	0	0	0	0	0	0
1	0	0	0	0	0	0	0
0	0	0	0	0	0	0	0
0	0	0	0	0	0	0	0
0	0	0	0	0	0	0	99
0	0	0	0	0	0	0	0
1	0	0	0	1	0	0	0
0	0	0	0	0	0	0	0
0	0	0	0	0	0	0	0
0	0	0	0	0	0	0	0
1	0	0	0	0	0	0	0
2	0	0	0	0	0	0	0
1	0	0	0	0	0	0	0
0	0	0	0	0	0	0	0
0	0	0	0	0	0	0	0
0	0	0	0	0	0	0	0

0	0	0	0	0	0	0	0
0	0	0	0	0	0	0	0
1	0	0	0	0	0	0	0
0	0	0	0	0	0	0	0
0	0	0	0	0	0	0	0
0	0	0	0	0	0	0	0
0	0	0	0	0	0	0	0
0	1	0	0	0	0	1	2
1	5	0	0	0	0	1	2
0	0	0	0	0	0	0	0
0	0	0	0	0	0	0	0
0	0	0	0	0	0	0	0
0	0	0	0	0	0	0	0
0	2	2	0	0	0	0	0
0	0	0	0	0	0	0	0
0	0	0	0	0	0	0	0
1	0	0	0	0	0	0	0
1	0	0	0	0	0	0	0
1	0	0	0	0	0	0	0
0	0	0	0	0	0	0	99
1	0	0	0	0	0	0	0
0	0	0	1	0	0	0	0
0	0	0	0	0	0	0	0
0	0	0	0	0	0	0	0
0	0	0	0	0	0	0	0
0	0	0	0	0	0	0	0
0	0	0	0	0	0	0	0
2	0	0	0	0	0	0	0
0	0	0	0	0	0	0	0
0	0	0	0	0	0	0	0
0	0	0	0	0	0	99	99
0	0	0	0	0	0	0	0
0	0	0	0	0	0	0	0
0	0	0	0	0	0	0	0
0	0	0	0	0	0	0	0
0	0	0	0	0	0	0	0
0	0	0	0	0	0	0	0
0	0	0	0	0	0	0	0
1	0	0	0	0	0	0	0
1	0	0	0	0	0	0	0
0	0	0	0	0	0	0	0
0	0	0	0	0	0	0	0
2	0	0	0	2	0	1	1
1	0	0	0	0	0	0	0
0	0	0	0	0	0	99	99
1	0	0	0	0	0	1	1
0	0	0	0	0	0	1	1
0	0	0	0	0	0	0	0

0	0	0	0	0	0	0	7
0	0	0	0	0	0	0	0
0	0	0	0	0	0	0	0
0	0	0	0	0	0	0	0
0	0	0	0	0	0	0	7
1	0	0	0	0	0	0	0
0	0	0	0	0	0	0	0
0	0	0	0	0	0	0	0
0	0	0	0	0	0	0	0
1	0	0	0	0	0	2	1
0	0	0	0	0	0	0	0
0	0	0	0	0	0	0	0
0	0	0	0	0	0	0	0
0	0	0	0	0	0	0	0
2	0	0	0	0	0	0	0
3	0	0	0	0	0	0	0
0	0	0	0	0	0	0	0
1	0	0	0	0	0	0	0
1	0	0	0	0	0	0	0
0	0	0	0	0	0	0	0
0	0	0	0	0	0	99	99
1	0	0	0	0	0	0	0
0	0	0	0	0	0	0	0
0	0	0	0	0	0	0	0
0	0	0	0	1	0	0	0
0	0	0	0	0	0	0	0
0	0	0	0	0	0	0	0
0	0	0	0	0	0	0	0
0	0	0	0	0	0	0	0
0	0	0	0	0	0	0	0
0	0	0	0	0	0	0	0
0	0	0	0	0	0	0	0
0	0	0	0	0	0	0	0
1	0	0	0	0	0	0	0
0	0	0	0	0	0	0	0
0	0	0	0	0	0	0	0
0	0	0	0	0	0	0	0
0	0	0	0	0	0	0	0
0	0	0	0	0	0	0	0
1	0	0	0	0	0	0	0
0	0	0	0	0	0	0	0
0	0	0	0	0	0	0	0
0	0	0	0	0	0	2	7
0	0	0	0	0	0	0	0
0	0	0	0	0	0	0	0
3	0	0	0	0	0	0	0
1	0	0	0	0	0	0	0
99	1	99	2	5	1	0	1

5	0	0	0	0	0	0	0
0	0	0	0	0	0	0	0
0	0	0	0	0	0	0	0
99	3	2	0	0	0	0	0
0	0	0	0	0	0	0	0
1	0	0	0	0	0	0	0
0	0	0	0	0	0	0	0
2	0	0	0	0	0	0	0
0	0	0	0	0	0	0	0
0	0	0	0	0	0	0	0
0	0	0	0	2	0	0	0
0	0	0	0	0	0	0	0
1	0	0	0	0	0	0	0
0	1	0	0	0	0	0	0
0	0	0	0	1	0	0	0
2	0	0	1	1	0	1	3
0	0	0	0	0	0	0	0
1	1	0	0	0	0	0	0
2	1	0	0	1	0	1	1
1	0	0	0	0	0	0	0
0	0	99	1	0	0	0	0
0	0	0	0	0	0	0	0
0	0	0	0	0	0	0	0
1	0	0	0	0	0	0	0
1	0	0	0	0	0	0	0
0	0	0	0	0	0	0	0
4	0	0	1	2	0	0	0
0	0	0	0	0	0	0	0
3	1	1	0	0	0	0	0
0	0	0	0	0	0	0	0
1	1	99	1	0	0	0	0
0	0	0	0	0	0	0	0
1	0	0	0	0	0	0	0
1	0	0	0	0	0	0	0
0	0	0	0	0	0	0	0
2	0	0	0	0	0	0	0
1	0	0	0	0	0	1	1
1	0	0	0	0	0	0	0
1	0	0	0	1	0	0	0
0	0	0	0	0	0	0	0
2	3	0	0	0	0	2	7
0	0	0	0	0	0	0	0
0	0	0	0	0	0	0	0
4	0	0	0	0	0	0	0
0	0	0	1	0	0	0	0
0	0	0	0	0	0	0	0
2	2	0	0	0	0	0	0
0	0	0	0	0	0	0	0

0	0	0	0	0	0	0	0
0	0	0	0	0	0	0	0
0	0	0	0	0	0	0	0
1	5	0	0	0	0	0	0
0	0	0	0	0	0	0	0
0	0	0	0	0	0	0	0
1	0	0	0	0	0	0	0
0	0	0	0	0	0	0	0
1	1	0	0	0	0	0	7
0	0	0	0	0	0	0	0
1	0	0	0	0	0	0	0
1	0	0	0	0	0	0	0
0	0	0	0	0	0	0	0
0	0	0	0	0	0	0	0
2	0	0	0	0	0	0	0
0	0	0	0	0	0	0	0
0	0	0	0	0	0	0	0
0	0	0	0	0	0	0	0
0	0	0	0	0	0	0	0
0	0	0	1	0	0	0	0
0	0	0	0	99	99	2	0
0	0	0	0	0	0	0	0
0	0	0	0	0	0	0	0
1	0	0	0	1	0	2	7
0	0	0	0	1	0	0	0
1	0	0	0	0	0	0	0
0	0	0	0	0	0	0	0
5	3	0	0	3	1	1	1
0	0	0	0	0	0	0	0
0	0	0	1	0	0	0	0
0	0	0	0	0	0	0	0
0	0	0	0	0	0	0	0
0	0	0	0	0	0	0	0
0	0	0	0	0	0	0	0
0	0	0	0	0	0	0	0
1	0	0	0	1	0	2	7
0	0	0	0	1	0	0	0
0	0	0	0	0	0	0	0
0	0	0	0	0	0	0	0
0	0	0	0	0	0	0	0
0	0	0	0	0	0	2	7
0	0	0	0	0	0	2	7
0	0	0	0	0	0	0	0
0	0	0	0	0	0	0	0
0	0	0	0	0	0	0	0
0	0	0	0	0	0	0	0
1	0	0	1	0	0	0	7
1	0	0	0	0	0	0	0

0	0	0	0	0	0	0	0
0	1	0	0	1	0	99	0
1	0	0	0	0	0	0	0
0	0	0	0	0	0	0	0
0	0	0	0	0	0	0	0
0	0	0	0	0	0	2	7
0	0	0	0	0	0	0	0
0	0	0	0	0	0	0	0
0	0	0	0	0	0	1	5
5	0	0	0	2	0	0	0
0	0	0	0	0	0	0	0
0	0	0	0	0	0	0	0
3	0	0	0	0	0	0	0
0	0	0	0	0	0	0	0
0	3	0	0	0	0	2	7
0	0	0	0	0	0	0	0
1	0	0	0	0	0	0	0
0	0	0	0	1	0	2	0
2	0	0	0	1	0	0	0
0	0	0	0	0	0	0	0
2	0	0	0	0	0	0	0
0	0	0	0	0	0	0	0
0	1	0	0	0	0	2	7
2	0	0	1	0	0	0	0
0	0	0	0	0	0	0	0
1	1	0	0	0	0	0	0
2	0	0	1	0	0	2	0
0	1	0	0	0	0	0	0
0	0	0	0	0	0	0	0
0	0	0	0	0	0	0	0
0	0	0	0	0	0	0	0
2	1	2	0	1	0	2	7
0	0	0	0	0	0	0	0
2	0	0	0	0	0	0	0
0	0	0	0	0	0	1	6
0	0	0	1	0	0	0	0
0	0	0	0	0	0	0	0
3	0	0	0	3	0	0	0
1	0	0	1	0	0	0	0
3	0	0	0	0	0	2	7
0	0	0	0	0	0	0	0
1	0	0	0	0	0	0	99
0	5	0	0	0	0	0	0
2	0	0	0	0	0	1	1
1	2	1	0	1	0	1	1
4	4	4	0	2	0	1	7
1	0	0	0	0	0	0	0
0	0	0	0	0	0	0	0

0	0	0	0	0	0	0	0
0	0	0	0	0	0	0	0
0	0	0	0	0	0	0	0
3	0	0	0	0	0	0	0
2	0	0	0	0	0	0	0
4	0	0	0	0	0	2	7
0	0	0	0	0	0	0	0
0	0	0	0	0	0	0	0
0	0	0	0	0	0	0	0
0	0	0	0	0	0	0	0
0	0	0	0	0	0	0	0
1	2	0	0	0	0	2	7
3	3	0	0	0	0	0	0
0	0	0	0	0	0	0	0
99	0	0	2	0	0	0	0
0	0	0	0	0	0	0	0
0	0	0	0	0	0	0	0
0	0	0	0	0	0	0	0
0	0	0	0	0	0	0	0
2	0	0	0	1	0	0	0
3	0	0	0	0	0	0	0
1	0	0	0	0	0	0	0
1	1	1	0	0	0	0	0
0	0	0	0	0	0	0	0
2	0	0	0	0	0	0	0
1	0	0	0	0	0	0	0
1	99	99	99	99	99	99	99
0	0	0	0	0	0	0	0
0	0	0	0	0	0	0	0
0	0	0	0	0	0	0	0
1	0	0	0	0	0	0	0
0	0	0	0	0	0	2	0
0	0	0	0	0	0	0	0
1	0	0	0	0	0	2	7
0	0	0	0	0	0	0	0
0	0	0	0	0	0	0	0
0	0	0	0	0	0	0	0
0	0	0	0	0	0	0	0
0	0	0	0	0	0	0	0
0	0	0	0	0	0	0	0
1	0	0	0	0	0	0	0
0	0	0	0	0	0	0	0
0	0	0	0	0	0	2	7
0	0	0	0	0	0	0	0
0	0	0	0	0	0	0	0
0	0	0	0	0	1	0	0
0	0	0	0	0	0	0	0
0	0	0	0	0	0	0	0

0	0	0	0	0	0	0	0
0	0	0	0	0	0	0	0
0	0	0	0	0	0	0	0
1	0	0	0	0	0	0	0
0	0	0	0	0	0	0	0
0	0	0	0	0	0	0	0
0	0	0	0	0	0	0	7
0	0	0	0	0	0	0	0
0	0	0	0	0	0	0	0
1	0	0	0	0	0	2	7
0	0	0	0	0	0	0	0
0	0	0	0	0	0	0	0
0	0	0	0	0	0	0	0
0	0	0	0	0	0	0	0
0	0	0	0	0	0	0	0
1	0	0	0	2	0	0	0
0	0	0	0	0	0	0	0
0	0	0	0	0	0	0	0
0	0	0	0	0	0	0	0
0	0	0	0	0	0	0	0
1	0	0	0	0	0	0	0
0	0	0	0	0	0	0	0
0	0	0	0	0	0	0	0
0	0	0	0	0	0	0	7
1	0	0	0	0	0	0	0
0	0	0	0	0	0	0	0
0	0	0	0	0	0	0	0
0	0	0	0	0	0	0	0
2	5	0	0	0	0	0	0
0	0	0	0	0	0	0	0
0	0	0	0	0	0	2	0
99	99	99	99	99	99	99	99
0	0	0	0	0	0	0	0
1	0	0	0	0	0	0	0
0	0	0	0	0	0	0	0
4	1	0	0	0	0	0	0
2	0	0	0	0	0	0	0
0	0	0	0	0	0	0	0
1	0	0	0	0	0	0	0
0	0	0	0	0	0	0	0
0	0	0	0	0	0	0	0
0	0	0	0	0	0	0	0
0	0	0	0	0	0	0	0
0	0	0	0	0	0	0	0
0	0	0	0	0	0	0	0
0	0	0	0	0	0	2	7
0	0	0	0	0	0	0	0
2	3	2	1	0	0	0	0

0	0	0	0	0	0	0	0
0	0	0	0	0	0	0	0
1	0	0	0	0	0	0	0
2	1	0	1	1	2	0	0
0	0	0	0	0	0	0	0
1	0	0	1	0	1	2	7
0	0	0	0	0	0	0	0
0	0	0	0	0	0	0	0
1	0	0	0	0	0	0	0
5	1	0	1	0	0	0	0
99	3	2	1	0	0	2	7
0	0	0	0	0	0	0	0
0	0	0	0	0	0	0	0
99	99	99	99	99	99	99	99
1	0	0	0	0	0	0	0
1	1	0	0	0	0	0	3
2	1	0	2	0	0	0	0
3	4	1	0	0	0	1	5
5	5	0	0	0	0	1	2
2	0	0	0	0	0	0	0
0	0	0	0	0	0	0	0
0	0	0	0	0	0	0	0
0	0	0	0	0	0	0	0
0	0	0	0	0	0	0	0
1	0	0	0	0	0	0	0
1	0	0	0	0	0	0	0
0	0	0	0	0	0	0	0
1	0	0	0	0	0	2	7
0	0	0	0	0	0	0	0
1	0	0	0	0	0	0	0
2	1	0	2	0	0	0	0
1	1	1	0	0	0	0	0
0	0	0	0	0	0	0	0
5	0	0	0	2	0	0	0
2	0	0	1	0	0	0	0
0	0	0	0	0	0	0	0
0	0	0	0	0	0	0	0
1	0	0	0	0	0	0	0
0	0	0	0	0	0	0	0
1	0	0	0	0	0	0	0
3	0	0	0	1	0	0	0
0	0	0	0	0	0	0	0
0	0	0	0	0	0	0	0
0	0	0	0	0	0	0	0
0	0	0	0	0	0	0	0
0	0	0	0	0	0	0	0
0	0	0	0	0	0	0	0
0	0	0	0	1	0	0	0

0	0	0	0	0	0	0	7
0	0	0	0	0	0	0	0
2	0	0	0	0	0	0	0
0	0	0	0	0	0	2	7
3	0	0	0	0	0	0	7
1	0	0	0	0	0	2	0
0	0	0	0	0	0	0	0
0	0	0	1	0	0	2	7
0	0	0	0	0	0	2	0
0	0	0	0	0	0	0	0
1	0	0	0	1	0	0	0
1	0	0	0	0	0	0	0
1	0	0	0	0	0	0	0
0	0	0	0	0	0	0	0
99	99	99	99	99	99	0	0
0	0	0	0	0	0	0	0
1	2	0	0	1	0	0	0
1	0	0	0	0	0	0	0
0	0	0	0	0	0	0	0
99	99	99	99	99	99	99	99
2	1	0	0	0	0	0	0
1	0	0	0	0	0	0	0
2	0	0	1	0	0	0	0
2	0	0	0	5	0	0	0
0	0	0	0	0	0	0	0
0	0	0	0	0	0	0	0
1	2	0	0	1	0	0	0
0	0	0	0	0	0	0	0
0	0	0	0	0	0	0	0
0	0	0	0	0	0	0	0
2	0	0	0	0	0	0	0
1	0	0	0	0	0	0	0
0	0	0	0	0	0	0	0
1	0	0	0	0	0	0	0
1	0	0	0	0	0	2	7
3	0	0	0	0	0	0	0
0	0	0	0	0	0	0	0
0	0	0	0	0	0	0	0
0	0	0	0	0	0	0	0
0	0	0	0	0	0	0	7
0	0	0	0	0	0	0	0
0	0	0	0	99	0	0	0
1	0	0	0	0	0	0	0
0	0	0	0	0	0	0	0
0	0	0	0	0	0	0	0
1	0	0	0	0	0	0	0
0	0	0	0	0	0	99	99
3	0	0	2	1	0	0	0

0	0	0	0	0	0	0	0
0	0	0	0	0	0	0	0
0	0	0	0	0	0	0	0
1	0	0	0	0	0	0	0
0	0	0	0	0	0	0	0
1	0	0	3	0	0	2	7
0	0	0	0	1	0	0	0
0	0	0	0	0	0	0	0
0	0	0	0	0	0	0	0
3	0	0	3	0	0	0	0
5	4	5	0	1	0	1	1
0	0	0	0	0	0	0	99
1	3	0	0	0	0	0	0
0	0	0	0	0	0	0	0
3	3	0	0	0	0	0	0
1	0	0	0	0	0	0	0
0	0	0	0	0	0	0	0
1	0	0	0	0	0	0	0
1	0	0	0	0	0	0	0
0	0	0	0	0	0	0	0
0	0	0	0	0	0	0	0
0	0	0	0	0	0	0	0
3	0	0	0	0	0	0	7
1	0	0	0	0	0	0	0
0	0	0	0	0	0	0	0
0	0	0	0	0	0	0	0
0	0	0	0	0	0	0	0
1	0	0	0	0	0	0	0
0	0	0	0	0	0	0	0
2	2	99	0	0	0	0	0
2	0	0	0	0	0	0	0
0	0	0	0	0	0	0	0
1	0	0	1	1	0	0	0
0	0	0	0	0	0	0	0
0	0	0	0	0	0	0	0
5	5	5	0	0	2	1	4
0	0	0	0	0	0	0	0
0	0	0	0	0	0	0	0
2	1	1	1	0	0	0	0
5	5	5	5	5	5	1	6
1	0	0	0	0	0	0	0
5	5	5	5	5	5	0	6
0	0	0	0	0	0	0	0
1	0	0	0	0	0	0	0
1	0	0	0	0	0	0	0
2	0	0	0	0	0	0	0
0	0	0	0	0	0	0	0
1	0	0	0	0	0	0	0

3	1	0	0	0	0	0	0
0	0	0	0	0	0	0	0
0	0	0	0	0	0	0	0
0	0	0	0	0	0	0	0
1	0	0	0	0	0	0	0
0	0	0	0	0	0	0	0
0	0	0	0	0	0	2	0
1	0	0	0	0	0	0	0
1	0	0	0	0	0	0	0
2	1	0	0	1	0	0	0
1	0	0	0	0	0	0	0
0	0	0	0	0	0	0	0
0	0	0	0	0	0	0	0
1	0	0	0	0	0	0	0
0	0	0	0	0	0	99	99
0	0	0	0	0	0	99	99
0	0	0	0	0	0	0	0
1	0	0	0	0	0	0	0
0	0	0	0	0	0	0	0
0	0	0	0	0	0	0	0
0	0	0	0	0	0	0	0
2	0	0	0	0	0	0	0
0	0	0	0	0	0	0	0
5	0	0	2	1	0	0	0
0	0	0	0	0	0	0	0
1	0	0	0	1	0	0	0
0	0	0	0	0	0	0	0
0	0	0	0	0	0	2	0
0	0	0	0	0	0	0	0
0	0	0	0	0	0	0	0
1	0	0	0	0	0	0	0
1	1	0	1	0	0	0	0
0	0	0	0	0	0	0	0
1	0	0	0	0	0	0	0
4	1	0	0	0	0	0	0
0	0	0	0	1	0	0	0
0	0	0	0	0	0	99	99
0	0	0	0	0	0	0	0
1	1	0	0	0	0	0	0
0	0	0	0	0	0	0	0
0	0	0	0	0	0	0	0
1	0	0	0	0	0	0	0
1	0	0	0	1	0	0	0
0	0	0	0	0	0	0	0
0	0	0	0	0	0	0	0
1	0	0	1	0	0	0	0
1	0	0	0	0	0	0	0
1	0	0	0	0	0	0	0

2	1	0	0	0	0	0	0
0	0	0	0	0	0	0	0
3	2	1	0	0	0	0	0
0	0	0	0	0	0	0	0
0	0	0	0	0	0	0	0
0	0	0	0	0	0	0	0
0	0	0	0	0	0	0	0
1	1	0	0	0	0	0	0
3	3	0	0	0	0	0	0
1	0	0	0	0	0	0	0
0	0	0	0	0	0	0	0
0	0	0	0	0	0	0	0
1	0	0	0	0	0	2	7
4	0	0	0	0	0	0	0
0	0	0	0	0	0	1	1
0	0	0	0	0	0	0	0
0	0	0	0	0	0	0	0
0	0	0	0	0	0	0	0
1	0	0	0	0	0	2	7
0	0	0	0	0	0	0	0
0	0	0	0	0	0	0	0
0	0	0	0	0	0	0	0
1	0	0	0	0	0	0	0
1	0	0	0	0	0	0	0
4	5	1	2	0	5	1	6
1	0	0	0	0	0	0	0
0	0	0	0	0	0	0	0
2	3	0	0	0	0	0	0
0	0	0	0	0	0	0	0
2	0	0	0	0	0	0	0
0	0	0	0	0	0	2	7
0	0	0	0	0	0	2	0
0	0	0	0	0	0	0	0
0	0	0	0	0	0	0	0
0	0	0	0	0	0	0	0
0	0	0	0	0	0	0	0
99	99	99	99	99	99	99	99
0	0	0	0	0	0	0	0
0	0	0	0	0	0	0	0
0	0	0	0	0	0	0	0
0	0	0	0	0	0	0	0
0	0	0	0	0	0	0	0
1	0	0	0	0	0	0	0
0	0	0	0	0	0	0	0
1	0	0	0	0	0	0	0
1	0	0	0	0	0	2	7
0	0	0	0	0	0	0	0
1	0	0	0	0	0	0	0

0	0	0	0	0	0	0	0
5	0	0	0	0	0	0	0
2	0	0	1	0	0	0	0
0	0	0	0	0	0	0	0
0	0	0	0	0	0	0	0
0	0	0	0	0	0	0	0
0	0	0	0	0	0	0	0
0	0	0	0	0	0	2	7
0	0	0	0	0	0	0	0
1	0	0	0	0	0	0	0
0	0	0	0	0	0	0	0
0	0	0	0	0	0	0	0
0	0	0	0	0	0	0	0
0	0	0	0	0	0	0	0
0	0	0	0	0	0	2	0
0	0	0	0	0	0	0	0
0	0	0	0	0	0	0	0
0	0	0	0	0	0	0	0
0	0	0	0	0	0	0	0
0	0	0	0	0	0	0	0
0	0	0	0	0	0	0	0
0	0	0	0	0	0	0	0
0	0	0	0	0	0	99	99
1	0	0	0	0	0	99	0
0	0	0	0	0	0	0	0
0	0	0	0	0	0	0	0
0	0	0	0	0	0	0	0
0	0	0	0	0	0	0	0
0	0	0	0	0	0	2	0
0	0	0	0	0	0	0	0
0	0	0	0	0	0	0	0
0	0	0	0	0	0	0	0
0	0	0	0	0	0	0	0
0	0	0	0	0	0	0	0
0	0	0	0	0	0	2	7
0	0	0	0	0	0	0	0
0	0	0	0	0	0	0	0
0	0	0	0	0	0	0	0
0	0	0	0	0	0	0	0
0	0	0	0	0	0	0	0
0	0	0	0	0	0	0	0
0	0	0	0	0	0	0	0
0	0	0	0	0	0	0	0
0	0	0	0	0	0	0	0
0	0	0	0	0	0	0	0
0	0	0	0	0	0	0	0
1	0	0	0	0	0	0	0
0	0	0	0	0	0	0	0

0	0	0	0	0	0	0	0
0	0	0	0	0	0	0	0
0	0	0	0	0	0	0	0
0	0	0	0	0	0	0	0
1	0	0	0	0	0	0	0
0	0	0	0	0	0	0	0
0	0	0	0	0	0	0	0
1	0	0	0	0	0	0	0
0	0	0	0	0	0	0	0
0	0	0	0	0	0	0	0
0	0	0	0	0	0	0	0
1	0	0	0	0	0	0	0
0	0	0	0	0	0	0	0
0	0	0	0	0	0	0	7
0	0	0	0	0	0	0	0
1	0	0	0	0	0	0	0
1	0	0	0	0	0	0	0
0	0	0	0	0	0	0	0
2	0	0	1	0	0	0	0
1	0	0	0	0	0	0	0
0	0	0	0	0	0	0	0
0	0	0	0	0	0	0	0
0	0	0	1	0	0	0	0
0	0	0	1	0	0	0	0
0	0	0	0	0	0	0	0
0	0	0	0	0	0	0	0
0	0	0	0	0	0	2	0
0	0	0	99	0	0	0	0
1	0	0	0	0	0	0	0
0	0	0	0	0	0	0	0
1	0	0	0	0	0	0	0
2	0	0	0	0	0	0	0
0	0	0	0	0	0	0	0
0	0	0	0	0	0	0	0
0	0	0	0	0	0	0	0
0	0	0	0	0	0	0	0
99	99	99	99	99	99	99	99
0	0	0	0	0	0	0	0
0	0	0	0	0	0	0	0
0	0	0	0	0	0	0	0
1	0	0	1	0	0	0	0
0	0	0	0	0	0	0	0
99	0	0	0	0	0	0	0
0	0	0	0	0	0	2	7
0	0	0	0	0	0	0	0
0	0	0	0	0	0	0	0
0	0	0	1	0	0	2	0
0	0	0	0	0	0	0	0

0	0	0	0	0	0	0	0
0	0	0	0	0	0	0	0
0	0	0	0	0	0	0	0
0	0	0	0	0	0	0	0
0	0	0	0	0	0	2	2
0	0	0	0	0	0	0	0
0	0	0	0	0	0	2	7
0	0	0	0	0	0	0	0
0	0	0	0	0	0	0	0
1	0	0	0	0	0	2	0
0	0	0	0	0	0	0	0
0	0	0	0	0	0	0	0
0	0	0	0	0	0	0	0
0	0	0	0	0	0	0	0
0	0	0	0	0	0	0	0
0	0	0	0	0	0	0	0
0	0	0	0	0	0	0	0
0	0	0	0	0	0	0	0
0	0	0	0	0	0	0	0
0	0	0	0	0	0	0	0
0	0	0	0	0	0	0	0
0	0	0	0	0	0	0	0
0	0	0	0	0	0	0	0
0	0	0	0	0	0	0	0
2	0	0	0	1	0	0	0
0	0	0	0	0	0	0	0
0	0	0	0	0	0	0	0
0	0	0	0	0	0	0	0
0	0	0	0	0	0	0	0
0	0	0	0	0	0	0	99
0	0	0	0	0	0	0	0
0	0	0	0	0	0	0	0
0	0	0	0	0	0	0	0
0	0	0	0	0	0	0	0
0	0	0	0	0	0	0	0
99	0	0	1	0	0	0	0
0	0	0	0	0	0	0	0
99	99	99	99	99	99	99	99
1	0	0	0	1	0	0	0
0	0	0	0	0	0	0	0
0	0	0	0	0	0	0	0
0	0	0	0	0	0	0	0
0	0	0	0	0	0	0	0
0	0	0	0	0	0	0	0
1	0	0	0	0	0	0	0
0	0	0	0	0	0	0	0
0	0	0	0	1	0	0	0
0	0	0	0	0	0	0	0

0	0	0	0	0	0	99	99
1	0	0	0	0	0	0	99
0	0	0	0	0	0	0	0
0	0	0	0	0	0	0	0
1	0	0	0	0	0	0	0
0	0	0	1	0	0	0	0
0	0	0	0	0	0	0	0
0	0	0	0	0	0	0	0
1	0	0	0	0	0	0	0
1	0	0	0	0	0	0	0
1	0	0	0	0	0	0	0
1	0	0	0	0	0	0	0
0	0	0	0	0	0	0	0
1	0	0	1	0	0	0	0
1	0	0	0	0	0	0	0
0	0	0	0	0	0	0	0
0	0	0	0	0	0	0	0
0	0	0	0	0	0	0	0
0	0	0	0	0	0	0	0
0	0	0	0	0	0	2	7
0	0	0	0	0	0	0	0
1	0	0	0	0	0	2	7
99	99	99	99	99	99	2	99
0	0	0	0	0	0	0	0
0	0	0	0	0	0	0	0
0	0	0	0	0	0	0	0
0	0	0	0	0	0	0	0
0	0	0	0	0	0	0	0
1	0	0	1	0	0	0	0
0	0	0	0	0	0	0	0
0	0	0	1	0	0	0	0
0	0	0	0	0	0	0	0
0	0	0	0	0	0	0	0
0	0	0	0	0	0	0	0
0	0	0	0	0	0	0	0
0	0	0	0	0	0	0	0
0	0	0	0	0	0	0	0
0	0	0	0	0	0	0	0
0	0	0	0	0	0	0	0
1	0	0	0	0	0	0	0
0	0	0	0	0	0	0	0
0	0	0	0	0	0	0	0
0	0	0	0	0	0	0	0
0	0	0	0	0	0	0	0
0	0	0	1	0	0	0	0
0	0	0	0	0	0	0	0
0	0	0	0	0	0	0	0
0	0	0	0	0	0	0	0

0	0	0	0	0	0	2	7
1	5	0	0	3	0	1	4
0	0	0	0	0	0	0	0
0	0	0	0	0	0	2	7
0	0	0	0	0	0	0	0
0	0	0	0	0	0	0	0
0	0	0	0	0	0	0	0
0	0	0	0	0	0	0	0
1	0	0	0	0	0	0	0
0	0	0	0	0	0	0	0
0	0	0	0	0	0	0	0
0	0	0	0	0	0	0	99
0	0	0	0	0	0	0	0
0	0	0	0	0	0	0	0
0	0	0	0	0	0	0	0
0	0	0	0	0	0	0	0
0	0	0	0	0	0	0	0
0	0	0	0	0	0	0	0
0	0	0	0	0	0	0	0
0	0	0	0	0	0	0	0
0	0	0	0	0	0	0	0
0	0	0	0	0	0	0	0
0	0	0	0	0	0	0	0
0	0	0	0	0	0	0	0
0	0	0	0	0	0	99	99
0	0	0	0	0	0	0	0
1	0	0	0	0	0	0	0
0	0	0	0	0	0	0	0
99	99	99	99	99	99	0	0
1	0	0	0	0	0	0	0
0	0	0	0	0	0	0	0
1	0	0	0	0	0	0	0
0	0	0	0	0	0	0	0
0	0	0	0	0	0	0	0
0	0	0	0	0	0	0	0
0	0	0	0	0	0	0	0
0	0	0	0	0	0	0	0
0	0	0	0	0	0	0	0
0	0	0	0	0	0	2	7
0	0	0	0	0	0	0	0
0	0	0	0	0	0	0	0
1	0	0	0	0	0	0	0
0	0	0	0	0	0	0	0
1	0	0	0	0	0	0	0
0	0	0	0	0	0	0	0
1	0	0	0	0	0	0	0
0	0	0	0	0	0	0	0
1	1	0	0	0	0	0	0

2	0	0	3	4	0	0	0
1	1	0	0	0	0	0	99
0	0	0	0	0	0	0	0
0	0	0	0	0	0	0	0
1	0	0	0	0	0	0	0
0	0	0	0	0	0	0	0
0	0	0	0	0	0	0	0
0	0	0	0	0	0	0	0
0	0	0	0	0	0	0	0
0	0	0	0	0	0	0	0
2	0	0	1	0	0	0	0
1	0	0	0	0	0	0	0
0	0	0	0	0	0	0	0
0	2	0	0	0	0	0	0
3	3	3	3	0	0	0	0
2	0	0	2	0	0	0	0
0	0	0	0	0	0	0	0
1	0	0	0	0	0	0	0
1	0	0	0	0	0	0	0
0	0	0	0	1	0	0	0
3	2	3	2	3	1	1	1
0	0	0	0	0	0	0	0
0	0	0	0	0	0	0	0
0	1	0	0	0	0	0	0
0	0	0	0	0	0	0	0
99	99	99	99	99	99	99	99
0	0	0	0	0	0	0	0
0	0	0	0	0	0	0	0
0	0	0	0	0	0	0	0
0	0	99	0	0	0	2	7
1	0	0	0	0	0	0	0
0	0	0	0	0	0	0	0
0	0	0	0	0	0	0	0
0	0	0	0	0	0	0	0
0	0	0	0	0	0	2	7
0	0	0	0	0	0	0	0
1	0	0	0	0	0	0	0
0	0	0	0	0	0	0	0
1	0	0	0	0	0	2	7
0	0	0	0	0	0	0	0
2	0	0	0	0	0	0	0
0	0	0	0	0	0	0	0
0	0	0	0	0	0	0	0
0	0	0	0	0	0	0	0
0	0	0	0	0	0	0	0
0	0	0	0	0	0	0	0
0	0	0	0	0	0	0	0
0	0	0	0	0	0	0	0

0	0	0	0	0	0	0	0
0	0	0	0	0	0	0	0
0	0	0	0	0	0	0	0
0	0	0	0	0	0	0	0
0	0	0	0	0	0	0	0
0	0	0	0	0	0	0	0
0	0	0	0	0	0	0	0
0	0	0	1	0	0	0	0
0	0	0	0	0	0	0	0
1	0	0	0	0	0	0	0
1	0	0	1	1	0	0	0
0	0	0	0	0	0	0	0
0	0	0	0	0	0	0	0
2	1	0	0	0	0	0	0
0	0	0	0	0	0	0	0
0	0	0	0	0	0	0	0
0	0	0	0	0	0	0	0
0	0	0	0	0	0	0	0
1	0	0	0	0	0	0	0
0	0	0	0	0	0	0	0
0	3	0	99	99	0	0	0
1	0	0	0	1	0	0	0
1	1	0	0	0	0	0	0
1	0	0	0	0	0	0	0
0	0	0	0	0	0	0	0
0	0	0	0	0	0	0	0
0	0	0	0	0	0	0	0
5	1	0	0	1	0	1	1
0	0	0	0	0	0	0	0
0	0	0	0	0	0	0	0
0	0	0	0	0	0	0	0
0	0	0	0	0	0	0	0
0	0	0	0	0	0	0	0
1	0	0	0	0	0	0	0
0	0	0	0	0	0	0	0
0	0	0	0	0	0	0	0
1	0	0	0	0	0	2	7
1	1	0	0	0	0	0	0
99	0	0	0	0	0	0	0
0	0	0	0	0	0	0	0
0	0	0	0	0	0	0	0
2	0	0	0	0	0	0	0
0	0	0	0	0	0	0	0
1	0	0	0	0	0	0	0
1	2	1	0	0	0	0	0
3	1	0	0	0	0	1	1
0	0	0	0	0	0	0	0
99	99	99	1	99	99	0	0

1	0	0	0	0	0	0	0
0	0	0	0	0	0	0	0
0	0	0	0	0	0	0	0
0	0	0	0	0	0	0	0
0	0	0	0	0	0	0	0
0	0	0	0	0	0	0	0
0	0	0	0	0	0	0	0
1	0	0	1	0	0	0	0
0	0	0	0	0	0	0	0
1	1	0	0	0	0	0	0
0	0	0	0	0	0	0	0
1	0	0	0	0	0	2	7
0	0	0	0	0	0	0	0
0	0	0	0	0	0	0	0
0	0	0	0	0	0	0	0
1	0	0	0	0	0	0	0
0	0	0	0	0	0	0	0
2	0	0	0	0	0	2	7
99	99	99	99	99	99	99	99
1	99	99	99	99	99	0	0
1	0	0	0	1	0	0	0
2	0	0	0	1	0	0	0
3	0	0	0	0	0	0	0
1	0	0	0	0	0	0	0
0	0	0	0	0	0	0	7
0	0	0	0	0	0	0	0
0	0	0	2	1	0	0	0
0	0	0	0	0	0	2	7
2	0	0	0	0	0	0	0
0	0	0	0	0	0	2	7
0	0	0	0	0	0	2	99
0	0	0	0	0	0	0	0
0	0	0	0	0	0	0	0
0	0	0	0	0	0	0	0
0	0	0	0	0	0	0	0
1	0	0	0	0	0	0	0
0	0	0	0	0	0	0	0
0	0	0	0	0	0	0	0
0	0	0	0	0	0	0	0
0	0	0	0	0	0	0	0
0	0	0	0	0	0	0	0
0	0	0	0	0	0	0	0
0	0	0	0	0	0	0	0
0	0	0	0	0	0	0	0
0	0	0	0	0	0	0	0
0	0	0	0	0	0	0	0
0	0	0	0	0	0	0	0
3	0	0	1	3	0	1	1

0	0	0	0	0	0	0	0
0	0	0	0	0	0	0	99
0	0	0	0	0	0	0	0
1	1	0	0	0	0	0	0
1	0	0	0	0	0	0	0
0	0	0	0	0	0	0	0
2	0	0	0	0	0	0	0
0	0	0	0	0	0	0	0
1	0	0	0	0	0	0	0
0	0	0	0	0	0	0	0
0	0	0	0	0	0	0	0
0	0	0	0	0	0	0	0
0	0	0	0	0	0	0	0
0	0	0	0	0	0	0	0
0	0	0	0	0	0	1	1
1	0	0	1	0	0	0	0
0	0	0	0	0	0	0	0
99	0	0	0	0	0	0	0
0	0	0	0	0	0	0	0
1	1	1	0	0	0	0	0
0	0	0	0	2	0	0	0
1	0	0	0	0	0	0	0
0	0	0	0	0	0	2	7
2	4	4	0	0	5	1	0
0	0	0	0	0	0	0	0
3	0	0	0	0	0	2	7
0	0	0	0	0	0	0	0
1	1	0	0	0	0	0	0
0	99	0	0	0	0	0	0
2	2	1	0	0	0	0	0
0	5	0	0	0	0	0	0
1	0	0	0	0	0	0	0
1	0	0	1	0	0	0	0
1	0	0	0	0	0	0	0
0	0	0	0	0	0	0	0
1	0	0	0	0	0	0	0
0	0	0	0	0	0	0	0
2	0	0	0	0	0	0	0
1	0	0	0	0	0	0	0
0	0	0	0	0	0	0	0
0	0	0	0	0	0	2	7
3	4	1	1	0	0	2	7
5	5	2	0	0	0	2	7
1	0	0	0	0	0	0	0
0	2	0	0	0	0	0	0
0	0	0	0	0	0	0	0
1	1	0	5	0	0	0	0
0	0	0	0	1	0	0	0

0	0	0	0	0	0	0	0
1	0	0	0	0	0	0	0
0	0	0	0	0	0	0	0
0	0	0	0	0	0	0	0
0	0	0	0	0	0	0	0
1	0	0	0	0	0	0	0
0	0	0	0	0	0	0	0
0	0	0	0	0	0	0	0
0	0	0	0	0	0	0	0
0	0	0	0	0	0	0	0
1	0	0	0	0	0	2	7
1	0	0	0	2	0	0	0
2	0	0	1	0	0	0	0
0	0	0	0	0	0	0	0
1	0	0	0	0	0	0	0
0	0	0	0	0	0	99	99
0	0	0	0	0	0	0	0
1	0	0	1	0	0	99	0
0	0	0	0	0	0	99	99
1	0	0	0	0	0	0	0
0	0	0	0	0	0	0	0
0	0	0	0	1	0	0	0
0	0	0	0	0	0	0	0
1	3	99	99	99	99	0	99
1	0	0	0	0	0	0	0
0	0	0	0	0	0	0	0
0	0	0	0	0	0	0	0
0	0	0	0	0	0	0	0
0	0	0	0	0	0	0	0
0	0	0	0	0	0	0	0
0	0	0	0	1	0	0	0
1	0	0	0	0	0	0	0
1	0	0	0	0	0	0	0
2	0	0	0	0	0	0	0
0	0	0	0	0	0	0	0
0	0	0	0	0	0	0	0
0	0	0	0	0	0	0	0
0	0	0	0	0	0	0	0
1	0	0	0	0	0	0	0
0	0	0	0	0	0	0	0
0	0	0	0	0	0	0	0
0	0	0	0	0	0	0	0
0	0	0	0	0	0	0	0
2	0	0	0	0	0	2	0
0	0	0	0	0	0	0	0
2	0	0	0	0	0	0	0
0	0	0	0	0	0	0	0
4	0	0	0	0	0	0	0

0	0	0	0	0	0	0	0
0	0	0	0	0	0	0	0
0	0	0	0	0	0	0	0
0	0	0	0	0	99	2	7
0	0	0	0	0	0	0	0
0	0	0	0	0	0	0	0
0	0	0	0	0	0	0	0
1	0	0	0	0	0	0	0
0	0	0	0	0	0	0	0
0	0	0	0	0	0	0	0
2	0	0	0	0	0	0	0
0	0	0	0	0	0	0	0
0	0	0	3	0	0	0	0
0	0	0	0	0	0	2	7
1	0	0	1	0	0	1	1
0	0	0	0	0	0	0	0
0	0	0	0	0	0	0	0
0	0	0	0	0	0	0	0
2	0	0	0	0	0	0	0
0	0	0	0	0	0	0	0
0	0	0	0	0	0	1	0
0	0	0	0	0	0	0	0
1	0	0	0	0	0	0	0
1	1	0	0	0	0	0	0
0	0	0	0	0	0	0	0
0	0	0	0	0	0	0	0
0	0	0	0	0	0	1	1
0	0	0	0	0	0	0	0
1	0	0	0	0	0	0	0
1	99	99	0	0	0	0	0
0	0	0	0	0	0	0	0
0	0	0	0	0	0	0	0
2	0	0	0	0	0	0	0
0	0	0	0	0	0	0	0
0	0	0	0	0	0	0	0
0	0	0	0	0	0	0	0
0	0	0	0	0	0	0	0
0	0	0	0	0	0	0	0
0	0	0	0	0	0	0	0
2	5	0	0	0	0	2	4
0	0	0	0	0	0	0	0
0	0	0	0	0	0	0	0
0	0	0	0	0	0	0	0
1	0	0	0	0	0	0	0
0	0	0	0	0	0	0	0
0	0	0	0	0	0	0	0
0	0	0	0	0	0	0	0
1	0	0	0	0	0	0	7

0	0	0	0	0	0	0	0
1	0	1	0	0	0	0	1
0	0	0	0	0	0	0	0
0	0	0	0	0	0	0	0
0	0	0	0	0	0	0	0
1	0	0	0	0	0	0	0
0	0	0	0	0	0	0	0
0	0	0	0	0	0	0	0
0	0	0	0	0	0	0	0
0	0	0	0	0	0	0	0
1	0	0	0	0	0	2	7
1	2	3	0	4	99	99	0
0	0	0	0	0	0	0	0
0	0	0	0	0	0	0	0
0	0	0	0	0	0	0	0
1	0	0	0	2	0	0	0
0	0	0	0	0	0	0	0
1	0	0	0	0	0	0	0
0	0	0	0	0	0	0	0
0	0	0	0	0	0	0	0
0	0	0	0	0	0	0	0
0	0	0	0	0	0	0	0
0	0	0	0	0	0	0	0
0	0	0	0	0	0	0	0
0	0	0	0	0	0	0	0
0	0	0	0	0	0	0	0
0	0	0	0	0	0	0	0
0	0	0	0	0	0	0	0
0	0	0	0	0	0	0	0
0	0	0	0	0	0	0	0
1	0	0	0	0	0	0	0
0	0	0	0	0	0	0	0
1	0	0	1	0	0	0	0
0	0	0	0	0	0	0	0
0	0	0	0	0	0	0	0
4	0	0	0	0	0	2	7
0	0	0	0	0	0	0	0
0	0	0	0	0	0	0	0
0	0	0	0	0	0	0	0
1	0	0	0	0	0	0	0
0	0	0	0	0	0	0	0
1	0	0	0	0	0	0	0
3	4	0	1	0	0	0	0
0	0	0	1	2	0	0	0
2	1	0	0	0	0	0	0
0	1	0	0	0	0	0	0
0	0	0	0	0	0	0	99
0	0	0	0	0	0	0	0

0	0	0	0	0	0	0	0
3	5	0	0	0	0	0	0
0	0	0	0	0	0	0	0
0	0	0	0	0	0	0	0
0	0	0	0	0	0	0	0
1	0	0	0	0	0	0	0
2	0	0	1	0	0	0	0
0	0	0	0	0	0	0	0
0	0	0	0	0	0	0	0
0	0	0	0	0	0	0	0
1	0	0	0	0	0	0	0
1	0	0	0	0	0	0	0
1	0	0	0	0	0	0	0
1	0	0	0	0	0	0	0
0	0	0	0	0	0	0	0
0	0	0	0	0	0	0	0
0	0	0	0	0	0	0	0
1	1	0	0	0	0	0	0
0	0	0	0	0	0	0	0
0	0	0	0	0	0	0	0
0	1	0	0	0	0	0	7
0	0	0	0	0	0	0	0
1	0	0	0	0	0	0	0
0	0	0	0	0	0	0	0
0	0	0	0	0	0	0	0
0	0	0	0	0	0	0	0
0	0	0	0	0	0	0	0
0	0	0	0	0	0	0	0
0	0	0	0	0	0	2	7
0	0	0	0	0	0	0	0
1	0	0	0	0	0	0	0
1	0	0	0	0	0	0	0
0	0	0	0	0	0	0	0
0	0	0	0	0	0	2	7
0	0	0	0	0	0	0	0
0	0	0	0	0	0	0	0
0	0	0	1	0	0	0	0
0	0	0	0	0	0	0	0
1	2	1	0	1	0	0	0
0	0	0	0	0	0	0	0
0	0	0	0	1	0	0	0
0	0	0	0	0	0	0	0
5	1	0	0	0	0	0	0
1	0	0	0	2	0	0	7
0	0	0	0	0	0	0	0
1	0	0	0	0	0	0	0
0	0	0	0	0	0	0	0
0	0	0	0	0	0	0	0

0	0	0	0	0	0	0	0
0	1	99	0	0	0	0	0
2	0	0	1	2	0	0	0
0	0	0	0	0	0	0	0
0	0	0	0	0	0	0	0
0	0	0	1	0	0	0	0
0	0	0	0	0	0	0	0
0	0	0	0	0	0	0	0
0	0	0	0	0	0	0	0
0	0	0	0	0	0	0	0
0	0	0	0	0	0	0	0
0	0	0	0	0	0	0	0
0	0	0	0	0	0	0	0
1	0	0	0	0	0	0	0
0	0	0	0	0	0	0	0
0	0	0	0	0	0	0	0
4	5	1	0	1	0	2	7
0	0	0	0	0	0	0	0
0	0	0	0	0	0	0	0
1	0	0	0	0	0	0	0
0	0	0	0	0	0	0	0
0	0	0	0	0	0	0	0
1	0	0	0	0	0	0	0
0	0	0	0	0	0	0	0
0	0	0	0	0	0	0	0
2	2	1	0	0	0	0	0
1	0	0	0	0	0	0	0
0	0	0	0	0	0	0	0
0	0	0	0	0	0	0	0
0	0	0	0	0	0	0	0
0	0	0	0	0	0	0	0
0	0	0	0	0	0	0	0
0	0	0	0	0	0	0	0
1	0	0	0	0	0	1	1
1	2	99	0	0	0	0	0
0	0	0	0	0	0	0	0
2	0	0	0	3	0	0	0
0	0	0	0	0	0	0	0
1	0	0	0	0	0	0	0
0	0	0	0	0	0	0	0
1	0	0	0	0	0	0	0
1	0	0	0	0	0	0	0
99	0	0	0	0	0	2	7
0	0	0	0	0	0	0	0
1	0	0	0	0	0	0	0
2	0	0	2	0	0	0	0
0	0	0	0	0	0	0	0
0	0	0	0	0	0	0	0

0	0	0	0	0	0	0	0
2	1	0	0	0	0	0	0
0	0	0	0	0	0	0	0
0	0	0	0	0	0	0	#NULL!
0	0	0	0	0	0	0	#NULL!
0	0	0	0	0	0	0	#NULL!
0	0	0	0	0	0	0	#NULL!
1	0	0	1	0	0	0	#NULL!
4	0	0	0	0	0	0	#NULL!
4	0	0	0	0	0	0	#NULL!
0	0	0	0	0	0	0	#NULL!
0	0	0	0	0	0	0	#NULL!
1	0	0	0	0	0	0	#NULL!
0	0	0	0	0	0	0	#NULL!
0	0	0	0	0	0	0	#NULL!
0	0	0	0	0	0	0	#NULL!
0	0	0	0	0	0	0	#NULL!
0	0	0	0	0	0	0	#NULL!
0	0	0	0	0	0	0	#NULL!
1	0	0	0	0	0	0	#NULL!
0	0	0	0	0	0	0	#NULL!
0	0	0	0	0	0	0	#NULL!
0	0	0	0	0	0	0	#NULL!
0	0	0	0	0	0	0	#NULL!
0	0	0	0	0	0	0	#NULL!
0	0	0	0	0	0	0	#NULL!
0	0	0	0	0	0	0	#NULL!
0	0	0	0	0	0	0	#NULL!
0	0	0	0	1	0	0	#NULL!
3	2	1	1	1	1	#NULL!	#NULL!
0	0	0	0	0	0	0	#NULL!
5	5	5	0	0	0	1	#NULL!
2	1	0	0	0	0	0	#NULL!
0	0	0	0	0	0	0	#NULL!
0	0	0	0	0	0	0	#NULL!
2	1	0	0	0	0	0	#NULL!
0	0	0	0	0	0	0	#NULL!
0	0	0	0	0	0	0	#NULL!
0	0	0	0	0	0	0	#NULL!
0	0	0	0	0	0	0	#NULL!
0	0	0	0	0	0	0	#NULL!
0	0	0	0	0	0	0	#NULL!
1	0	0	0	1	0	0	#NULL!
0	0	0	0	0	0	0	#NULL!
1	1	0	1	1	0	0	#NULL!
0	0	0	0	0	0	0	#NULL!
0	0	0	0	0	0	0	#NULL!
0	0	0	0	0	0	0	#NULL!

0	0	0	0	0	0	0	#NULL!
0	0	0	0	0	0	0	#NULL!
0	0	0	0	0	0	0	#NULL!
0	0	0	0	0	0	0	#NULL!
1	0	0	0	0	0	0	#NULL!
0	0	0	0	0	0	0	#NULL!
1	0	0	0	0	0	2	#NULL!
0	0	0	0	0	0	0	#NULL!
0	0	0	0	0	0	0	#NULL!
2	1	0	0	0	0	0	#NULL!
0	0	0	0	0	0	0	#NULL!
2	2	0	1	1	0	2	#NULL!
#NULL!	#NULL!	#NULL!	#NULL!	#NULL!	#NULL!	#NULL!	#NULL!
0	0	0	0	0	0	0	#NULL!
1	0	0	0	0	0	2	#NULL!
0	0	0	0	0	0	0	#NULL!
0	0	0	0	0	0	0	#NULL!
5	0	0	0	1	0	0	#NULL!
1	0	0	0	0	0	0	#NULL!
0	0	0	0	0	0	0	#NULL!
2	0	0	0	0	0	0	#NULL!
2	0	0	0	0	0	0	#NULL!
0	0	0	0	0	0	2	#NULL!
0	0	0	0	0	0	0	#NULL!
0	0	0	0	0	0	2	#NULL!
0	0	0	0	0	0	0	#NULL!
#NULL!	3	0	0	0	0	0	#NULL!
0	0	0	0	0	0	0	#NULL!
1	0	0	0	0	0	0	#NULL!
0	0	0	0	0	0	2	#NULL!
0	0	0	0	0	0	0	#NULL!
0	0	0	0	0	0	0	#NULL!
0	0	0	0	0	0	0	#NULL!
0	0	0	0	0	0	2	#NULL!
0	0	0	0	0	0	0	#NULL!
1	0	0	0	0	0	0	#NULL!
0	0	0	0	0	0	0	#NULL!
0	0	0	0	0	0	0	#NULL!
0	0	0	0	0	0	0	#NULL!
0	0	0	0	1	0	2	#NULL!
0	0	0	0	0	0	0	#NULL!
1	0	0	0	0	0	0	#NULL!
0	0	0	0	0	0	0	#NULL!
0	0	0	0	0	0	0	#NULL!
2	0	0	0	0	0	0	#NULL!
0	0	0	0	0	0	1	#NULL!
0	0	0	0	0	0	0	#NULL!
0	0	0	0	0	0	0	#NULL!

1	0	0	0	0	0	0	#NULL!
0	0	0	0	0	0	0	#NULL!
0	0	0	0	0	0	0	#NULL!
0	0	0	0	0	0	0	#NULL!
1	0	0	0	0	0	0	#NULL!
0	0	0	0	0	0	0	#NULL!
0	0	0	0	0	0	0	#NULL!
0	0	0	0	0	0	0	#NULL!
0	0	0	0	0	0	0	#NULL!
1	0	0	0	0	0	0	#NULL!
0	0	0	0	0	0	0	#NULL!
0	0	0	0	0	0	0	#NULL!
0	0	0	0	0	0	0	#NULL!
0	0	0	0	0	0	0	#NULL!
5	2	0	0	1	0	0	#NULL!
0	0	0	0	0	0	0	#NULL!
#NULL!	#NULL!	#NULL!	#NULL!	#NULL!	#NULL!	#NULL!	#NULL!
0	0	0	0	0	0	1	#NULL!
1	0	0	0	1	0	0	#NULL!
1	0	0	0	0	0	0	#NULL!
0	0	0	0	0	0	0	#NULL!

condom_pre	oral_pre	pressurea_pre	pressureb_pre	pressurec_pre	pressured_pre	pressuree_pre
3	0	99	99	99	99	99
0	0	99	4	1	4	99
3	0	1	4	1	1	1
3	0	4	4	1	4	1
3	0	1	4	1	4	2
99	99	99	99	99	99	99
1	2	2	4	1	1	3
3	2	3	2	2	2	2
3	2	99	99	99	99	99
3	2	1	1	1	1	1
3	0	3	3	3	99	2
3	0	1	4	1	4	1
3	0	2	4	1	4	1
3	0	1	1	1	1	1
0	0	1	1	1	1	1
3	0	2	3	2	3	2
3	0	99	99	99	99	99
99	0	2	3	3	2	2
3	0	99	4	1	4	2
1	1	2	3	2	3	2
99	99	99	99	99	99	99
99	99	1	1	1	1	1
3	0	99	99	99	99	99
3	0	1	4	1	4	2
3	0	99	4	1	4	2
3	0	1	4	1	4	1
3	0	2	3	2	3	2
3	0	4	4	1	4	1
3	0	3	3	2	3	1
3	0	1	1	1	1	1
3	0	1	1	1	1	1
1	1	1	4	4	4	1
3	0	2	4	2	2	1
3	0	1	1	1	1	1
0	0	99	99	99	99	99
3	0	1	4	1	4	1
3	0	2	3	2	3	2
3	0	1	4	1	4	1
3	0	1	4	1	4	1
3	0	1	1	2	3	1
3	0	1	1	1	4	1
3	0	2	1	1	1	1
3	0	1	4	99	99	99
3	0	1	4	1	4	1
3	0	3	1	99	3	1
3	0	1	4	1	4	1
1	1	2	3	2	3	2

3	0	4	4	2	4	3
3	0	1	4	1	4	1
3	0	1	4	1	4	1
3	0	2	3	2	3	2
1	0	3	4	1	3	2
3	0	1	4	2	4	2
3	0	1	4	1	4	1
2	2	2	4	1	4	1
3	0	1	4	1	4	1
3	0	1	4	1	3	2
3	0	2	3	2	3	2
3	0	1	1	1	4	1
3	0	2	3	2	3	3
3	0	1	4	1	4	1
3	0	1	4	1	4	1
0	0	2	4	1	4	1
3	0	2	2	2	3	2
3	0	1	4	1	4	3
3	0	2	4	2	4	2
3	0	1	4	2	1	2
0	0	2	2	2	2	2
3	0	1	4	1	4	1
3	0	1	4	1	4	1
3	0	3	4	1	2	1
3	0	1	4	1	4	1
3	0	4	4	1	4	1
0	0	4	4	4	4	4
2	0	2	3	3	3	2
3	0	2	4	2	3	2
3	0	1	4	1	4	1
3	0	1	4	1	4	1
3	0	2	3	2	3	2
3	0	1	4	1	4	1
3	1	1	4	1	4	1
3	0	1	4	1	4	1
3	0	1	4	1	4	1
3	0	1	4	1	4	1
3	0	1	4	1	4	1
3	0	3	4	1	4	1
3	0	1	4	1	4	1
3	0	2	1	2	3	2
0	1	3	3	2	2	3
3	0	1	4	1	4	1
0	0	3	2	2	2	2
3	0	2	3	2	3	2
3	0	1	4	1	4	1
3	0	1	4	1	4	1
3	0	4	4	1	4	1

3	0	1	4	2	2	2
3	0	1	4	1	4	1
3	0	3	3	2	3	2
3	0	1	4	1	4	1
3	0	1	4	1	4	1
3	0	1	4	1	4	1
3	0	1	4	1	4	1
3	0	1	4	1	4	1
1	0	1	4	1	4	99
3	0	1	4	1	4	1
3	0	4	4	1	4	1
1	0	4	4	2	2	2
3	0	2	4	1	4	1
3	0	2	3	1	3	1
3	0	2	3	2	2	2
1	1	1	4	1	4	1
3	0	1	4	1	4	1
3	0	2	1	1	3	1
3	0	2	3	1	3	1
3	0	3	4	2	4	3
3	0	1	4	1	4	2
3	0	1	4	1	4	1
1	0	4	4	4	4	4
3	0	4	4	1	4	1
3	0	1	4	1	4	1
1	1	1	1	99	99	4
3	0	4	3	3	3	1
3	0	2	4	1	4	1
3	0	2	3	2	2	3
3	0	1	4	1	4	1
3	0	99	1	1	4	1
3	0	1	99	4	1	4
3	0	1	4	1	4	1
3	0	1	4	1	4	1
3	0	2	3	2	3	3
3	0	4	4	1	4	1
2	0	2	4	1	3	3
3	0	2	4	1	3	2
3	0	3	4	1	1	1
3	0	3	4	1	4	1
3	0	1	4	1	4	2
3	0	1	1	1	1	1
3	0	1	4	1	4	2
3	0	2	4	1	4	1
3	0	1	4	1	4	1
3	0	1	4	1	4	1
3	0	1	4	2	4	2
3	0	99	4	1	4	2

3	0	4	4	1	4	1
3	0	2	3	1	4	3
3	0	1	4	1	1	1
3	0	1	4	1	4	1
2	0	99	4	2	4	2
3	0	1	4	2	2	1
0	0	2	2	2	2	2
3	0	3	4	1	3	2
3	0	3	3	1	99	99
3	0	3	1	2	1	4
3	0	1	4	1	4	1
3	0	1	1	1	1	1
0	0	1	3	2	3	2
3	0	1	4	2	2	2
3	0	2	2	1	3	1
3	0	2	2	1	2	2
3	0	1	99	1	1	1
3	0	1	4	1	4	3
3	0	1	4	1	4	1
3	0	2	3	1	3	2
0	0	4	4	4	3	3
3	0	3	3	2	3	3
3	0	1	4	1	4	1
3	0	1	4	1	1	1
3	0	1	4	1	4	1
3	0	1	4	1	1	1
3	0	2	3	2	3	2
3	0	3	3	99	3	4
3	0	1	4	1	4	1
3	0	4	4	1	4	2
3	0	99	99	99	99	99
3	0	1	4	2	3	1
0	0	2	3	2	3	2
3	0	4	3	1	4	1
3	0	4	4	1	4	1
3	0	1	4	1	4	1
0	2	1	4	1	3	1
0	0	2	2	2	2	2
3	0	4	4	4	4	1
3	0	1	1	1	4	1
3	0	1	4	1	4	1
99	99	4	3	3	3	4
3	0	1	4	1	4	1
3	0	1	4	1	4	1
3	0	1	4	1	4	1
3	0	1	4	1	4	4
3	0	4	4	4	4	1
3	0	1	4	1	4	1

3	0	1	4	1	4	1
3	0	1	4	1	4	1
2	2	1	4	4	1	1
2	0	3	3	2	3	2
3	0	1	3	1	4	1
3	0	4	4	1	4	1
3	0	1	3	2	3	2
3	0	1	4	1	4	1
3	0	3	3	3	3	3
3	0	1	4	1	4	1
3	0	3	4	1	4	1
3	0	1	1	1	4	2
3	0	1	4	1	4	1
1	1	2	4	2	4	2
3	0	4	4	1	4	1
3	0	1	4	1	4	1
3	0	1	4	1	4	1
3	0	1	4	1	4	1
3	0	1	4	1	3	1
3	0	1	4	1	4	2
3	0	1	4	1	4	1
3	0	2	3	2	2	2
2	2	1	2	2	2	2
3	0	1	4	1	4	2
3	1	1	4	1	4	2
3	0	1	4	1	4	1
3	0	1	4	1	4	1
3	0	1	4	1	1	1
3	0	1	4	1	4	1
3	0	1	4	1	4	1
3	0	1	4	1	4	1
2	2	1	4	1	4	1
3	0	1	4	1	4	1
3	0	1	4	1	4	1
3	0	4	4	2	3	1
3	1	1	4	1	4	1
3	0	2	4	1	4	1
3	0	3	3	2	3	2
3	0	2	3	2	4	1
3	0	1	4	1	4	1
3	0	2	3	2	4	2
1	1	1	3	2	3	2
3	0	1	4	1	4	1
3	0	1	3	1	4	99
3	0	1	4	1	4	3
3	0	1	4	1	4	1
0	0	4	4	1	4	1
3	0	4	4	2	1	1

3	0	2	3	2	3	2
3	0	1	4	1	4	1
3	0	1	4	1	4	2
2	0	1	4	1	4	1
0	0	2	3	2	3	2
3	0	1	4	1	4	1
3	0	1	4	1	4	1
0	0	2	3	99	99	3
3	0	2	3	2	3	2
0	0	1	4	2	3	1
3	0	1	4	1	4	99
0	0	99	2	2	2	2
3	0	2	3	2	3	2
2	0	3	2	2	3	3
3	2	1	4	1	4	4
3	0	1	4	1	4	1
3	0	1	4	1	4	1
3	0	99	99	99	99	99
3	0	1	99	1	4	1
3	0	1	4	1	4	1
3	0	2	2	2	3	3
3	0	1	4	1	4	1
3	0	2	4	1	4	2
99	99	1	4	1	4	1
3	0	1	4	2	4	1
3	0	2	99	2	3	2
3	0	1	3	1	4	1
3	0	4	4	1	4	1
3	0	1	4	1	4	1
3	0	99	4	1	4	1
3	0	4	1	4	4	1
3	0	3	4	2	2	3
3	0	3	1	1	4	4
3	0	1	3	1	3	1
1	99	1	4	1	4	1
0	1	2	2	2	3	2
3	0	2	4	2	3	2
2	2	3	3	2	3	2
3	0	4	4	1	4	1
0	0	2	4	2	4	1
3	0	4	4	2	4	4
3	0	2	4	1	4	1
3	0	4	4	2	3	2
3	0	4	4	1	4	1
3	0	1	4	1	4	2
3	0	3	4	1	3	1
3	0	1	4	1	4	1
3	0	1	4	1	4	1

3	0	3	3	2	3	3
3	0	4	4	1	4	1
3	0	2	4	2	4	99
3	0	3	2	2	4	99
3	0	1	4	1	4	1
0	0	2	4	2	3	2
3	0	3	3	2	3	2
3	0	99	3	2	3	2
3	0	3	4	2	3	1
3	0	99	4	1	4	2
3	0	1	4	1	4	1
3	0	1	4	1	4	1
3	0	1	4	1	99	99
3	0	3	4	1	1	2
1	1	1	1	1	4	1
3	0	1	4	1	4	1
3	0	3	3	2	3	3
3	0	1	4	1	4	1
3	0	4	4	1	4	1
3	0	1	4	1	4	1
3	0	1	4	1	4	1
3	0	1	4	1	4	1
0	1	3	3	2	3	2
1	1	2	3	3	2	2
2	2	2	1	1	1	1
3	0	2	3	2	3	2
0	0	2	3	2	3	2
3	0	2	3	2	3	2
0	0	99	99	99	99	99
0	0	99	4	2	4	2
3	0	4	4	1	4	1
3	0	3	3	2	3	3
2	2	2	4	1	4	1
1	0	2	4	1	4	1
0	0	4	4	1	4	1
1	1	1	4	1	4	1
1	1	1	4	1	4	2
3	0	2	4	1	4	1
3	0	1	1	1	1	3
3	0	2	4	1	4	1
3	0	1	4	2	3	2
3	0	1	4	1	4	1
3	0	3	3	2	2	2
3	0	2	4	1	4	1
3	0	1	3	2	3	1
3	0	1	4	1	4	1
99	0	3	4	1	4	1
3	0	3	4	1	4	2

3	0	4	4	1	99	1
3	0	3	3	2	2	3
3	0	3	3	3	4	3
3	0	2	3	2	3	2
3	0	2	3	1	4	3
99	99	99	99	99	99	99
2	1	1	4	1	4	1
3	0	1	4	1	4	1
3	0	1	4	1	4	1
3	0	4	4	1	4	4
3	0	1	1	1	1	1
3	0	99	99	99	99	99
0	1	1	4	1	4	1
0	0	2	4	2	4	1
3	0	1	4	1	3	3
3	0	3	4	1	4	2
3	0	1	4	1	4	1
3	0	3	1	1	1	1
3	0	1	4	1	3	2
3	0	3	3	2	3	2
3	0	3	4	1	4	1
3	0	99	4	1	4	1
3	0	1	4	1	3	2
3	0	3	3	1	3	1
3	0	1	3	2	3	2
3	0	1	4	2	2	1
3	0	1	4	1	4	1
99	99	1	1	1	99	1
3	0	2	99	99	3	1
3	0	1	1	1	1	1
3	0	1	4	1	4	1
3	0	1	4	1	4	4
3	0	1	4	1	1	1
3	0	4	4	1	4	1
3	0	3	3	2	3	1
3	0	1	4	1	4	1
3	0	4	4	1	4	1
2	2	2	1	2	3	2
3	0	1	4	1	4	1
3	0	1	4	2	3	2
3	0	3	4	1	4	2
3	0	1	3	2	4	1
3	0	2	3	2	3	2
3	0	2	3	1	3	1
3	0	1	4	1	4	1
3	0	1	4	1	4	1
3	0	1	4	1	4	1
3	0	1	4	1	4	1

3	0	99	4	1	4	1
3	0	99	4	1	4	1
3	0	1	3	1	4	1
3	0	1	4	1	1	1
3	0	1	4	1	4	1
3	1	1	4	2	1	1
3	0	1	4	2	3	2
3	0	2	3	2	3	2
3	0	2	3	3	3	3
3	0	1	4	1	4	1
3	0	99	99	99	99	99
3	0	1	4	1	4	1
3	0	3	3	3	2	2
3	0	99	3	2	2	2
3	0	1	1	1	1	1
3	0	4	4	4	4	4
0	1	3	3	1	3	1
3	0	1	4	1	4	1
3	0	2	3	2	3	2
3	0	3	4	2	4	2
3	0	3	3	3	3	3
3	0	99	1	1	2	1
1	0	1	4	1	2	2
2	99	3	3	3	3	3
3	0	2	3	2	3	1
3	2	1	4	1	4	1
3	0	2	4	1	4	1
3	0	1	4	1	4	1
2	0	99	99	99	99	99
2	2	2	3	2	3	2
3	0	1	4	1	4	1
3	0	99	4	4	4	1
3	2	3	4	2	1	1
3	0	2	3	2	3	3
3	0	1	1	1	1	1
3	0	2	4	1	4	1
99	2	4	4	4	4	2
3	0	4	4	1	4	1
3	0	1	4	1	4	1
3	0	1	4	1	4	1
3	0	1	4	1	4	1
3	0	3	4	1	4	1
1	0	1	3	1	4	1
3	0	4	4	3	3	1
3	0	1	4	1	4	1
3	0	1	4	1	1	1
3	0	2	4	1	3	1
3	0	2	1	1	1	1

3	0	1	4	1	4	1
3	0	99	99	99	4	1
3	0	1	4	1	4	1
3	0	1	4	1	4	1
3	0	1	4	1	4	2
3	0	1	4	1	4	2
3	0	1	4	1	4	1
3	0	1	4	1	4	1
0	1	2	3	2	3	1
3	0	2	4	1	4	2
3	0	1	3	1	4	1
3	0	2	3	1	3	2
3	0	1	4	1	4	1
3	0	1	4	1	4	1
3	0	2	3	2	3	2
3	0	1	1	1	1	1
3	0	2	4	4	4	1
3	0	1	4	1	4	1
3	0	1	4	1	4	1
3	0	3	3	2	3	2
3	0	1	4	1	4	1
3	1	1	4	1	4	1
3	0	1	4	1	4	1
0	1	2	3	2	3	2
3	0	2	4	1	4	1
3	0	3	3	1	3	2
2	2	1	4	1	4	2
3	0	1	4	1	4	1
3	0	2	3	1	4	1
0	0	2	3	2	3	2
3	0	2	4	1	4	1
2	2	2	4	1	4	2
3	0	1	4	1	4	1
3	0	1	1	1	4	1
3	0	4	4	1	3	1
3	0	2	4	4	3	2
3	0	2	4	1	4	1
3	0	1	4	1	4	1
3	0	2	3	2	3	2
3	0	1	1	1	4	2
3	0	1	4	1	4	1
3	0	1	99	99	4	1
3	0	2	1	1	1	1
2	0	1	4	1	4	2
3	0	2	2	3	3	1
0	0	3	4	4	4	4
3	0	2	3	2	2	3
3	0	4	4	1	1	1

3	0	1	1	1	3	4
3	0	1	4	1	4	2
3	0	1	4	1	4	1
3	0	99	99	1	3	1
2	0	2	3	1	4	1
3	0	99	4	2	3	1
2	2	99	99	99	4	99
3	0	2	99	99	4	2
1	0	1	4	1	4	1
0	1	4	4	4	4	4
3	0	1	4	1	4	1
2	2	1	4	1	3	1
3	0	1	4	1	4	1
3	0	1	4	2	3	1
3	0	4	4	4	4	4
3	0	1	4	1	4	1
3	0	1	4	1	1	1
3	0	2	2	2	2	2
3	0	1	1	1	4	1
0	99	1	4	1	4	2
3	0	1	4	1	4	1
3	0	2	4	1	4	1
3	0	1	3	1	4	2
3	0	1	4	1	4	1
99	99	99	99	99	99	99
3	0	1	3	1	3	2
3	0	1	3	1	4	2
3	0	4	3	1	4	3
3	0	1	4	1	4	1
3	0	1	4	1	4	1
3	0	1	3	99	99	1
3	0	1	1	99	4	99
3	0	1	4	1	4	1
3	0	2	4	2	2	2
0	0	2	2	2	2	2
3	1	1	3	3	3	2
3	0	3	3	2	3	2
3	0	3	2	2	3	1
3	0	2	3	1	3	2
3	0	4	2	3	3	4
3	0	2	4	1	4	1
2	2	99	3	2	3	2
3	0	1	4	1	3	1
3	0	3	4	2	3	2
3	2	1	4	1	4	1
3	0	1	4	1	1	1
3	0	4	4	1	1	1
3	0	2	4	1	4	1

3	0	2	4	1	1	1
3	0	1	4	1	4	1
99	99	99	4	1	4	99
3	0	1	4	2	4	2
3	0	1	4	1	4	1
3	0	1	4	1	4	1
3	0	2	3	2	4	2
3	0	2	4	99	4	2
3	0	1	1	1	4	1
2	2	2	3	2	3	2
3	0	4	4	1	4	1
3	0	1	4	1	4	1
3	0	1	4	1	4	1
3	0	1	4	1	1	1
3	0	1	4	1	4	1
3	0	1	4	1	4	1
3	0	1	4	1	4	1
3	0	1	4	1	4	1
3	0	2	4	1	4	1
3	0	1	4	1	4	1
3	0	3	3	2	3	2
3	0	1	1	99	4	1
3	0	4	4	1	4	1
2	2	4	4	4	4	4
3	0	1	4	1	4	1
3	0	2	3	2	3	2
3	0	1	4	1	4	1
3	1	4	4	4	4	4
3	0	1	4	1	4	1
3	0	2	4	1	4	1
3	0	4	4	1	4	1
0	2	2	3	1	4	2
3	0	1	4	1	4	1
0	0	3	4	2	4	1
3	0	99	4	1	4	99
3	0	99	99	99	99	99
3	0	2	3	2	3	2
3	0	1	4	1	4	1
3	0	2	4	1	4	1
3	0	1	4	1	4	2
3	0	4	4	1	4	1
3	0	1	1	1	4	1
1	0	2	4	1	4	2
3	0	1	2	2	3	2
3	0	2	3	2	3	2
99	99	4	4	4	4	4
99	99	99	99	99	99	99
3	0	3	4	1	4	1

3	0	2	2	2	2	2
3	0	1	99	1	4	1
3	0	1	4	1	1	1
1	0	2	4	1	3	1
3	0	1	4	1	4	1
3	0	1	4	1	4	1
3	0	2	4	2	4	1
3	0	1	4	1	4	1
3	0	1	4	2	4	2
3	0	2	99	3	2	3
1	1	2	4	1	3	1
2	2	1	2	1	3	3
3	0	1	4	1	4	1
3	0	1	4	1	4	1
3	0	1	4	1	4	2
0	0	2	2	2	2	2
3	0	99	99	1	4	1
3	0	1	4	1	4	1
2	2	1	4	1	4	1
3	0	4	4	1	4	1
2	2	2	2	2	3	3
3	0	1	4	1	4	1
3	0	2	99	99	99	99
3	0	1	4	1	4	1
0	0	2	4	1	4	2
3	0	1	4	1	4	99
0	0	4	4	1	4	1
3	0	1	3	2	3	99
3	0	2	2	2	2	2
3	0	1	1	1	1	1
99	99	99	99	99	99	99
3	0	4	4	1	4	1
3	0	99	4	1	4	4
3	0	1	4	1	4	1
3	0	99	99	99	99	99
3	0	99	3	2	2	2
99	99	4	4	1	4	1
3	0	2	4	1	4	2
3	0	1	4	1	4	1
3	0	1	4	1	4	1
3	0	1	1	1	1	1
3	0	1	4	1	4	1
3	0	1	3	1	3	2
3	0	1	4	1	4	1
3	0	1	1	1	1	3
3	0	1	4	1	4	1
3	0	1	4	1	4	1
3	0	1	1	1	1	2

0	2	1	4	1	4	2
3	0	2	3	2	3	2
3	0	1	4	1	4	1
2	0	1	3	2	1	1
3	0	2	4	1	3	2
3	0	1	99	99	4	1
3	0	1	3	1	3	1
3	0	1	1	1	4	2
3	0	4	3	2	4	2
2	0	4	99	1	99	3
0	0	2	3	2	3	2
3	0	4	4	4	4	1
3	0	1	4	1	4	1
3	0	2	3	2	3	3
3	0	2	3	2	3	2
3	0	1	2	2	2	2
2	0	2	4	1	3	3
3	0	2	3	2	3	2
3	0	2	4	2	4	2
3	0	1	1	1	1	1
1	0	1	4	1	4	2
3	0	3	4	3	3	3
3	0	1	3	2	3	2
3	0	2	4	2	3	2
3	0	1	4	4	3	2
3	0	3	2	3	3	2
3	0	1	4	1	4	1
3	0	99	3	2	3	2
3	99	99	99	1	4	99
3	0	1	3	1	4	2
3	0	1	4	1	4	2
3	0	1	4	1	4	1
3	0	2	4	1	4	3
3	0	99	3	2	3	2
3	0	99	3	1	3	1
3	0	2	3	2	3	2
0	0	2	2	2	2	2
3	0	1	1	1	4	1
3	0	2	1	1	4	1
3	0	1	4	1	4	1
1	1	1	4	1	4	1
3	0	2	3	2	3	2
3	2	1	4	1	4	1
3	0	1	4	1	4	1
3	0	1	4	1	4	1
3	0	2	3	2	3	2
3	0	1	99	1	99	2
3	0	1	4	1	4	1

3	0	4	4	1	4	4
3	0	4	4	1	4	4
99	99	99	99	99	99	99
3	0	1	4	1	4	1
3	0	1	4	1	4	1
3	0	2	3	2	3	2
3	0	1	4	1	4	1
3	0	1	3	1	4	1
99	99	99	99	99	99	99
3	0	2	4	1	3	2
2	1	1	4	1	4	1
3	0	2	2	1	4	2
3	0	2	4	1	4	2
2	2	4	4	2	4	3
3	0	4	4	1	3	1
3	0	1	1	1	1	1
3	0	1	1	1	4	1
2	2	1	4	1	4	1
3	0	1	4	4	3	1
3	0	4	4	2	3	1
3	0	1	4	1	1	1
3	0	1	4	1	4	1
3	0	3	2	3	2	3
3	0	2	3	1	4	2
3	0	1	4	1	1	1
3	0	99	4	99	99	99
3	0	2	3	1	4	2
3	0	3	4	1	4	1
3	0	1	4	1	4	1
3	0	1	4	1	4	1
3	0	3	4	1	3	3
3	0	3	3	1	3	2
3	0	2	99	2	3	2
3	0	1	4	1	4	1
3	0	3	3	3	3	3
3	0	99	4	1	4	1
3	0	2	2	2	99	99
99	99	2	1	2	1	2
3	0	1	4	1	4	1
3	0	99	3	2	3	2
2	99	1	4	1	4	1
2	2	3	3	2	3	3
3	0	1	1	1	1	1
3	0	1	1	2	4	2
3	0	99	99	99	99	99
3	0	3	4	2	3	3
3	0	1	4	1	4	1
3	0	1	4	2	3	1

3	0	2	3	1	3	1
3	0	99	4	1	4	1
3	0	1	4	1	4	2
3	0	4	4	1	4	1
3	2	99	3	2	3	2
3	0	1	1	1	1	1
3	0	4	4	1	4	1
3	0	4	4	2	4	2
3	0	2	4	1	4	1
3	0	2	4	1	1	3
3	0	1	4	1	4	1
3	0	1	1	1	1	2
3	0	2	3	2	3	2
3	0	1	4	1	4	1
3	0	99	4	1	4	1
3	0	1	4	1	4	1
3	0	4	4	1	4	3
3	0	99	99	99	99	99
3	0	3	4	2	3	1
3	0	1	1	99	99	1
3	0	1	4	1	4	2
3	0	1	3	1	3	1
3	0	1	4	1	4	1
3	0	1	4	1	4	1
3	0	2	3	2	3	2
3	0	2	3	1	4	1
3	0	1	4	1	4	99
3	0	1	1	1	1	1
3	0	3	3	3	3	2
3	0	1	3	2	3	2
3	0	3	4	1	1	3
3	0	1	4	1	4	1
3	0	1	99	1	4	1
3	0	1	1	1	3	1
3	0	1	4	2	4	2
3	0	1	1	1	4	2
3	0	1	4	1	4	1
3	2	1	4	1	4	1
2	2	2	2	2	2	2
3	0	3	4	1	4	1
3	0	1	3	2	4	2
3	0	2	99	3	99	2
3	0	2	1	2	1	1
3	0	1	4	1	4	1
3	0	4	4	2	4	3
0	0	4	3	2	3	2
0	0	3	3	2	3	2
3	0	4	4	1	4	1

3	0	3	4	1	4	3
3	0	4	4	1	1	1
3	0	2	4	99	3	1
3	0	3	4	1	4	1
3	0	1	4	1	4	1
3	0	1	2	2	2	2
3	0	3	4	1	3	2
3	1	1	4	1	4	1
3	0	1	4	1	4	1
3	0	4	4	2	3	4
3	0	1	1	1	1	2
0	0	1	1	1	4	1
99	99	99	99	99	99	99
3	0	3	3	1	3	1
3	0	1	4	1	4	1
3	0	1	4	1	4	1
3	0	1	3	2	3	2
3	0	2	3	1	3	2
3	0	1	4	1	4	1
3	0	2	4	1	4	1
3	0	2	3	2	3	2
3	0	1	4	1	4	2
3	0	1	2	1	1	1
3	0	1	4	1	4	1
3	0	1	4	1	4	1
99	0	2	3	2	3	2
1	0	4	2	2	4	2
3	0	2	1	1	2	3
2	2	1	1	1	1	1
3	0	2	2	2	2	2
1	0	1	4	2	1	3
3	0	1	4	2	4	2
0	0	3	4	1	4	3
3	0	3	4	1	4	1
3	0	1	4	1	4	1
3	0	2	3	2	2	2
3	0	3	2	2	2	3
3	1	3	3	3	99	2
3	0	1	4	1	4	1
3	0	2	3	1	1	3
3	0	1	3	2	4	2
2	2	1	4	1	4	1
3	0	3	4	1	3	2
3	0	3	4	99	4	99
3	0	4	4	1	4	1
3	0	4	4	1	4	1
3	0	1	4	1	4	1
3	0	1	4	1	4	1

3	0	3	3	2	2	1
3	0	2	4	1	4	1
0	2	3	3	2	3	3
3	0	1	4	1	4	1
3	0	4	4	1	4	2
2	2	2	1	2	1	1
3	0	1	4	1	4	2
3	0	1	4	1	1	4
3	0	1	4	1	4	4
3	0	2	4	1	1	1
3	0	1	4	2	1	2
3	0	1	4	1	4	1
0	0	2	4	1	99	1
3	0	4	4	1	4	1
3	0	1	4	1	4	1
3	0	3	3	3	3	4
3	0	3	3	2	3	3
2	0	1	4	1	4	4
1	2	3	4	1	1	3
2	0	99	99	1	4	4
3	0	1	4	1	4	1
1	1	1	4	1	4	1
3	2	2	3	99	99	2
3	0	2	3	2	3	3
3	0	1	4	1	4	1
3	0	1	4	1	4	2
3	0	99	3	2	4	1
3	0	1	4	1	1	2
3	0	2	3	2	3	2
3	0	1	4	1	4	1
3	0	1	4	1	4	1
2	2	1	4	1	3	2
3	0	1	4	1	4	1
3	0	1	4	1	4	2
3	0	2	2	2	3	2
3	0	1	4	1	4	1
3	0	1	3	1	3	1
3	0	1	4	1	4	2
0	1	2	3	2	3	99
3	0	1	4	4	4	1
3	0	1	4	1	4	99
3	0	1	3	1	3	2
3	0	1	4	1	4	1
3	0	2	4	1	1	2
3	0	4	3	2	2	3
3	0	3	4	1	4	3
1	0	4	2	2	3	4
3	0	1	4	1	1	1

3	0	1	4	1	4	1
3	0	1	4	1	4	1
3	2	3	4	2	4	2
3	0	1	4	1	4	1
3	0	2	3	2	3	2
3	0	2	4	4	4	3
3	0	1	3	1	3	2
3	0	2	4	1	3	1
3	0	1	4	1	4	1
99	0	1	4	1	4	2
3	0	2	3	1	3	1
3	0	3	3	1	4	1
3	0	2	3	3	2	2
3	0	1	4	1	4	1
3	0	1	4	1	1	1
3	0	1	4	1	3	1
3	0	2	4	1	3	2
3	0	2	4	2	99	2
0	0	4	4	3	3	2
3	0	1	4	1	4	1
3	0	2	4	1	4	3
3	0	2	4	1	4	1
3	0	3	3	2	3	3
0	0	1	1	1	1	1
3	0	3	4	4	3	4
3	0	1	4	1	4	1
3	0	4	4	1	4	1
3	0	1	4	1	4	1
0	1	3	2	3	2	4
3	0	1	4	1	4	2
3	0	1	4	1	4	1
3	0	1	4	1	4	1
3	0	2	3	2	3	2
3	0	3	3	2	3	3
3	0	1	4	1	4	1
3	0	1	4	1	4	1
0	0	2	2	2	2	2
3	0	1	4	1	4	1
3	0	1	4	1	4	1
3	0	1	4	1	4	1
1	1	4	3	2	3	3
3	0	4	4	1	4	1
1	0	4	4	4	4	4
1	0	2	3	2	2	2
3	0	1	4	1	4	4
1	1	2	2	3	2	2
3	0	1	4	1	1	1
3	0	1	4	1	4	3

3	0	1	4	1	4	1
3	0	2	3	1	1	1
3	0	3	3	3	3	2
3	0	1	4	1	4	1
0	1	4	1	4	1	4
3	0	1	4	2	3	1
3	2	2	4	1	4	1
3	0	1	4	1	4	1
3	0	4	4	1	4	1
3	0	4	4	4	4	4
3	0	1	4	2	3	2
3	0	1	4	2	4	2
3	0	1	4	1	1	4
3	0	1	3	1	1	2
2	99	2	2	2	2	2
3	2	3	3	4	3	4
3	0	99	4	1	4	1
3	0	3	3	2	3	2
3	0	3	3	1	4	2
3	0	1	4	1	4	1
3	0	2	4	1	4	2
3	0	2	2	2	3	3
3	0	2	3	2	3	2
3	0	99	4	1	1	1
3	0	1	4	1	4	2
3	0	1	4	1	4	1
0	2	1	4	1	4	1
3	0	1	4	1	4	1
3	0	1	3	1	4	1
0	0	4	4	1	1	1
2	2	2	3	2	4	3
0	2	1	1	1	1	1
0	0	1	4	1	4	1
3	0	1	4	1	4	1
3	0	1	4	1	4	99
3	0	1	4	1	4	1
3	2	3	4	1	4	2
3	2	3	2	4	4	1
3	0	4	4	1	4	1
1	0	4	4	1	4	1
3	0	1	2	4	4	1
3	0	1	4	1	4	1
3	0	1	4	1	4	1
3	0	2	4	1	4	1
99	0	2	4	2	3	3
3	0	99	99	99	4	1
3	0	1	4	1	4	1
3	0	4	4	1	4	1

3	0	1	1	1	4	1
2	2	4	4	2	1	2
3	0	1	1	1	1	1
3	0	99	3	2	3	2
1	0	1	3	1	3	1
2	2	1	4	1	4	1
3	0	1	4	1	4	1
3	0	99	4	1	3	1
3	2	1	1	3	2	3
3	0	4	4	1	4	1
3	0	1	4	1	4	2
3	0	1	4	2	4	1
3	0	1	4	1	4	1
3	0	2	3	2	3	1
3	0	4	4	1	4	1
3	0	2	3	2	3	1
3	0	4	4	1	4	4
3	0	4	3	4	3	4
3	0	2	3	1	3	2
3	0	2	3	2	3	2
3	0	1	4	1	4	2
3	0	2	3	2	3	2
3	0	2	3	2	99	99
3	0	1	4	1	4	1
3	1	3	3	2	3	2
3	0	2	4	1	4	2
3	0	2	3	2	3	2
3	0	1	99	99	2	2
3	0	1	4	1	4	1
3	0	1	4	1	4	1
3	0	1	4	1	4	1
3	0	1	4	1	4	99
3	0	2	4	1	3	99
3	0	1	3	2	4	1
3	0	1	4	1	4	2
3	0	2	2	2	2	2
3	0	2	3	2	3	2
3	0	1	4	1	4	1
3	0	2	3	2	4	1
3	0	2	3	2	3	2
3	0	1	4	1	4	1
3	0	2	4	1	4	2
3	0	1	4	99	99	3
0	0	1	4	1	3	99
3	0	1	4	1	4	1
3	0	2	2	2	2	2
3	0	1	4	1	1	1
3	0	1	4	1	4	1

3	0	4	4	1	1	1
3	0	2	3	2	3	2
3	0	1	4	1	4	3
3	0	1	4	1	4	1
3	0	99	99	99	99	99
3	0	99	4	1	4	2
3	0	1	4	1	4	3
0	0	2	2	2	2	2
3	0	3	3	2	3	2
3	0	2	4	2	4	4
3	0	1	4	1	4	1
3	0	2	3	2	3	2
3	0	1	4	1	4	1
3	0	1	3	3	3	2
3	0	1	4	1	4	1
3	0	2	4	1	4	1
3	0	2	1	1	1	1
3	0	2	3	2	3	2
0	0	4	1	4	4	4
3	0	1	1	1	1	1
2	2	4	4	1	4	4
2	2	3	3	3	4	4
3	0	99	4	1	4	1
3	0	1	4	1	4	1
3	0	1	4	1	4	1
3	0	4	4	1	4	1
3	0	2	3	2	3	2
3	0	99	3	2	3	3
3	0	1	4	1	4	1
3	0	2	4	1	4	1
0	0	2	3	2	4	3
3	0	2	3	1	3	1
3	0	1	4	1	4	1
3	0	1	4	1	4	1
3	0	2	4	1	4	3
3	0	1	4	2	3	2
3	0	1	4	1	4	1
3	0	1	1	1	4	1
3	0	1	4	1	4	1
3	0	1	4	1	4	1
3	0	1	4	1	4	1
3	0	1	4	1	4	1
3	0	2	3	2	3	2
0	0	3	3	2	3	2
2	0	4	4	1	4	2
0	1	2	3	2	3	2
2	2	2	3	2	2	2
3	0	1	2	3	3	2

3	0	1	4	1	4	1
3	0	4	4	1	1	1
3	0	2	3	2	2	2
0	0	1	1	2	3	1
3	0	4	4	1	4	1
3	0	1	4	1	4	1
3	0	2	3	2	2	2
3	0	2	4	1	3	2
3	0	1	4	1	4	1
3	0	1	4	1	4	2
3	0	99	99	99	99	1
3	0	1	4	1	4	1
3	0	1	4	1	4	1
3	0	2	3	2	2	2
3	0	99	99	99	4	99
3	0	1	4	1	4	1
3	0	1	4	1	4	1
3	2	1	4	1	4	1
3	0	1	4	1	3	2
3	0	99	4	1	3	99
3	0	1	3	2	4	3
0	0	3	3	2	3	2
3	0	3	3	2	3	2
3	0	2	3	1	4	1
2	2	1	4	1	4	1
3	0	2	4	1	4	1
3	0	99	99	1	1	2
3	0	1	4	1	4	1
3	0	1	4	1	4	1
3	0	99	4	1	4	1
3	0	1	4	1	4	1
3	0	1	4	1	4	2
3	0	2	4	1	4	1
3	0	1	4	1	4	1
3	0	1	3	2	3	2
3	0	1	4	1	4	1
3	0	1	4	1	4	1
3	0	1	4	1	4	99
3	1	2	3	2	3	3
3	0	1	3	2	4	1
3	0	1	99	1	4	1
3	0	1	4	1	4	1
99	99	99	4	1	4	99
1	2	2	3	1	3	3
3	0	1	4	1	4	1
3	0	2	4	1	4	2
3	0	1	4	1	4	1
3	0	1	4	1	4	1

3	0	2	3	1	3	2
3	0	2	2	2	2	2
3	0	3	99	2	4	2
3	1	1	4	1	4	1
3	0	1	99	4	3	1
2	0	2	3	2	1	2
1	1	2	3	2	3	2
3	0	1	4	1	4	1
3	0	1	1	1	1	1
3	0	1	4	1	4	1
0	0	4	4	4	4	4
3	0	1	4	1	4	1
3	0	1	4	1	4	2
3	0	1	3	2	3	2
3	1	2	3	3	2	3
0	2	2	3	3	2	3
3	0	3	4	1	4	1
3	0	2	3	1	2	2
3	0	1	4	1	4	1
0	1	99	99	99	99	99
3	0	1	4	1	4	1
1	1	1	4	1	4	1
3	0	2	2	2	3	2
3	0	1	1	1	4	1
3	0	1	3	2	3	2
3	0	3	3	2	3	2
3	0	3	3	3	3	3
3	0	1	4	1	4	1
3	0	1	4	1	4	1
3	0	1	4	1	4	2
3	0	1	4	1	4	1
3	0	1	4	1	4	1
3	0	1	4	1	4	1
1	1	4	4	1	4	1
0	1	1	3	2	3	2
3	0	1	4	1	1	1
3	0	1	4	1	4	1
3	0	2	3	2	3	2
3	1	2	4	2	3	2
3	0	1	4	1	4	1
3	0	4	4	2	2	2
3	0	1	4	1	4	1
3	0	4	4	1	4	1
3	0	1	4	1	4	1
3	0	1	4	1	4	1
3	0	2	4	1	4	1
3	0	1	4	2	3	3
3	0	2	4	2	4	2

3	0	1	4	1	4	1
0	1	2	4	2	3	2
3	0	2	3	2	3	2
3	0	2	3	2	2	2
3	0	1	4	1	4	1
0	1	2	3	2	2	2
3	0	1	3	2	3	2
0	0	3	3	3	3	3
1	1	3	3	2	2	2
0	0	1	1	1	1	1
3	0	1	3	2	3	2
3	0	1	4	1	1	2
3	0	1	4	1	4	1
3	0	1	4	2	3	2
3	0	2	4	1	4	1
3	0	2	3	1	2	3
3	0	1	4	1	4	1
3	0	1	4	1	4	1
3	0	2	3	2	3	1
3	0	1	4	1	4	1
3	0	1	4	1	4	1
3	0	2	3	1	3	2
3	0	2	2	2	2	2
3	0	4	99	2	3	2
3	0	2	3	2	3	3
3	0	1	4	1	4	1
3	0	2	3	2	2	2
3	0	1	4	1	4	1
3	0	99	99	99	99	99
3	0	4	4	1	4	1
3	0	1	3	1	4	2
3	0	1	4	1	4	1
3	0	1	4	1	4	1
3	0	1	4	1	4	1
3	0	2	2	2	1	1
3	0	1	4	1	4	1
3	0	1	1	1	4	1
3	0	1	1	1	1	1
3	0	4	3	1	99	2
3	0	1	4	1	4	1
0	1	4	1	4	1	4
3	0	1	4	1	4	1
3	2	2	1	2	1	2
3	0	1	4	1	1	2
3	0	1	4	1	4	1
3	0	1	4	1	4	1
3	0	2	2	3	2	4
3	0	1	4	2	4	1

3	0	1	4	1	4	1
0	1	1	4	1	4	1
3	0	99	3	2	3	2
3	0	1	4	1	4	2
3	0	1	4	1	4	1
3	0	99	99	99	99	99
2	2	2	2	2	3	2
3	2	1	4	1	4	1
2	2	1	4	2	4	1
3	0	2	4	1	4	2
3	0	4	4	1	4	1
3	0	1	4	1	4	1
3	0	2	2	2	3	3
0	0	2	3	2	3	2
3	0	1	4	1	4	1
3	0	1	4	1	4	1
3	0	4	4	4	4	4
3	0	2	3	1	4	2
3	0	2	3	2	3	2
3	0	1	4	1	4	1
3	0	1	4	1	4	2
3	0	1	4	1	4	1
3	0	1	1	1	1	1
3	0	1	4	1	4	1
3	0	1	3	2	3	99
3	0	2	2	2	2	2
3	0	2	3	2	3	2
3	0	1	4	1	4	1
3	0	99	4	1	4	99
3	0	2	4	1	4	2
3	0	1	4	1	4	1
1	1	1	1	1	1	1
3	0	1	4	1	4	1
3	0	1	4	1	4	1
3	2	1	4	1	4	1
0	1	1	4	1	4	1
3	0	1	4	1	4	1
3	0	1	4	1	4	1
2	2	1	3	1	1	1
3	0	1	99	99	99	99
3	0	1	4	1	4	1
1	0	1	4	1	4	1
3	0	2	4	1	4	2
3	0	3	3	3	2	2
3	0	4	4	4	4	4
2	2	1	4	1	4	99
3	0	1	4	1	4	1
3	0	1	4	1	4	1

3	0	2	4	2	3	2
2	2	2	3	2	3	2
3	0	2	3	1	3	1
1	1	4	4	4	4	4
3	0	1	4	1	4	1
3	0	3	3	3	3	2
3	1	4	1	4	1	4
3	0	1	4	1	4	1
3	0	1	4	1	4	1
3	0	2	4	2	4	1
3	0	3	4	1	4	1
3	0	1	4	1	4	1
3	0	1	4	1	4	1
3	0	1	4	1	4	1
3	0	1	4	99	99	1
3	0	1	4	1	4	1
0	1	3	2	3	2	3
3	0	2	3	2	3	2
3	0	1	4	1	4	1
3	0	1	4	1	4	1
3	0	2	4	2	4	2
3	0	2	4	1	99	4
3	0	1	4	1	4	1
3	0	1	4	1	4	1
3	0	1	4	1	4	1
3	0	3	4	2	3	2
3	0	1	4	1	4	1
3	0	1	4	1	4	1
3	0	1	1	1	1	1
3	0	1	4	2	3	2
3	0	3	3	4	3	1
3	0	1	4	1	4	1
3	0	2	3	2	3	2
3	0	2	3	2	3	2
2	2	2	1	2	1	1
3	0	1	4	1	4	1
3	0	1	4	1	4	1
3	0	1	3	1	3	1
3	0	1	4	1	4	1
2	2	1	1	1	2	1
3	0	1	1	1	4	1
3	0	3	3	4	99	99
3	0	1	4	1	4	1
3	0	4	4	1	4	1
3	0	2	4	2	3	1
3	0	1	4	1	4	1
99	99	4	4	1	4	1
3	0	1	4	1	4	1

3	0	3	3	2	3	2
3	0	4	4	4	4	4
3	0	4	4	4	4	4
3	0	1	4	1	4	1
3	0	2	3	2	2	2
3	0	1	4	1	4	1
3	0	1	4	1	4	1
3	0	1	4	1	1	1
0	0	2	2	2	2	2
3	0	3	4	1	4	1
0	0	4	4	4	4	1
3	0	1	4	1	4	1
3	0	2	3	2	3	2
2	2	1	1	1	1	2
3	0	1	4	1	4	1
3	0	3	3	3	3	3
3	0	1	4	1	4	1
3	0	2	4	4	4	2
2	2	2	3	2	2	2
3	0	1	4	1	4	1
3	0	99	4	1	4	1
3	0	1	4	1	4	2
3	0	4	4	1	4	1
0	2	2	2	1	2	3
3	0	2	2	2	2	2
3	0	1	4	1	4	1
3	0	1	4	1	4	1
3	0	1	4	1	4	1
3	0	99	4	1	4	1
3	0	1	4	1	4	1
3	0	1	4	1	1	1
1	1	3	4	1	4	3
3	0	1	4	1	4	1
3	0	1	4	1	4	1
3	0	1	4	1	4	1
3	0	2	3	2	3	2
99	99	1	4	1	1	2
3	0	1	4	1	4	1
3	2	2	4	3	2	3
3	0	1	4	1	4	1
3	0	99	99	99	99	99
3	0	2	4	1	4	1
3	0	1	4	1	4	1
3	0	1	4	4	4	4
3	0	1	3	1	4	1
3	0	2	3	2	3	2
0	0	2	3	2	3	2
3	0	1	4	1	4	1

3	0	1	4	1	4	1
0	0	1	4	2	3	2
3	0	99	3	2	4	2
3	0	1	4	1	4	1
3	0	2	4	1	4	3
3	0	3	3	2	3	2
3	0	1	4	1	4	99
1	0	1	3	4	1	1
1	1	1	1	1	4	1
3	0	1	4	1	4	1
3	0	1	4	1	1	1
3	0	1	4	1	4	1
3	0	2	3	2	3	2
3	0	2	1	1	1	1
3	0	1	3	1	3	1
3	0	2	3	2	3	2
3	0	4	4	2	4	2
3	0	1	3	1	3	2
3	0	1	3	2	3	2
99	0	3	4	1	3	2
3	0	1	4	1	4	1
3	0	1	4	4	4	1
3	0	1	4	1	4	1
3	0	4	4	1	4	1
0	0	1	1	1	1	1
3	0	1	4	1	4	1
3	0	1	4	1	4	1
3	0	1	4	1	4	1
3	0	1	4	1	4	1
3	0	3	4	1	4	1
99	0	2	3	1	3	2
3	0	3	3	2	3	3
3	0	1	4	2	4	1
3	0	1	4	1	4	1
3	0	1	4	1	4	1
3	0	4	4	1	1	1
3	0	1	4	1	4	1
3	0	2	3	2	3	2
3	0	1	4	1	4	1
3	0	1	4	1	4	1
3	0	1	4	1	4	1
3	0	1	4	1	4	1
0	0	1	3	1	3	2
3	0	3	3	2	2	2
99	99	1	4	1	4	1
1	1	1	4	1	4	1
0	1	4	4	2	4	2
3	0	2	2	1	1	1

0	99	2	2	2	2	2
3	0	99	3	2	4	1
3	0	99	4	1	4	1
3	0	1	4	1	4	1
2	99	99	4	1	4	99
3	0	99	4	1	4	1
3	0	99	4	1	4	2
3	0	1	1	1	1	1
3	0	1	4	1	4	1
2	0	3	1	2	2	3
3	0	1	4	1	4	1
3	0	1	1	1	4	1
3	0	1	4	1	4	1
3	0	1	4	1	1	1
3	0	1	1	1	4	1
0	0	99	1	1	1	1
3	0	1	4	1	4	1
3	0	1	1	1	1	1
3	0	1	4	1	3	99
3	0	1	4	1	1	1
99	99	1	3	2	3	1
3	0	99	3	2	3	1
3	0	1	4	1	4	1
3	0	2	3	1	3	1
3	0	2	4	1	1	1
3	0	2	4	2	4	2
3	0	4	4	4	4	4
0	0	2	2	3	3	3
3	0	1	4	1	4	1
3	0	1	4	1	4	1
3	0	3	4	1	4	2
3	0	1	1	1	1	1
3	0	2	4	2	3	3
3	0	2	2	2	2	2
3	0	1	4	1	4	1
3	0	1	4	1	4	1
3	0	1	4	1	4	1
3	0	1	4	1	3	1
3	0	1	4	1	4	1
3	0	1	4	1	4	1
3	0	1	4	1	4	1
3	0	1	3	1	3	1
2	2	1	4	1	4	2
3	0	1	4	1	4	2
3	0	4	4	4	4	4
3	0	1	4	1	4	1
3	0	1	4	1	4	1
0	1	2	3	4	3	4

3	0	2	2	2	2	2
3	0	1	4	1	4	1
3	0	1	4	1	4	1
3	0	3	3	3	3	3
0	0	4	4	1	4	1
3	0	4	4	1	4	1
3	0	2	3	2	3	2
3	0	2	4	2	3	2
3	0	2	4	2	3	3
3	0	1	4	1	4	1
3	0	1	2	1	2	1
3	0	1	4	1	4	1
3	0	99	4	1	4	2
3	0	2	3	2	4	1
3	0	1	4	1	4	2
0	1	3	4	1	1	2
3	0	1	4	1	4	1
3	0	1	4	1	4	1
1	0	2	3	1	3	2
3	0	2	4	2	2	2
3	2	1	4	2	3	3
3	0	1	4	1	4	2
3	0	4	4	1	4	1
0	0	1	4	1	1	1
3	0	1	4	1	4	2
3	0	99	99	99	99	99
3	0	3	4	1	4	1
3	0	1	4	1	4	99
3	0	2	3	2	3	2
3	0	1	4	1	4	2
3	1	1	4	1	4	1
3	0	1	4	1	4	1
3	0	1	4	1	4	1
3	0	1	4	1	4	1
3	0	2	4	2	4	1
3	0	1	4	1	4	1
1	1	3	3	2	2	2
3	0	2	3	2	3	2
3	0	2	3	2	3	1
3	0	1	4	1	4	2
2	2	1	4	1	4	1
3	0	1	4	1	4	1
3	0	1	4	1	4	1
3	0	1	3	2	4	4
3	0	1	4	1	4	1
3	0	1	4	1	4	1
3	0	1	4	1	4	1
3	0	1	4	1	4	1

3	0	2	4	1	4	1
3	0	3	3	2	3	3
3	0	1	4	1	4	1
3	0	2	4	1	2	2
3	0	1	4	1	4	1
3	0	1	4	1	1	1
3	0	1	4	1	4	1
3	0	99	4	1	4	1
3	0	2	3	2	3	2
3	0	1	4	1	4	1
3	0	2	2	2	3	2
3	0	2	3	1	3	2
99	0	2	3	2	3	2
3	0	1	4	1	4	1
3	0	1	3	2	3	2
3	0	1	4	1	4	1
0	0	2	2	2	2	2
3	0	2	4	1	4	1
3	0	1	4	1	1	1
3	0	1	4	1	4	1
3	2	2	2	1	3	99
3	0	1	4	1	4	1
3	0	2	3	2	3	2
2	2	1	4	1	4	1
3	0	1	3	1	3	1
3	0	2	3	1	3	3
3	0	2	4	1	4	1
1	1	2	2	2	2	3
3	0	2	2	2	4	2
3	0	2	3	2	3	2
3	0	1	4	2	3	2
3	99	1	4	1	4	1
3	0	1	3	2	3	1
3	0	1	3	1	1	1
3	0	1	4	4	1	1
2	2	1	4	1	3	3
3	0	1	4	1	4	1
3	0	1	4	1	4	1
3	0	4	4	1	1	1
3	0	2	2	2	1	2
2	2	1	1	1	1	1
2	2	4	3	1	1	2
3	0	3	4	1	4	1
3	0	1	4	1	4	1
3	0	2	3	2	3	2
3	0	1	3	1	1	1
3	0	4	4	2	4	2
3	0	1	4	1	4	1

3	0	3	1	4	1	1
3	99	3	3	2	2	2
3	0	2	3	2	3	3
3	0	99	99	99	99	99
2	2	1	1	1	1	1
2	2	1	4	1	4	1
3	0	1	3	2	3	2
3	0	1	4	1	4	1
2	0	2	3	2	3	3
3	0	2	4	2	4	4
3	0	99	99	99	99	99
3	0	1	4	1	4	1
3	0	1	4	4	1	1
3	0	1	4	1	4	1
2	2	1	4	1	4	1
3	0	1	4	1	4	1
3	0	1	4	1	3	2
3	0	2	3	2	3	3
3	2	1	3	2	4	1
3	0	1	3	1	4	2
3	0	1	2	3	2	1
3	0	1	3	1	4	2
2	2	1	4	1	4	1
3	0	3	3	2	3	2
3	0	2	4	2	3	2
3	0	99	4	1	4	1
3	2	1	4	1	4	1
3	0	1	4	1	4	1
3	0	1	4	1	4	1
3	0	1	4	1	4	1
3	0	2	3	2	3	2
2	2	1	4	1	4	1
3	0	1	4	1	4	1
3	0	2	4	1	4	2
0	1	4	1	4	1	4
0	0	1	4	2	4	3
3	0	1	4	1	4	1
3	0	3	3	2	3	3
3	0	1	4	1	4	1
2	2	3	1	4	2	3
3	0	1	4	1	4	2
99	99	1	3	1	4	3
3	0	1	4	4	4	1
0	1	1	1	4	1	4
1	1	1	4	2	2	1
0	1	1	4	1	4	2
3	0	1	4	1	4	1
3	0	99	4	1	1	1

3	0	3	4	1	3	1
3	0	1	3	1	4	1
3	0	1	4	1	4	1
3	0	2	4	2	4	1
3	0	1	4	1	4	1
2	2	1	1	1	1	1
3	0	1	4	1	4	1
3	0	1	4	1	4	1
3	0	2	3	1	4	1
3	0	1	4	1	4	1
3	0	1	99	99	99	99
1	2	2	3	2	3	1
3	0	1	4	1	4	1
3	0	1	4	1	4	1
3	0	3	3	1	4	1
3	0	1	4	1	4	1
0	0	99	99	99	99	99
3	0	1	1	1	1	1
3	0	2	3	2	3	2
3	0	2	4	1	4	1
3	0	4	4	1	4	1
3	0	1	4	1	4	1
3	0	1	4	1	4	1
3	0	1	4	1	4	1
3	0	1	4	1	3	2
3	0	2	4	2	4	2
99	99	3	3	99	3	99
3	0	2	4	1	4	1
3	0	2	3	2	3	2
3	0	1	1	1	1	1
3	0	2	4	1	4	1
3	0	2	3	1	4	99
3	0	3	1	4	2	2
2	2	2	3	2	3	3
3	0	99	4	1	4	1
3	0	1	4	2	4	1
3	0	1	4	1	4	1
3	0	1	4	1	4	1
3	0	1	4	1	4	1
3	0	1	4	1	4	1
3	0	3	4	4	4	4
3	0	2	3	2	3	99
2	2	1	4	1	99	1
3	0	2	2	2	2	2
3	0	2	2	2	2	2
3	0	99	99	99	4	1
3	0	1	4	1	4	1
3	99	4	4	1	4	1

3	0	1	4	1	4	1
3	0	2	4	99	99	1
3	0	2	3	2	2	2
3	0	1	4	1	4	1
3	0	1	4	1	4	1
3	0	2	3	2	3	2
3	0	1	4	1	3	2
3	0	1	1	1	1	1
3	0	4	4	4	4	2
2	2	2	4	1	4	1
3	0	2	3	2	3	2
3	0	2	3	2	3	2
3	0	1	4	1	4	2
3	0	99	4	1	4	99
3	0	1	4	1	4	2
3	0	1	1	1	1	1
3	0	1	4	1	4	1
3	0	1	1	1	4	1
3	0	2	2	2	2	2
3	0	1	4	1	4	1
3	0	2	3	1	3	1
3	0	1	4	1	4	1
3	0	1	3	1	4	1
99	0	1	3	2	3	3
0	0	2	3	2	3	99
3	0	1	4	1	4	1
3	0	1	4	1	4	2
3	0	2	4	1	4	2
3	0	4	4	1	4	2
3	0	1	1	1	4	2
3	0	99	3	2	3	2
99	99	99	99	99	99	99
3	0	1	4	1	4	1
3	0	1	4	1	4	1
3	0	1	4	1	4	2
3	0	2	3	2	3	1
3	0	3	3	2	3	2
3	0	1	4	1	4	1
3	0	2	4	2	3	2
3	0	1	4	1	4	2
3	0	2	3	2	3	2
3	0	1	4	1	4	1
3	0	2	3	2	3	2
3	0	1	4	1	4	1
3	0	99	3	2	3	2
3	2	1	4	1	4	1
3	0	2	3	2	3	2
3	0	2	4	1	4	2

3	0	4	4	1	4	99
3	0	1	4	1	4	1
3	0	1	4	1	4	1
3	1	1	4	1	4	1
3	0	1	4	1	4	1
2	2	1	99	1	99	99
3	0	1	4	1	4	1
3	0	1	4	1	4	1
3	0	1	4	1	4	1
2	0	2	3	1	3	2
2	2	2	3	2	3	2
3	0	2	3	1	1	2
3	0	1	4	1	4	1
99	99	99	99	99	99	99
3	0	2	3	2	3	2
0	0	3	2	4	1	1
3	0	2	3	2	3	1
1	1	2	4	1	4	1
1	1	2	4	1	4	1
3	0	2	3	2	2	1
3	0	1	4	1	4	2
3	0	3	4	1	4	1
3	0	1	1	1	1	2
3	0	99	3	2	3	2
3	0	1	1	1	1	1
3	0	2	3	2	3	2
3	0	3	3	4	3	2
2	0	1	4	1	4	1
3	0	1	4	1	4	1
3	0	1	4	1	4	1
3	0	1	3	1	1	1
3	0	1	4	1	4	1
3	0	2	4	1	4	2
3	0	1	4	1	4	2
3	0	1	4	1	2	2
3	0	3	4	1	4	1
3	0	1	4	1	4	1
3	0	1	4	1	4	1
3	0	2	4	2	3	1
3	0	2	4	2	4	2
3	0	1	4	1	4	2
3	0	1	3	1	2	2
3	0	1	4	1	4	2
3	0	2	3	2	4	1
3	0	2	3	2	3	2
3	0	1	1	1	1	1
3	0	2	3	2	1	1
3	0	1	1	1	1	1

0	2	2	2	2	2	99
3	0	1	2	2	1	1
3	0	1	3	1	1	2
2	2	1	4	1	4	2
2	0	2	3	2	3	2
3	2	2	3	1	3	2
3	0	2	4	1	4	2
1	0	4	3	1	4	1
3	0	4	2	1	4	1
3	0	2	3	2	3	1
3	0	3	3	3	3	2
3	0	1	99	99	4	1
3	0	99	99	1	4	1
3	0	4	4	1	4	1
3	0	4	4	1	4	1
3	0	3	3	1	3	3
3	0	2	3	2	3	3
3	0	4	4	1	3	1
3	0	2	4	1	4	99
99	99	99	99	99	99	99
3	0	3	3	2	3	2
3	0	1	4	1	3	1
3	0	2	3	2	3	1
3	0	1	4	1	4	1
3	0	2	3	2	3	2
3	0	1	4	1	4	1
3	0	2	3	1	4	2
3	0	2	4	1	3	1
3	0	1	4	1	4	1
3	0	1	4	1	4	1
3	0	3	2	2	3	2
3	0	1	4	1	3	2
3	0	1	4	1	4	1
3	0	1	4	1	4	1
2	2	2	3	2	3	3
3	0	1	4	1	3	1
3	0	2	4	1	4	1
3	0	2	3	1	4	1
3	0	2	4	2	4	1
2	0	1	4	1	4	1
3	0	2	3	1	1	1
3	99	3	3	2	3	99
3	0	3	3	2	2	2
3	0	3	3	2	3	2
3	0	4	4	1	4	1
3	0	3	3	1	3	1
99	99	2	3	2	3	2
3	0	1	4	1	4	1

3	0	1	4	1	4	1
3	0	2	3	2	2	2
3	0	1	1	1	2	2
3	0	2	3	2	3	3
3	0	1	4	1	4	1
2	2	2	1	3	1	3
3	0	99	4	2	4	1
3	0	2	3	1	3	2
3	0	1	4	1	4	1
3	0	1	4	1	4	2
0	0	1	3	1	4	1
3	0	1	4	1	1	1
3	0	2	3	2	4	1
3	0	1	4	2	4	2
3	0	1	4	1	4	1
3	0	1	3	2	3	2
3	0	1	4	1	4	2
3	0	2	3	2	3	1
3	0	1	4	1	4	1
3	0	1	4	1	4	1
3	0	99	4	1	4	1
3	0	1	4	1	4	1
2	2	4	4	1	4	3
3	0	2	3	2	3	2
3	0	3	4	1	4	2
3	0	2	3	2	3	2
3	0	99	4	1	4	1
3	0	3	3	2	3	2
3	0	1	4	1	4	1
3	0	2	3	2	3	1
3	0	2	4	2	4	1
3	0	1	4	1	4	1
3	0	1	4	1	4	1
3	0	2	3	1	4	1
3	0	1	4	1	4	1
0	1	3	4	1	4	2
3	0	1	4	1	4	1
3	0	4	4	1	4	1
0	0	1	4	1	4	1
0	0	4	1	4	1	4
3	0	1	4	1	4	1
0	1	1	1	4	1	4
3	0	1	4	1	4	1
3	0	1	4	1	4	1
3	0	1	4	1	4	1
3	0	2	4	1	3	1
3	0	1	4	1	4	1
3	0	2	1	1	3	2

3	0	1	4	1	4	1
3	0	1	4	1	4	1
3	0	1	4	1	4	99
3	0	1	4	1	4	1
3	0	1	4	1	1	1
3	0	1	4	1	4	1
3	2	99	4	1	1	1
3	0	1	4	1	4	2
3	0	2	2	1	2	1
3	0	2	3	3	3	3
3	0	2	3	2	3	3
3	0	1	3	2	3	99
3	0	1	4	1	4	1
3	0	1	99	1	1	2
0	99	1	4	1	1	1
99	99	99	99	99	99	2
3	0	4	4	1	4	1
3	0	1	99	1	1	1
3	0	1	4	1	4	1
3	0	1	4	1	4	1
3	0	1	4	1	4	1
3	0	1	4	1	4	1
3	0	1	4	1	4	2
3	0	1	4	1	4	1
3	0	1	4	1	4	1
3	0	2	4	2	4	2
3	0	2	3	2	3	2
3	2	1	3	2	3	2
3	0	1	4	1	4	1
3	0	1	4	1	4	1
3	0	2	3	2	3	2
3	0	2	4	1	3	3
3	0	1	4	1	1	2
3	0	3	4	1	3	1
3	0	2	3	2	3	2
3	0	3	3	2	3	2
99	99	99	99	99	99	99
3	0	2	3	2	3	2
3	0	3	3	1	3	1
3	0	4	4	1	4	1
3	0	1	4	1	4	1
3	0	4	4	1	4	2
3	0	2	4	1	3	1
3	0	1	4	1	4	1
3	0	3	4	1	4	1
3	0	2	3	1	4	1
3	0	1	4	1	4	1
3	0	2	3	2	3	3

3	0	1	4	1	4	1
0	0	1	99	1	4	1
3	0	2	3	2	3	1
3	0	1	4	1	4	1
3	0	4	3	1	3	1
3	0	1	4	2	3	2
0	0	99	3	2	3	2
3	0	4	4	1	4	4
3	0	1	3	1	3	1
3	0	1	4	1	1	1
3	0	1	4	1	4	1
3	0	2	3	1	3	2
2	0	1	4	1	4	1
3	0	2	4	2	3	1
3	0	1	4	1	4	1
3	0	1	4	1	4	1
3	0	1	4	1	4	1
3	0	1	4	1	4	1
2	2	2	3	1	3	4
3	0	2	3	2	3	2
3	0	99	99	99	99	99
3	0	1	4	1	4	1
3	0	3	3	2	3	2
3	0	4	4	1	4	1
0	1	4	1	4	1	4
0	0	1	4	1	4	1
3	0	1	4	1	4	1
3	0	1	4	1	4	1
3	0	1	4	1	4	1
3	0	1	4	1	4	1
2	2	2	3	2	3	2
2	2	2	4	1	4	1
3	0	4	4	1	4	1
3	0	2	4	1	4	3
3	0	1	4	1	4	1
3	0	2	4	1	4	1
99	99	3	4	1	4	1
3	0	1	4	1	4	1
3	0	1	1	1	1	1
3	0	1	1	1	1	1
3	0	1	1	1	4	1
0	2	1	4	1	4	2
3	0	1	4	4	4	1
3	0	2	4	1	1	1
3	0	1	4	1	1	1
2	2	1	4	1	4	1
3	0	2	4	2	4	2
3	0	1	4	1	4	1

3	0	2	4	1	4	1
3	0	2	4	2	4	2
3	0	2	3	2	3	3
3	0	2	4	1	4	2
0	0	2	2	2	2	2
3	0	1	4	1	4	1
2	2	3	3	2	3	3
2	2	99	4	1	4	1
3	0	1	4	1	1	1
3	0	3	2	2	3	3
3	0	1	4	1	4	1
3	0	1	4	1	4	1
3	0	1	4	1	4	1
3	0	1	4	1	4	2
3	2	2	4	1	4	2
3	0	1	4	1	4	1
3	0	1	1	1	1	1
3	0	1	4	1	4	1
3	0	2	3	1	4	1
3	0	4	1	1	4	1
3	0	1	4	1	3	1
3	0	1	4	1	3	2
99	99	2	3	2	2	2
3	0	4	4	99	4	2
3	0	1	4	1	1	1
3	0	4	4	4	4	3
3	0	1	4	1	4	1
3	0	1	4	1	1	1
3	0	1	3	1	1	1
3	0	1	4	1	4	1
3	0	1	4	1	99	1
3	0	2	3	2	3	2
3	0	99	4	1	4	1
3	0	1	4	1	4	1
2	0	2	2	1	4	1
3	0	1	4	1	4	1
0	0	1	3	1	4	1
3	0	1	1	1	4	1
3	0	1	4	1	4	1
3	0	2	3	1	3	2
3	0	1	4	1	4	1
3	0	1	4	1	4	1
3	0	2	4	1	4	1
3	0	1	4	1	4	1
3	0	1	3	2	3	1
3	0	1	4	1	4	1
3	0	2	4	2	4	2
3	0	1	4	1	4	1

3	0	1	4	1	4	1
3	0	1	4	1	4	1
3	0	3	3	2	3	3
3	0	4	4	99	4	1
3	0	1	4	1	4	1
3	0	99	99	99	99	99
3	0	4	4	1	4	1
3	0	1	4	2	3	4
3	0	1	4	1	4	1
3	0	3	4	2	3	2
3	0	1	4	1	4	1
3	0	1	4	1	3	2
3	0	1	4	1	4	1
2	2	2	3	2	3	2
3	0	1	4	1	4	1
3	0	3	3	2	3	2
3	0	2	3	2	2	2
3	0	2	3	2	3	2
3	0	1	4	1	4	1
3	0	3	4	99	3	2
3	0	1	4	1	4	1
3	0	4	4	2	4	2
3	0	1	4	1	4	1
3	0	1	4	1	4	1
3	0	1	4	1	1	1
3	0	2	2	2	2	2
2	2	3	4	1	4	3
3	0	3	3	3	3	3
3	0	1	4	1	4	1
3	0	1	4	1	4	1
3	0	1	4	1	4	1
3	0	1	4	1	4	1
3	0	1	4	1	4	1
3	0	99	4	1	4	1
3	0	4	4	1	4	1
3	0	1	4	1	4	1
99	99	99	99	99	99	99
3	0	1	4	1	4	1
3	0	99	4	1	4	1
3	0	2	4	4	4	99
3	0	2	3	2	3	2
3	0	1	4	2	4	1
3	0	2	3	1	4	2
2	2	99	1	1	1	1
3	0	2	3	2	3	3
3	0	1	4	1	1	1
3	0	1	2	99	99	2
3	0	3	4	2	3	3

3	0	3	4	1	4	99
3	0	99	1	1	4	1
3	0	1	1	1	1	1
3	0	99	3	2	3	99
99	2	99	4	99	99	2
3	0	1	4	1	4	99
2	2	4	4	4	4	4
3	0	3	4	1	3	2
3	0	2	3	2	3	99
3	0	1	1	1	1	1
3	0	3	3	99	99	3
3	0	1	4	1	4	1
3	0	4	4	1	4	1
3	0	99	99	99	4	1
3	2	3	4	1	4	2
3	0	1	4	1	4	1
3	0	1	4	1	4	1
3	0	99	4	1	4	1
3	0	2	3	1	1	1
3	0	4	4	1	1	1
3	0	1	4	1	4	1
3	0	2	3	1	3	1
3	0	99	4	1	4	1
3	0	99	99	99	99	99
3	0	3	4	2	4	1
3	0	2	3	2	3	2
3	0	1	4	1	4	1
3	0	2	3	2	4	1
3	0	2	4	1	4	4
0	0	2	4	1	4	4
3	0	1	4	1	4	1
3	0	1	4	1	4	1
3	0	2	4	1	4	3
3	0	1	3	2	4	2
3	0	2	3	2	3	2
3	0	1	4	1	4	1
0	0	99	4	2	4	1
99	99	99	99	99	99	99
3	0	1	4	1	4	99
3	0	1	4	1	4	1
3	0	1	4	1	4	1
3	0	4	4	1	4	99
3	0	1	4	1	1	1
3	0	1	3	1	4	1
3	0	1	4	1	4	1
3	0	1	4	1	4	1
3	0	1	4	2	4	1
3	0	1	4	1	4	1

99	99	1	4	99	99	99
1	0	3	3	3	3	3
3	0	99	4	1	4	1
3	0	1	3	1	4	1
3	0	1	1	1	4	1
3	0	2	99	2	3	2
3	0	1	4	1	4	1
3	0	99	3	2	3	2
3	0	99	3	2	3	2
3	0	1	1	1	4	1
3	0	1	4	1	4	1
3	0	1	4	1	4	1
3	0	1	1	1	4	1
3	0	1	4	1	4	1
3	0	2	3	1	3	2
3	0	1	4	1	4	1
3	0	1	4	1	4	1
3	0	1	4	1	4	1
3	0	4	4	1	4	2
2	2	1	4	1	4	1
3	0	1	4	1	4	1
2	2	3	4	2	4	1
99	99	99	99	99	99	3
3	0	4	4	2	3	2
3	0	1	4	1	4	1
3	0	4	4	1	4	1
3	0	1	4	1	4	1
3	0	2	3	2	3	2
3	2	2	3	2	3	2
3	0	4	99	99	4	1
3	0	2	4	1	4	1
3	0	1	4	1	4	1
3	0	4	4	1	1	1
3	0	4	4	1	4	1
3	0	1	1	1	4	1
3	0	2	4	2	4	2
3	0	2	4	1	4	2
3	0	2	3	2	1	2
3	0	1	4	1	4	1
3	0	4	4	1	4	1
3	0	1	4	1	4	1
3	0	1	1	1	4	1
3	0	1	4	1	4	1
3	0	1	4	1	4	1
3	0	4	4	1	4	1
3	0	4	4	4	4	4
3	0	99	2	2	99	2
3	0	1	4	2	3	1

2	0	3	4	2	4	2
1	1	1	1	1	1	1
2	0	2	2	2	3	2
2	2	1	4	1	4	1
3	0	1	4	1	4	1
3	0	1	4	1	4	4
3	0	1	4	1	4	1
3	0	4	4	1	4	1
3	0	99	4	4	4	4
3	0	1	4	1	4	1
3	0	1	4	1	4	1
3	0	1	1	1	1	99
3	0	1	4	1	4	1
3	0	2	2	2	3	2
3	0	2	3	1	3	2
3	0	99	4	1	4	1
3	0	2	3	2	3	2
3	0	99	4	1	4	1
3	0	1	99	1	1	1
3	0	1	4	1	4	1
3	2	99	4	1	4	1
3	0	1	4	1	4	1
3	0	99	4	1	4	1
3	0	99	3	1	4	99
99	2	2	3	2	4	2
3	2	1	4	1	4	1
3	0	1	4	1	4	1
3	0	1	4	1	4	1
3	0	1	4	2	3	1
3	0	3	4	2	3	99
3	0	2	4	1	4	1
3	0	1	2	2	3	99
3	0	1	4	1	4	1
3	0	1	4	1	4	1
3	0	1	4	1	4	1
3	0	1	4	1	4	1
3	0	2	4	1	4	1
3	0	1	4	2	4	1
2	2	99	99	99	99	99
3	0	1	4	1	4	1
3	0	99	4	1	4	1
3	0	1	4	1	4	1
3	0	99	99	99	99	99
3	0	3	4	2	3	3
0	0	4	4	4	4	4
3	0	2	4	1	4	1
3	0	1	4	1	4	1
3	0	1	4	1	1	1

3	0	2	3	2	3	2
99	99	2	3	2	3	3
3	0	1	4	1	4	1
3	0	1	4	1	4	1
3	0	1	4	1	4	1
3	0	1	4	1	4	1
3	0	1	4	1	4	1
3	0	1	4	1	4	1
3	0	99	3	2	3	2
3	0	2	4	1	4	1
3	0	1	1	2	3	2
3	0	4	3	3	3	3
3	0	2	4	1	1	1
3	0	2	4	1	1	1
3	0	2	3	2	3	2
3	0	2	3	3	2	2
3	0	1	4	1	4	1
3	0	2	3	1	3	1
3	0	1	4	2	3	1
3	0	2	3	2	3	2
0	1	99	4	1	4	1
3	0	1	4	1	4	1
3	0	2	4	1	4	2
3	0	3	3	1	3	1
3	0	1	4	1	4	1
99	99	3	3	2	3	2
3	0	99	4	1	4	1
3	0	4	4	1	4	1
3	0	1	4	1	4	1
2	2	99	4	1	4	1
3	0	3	4	1	4	1
3	0	2	4	1	4	1
3	0	1	4	1	4	1
3	0	2	3	2	3	2
2	2	99	99	99	4	1
3	0	99	99	99	99	99
3	0	1	1	1	3	2
3	0	4	4	1	4	1
2	2	2	3	2	3	2
3	0	3	4	1	3	3
3	0	3	4	1	4	1
3	0	1	4	1	4	1
3	0	1	4	1	4	1
3	0	1	4	1	4	1
3	0	1	4	1	4	1
3	0	1	4	1	4	1
3	0	1	4	1	4	2
3	0	2	4	1	4	1

3	0	4	4	1	4	1
3	0	2	3	1	4	2
3	0	1	4	1	4	1
0	0	1	99	99	1	1
3	0	2	3	1	3	1
3	0	1	4	1	4	1
3	0	3	3	1	3	2
3	0	1	4	1	4	1
3	0	3	4	1	4	1
0	0	3	4	1	3	2
3	0	3	3	2	3	2
3	0	3	3	2	3	2
3	0	2	4	1	4	1
3	0	4	3	1	3	1
3	0	99	4	2	2	2
3	0	99	4	1	4	99
3	0	2	3	2	3	2
3	0	99	4	1	1	1
3	0	2	4	99	1	2
3	0	1	1	1	1	1
3	0	2	4	1	4	1
3	0	2	3	2	3	2
3	0	1	1	1	1	1
3	0	1	4	1	4	1
3	0	2	4	1	4	1
3	0	4	3	2	3	2
3	0	3	3	1	1	1
1	1	2	3	4	4	1
3	0	1	4	1	4	1
3	0	1	4	1	4	1
3	0	1	4	1	4	1
3	0	1	4	1	4	1
3	0	1	4	1	4	1
3	0	2	3	2	3	2
3	0	1	4	1	4	1
3	0	4	4	2	4	2
2	2	4	4	1	4	1
3	0	2	3	1	4	1
3	0	1	4	1	4	1
3	0	1	4	1	4	1
3	0	2	4	2	4	1
3	0	1	3	1	4	1
3	0	1	4	1	4	1
3	0	2	4	1	3	1
3	0	2	3	2	3	2
1	0	1	4	1	4	1
3	0	1	4	1	4	1
3	0	2	4	2	3	1

3	0	1	4	1	4	1
3	0	1	1	99	1	1
3	0	2	4	1	1	1
3	0	1	4	1	4	1
3	0	1	4	1	4	1
3	0	1	4	1	4	1
3	0	1	4	1	4	1
3	0	1	4	1	4	1
3	0	1	4	1	4	1
3	0	4	4	1	4	1
3	0	2	4	1	4	4
2	2	3	2	3	4	3
3	0	1	4	1	4	1
3	0	1	4	1	4	1
3	0	2	3	1	4	1
3	0	1	4	1	4	1
3	0	3	4	2	4	1
2	1	2	3	2	3	2
99	99	99	99	99	99	99
3	0	99	99	99	99	99
3	0	4	4	1	4	1
3	2	2	3	2	4	1
1	0	2	3	1	4	1
3	0	1	4	1	4	4
3	0	99	99	1	4	99
3	0	1	3	1	3	1
3	0	2	3	2	3	2
2	2	1	3	1	3	1
3	0	2	3	2	3	2
0	2	2	2	2	2	2
3	0	99	99	99	99	99
3	0	2	3	1	3	3
3	0	1	1	1	1	1
3	0	99	4	1	4	1
3	0	1	4	1	4	1
3	0	1	4	1	1	1
3	0	1	4	1	1	4
0	0	3	4	1	4	1
0	0	1	1	1	1	1
3	0	2	4	1	4	1
3	0	1	4	1	4	1
3	0	4	99	99	99	99
3	0	1	4	1	4	1
3	0	1	4	1	4	2
3	2	4	4	1	4	1
3	0	4	4	1	4	1
3	0	1	4	1	4	1
1	1	4	3	3	3	3

3	0	1	4	1	4	1
99	0	2	2	2	2	3
3	0	2	4	1	4	1
3	0	1	1	1	4	1
3	0	2	3	2	3	3
0	0	99	99	99	99	99
3	0	1	3	2	3	2
3	0	1	4	1	4	1
3	0	1	4	1	4	1
3	0	99	4	1	4	4
3	0	4	4	4	4	1
3	0	1	4	1	4	1
3	0	3	3	1	3	4
3	0	2	4	1	4	1
0	0	1	1	1	2	2
3	0	1	4	1	4	1
3	0	1	4	1	4	1
3	0	3	4	1	1	1
3	0	4	4	1	4	1
3	0	1	4	1	4	1
3	0	1	4	1	4	2
3	0	2	3	2	3	3
2	2	2	4	2	3	3
1	1	1	1	1	1	1
3	0	1	4	1	4	1
2	2	2	3	2	3	2
3	0	99	4	1	4	1
3	0	3	4	1	4	2
3	0	3	2	2	2	2
3	0	1	4	1	4	1
3	1	1	4	1	4	1
3	0	2	4	1	4	1
3	0	4	4	1	4	1
3	0	1	4	1	4	1
3	0	1	4	1	4	1
3	0	99	3	2	3	2
3	0	2	4	1	4	1
3	0	2	2	2	3	2
3	0	2	4	4	4	1
3	0	2	4	1	4	3
2	2	1	4	1	4	1
2	2	1	3	2	3	3
2	2	2	3	3	3	2
3	0	1	4	1	4	4
3	0	1	4	1	4	1
3	0	1	4	1	4	1
3	0	2	3	2	3	2
3	0	2	4	2	3	2

3	0	1	4	1	4	1
3	0	99	4	1	4	4
3	0	1	4	1	3	1
3	0	1	4	1	4	1
3	0	1	4	1	4	1
3	0	4	4	1	1	1
3	0	3	3	3	99	1
3	0	2	3	2	3	1
3	0	1	4	1	4	1
3	0	3	99	99	4	99
3	2	2	3	2	3	2
3	0	1	4	1	4	1
3	0	3	4	1	4	2
2	0	4	4	4	4	4
0	0	3	4	1	4	4
3	0	1	4	1	4	1
3	0	2	2	2	2	2
3	0	99	3	2	4	3
99	0	2	2	2	2	2
3	0	1	4	2	1	1
3	0	3	4	1	4	3
3	0	1	4	1	4	1
3	0	1	4	1	4	1
0	0	1	2	3	1	1
3	0	2	3	2	3	1
3	0	1	2	1	2	1
2	0	2	3	1	4	2
0	0	99	2	99	99	2
3	0	1	4	1	4	1
3	0	1	4	1	4	1
3	0	1	4	1	4	1
3	0	3	4	1	4	2
3	0	2	4	2	3	4
3	0	1	4	1	1	1
3	0	2	3	2	4	1
3	0	1	4	1	4	1
3	0	1	4	1	4	1
3	0	2	3	2	3	1
3	0	2	3	2	2	2
3	0	1	4	1	4	1
3	0	2	3	2	3	2
3	0	1	4	1	4	1
3	0	1	1	1	4	1
3	2	3	3	2	3	1
3	0	99	4	1	4	1
3	0	2	3	2	3	2
3	0	1	3	1	4	1
3	0	3	3	2	3	2

3	0	99	3	99	3	2
3	0	1	4	1	4	1
3	0	1	99	1	4	1
2	2	99	99	99	99	99
3	0	1	4	1	3	2
3	0	2	3	2	3	2
3	0	1	4	1	4	1
3	0	1	4	1	4	1
3	0	1	4	1	4	1
3	0	2	4	1	3	1
3	0	2	4	1	4	2
3	0	2	3	2	3	2
3	0	1	4	1	1	1
2	2	4	1	1	4	1
1	0	1	4	1	4	1
3	0	1	1	1	1	1
3	0	1	4	1	4	2
3	0	1	4	2	3	3
0	0	1	4	2	4	2
3	0	1	4	1	4	1
3	1	1	4	1	1	1
3	0	1	4	1	4	1
99	0	1	4	1	4	1
3	0	1	4	1	4	1
3	0	1	3	2	3	2
3	0	1	3	2	1	2
1	0	4	4	4	4	1
3	0	2	3	3	2	3
3	0	1	4	1	4	1
3	0	1	4	1	4	1
3	0	1	4	1	1	1
3	0	1	4	1	4	1
3	0	2	4	1	4	1
3	0	1	4	1	4	1
3	0	1	4	1	4	1
3	0	1	4	2	4	2
3	0	2	2	2	2	2
3	0	1	4	1	4	1
3	0	1	4	1	4	1
1	1	3	2	2	2	1
3	0	1	1	1	1	1
3	0	1	1	1	4	1
3	0	1	99	1	1	1
3	0	2	3	2	3	3
3	0	1	4	2	2	2
3	0	99	99	99	99	99
3	0	2	2	2	99	3
2	0	1	4	1	4	1

3	0	1	4	1	4	1
1	0	4	4	1	1	1
3	0	3	3	2	2	2
3	0	1	4	1	4	1
3	0	1	3	1	4	1
3	0	3	2	2	3	3
3	0	99	4	1	99	99
3	0	3	3	3	3	3
3	0	4	4	1	4	1
3	0	4	4	1	4	1
2	2	2	99	99	99	99
3	0	4	1	2	3	2
3	0	4	4	3	3	3
3	0	2	4	2	2	2
3	0	4	4	1	4	1
3	0	3	3	2	4	2
3	0	2	3	2	3	1
3	0	1	4	1	4	1
3	0	2	4	1	4	1
3	0	2	3	2	3	2
3	0	4	4	1	4	1
3	0	3	4	1	4	3
3	0	2	4	1	4	1
3	0	1	4	1	1	2
3	0	1	4	1	4	1
3	0	3	3	3	3	2
3	0	4	4	1	4	1
3	0	1	4	1	4	1
3	0	3	3	2	3	3
3	0	1	4	1	4	1
3	0	1	2	1	3	1
3	0	2	4	1	3	2
3	0	1	4	1	4	2
3	0	99	4	2	3	1
3	0	1	4	1	4	1
2	2	1	4	1	4	1
3	0	1	1	1	1	1
3	0	2	2	2	2	2
3	0	1	4	1	4	1
3	0	1	4	1	4	1
3	0	2	3	2	1	99
3	0	2	3	3	3	1
3	0	2	2	3	2	1
3	0	4	4	4	4	2
3	0	1	4	1	4	1
3	0	3	4	1	2	3
99	99	4	4	1	4	1
3	0	2	4	2	4	2

3	0	1	4	1	4	1
3	0	1	4	1	4	1
3	0	1	4	1	4	1
3	0	2	3	2	3	2
3	0	1	2	3	2	1
3	0	2	3	2	3	3
3	0	99	4	1	4	1
3	0	1	4	1	4	1
3	0	1	4	1	4	1
3	0	1	4	1	4	1
3	0	1	4	1	4	1
3	0	1	4	1	4	1
3	0	1	4	1	4	1
3	0	2	3	2	3	2
3	0	1	4	1	4	2
3	0	99	3	2	3	2
3	0	1	4	1	4	1
3	0	2	3	2	3	2
3	0	2	3	3	3	1
3	0	1	4	1	4	1
2	2	1	1	1	1	1
3	0	2	3	2	3	2
3	0	2	4	2	4	2
3	0	1	4	1	4	1
3	0	1	4	1	4	1
3	0	1	4	1	4	1
3	0	1	4	1	4	1
3	0	99	4	2	4	2
2	2	1	1	1	4	1
3	0	1	4	1	4	1
3	0	1	4	1	1	3
3	0	3	4	1	3	4
3	0	2	4	1	4	3
3	0	1	4	1	4	1
3	0	3	4	1	4	1
3	0	1	4	1	4	1
3	0	2	3	2	2	2
3	0	1	4	1	4	1
3	2	1	1	1	1	1
3	0	1	4	1	4	1
3	0	1	4	1	4	4
3	0	99	1	1	1	99
3	0	2	3	1	4	2
2	0	1	4	1	1	2
3	0	2	3	2	3	2
3	0	4	4	1	4	3
3	0	1	4	1	4	1
3	0	99	4	2	3	2

3	0	1	1	1	4	1
3	0	1	3	2	3	1
3	0	1	4	1	4	2
3	0	1	4	1	4	1
3	0	4	4	1	4	1
3	0	1	4	1	1	1
3	0	2	3	2	3	2
3	0	3	3	2	3	2
3	0	1	4	1	4	2
3	0	1	4	1	4	2
3	0	1	99	99	99	99
3	0	1	4	1	4	1
3	0	1	4	1	4	1
3	0	4	4	4	4	4
3	0	1	4	1	4	1
3	0	2	3	2	3	2
2	2	2	4	2	3	2
3	0	2	2	2	3	2
0	0	3	3	2	1	2
3	0	1	1	1	1	1
3	0	1	4	1	4	1
3	0	1	4	1	4	1
3	0	3	3	2	1	3
3	0	1	4	1	4	1
3	0	1	3	1	3	1
3	0	1	4	1	4	1
3	0	4	4	1	4	1
3	0	1	4	1	4	1
3	0	2	3	2	3	1
3	0	2	3	2	3	2
3	0	1	3	1	3	2
3	0	1	3	2	3	2
3	0	1	1	1	1	1
1	0	1	4	1	4	1
3	0	3	4	1	4	2
3	0	1	4	1	4	1
3	0	4	4	1	1	2
3	0	2	3	1	4	1
3	0	1	4	1	4	1
0	0	1	4	1	4	1
3	0	2	4	1	4	2
3	0	1	4	1	4	1
2	2	1	4	1	4	1
3	0	1	4	1	4	1
3	0	1	4	2	4	2
3	0	1	4	1	4	1
3	0	4	4	1	1	1
3	0	1	4	1	4	1

3	0	1	4	1	4	1
3	0	3	3	2	1	2
3	0	1	4	1	4	1
3	0	1	4	1	4	1
3	0	1	4	1	4	1
3	0	1	4	2	3	2
3	0	1	1	1	1	1
3	0	1	4	1	4	1
3	0	1	4	1	4	1
3	0	1	4	1	4	1
3	0	1	4	1	4	1
3	0	1	4	2	4	1
3	0	1	4	1	4	2
3	0	1	4	1	4	1
3	0	1	4	1	4	1
3	0	2	4	1	1	1
3	0	1	4	1	4	1
3	0	1	4	1	4	2
3	0	1	4	1	4	1
3	0	1	4	1	4	1
3	0	1	4	1	4	1
3	0	1	4	1	1	1
3	0	1	1	1	1	1
0	0	1	1	1	1	1
3	0	1	4	1	4	1
3	0	1	4	1	4	1
3	0	1	4	1	4	1
0	0	1	4	1	4	1
3	0	3	3	2	3	2
#NULL!	#NULL!	2	2	2	3	3
3	0	2	4	1	4	1
1	1	3	3	1	3	1
3	1	1	1	1	1	1
0	0	1	4	1	4	1
3	0	2	4	1	4	1
3	0	1	4	1	4	1
3	0	2	4	2	4	2
3	0	1	4	1	4	1
2	0	2	3	2	4	1
3	0	4	3	3	3	4
3	0	4	4	4	4	4
3	0	4	4	2	4	2
3	0	1	4	1	4	1
0	0	3	3	2	3	3
3	0	1	4	1	4	1
3	0	1	4	1	4	1
3	0	1	4	1	4	1
3	0	2	4	1	4	1

2	2	1	4	1	4	1
3	0	1	4	1	4	1
3	0	2	3	2	3	2
3	0	1	4	1	4	1
0	0	2	4	1	4	2
3	0	1	4	1	4	1
2	2	1	4	1	4	1
0	0	3	1	1	1	1
3	0	4	4	4	4	3
3	0	2	4	1	4	4
0	0	2	2	2	2	2
#NULL!	2	4	4	4	4	4
#NULL!	#NULL!	#NULL!	#NULL!	#NULL!	#NULL!	#NULL!
3	0	2	4	1	4	1
2	2	2	1	1	1	2
3	0	1	4	1	4	1
3	0	1	4	1	4	1
0	0	1	4	1	4	3
3	0	1	4	1	4	1
3	0	#NULL!	3	2	4	3
3	0	3	3	2	3	2
0	0	2	4	1	4	3
2	2	2	2	2	2	2
0	0	2	3	2	3	2
2	2	1	4	1	4	1
2	0	1	4	1	4	1
3	0	1	4	1	4	1
3	0	2	4	2	2	1
0	0	2	2	2	2	2
2	2	4	4	4	4	2
3	0	1	1	1	1	1
0	0	1	4	1	4	1
2	0	1	4	1	4	1
2	2	1	4	1	4	1
3	0	4	4	1	1	1
0	0	1	4	1	1	1
3	0	1	1	1	1	1
0	0	4	4	2	4	2
#NULL!	0	4	4	2	#NULL!	2
2	2	2	4	1	4	2
3	0	2	4	1	3	1
3	0	4	4	1	4	1
3	0	4	4	2	4	2
3	0	2	3	2	3	2
3	0	2	3	2	3	2
3	0	1	4	1	4	1
3	0	1	4	1	4	1
3	0	4	1	4	2	1

3	0	2	4	1	1	1
3	0	2	1	1	2	2
3	0	1	4	1	4	1
0	0	1	1	1	1	1
3	0	1	4	1	1	1
3	0	1	1	1	1	1
3	0	1	4	1	4	1
3	0	1	1	1	1	1
3	0	1	4	1	1	1
3	0	1	4	1	4	1
0	0	1	1	1	4	1
3	0	1	4	1	4	1
3	0	1	4	1	4	1
3	0	1	4	1	4	1
3	0	1	4	1	4	1
3	0	4	4	1	4	1
#NULL!	#NULL!	#NULL!	#NULL!	#NULL!	#NULL!	#NULL!
3	0	1	4	1	4	1
0	0	1	4	1	4	2
3	0	1	1	1	4	1
3	0	4	4	1	4	1

abstaina_pre	abstainb_pre	abstainc_pre	abstaind_pre	abstaine_pre	prega_pre	pregb_pre	pregc_pre
99	99	99	99	99	99	99	99
2	99	4	2	1	3	3	3
1	1	1	3	1	4	4	4
3	2	3	2	3	3	4	2
2	3	4	3	1	2	3	1
99	99	99	99	99	99	99	99
4	4	1	4	4	4	4	4
2	2	2	2	2	2	2	3
99	99	99	99	99	99	99	99
2	2	3	3	1	2	3	2
3	3	2	1	1	2	1	3
3	3	3	2	1	2	2	2
3	1	3	2	1	1	1	2
2	1	1	1	2	1	2	1
1	1	1	1	1	1	1	1
3	2	3	2	1	3	3	2
99	99	99	99	99	99	99	99
3	2	2	3	2	2	2	3
3	2	99	99	1	2	3	3
99	2	3	3	2	2	2	2
99	99	99	99	99	99	99	99
1	99	99	99	99	1	1	1
99	99	99	99	99	99	99	99
2	2	3	3	4	4	4	4
99	1	1	1	1	4	3	99
99	1	4	2	1	2	4	3
99	99	2	99	1	2	2	3
4	4	4	1	1	4	4	4
3	3	3	2	1	4	4	3
4	1	4	1	1	3	4	4
3	3	2	3	1	3	3	2
2	2	2	3	1	1	2	1
3	2	2	2	1	2	2	3
4	4	4	1	1	3	3	1
99	99	99	99	3	99	99	99
3	2	4	2	1	3	4	3
3	3	3	3	2	3	3	3
3	2	2	2	2	3	4	3
2	1	2	3	1	3	3	3
2	3	2	3	2	3	3	3
4	1	1	1	1	4	4	3
2	99	99	1	1	99	99	99
1	1	4	2	1	4	3	4
3	2	3	2	1	3	3	3
3	1	4	1	1	1	4	4
4	1	2	1	1	4	4	3
1	3	2	3	4	1	2	1

1	4	3	4	4	3	4	3
4	1	4	1	1	4	4	4
2	3	2	1	1	4	4	4
3	3	3	2	2	2	3	3
4	1	3	1	1	3	3	3
2	3	3	2	2	3	3	3
4	1	4	1	1	3	4	3
99	3	3	3	2	1	1	3
99	2	3	1	1	3	3	3
2	2	2	2	1	2	3	3
3	2	3	2	2	3	3	2
3	1	4	2	1	4	4	4
3	3	2	3	1	3	3	3
2	2	4	2	2	3	3	3
4	2	4	2	2	2	2	3
2	3	3	3	1	2	3	4
2	2	3	3	2	4	4	4
4	1	3	2	1	2	4	1
3	3	4	2	1	4	4	4
2	3	4	3	1	4	4	2
2	4	4	4	4	2	2	3
1	3	4	3	1	3	4	3
3	2	3	2	2	4	4	4
1	4	1	4	3	1	1	1
4	1	4	1	1	4	4	4
1	1	4	1	1	4	4	4
1	4	4	4	1	1	1	4
2	99	99	3	2	2	3	3
3	2	3	2	2	99	99	3
3	2	4	3	2	2	2	3
3	2	4	2	4	99	99	3
3	2	3	2	2	3	3	2
2	2	4	1	1	3	3	4
2	2	3	2	2	4	4	4
3	2	3	3	1	3	2	4
4	1	4	1	1	4	4	3
1	1	4	1	4	99	99	4
3	1	4	2	1	4	4	4
2	2	3	2	2	1	1	3
4	1	4	1	1	4	4	4
4	2	3	2	2	4	3	3
4	4	2	4	4	3	4	4
2	3	3	3	3	4	4	2
3	2	2	2	2	3	3	3
3	2	3	2	2	2	2	3
3	1	4	1	1	4	4	4
4	1	4	1	1	4	4	4
99	99	3	99	99	2	1	3

4	3	3	1	1	3	3	2
2	1	3	2	2	3	3	4
2	3	3	3	3	1	1	3
3	1	4	1	1	4	4	4
1	99	2	2	1	3	3	3
4	2	3	2	1	4	4	3
3	2	4	1	2	3	2	4
3	2	3	2	1	4	4	3
2	2	3	3	2	2	3	3
2	3	3	2	2	3	3	4
2	2	3	2	2	3	2	3
99	2	2	2	2	2	3	3
4	2	3	1	1	4	4	4
2	3	3	3	1	2	3	3
3	2	2	2	2	3	3	2
2	1	4	2	2	4	4	4
4	2	4	1	1	2	2	1
4	1	4	1	1	4	4	4
3	2	99	2	2	3	3	3
3	3	3	4	2	3	4	4
3	3	3	3	2	2	2	3
4	1	4	1	1	4	4	4
4	4	4	4	4	4	4	4
99	3	4	2	1	3	4	4
1	1	4	1	1	4	4	1
1	4	1	4	4	4	4	4
4	1	4	1	1	4	4	4
3	2	3	2	2	3	3	3
3	3	2	3	3	2	3	3
3	3	4	2	1	2	3	2
99	99	99	3	2	4	4	4
2	2	2	2	2	2	2	2
2	3	2	2	2	4	4	4
3	2	4	2	1	4	4	4
3	3	4	3	1	3	3	2
2	1	4	1	1	3	4	4
3	2	3	2	1	2	3	2
3	2	4	2	2	2	2	3
3	2	3	2	1	3	3	4
3	3	4	3	1	3	4	3
3	1	4	2	1	3	4	3
2	2	3	2	2	3	3	3
3	2	4	2	1	3	3	3
4	1	4	1	1	2	3	4
2	2	3	2	2	2	2	99
99	99	99	99	99	4	1	4
3	2	3	3	2	3	2	3
2	3	3	2	1	3	2	4

2	2	4	2	2	2	2	3
2	3	3	3	2	1	2	3
4	1	1	1	1	4	1	4
4	1	4	1	1	4	4	4
2	3	3	3	2	2	2	2
2	2	2	2	1	2	3	2
2	2	2	2	2	2	2	2
3	3	3	2	1	2	2	3
2	3	2	3	1	3	3	3
1	3	1	4	4	3	3	2
3	1	3	2	1	3	3	3
1	1	1	1	1	1	1	1
2	3	3	2	2	3	2	3
2	2	3	1	2	3	2	3
4	3	3	1	1	4	3	2
2	3	2	2	2	4	3	2
3	2	3	2	1	3	3	2
4	3	3	1	1	3	4	3
4	3	4	1	1	4	4	4
3	2	3	2	2	2	2	3
3	2	4	2	4	3	2	3
4	99	99	2	1	99	3	99
4	1	4	2	1	1	4	1
3	1	4	1	1	3	4	4
3	1	1	2	1	3	3	3
3	2	3	2	1	4	4	4
3	2	3	2	2	3	3	3
2	3	3	4	3	2	2	3
3	1	4	1	1	3	3	4
4	4	4	2	1	4	4	4
99	99	99	99	99	99	99	99
3	2	3	2	1	2	3	3
3	2	3	2	1	3	3	3
4	2	3	1	1	3	4	3
4	1	4	1	1	4	4	4
3	4	4	3	1	2	2	3
4	1	4	2	1	3	3	4
2	2	2	3	2	2	2	2
3	4	4	3	1	4	4	4
1	2	3	2	1	3	3	3
3	1	4	1	1	3	2	2
99	99	3	2	2	4	4	3
1	3	3	3	1	4	4	4
2	2	4	2	4	4	4	4
3	2	3	2	1	2	2	3
4	1	4	1	1	4	4	4
3	3	4	2	1	2	3	3
3	2	4	1	1	4	4	4

99	1	4	1	1	4	4	4
3	2	4	1	1	4	3	2
4	1	1	3	1	4	4	4
3	2	2	2	1	4	4	4
3	3	2	2	2	3	3	4
2	3	99	99	99	4	4	4
1	3	3	2	2	3	3	3
2	2	4	1	1	4	4	4
3	3	3	3	3	3	3	3
2	1	4	1	1	4	2	3
3	2	4	1	1	3	3	3
2	2	3	2	2	2	2	3
4	1	4	1	1	4	4	4
2	3	3	3	2	3	3	3
1	1	4	1	1	1	1	1
2	2	3	3	1	1	1	3
3	2	4	1	1	2	4	4
2	2	4	2	2	2	2	4
3	1	3	2	1	3	4	4
2	3	1	1	1	2	3	2
4	1	4	1	1	4	4	4
99	99	99	99	99	99	99	99
3	2	2	3	1	2	2	2
4	2	4	1	1	2	1	2
1	99	2	99	4	3	4	3
4	3	4	2	1	4	4	4
2	1	4	2	1	4	4	4
3	2	4	2	1	3	4	4
4	1	4	1	1	4	4	4
3	3	4	3	1	3	1	4
4	1	4	1	1	2	2	3
3	2	3	2	1	3	3	3
4	1	4	1	4	4	4	4
2	1	4	1	1	99	99	99
2	2	3	2	3	4	4	4
1	3	4	3	4	2	1	3
3	2	4	2	1	2	2	3
3	3	3	2	2	3	3	3
3	3	2	3	2	4	4	4
4	1	4	1	1	2	3	4
3	2	4	2	2	4	4	4
2	2	3	2	2	4	4	4
3	2	4	1	1	2	2	3
3	2	3	2	1	3	3	3
4	2	4	2	1	4	4	3
1	4	4	3	2	3	2	3
4	1	4	1	1	3	3	2
4	3	4	1	4	1	4	4

3	3	3	2	2	2	2	2
4	1	4	1	1	4	4	3
3	2	4	1	1	2	3	2
4	1	1	1	1	4	4	4
1	3	2	3	2	1	3	2
4	1	4	2	4	4	4	1
3	1	4	1	1	4	3	4
3	3	3	3	3	3	3	3
3	2	3	2	2	3	2	3
4	2	2	1	1	3	3	3
99	3	3	3	1	4	4	4
99	2	2	2	2	2	3	3
3	2	3	2	1	4	4	3
2	3	3	3	3	4	4	2
4	4	4	2	4	4	4	4
3	2	4	1	1	4	4	4
4	1	4	1	1	4	4	2
4	1	99	1	1	99	99	99
99	2	3	2	1	99	2	2
3	1	4	2	1	4	4	4
3	3	3	2	1	3	4	4
2	3	2	3	1	4	4	4
3	3	4	2	1	3	3	3
4	1	99	1	1	4	4	1
3	1	4	1	1	4	4	2
3	2	3	1	1	2	2	3
4	2	4	1	1	4	4	4
4	2	4	2	1	4	4	3
3	3	4	3	2	3	4	3
4	1	4	1	1	4	4	1
4	3	1	1	1	3	3	3
3	3	3	3	2	3	3	2
2	3	3	3	2	4	4	3
3	2	3	2	2	3	4	3
2	2	2	3	1	1	1	1
2	4	3	4	4	2	4	3
4	2	3	2	2	3	3	3
2	3	1	3	3	3	3	3
1	1	4	2	1	3	3	2
4	3	4	4	2	4	4	4
2	3	4	2	1	3	3	3
4	1	4	1	1	3	3	3
3	3	4	2	1	3	3	4
4	4	4	2	2	2	3	3
1	1	4	1	1	4	4	2
3	2	3	1	2	4	4	2
2	2	3	2	1	4	4	4
1	4	3	4	1	1	3	3

3	3	3	3	3	3	3	2
4	1	4	1	1	1	2	1
4	2	3	2	1	3	4	3
4	2	99	2	2	99	1	1
4	1	4	1	1	2	1	3
3	3	2	2	2	3	3	3
3	3	3	3	2	4	4	3
4	2	3	2	1	3	3	2
2	3	3	1	1	3	4	4
2	2	3	2	2	3	3	3
2	2	4	2	1	4	4	3
3	3	3	3	2	2	2	2
3	2	3	3	1	3	3	2
3	2	3	2	1	3	4	3
2	3	3	3	3	2	2	3
4	1	3	1	1	4	4	3
2	3	3	3	3	2	2	3
2	3	4	2	1	2	2	3
4	1	4	4	1	4	4	4
4	1	4	1	1	3	3	4
4	1	4	1	1	3	3	4
1	3	4	4	3	3	1	3
2	3	3	3	2	4	4	3
2	3	3	3	2	2	2	3
1	3	2	3	2	2	2	2
3	2	3	2	2	3	3	3
3	3	2	2	2	3	3	3
3	2	3	2	2	2	3	3
4	1	99	99	99	99	99	99
99	99	99	99	99	99	99	99
4	2	4	2	1	4	4	3
2	1	4	3	2	3	2	99
2	3	3	3	3	4	4	4
2	3	3	2	2	2	2	2
4	2	4	1	1	3	3	1
1	3	2	2	3	4	1	4
3	3	2	2	2	2	2	2
3	1	3	2	1	3	3	3
1	1	1	1	1	4	4	4
4	1	1	1	1	3	4	2
3	3	3	2	2	4	4	4
2	3	3	3	2	3	2	3
2	3	2	3	3	3	2	3
3	1	4	2	1	3	3	4
2	3	3	3	2	2	2	3
4	3	4	2	1	3	3	4
2	2	3	4	1	2	2	3
2	2	3	3	3	3	2	4

3	3	4	3	2	2	3	4
3	3	2	3	2	3	3	2
3	2	3	99	2	99	99	3
2	2	2	3	3	3	3	4
3	3	3	3	2	4	3	2
99	99	99	99	99	99	99	99
3	2	3	3	2	4	3	3
2	2	3	3	3	4	4	3
1	3	3	2	3	2	3	3
2	4	2	3	4	3	3	4
1	3	4	3	3	2	3	3
1	1	1	1	1	4	4	4
2	2	3	2	3	3	2	2
3	2	3	2	2	2	3	3
2	1	4	2	1	4	4	4
3	2	3	2	1	3	3	3
4	1	4	3	1	4	4	4
2	2	2	2	2	3	3	2
3	1	4	2	2	3	3	3
3	2	3	99	1	2	2	2
4	1	99	1	1	2	3	3
3	1	4	1	1	2	3	2
2	3	3	2	2	3	3	3
3	2	3	2	1	4	3	4
3	3	3	2	1	3	3	4
3	3	3	3	1	4	4	4
2	1	4	1	1	4	4	4
99	99	99	99	99	99	99	99
3	1	3	2	1	3	3	3
1	1	1	1	1	3	3	3
4	2	3	1	1	3	3	3
4	1	4	1	1	4	4	4
4	2	4	2	1	4	4	4
2	1	1	1	1	4	4	3
3	1	3	1	1	4	4	4
99	99	99	99	1	3	4	3
4	3	3	2	1	4	4	4
1	3	2	3	2	3	2	4
3	2	2	2	1	4	4	3
99	99	3	3	99	99	99	2
2	99	3	99	3	2	3	4
2	3	4	1	1	3	4	3
3	3	3	3	2	3	4	2
3	2	3	2	2	4	2	3
4	1	4	1	1	4	4	4
4	1	1	1	1	4	4	4
2	3	4	4	2	4	4	3
4	3	4	1	1	4	4	4

99	2	3	2	1	99	3	3
4	2	4	1	1	4	4	3
4	2	3	2	1	3	3	2
2	2	3	99	1	3	4	4
4	1	4	1	1	1	1	1
2	3	3	3	2	3	4	4
3	2	3	2	1	3	2	4
3	3	3	2	2	4	4	4
3	2	3	2	2	3	3	3
1	2	4	2	1	4	4	3
99	99	99	99	99	4	4	4
3	1	3	2	1	4	4	4
3	2	3	2	2	3	3	3
3	2	3	2	2	3	3	3
2	2	3	1	2	3	3	4
4	4	4	4	4	4	4	4
2	3	3	2	2	3	2	3
2	4	4	3	2	4	4	4
3	2	3	2	2	2	2	3
4	4	4	4	1	3	2	2
2	4	1	4	3	4	4	4
2	2	2	3	2	2	2	3
3	3	2	3	2	3	2	3
99	99	99	2	2	4	4	3
2	3	3	3	2	4	4	4
2	3	3	3	1	4	4	4
2	2	3	2	2	3	4	3
2	1	4	2	2	1	1	2
99	99	99	99	99	99	99	99
2	4	3	3	3	2	2	2
2	3	3	3	2	3	3	3
4	1	4	2	1	3	3	4
99	99	99	99	99	99	99	99
3	2	3	2	2	2	2	99
3	1	3	3	1	1	1	1
4	1	4	1	1	4	4	4
1	3	4	4	2	3	4	4
1	3	2	99	1	4	4	4
4	2	4	2	2	4	4	4
3	1	4	1	1	3	3	4
3	2	1	1	1	3	3	4
1	3	2	2	2	4	3	4
2	3	3	3	2	3	3	3
99	99	99	99	99	99	99	99
2	2	3	2	1	4	4	4
3	2	3	2	2	3	3	4
2	3	3	2	2	2	4	3
2	2	2	3	3	2	3	4

4	2	3	2	1	4	4	4
4	1	4	1	1	3	4	4
4	1	4	1	1	4	4	3
2	99	99	1	1	4	4	1
2	2	3	2	2	4	4	3
3	1	1	1	1	3	3	4
1	2	3	3	3	4	3	4
3	3	3	2	2	3	4	4
2	3	3	2	3	3	2	3
3	3	4	3	1	4	2	4
3	3	3	2	2	3	3	3
2	3	3	3	3	3	3	3
3	2	3	2	2	2	4	4
3	2	3	2	1	2	2	3
2	2	3	2	2	3	3	3
4	1	4	1	1	4	4	4
4	1	4	4	1	4	4	4
4	1	1	1	1	4	4	1
3	2	4	2	1	4	4	4
2	2	3	2	1	3	2	3
1	1	4	1	1	3	3	2
2	2	4	2	2	4	4	4
3	2	4	2	2	4	4	4
3	3	2	3	3	2	3	3
2	3	4	3	3	4	4	4
2	3	2	3	2	4	4	3
2	2	3	3	3	1	1	1
3	1	4	2	1	4	4	4
3	2	3	2	2	3	3	3
2	3	3	3	3	3	3	3
3	2	3	2	1	4	4	4
2	2	2	2	3	3	3	4
3	2	4	1	1	3	2	2
3	1	4	1	1	4	4	4
4	2	4	1	1	4	4	3
3	3	2	1	3	4	3	3
2	2	3	2	1	3	2	3
3	1	3	1	1	3	3	2
3	3	3	2	2	2	3	2
2	3	2	4	3	3	3	4
3	2	4	2	1	4	4	4
4	3	1	1	1	2	1	2
3	2	2	1	1	3	4	3
2	3	2	3	2	2	2	2
2	3	3	3	1	3	4	3
4	3	3	2	3	4	4	3
3	2	3	2	2	3	2	3
4	4	4	4	1	4	4	4

4	3	4	2	1	4	4	3
3	3	3	3	2	3	2	3
2	1	3	1	1	4	4	3
3	2	3	2	1	99	99	99
3	3	3	3	2	2	3	3
2	2	3	2	1	4	4	4
4	1	4	1	1	99	99	99
4	3	2	2	1	4	4	3
1	3	3	3	3	4	4	4
1	3	4	4	4	2	2	2
4	1	4	1	1	3	4	3
2	2	4	4	4	4	4	4
3	2	3	2	1	4	4	2
2	2	3	2	1	3	4	3
4	4	4	4	99	99	99	99
4	2	4	2	2	2	3	3
2	2	1	1	1	3	3	1
2	2	2	2	2	2	2	2
4	3	3	3	1	4	4	3
2	1	99	3	2	2	3	1
4	1	4	1	1	4	4	2
3	2	4	1	1	4	4	4
3	2	4	2	1	4	4	4
2	3	4	3	3	3	3	3
99	99	99	99	99	99	99	99
2	3	4	3	2	2	2	3
3	3	3	2	1	2	3	4
4	2	1	2	1	4	4	2
4	1	4	1	1	4	4	4
3	2	3	2	1	4	4	1
3	2	3	2	1	3	3	3
3	99	3	1	1	4	3	99
4	1	4	1	1	4	4	4
2	2	2	2	2	1	1	1
2	2	2	2	2	2	2	2
3	3	4	1	2	1	2	3
2	3	3	3	2	4	4	4
4	3	3	2	2	2	3	2
3	3	4	2	2	3	3	4
1	4	1	4	4	4	4	3
2	1	3	2	2	4	4	4
3	2	3	2	2	2	2	3
3	2	3	2	2	3	4	99
2	3	3	2	2	3	3	3
2	3	4	2	2	3	4	4
3	2	4	1	1	4	4	4
3	99	3	99	99	3	3	3
3	2	3	2	2	2	2	2

3	3	3	2	1	3	3	3
3	2	4	3	1	4	4	4
99	99	99	99	99	99	99	3
3	3	2	3	1	3	3	3
1	2	3	3	3	2	2	3
2	1	4	1	1	2	4	4
3	2	3	2	2	3	3	4
4	2	4	3	2	99	99	99
2	3	2	3	2	3	4	4
3	2	3	2	2	2	3	2
4	1	4	1	1	4	4	99
4	1	4	1	1	4	4	4
2	1	3	1	1	3	3	2
2	4	2	3	3	2	3	2
4	2	3	2	2	2	2	3
4	2	4	1	1	4	4	4
4	2	4	1	1	3	3	4
2	1	1	1	1	4	4	4
3	2	3	2	1	2	2	3
2	3	4	3	1	3	3	3
3	2	3	2	2	3	3	3
4	1	4	1	1	4	4	3
2	1	4	1	1	4	4	4
1	1	1	1	1	4	3	2
2	3	4	2	2	3	4	4
3	2	3	2	2	3	3	3
4	1	4	1	1	4	4	2
1	3	3	99	99	99	99	99
2	4	2	3	2	4	4	4
3	1	4	1	1	4	4	4
2	2	4	2	2	3	3	3
2	3	99	99	1	3	2	1
2	2	3	2	2	4	2	2
2	2	3	99	2	2	2	3
99	1	4	1	1	99	4	4
99	99	99	99	99	1	1	1
3	2	3	2	2	3	3	3
4	1	4	1	1	2	3	3
3	2	3	2	2	4	4	3
2	3	4	3	2	2	1	3
4	2	4	1	1	4	4	3
3	1	3	2	1	4	3	4
2	1	4	3	2	3	4	4
1	2	3	3	3	4	4	4
2	3	3	99	2	3	3	3
4	4	4	4	4	4	4	4
99	99	99	99	99	99	99	99
4	1	1	1	1	2	2	3

99	3	3	3	2	4	4	4
4	1	99	1	1	4	4	4
2	1	3	1	1	1	1	1
2	3	3	4	3	2	3	3
3	1	4	2	1	3	3	3
3	2	4	3	1	3	4	4
2	2	2	2	2	4	2	4
3	1	3	1	1	4	4	3
3	2	4	2	1	2	2	4
3	2	3	2	2	3	3	3
2	3	3	3	2	3	2	2
3	2	2	2	2	3	2	2
4	3	99	99	1	4	4	4
1	1	1	1	1	4	4	4
3	2	3	3	1	3	3	3
4	2	2	2	2	4	4	4
4	2	4	2	1	4	4	4
4	1	4	1	1	4	4	4
3	2	4	1	1	4	4	4
4	1	4	1	1	4	4	4
2	2	3	99	99	3	3	3
1	99	4	1	1	4	4	3
99	99	99	99	99	99	99	99
3	2	3	2	1	4	4	4
1	2	2	3	1	4	4	3
4	1	4	1	1	99	99	99
1	99	99	99	99	99	99	99
3	2	3	2	1	2	2	99
2	2	2	2	2	3	3	3
1	1	4	1	1	3	4	3
99	99	99	99	99	99	99	99
4	1	4	2	1	1	1	4
4	1	3	1	1	3	3	3
4	1	99	2	1	4	4	3
99	99	99	99	99	99	99	99
99	99	99	2	2	3	4	99
4	1	2	1	1	4	4	4
4	3	4	2	1	4	4	4
4	1	4	1	1	4	4	2
4	99	4	1	1	4	4	99
1	1	1	1	1	3	3	4
4	1	3	1	1	2	3	1
3	3	3	2	1	2	2	2
4	1	4	1	1	4	4	4
1	3	4	3	1	1	4	4
4	3	3	3	1	2	3	4
4	1	4	1	1	4	4	4
3	1	1	1	1	4	4	3

3	3	1	1	1	4	4	4
2	2	3	3	2	3	3	3
4	1	4	1	1	99	99	1
4	2	1	1	1	2	3	3
2	3	3	3	1	3	4	2
4	1	1	1	1	3	3	1
4	2	3	1	1	3	4	3
4	2	3	3	1	3	3	2
2	1	4	3	1	4	4	1
3	3	4	3	1	4	4	99
3	3	3	2	2	3	3	3
4	1	4	1	1	2	4	3
2	2	2	3	1	3	4	1
3	3	3	3	2	4	4	4
3	1	3	1	1	3	3	4
1	3	2	3	2	1	1	4
3	3	3	3	3	4	2	3
2	1	4	2	2	3	3	3
2	3	3	2	2	3	3	3
4	2	4	1	2	4	4	4
2	3	3	3	3	3	2	4
3	4	3	3	2	3	3	2
3	2	3	2	2	2	2	3
2	3	2	3	2	4	4	3
2	3	4	3	2	2	1	3
4	4	4	2	4	99	99	99
4	1	4	1	1	4	4	4
3	2	3	2	1	4	4	3
4	1	4	1	1	4	4	4
4	1	3	1	1	4	4	3
3	1	4	1	1	4	3	4
3	1	4	1	1	3	4	1
2	3	3	4	4	99	99	99
3	99	99	2	2	99	99	99
3	2	3	2	1	3	3	3
3	3	2	3	2	99	99	99
2	2	2	2	2	2	2	2
4	1	4	1	1	4	4	4
3	2	3	2	2	3	3	4
3	1	4	1	1	4	4	3
1	2	4	3	3	3	1	3
3	3	3	3	2	3	3	3
2	2	3	1	1	4	4	3
4	1	4	1	1	4	4	4
2	3	3	3	2	2	3	4
2	3	3	2	2	3	2	3
4	2	3	2	1	3	4	2
3	1	4	1	1	3	3	3

4	1	4	1	1	4	4	4
1	4	4	1	1	4	4	4
99	99	99	99	99	99	99	99
2	3	3	4	2	4	4	4
4	1	4	1	1	4	4	99
3	3	4	2	2	3	3	4
4	4	1	1	1	4	4	4
2	3	4	4	2	1	2	3
99	99	99	99	99	99	99	99
3	3	3	3	1	4	3	3
2	4	4	1	1	4	4	4
4	2	3	2	3	4	3	3
4	4	4	2	1	4	3	3
3	3	2	2	2	2	2	2
3	2	2	1	1	4	4	4
4	1	4	1	1	4	4	4
2	1	1	1	1	3	4	3
3	3	3	2	1	2	3	3
2	3	3	3	3	3	3	3
4	3	3	1	1	4	4	3
3	3	3	2	2	2	3	3
3	2	4	2	1	3	4	4
1	3	1	3	1	3	3	2
3	2	3	2	2	2	2	2
3	99	2	2	1	2	1	3
4	1	99	1	1	1	99	99
4	2	4	2	1	2	2	3
4	1	4	2	1	3	2	3
3	2	4	2	1	3	4	3
4	4	4	99	1	1	1	1
3	3	4	3	2	3	3	4
2	3	3	2	2	3	3	3
3	2	3	2	2	3	3	99
2	1	99	99	1	3	3	3
3	3	3	3	1	3	3	3
3	1	4	1	1	4	4	3
2	2	3	2	1	3	3	3
3	2	3	3	2	3	3	99
3	2	4	2	1	2	1	3
3	2	3	2	1	3	4	3
4	1	4	1	1	4	4	4
1	4	99	3	2	4	4	2
2	3	1	2	2	3	3	3
4	2	3	3	1	1	1	4
99	1	99	99	99	2	2	2
3	2	3	3	2	2	2	3
4	1	4	1	1	4	4	4
4	2	4	3	1	3	4	3

3	3	3	2	1	3	3	2
4	1	4	1	1	2	3	2
3	2	3	2	1	4	4	4
4	1	4	1	1	4	4	4
3	99	3	99	1	3	3	3
99	99	4	2	2	4	4	4
4	1	4	1	1	4	4	4
3	2	3	1	1	4	3	4
4	1	4	1	1	2	2	3
2	4	1	4	2	2	2	3
4	1	4	1	1	4	3	3
2	2	3	2	1	2	2	3
3	2	3	2	2	3	3	2
4	2	4	2	1	99	4	4
4	1	4	4	1	3	4	4
4	1	4	1	1	4	4	4
4	1	4	3	1	4	4	4
99	99	4	1	1	99	99	99
4	1	3	1	1	3	3	3
1	1	1	1	1	99	99	99
4	1	3	1	1	3	3	4
3	2	4	2	2	4	3	3
1	2	3	2	2	1	1	1
4	1	4	1	1	1	1	4
3	2	3	2	2	3	3	3
3	3	3	2	1	4	4	3
3	2	3	2	1	2	2	1
2	1	2	3	1	4	4	4
3	3	3	2	2	3	3	3
3	3	3	3	2	3	2	3
3	2	3	2	1	4	3	3
3	2	3	1	1	2	3	3
1	99	99	1	1	2	3	4
4	1	99	1	1	4	4	4
2	3	4	1	1	3	3	3
4	1	4	1	1	2	4	2
4	1	4	1	1	4	4	4
4	1	4	1	1	99	99	4
1	1	1	2	2	2	2	2
3	1	1	1	1	3	4	4
3	2	3	2	2	99	2	2
3	2	3	2	1	3	3	3
4	2	4	2	1	2	2	2
4	1	4	1	1	4	4	4
3	3	3	3	99	2	2	3
4	3	4	2	2	4	4	4
2	3	3	3	2	2	2	3
3	2	4	1	1	4	4	4

3	2	2	2	2	4	4	4
99	2	99	99	99	99	99	99
3	2	3	2	1	3	3	3
4	2	4	2	1	4	4	4
4	1	4	1	1	4	4	4
3	2	4	1	2	2	3	3
3	2	1	1	1	3	3	1
1	3	2	4	3	4	4	4
3	2	3	2	1	4	4	4
99	2	3	3	2	4	4	4
2	3	3	2	2	2	3	3
3	2	3	2	1	4	4	4
99	99	99	99	99	99	99	99
3	2	2	2	2	3	2	2
3	2	4	2	1	4	4	4
3	1	4	1	1	1	1	2
99	2	3	2	2	3	2	2
2	2	3	2	2	2	2	2
3	1	4	1	1	4	3	4
3	2	3	2	2	3	3	4
3	2	3	2	2	3	3	3
3	2	3	2	2	2	2	3
1	2	3	1	1	3	4	3
1	2	1	1	1	3	3	4
1	1	4	1	1	3	3	1
2	2	3	2	1	3	3	3
1	2	4	2	2	4	4	4
3	3	4	1	1	2	4	2
4	3	4	3	1	1	1	2
3	2	2	3	1	4	4	4
1	3	2	3	1	3	2	3
4	3	4	3	1	4	4	3
3	3	3	3	1	4	4	3
2	3	3	2	3	4	4	3
4	1	4	3	1	4	4	2
2	2	2	2	2	3	3	3
1	4	3	4	3	4	3	2
2	3	2	3	3	3	3	2
4	1	4	1	1	4	4	4
3	2	3	1	1	1	1	4
2	2	3	3	1	3	3	2
4	1	4	1	1	1	1	1
99	3	3	99	2	3	3	3
4	99	4	2	2	2	2	99
4	1	3	99	1	4	4	4
99	99	99	1	2	4	4	99
4	2	4	1	1	3	3	4
1	3	3	2	1	3	3	2

3	2	3	2	2	4	4	3
2	1	3	2	1	3	3	4
3	3	3	2	2	3	3	2
4	1	4	1	1	2	1	2
1	2	4	1	1	4	4	4
2	3	3	2	1	3	3	2
3	99	1	99	1	3	3	3
1	1	4	2	1	2	2	4
4	1	4	1	1	4	4	1
3	1	1	1	1	4	3	3
4	2	4	1	1	3	3	3
3	2	3	3	2	4	4	3
3	2	3	2	1	2	2	3
4	1	4	2	1	4	4	4
4	1	99	1	1	4	4	4
1	3	2	3	3	4	4	4
3	2	3	3	2	3	3	3
4	1	4	1	1	4	4	4
1	3	2	3	4	4	4	3
4	99	4	1	1	4	4	4
4	1	3	1	1	2	1	3
2	2	2	2	99	1	1	4
2	3	2	2	2	4	4	4
3	2	3	2	2	3	3	2
3	2	4	1	1	4	4	4
2	1	1	1	1	4	4	4
3	2	99	2	1	3	3	99
2	2	3	2	2	3	3	3
3	2	3	2	2	3	3	3
4	1	4	3	2	4	4	4
2	3	4	3	1	2	3	2
2	3	3	2	2	3	3	3
99	4	99	1	1	3	3	99
2	2	4	2	1	2	2	2
2	3	2	3	2	2	2	3
3	2	4	2	1	3	4	4
3	3	3	3	3	99	99	99
4	1	99	99	1	2	4	4
2	2	3	99	2	4	3	3
3	2	4	2	1	2	3	2
3	2	4	2	1	99	99	99
99	99	3	3	2	2	2	3
3	2	4	2	1	3	3	3
2	2	3	2	1	3	2	3
3	3	2	2	2	4	4	2
4	3	3	1	1	4	4	4
1	4	2	3	3	4	4	2
4	1	4	1	1	4	4	4

3	1	4	1	1	3	4	4
4	1	4	1	1	3	4	4
3	2	4	2	1	3	4	99
4	1	4	2	1	4	4	4
1	3	2	3	2	2	2	3
3	2	4	3	2	4	4	4
3	1	4	2	1	3	4	3
2	2	3	2	1	99	99	3
1	1	4	1	1	4	4	4
4	2	3	2	1	99	99	99
3	2	3	2	2	3	3	3
3	2	3	3	2	2	2	3
3	2	3	2	2	3	3	3
4	2	3	2	1	3	4	3
2	2	3	2	2	3	3	4
4	2	3	2	2	3	3	3
3	2	3	3	2	4	4	2
2	2	2	3	2	4	4	3
3	3	3	2	2	3	3	3
3	1	4	2	1	4	4	4
4	3	3	3	2	2	2	3
4	3	4	1	1	4	4	2
3	2	3	2	2	3	3	3
1	1	1	1	1	2	2	2
3	4	3	3	4	4	4	4
2	2	2	2	1	1	1	1
4	2	4	1	1	2	2	1
4	1	4	1	1	3	4	4
2	3	2	3	3	3	3	3
2	3	2	3	3	4	4	4
3	2	3	2	2	4	2	3
2	2	3	2	1	3	3	4
3	3	3	2	2	3	3	3
2	2	3	3	2	3	3	3
4	2	4	1	1	4	4	4
3	2	3	2	2	4	4	4
2	2	2	2	2	2	2	2
2	2	3	2	2	3	3	3
3	2	4	3	3	3	3	3
2	2	3	3	2	4	4	4
3	2	3	3	3	3	3	3
2	1	99	1	1	4	4	4
4	3	3	3	2	3	4	2
3	3	2	3	2	3	3	3
4	1	4	1	1	4	4	4
2	3	2	4	3	3	3	4
2	2	4	2	2	2	2	3
99	3	4	2	2	3	3	3

2	3	3	1	1	4	4	4
3	2	2	1	1	3	3	2
3	3	2	3	2	3	3	3
4	1	99	1	1	4	4	4
4	1	1	4	4	99	99	99
3	1	4	1	1	3	4	4
2	2	3	2	1	3	3	4
4	1	4	1	1	3	4	3
3	2	4	2	1	4	4	3
4	1	4	1	1	4	4	4
2	3	3	3	2	2	2	3
4	1	4	1	1	3	4	3
3	2	3	3	3	3	2	2
2	2	2	2	1	3	3	3
3	2	2	2	2	3	99	2
3	2	2	1	1	4	4	4
99	1	4	1	1	3	4	99
3	3	3	2	2	3	3	3
2	3	3	3	2	2	4	3
2	3	3	3	1	4	4	3
3	2	3	2	1	2	2	3
3	2	3	3	2	3	3	3
4	2	3	2	2	3	3	3
2	3	4	2	1	4	4	3
2	1	2	4	2	2	4	3
4	1	4	1	1	4	4	4
2	2	99	3	3	4	1	3
3	1	4	1	1	4	4	3
3	2	4	1	1	3	4	3
4	1	4	1	1	4	3	3
1	3	3	3	3	3	2	4
1	3	3	4	2	2	2	3
4	3	3	2	1	4	4	3
3	1	3	2	1	2	2	3
4	2	4	1	1	99	4	3
1	1	1	1	1	2	99	1
3	2	3	2	2	3	3	3
3	4	2	4	2	2	3	4
4	1	1	2	1	4	4	4
4	1	1	2	1	4	4	4
4	1	4	2	1	3	1	2
4	1	4	2	1	4	4	4
3	1	4	1	1	1	1	4
2	3	3	3	1	3	3	4
3	3	4	2	2	2	3	2
99	99	3	1	1	4	4	4
4	2	4	2	1	3	3	3
4	1	99	1	1	1	1	3

4	1	4	1	1	4	4	4
2	3	4	3	1	99	99	99
4	1	4	1	1	1	1	4
3	2	3	2	1	3	3	3
4	4	3	4	3	4	4	4
4	1	3	1	1	3	3	2
4	2	3	2	2	3	3	3
3	99	99	99	99	2	2	3
2	3	3	3	1	3	2	2
3	3	3	3	1	1	3	1
3	1	4	1	1	3	3	3
4	2	3	2	1	4	4	3
3	3	4	2	1	4	4	3
2	3	3	2	3	2	2	3
4	4	4	1	1	4	4	1
2	2	3	3	1	4	3	3
4	1	4	1	1	1	1	1
2	1	99	3	99	2	3	4
3	2	3	3	2	1	1	1
3	2	3	2	2	3	3	2
3	2	2	2	1	3	3	1
2	2	2	3	3	3	3	3
99	2	99	2	2	99	2	3
3	2	3	2	1	3	2	4
2	3	2	3	2	4	3	4
2	1	4	2	1	3	3	3
3	99	3	99	2	3	99	3
3	2	3	3	1	3	3	2
3	2	4	2	1	4	4	4
3	3	4	2	1	2	2	3
4	1	4	1	1	4	4	99
4	2	4	2	1	3	3	4
3	3	3	2	2	3	3	3
4	2	2	3	1	1	1	2
3	1	3	1	1	3	3	3
1	1	1	1	1	3	3	2
2	3	3	2	2	3	3	3
3	2	3	2	2	3	3	3
3	2	4	3	1	4	3	3
2	2	3	2	2	3	2	3
4	1	2	2	1	4	4	4
3	2	3	2	2	2	2	3
4	2	3	1	1	3	3	4
3	3	99	3	1	3	99	99
4	2	4	1	1	3	3	4
3	2	2	2	2	3	3	2
3	2	4	1	1	3	4	3
3	1	4	1	1	3	4	4

4	1	4	3	1	4	4	4
3	3	3	3	99	3	3	3
4	1	99	99	1	4	4	4
3	1	4	1	1	3	3	4
99	99	99	99	99	99	99	99
4	1	4	1	1	3	2	3
1	3	3	3	1	4	4	4
2	2	2	2	2	2	2	2
3	2	2	2	2	2	2	3
3	3	4	3	2	2	3	3
3	2	3	3	2	3	3	3
2	2	3	2	2	2	3	3
4	2	3	1	1	4	4	4
3	2	3	2	2	4	1	3
4	1	4	1	1	3	3	1
3	1	4	1	1	3	4	4
2	2	2	1	1	3	3	3
3	2	3	2	2	3	3	3
3	4	1	4	4	1	3	2
3	2	3	2	2	3	2	3
4	3	4	2	1	3	2	4
3	4	3	4	2	4	3	4
2	2	3	2	2	4	4	4
3	2	3	2	2	4	4	4
3	2	4	2	1	3	4	4
2	2	4	1	1	3	3	4
2	2	3	3	99	3	3	3
2	2	3	2	2	3	3	2
2	2	4	3	3	3	1	3
3	2	3	2	2	2	2	3
2	3	2	3	2	2	3	3
2	2	3	2	2	1	1	3
4	2	3	2	1	4	4	3
4	1	4	1	1	4	4	4
3	2	3	2	2	4	2	3
3	2	3	2	1	4	4	4
3	2	4	2	1	2	3	4
3	2	4	1	1	1	1	3
3	2	3	2	2	3	3	3
4	2	4	2	1	4	4	4
4	2	4	1	1	4	3	3
2	3	3	2	3	1	1	1
2	2	4	2	2	3	3	4
2	2	3	2	2	3	3	2
3	2	1	2	1	4	4	4
2	4	3	99	3	2	1	2
2	3	3	99	3	2	2	2
4	3	3	2	1	3	3	3

3	3	4	3	1	3	4	3
1	1	3	3	1	3	3	3
3	2	2	2	2	2	2	2
2	3	2	2	1	4	4	3
4	1	4	1	1	4	4	4
3	2	4	3	1	4	4	4
4	1	1	1	1	3	3	3
2	3	3	3	1	4	4	3
4	1	4	1	1	4	4	3
2	1	3	3	1	2	3	4
4	1	3	1	1	2	3	2
3	1	3	1	1	3	3	3
4	1	4	1	1	3	3	4
3	2	3	3	1	2	3	3
99	99	99	99	99	4	4	99
2	2	4	3	1	4	4	4
3	3	3	3	1	4	4	4
4	1	99	1	1	4	4	4
4	2	3	2	1	3	3	3
4	1	3	2	1	99	99	99
3	3	4	3	1	3	4	3
3	2	3	2	2	2	2	2
3	3	3	3	1	3	2	2
3	2	3	1	1	3	4	3
1	3	4	1	1	4	3	3
1	1	2	1	1	3	2	4
4	3	3	2	2	3	3	3
3	1	4	1	1	4	4	4
3	1	3	2	1	3	2	3
4	2	4	1	1	4	4	3
4	2	4	2	1	2	3	3
3	99	4	3	1	3	4	3
4	3	3	2	1	4	3	4
3	2	3	2	1	3	3	2
2	3	2	2	1	4	3	4
4	1	4	1	1	3	4	4
2	3	4	2	1	1	1	2
3	1	4	1	1	1	1	2
1	2	3	2	1	2	2	4
2	2	3	1	1	4	4	4
4	1	4	1	1	4	4	4
4	1	4	1	1	4	4	4
99	99	99	99	1	99	99	99
2	3	2	3	3	3	2	3
3	1	4	2	1	4	4	4
4	4	4	1	1	4	4	4
3	2	3	1	1	3	2	2
2	3	3	3	2	3	3	3

3	3	3	2	2	3	3	3
3	2	3	3	3	3	3	2
3	2	3	2	2	2	3	3
2	2	3	2	1	3	3	2
2	2	3	2	2	3	3	3
1	4	1	4	2	4	4	3
2	3	3	3	2	3	3	3
2	1	4	1	1	1	3	4
1	1	4	3	1	2	3	4
4	1	4	1	1	4	4	4
2	4	2	4	4	2	2	2
2	1	3	1	1	1	1	1
3	3	3	3	3	99	2	3
2	2	3	3	1	2	3	3
2	3	3	3	3	2	2	2
2	3	3	3	3	2	2	2
3	3	4	2	2	2	2	3
3	2	2	2	2	2	2	3
3	2	4	1	1	2	3	1
1	4	4	2	1	3	2	4
3	2	3	2	2	4	4	4
99	99	99	2	2	3	3	3
3	2	3	2	2	3	3	3
2	2	3	2	1	3	2	3
2	2	3	2	2	4	4	3
2	3	3	2	1	2	3	3
1	3	3	3	3	3	3	3
99	2	3	2	99	4	3	4
3	2	4	1	1	3	4	4
3	2	3	2	2	4	4	4
3	1	3	2	1	3	3	2
3	3	4	4	3	3	2	3
3	2	3	2	1	4	4	4
3	3	4	3	2	4	4	4
2	3	3	3	3	3	3	4
3	2	2	2	3	4	4	4
3	2	2	2	2	3	4	4
4	2	3	2	2	2	2	2
2	3	3	2	2	3	3	3
3	2	3	2	2	4	4	4
3	3	2	2	2	4	4	4
4	2	4	1	2	3	4	2
2	2	3	2	2	2	3	4
4	1	4	1	1	3	3	3
1	3	4	2	2	2	2	3
3	2	4	1	1	3	3	3
2	3	2	3	3	4	4	4
3	2	3	2	2	3	3	2

4	1	4	1	1	4	4	4
1	3	1	4	3	2	1	1
1	4	2	4	4	4	4	4
3	2	3	2	2	3	3	3
1	1	4	1	1	3	1	4
1	3	2	3	3	2	1	2
2	3	3	3	2	3	3	3
3	3	3	3	3	3	3	3
3	3	2	3	2	3	2	2
4	2	3	2	1	1	2	4
2	2	3	3	2	3	3	3
2	2	3	4	2	4	4	2
4	99	4	1	1	4	4	4
3	3	3	2	2	3	3	3
3	2	3	2	2	2	2	3
2	3	3	2	1	3	4	3
1	1	4	1	1	4	1	1
3	1	4	1	1	4	4	4
3	2	3	1	2	3	3	3
2	4	4	3	1	3	3	4
3	4	3	3	1	4	4	2
4	1	4	1	1	3	3	4
3	3	3	2	2	3	3	3
99	3	3	3	1	4	4	3
3	3	3	2	2	4	4	4
3	2	4	2	1	3	3	3
2	3	3	3	2	2	2	2
4	1	4	1	1	4	4	4
2	99	99	2	99	3	3	2
4	1	4	1	1	4	4	3
3	2	3	2	4	2	3	3
3	2	3	2	1	2	3	2
2	2	4	2	1	3	4	3
1	1	3	1	1	3	4	3
2	3	2	3	2	4	4	4
1	2	4	2	1	4	4	4
4	1	4	1	1	3	4	4
3	3	4	4	2	3	3	3
3	3	4	2	2	4	3	3
3	3	3	3	1	2	2	4
2	4	1	4	3	2	2	1
4	2	4	2	1	4	4	4
2	2	1	2	1	3	4	4
4	1	4	2	1	2	4	2
4	1	4	1	1	4	4	99
4	1	4	2	1	4	4	4
2	3	2	3	2	3	3	2
3	2	3	2	1	3	4	3

2	2	4	2	2	3	2	4
1	3	3	3	2	4	4	4
3	2	3	2	2	3	3	2
3	2	3	2	1	3	3	3
4	1	4	1	1	4	4	4
4	99	99	99	1	4	99	99
2	2	1	1	1	3	2	2
3	2	3	3	2	4	3	2
1	3	4	3	3	1	1	1
2	2	3	2	2	4	3	3
4	1	4	1	1	2	4	3
4	1	4	1	1	4	4	1
2	3	99	3	99	4	3	3
2	2	2	3	2	4	2	3
3	1	3	1	1	3	3	3
3	2	3	2	2	3	3	3
4	1	4	1	2	4	3	4
4	3	4	3	1	4	1	3
99	2	99	2	2	2	3	2
3	3	2	3	1	3	3	4
4	2	3	2	1	4	4	3
3	1	4	2	1	4	4	4
3	2	3	2	2	3	3	3
99	3	3	99	99	3	3	3
3	99	99	2	2	3	3	3
3	2	2	2	2	2	2	2
2	3	2	2	2	3	3	3
4	2	4	2	1	2	2	4
4	99	3	1	1	3	4	3
99	1	4	1	1	4	3	3
4	1	4	1	1	4	4	4
1	1	1	1	1	1	1	1
3	2	3	2	1	4	4	4
1	3	4	2	2	3	3	4
4	2	4	1	1	4	4	4
2	2	2	3	2	3	3	3
3	2	3	2	2	4	4	4
2	1	3	2	3	2	3	4
2	2	99	99	99	99	99	99
99	99	99	99	99	3	3	4
3	1	3	3	3	4	4	4
2	3	2	3	2	1	1	4
3	2	3	2	1	3	3	3
2	3	3	3	3	4	4	3
4	4	4	4	4	4	4	4
1	2	99	3	1	3	3	3
3	2	3	2	2	4	4	4
2	1	4	2	1	2	1	2

1	3	3	3	3	2	3	3
3	4	2	99	3	3	3	3
2	2	3	2	3	4	4	4
1	1	4	4	4	3	3	3
2	2	3	3	2	3	3	3
2	2	2	2	2	3	3	3
1	4	1	4	4	4	4	1
1	1	4	1	1	4	4	4
4	1	4	1	4	4	4	4
3	2	3	2	1	3	3	2
99	1	4	1	1	4	4	4
4	1	4	1	1	4	4	4
3	1	4	3	2	4	4	4
4	1	4	1	1	4	4	4
4	99	1	99	2	4	3	4
3	2	4	3	2	2	3	3
3	3	3	3	3	4	4	4
2	3	2	3	2	3	3	4
3	2	2	3	3	4	3	3
3	2	2	4	1	4	4	4
3	3	4	2	2	2	4	4
2	1	1	1	1	2	3	3
4	1	4	1	1	4	4	4
1	4	3	1	1	1	1	1
1	4	4	1	1	1	1	1
3	2	3	2	2	3	3	3
4	1	4	1	1	3	3	3
1	1	4	1	1	1	1	1
3	1	1	1	1	4	4	1
3	2	3	2	2	3	3	2
3	2	3	2	1	4	4	4
4	1	1	1	1	99	99	99
3	2	99	99	99	99	99	99
3	3	2	3	2	2	2	2
3	3	3	2	1	3	3	3
4	1	4	1	1	4	4	4
4	1	4	1	1	3	4	4
99	99	99	1	1	99	99	99
4	3	99	99	1	3	4	4
1	1	1	2	1	2	2	1
4	1	4	1	1	1	1	1
4	3	3	2	1	4	4	4
4	1	4	1	1	4	4	4
4	1	4	1	1	1	1	1
4	2	3	2	2	2	2	3
4	1	4	1	1	4	4	4
4	1	4	2	1	3	4	4
2	2	4	2	1	3	2	4

3	2	3	2	2	3	3	3
1	1	4	4	1	1	1	1
1	1	4	1	1	1	1	1
4	1	4	2	1	1	3	4
3	2	3	2	2	3	3	2
4	1	3	1	1	3	3	2
4	1	4	1	1	4	4	4
3	1	4	2	1	4	4	4
3	2	3	2	2	2	2	2
3	2	4	1	1	4	4	2
4	4	4	1	1	4	4	4
4	1	4	1	1	3	4	3
3	3	2	3	2	2	2	99
4	3	3	2	1	4	4	99
3	2	3	2	2	2	2	2
4	4	4	2	4	3	3	3
4	1	4	1	1	4	4	4
4	2	99	1	1	99	99	99
2	3	3	3	2	2	3	2
1	2	4	2	1	4	4	4
4	2	3	2	1	3	3	3
3	2	4	2	1	3	3	3
4	2	4	1	1	3	3	4
3	3	3	2	3	2	3	3
2	2	2	2	2	2	2	2
4	2	4	1	1	4	4	4
4	1	4	1	1	4	4	4
4	1	4	1	1	4	4	4
4	1	4	1	1	2	3	3
4	99	99	1	1	99	99	99
4	1	4	1	1	4	4	4
2	2	2	4	4	4	2	4
3	1	1	1	1	2	3	4
3	1	4	1	1	2	3	4
3	1	4	1	1	4	4	4
3	2	2	2	2	4	4	4
4	4	4	1	1	1	1	1
1	4	1	4	2	4	3	2
4	3	4	2	1	4	4	4
4	1	4	1	1	4	4	4
99	99	99	99	1	4	4	4
3	2	3	2	1	4	4	3
2	2	4	2	1	3	2	3
4	2	3	2	1	4	4	4
3	1	4	1	1	3	3	3
3	2	3	2	1	3	3	2
3	2	3	2	2	3	3	99
4	1	4	1	1	4	4	4

3	2	4	2	1	4	4	3
4	1	2	1	1	4	4	4
3	2	3	2	2	3	3	3
3	2	4	1	1	4	4	4
4	1	4	3	1	3	3	3
3	2	3	2	2	3	3	2
99	1	4	1	1	4	4	3
2	3	3	2	2	4	3	3
2	3	2	3	3	2	2	2
4	2	4	2	1	3	4	2
1	3	4	4	1	4	4	4
3	1	3	2	1	4	4	4
3	2	3	2	2	3	3	3
2	3	2	3	3	3	2	4
3	3	3	2	2	2	2	3
3	2	3	2	2	3	4	3
4	3	4	2	1	3	3	2
4	2	4	1	1	2	2	3
2	2	4	2	2	3	4	4
2	2	3	2	1	2	2	3
3	3	3	3	2	3	3	2
4	1	1	1	1	4	4	4
4	1	4	1	1	4	4	4
4	1	4	2	1	2	2	2
3	1	3	1	1	1	1	1
3	1	3	1	1	3	3	3
3	3	3	3	1	3	3	4
4	1	4	1	1	4	4	4
4	1	4	1	1	4	4	4
3	2	3	1	1	3	3	3
3	2	3	2	2	4	4	4
2	3	3	3	2	3	3	3
3	2	3	2	1	3	3	3
2	3	3	2	1	2	3	3
2	1	3	2	1	3	4	4
99	99	2	99	99	4	4	4
4	1	4	1	1	4	4	4
3	2	3	2	2	3	3	3
4	1	4	1	1	4	4	4
3	3	3	99	2	4	4	2
3	2	3	2	2	3	3	3
1	2	2	2	2	3	4	3
2	3	2	3	3	3	2	3
2	2	99	2	2	3	3	2
99	99	99	99	99	2	3	4
2	3	2	2	2	4	4	4
3	3	3	2	2	3	3	3
3	2	3	2	1	4	4	3

2	2	2	2	2	2	2	2
3	99	3	2	2	1	1	99
3	1	99	1	1	99	99	99
2	2	2	1	1	3	4	3
3	2	4	2	1	4	4	1
4	3	3	2	1	3	3	3
99	2	3	2	1	99	99	99
4	2	2	1	1	99	2	2
4	2	3	2	1	4	4	4
2	2	2	1	2	3	4	3
99	99	99	99	99	4	4	4
4	1	4	1	1	99	3	99
3	1	1	1	1	3	3	3
4	1	4	1	1	1	4	4
2	1	4	2	1	4	4	4
4	1	4	1	1	4	4	4
2	2	2	2	1	3	3	2
4	3	1	1	1	4	4	4
4	2	4	2	1	2	2	2
4	1	4	1	1	4	4	4
1	2	4	1	1	4	4	4
3	99	3	99	99	99	99	4
4	1	4	2	1	3	3	4
3	2	3	2	2	2	3	3
3	3	3	3	1	4	4	3
4	1	4	1	1	3	3	3
4	2	4	2	2	3	3	2
1	3	3	3	2	3	3	2
4	1	4	1	1	4	4	4
4	1	4	1	1	4	4	4
4	2	4	2	1	2	2	2
3	3	4	2	1	3	3	4
3	2	99	99	2	3	3	3
99	99	3	3	2	4	3	3
4	1	1	1	1	3	4	1
99	1	99	1	1	3	3	4
4	1	4	1	1	4	4	4
2	1	3	1	2	2	3	4
4	1	4	1	1	3	3	2
4	2	3	2	1	4	4	3
2	2	3	2	1	1	1	1
2	2	3	3	1	3	3	2
2	2	2	2	2	3	2	3
3	2	4	2	2	3	3	3
2	2	4	1	1	3	4	3
4	1	4	1	1	3	4	4
3	2	3	2	1	4	4	4
2	3	3	4	4	2	2	4

3	2	99	2	2	3	2	3
4	1	4	1	1	4	4	4
3	1	3	1	1	2	3	4
2	2	3	3	99	3	3	99
99	1	4	1	1	2	2	99
4	2	1	4	1	4	4	4
3	2	3	2	2	3	3	3
3	2	3	2	2	4	4	4
3	2	3	2	2	4	4	4
3	2	3	1	1	4	4	4
2	3	3	2	2	3	3	3
3	1	4	2	1	4	4	4
3	2	4	2	1	3	3	4
4	3	4	4	1	4	4	3
4	3	4	2	2	4	3	4
2	2	2	1	2	3	3	2
4	1	4	1	1	4	4	4
4	1	4	1	1	4	4	3
4	2	3	2	2	3	3	3
4	2	3	2	2	4	3	4
4	3	4	4	4	4	4	4
3	3	3	3	1	2	1	2
4	1	4	1	1	4	4	4
4	1	3	1	1	4	4	2
3	3	4	3	1	4	4	4
99	99	99	99	99	99	99	99
2	3	3	3	1	4	4	2
4	1	4	1	1	4	4	99
2	2	3	2	2	3	3	3
3	2	3	3	1	3	2	3
2	2	3	3	2	3	3	3
3	1	4	2	1	2	3	4
4	1	4	1	1	3	3	3
2	2	4	2	2	2	2	4
3	2	3	2	2	2	2	3
3	2	4	3	1	3	3	3
2	3	2	3	3	2	2	2
2	3	3	3	2	3	3	2
4	2	3	2	2	4	3	3
4	1	4	1	1	4	4	4
99	99	99	99	99	3	3	3
1	1	4	1	1	2	2	4
3	2	3	2	2	3	2	3
4	1	3	1	1	4	4	4
4	1	4	1	1	4	4	4
3	2	3	3	2	2	2	3
3	3	4	2	1	3	3	4
3	2	4	2	2	3	3	3

4	2	4	2	1	4	4	2
2	2	2	2	2	1	1	2
4	1	4	2	1	3	4	4
2	3	3	3	2	4	3	3
4	1	4	1	1	3	3	3
3	1	4	2	1	1	1	4
4	1	4	1	1	3	3	4
1	1	1	1	1	3	3	3
2	3	3	3	2	2	2	3
4	1	3	1	1	3	3	3
2	2	2	2	2	3	3	3
3	3	3	2	2	2	1	1
2	3	3	3	3	3	3	2
4	1	1	1	1	4	4	4
3	3	3	2	1	4	4	3
4	1	4	1	1	4	4	4
2	2	1	1	1	2	2	2
4	2	4	2	2	3	3	3
4	1	4	1	1	3	4	4
4	1	4	2	1	4	4	4
3	2	3	2	2	2	2	1
4	2	3	2	1	4	4	3
3	2	3	2	1	4	3	3
1	4	4	3	2	4	4	2
2	2	3	2	1	3	3	3
1	2	3	3	1	3	3	3
4	1	4	1	1	4	4	3
2	3	2	3	2	3	2	3
3	3	3	2	1	2	2	2
2	3	3	2	2	3	3	3
4	3	3	2	1	4	4	2
4	1	4	1	1	4	3	4
3	3	3	2	1	3	3	3
3	2	3	1	1	3	4	3
4	1	4	2	1	4	4	1
2	3	3	3	2	3	3	4
4	1	4	1	1	4	4	3
4	1	4	1	1	4	4	4
4	1	4	1	1	4	4	4
4	3	3	2	2	3	3	2
3	2	3	2	1	2	3	2
4	3	99	1	1	4	4	3
4	1	4	1	1	1	4	1
3	3	3	2	1	3	3	3
2	2	3	2	2	3	2	2
2	1	99	2	1	3	3	99
4	99	99	1	1	4	4	4
1	1	4	1	1	4	4	4

3	2	3	2	1	2	4	2
2	3	2	3	2	3	2	3
4	2	4	99	99	3	3	3
99	99	99	99	99	99	99	99
4	1	4	1	1	4	4	4
3	2	4	1	1	3	3	2
3	2	3	2	2	3	3	3
3	2	3	3	2	3	3	3
2	2	3	2	3	1	1	3
4	2	4	2	2	4	4	4
99	99	99	99	99	99	99	99
4	1	3	2	1	3	4	4
2	99	3	2	99	99	3	4
4	1	4	1	1	4	4	4
2	2	3	2	2	2	3	2
4	1	4	1	1	4	4	4
3	4	3	3	1	4	4	3
3	2	3	2	1	3	3	3
3	2	4	2	2	3	3	3
4	1	3	2	1	3	3	4
4	2	3	1	1	3	2	4
3	2	3	2	2	3	3	3
4	3	3	3	99	3	1	99
2	3	3	2	2	3	3	3
3	2	4	2	1	2	2	2
3	3	3	2	1	4	3	4
4	2	3	1	1	3	3	3
4	1	4	1	1	4	4	4
4	1	4	1	1	3	3	3
3	3	3	1	1	4	4	4
3	99	2	2	2	3	3	3
2	3	2	3	2	3	3	2
4	1	4	1	1	1	1	1
4	2	3	2	1	3	3	3
1	1	1	1	3	4	1	1
3	4	4	2	2	3	2	3
4	1	4	1	1	4	4	3
3	2	3	2	2	3	3	3
2	2	3	3	3	2	2	1
1	3	2	3	3	3	2	2
4	2	3	2	1	4	4	3
2	2	1	3	1	4	4	3
4	1	4	1	1	4	4	4
1	4	1	4	4	4	2	2
2	3	3	3	3	3	3	3
3	3	2	2	2	4	3	4
99	2	3	2	2	3	3	4
3	99	3	2	2	3	3	3

99	99	99	99	99	99	99	99
3	3	3	3	1	3	3	3
4	1	4	1	1	4	4	4
3	2	2	2	1	2	3	2
4	1	4	1	1	3	4	4
2	2	2	2	2	3	3	3
3	2	3	1	1	4	4	4
4	1	4	1	1	99	99	4
2	3	3	2	1	3	3	3
4	2	4	1	2	1	2	1
99	99	99	99	99	99	99	99
99	99	99	99	99	99	99	99
2	3	99	99	1	3	99	3
1	1	4	1	1	4	4	4
4	2	4	1	2	4	4	3
99	2	3	99	99	4	3	4
99	99	99	99	99	99	99	99
99	2	4	99	1	4	4	4
3	2	3	2	2	3	3	3
3	2	4	2	1	2	2	3
3	2	4	2	1	4	4	2
3	2	4	1	1	4	4	4
4	2	3	1	2	4	4	4
2	1	3	2	1	4	4	4
3	2	3	2	2	2	3	3
2	3	3	2	2	3	3	3
99	99	99	3	99	99	99	99
2	3	2	3	2	2	3	2
3	2	3	2	2	3	3	3
3	99	2	2	1	3	3	3
4	2	2	2	1	2	3	3
4	99	1	1	1	3	3	99
4	3	4	3	2	3	4	3
3	2	3	2	1	4	4	4
4	1	99	3	1	4	4	1
3	2	3	3	1	4	4	3
4	1	4	1	1	3	4	3
4	1	4	1	1	4	4	4
4	1	4	1	1	4	4	4
4	1	4	1	1	3	3	3
3	3	3	3	2	3	3	3
3	2	3	2	1	4	4	4
4	99	4	99	1	99	99	99
3	2	3	2	1	3	4	3
2	2	2	2	2	3	3	2
4	2	3	1	1	4	4	3
1	1	4	1	1	4	4	4
4	2	4	1	1	4	4	2

1	4	4	1	1	4	4	4
4	1	3	2	1	2	3	3
3	2	3	2	2	3	3	3
3	1	3	1	1	4	4	4
4	1	4	1	1	4	4	4
3	3	2	2	1	3	3	2
2	2	3	2	2	3	3	2
4	2	4	2	1	3	4	3
4	4	4	1	1	4	4	4
2	3	2	3	2	2	2	2
3	3	3	2	2	2	2	2
3	3	3	2	2	2	2	2
3	2	2	2	1	2	1	2
4	1	99	2	1	4	4	4
4	2	4	1	1	2	3	3
3	3	3	1	1	3	3	3
4	1	4	2	1	3	3	1
1	1	4	2	1	3	3	4
2	3	2	3	2	3	3	3
4	1	4	1	1	4	4	4
3	2	3	2	1	4	4	2
4	1	4	1	1	4	4	4
3	3	4	1	1	4	3	2
99	99	99	99	99	99	99	99
3	3	3	2	2	3	3	3
3	2	4	2	1	4	3	4
4	2	4	2	1	4	4	3
3	2	3	1	1	3	4	3
1	3	2	4	4	1	1	1
4	1	4	2	1	2	3	3
3	99	3	2	2	3	3	99
99	99	99	99	99	99	99	99
3	99	3	1	2	3	4	3
4	2	3	2	1	4	4	4
2	3	3	3	2	3	3	3
3	2	3	2	2	3	2	2
3	3	2	2	2	3	3	3
3	1	1	1	1	4	4	4
3	2	3	2	2	3	3	3
3	2	3	1	2	3	3	2
3	2	3	3	2	2	2	2
1	1	1	1	1	2	2	3
99	99	3	2	2	3	3	2
4	2	3	1	1	3	3	4
99	3	99	99	2	99	99	99
4	4	4	1	1	4	4	4
2	3	4	1	1	2	2	4
3	1	4	2	1	3	4	4

3	2	4	2	2	4	4	4
4	1	4	1	1	4	4	4
3	2	3	3	1	4	4	4
2	2	2	2	2	3	3	3
4	2	4	1	2	4	4	4
2	3	2	2	2	3	3	2
4	1	4	1	1	4	4	1
4	1	4	1	1	4	4	4
3	2	4	1	2	3	3	3
3	3	3	2	2	3	3	3
2	3	2	3	3	3	3	3
3	3	2	3	1	3	3	3
2	1	4	3	2	3	2	3
99	99	99	99	99	99	99	99
99	99	3	99	99	2	2	2
3	2	4	1	1	4	4	4
2	99	3	2	3	4	4	3
1	3	1	3	4	4	3	3
1	4	4	4	3	4	1	2
3	2	3	2	2	2	3	3
2	2	2	2	2	3	3	3
3	2	4	1	1	4	4	4
4	2	4	2	2	4	4	4
4	2	3	2	1	4	4	3
4	2	4	2	1	2	2	3
3	2	3	99	2	3	3	3
2	3	3	3	2	3	3	3
4	2	4	1	1	4	4	3
2	1	3	1	1	3	4	4
3	2	2	2	2	4	4	3
3	3	3	3	1	3	3	4
3	99	4	99	99	4	4	4
3	2	1	2	1	3	3	4
4	3	3	3	3	4	3	2
4	1	4	1	1	4	4	4
4	1	1	1	1	2	2	2
2	2	2	2	2	3	3	3
4	1	3	2	1	3	4	3
4	1	4	2	1	3	3	3
2	2	3	2	2	2	2	3
3	2	3	2	1	4	3	3
3	1	2	2	1	2	2	2
2	1	4	1	1	2	3	3
3	2	3	2	1	2	2	2
2	3	3	2	2	3	3	3
4	4	4	1	1	4	4	4
3	2	4	1	1	4	3	3
3	2	3	2	1	4	4	4

2	2	2	99	2	2	2	2
3	2	3	2	2	3	3	2
2	3	3	3	1	4	4	1
1	2	1	2	2	4	4	4
99	3	3	3	3	2	3	2
3	2	2	2	2	3	3	2
3	3	4	2	1	1	2	2
1	4	1	4	2	2	1	3
4	2	4	1	1	3	3	4
3	2	3	2	1	3	3	99
2	2	3	99	2	2	2	3
4	1	3	1	1	4	4	4
4	1	4	1	1	4	4	4
4	1	4	1	1	4	4	2
99	99	4	1	1	2	99	99
4	2	4	2	1	2	2	3
2	3	3	3	2	4	4	4
4	2	3	3	1	4	4	4
3	2	3	2	1	3	3	2
99	99	99	99	99	99	99	99
3	3	2	3	2	3	3	3
3	2	3	3	1	4	3	3
2	3	3	3	1	3	3	4
2	2	4	2	1	4	4	4
3	2	2	2	2	2	1	2
3	1	3	3	1	3	3	3
2	2	3	2	2	3	2	2
4	2	3	2	1	3	3	3
4	1	3	1	1	2	1	3
2	1	3	1	1	3	3	3
3	2	3	2	1	1	3	3
4	1	4	1	1	1	1	1
3	2	4	2	1	3	4	4
2	2	3	2	2	3	4	4
99	3	1	3	3	4	4	4
3	1	3	1	1	4	4	4
3	2	3	2	2	4	4	4
3	2	3	1	1	3	2	3
3	2	99	1	99	4	3	4
3	3	4	2	1	3	3	4
3	1	3	1	1	3	3	3
3	2	99	2	2	4	4	99
3	2	3	2	2	2	3	3
2	3	3	3	2	3	3	3
3	2	4	2	1	2	2	3
3	1	3	1	1	3	3	3
99	99	99	99	2	99	99	99
1	2	3	3	2	3	1	2

4	1	4	1	1	3	4	4
3	3	3	3	2	3	3	2
3	3	3	2	1	2	2	2
2	3	3	3	2	3	3	3
4	1	4	1	1	4	4	4
2	4	3	4	2	3	3	2
1	99	99	1	1	4	4	3
1	2	2	2	1	3	3	3
4	1	4	1	1	1	1	1
1	4	1	4	2	4	4	99
2	4	1	4	4	4	3	2
2	1	4	1	1	4	4	4
2	4	3	4	1	3	4	3
3	2	4	1	1	3	3	3
4	2	4	2	1	4	4	4
99	2	3	2	2	4	4	3
1	4	1	4	2	4	4	4
3	1	4	1	1	3	3	4
4	1	4	1	1	4	4	4
99	3	3	99	2	2	99	3
3	1	4	1	1	4	4	99
3	2	3	2	2	3	3	3
3	3	4	2	2	4	4	4
3	2	3	2	1	3	3	99
4	1	3	1	2	4	4	4
2	3	3	3	2	3	3	3
4	2	4	1	1	2	2	3
3	2	2	2	2	3	3	2
3	1	4	1	1	4	4	4
3	2	3	2	2	3	3	3
4	2	4	2	1	4	4	4
4	2	4	2	1	4	4	3
3	2	4	2	1	1	4	4
3	2	3	2	2	3	3	3
4	1	4	1	1	4	4	4
1	4	1	4	4	3	4	1
3	1	4	1	1	3	3	4
2	2	4	1	2	4	4	4
4	1	4	1	1	4	4	4
4	4	1	4	1	4	4	4
4	2	4	1	1	3	3	3
1	4	1	4	4	1	1	1
3	2	4	2	1	4	4	4
3	2	4	2	1	2	3	4
3	3	4	3	2	3	2	4
4	1	4	1	1	4	4	4
3	1	3	1	1	3	2	3
3	3	3	2	2	2	4	3

2	2	2	2	2	4	4	4
4	1	4	1	1	3	3	3
4	1	4	99	99	4	4	4
4	1	4	1	1	4	4	4
4	1	4	1	1	4	4	4
4	1	4	1	1	3	3	4
4	1	3	99	1	2	1	99
4	2	4	1	1	99	99	1
1	2	3	4	3	2	4	4
2	2	3	3	2	2	3	3
3	2	3	2	2	2	3	2
99	99	99	99	99	99	99	4
4	1	4	1	1	4	4	3
2	2	3	2	1	2	3	3
2	3	4	1	1	1	1	3
99	99	99	99	99	99	99	99
4	1	4	1	1	4	4	4
4	1	4	1	1	4	3	3
4	2	99	1	1	99	99	3
4	1	4	1	1	4	4	4
4	2	4	2	2	4	4	4
3	3	3	3	2	2	2	3
2	3	3	3	2	2	2	3
4	1	4	1	1	3	99	3
4	99	4	1	1	4	4	4
99	99	99	99	99	4	4	4
4	1	3	1	1	3	3	3
3	2	3	2	1	3	3	3
3	1	4	2	2	4	4	3
4	2	3	1	1	3	3	4
2	99	3	2	2	3	2	3
2	3	3	3	1	3	3	4
3	1	4	1	1	99	99	99
3	3	4	3	2	2	3	2
3	2	3	2	2	3	3	3
3	3	3	2	2	3	3	3
99	99	99	99	99	99	99	99
3	2	2	2	2	3	3	3
3	2	3	2	1	2	2	3
4	1	4	1	1	1	1	4
4	1	4	1	1	4	4	1
4	2	4	1	1	1	1	3
2	2	3	2	1	2	2	3
4	1	4	1	1	4	4	4
3	2	4	2	2	2	2	2
2	1	3	3	2	3	2	3
4	1	4	2	1	4	4	4
3	2	3	2	2	4	4	3

2	2	3	3	3	3	4	4
4	2	4	2	1	4	4	4
2	3	3	4	2	2	2	2
4	1	4	1	1	4	4	4
3	2	3	2	2	2	2	2
3	2	4	2	1	3	4	4
2	3	3	3	3	3	3	3
4	2	4	2	1	3	4	3
2	3	3	3	2	3	3	3
3	2	3	1	1	3	3	3
4	1	4	1	1	3	4	3
3	2	3	2	2	3	3	3
3	99	3	2	1	2	2	3
3	2	3	2	1	4	4	3
2	3	3	3	3	1	1	1
4	1	4	2	1	2	3	3
4	1	4	1	1	4	4	4
4	2	4	1	1	4	3	2
4	2	3	2	2	2	2	3
3	2	3	2	2	3	2	2
3	2	3	2	2	3	3	3
4	1	3	1	1	4	4	3
3	2	3	2	2	3	3	3
4	2	4	2	1	4	4	3
1	4	1	4	4	1	1	1
2	1	3	2	1	4	4	4
4	1	4	1	1	4	4	4
2	2	2	2	2	3	3	3
1	2	3	2	2	3	3	3
3	2	3	2	2	2	3	3
2	3	3	3	4	2	2	3
99	99	99	99	99	3	3	3
2	1	4	3	2	4	4	4
2	3	3	3	3	4	4	4
2	2	3	3	1	2	2	2
3	3	3	3	3	4	4	4
4	1	1	1	1	1	1	3
3	1	4	1	1	4	4	4
1	1	1	1	1	1	1	1
3	2	3	3	1	1	1	3
2	2	2	3	1	4	1	3
1	3	2	3	4	4	3	4
4	3	1	2	2	4	4	3
3	2	4	2	2	2	2	3
1	3	2	3	3	2	2	2
2	2	2	3	3	4	4	4
3	2	2	2	1	4	4	2
2	2	2	2	1	3	3	4

4	1	4	1	1	4	4	4
4	2	3	2	2	3	3	3
3	2	3	2	2	2	3	3
4	2	3	2	1	4	3	3
3	2	3	2	2	2	3	2
4	2	3	1	1	2	2	3
3	3	2	3	2	2	2	3
99	99	99	2	1	3	3	3
4	1	4	1	1	1	1	1
4	3	3	3	1	4	4	4
4	1	4	2	1	3	4	4
4	1	4	1	1	4	4	4
3	1	4	2	1	4	4	4
3	2	4	2	1	4	4	4
4	3	3	2	1	99	1	4
3	1	4	1	1	3	4	3
4	1	4	1	1	4	4	1
3	1	4	2	1	4	4	3
4	1	4	2	1	3	4	3
4	1	1	1	1	2	2	1
4	1	4	1	1	4	4	1
3	2	4	2	1	3	2	2
2	3	99	3	99	3	3	3
4	1	4	1	1	2	99	4
4	1	4	1	1	4	4	4
4	2	4	1	1	1	1	99
4	1	4	1	1	1	1	1
4	2	4	1	1	3	4	3
3	3	3	1	1	99	99	99
4	1	4	1	1	4	4	4
4	1	4	1	1	4	4	99
3	2	3	2	2	3	3	3
4	1	4	2	1	3	3	3
4	1	4	1	1	4	4	4
2	3	3	2	2	3	3	3
4	1	4	1	1	3	3	3
3	2	3	4	1	3	4	2
3	2	3	1	1	3	3	2
99	99	99	99	1	4	4	4
3	1	3	2	1	3	3	2
4	1	4	1	1	4	4	4
99	99	99	99	1	2	2	4
4	1	4	1	1	3	3	3
3	3	4	2	1	3	3	4
2	2	3	3	2	3	2	4
4	2	4	1	1	3	4	3
4	2	4	2	2	3	3	4
4	1	4	1	1	99	99	99

4	1	4	1	1	3	4	3
4	1	4	1	1	4	4	4
3	3	3	3	1	4	4	3
4	1	4	1	1	4	4	4
4	2	4	3	1	3	2	3
99	99	99	99	99	99	99	99
4	1	4	1	1	4	4	4
3	2	4	3	1	3	3	3
4	1	4	2	4	4	4	3
2	2	3	3	1	1	1	2
3	1	4	1	1	3	3	2
3	2	3	2	1	3	3	3
4	99	4	2	1	99	99	99
3	2	3	2	2	3	3	3
2	2	3	3	1	4	4	4
2	2	2	2	2	3	3	3
2	3	3	2	2	3	2	3
2	3	4	2	1	4	4	4
3	2	4	1	1	4	4	4
3	2	3	3	1	3	3	3
2	2	4	2	1	2	2	2
2	2	3	1	1	3	3	3
4	2	4	1	1	2	2	3
4	2	4	3	1	3	3	3
4	1	4	1	1	4	4	4
2	2	2	2	2	2	2	2
4	2	3	2	2	3	3	2
3	99	3	2	2	4	2	4
2	2	3	3	1	3	4	4
3	2	4	99	2	2	2	3
3	2	4	2	1	2	2	1
2	2	3	2	1	3	2	2
3	2	3	2	1	4	4	4
3	1	3	2	1	99	99	99
4	1	4	1	1	4	4	4
4	1	4	1	1	3	4	2
99	99	99	99	99	99	99	99
4	1	4	1	1	1	1	1
2	99	3	2	2	4	4	4
4	1	4	1	1	4	4	3
3	2	3	3	2	3	2	3
3	1	4	1	1	3	3	4
4	2	4	2	1	3	2	3
1	1	1	1	1	1	1	1
3	2	4	2	2	4	4	4
4	1	4	1	1	4	4	4
2	3	3	2	4	2	3	4
3	2	3	2	2	3	3	2

4	2	4	2	1	2	3	3
3	2	3	2	1	3	3	3
4	2	1	1	1	3	3	4
3	2	3	1	1	3	3	99
4	3	99	2	1	4	4	99
3	2	4	2	1	99	3	2
4	3	4	2	1	2	1	2
3	2	3	2	1	4	3	2
3	2	3	2	2	2	2	2
4	1	1	1	1	2	2	2
3	3	4	3	1	4	3	2
4	1	4	1	1	3	3	3
4	1	4	1	1	4	4	3
4	1	4	1	1	4	4	3
4	99	4	3	1	4	4	99
4	1	4	1	1	4	4	1
4	1	4	1	1	4	4	4
4	1	4	1	1	3	3	3
3	2	3	1	1	2	3	2
4	99	99	1	1	4	4	99
4	99	4	1	1	99	99	99
3	99	99	2	1	3	3	3
4	1	4	1	1	99	4	4
99	99	99	99	99	99	99	99
1	99	4	1	1	1	1	2
3	2	3	2	2	3	3	3
3	1	4	1	1	4	4	4
3	3	3	2	2	2	3	4
3	1	4	2	1	3	2	3
3	99	99	99	2	3	2	3
3	3	4	2	2	3	3	3
4	1	4	1	1	4	4	4
4	2	4	2	1	3	3	2
3	2	3	2	1	4	3	3
2	99	3	2	2	2	2	3
3	2	4	2	1	3	3	99
3	2	3	1	1	3	3	99
99	99	99	99	99	99	99	99
3	2	3	2	2	2	2	99
4	1	4	1	1	2	4	4
3	1	4	1	1	3	3	4
4	2	4	2	1	4	4	99
4	2	4	1	1	4	4	4
3	3	4	1	1	1	2	3
2	2	3	2	1	4	4	4
3	1	4	1	1	3	4	3
4	1	2	2	1	3	3	3
3	3	4	2	1	1	2	3

99	99	99	99	99	99	99	99
2	3	2	2	3	2	2	3
4	1	4	1	1	4	4	4
3	99	99	2	1	3	3	3
3	2	3	3	2	3	3	3
3	99	3	2	2	99	99	3
99	99	4	1	1	4	4	99
3	2	3	2	2	3	2	3
3	2	3	2	2	3	2	3
3	2	3	3	2	3	3	3
4	1	4	1	1	3	4	4
4	1	4	2	1	3	3	99
3	2	3	3	2	3	3	3
99	1	3	2	1	3	3	4
2	2	3	3	1	2	3	2
4	1	4	1	1	4	4	4
4	2	4	1	1	3	3	4
4	2	4	1	1	3	4	4
1	4	3	99	2	1	2	3
3	1	4	3	1	2	2	3
4	1	4	1	1	4	4	4
3	2	1	1	1	2	3	4
99	99	99	99	99	99	99	99
4	2	3	2	2	3	3	3
3	1	4	1	1	3	3	3
1	1	4	1	1	4	4	4
4	2	1	1	1	2	3	3
3	2	3	2	1	99	99	99
3	2	3	2	2	3	3	3
3	2	2	1	1	4	4	2
3	3	3	2	2	3	3	3
3	1	4	1	1	4	4	4
4	1	1	1	1	1	1	1
4	2	4	2	1	2	2	2
4	1	4	1	1	4	4	4
4	2	4	2	1	2	2	2
3	3	3	3	2	3	2	2
2	2	99	1	1	3	3	3
4	1	4	1	1	4	4	4
3	1	3	99	3	4	4	3
4	1	4	1	1	4	4	4
1	1	1	1	1	4	1	4
4	1	4	1	1	3	3	4
4	1	1	1	1	4	4	4
3	2	4	1	1	2	3	2
4	4	4	4	4	4	4	4
99	99	99	2	2	99	99	99
2	1	3	1	1	2	2	3

2	3	4	3	1	4	4	3
1	3	3	3	2	4	1	2
3	2	2	2	2	2	2	2
3	1	3	2	1	4	4	3
4	2	4	2	1	4	3	2
4	1	4	2	1	4	4	4
4	1	4	1	1	4	4	4
3	1	4	2	1	3	4	3
4	99	99	1	1	3	4	4
4	1	99	1	1	4	3	4
4	1	4	1	1	4	4	4
3	2	3	1	1	99	3	3
4	1	4	1	1	2	4	4
2	2	2	2	2	2	2	2
3	2	3	2	1	3	3	3
3	2	3	99	2	2	1	3
3	2	3	2	2	3	2	3
3	99	4	99	1	3	4	4
99	1	99	1	1	4	4	99
4	1	4	1	1	3	4	4
4	1	4	1	1	4	4	4
3	2	3	2	1	99	99	99
3	2	4	2	1	2	2	3
3	99	3	1	1	4	4	3
3	2	4	2	2	4	3	3
2	3	3	3	2	3	4	4
3	99	4	2	1	3	4	2
4	1	4	1	1	4	4	4
99	99	99	99	99	2	2	3
3	3	99	2	1	3	3	2
3	3	3	3	2	4	4	3
4	4	4	2	1	2	2	4
4	1	4	1	1	3	3	4
4	2	4	2	1	3	3	3
4	1	1	1	1	4	4	4
4	1	4	1	1	1	2	1
2	2	4	1	1	4	4	2
3	2	3	2	1	4	4	4
99	99	99	99	99	4	4	3
4	1	4	1	1	1	1	1
3	1	4	1	1	4	4	3
4	1	4	1	1	4	4	4
4	1	99	1	1	3	3	99
3	2	3	3	1	3	3	3
4	1	1	1	1	1	1	2
4	2	4	2	1	4	4	4
4	1	4	1	1	4	4	4
2	4	2	3	2	2	2	4

1	3	2	3	3	1	1	4
3	3	3	2	99	3	1	2
4	2	4	2	1	2	2	3
2	2	3	3	2	3	3	4
3	3	4	3	3	3	3	4
4	1	4	1	1	4	4	4
3	2	3	2	1	3	3	4
4	99	3	99	1	3	3	3
3	2	99	2	2	3	3	3
3	2	3	2	1	3	3	3
2	2	3	3	2	3	2	2
3	3	4	3	3	4	4	4
2	3	3	3	2	4	4	4
2	2	2	2	2	3	3	3
3	2	3	3	2	3	3	3
4	3	3	3	3	4	2	2
3	2	3	2	1	3	4	4
3	2	3	2	1	3	3	3
2	2	2	3	2	2	2	3
3	99	3	99	99	99	99	99
2	3	1	4	3	1	1	2
4	2	4	1	1	3	4	4
3	2	99	1	1	3	3	4
4	2	3	2	1	4	4	4
4	99	4	1	1	4	4	4
3	2	3	2	2	2	2	2
4	1	4	1	1	4	4	4
4	1	4	1	1	4	4	4
99	3	4	99	2	1	2	1
4	1	4	1	1	99	99	99
4	1	4	1	1	4	4	4
4	1	4	3	1	4	3	4
4	2	4	2	1	2	3	3
3	2	3	2	2	3	2	2
1	99	99	1	1	3	4	2
99	99	99	99	99	4	4	4
4	2	3	3	2	4	4	4
4	1	4	1	1	4	4	4
2	3	3	2	2	3	3	3
2	3	99	3	1	3	4	2
3	1	3	2	1	1	1	4
3	2	4	2	1	4	4	3
99	99	99	99	99	4	4	99
4	1	4	1	1	4	4	4
4	1	4	1	1	4	4	2
4	1	4	1	1	3	3	4
4	2	3	2	1	4	4	4
3	2	3	2	2	3	3	2

4	1	4	1	1	3	3	3
3	3	3	3	2	4	4	3
3	2	3	2	2	3	3	3
1	1	1	1	1	1	1	2
4	2	4	1	1	2	2	2
4	99	4	1	1	4	4	4
3	2	2	2	2	2	2	2
4	1	4	1	1	4	4	4
4	3	4	1	1	3	3	1
3	2	3	2	2	3	4	3
2	3	3	3	2	3	3	2
3	2	3	2	2	3	3	3
4	99	4	1	1	4	4	4
2	3	2	1	1	4	99	4
4	2	3	2	1	2	3	3
4	1	4	1	1	4	4	4
2	2	3	3	3	3	3	4
99	2	3	2	2	4	4	4
4	2	3	2	2	2	2	2
4	1	4	1	1	4	4	4
1	1	4	2	1	4	3	1
2	3	4	3	2	3	3	4
4	3	4	1	1	1	1	1
2	1	4	2	1	1	1	1
3	2	3	2	1	3	3	3
3	3	3	1	1	3	3	3
2	3	3	2	1	3	3	3
2	1	1	4	4	4	2	4
4	1	4	1	1	99	99	99
4	1	4	1	1	4	4	4
3	1	4	1	1	2	2	1
4	1	4	1	1	4	4	4
4	2	4	3	1	3	4	4
2	2	3	2	2	3	3	3
4	1	4	1	1	4	4	99
3	2	3	3	1	2	3	3
4	4	4	2	2	1	2	2
3	2	3	2	2	4	4	3
3	2	3	2	2	2	2	3
3	2	3	2	2	2	2	2
3	2	4	1	1	4	4	4
3	1	3	2	1	4	3	4
4	1	4	1	1	4	4	4
4	2	3	1	1	3	3	3
2	2	2	2	2	3	3	3
3	2	3	2	2	4	4	4
4	3	4	3	2	2	2	2
3	1	4	1	2	3	3	3

3	1	4	2	1	4	4	4
4	2	3	2	2	4	4	4
2	1	4	2	2	4	4	4
4	1	4	1	1	3	4	4
3	1	4	1	1	4	4	4
3	2	3	2	1	4	4	4
4	1	4	1	1	1	3	4
3	1	3	2	1	3	4	4
4	1	4	1	1	4	4	4
2	2	3	2	2	99	3	3
3	2	3	2	1	1	3	2
3	2	4	2	1	3	4	3
3	1	4	2	1	1	2	3
2	2	2	3	2	2	1	3
4	2	4	1	1	3	3	3
4	1	4	1	1	1	1	1
2	2	3	2	1	4	4	4
2	2	3	2	2	2	2	2
99	99	99	99	3	99	99	99
1	4	3	4	3	3	2	2
4	1	4	1	1	4	4	4
1	3	2	3	4	3	3	4
3	99	3	2	1	3	3	3
4	4	4	4	1	1	1	2
4	99	4	99	1	4	4	2
3	99	3	2	1	3	3	2
2	3	2	3	2	3	3	3
3	1	1	1	1	3	3	3
2	3	2	3	3	2	2	99
2	2	2	2	2	2	3	3
99	99	99	99	99	99	99	99
3	99	3	2	1	3	3	3
1	1	1	1	1	2	2	2
3	3	4	2	1	99	3	99
3	1	4	2	2	4	4	99
3	1	4	1	1	4	3	4
4	1	4	2	1	3	3	3
3	2	3	3	2	3	2	3
3	1	3	2	1	2	2	2
3	1	3	2	1	2	3	3
4	1	4	1	1	4	4	4
99	99	99	99	1	4	4	99
4	1	4	1	1	4	4	4
4	1	4	3	1	4	4	4
4	3	4	1	1	1	3	1
4	1	4	1	1	4	4	4
4	1	4	1	1	4	4	4
2	3	4	4	4	2	3	3

4	1	4	1	1	4	4	99
99	2	3	99	99	3	2	99
4	1	1	1	1	3	4	3
3	2	3	2	2	3	3	3
3	2	3	2	2	3	2	3
4	99	99	2	2	99	99	99
3	2	2	3	1	4	4	4
3	2	4	1	1	4	4	4
2	3	3	2	2	3	3	2
4	1	4	1	1	1	4	4
4	1	1	1	1	2	2	2
3	1	4	2	1	1	4	1
4	1	1	1	1	4	4	4
3	2	3	3	1	3	3	3
2	3	3	3	2	3	3	3
4	1	4	1	1	4	4	4
3	2	4	1	1	2	2	3
4	2	3	2	1	2	2	2
4	4	1	1	1	3	3	3
4	2	4	2	1	1	2	3
4	2	4	1	1	3	3	4
3	3	3	2	1	4	4	2
2	3	3	2	2	3	3	2
3	3	3	3	3	3	3	3
4	1	3	1	1	4	4	4
3	2	3	2	2	3	3	4
4	1	4	2	4	99	99	99
1	1	4	1	1	3	3	4
1	2	3	3	3	1	1	2
2	2	3	2	2	2	2	3
4	1	4	1	2	4	4	4
3	2	3	2	1	4	4	4
3	1	1	1	1	4	4	4
4	1	4	1	1	2	3	4
3	1	3	1	1	4	4	4
2	2	3	99	2	2	3	3
3	3	3	2	1	2	3	3
3	2	3	2	2	3	3	3
4	1	4	1	1	3	3	3
4	2	4	1	1	1	1	4
2	2	2	2	2	3	3	3
2	3	3	3	2	3	3	3
1	2	2	3	2	3	2	3
4	1	4	1	1	3	4	4
2	1	3	4	1	3	3	3
4	1	4	1	1	3	4	4
2	2	3	2	1	3	3	3
3	2	3	1	1	3	4	3

4	1	4	1	1	4	4	4
4	2	4	2	1	3	3	3
4	2	3	2	1	4	3	4
2	2	4	2	2	4	4	4
3	1	1	1	1	4	4	4
4	2	3	2	2	4	4	4
3	3	1	1	1	99	99	99
3	2	2	2	2	2	3	2
3	1	4	1	1	3	3	3
3	99	4	2	1	4	4	99
2	3	3	2	2	3	3	3
2	2	3	2	1	4	4	3
3	3	2	2	2	3	3	3
4	1	3	1	3	3	3	1
2	2	3	3	2	4	4	4
4	1	4	1	1	4	4	1
3	2	3	2	2	2	2	2
3	4	3	3	1	99	3	3
3	2	3	2	3	3	3	3
3	2	3	1	1	1	3	1
4	3	4	2	1	3	3	4
4	1	4	1	1	4	4	4
4	2	4	1	1	4	4	4
1	3	3	1	1	4	1	1
2	3	3	3	2	3	3	3
4	2	3	2	1	3	3	3
3	2	3	2	1	2	3	1
4	1	4	1	1	3	3	3
4	1	4	1	1	4	4	4
4	1	4	1	1	1	3	4
4	2	4	1	1	3	99	99
4	1	4	1	1	4	4	4
4	1	4	1	1	4	1	1
4	1	4	2	1	3	3	4
2	2	3	2	1	3	4	3
3	2	3	2	2	3	3	3
3	2	4	2	1	4	4	3
4	1	3	1	1	2	3	3
3	3	3	3	2	2	2	3
2	2	4	2	2	2	2	4
1	4	3	3	2	3	3	3
4	1	4	1	1	4	4	4
1	1	1	1	1	4	4	4
4	2	3	2	1	4	4	2
4	2	4	1	1	4	4	99
3	3	3	2	1	2	2	2
3	2	3	2	1	3	3	3
3	2	3	2	2	3	3	2

3	3	3	2	1	2	2	3
4	1	4	1	1	4	3	3
3	2	4	2	1	3	4	4
4	99	3	2	1	3	3	99
4	1	4	1	1	4	4	4
4	2	3	2	1	3	3	2
3	1	3	3	2	4	4	4
4	3	4	4	99	4	4	4
4	1	4	1	1	4	4	4
4	2	4	1	2	4	4	4
3	2	4	2	1	3	3	4
4	1	3	1	1	3	3	3
2	1	4	1	1	2	4	3
1	1	4	1	1	4	4	1
2	3	99	99	2	4	3	3
3	2	2	2	2	3	3	3
3	2	4	1	1	3	4	2
3	1	4	2	1	3	2	3
4	1	3	2	1	4	4	3
2	2	3	99	1	4	4	4
4	1	4	1	1	1	1	3
4	1	4	1	1	4	4	4
4	1	4	1	1	3	3	4
4	1	4	1	1	4	4	4
3	3	3	3	1	3	4	3
2	3	3	3	2	4	4	3
2	2	4	99	3	4	4	3
1	4	3	3	2	3	4	4
3	2	4	2	2	4	4	4
3	2	3	2	2	3	4	3
4	1	4	1	1	4	4	4
3	2	3	1	1	3	4	4
4	2	4	1	1	4	3	3
3	1	99	2	1	3	3	3
3	1	4	2	1	3	4	3
99	2	99	3	2	2	2	2
3	99	3	2	2	2	2	2
4	1	4	1	1	4	4	4
4	1	3	1	1	3	3	4
3	3	2	3	1	4	4	4
1	1	1	1	1	3	3	99
4	1	4	1	1	3	2	3
3	4	1	4	1	4	4	4
3	3	3	2	2	2	3	3
3	2	3	2	2	2	2	3
4	99	99	99	1	4	4	1
4	1	1	1	1	4	4	4
4	2	4	1	1	4	4	4

4	1	4	1	1	4	4	4
1	3	2	1	2	2	2	3
3	2	2	3	2	3	3	3
4	2	3	2	4	1	1	2
4	2	4	2	1	4	4	4
3	2	3	2	1	3	3	3
1	1	99	99	99	99	99	99
3	2	3	2	2	2	3	2
4	1	1	1	1	4	4	4
1	1	4	1	1	4	4	3
2	2	3	99	2	2	2	3
1	1	2	4	4	2	4	4
3	3	3	2	3	4	4	4
3	3	3	2	2	3	3	2
4	1	4	1	1	4	4	4
2	3	4	2	1	4	4	4
2	2	3	2	2	4	4	4
3	1	4	2	1	4	4	3
4	3	3	3	1	3	3	4
3	3	3	2	2	4	4	4
4	2	4	3	1	4	4	4
4	3	3	1	2	3	3	3
4	2	4	2	1	4	4	3
2	2	99	2	2	2	2	3
1	1	3	1	1	3	3	4
3	3	3	2	2	2	2	2
4	2	4	2	1	2	2	2
4	1	4	1	1	4	4	4
3	3	3	3	2	3	3	3
1	1	4	1	1	4	2	1
4	3	3	2	1	3	3	3
3	2	3	2	1	2	2	2
2	1	3	1	1	3	3	2
4	1	4	1	1	3	4	4
3	1	1	1	1	3	3	3
3	2	3	2	2	3	3	3
4	1	4	2	2	4	3	4
3	2	3	3	2	3	3	3
3	2	4	2	1	3	4	4
4	2	3	2	1	4	4	4
4	1	3	1	1	3	4	4
2	2	3	3	2	4	4	4
2	3	3	3	2	2	2	3
4	2	3	2	1	4	4	4
2	3	3	3	3	4	4	4
2	3	2	3	3	3	3	3
4	1	4	1	99	1	2	2
3	2	3	2	1	3	3	3

2	2	3	2	2	3	4	3
4	2	3	3	3	2	2	3
4	1	4	2	1	4	4	4
3	2	3	2	2	3	3	3
1	1	1	1	1	4	4	4
4	2	3	1	1	3	3	3
4	3	4	3	1	2	2	3
4	1	4	1	1	3	3	3
2	3	4	1	1	4	4	3
2	3	4	1	1	3	4	3
4	1	4	2	2	4	4	4
99	99	99	99	99	3	3	4
3	2	3	2	1	3	3	3
4	2	3	2	1	4	4	3
2	3	2	2	1	2	2	4
2	2	3	2	2	2	2	99
3	2	3	1	1	4	4	4
2	3	3	2	2	3	3	3
4	1	4	1	1	4	4	1
4	99	4	2	1	2	99	3
1	3	3	3	1	1	1	1
3	2	3	2	1	3	3	3
99	2	99	2	2	2	2	99
3	1	3	2	1	4	4	4
4	1	4	1	1	1	99	99
4	1	4	1	1	4	4	4
3	1	4	1	1	3	3	2
4	99	99	2	2	3	3	4
3	2	2	2	1	4	4	4
4	1	4	1	1	4	4	4
4	2	4	2	1	2	3	2
3	3	3	2	1	3	3	3
3	3	3	3	1	1	1	4
4	4	4	1	1	4	4	4
2	2	4	2	1	4	4	2
3	2	4	1	1	3	3	4
2	2	3	2	2	2	2	2
3	1	4	1	1	4	4	4
1	1	1	1	1	3	3	2
4	2	3	1	2	3	3	3
4	1	4	2	1	3	2	2
99	99	99	99	1	4	99	99
4	2	2	1	1	3	3	4
2	2	4	3	1	4	4	4
4	2	2	2	2	3	3	3
4	3	4	3	1	2	3	2
4	1	4	1	1	4	4	4
99	99	99	99	99	3	3	99

4	3	3	2	1	4	4	4
2	3	3	3	1	3	3	3
3	3	4	3	1	4	4	3
4	1	4	2	1	4	4	4
1	1	4	1	1	4	4	4
3	2	4	1	1	4	4	3
4	1	3	1	1	4	4	3
3	2	3	2	2	3	3	3
4	1	4	1	1	4	4	4
4	1	4	1	1	4	4	3
4	1	4	1	1	1	1	1
99	3	3	2	1	4	4	4
4	1	4	1	1	3	4	3
4	1	4	1	1	1	1	4
4	2	3	2	2	4	3	4
3	2	2	2	2	3	3	3
2	3	3	3	3	3	3	4
3	2	3	2	2	3	3	3
99	99	99	99	99	99	99	99
4	1	4	1	1	4	4	4
3	2	4	2	1	1	1	2
4	1	4	1	1	4	4	4
4	2	3	3	2	3	3	3
4	2	4	2	1	99	99	99
2	3	2	1	1	3	3	1
2	1	4	1	1	4	2	2
4	4	3	1	1	4	2	3
4	1	4	2	1	2	2	2
3	2	2	1	2	2	2	2
2	2	3	2	2	2	2	3
3	2	3	2	1	4	4	4
2	2	3	2	1	2	3	3
3	2	3	2	1	4	4	3
2	3	2	3	3	3	3	3
4	2	4	1	1	4	4	4
3	2	4	2	2	4	4	4
3	2	2	2	2	4	4	4
2	2	4	2	2	3	3	3
2	2	2	3	2	3	3	3
4	1	4	1	1	4	4	4
2	2	3	2	2	2	1	2
99	2	3	3	2	4	2	4
1	2	99	4	3	99	4	2
4	1	4	1	1	4	4	4
3	2	3	1	1	4	4	4
3	2	4	1	1	2	2	3
3	2	3	2	1	4	4	4
4	1	4	1	1	4	4	4

4	1	4	1	1	2	2	3
2	1	3	3	2	2	1	3
4	1	4	1	1	4	4	4
4	1	4	1	1	#NULL!	#NULL!	#NULL!
4	2	4	1	1	#NULL!	#NULL!	#NULL!
4	2	3	1	1	#NULL!	#NULL!	#NULL!
1	1	1	1	1	#NULL!	#NULL!	#NULL!
2	3	4	2	2	#NULL!	#NULL!	#NULL!
2	3	3	4	4	#NULL!	#NULL!	#NULL!
2	3	3	4	4	#NULL!	#NULL!	#NULL!
2	2	4	2	2	#NULL!	#NULL!	#NULL!
3	2	1	2	1	#NULL!	#NULL!	#NULL!
2	2	4	2	2	#NULL!	#NULL!	#NULL!
3	2	3	2	2	#NULL!	#NULL!	#NULL!
1	2	3	2	2	#NULL!	#NULL!	#NULL!
2	2	3	2	2	#NULL!	#NULL!	#NULL!
4	2	4	2	2	#NULL!	#NULL!	#NULL!
3	2	3	2	2	#NULL!	#NULL!	#NULL!
3	1	4	1	1	#NULL!	#NULL!	#NULL!
3	4	4	2	2	#NULL!	#NULL!	#NULL!
1	4	3	3	2	#NULL!	#NULL!	#NULL!
3	1	4	2	1	#NULL!	#NULL!	#NULL!
1	2	4	1	2	#NULL!	#NULL!	#NULL!
1	1	1	1	1	#NULL!	#NULL!	#NULL!
2	2	3	2	2	#NULL!	#NULL!	#NULL!
4	2	4	1	2	#NULL!	#NULL!	#NULL!
3	3	3	3	1	#NULL!	#NULL!	#NULL!
4	1	4	1	1	#NULL!	#NULL!	#NULL!
4	2	3	2	2	#NULL!	#NULL!	#NULL!
4	4	4	4	4	#NULL!	#NULL!	#NULL!
4	1	4	1	1	#NULL!	#NULL!	#NULL!
2	3	3	3	3	#NULL!	#NULL!	#NULL!
1	3	3	3	3	#NULL!	#NULL!	#NULL!
3	2	3	2	2	#NULL!	#NULL!	#NULL!
2	1	4	2	1	#NULL!	#NULL!	#NULL!
1	2	4	2	2	#NULL!	#NULL!	#NULL!
4	4	2	2	2	#NULL!	#NULL!	#NULL!
2	1	4	2	1	#NULL!	#NULL!	#NULL!
2	1	3	2	2	#NULL!	#NULL!	#NULL!
3	3	3	3	3	#NULL!	#NULL!	#NULL!
4	4	4	4	4	#NULL!	#NULL!	#NULL!
3	4	3	3	2	#NULL!	#NULL!	#NULL!
2	3	4	2	1	#NULL!	#NULL!	#NULL!
2	3	2	3	2	#NULL!	#NULL!	#NULL!
1	1	4	1	1	#NULL!	#NULL!	#NULL!
2	4	4	3	1	#NULL!	#NULL!	#NULL!
3	3	3	1	1	#NULL!	#NULL!	#NULL!
4	3	4	1	1	#NULL!	#NULL!	#NULL!

4	1	4	1	1	#NULL!	#NULL!	#NULL!
3	2	4	2	1	#NULL!	#NULL!	#NULL!
3	2	3	2	2	#NULL!	#NULL!	#NULL!
2	2	4	2	1	#NULL!	#NULL!	#NULL!
3	2	3	2	2	#NULL!	#NULL!	#NULL!
4	1	4	1	1	#NULL!	#NULL!	#NULL!
2	3	4	3	2	#NULL!	#NULL!	#NULL!
1	1	1	1	1	#NULL!	#NULL!	#NULL!
4	4	4	4	4	#NULL!	#NULL!	#NULL!
1	4	4	3	3	#NULL!	#NULL!	#NULL!
4	1	1	1	1	#NULL!	#NULL!	#NULL!
#NULL!	3	#NULL!	3	3	#NULL!	#NULL!	#NULL!
#NULL!	#NULL!	#NULL!	#NULL!	#NULL!	#NULL!	#NULL!	#NULL!
2	1	4	3	1	#NULL!	#NULL!	#NULL!
1	3	2	2	2	#NULL!	#NULL!	#NULL!
1	1	1	1	1	#NULL!	#NULL!	#NULL!
2	3	4	3	1	#NULL!	#NULL!	#NULL!
3	2	2	4	1	#NULL!	#NULL!	#NULL!
2	1	4	2	1	#NULL!	#NULL!	#NULL!
4	2	4	3	1	#NULL!	#NULL!	#NULL!
3	2	3	2	#NULL!	#NULL!	#NULL!	#NULL!
4	3	4	4	2	#NULL!	#NULL!	#NULL!
2	2	2	2	2	#NULL!	#NULL!	#NULL!
2	2	3	2	2	#NULL!	#NULL!	#NULL!
4	2	3	2	2	#NULL!	#NULL!	#NULL!
#NULL!	2	4	1	1	#NULL!	#NULL!	#NULL!
2	3	4	3	2	#NULL!	#NULL!	#NULL!
4	3	2	3	1	#NULL!	#NULL!	#NULL!
3	3	4	2	1	#NULL!	#NULL!	#NULL!
2	3	1	1	1	#NULL!	#NULL!	#NULL!
2	3	1	3	1	#NULL!	#NULL!	#NULL!
3	2	3	1	1	#NULL!	#NULL!	#NULL!
3	2	4	3	1	#NULL!	#NULL!	#NULL!
1	1	1	1	1	#NULL!	#NULL!	#NULL!
2	3	4	2	1	#NULL!	#NULL!	#NULL!
3	2	3	1	2	#NULL!	#NULL!	#NULL!
3	2	3	2	1	#NULL!	#NULL!	#NULL!
3	2	3	2	2	#NULL!	#NULL!	#NULL!
4	1	4	1	1	#NULL!	#NULL!	#NULL!
2	1	4	1	1	#NULL!	#NULL!	#NULL!
4	3	4	3	1	#NULL!	#NULL!	#NULL!
3	3	4	3	1	#NULL!	#NULL!	#NULL!
3	3	4	2	1	#NULL!	#NULL!	#NULL!
3	2	3	2	2	#NULL!	#NULL!	#NULL!
4	3	4	3	1	#NULL!	#NULL!	#NULL!
2	1	3	2	2	#NULL!	#NULL!	#NULL!
4	1	4	3	1	#NULL!	#NULL!	#NULL!
2	2	2	2	2	#NULL!	#NULL!	#NULL!

2	2	4	1	1	#NULL!	#NULL!	#NULL!
4	2	3	2	1	#NULL!	#NULL!	#NULL!
3	1	3	2	1	#NULL!	#NULL!	#NULL!
1	1	1	1	1	#NULL!	#NULL!	#NULL!
4	1	4	1	1	#NULL!	#NULL!	#NULL!
4	1	1	1	1	#NULL!	#NULL!	#NULL!
2	1	4	2	1	#NULL!	#NULL!	#NULL!
1	1	3	1	1	#NULL!	#NULL!	#NULL!
1	1	4	1	1	#NULL!	#NULL!	#NULL!
2	2	4	2	1	#NULL!	#NULL!	#NULL!
4	1	3	2	1	#NULL!	#NULL!	#NULL!
4	1	4	1	1	#NULL!	#NULL!	#NULL!
4	1	4	1	1	#NULL!	#NULL!	#NULL!
4	2	3	2	1	#NULL!	#NULL!	#NULL!
2	2	4	3	3	#NULL!	#NULL!	#NULL!
4	1	4	1	1	#NULL!	#NULL!	#NULL!
#NULL!	#NULL!	#NULL!	#NULL!	#NULL!	#NULL!	#NULL!	#NULL!
4	1	4	2	1	#NULL!	#NULL!	#NULL!
2	#NULL!	4	3	2	#NULL!	#NULL!	#NULL!
2	1	4	1	1	#NULL!	#NULL!	#NULL!
3	1	4	1	1	#NULL!	#NULL!	#NULL!

pregd_pre	pccomma_pre	pccommb_pre	pccommc_pre	pccommd_pre	pccomme_pre	pccommf_pre
99	0	0	0	0	0	0
2	0	0	0	0	0	0
4	0	0	0	0	0	0
4	0	0	0	1	0	0
1	0	0	0	0	0	1
99	99	99	99	99	99	99
4	0	0	2	0	99	0
2	0	0	0	1	0	1
99	99	99	99	99	99	99
1	1	0	0	2	1	1
1	0	0	0	0	0	0
2	1	0	0	1	0	1
2	0	0	0	0	0	0
1	0	0	0	0	0	0
1	0	0	0	0	0	0
3	1	1	0	1	1	0
99	0	0	0	0	0	0
2	0	0	0	99	0	0
99	2	1	1	1	2	1
2	1	0	0	2	1	99
99	99	99	99	99	99	99
1	0	0	0	0	0	0
99	0	0	0	0	0	0
4	2	0	0	2	0	2
4	0	0	0	0	0	0
3	0	0	0	0	0	0
2	0	0	0	0	0	0
4	0	0	0	0	0	0
4	0	0	0	0	0	0
3	1	1	0	1	0	0
3	0	0	0	2	0	0
1	2	0	0	2	0	1
3	0	0	0	1	0	0
3	0	0	0	0	0	0
99	0	0	99	0	0	99
4	1	0	1	2	0	1
2	0	0	0	1	0	0
4	0	0	0	1	0	0
4	0	0	0	1	0	1
2	1	1	0	2	1	0
4	0	0	0	2	0	2
99	0	0	0	1	0	0
4	1	1	0	0	0	0
3	0	0	0	0	0	0
2	1	0	2	2	1	0
4	1	0	1	1	1	2
2	0	0	0	0	0	0

4	1	1	1	0	0	0
4	0	0	0	1	0	1
4	2	0	2	2	1	0
3	1	1	1	2	0	1
3	0	0	0	2	0	0
2	1	2	1	0	0	0
3	0	0	0	1	0	0
2	1	0	1	1	0	0
2	0	0	0	1	0	1
1	1	0	0	2	0	1
3	0	0	0	0	0	0
4	0	0	0	1	0	0
3	0	0	0	1	0	0
2	2	2	2	2	2	0
2	0	0	1	1	0	0
2	2	99	2	2	0	0
4	1	1	0	2	0	0
4	0	0	0	1	1	0
4	1	1	0	0	0	0
2	2	1	0	2	1	0
2	2	0	2	2	1	1
99	1	0	0	2	0	0
4	0	0	0	2	0	0
1	0	0	0	2	0	0
4	2	2	99	2	1	1
4	0	0	0	2	0	1
1	0	0	0	2	0	0
2	2	2	2	2	2	2
99	0	0	0	1	0	0
2	1	0	1	2	0	1
99	0	0	0	2	0	0
2	1	0	0	2	0	0
2	0	0	2	2	2	2
4	2	2	1	2	1	2
2	1	0	0	1	0	1
4	0	0	0	0	0	0
4	0	0	0	0	0	0
3	1	1	2	2	1	1
2	0	0	0	2	1	2
4	2	1	2	99	0	0
4	1	0	1	2	1	1
4	0	0	0	0	0	0
1	1	1	0	1	0	0
3	0	0	0	0	0	0
2	2	1	1	99	0	1
4	0	0	0	1	0	0
4	0	0	0	0	0	0
1	1	0	0	1	0	0

2	0	99	0	1	0	0
4	1	1	1	1	1	0
2	2	2	1	2	1	1
4	1	1	1	2	0	1
3	2	1	2	2	2	2
3	1	0	1	0	0	0
1	2	1	1	2	1	2
3	1	0	0	2	0	0
2	1	1	1	2	1	1
4	2	2	2	2	2	2
1	0	0	1	2	0	0
2	1	1	0	1	0	0
4	2	2	2	1	1	2
1	2	1	1	1	0	1
3	2	0	0	2	0	2
2	0	0	0	0	0	0
2	2	1	2	2	1	2
1	1	0	0	2	0	0
3	2	2	0	2	1	2
3	0	0	0	1	1	0
2	1	0	1	2	0	1
4	1	1	1	1	1	1
4	2	0	0	0	0	0
4	1	0	0	2	0	0
4	2	2	2	2	2	2
4	1	1	1	1	1	1
4	2	0	1	99	0	99
3	0	0	0	0	0	0
2	0	0	0	2	0	0
3	1	0	1	1	0	1
4	0	0	0	1	0	1
2	1	0	0	1	0	0
4	99	99	99	99	99	99
4	1	0	0	1	0	99
2	1	1	1	2	1	0
1	0	99	0	0	0	0
2	0	0	0	1	0	0
2	2	1	1	2	1	2
1	1	0	0	2	0	2
3	0	0	0	0	0	0
2	0	0	0	1	0	0
3	2	2	1	1	1	2
3	1	1	1	1	0	0
3	0	0	0	1	0	0
2	1	0	1	0	1	1
2	0	0	0	1	0	0
2	1	0	0	2	0	0
1	1	2	2	2	0	0

2	1	0	0	0	0	0
1	1	1	1	2	1	1
1	0	0	0	1	0	0
4	2	1	1	2	1	2
2	1	0	0	1	0	0
1	99	99	99	99	99	99
2	0	0	0	0	0	0
2	1	0	0	1	0	0
4	1	0	1	1	0	0
4	0	0	0	0	0	0
3	1	0	0	1	0	1
1	0	0	0	0	0	0
3	0	0	0	0	0	0
2	1	0	0	2	0	0
4	1	0	0	0	0	0
3	2	1	0	1	0	1
3	1	0	0	1	1	1
4	0	0	0	1	0	0
4	1	0	0	1	1	1
2	0	0	0	1	0	0
3	0	0	0	1	0	0
2	0	0	0	2	0	0
4	1	1	1	1	1	1
3	1	0	1	1	0	1
3	0	0	0	0	0	0
2	0	0	0	0	0	0
3	0	0	0	0	0	0
4	1	0	0	1	0	0
4	0	0	0	0	0	0
3	1	0	0	0	0	0
99	0	0	0	0	0	0
99	0	0	0	1	0	0
4	0	0	0	0	0	0
4	0	0	0	0	0	0
4	1	1	0	1	0	0
1	1	1	0	1	0	0
99	0	0	0	1	0	0
2	0	0	0	0	0	0
4	0	0	99	99	0	0
2	1	1	0	1	0	1
3	1	0	0	1	2	0
4	0	0	0	2	0	0
4	0	0	0	2	0	1
2	1	0	2	99	0	2
2	1	0	0	1	0	0
4	2	1	2	2	2	2
1	1	1	0	1	99	1
4	0	0	0	1	0	0

3	0	0	0	0	0	0
4	0	0	0	0	0	0
1	0	0	0	1	0	0
4	0	0	0	1	0	0
3	1	0	0	1	0	0
4	0	0	0	0	0	0
3	0	0	0	0	0	0
4	2	2	2	2	0	2
3	0	0	0	0	0	0
2	1	1	1	2	1	1
3	0	0	0	0	0	0
2	2	0	1	2	99	1
2	1	1	1	1	0	0
2	99	99	99	99	99	99
1	1	0	1	1	1	1
1	2	2	1	2	2	2
3	0	0	0	1	0	1
2	0	0	0	0	0	0
3	1	1	1	2	1	1
1	0	0	0	1	0	0
3	0	1	0	0	0	0
99	0	0	0	1	0	0
3	0	0	0	0	0	0
1	0	0	0	0	0	0
1	1	0	0	1	0	0
1	1	0	0	0	0	2
2	0	0	0	1	0	0
3	1	0	0	2	0	1
1	1	0	0	2	1	0
4	0	0	0	0	0	0
2	2	1	2	2	1	2
3	1	0	1	2	1	1
4	2	2	2	2	2	2
99	1	1	0	1	0	1
4	2	2	2	2	1	1
1	1	0	2	2	0	0
2	0	0	0	0	0	0
3	1	0	0	0	0	0
4	0	0	0	0	0	0
3	1	2	2	2	2	2
4	0	0	0	2	0	0
2	0	0	0	1	0	0
2	1	1	2	2	1	2
4	0	0	0	1	0	0
4	0	0	0	1	0	0
2	2	1	1	0	0	0
3	0	0	0	0	0	0
4	0	0	0	0	0	0

3	0	0	0	1	0	0
4	1	0	1	1	0	1
2	1	0	0	1	0	0
4	0	0	0	99	0	0
1	2	0	0	2	2	1
1	1	0	0	1	0	1
3	1	0	0	2	0	0
3	0	0	0	0	0	0
3	1	2	1	2	1	1
4	1	0	0	1	0	0
4	0	0	0	0	0	0
3	0	0	0	0	0	0
3	0	0	0	1	0	0
2	1	0	0	1	1	0
1	0	0	1	1	0	0
2	1	0	0	1	0	0
4	0	0	0	1	0	0
99	0	0	0	0	0	0
4	1	0	0	2	0	0
3	1	0	0	2	0	1
2	1	1	1	2	1	0
4	1	0	0	2	0	0
4	0	0	0	1	0	0
4	0	0	0	0	0	0
4	0	0	0	0	0	0
2	1	0	99	0	0	0
4	1	0	0	0	0	0
4	1	0	0	1	0	1
3	1	1	99	2	1	1
4	0	0	0	0	0	0
3	1	0	1	2	0	0
4	0	0	0	2	0	1
3	1	0	0	1	0	0
3	2	0	0	2	2	0
1	0	0	0	2	0	0
4	2	99	1	1	1	1
3	1	0	0	2	0	1
2	1	1	0	1	0	0
2	0	0	0	1	0	0
2	2	0	2	2	2	99
1	0	0	1	1	0	1
3	0	0	0	0	0	0
2	1	0	0	2	0	0
2	0	0	0	0	0	0
2	0	0	0	2	0	2
2	0	0	0	1	0	1
4	0	0	0	1	0	1
1	1	0	1	2	0	1

2	0	0	0	1	1	1
4	1	0	0	2	0	2
4	2	0	0	2	0	0
4	1	0	0	1	0	0
1	0	0	1	1	0	0
3	0	0	0	0	0	0
4	1	0	0	1	0	0
3	0	0	0	1	0	0
4	1	0	0	2	0	0
3	2	2	1	2	2	2
4	1	0	0	2	1	0
2	0	0	0	0	0	0
2	0	1	1	2	0	0
3	1	0	0	1	0	1
2	2	0	0	2	0	0
3	1	1	0	2	0	0
2	1	0	0	2	0	0
1	2	1	2	2	1	0
4	1	0	1	2	2	1
3	2	1	1	2	0	2
3	1	0	0	2	0	0
1	1	0	1	2	0	0
4	0	0	0	1	0	0
2	2	1	1	2	1	99
2	0	0	0	2	0	0
3	0	0	0	1	0	0
2	2	1	1	1	0	0
2	1	1	1	1	1	1
99	1	0	0	0	0	2
99	0	0	0	0	0	0
1	2	1	0	1	1	2
2	0	0	0	1	0	0
4	2	1	2	2	2	1
2	0	0	0	0	0	0
3	0	0	0	0	0	0
1	0	0	0	0	0	0
2	1	0	1	2	0	0
2	0	0	1	1	0	0
4	1	0	0	1	0	0
2	0	0	0	0	0	0
4	0	1	0	0	0	2
3	2	0	1	2	1	0
2	0	0	0	1	0	0
3	0	0	0	2	0	0
2	1	0	1	1	1	1
3	1	1	1	2	0	1
2	0	0	0	1	0	0
3	1	0	0	1	0	0

3	1	0	0	0	0	0
2	0	0	0	1	0	0
1	2	1	1	2	2	2
2	1	1	0	1	0	1
4	0	0	0	1	1	0
99	0	0	0	0	0	0
2	1	0	1	0	0	0
2	1	1	1	1	0	1
1	2	0	2	2	2	0
3	0	0	0	1	0	0
1	1	2	2	1	0	0
2	1	0	0	1	0	0
1	0	0	0	1	0	0
1	2	1	0	2	1	1
2	1	0	1	2	0	0
3	0	0	0	0	2	2
4	0	0	0	1	0	0
4	1	1	1	99	1	1
3	1	0	1	1	0	1
2	1	0	1	0	1	1
3	1	0	0	1	0	1
4	0	0	0	1	0	0
2	1	1	0	2	0	1
3	1	1	1	1	0	1
4	1	1	1	1	1	0
4	0	0	0	1	0	0
4	1	0	0	2	0	0
99	99	99	99	99	99	99
3	0	0	0	1	0	0
3	1	0	0	1	1	1
2	1	0	0	1	0	0
4	0	0	0	0	1	1
4	0	0	0	1	0	0
2	1	0	0	0	0	0
4	0	0	0	1	0	0
4	0	0	0	1	0	0
3	1	0	0	0	1	2
1	1	0	0	0	1	0
3	2	1	1	2	1	1
2	0	0	0	0	0	0
3	1	1	0	1	0	0
4	0	0	0	2	0	0
2	0	0	0	1	0	0
1	1	0	0	2	0	0
4	0	0	0	1	0	0
1	0	0	0	0	0	0
2	1	1	1	2	0	2
4	0	0	0	1	0	0

99	0	0	0	0	0	0
4	0	0	0	1	0	0
2	2	2	2	2	1	1
3	1	1	0	2	0	0
1	0	0	0	0	0	0
2	2	0	1	2	1	1
3	1	0	0	1	0	0
4	0	1	0	1	0	0
3	0	0	0	2	0	2
2	1	0	0	0	0	0
3	0	0	0	2	0	0
4	2	2	2	1	2	2
2	1	0	0	1	0	0
3	1	0	0	1	0	0
4	0	0	1	99	0	0
4	0	0	0	1	0	0
1	2	2	2	1	0	0
4	1	0	0	2	0	0
2	0	1	0	0	0	0
1	0	0	0	0	0	0
4	1	0	0	2	0	0
1	1	0	1	0	0	0
2	1	0	1	1	1	0
4	1	0	1	0	0	0
4	1	0	0	0	0	0
4	2	2	2	1	1	1
2	1	1	1	2	0	0
1	1	1	1	0	1	1
99	0	0	0	0	0	0
2	0	0	0	0	0	0
2	0	0	0	2	0	1
2	1	1	0	2	0	0
99	0	0	0	0	0	0
99	0	0	0	0	0	0
3	0	1	1	1	0	0
3	1	1	2	2	2	2
99	2	2	2	2	2	2
4	1	1	0	2	0	0
4	2	1	0	2	0	0
1	0	0	0	1	0	0
2	2	2	2	2	2	2
3	0	0	0	0	0	0
2	2	0	1	2	1	2
99	99	99	99	99	99	99
2	2	2	1	2	0	0
2	2	2	1	1	0	0
1	1	2	0	2	0	0
99	2	0	2	2	1	0

4	1	0	0	2	0	0
3	0	0	0	1	0	0
4	0	0	0	0	0	1
2	0	0	0	0	0	0
3	0	0	0	1	0	0
3	0	0	0	2	0	0
4	0	0	0	0	0	0
3	1	1	0	2	1	1
3	0	0	0	2	0	0
3	2	2	1	1	2	1
2	2	2	2	2	1	2
2	1	1	1	1	1	0
2	2	2	2	2	1	2
2	2	2	2	2	2	2
3	0	0	0	1	0	0
4	2	0	0	2	1	2
4	0	0	0	1	0	0
1	0	0	0	1	0	0
3	1	0	0	1	0	0
2	0	0	0	1	1	0
3	1	1	1	1	1	1
2	2	1	1	2	0	2
4	1	0	0	2	0	0
2	1	0	1	1	0	0
1	1	1	0	0	0	0
4	0	0	0	1	0	0
1	2	1	2	2	2	1
2	0	1	0	1	0	0
3	0	0	0	0	0	0
3	2	0	0	2	0	0
2	0	0	0	0	0	0
4	2	1	0	1	1	1
2	1	0	1	2	0	0
4	2	2	2	1	0	2
2	1	1	1	2	0	0
2	1	0	0	2	0	0
2	2	1	2	2	1	2
2	2	0	2	2	0	1
2	1	1	0	1	0	1
2	2	1	0	2	0	1
4	0	0	0	2	0	0
1	0	0	0	0	0	1
4	0	0	0	2	0	0
2	1	0	0	2	0	0
1	1	1	0	2	1	0
3	0	0	0	1	1	0
2	0	0	0	0	0	0
4	1	0	0	2	1	0

4	1	0	0	1	0	0
3	0	0	0	1	0	0
4	1	0	0	2	0	1
3	0	0	0	1	0	0
2	1	1	0	2	0	0
2	1	0	1	1	0	0
99	0	0	0	1	0	0
4	1	1	0	1	0	0
2	2	2	0	1	0	2
2	0	0	0	0	0	0
4	1	2	1	1	0	2
4	2	2	2	2	2	2
4	0	0	0	1	0	0
2	0	0	0	1	0	0
99	0	0	0	0	0	0
3	0	0	0	1	0	1
4	0	0	0	0	0	0
2	0	0	0	0	0	0
3	0	0	0	1	0	0
2	2	0	0	2	2	1
3	0	0	0	0	0	0
4	0	0	0	0	0	0
4	0	0	0	1	0	0
2	1	0	1	2	0	0
99	99	99	99	99	99	99
2	0	0	0	0	0	0
1	0	0	0	1	0	0
2	1	1	1	2	0	0
4	0	1	0	0	0	0
4	1	0	0	1	0	0
2	1	1	2	2	1	0
3	0	0	0	1	0	0
4	0	0	0	1	0	0
1	1	0	1	1	1	1
2	0	0	0	0	0	0
1	2	1	0	2	0	2
3	0	0	0	2	0	0
2	1	0	1	0	0	0
4	2	1	1	2	0	2
4	0	0	1	1	2	0
4	0	0	0	2	0	0
3	0	0	0	0	0	0
99	1	0	1	2	0	0
2	1	0	1	0	0	0
1	2	2	2	2	2	2
4	2	1	2	1	0	1
3	1	0	1	2	1	2
2	1	0	0	1	0	0

4	1	1	1	1	1	1
4	2	0	0	2	0	0
2	1	99	1	2	0	0
3	1	0	1	2	1	0
3	1	2	0	2	0	0
3	1	0	0	1	0	0
2	0	0	0	2	0	0
99	0	0	0	1	0	0
4	1	0	0	2	0	0
2	0	0	0	1	0	0
4	0	0	0	0	0	0
4	0	0	0	0	0	0
2	1	0	0	2	0	0
2	0	0	0	1	0	0
2	99	99	99	99	99	99
4	0	0	0	1	0	0
2	1	0	0	2	0	0
3	1	1	1	1	1	1
2	1	0	0	2	0	1
2	2	1	2	2	2	2
2	0	0	0	0	0	0
4	0	0	0	2	0	0
4	0	0	0	0	0	0
1	2	0	2	1	99	0
3	1	1	0	2	1	1
2	0	0	0	1	0	0
2	2	0	0	2	0	0
99	1	1	1	1	0	2
4	2	0	0	1	0	0
4	2	2	0	2	0	0
3	2	2	2	2	2	2
4	0	0	0	0	0	0
1	0	0	0	1	0	0
2	1	2	1	2	1	1
4	0	0	0	1	0	0
1	0	0	0	0	0	0
3	1	0	0	1	0	1
1	2	0	1	2	1	1
4	2	2	2	2	2	2
1	2	1	2	2	0	0
3	0	0	0	0	0	0
2	2	1	2	1	0	1
3	2	2	1	2	2	2
4	1	1	1	1	0	1
3	1	0	0	0	0	0
4	0	0	0	0	0	0
99	1	1	1	2	1	2
2	0	0	0	1	0	1

4	0	0	0	0	0	0
4	1	0	0	1	0	0
1	1	0	0	2	0	0
4	1	0	1	2	0	0
2	2	1	1	2	0	1
3	1	1	2	2	1	1
4	0	0	0	0	0	0
3	0	0	0	0	0	0
2	1	0	1	2	1	0
4	0	0	0	0	0	0
2	1	0	0	99	0	0
3	0	0	0	0	0	0
3	1	99	2	0	0	0
4	0	0	0	0	0	0
3	1	0	1	2	1	1
4	0	0	0	0	0	0
99	1	0	0	1	0	0
4	1	0	0	1	0	0
4	1	0	0	0	0	1
4	1	1	1	2	1	1
3	2	0	0	2	1	1
4	1	0	1	2	2	0
99	0	0	0	0	0	0
4	0	0	0	2	0	0
3	1	0	0	1	0	0
99	1	0	0	0	0	1
99	99	99	99	99	99	99
2	1	0	0	2	0	0
2	1	1	1	1	1	1
4	1	0	0	1	0	0
99	99	99	99	99	99	99
4	1	0	0	1	1	0
4	1	0	0	0	0	0
4	0	0	0	2	0	0
99	0	0	0	0	0	0
4	0	0	0	0	0	0
4	0	0	0	0	0	0
3	1	2	0	2	0	0
4	0	0	0	0	0	0
4	0	0	0	0	0	0
4	0	0	0	0	0	0
4	1	0	0	2	0	0
3	0	0	0	2	0	0
4	1	0	0	2	0	0
1	1	2	0	2	0	0
2	1	1	1	1	1	1
4	0	0	0	0	0	0
1	0	0	0	1	0	0

4	0	0	0	0	0	0
3	2	2	2	2	2	2
1	1	0	1	1	0	1
2	0	0	99	0	1	0
2	0	0	0	1	0	0
4	0	0	0	0	0	0
3	0	1	0	2	0	0
3	2	1	2	1	1	2
4	1	0	0	0	0	0
3	1	0	0	1	0	0
3	0	0	0	0	0	0
3	1	0	0	2	0	2
4	2	2	2	2	1	2
4	1	1	1	1	1	0
3	0	0	0	0	0	0
2	1	1	0	0	0	0
1	99	99	99	99	99	99
4	0	0	0	2	0	0
2	1	1	1	1	1	1
2	2	2	2	2	2	0
1	1	0	0	0	0	0
3	1	0	0	1	0	0
3	0	0	0	0	0	0
3	0	0	0	0	0	0
1	2	1	0	2	1	2
99	0	0	0	0	0	0
4	2	0	0	1	0	0
4	0	0	0	1	0	0
4	0	1	0	1	0	0
3	1	0	2	2	1	0
2	1	0	1	2	0	1
4	0	0	0	1	0	0
99	2	0	0	2	0	0
99	2	2	99	2	1	1
3	1	0	1	1	1	1
99	0	0	0	1	0	0
2	0	0	0	0	0	0
4	2	0	2	2	2	2
3	0	0	0	1	0	1
4	0	0	0	0	0	0
1	2	2	2	2	1	2
3	1	0	0	1	0	0
2	0	1	1	0	0	0
3	1	1	1	2	0	1
4	0	0	0	1	0	0
2	1	0	1	1	0	1
4	1	0	0	1	0	1
3	0	0	0	1	0	0

4	0	0	0	0	0	0
4	0	0	0	2	0	1
99	0	0	0	0	0	0
2	2	2	2	1	1	1
4	0	0	0	2	0	0
3	1	0	0	2	0	0
4	0	0	0	0	0	0
2	2	0	1	2	1	0
99	2	0	0	2	1	0
2	2	0	0	2	0	0
4	0	0	0	2	0	0
2	0	1	1	2	1	2
2	1	1	1	2	0	0
2	1	0	0	1	0	0
4	1	0	0	1	0	1
4	99	99	99	99	99	99
3	0	0	0	2	0	0
2	2	1	1	2	1	2
2	2	0	2	2	1	0
3	0	0	0	1	0	0
2	1	1	1	2	1	0
3	2	1	2	1	2	2
2	0	0	0	0	0	0
2	1	2	0	1	0	0
1	0	0	1	1	0	99
4	0	0	0	0	0	0
2	0	0	0	0	0	0
1	1	1	0	1	0	0
4	1	1	1	2	1	1
1	0	0	0	0	0	0
2	0	0	0	1	0	0
3	0	0	0	0	0	0
3	0	0	0	0	0	0
3	99	0	99	99	99	99
3	1	0	0	0	0	0
4	0	0	99	1	0	0
3	0	0	0	0	0	0
3	0	0	0	1	0	0
2	1	0	1	0	0	1
4	0	0	0	2	0	0
4	0	0	0	0	0	0
4	1	1	2	1	0	1
99	2	1	0	1	1	2
1	2	1	0	1	1	0
99	0	0	0	99	99	99
3	0	0	0	1	0	0
4	0	0	0	0	0	0
2	1	1	2	2	0	0

3	0	0	0	1	0	0
4	0	0	0	2	0	0
3	0	0	1	2	0	0
4	0	0	0	1	1	2
4	0	0	0	0	0	0
4	0	0	0	0	0	0
4	0	0	0	1	0	0
3	1	1	2	1	0	0
3	0	0	0	1	0	1
2	0	0	2	2	2	0
4	1	1	99	2	0	1
3	1	99	1	2	0	0
3	0	0	0	1	0	0
3	1	2	99	2	1	2
99	1	1	0	1	0	0
3	0	0	0	2	0	0
4	1	1	1	0	0	0
4	0	0	1	0	1	99
4	1	0	0	1	0	0
99	0	0	0	0	0	0
4	2	2	2	2	1	2
4	0	0	0	0	0	0
1	1	0	0	2	0	0
1	0	0	0	1	0	0
3	1	0	0	2	0	0
3	2	1	1	2	1	1
2	1	0	1	2	1	1
4	0	0	0	0	0	0
3	0	0	0	1	0	0
2	2	1	0	2	1	1
4	0	0	0	1	0	0
2	2	0	0	99	1	0
4	0	0	0	0	0	0
3	2	2	2	2	2	2
3	0	0	0	0	0	0
4	0	0	0	1	0	0
4	0	0	0	0	0	0
99	0	0	0	1	0	0
2	0	0	0	0	0	0
4	0	0	0	0	0	0
2	0	0	0	1	0	0
4	2	2	2	2	2	2
2	0	0	0	1	0	0
4	1	0	0	2	0	0
2	1	0	0	1	1	0
4	0	0	0	1	0	0
2	1	0	1	1	1	0
4	1	0	0	1	0	1

2	1	0	0	1	0	2
99	0	0	0	0	0	0
2	1	0	0	1	99	99
3	1	0	0	0	0	0
2	2	1	2	2	1	2
4	0	0	0	1	0	1
3	1	1	0	2	0	0
4	2	1	0	2	0	0
3	0	0	0	1	0	0
4	0	0	1	1	0	2
2	1	1	0	1	0	1
4	0	0	2	0	2	2
99	99	99	99	99	99	99
2	0	0	0	1	0	0
3	0	0	0	0	0	0
1	1	1	1	1	1	0
1	2	2	2	2	1	2
2	1	0	2	2	0	0
3	1	0	2	1	0	0
2	2	1	1	2	1	2
3	0	0	0	0	0	0
2	0	0	0	1	0	0
4	0	0	0	1	0	0
2	2	2	2	2	1	2
3	0	0	0	1	0	0
2	1	0	1	1	0	0
4	1	0	0	2	2	2
4	1	1	0	2	0	1
2	0	0	0	0	0	0
4	0	0	0	0	0	0
1	1	2	2	1	2	2
4	0	0	0	1	0	0
4	1	0	0	2	0	0
1	1	0	0	2	1	0
2	1	1	1	1	0	0
3	0	0	0	0	0	0
1	1	1	1	2	1	0
2	0	0	0	1	0	0
4	1	2	0	1	0	2
1	0	0	0	1	0	0
2	1	1	1	2	1	0
1	0	0	0	0	0	0
3	1	0	0	2	0	1
99	0	0	0	1	0	0
3	0	0	0	1	0	0
4	0	0	0	1	0	0
3	0	0	0	1	0	0
4	0	0	0	1	0	0

4	1	0	0	1	0	1
3	1	1	1	0	0	0
3	1	0	0	1	0	0
2	1	0	0	1	0	0
4	1	1	0	1	0	0
2	0	0	0	0	0	0
3	0	0	0	0	0	0
1	1	0	0	1	0	0
4	0	0	0	1	0	0
4	0	0	0	0	0	0
3	0	0	0	1	0	0
4	1	1	0	2	0	0
2	0	0	0	1	0	0
4	0	0	0	2	0	0
4	0	0	0	0	0	0
2	2	1	1	2	1	1
2	0	1	0	1	0	0
4	0	1	0	2	0	2
1	0	0	0	2	1	0
4	0	0	0	1	0	0
2	1	0	0	2	0	0
1	2	0	0	0	0	0
1	0	0	0	1	0	0
3	1	0	1	1	1	0
3	0	0	0	1	0	0
3	0	0	1	1	0	0
4	0	0	0	0	0	0
3	0	1	99	0	0	0
3	1	0	0	0	0	1
3	1	0	0	0	0	0
2	1	1	0	2	0	0
3	0	0	1	2	0	1
99	0	0	0	99	0	0
2	2	1	1	2	0	1
2	1	1	1	2	0	1
2	1	1	1	2	0	0
99	0	0	0	1	0	0
3	1	1	1	1	1	1
99	1	0	1	1	0	0
2	1	0	0	0	0	2
99	0	0	0	2	0	0
99	1	1	1	1	1	1
3	1	0	0	1	0	0
3	1	0	0	2	0	1
2	0	0	0	1	0	0
4	1	0	0	0	0	0
1	1	2	1	2	1	0
4	2	2	2	2	2	2

4	1	1	0	0	1	0
2	0	0	0	2	0	0
3	0	0	0	1	0	1
4	0	0	0	2	0	0
1	0	0	0	1	0	0
3	2	2	2	2	2	2
3	0	0	0	1	0	0
2	1	0	0	0	0	0
4	0	0	0	1	0	0
99	0	0	0	1	0	0
2	1	0	0	2	0	0
3	1	1	1	1	1	1
2	2	0	1	1	1	0
3	1	1	1	1	1	0
2	2	2	1	2	1	1
1	1	1	1	1	1	1
3	0	0	0	1	0	0
2	0	0	0	2	0	0
3	1	0	1	1	1	1
4	1	1	1	99	0	2
2	1	1	1	1	1	1
4	0	0	0	0	0	0
3	0	0	0	1	0	0
2	0	0	0	0	0	0
4	2	2	0	99	0	0
1	1	1	1	1	1	1
1	1	0	0	0	0	0
2	2	2	2	1	2	2
2	1	0	1	2	1	1
4	2	1	1	1	1	1
1	2	0	1	1	1	1
2	0	0	0	1	0	0
3	1	1	0	1	1	0
3	1	0	1	1	0	0
4	1	0	2	2	0	1
2	1	1	1	1	1	1
2	0	0	0	0	0	0
2	1	0	1	2	0	2
1	2	1	1	2	0	1
3	1	0	0	1	0	0
3	2	2	2	2	2	2
3	2	1	2	2	2	2
4	0	1	0	1	99	99
2	2	2	2	1	1	1
4	1	0	1	1	1	1
2	1	0	0	2	1	0
2	1	0	0	1	0	1
1	2	1	1	2	0	1

4	1	1	1	1	0	0
4	1	0	0	1	0	0
3	0	0	0	1	0	0
4	0	0	0	0	0	0
99	99	99	99	99	99	99
2	2	1	1	2	1	1
2	0	0	0	2	0	1
4	0	0	0	2	0	0
4	0	0	0	1	0	1
4	0	0	0	0	0	0
2	1	0	1	1	1	1
4	0	0	0	1	0	0
2	0	0	0	1	0	0
3	0	0	0	1	0	0
3	0	0	0	1	0	0
4	2	2	2	2	2	2
4	0	0	0	2	1	1
3	1	0	0	2	1	0
4	2	2	1	2	1	2
2	1	0	0	1	0	0
2	0	1	0	0	0	0
2	0	0	0	2	0	0
3	1	0	0	1	0	1
4	0	0	0	1	0	1
3	0	0	0	2	1	0
3	0	0	0	1	1	1
2	1	0	1	1	1	0
4	0	0	0	1	0	0
4	0	0	0	2	0	0
4	0	0	0	0	0	0
3	1	0	0	2	1	0
2	0	0	0	1	0	0
4	0	0	0	1	0	0
2	0	0	0	0	0	0
3	0	0	0	0	0	0
1	0	0	0	0	0	0
3	0	0	0	0	0	0
1	2	1	2	2	2	0
4	0	0	0	1	0	0
4	0	0	0	1	0	0
2	0	0	0	1	0	0
4	0	0	0	0	0	0
4	1	1	1	1	1	1
3	1	0	2	2	1	2
4	1	0	0	1	0	0
4	0	0	0	2	0	1
2	0	1	1	1	0	1
2	0	0	0	0	0	0

4	1	1	1	2	1	2
99	0	0	0	0	0	1
1	0	0	0	0	0	0
3	1	0	0	1	0	1
3	0	0	0	1	0	1
4	1	0	0	1	0	0
3	1	1	1	1	0	0
3	99	0	0	2	0	0
2	0	0	0	0	0	0
3	0	0	0	1	0	0
3	0	0	0	1	0	0
4	1	0	1	1	0	1
4	2	1	0	2	0	0
2	1	1	1	2	0	0
4	0	0	0	1	0	0
1	0	0	0	2	0	0
1	1	0	1	1	0	0
2	0	0	0	1	0	0
1	1	0	0	1	0	1
3	1	0	0	1	1	0
2	1	0	0	0	0	0
2	2	1	2	0	2	2
4	1	0	99	1	0	0
4	2	2	2	2	2	2
2	0	0	0	0	0	0
3	0	0	0	0	0	0
99	0	0	0	1	0	0
3	1	0	0	1	99	0
4	1	1	1	2	0	1
2	1	0	0	1	0	0
3	0	0	0	0	0	0
2	0	0	0	2	0	1
2	0	0	0	2	0	0
2	1	1	0	0	1	0
4	0	0	0	1	0	0
3	1	0	1	1	1	0
3	0	0	0	0	0	0
2	0	0	0	1	0	1
4	0	0	0	1	0	0
2	1	1	1	1	0	0
4	1	0	0	1	0	0
3	0	0	0	1	0	0
4	0	1	0	1	0	0
4	2	2	0	1	2	0
2	0	0	2	2	1	2
3	0	0	0	0	0	0
3	0	0	0	2	0	0
3	0	0	0	1	0	0

4	1	0	1	2	0	0
3	1	0	1	2	0	0
3	1	0	0	1	1	2
3	0	1	0	1	0	1
99	99	99	99	99	99	99
3	1	1	1	1	0	1
4	2	2	2	2	2	2
2	0	0	0	0	0	0
2	0	0	0	0	0	0
2	1	1	0	2	0	0
3	1	1	1	2	1	1
2	1	0	0	2	0	0
3	1	1	2	2	0	0
4	2	1	1	2	1	0
2	0	0	0	0	0	0
3	2	2	2	2	1	2
3	0	0	0	0	0	0
3	0	0	0	2	0	0
1	0	0	0	1	0	0
1	0	0	1	1	0	1
1	0	0	0	0	0	0
3	2	0	0	2	1	0
4	1	1	1	1	0	1
2	2	2	2	2	1	1
4	2	1	1	2	0	0
1	2	2	2	2	2	2
2	1	1	0	2	0	0
2	0	0	0	0	0	0
1	1	0	0	2	0	0
2	0	0	1	1	0	0
4	1	0	1	2	0	1
1	1	0	1	1	0	1
3	2	1	2	2	2	1
4	0	0	0	0	0	0
2	0	0	0	0	0	0
2	0	0	0	1	0	0
4	1	1	1	2	1	1
4	2	1	2	1	1	2
2	0	0	0	1	0	0
3	1	0	0	1	0	1
3	1	1	2	2	1	1
1	0	0	0	2	1	0
3	0	0	2	2	0	1
3	1	1	1	1	1	1
4	2	0	2	2	2	0
2	2	0	0	2	1	1
1	2	0	1	2	0	0
4	0	0	0	0	0	0

2	0	0	1	2	0	1
4	0	0	0	1	0	0
3	1	0	0	2	0	0
4	0	0	0	1	0	0
4	0	0	0	0	0	0
4	1	0	1	2	0	0
3	0	0	0	0	0	0
3	0	0	0	1	0	0
4	1	1	0	1	0	0
2	1	2	2	1	1	2
3	0	0	0	2	0	0
3	0	0	0	1	0	0
3	1	1	1	1	1	1
2	1	0	0	2	0	0
4	1	0	0	0	0	0
4	0	0	0	0	0	0
4	1	0	0	1	0	0
4	0	0	0	2	0	0
2	0	0	0	2	0	0
99	0	0	0	1	0	0
4	1	1	1	2	2	1
2	0	0	0	0	0	0
1	1	1	0	1	1	1
3	0	0	0	1	0	0
2	0	0	0	0	0	0
4	1	0	2	2	0	0
3	1	1	2	2	1	0
2	1	1	1	1	1	1
99	0	0	1	1	0	0
4	1	1	0	0	0	0
3	0	0	0	1	0	0
3	0	1	1	2	1	0
3	1	1	1	2	1	1
1	1	1	1	1	1	1
4	1	0	0	2	0	0
3	1	1	0	2	0	1
2	1	0	1	2	1	0
2	2	2	99	2	1	2
3	2	0	0	2	0	0
4	1	1	1	1	0	1
1	2	0	0	2	0	1
4	2	0	0	2	0	2
99	99	99	99	99	99	99
1	0	1	1	0	0	0
4	0	0	0	2	0	1
4	2	0	0	2	0	2
2	1	1	1	2	1	1
2	1	1	0	1	1	0

2	0	0	0	1	0	0
3	1	0	1	2	0	0
3	0	0	0	1	0	0
1	0	1	1	2	0	0
1	0	0	0	1	0	0
2	0	0	1	2	2	0
2	0	0	0	1	0	0
3	2	2	2	2	2	2
2	2	0	0	2	0	2
2	0	0	0	2	0	0
2	0	0	0	0	0	0
1	1	0	0	2	0	0
2	0	0	0	0	0	0
2	0	0	0	2	0	0
2	0	0	0	2	1	1
2	1	0	0	2	1	1
2	1	0	1	2	0	0
2	0	0	0	1	0	0
2	1	0	0	0	0	0
1	0	2	0	1	0	2
4	1	0	0	1	0	1
3	1	0	99	2	99	99
2	1	0	0	0	0	0
2	0	0	2	2	1	0
4	1	1	0	1	1	0
2	0	0	0	0	0	0
2	0	0	0	1	0	99
4	99	99	99	99	99	99
3	2	2	2	0	2	2
4	1	0	0	2	1	0
3	99	99	99	99	99	99
1	2	1	2	2	2	2
2	1	0	0	1	0	0
3	2	2	1	1	1	0
4	2	0	0	2	2	1
3	2	2	2	2	1	1
3	1	0	0	1	0	0
2	2	1	2	2	1	1
4	1	1	1	2	2	1
1	0	0	0	2	0	0
4	1	1	1	1	1	1
3	2	2	2	2	2	2
1	1	1	1	2	0	0
4	1	0	0	2	0	1
1	1	1	0	2	1	0
2	1	1	1	2	1	1
3	0	0	1	2	2	2
2	1	0	0	2	0	0

3	1	1	1	1	0	0
1	1	0	0	2	0	0
4	0	0	0	0	0	0
3	0	0	0	1	0	0
2	2	99	2	2	0	2
1	2	1	0	2	0	0
3	0	0	0	0	0	0
3	1	0	1	1	1	1
3	1	0	0	2	99	0
2	0	0	0	0	0	0
2	1	0	0	1	0	0
2	0	0	1	2	0	0
4	1	0	0	2	1	0
2	1	1	1	2	1	1
2	1	1	0	2	0	1
2	1	1	0	2	0	0
1	0	2	2	2	0	2
4	1	0	0	1	0	0
3	0	1	0	1	0	0
4	0	1	0	2	0	0
4	2	0	1	2	2	2
4	0	0	0	0	0	0
3	2	1	1	1	0	1
4	0	0	0	1	0	0
4	1	1	0	0	0	0
3	2	2	2	2	2	2
2	0	0	0	2	0	0
4	2	2	2	2	2	2
3	0	0	0	2	0	0
4	0	0	0	1	0	0
3	2	0	0	2	0	0
3	0	0	0	1	0	0
3	1	1	0	1	0	1
2	0	0	0	1	0	0
3	1	1	1	2	1	1
4	2	2	2	2	0	0
4	1	0	0	1	1	0
3	0	0	0	1	0	0
4	0	0	0	1	0	0
3	1	1	0	1	0	1
1	2	2	2	2	2	2
4	0	0	0	2	0	0
4	1	0	0	1	0	0
4	2	0	0	0	1	0
4	0	0	0	1	0	0
3	0	0	99	1	0	0
2	2	0	0	2	1	0
4	2	1	2	2	2	2

1	2	0	0	2	0	2
2	1	0	0	0	0	0
2	0	0	0	0	0	0
2	2	0	1	2	0	2
4	0	0	0	99	0	0
99	0	0	0	0	2	2
2	0	0	0	0	0	0
1	1	0	1	0	0	1
1	2	2	2	0	0	0
2	1	0	0	1	0	0
1	2	2	2	2	1	2
4	0	0	0	0	0	0
4	0	0	0	1	0	0
99	1	0	0	1	0	0
2	1	0	1	2	1	2
3	2	1	2	1	1	2
2	0	0	1	2	0	1
2	0	0	0	1	0	0
2	0	0	0	1	0	0
2	1	1	1	2	1	2
3	0	0	0	2	0	0
3	1	1	0	2	0	1
3	0	0	0	1	0	0
2	1	0	1	2	1	0
3	0	0	0	0	0	0
2	0	0	0	0	0	0
2	0	0	0	0	0	0
3	0	1	0	0	0	1
4	0	0	0	2	0	1
4	0	0	0	1	0	0
4	1	1	2	2	2	1
1	0	0	2	2	0	0
1	1	0	0	1	0	1
3	1	1	0	0	0	0
3	1	0	0	2	0	1
3	1	0	0	2	0	0
4	0	0	0	1	0	0
2	1	0	0	2	0	1
99	99	99	99	99	99	99
4	1	99	99	2	1	1
1	1	0	0	1	0	0
1	2	2	2	2	2	1
2	2	2	2	2	2	2
4	2	2	2	2	2	2
4	0	0	0	0	0	0
3	0	0	0	0	0	0
3	1	0	0	1	0	0
1	0	0	0	2	0	0

3	2	1	2	2	2	1
3	1	1	1	1	1	1
4	1	0	1	0	1	1
3	0	0	0	2	0	0
2	1	1	1	1	1	1
3	0	0	0	0	0	0
4	0	0	0	0	0	0
4	0	0	1	0	0	1
4	0	1	2	2	0	0
3	1	1	1	1	1	1
2	0	0	0	2	0	0
4	2	0	1	1	0	1
4	1	0	1	2	0	0
3	1	1	1	1	1	1
3	2	1	2	2	1	1
1	1	0	2	2	1	1
4	2	2	2	2	2	2
2	1	0	2	2	1	0
1	1	0	0	1	0	0
2	1	0	0	2	0	0
4	1	0	2	99	0	0
3	2	0	0	2	0	1
4	1	1	1	1	0	0
1	2	1	2	2	1	0
1	2	1	2	2	1	1
4	2	0	2	2	0	1
2	1	0	2	2	1	2
1	0	0	0	2	0	0
1	0	0	0	0	0	0
2	0	0	0	1	0	0
4	1	0	0	0	0	0
99	0	0	0	0	0	0
99	1	1	1	1	1	1
1	0	0	0	0	0	0
3	0	0	0	1	0	0
4	2	1	0	0	0	2
4	0	0	0	0	0	0
99	0	0	0	0	0	0
3	0	0	0	1	0	0
2	0	0	0	0	0	0
4	2	0	1	1	0	1
4	0	0	0	1	0	1
4	0	0	0	0	0	0
1	0	0	0	0	0	0
2	1	1	0	0	0	0
4	0	0	0	0	0	0
4	1	0	0	0	0	0
4	1	0	1	1	2	1

3	0	0	0	1	0	0
1	0	0	0	1	0	0
1	0	0	0	2	0	0
4	0	0	0	2	0	0
2	0	0	0	0	0	0
3	0	0	0	1	0	0
4	1	0	0	2	2	2
3	1	0	2	1	0	1
3	0	0	0	0	0	0
4	0	1	0	2	0	1
4	1	0	0	2	0	0
4	1	0	0	1	0	0
2	0	0	0	0	0	0
4	0	0	0	0	0	0
2	0	1	0	2	0	0
3	1	0	0	1	1	1
4	1	1	0	2	0	0
4	2	1	1	1	0	2
3	1	1	1	2	1	1
1	0	0	0	2	0	0
2	0	0	0	0	0	0
3	1	0	1	2	1	1
4	1	0	0	1	0	0
3	0	0	0	1	0	0
2	0	0	0	0	0	0
4	2	1	2	1	0	2
4	0	0	0	0	0	0
4	0	0	0	0	0	0
3	0	1	1	1	0	1
99	0	0	0	1	0	0
4	0	0	0	0	0	0
2	1	1	1	1	1	1
2	1	0	1	1	0	1
2	1	0	1	2	0	1
4	0	0	0	1	0	2
4	0	0	0	0	0	0
1	0	0	0	0	0	0
1	2	1	2	1	1	2
4	0	0	0	0	0	0
4	0	0	0	0	0	0
4	0	0	0	2	0	0
3	1	0	1	1	0	2
2	2	1	2	2	1	2
4	1	1	1	2	0	1
2	1	0	0	2	0	0
3	1	0	0	2	1	0
3	0	0	0	0	0	1
2	1	1	1	1	1	2

4	0	0	0	1	0	0
4	0	0	0	0	0	0
3	0	0	0	1	0	0
4	1	0	0	0	0	0
3	1	0	0	1	0	1
2	1	0	1	2	0	0
4	1	0	0	2	1	0
3	0	0	0	0	0	0
2	1	1	1	1	1	1
3	0	0	0	0	0	0
4	1	0	1	1	1	2
3	2	1	2	2	1	1
3	0	0	0	0	0	0
2	1	1	0	1	0	1
2	0	0	0	1	0	0
3	1	0	1	0	0	0
4	1	1	1	1	1	1
3	1	0	0	2	0	0
2	1	0	0	1	0	0
2	0	0	0	1	0	0
2	0	0	0	1	0	0
4	0	0	0	0	0	0
4	0	0	0	0	0	0
2	2	1	1	1	1	1
1	0	0	0	0	0	0
3	0	0	0	1	0	0
3	0	0	1	0	0	0
4	0	0	0	1	0	0
3	0	0	0	1	0	0
3	1	1	1	1	0	0
4	2	0	0	2	0	1
3	0	0	0	0	0	0
3	1	0	0	1	1	1
2	1	0	1	1	1	1
2	2	2	2	2	2	2
2	99	2	1	2	1	0
4	1	0	1	1	0	1
3	1	0	0	1	0	0
4	1	0	0	1	0	0
2	1	0	1	1	0	1
3	1	0	1	2	0	1
3	0	0	0	1	0	0
2	2	0	2	2	0	2
3	0	0	0	0	0	0
1	0	0	0	0	0	0
4	1	1	1	1	1	1
2	1	1	0	0	1	0
3	0	0	0	1	0	0

2	0	0	0	0	0	0
1	0	0	0	0	0	0
3	0	0	0	0	0	0
2	0	0	0	1	0	0
4	0	0	0	1	0	0
3	0	0	0	1	0	0
99	0	0	0	0	0	0
99	1	0	0	1	1	0
4	1	0	0	1	0	1
99	0	0	0	1	0	0
4	0	0	0	0	0	0
4	0	0	0	0	0	0
2	0	0	0	0	0	0
4	1	1	2	2	1	2
4	1	0	1	1	0	1
4	0	0	0	0	0	0
3	1	1	1	1	1	1
3	0	0	0	1	1	0
1	2	1	1	2	1	1
4	0	0	0	0	0	0
4	0	0	0	1	0	0
99	0	0	0	0	0	0
3	0	0	0	1	0	0
3	0	1	0	1	1	1
4	1	0	0	2	0	0
3	0	0	0	1	0	0
3	1	0	0	1	0	0
3	0	0	0	0	0	0
4	0	0	0	0	0	0
3	1	0	0	1	1	0
2	0	0	0	1	0	0
2	0	0	0	0	0	0
4	0	0	0	1	0	0
3	1	0	1	0	0	0
4	0	0	0	0	0	0
4	0	0	0	1	0	0
4	0	0	0	0	0	0
3	1	0	2	2	0	0
3	1	0	1	2	0	1
1	0	0	0	2	0	0
1	0	0	0	1	2	0
2	2	1	1	2	0	1
3	1	0	0	2	0	0
3	1	1	2	2	1	1
4	1	0	0	1	0	0
4	0	0	0	1	0	0
3	0	0	0	0	0	0
4	1	0	1	2	1	0

2	1	1	1	1	1	0
4	1	1	1	1	1	1
3	1	1	0	2	0	1
3	1	0	0	1	1	1
99	0	0	0	0	0	0
4	1	1	1	1	0	0
3	1	0	1	2	0	0
4	0	0	0	0	0	0
2	0	0	0	1	0	0
2	1	1	1	1	1	1
3	2	0	0	2	0	0
4	0	0	0	0	0	0
2	1	0	0	2	0	0
4	1	0	0	1	0	0
3	0	0	0	0	0	0
2	2	2	2	2	2	2
4	1	1	1	1	0	0
3	2	1	1	1	0	0
2	1	0	0	2	0	1
3	1	0	1	1	0	1
4	1	0	0	2	1	2
3	1	1	1	1	0	1
4	1	0	0	0	1	0
4	1	1	1	0	0	0
4	1	0	0	2	0	0
99	99	99	99	99	99	99
1	2	2	2	2	2	1
4	0	0	0	1	0	0
3	1	0	2	2	1	1
2	0	0	1	1	0	0
2	2	1	2	2	0	1
1	0	0	1	1	0	0
3	0	0	0	0	0	0
2	1	0	0	2	1	0
2	2	2	2	2	2	2
2	0	1	1	0	0	0
2	1	0	0	1	1	1
2	2	0	2	2	0	2
2	0	0	0	0	0	0
4	2	0	2	2	0	1
3	1	0	0	2	0	0
2	1	1	2	1	1	1
2	1	0	1	1	0	0
4	2	2	2	2	2	2
4	0	0	0	1	0	0
2	1	1	1	1	1	1
2	2	1	1	0	0	0
99	1	0	1	1	0	0

3	0	0	0	0	0	0
2	1	1	1	1	1	0
1	1	0	0	2	0	0
3	1	0	0	1	1	1
2	1	0	0	2	1	2
1	0	0	0	2	0	0
2	2	1	2	2	1	2
3	0	0	0	0	0	0
2	1	1	1	2	1	1
2	1	1	0	1	0	1
3	0	0	0	1	0	0
2	1	0	1	2	0	0
2	0	0	0	0	0	0
4	1	1	0	2	1	1
4	2	1	1	2	1	1
3	1	0	1	1	0	0
2	0	0	0	0	0	0
4	0	0	0	1	0	0
4	1	0	2	0	0	0
3	1	1	1	2	1	0
1	0	0	0	1	0	0
3	0	0	0	1	0	0
3	1	0	0	1	0	0
3	2	0	1	2	0	2
3	0	0	0	1	0	0
2	1	0	1	2	1	1
4	2	1	1	0	0	1
2	1	0	0	1	0	0
2	0	0	0	0	0	0
2	0	0	0	0	0	0
3	1	0	0	2	0	0
4	1	0	0	0	0	0
3	1	0	0	1	0	0
4	0	0	0	1	0	0
2	2	1	2	2	1	2
4	1	0	0	2	1	1
4	0	0	0	2	0	0
4	0	0	0	0	0	0
4	1	1	1	1	1	2
3	1	0	0	2	0	0
3	1	1	1	1	1	1
4	1	0	0	0	0	0
4	1	0	1	2	0	0
2	1	0	0	2	0	0
3	0	0	0	0	0	0
99	0	0	0	0	0	0
4	0	0	0	1	0	0
3	0	0	2	2	0	0

4	1	0	0	1	0	0
4	0	0	0	1	1	0
4	0	0	0	1	2	2
99	99	99	99	99	99	99
3	2	2	2	2	1	1
4	0	0	0	0	0	0
2	0	0	0	1	0	0
3	1	0	0	1	0	1
3	0	0	0	1	0	0
3	0	0	0	1	0	0
99	0	0	0	0	0	0
2	1	0	1	2	0	0
99	2	1	1	2	0	2
4	0	0	0	1	0	0
2	0	0	0	2	0	1
4	1	1	2	1	0	0
3	1	0	0	2	1	1
2	0	0	0	2	0	0
3	2	0	1	2	1	2
2	1	0	1	2	0	1
2	2	1	1	1	1	0
3	0	0	0	1	0	0
99	1	0	0	1	0	0
2	2	2	0	2	0	0
2	1	1	1	1	0	0
4	2	1	0	2	0	1
3	2	0	2	2	1	1
4	1	0	1	0	0	0
3	2	1	2	0	1	0
4	2	2	99	2	0	0
3	0	0	0	1	0	0
2	2	1	1	2	0	1
1	1	0	0	1	0	0
3	0	0	0	0	0	0
4	2	2	0	2	2	2
2	1	0	1	2	1	0
4	1	1	0	0	0	0
3	0	0	2	0	0	0
1	1	0	0	1	0	0
2	0	0	0	2	0	0
4	0	0	0	0	0	0
4	0	0	0	1	0	0
4	0	0	0	2	0	0
1	0	0	0	0	0	0
3	1	0	0	1	0	0
3	2	0	1	2	0	1
3	1	1	2	2	1	1
2	1	0	0	2	0	1

99	0	0	0	0	0	0
4	0	0	0	2	0	0
3	1	0	2	1	1	1
2	1	1	1	1	0	1
3	1	0	0	1	0	0
3	0	0	0	0	0	0
4	2	1	1	1	0	0
4	1	1	0	1	0	0
2	0	0	0	1	0	0
1	1	0	0	1	0	0
99	1	1	1	2	0	0
99	2	2	2	2	2	2
2	1	0	0	2	0	2
2	1	1	2	2	1	2
4	0	0	2	99	0	2
4	1	1	1	1	0	0
99	99	99	99	99	99	99
4	0	0	0	0	0	0
3	1	0	0	1	0	0
2	2	2	1	1	0	0
3	2	1	2	2	1	1
4	0	0	0	1	0	0
4	2	1	1	2	2	1
3	1	0	1	1	0	1
2	2	1	2	1	0	0
2	0	0	0	0	0	0
99	1	99	99	1	99	99
2	0	0	0	1	0	0
3	1	0	1	1	1	0
2	0	0	0	0	0	0
3	1	0	1	1	1	0
4	0	99	99	1	0	0
3	1	0	0	1	0	0
4	1	1	1	2	0	0
4	0	0	0	1	0	0
3	1	1	0	2	2	1
3	0	0	0	1	0	1
4	1	1	0	1	1	0
4	0	1	0	0	0	0
3	0	0	0	0	0	0
3	1	0	1	1	1	1
4	0	0	0	0	0	0
99	0	0	0	99	0	0
3	0	0	0	0	0	0
2	0	0	0	0	0	0
4	0	0	0	1	99	1
4	0	1	0	0	0	1
4	0	0	0	1	0	1

4	0	0	0	2	0	0
3	1	0	1	2	0	1
3	0	0	0	0	0	0
4	0	0	0	1	0	0
2	1	0	1	2	0	1
3	0	0	0	1	0	0
3	0	0	0	0	0	1
1	0	0	0	1	0	0
4	0	0	0	0	0	0
1	1	0	0	2	0	0
2	1	1	1	2	2	1
99	1	1	1	2	2	1
1	0	0	0	2	0	0
3	0	0	0	1	0	0
1	1	0	1	2	0	0
3	2	2	0	2	0	0
2	0	1	0	0	0	0
1	1	0	1	1	1	0
3	0	0	0	0	0	0
4	0	0	0	0	0	0
4	0	0	0	0	0	0
4	0	0	0	0	0	0
4	0	0	0	0	0	0
99	99	99	99	99	99	99
3	99	0	0	0	0	0
3	1	0	1	0	0	0
4	1	1	2	2	0	1
2	1	1	1	2	1	0
1	0	0	0	0	0	0
3	1	1	0	1	0	1
3	2	1	99	1	1	99
99	99	99	99	99	99	99
99	0	0	2	0	0	0
3	2	0	1	1	1	1
3	0	0	0	2	0	0
2	2	1	2	2	2	2
3	0	0	0	2	0	0
4	1	0	0	2	0	0
2	0	0	0	0	0	0
3	2	1	1	2	2	1
3	0	1	0	1	0	0
1	0	0	0	1	0	0
3	0	0	0	0	0	0
2	1	1	1	2	0	2
99	1	1	0	0	0	0
4	0	0	0	0	0	0
1	1	0	0	1	1	1
3	2	2	2	2	2	1

4	1	0	0	1	0	0
4	1	1	1	1	1	1
4	1	0	1	1	1	1
3	1	1	1	1	1	0
3	2	1	2	2	2	1
2	1	0	0	2	0	0
4	0	0	0	0	0	0
4	0	0	0	0	0	0
2	0	0	1	2	99	0
2	0	0	0	1	0	0
2	1	1	0	1	0	0
2	0	0	0	0	0	0
1	1	0	0	0	0	0
99	1	1	1	2	0	0
2	1	1	1	2	1	2
4	1	0	0	1	1	1
1	1	1	1	1	1	1
3	2	2	0	2	1	0
1	2	1	2	2	0	0
3	2	1	2	1	1	0
3	0	0	0	0	0	0
4	1	0	1	2	0	0
4	0	0	0	2	0	0
4	1	1	0	2	1	1
1	1	0	0	1	0	1
2	0	0	0	1	0	0
3	1	0	0	1	0	0
3	0	0	0	1	0	0
3	1	0	99	1	0	1
2	1	0	0	2	0	0
1	2	0	1	2	1	1
4	1	1	0	0	0	0
3	0	0	0	1	0	0
1	2	0	1	2	0	1
4	0	0	1	1	0	0
2	0	0	0	0	0	0
3	0	0	0	0	0	0
4	1	1	0	1	0	1
3	0	0	0	0	0	0
2	0	0	1	0	0	0
2	1	1	2	2	0	1
3	1	0	0	0	0	0
3	1	0	2	2	0	0
3	0	0	0	0	0	0
3	0	0	0	0	0	0
4	1	0	0	1	0	1
3	0	0	0	0	0	0
3	0	0	0	0	0	0

3	1	0	99	99	1	1
4	1	0	0	2	0	0
1	2	1	0	2	1	1
3	2	2	0	2	2	2
3	1	0	1	1	0	0
2	0	0	0	1	0	0
4	0	0	0	0	99	0
1	2	1	1	2	1	0
4	0	0	0	0	0	0
3	0	0	0	1	0	0
3	0	0	0	99	0	1
4	1	0	0	1	0	0
4	2	2	2	2	2	2
4	0	0	0	0	0	0
99	99	99	0	99	99	99
2	0	0	0	1	0	0
4	0	0	2	2	0	1
4	0	0	0	1	0	0
4	0	99	0	0	0	0
99	99	99	99	99	99	99
2	1	0	0	0	0	1
4	1	0	0	2	0	0
2	0	0	0	1	0	0
3	2	2	2	2	2	2
1	0	0	0	1	0	0
3	0	0	0	0	0	0
2	2	0	1	2	1	0
2	1	0	0	2	0	0
3	1	0	0	1	0	0
3	1	0	0	1	0	0
2	2	1	1	2	1	1
1	0	0	0	2	0	0
4	2	0	2	2	0	2
2	1	1	0	2	1	1
1	2	2	2	2	2	2
1	0	0	1	2	0	0
2	1	1	1	1	1	0
2	1	0	0	2	1	1
3	2	0	1	2	0	2
3	2	0	0	1	0	0
4	0	0	0	2	0	0
4	0	99	0	1	0	99
2	0	0	0	1	1	0
2	1	0	2	1	1	2
2	2	0	0	0	1	2
2	1	0	0	1	0	0
99	0	0	0	1	0	0
4	2	2	1	2	2	1

4	1	1	1	1	1	1
3	1	0	0	1	0	0
3	0	0	0	0	0	0
2	1	0	0	1	0	0
4	1	0	0	1	0	0
3	2	0	0	1	2	0
4	2	0	2	2	1	99
4	0	0	0	0	0	0
1	0	0	0	0	0	0
99	2	2	0	2	2	0
1	1	0	2	1	2	99
4	2	2	2	2	2	2
4	1	0	0	2	0	0
1	2	1	0	1	0	0
2	2	2	2	2	2	2
3	1	1	1	1	1	0
4	2	1	0	1	0	1
4	0	0	0	2	0	0
4	1	1	0	0	0	0
2	0	0	0	0	0	0
4	0	0	0	0	0	0
2	1	0	1	1	0	0
4	0	0	0	1	0	0
2	1	0	1	1	2	0
4	0	0	1	1	0	0
2	0	0	0	0	0	0
3	1	0	0	1	1	2
3	0	0	0	1	0	0
4	0	0	0	1	0	0
3	2	1	1	2	0	0
4	0	0	0	0	0	0
3	1	1	1	1	1	1
3	0	0	2	0	0	0
3	1	1	1	0	0	1
4	0	0	0	2	0	0
1	2	1	2	2	0	0
3	1	0	0	0	0	0
4	1	1	0	2	0	1
2	1	1	1	1	0	1
4	1	1	1	2	1	1
1	1	2	2	2	0	0
1	2	2	2	2	2	2
4	1	1	1	1	1	1
2	1	1	1	2	0	0
2	1	1	1	2	0	0
3	1	0	0	2	0	0
2	1	0	1	1	1	0
2	0	0	0	1	0	0

3	2	1	2	0	0	0
2	2	1	2	2	0	0
4	1	1	0	2	1	2
4	1	1	1	1	1	1
4	1	1	1	2	1	2
2	1	0	0	1	0	0
4	0	0	0	1	0	0
4	0	1	0	1	0	0
3	1	2	1	1	0	1
4	0	0	0	0	0	0
2	0	0	0	1	0	0
99	0	0	0	0	0	0
2	0	0	0	1	0	0
99	0	0	0	0	0	0
2	0	0	0	1	0	0
99	0	0	0	0	0	0
4	0	0	0	1	0	0
4	1	1	99	1	1	0
2	0	0	0	1	0	1
4	1	2	2	2	2	2
4	1	1	1	1	0	1
2	0	1	0	2	0	0
1	1	1	0	1	0	0
99	0	1	0	2	0	1
4	2	2	2	2	2	0
2	0	1	0	1	0	0
3	0	0	2	0	0	0
3	0	0	0	1	0	0
3	1	1	1	0	0	0
2	0	0	0	2	1	2
2	1	1	1	2	0	1
3	1	0	1	2	0	0
99	0	0	0	0	0	0
2	1	1	0	2	0	2
3	1	1	1	2	0	1
3	1	0	1	2	0	0
99	0	0	0	99	99	99
3	0	0	0	1	0	0
2	0	1	0	1	0	0
4	0	0	0	0	0	0
4	0	0	0	0	0	0
1	1	1	0	2	0	1
2	2	1	1	2	1	1
3	1	1	1	1	0	0
1	1	0	1	2	1	1
3	2	1	2	2	2	1
4	0	1	1	1	0	0
3	0	0	0	0	0	0

2	1	0	1	1	0	0
4	1	0	0	1	0	1
2	2	1	1	2	1	1
4	0	0	0	1	0	1
2	0	0	0	1	0	0
2	1	0	0	2	0	0
3	0	0	0	1	1	0
2	1	1	0	1	1	99
3	1	1	1	1	2	2
99	0	0	0	0	0	0
3	1	0	0	2	0	0
3	0	0	0	0	0	0
2	2	2	2	2	2	2
4	0	0	0	0	0	0
1	1	0	0	2	0	0
3	0	0	0	2	0	0
4	0	0	0	1	0	0
4	1	2	1	2	0	2
2	1	0	1	2	1	1
3	0	1	0	1	0	0
3	1	0	0	1	0	1
2	1	0	2	1	0	0
3	0	0	0	1	0	0
3	1	1	0	2	0	1
4	0	0	0	0	0	0
2	1	0	1	2	0	0
4	1	0	1	1	0	1
3	2	1	1	1	1	1
3	2	1	1	1	0	1
2	2	1	2	2	1	1
2	1	1	1	1	1	1
3	1	1	1	1	0	1
4	0	0	0	1	0	0
4	2	0	2	2	2	1
3	1	1	0	2	1	1
4	0	0	0	1	0	0
2	1	99	1	2	1	1
4	2	2	1	2	2	2
1	0	0	0	0	0	0
2	2	1	1	2	1	0
1	0	0	0	0	0	0
4	2	1	0	1	0	2
3	0	0	0	0	0	0
2	0	0	99	1	0	0
2	0	0	0	0	0	0
4	1	0	0	1	0	1
4	2	0	0	2	0	0
3	1	1	1	1	0	1

4	0	0	0	0	0	0
2	0	0	0	1	0	0
2	0	0	0	1	0	0
2	1	0	1	0	0	1
3	0	0	0	0	0	0
3	0	0	0	1	0	0
2	1	0	0	1	0	1
4	1	0	1	0	0	0
1	1	0	0	0	0	0
4	1	0	1	1	0	1
4	1	0	0	1	0	1
4	1	0	1	1	0	1
4	0	0	0	0	0	0
4	0	1	0	1	0	0
3	1	0	0	2	0	0
3	2	2	99	1	99	1
4	0	0	0	1	0	0
3	0	0	0	0	0	0
4	0	0	0	1	0	1
4	0	0	0	0	0	0
4	0	0	1	2	0	0
2	0	0	0	1	0	0
3	2	1	1	1	2	0
4	0	0	0	1	0	0
4	1	0	1	1	0	0
1	1	0	0	99	1	1
1	0	0	0	1	0	0
3	0	0	0	0	0	0
99	0	0	0	0	0	0
3	0	0	0	0	0	0
4	0	0	0	0	0	0
3	0	0	0	1	0	0
4	0	0	0	0	0	0
4	99	99	2	2	99	99
2	1	1	0	1	0	0
3	0	0	0	2	0	0
4	1	0	0	0	0	0
2	0	0	0	2	0	1
4	0	99	0	2	0	0
3	0	0	0	0	0	0
4	1	0	0	1	0	0
4	0	0	0	0	0	0
3	0	0	0	0	0	0
4	1	1	1	1	1	1
3	2	1	2	1	1	1
2	1	1	1	2	0	0
2	0	0	0	1	0	0
99	1	0	0	1	0	0

1	0	0	99	1	0	0
4	1	1	1	1	1	1
3	0	0	0	1	0	0
4	0	0	0	0	0	0
2	1	0	2	2	0	0
99	1	1	0	1	0	0
4	0	1	0	0	0	0
2	1	0	0	2	1	1
4	1	0	0	1	0	1
1	0	0	0	1	0	0
2	1	1	1	2	0	2
3	0	0	1	1	0	0
99	1	0	99	1	0	1
4	2	2	2	2	2	1
4	1	1	0	1	1	1
3	0	0	0	1	0	0
3	0	0	0	0	0	0
3	1	0	0	1	0	0
3	1	1	1	2	0	0
2	1	0	0	2	0	1
2	1	0	0	1	0	0
2	1	0	0	1	0	2
2	2	2	1	1	2	1
2	1	1	1	1	1	1
4	1	1	0	1	1	0
2	0	0	0	0	0	0
3	0	1	0	0	0	0
2	1	0	0	2	1	1
2	1	1	1	2	1	1
2	99	0	1	1	0	0
2	1	0	0	2	0	1
2	2	1	0	2	0	0
4	1	2	1	2	1	2
99	0	1	0	2	0	2
4	1	1	0	1	1	2
3	1	1	0	1	0	1
99	99	99	99	99	99	99
1	0	0	0	1	0	0
4	0	0	0	0	0	0
4	1	1	1	1	1	0
2	1	0	0	2	1	1
2	2	2	2	1	2	1
2	1	0	0	0	1	1
1	0	0	0	0	0	0
1	0	0	0	1	0	0
4	0	0	0	1	0	0
3	0	0	0	0	0	1
2	1	0	0	1	1	0

2	0	0	0	1	0	0
3	0	0	0	1	0	0
2	0	0	0	0	0	0
3	0	0	0	1	0	0
3	1	0	0	0	0	0
3	0	0	0	0	0	0
1	1	0	0	1	0	0
3	0	0	0	0	0	0
2	1	1	0	0	0	0
2	0	0	0	0	0	0
1	0	0	0	0	0	0
3	0	0	0	0	0	0
4	0	0	0	0	0	0
3	0	0	0	0	0	0
3	1	0	1	1	0	0
4	0	0	0	0	0	0
4	2	1	2	2	1	1
3	0	0	0	0	0	0
2	1	0	0	1	0	1
4	1	0	0	0	0	0
4	0	0	0	1	0	0
3	0	0	0	1	0	0
99	99	99	99	99	99	99
99	0	0	0	1	0	0
1	1	0	1	2	0	1
3	1	0	1	2	0	0
4	2	1	0	2	1	0
3	1	0	0	0	0	0
3	0	0	0	0	0	0
3	0	0	0	0	0	0
2	0	0	0	0	0	0
4	0	0	0	0	0	0
2	1	0	0	1	1	0
3	0	0	0	0	0	0
3	0	0	0	2	1	1
2	0	0	0	2	0	0
2	0	0	0	0	0	0
99	99	99	99	99	99	99
1	1	1	1	1	0	0
3	2	1	2	2	1	1
3	0	0	0	0	0	0
4	2	0	1	2	2	2
3	1	0	1	2	1	0
3	0	0	0	1	0	0
4	0	1	0	0	0	0
3	2	1	2	2	0	1
3	0	1	0	1	0	0
2	0	0	0	1	0	0

99	99	99	99	99	99	99
3	1	1	1	1	1	99
3	1	0	2	1	0	0
4	0	0	0	0	0	0
3	1	0	1	2	0	0
3	0	0	0	1	0	0
99	1	99	99	1	99	99
2	0	0	0	1	0	0
2	1	0	0	1	0	0
4	1	0	0	2	0	0
3	0	0	1	1	0	0
3	2	2	99	2	0	0
3	1	0	1	2	0	0
4	0	0	0	0	0	0
1	1	1	0	2	1	1
4	0	1	1	1	1	1
2	0	1	0	1	0	0
3	1	2	2	1	0	0
3	0	0	0	0	0	0
1	1	2	2	2	2	1
4	1	1	0	2	1	0
2	1	1	1	1	1	1
99	0	0	99	2	99	99
4	1	0	0	1	0	0
3	1	0	0	2	1	1
4	1	0	0	1	0	1
3	0	0	0	0	0	0
99	1	2	1	2	1	2
3	1	1	1	1	0	0
3	0	0	0	0	0	0
3	0	0	0	2	0	0
4	0	0	0	2	0	0
4	0	0	0	1	0	0
3	0	0	0	1	0	0
1	1	1	1	1	1	1
2	0	0	99	2	0	0
3	1	0	0	1	0	0
3	99	99	99	1	99	99
4	0	0	0	0	0	0
4	0	0	1	2	1	0
4	2	2	1	0	1	1
1	2	1	1	1	2	1
3	1	0	0	1	1	0
4	0	0	0	0	0	0
3	1	1	0	1	2	0
4	0	0	0	0	0	0
99	0	0	99	99	99	99
4	2	1	2	2	1	0

3	0	0	1	2	0	0
99	2	2	2	2	2	1
2	0	0	0	0	0	0
3	0	1	0	2	0	1
1	1	1	1	1	1	1
4	0	0	0	0	0	0
4	0	1	1	0	0	0
4	1	0	0	2	1	1
3	1	1	0	1	1	0
4	1	1	0	1	0	0
4	0	0	0	0	0	0
3	0	0	0	0	0	0
3	1	0	0	1	0	0
2	0	0	0	0	0	0
2	0	0	0	0	0	1
2	0	0	0	1	0	0
3	1	1	0	1	0	1
3	1	0	0	2	0	0
4	0	0	0	0	0	0
4	0	0	0	1	0	0
4	0	0	0	1	0	0
99	0	0	0	0	0	0
2	1	1	0	2	0	0
4	0	99	0	0	0	0
2	1	0	2	1	0	0
3	2	1	99	2	1	1
3	0	0	0	1	0	0
4	0	0	0	0	0	0
1	2	2	2	2	2	2
3	0	0	0	0	0	0
3	1	1	0	1	0	0
1	1	0	1	0	2	2
3	1	0	1	2	0	0
3	1	0	1	0	0	0
4	0	0	0	1	0	1
2	0	0	0	0	0	0
4	0	0	0	0	0	0
4	1	0	1	1	1	1
4	99	99	99	99	99	99
1	0	0	0	0	0	0
4	0	0	0	0	0	0
4	1	0	0	0	0	1
3	1	1	1	1	0	0
2	0	0	0	0	0	0
4	0	0	0	1	0	0
4	0	0	1	1	0	0
4	2	1	2	1	1	2
3	1	0	0	2	0	1

3	0	0	1	2	1	1
2	0	0	0	1	0	1
3	1	1	0	2	0	0
3	1	2	1	2	0	0
4	1	1	2	2	0	1
3	0	0	0	1	0	0
4	1	1	1	2	1	1
99	0	0	0	1	0	0
3	0	0	0	0	0	0
3	1	1	1	2	1	1
1	2	1	0	1	0	0
4	1	0	0	1	0	0
4	0	1	0	1	1	0
2	0	1	0	1	0	1
3	1	0	0	1	0	0
1	2	1	1	2	0	2
4	0	0	0	1	0	0
4	0	0	0	1	0	0
2	1	1	2	1	1	0
99	1	0	0	1	0	0
1	2	1	2	0	1	0
4	1	0	2	2	0	1
3	0	0	0	1	0	0
4	1	1	2	1	2	1
2	1	1	1	1	0	1
2	1	99	0	2	1	2
4	0	0	0	1	0	0
4	1	0	1	2	1	2
1	2	2	2	2	0	1
99	99	99	99	99	99	99
3	0	0	0	1	0	0
4	1	0	0	2	1	2
1	1	1	1	2	0	0
3	0	0	0	0	0	0
3	0	0	0	2	0	0
4	0	0	0	0	0	0
4	0	0	0	1	0	0
4	1	2	0	2	0	0
4	0	0	0	0	0	0
3	0	0	0	2	0	0
1	0	0	0	0	0	0
3	1	0	1	2	0	0
4	1	1	1	99	99	1
4	0	0	0	0	0	0
4	1	0	0	0	0	0
4	1	0	1	1	0	0
4	1	1	1	1	1	1
3	1	0	0	1	0	0

3	2	0	1	1	0	0
3	1	0	0	2	0	0
2	2	2	0	2	0	0
1	0	0	0	0	0	0
2	1	0	0	1	0	0
4	1	1	0	1	0	0
2	0	1	0	1	0	0
4	1	0	0	1	0	0
4	1	0	0	1	0	0
4	0	0	1	0	1	0
2	0	0	0	0	0	0
3	1	0	1	0	0	0
4	0	0	0	1	0	99
4	1	1	0	0	0	1
2	0	0	0	1	0	0
4	0	0	0	1	0	1
2	0	0	0	1	0	0
4	1	1	2	0	0	0
2	0	0	0	0	0	0
4	0	0	1	2	0	0
2	2	1	1	0	0	0
4	2	1	2	2	1	0
3	1	1	1	1	0	1
1	1	2	0	1	0	0
4	1	1	0	2	0	0
3	0	0	0	1	0	0
3	0	0	0	1	0	0
2	0	0	0	0	0	0
99	0	0	0	1	0	0
4	1	0	0	0	0	0
3	2	1	2	1	0	1
4	0	0	0	0	0	0
2	1	1	2	2	1	1
3	1	0	0	2	0	0
4	1	0	0	1	0	0
2	0	0	1	1	0	0
1	0	1	1	0	1	1
2	1	0	1	2	1	2
1	0	0	0	1	1	1
2	1	1	1	2	2	1
2	0	0	0	1	0	0
4	1	0	2	2	0	0
2	0	0	0	1	0	0
2	1	1	1	0	0	0
3	1	0	0	0	0	0
3	1	1	0	2	0	0
2	2	2	2	2	2	2
3	1	1	1	2	1	0

4	2	0	1	2	0	0
2	0	0	0	1	0	0
4	1	0	1	0	0	0
2	2	2	2	2	2	2
3	1	1	1	2	1	1
3	1	0	1	1	0	0
1	0	0	0	1	0	1
4	0	2	1	1	2	2
4	1	1	1	1	1	1
2	99	1	0	0	0	0
2	1	1	1	1	1	2
3	1	0	1	1	1	2
2	1	1	1	2	0	1
2	0	0	0	2	0	1
2	1	1	1	1	1	1
1	0	0	0	2	0	1
4	0	0	0	2	1	0
2	0	0	0	1	0	0
99	1	99	99	2	99	99
2	1	0	1	2	1	1
4	0	0	0	0	0	0
2	1	1	0	2	1	0
4	0	0	0	1	0	0
3	1	0	0	1	0	0
4	1	0	0	0	0	0
3	1	99	99	1	0	99
2	1	1	0	2	0	0
2	0	0	0	0	0	0
2	0	0	0	2	0	0
3	2	2	2	2	2	2
99	0	0	0	0	0	0
3	0	0	0	1	0	0
2	0	0	0	0	0	0
99	1	0	0	1	1	1
3	0	0	0	0	0	0
4	1	0	2	1	2	2
4	1	0	1	2	2	0
1	1	2	1	2	1	1
2	0	0	0	0	0	0
4	1	0	1	2	0	0
4	0	0	1	0	0	1
4	0	99	99	1	0	0
3	1	1	0	1	0	0
4	0	1	0	0	0	0
1	1	0	0	2	0	1
4	0	0	0	99	0	0
4	0	0	0	0	0	0
2	2	0	2	2	0	0

4	1	0	0	2	0	1
99	1	0	0	1	0	0
4	1	0	0	0	0	0
4	2	0	1	1	0	0
2	1	0	0	1	1	1
99	0	0	0	0	0	0
4	1	0	2	2	0	0
3	0	1	0	2	0	1
2	1	1	1	2	1	0
4	1	1	1	1	0	1
2	99	99	99	99	99	99
4	2	2	2	2	2	1
4	1	1	0	1	0	0
3	1	1	0	2	0	1
3	1	1	1	0	1	0
4	0	0	0	1	0	0
2	0	0	0	1	0	0
3	0	0	0	0	0	0
3	1	1	0	2	2	1
2	2	1	2	0	0	1
4	1	0	2	1	1	1
4	0	1	0	0	0	0
2	1	1	1	0	0	0
3	1	0	2	2	2	0
4	2	1	1	2	0	0
1	2	1	2	2	1	1
99	0	1	0	1	0	1
3	0	0	0	0	0	1
1	1	1	0	0	0	1
2	2	2	2	2	2	2
4	1	1	0	2	0	0
4	1	1	0	1	0	0
2	0	0	0	0	0	0
2	0	0	0	1	0	2
3	1	1	2	2	1	0
2	2	1	2	1	1	1
2	0	0	0	1	1	0
3	0	1	0	2	0	0
2	0	0	2	2	1	1
2	1	0	0	2	0	0
2	1	0	0	1	0	0
4	0	0	0	2	0	1
2	1	1	1	1	1	1
3	2	1	1	2	0	2
1	0	0	0	1	0	0
4	0	0	1	0	0	0
2	0	0	0	1	0	0
3	0	0	0	1	0	1

4	0	0	0	0	0	0
2	2	0	0	1	0	1
4	0	0	0	1	0	0
4	1	1	1	2	1	1
2	0	0	0	2	0	0
2	1	0	0	1	0	0
99	1	0	0	1	1	1
3	1	0	99	1	1	1
99	1	0	1	1	1	1
2	0	0	0	1	0	0
2	1	0	1	2	1	0
4	1	0	0	1	0	0
3	2	1	1	2	1	1
3	0	0	0	0	0	0
3	0	0	0	1	0	0
4	0	0	0	1	0	0
2	0	0	0	0	0	0
2	0	0	0	1	0	0
3	0	0	1	1	0	0
3	0	0	0	0	0	0
4	0	0	0	0	0	0
4	1	1	0	1	0	0
4	1	0	0	2	0	1
1	99	99	99	99	99	99
2	1	0	1	2	0	0
3	2	1	2	1	0	1
2	0	0	0	0	0	0
1	1	0	0	2	0	1
3	0	0	0	1	0	0
4	0	0	0	0	0	0
1	0	0	0	2	1	0
4	2	1	1	2	2	2
1	0	0	0	2	0	0
3	0	1	0	1	0	0
2	1	2	2	1	2	99
3	1	1	1	1	1	2
3	2	1	0	2	0	2
4	1	1	0	1	0	1
2	0	0	0	2	0	0
1	1	1	1	1	0	1
3	0	0	0	0	0	0
1	2	0	2	2	2	1
4	0	0	0	0	0	0
4	1	1	1	0	0	0
3	1	99	99	1	0	0
2	1	0	0	1	1	0
3	1	0	0	1	0	0
2	2	0	2	2	2	2

2	0	0	0	2	0	0
3	0	0	0	0	0	0
1	1	0	1	2	1	1
3	0	99	99	99	99	99
4	1	0	0	1	0	0
2	0	0	0	1	0	0
4	2	2	2	2	2	2
2	1	0	0	1	0	0
4	0	0	0	0	0	0
4	2	0	0	2	0	0
2	2	1	1	2	1	2
3	1	0	0	1	0	0
3	1	0	1	2	1	0
4	0	0	0	1	0	0
2	1	0	1	2	0	1
3	2	2	2	2	2	2
3	2	2	2	2	2	2
2	1	1	1	1	1	1
4	1	1	1	1	1	1
3	1	0	0	2	0	0
4	2	0	2	2	2	2
4	0	0	0	0	0	0
4	0	0	1	1	1	1
1	0	0	0	1	1	1
4	0	0	0	1	0	0
4	1	1	1	2	1	1
2	2	2	1	2	1	2
2	1	1	1	2	1	1
4	1	1	0	2	0	0
4	0	0	0	1	0	0
4	0	0	0	0	0	0
4	1	0	0	2	0	1
2	1	1	1	2	1	1
4	0	0	0	1	0	0
3	2	1	1	2	1	1
2	1	1	0	1	0	0
2	2	2	2	2	2	2
4	0	0	0	0	0	0
4	1	0	0	1	0	0
2	1	1	1	1	1	1
3	0	0	0	0	0	0
2	1	0	0	1	0	0
4	1	0	0	1	2	2
3	1	0	0	2	0	0
2	0	0	0	1	0	0
4	0	0	0	0	0	0
3	1	0	0	0	0	0
4	1	1	0	1	0	0

4	1	0	0	0	0	0
1	2	0	0	1	0	1
2	1	0	0	0	0	0
2	0	1	0	1	0	0
3	0	0	0	1	0	0
3	0	0	0	0	0	0
99	0	0	0	0	0	0
3	0	0	0	0	0	0
4	0	0	0	0	0	0
3	1	0	0	1	1	0
4	1	0	0	0	2	0
2	0	0	0	1	0	2
2	0	0	0	2	1	0
3	0	0	0	0	0	0
4	0	0	0	0	0	0
4	0	1	0	1	0	0
2	1	0	1	1	1	0
3	0	1	1	2	0	0
1	0	0	0	1	0	0
4	1	1	0	2	0	1
4	1	0	0	1	0	1
2	1	1	2	2	1	1
4	1	0	0	1	0	1
2	0	0	0	0	0	0
2	0	0	2	1	0	1
3	0	0	0	1	0	0
2	1	0	0	1	2	0
4	0	0	0	0	0	0
3	0	0	0	1	0	1
1	0	0	0	0	0	0
3	2	0	0	1	0	0
2	0	0	0	0	0	0
3	1	1	1	2	0	1
4	0	0	0	0	0	0
99	1	0	0	1	0	0
3	1	0	0	2	0	0
4	0	0	0	0	0	0
3	1	0	1	1	0	0
3	1	0	0	1	0	0
3	1	0	1	2	0	1
3	0	0	0	0	0	0
4	0	0	0	1	0	0
4	2	1	1	2	1	1
4	2	1	1	1	0	0
4	1	1	0	2	0	0
3	1	0	1	1	1	1
1	1	0	0	1	0	0
2	1	1	1	0	0	0

4	0	0	0	0	0	0
2	2	2	1	2	0	1
4	0	0	0	0	0	0
2	1	0	99	1	0	1
4	1	1	0	0	0	0
3	1	0	0	1	0	0
2	1	1	1	2	0	0
2	1	1	1	2	0	0
3	1	0	0	2	1	2
3	2	2	2	2	2	2
2	1	2	2	1	2	1
4	1	1	1	0	0	0
3	1	1	1	2	1	0
3	0	0	0	1	0	0
2	2	1	2	2	2	2
99	1	1	1	1	1	1
4	1	0	0	1	0	0
3	0	0	0	0	0	0
4	0	0	0	1	0	0
1	0	0	0	1	0	0
1	0	0	0	1	0	0
4	1	0	0	1	0	0
99	2	1	2	2	2	99
4	0	0	0	0	0	0
99	0	0	0	1	0	0
4	0	1	0	2	0	0
3	0	0	0	1	0	0
4	0	0	0	1	0	0
3	1	0	1	2	0	0
4	0	0	0	0	0	1
2	1	1	1	1	1	1
2	1	1	0	2	1	1
3	1	0	0	1	0	0
4	0	0	0	0	0	0
4	1	1	0	2	1	1
3	1	1	2	2	1	0
2	0	0	0	0	0	0
3	1	1	0	2	0	2
3	1	0	0	1	0	0
3	0	0	0	0	0	0
2	0	0	0	1	0	0
99	0	0	99	99	99	99
4	1	0	1	2	1	0
3	1	1	2	2	0	1
3	0	0	0	0	0	0
2	1	1	0	2	1	2
4	1	0	1	1	0	1
3	0	1	99	99	0	0

4	0	0	0	1	0	0
3	1	2	0	2	0	1
4	2	1	2	2	1	1
4	0	0	0	0	0	0
4	0	0	0	0	0	0
3	2	1	2	1	2	0
3	0	0	0	0	0	0
3	0	0	0	1	0	0
4	0	0	0	0	0	1
4	2	2	2	2	2	2
4	0	0	0	0	0	0
4	0	0	0	0	0	0
4	0	0	0	2	0	1
1	0	0	0	0	0	0
4	1	1	2	1	1	0
3	0	0	0	0	0	0
4	1	2	1	2	0	0
3	1	0	1	1	0	0
99	0	1	0	1	0	0
4	0	0	0	0	0	0
1	1	1	1	2	1	2
2	0	0	1	1	0	0
3	0	0	0	1	0	1
99	1	1	1	1	0	0
3	0	0	0	0	0	0
2	1	1	1	2	1	0
4	0	0	0	0	0	0
3	1	1	1	2	1	2
3	1	0	1	2	0	1
3	1	0	0	1	0	0
4	0	0	0	2	0	0
3	1	1	0	0	0	0
4	0	0	0	0	0	0
1	0	1	0	0	0	0
3	1	1	2	1	2	2
4	1	1	1	2	0	0
4	0	0	99	2	0	0
3	1	0	0	0	0	0
2	1	1	1	2	1	1
3	0	0	0	1	0	1
1	1	1	1	2	2	0
4	2	2	1	1	1	1
4	0	0	0	1	0	0
3	1	0	1	0	0	0
3	1	2	1	1	1	2
1	0	0	99	2	2	0
3	1	1	2	2	1	0
3	1	1	1	1	1	1

2	1	0	0	1	0	0
1	1	0	0	1	0	0
4	1	1	1	1	1	1
#NULL!	0	0	0	0	0	0
#NULL!	2	1	2	1	0	2
#NULL!	0	1	0	1	0	0
#NULL!	0	0	0	0	0	0
#NULL!	1	0	0	0	0	0
#NULL!	1	0	0	2	0	0
#NULL!	1	0	0	2	0	0
#NULL!	1	0	1	1	1	0
#NULL!	2	1	1	2	1	1
#NULL!	2	2	1	2	1	1
#NULL!	1	1	1	1	0	1
#NULL!	1	1	1	2	1	0
#NULL!	1	0	1	2	1	0
#NULL!	0	0	1	1	0	0
#NULL!	1	0	1	2	1	0
#NULL!	1	1	1	0	0	0
#NULL!	2	1	2	2	0	0
#NULL!	2	1	2	2	0	0
#NULL!	2	1	2	2	1	1
#NULL!	1	1	2	2	1	1
#NULL!	0	0	0	0	0	0
#NULL!	0	0	0	2	0	0
#NULL!	1	1	1	1	1	1
#NULL!	1	1	1	1	1	0
#NULL!	0	0	0	1	0	0
#NULL!	1	0	0	2	0	0
#NULL!	1	0	0	0	0	0
#NULL!	0	0	0	0	0	0
#NULL!	1	2	2	2	2	2
#NULL!	2	2	2	2	0	0
#NULL!	1	1	0	1	0	0
#NULL!	1	0	1	2	0	0
#NULL!	2	1	2	2	0	0
#NULL!	0	0	0	0	0	0
#NULL!	1	0	0	0	0	0
#NULL!	2	1	0	2	0	0
#NULL!	0	0	0	0	0	0
#NULL!	0	0	0	0	0	0
#NULL!	0	0	0	1	0	0
#NULL!	1	1	0	2	0	0
#NULL!	0	0	0	1	0	0
#NULL!	0	0	0	0	0	0
#NULL!	1	1	1	1	1	1
#NULL!	0	0	0	1	0	0
#NULL!	0	0	0	2	0	0

#NULL!	0	0	0	0	0	0
#NULL!	1	1	0	1	0	0
#NULL!	0	0	0	1	0	0
#NULL!	0	0	0	1	0	0
#NULL!	0	0	0	2	0	0
#NULL!	0	0	0	0	0	0
#NULL!	1	1	0	2	0	1
#NULL!	0	0	0	0	0	0
#NULL!	0	0	0	1	0	0
#NULL!	1	1	1	1	0	1
#NULL!	0	0	0	1	0	0
#NULL!	0	0	0	0	0	0
#NULL!	#NULL!	#NULL!	#NULL!	#NULL!	#NULL!	#NULL!
#NULL!	1	0	0	1	0	1
#NULL!	0	0	0	2	0	0
#NULL!	0	0	0	0	0	0
#NULL!	0	0	0	2	0	0
#NULL!	0	0	0	0	1	0
#NULL!	1	0	0	1	0	0
#NULL!	1	0	0	1	0	0
#NULL!	0	0	0	0	0	0
#NULL!	2	2	1	2	1	1
#NULL!	0	0	0	0	0	0
#NULL!	0	0	0	0	0	0
#NULL!	1	1	1	1	0	1
#NULL!	0	0	0	1	0	0
#NULL!	1	0	0	2	1	1
#NULL!	0	0	0	0	0	0
#NULL!	0	0	0	1	0	0
#NULL!	0	0	0	0	0	0
#NULL!	0	0	0	0	0	0
#NULL!	0	0	0	2	0	0
#NULL!	1	0	0	1	0	1
#NULL!	0	0	0	0	0	0
#NULL!	1	0	0	1	0	0
#NULL!	0	0	0	1	0	0
#NULL!	0	0	0	0	0	0
#NULL!	0	0	0	1	0	0
#NULL!	0	0	0	1	0	0
#NULL!	0	0	0	1	0	0
#NULL!	1	0	0	1	0	0
#NULL!	0	0	0	0	0	0
#NULL!	1	0	0	1	0	0
#NULL!	0	0	0	0	0	0
#NULL!	0	0	0	2	0	0
#NULL!	1	1	1	1	0	1
#NULL!	1	0	1	2	0	0
#NULL!	0	0	1	0	0	0

#NULL!	0	0	2	2	0	0
#NULL!	1	0	0	1	0	0
#NULL!	0	1	1	2	0	0
#NULL!	0	0	0	0	0	0
#NULL!	1	0	1	2	0	0
#NULL!	1	0	1	2	0	0
#NULL!	0	0	0	2	0	0
#NULL!	0	0	0	2	0	0
#NULL!	0	0	0	1	0	0
#NULL!	1	0	1	2	1	2
#NULL!	0	0	0	2	0	0
#NULL!	0	0	0	1	0	0
#NULL!	1	0	0	1	0	0
#NULL!	1	1	0	1	1	2
#NULL!	1	1	0	2	0	0
#NULL!	0	0	0	1	0	0
#NULL!	#NULL!	#NULL!	#NULL!	#NULL!	#NULL!	#NULL!
#NULL!	2	2	2	2	2	2
#NULL!	1	1	1	2	0	1
#NULL!	1	0	0	1	0	0
#NULL!	0	0	0	0	0	0

pccommg_pre	pccommh_pre	pccommi_pre	pccommj_pre	pccommk_pre	bullya_pre	bullyb_pre
0	0	0	0	0	0	0
0	0	0	0	0	0	0
0	0	0	0	0	1	4
1	0	0	0	1	4	4
0	0	0	0	2	0	0
99	99	99	99	99	99	99
1	2	2	2	2	0	0
0	0	1	0	0	1	1
99	99	99	99	99	99	99
0	0	2	1	1	1	0
0	0	0	0	2	0	1
0	0	1	0	2	2	0
0	0	0	0	1	2	2
0	0	0	0	0	2	99
0	0	0	0	0	0	0
0	1	1	1	2	1	1
0	0	0	0	0	0	0
0	0	0	0	0	0	1
2	0	2	2	2	1	99
0	1	2	1	2	4	3
99	99	99	99	99	99	99
0	0	0	0	0	0	0
0	0	0	0	0	0	0
1	0	1	0	2	0	0
0	0	1	0	2	0	0
0	0	0	0	0	0	1
0	0	0	0	0	0	1
0	0	0	0	0	1	2
0	0	1	0	0	0	0
99	0	0	0	0	2	1
0	0	0	1	0	1	3
1	1	1	0	2	2	0
0	0	0	0	0	2	1
0	0	0	0	0	0	0
99	0	0	0	0	99	1
1	0	1	1	2	4	4
0	0	0	0	1	1	1
0	0	0	0	0	2	2
0	0	1	0	2	4	4
0	1	1	1	2	3	3
0	0	0	0	1	3	3
0	0	0	0	0	0	0
1	0	1	1	0	99	99
0	0	0	0	0	1	0
0	0	2	1	2	0	0
0	0	1	1	2	0	3
0	0	0	0	0	2	0

0	0	0	1	2	0	0
0	0	1	1	1	2	0
0	0	2	2	2	0	0
1	0	1	1	1	3	3
0	0	1	1	0	2	2
0	0	1	1	1	1	0
0	0	0	0	1	0	0
0	1	0	0	1	1	0
0	0	0	0	0	0	0
0	0	1	0	0	0	1
0	0	0	0	0	1	0
0	0	0	0	1	0	0
0	0	0	0	0	1	1
1	0	2	1	2	2	2
0	0	0	0	0	1	0
0	1	2	0	2	1	4
2	1	1	1	2	4	2
0	0	2	1	2	1	0
0	0	1	0	0	4	3
0	0	1	0	0	2	1
1	0	2	0	1	1	1
0	0	2	2	2	2	0
0	0	0	0	1	4	4
0	0	2	0	0	4	4
1	0	1	1	2	1	0
0	0	0	0	0	0	0
0	0	0	0	0	2	1
2	2	2	2	2	0	0
0	0	0	0	1	1	1
0	0	1	0	2	1	0
0	0	2	0	0	4	0
0	0	1	0	2	2	3
99	0	2	2	1	2	0
0	0	2	2	2	3	2
0	0	1	0	2	4	2
0	0	0	0	0	1	0
0	0	0	0	0	2	4
0	1	1	1	2	3	0
0	1	2	2	1	3	2
2	0	2	0	2	2	0
1	0	1	2	1	2	0
0	0	0	0	0	1	0
0	0	1	1	1	2	2
0	0	0	0	0	0	0
1	1	1	1	2	0	0
0	0	0	0	0	0	0
0	0	0	0	0	1	1
1	0	1	0	0	3	0

0	0	1	1	1	2	0
0	0	1	1	0	2	2
0	1	1	1	1	0	0
0	0	1	1	2	2	0
0	0	2	2	1	0	0
0	0	1	0	1	0	0
2	0	1	1	2	2	3
0	0	2	0	0	1	0
0	0	1	0	0	4	3
2	2	2	2	2	4	4
0	0	0	1	1	1	1
0	0	0	0	0	1	0
2	0	2	2	2	4	1
0	0	2	1	2	2	2
0	0	0	2	2	0	0
2	0	0	0	2	1	4
2	1	2	2	2	2	2
0	0	1	0	2	0	2
1	1	1	1	2	0	0
0	0	1	0	0	0	0
0	0	2	2	2	4	1
1	1	1	2	2	1	3
0	0	0	0	0	0	0
2	0	0	0	1	4	4
2	2	2	2	2	1	0
1	1	1	1	1	4	0
99	1	1	1	1	3	4
0	0	1	0	0	1	0
0	0	0	0	2	0	0
0	0	1	1	1	1	0
0	0	0	0	1	99	99
0	0	1	0	0	4	2
99	99	99	99	99	99	99
1	0	1	1	99	99	0
1	1	1	1	1	0	0
0	0	0	0	2	1	3
0	0	0	0	0	0	0
1	1	1	1	2	3	0
0	0	1	1	2	2	2
0	0	0	0	0	4	3
0	0	0	0	2	0	1
2	2	2	2	2	3	0
0	0	0	2	2	2	4
0	0	0	0	0	1	2
0	0	1	0	1	0	1
0	0	0	0	0	99	99
0	0	1	1	2	4	4
0	0	1	1	2	3	4

0	0	1	0	1	1	0
2	2	1	1	2	0	0
0	0	1	0	2	1	0
1	1	1	1	2	2	1
0	0	0	1	1	0	0
99	99	99	99	99	99	99
0	0	0	0	0	0	0
0	0	0	0	0	1	0
0	0	0	0	0	3	0
0	0	0	0	1	4	2
0	0	1	0	1	1	1
0	0	0	0	0	0	0
0	0	0	0	0	1	0
0	0	0	0	0	2	2
0	0	1	1	1	2	0
0	1	2	1	1	2	0
0	0	1	0	0	1	0
0	0	1	1	0	1	0
0	0	2	0	2	4	2
1	0	0	0	0	0	0
0	0	0	0	0	2	0
0	0	0	0	0	0	0
1	1	1	1	1	0	0
0	0	1	0	1	1	0
0	0	0	0	1	0	0
0	0	0	0	1	1	0
0	0	0	0	1	3	2
0	99	99	0	0	4	0
0	0	0	0	0	2	1
0	0	1	0	0	0	0
0	0	0	0	0	1	0
0	0	0	0	99	0	1
0	0	0	0	0	0	0
0	0	0	0	0	0	0
0	0	1	0	0	2	3
0	0	1	1	1	2	1
0	0	0	0	0	0	0
0	0	0	0	0	1	0
0	0	0	0	0	4	0
0	0	1	1	0	1	0
0	0	1	1	1	0	1
0	0	0	1	0	2	2
0	1	2	1	2	4	4
1	0	0	0	2	4	3
0	0	1	0	0	1	2
1	1	2	1	2	0	0
0	1	1	1	1	1	1
0	0	1	0	1	0	0

0	0	0	0	0	2	3
0	0	1	0	0	0	1
0	0	0	0	0	4	4
0	0	0	0	1	0	0
0	0	0	0	2	3	0
0	0	0	0	0	2	0
0	0	0	0	0	2	2
0	2	2	2	2	3	0
0	0	0	0	0	0	0
0	0	1	1	2	4	3
0	0	0	0	0	1	1
0	0	1	1	0	2	0
0	2	1	0	2	3	2
99	99	99	99	99	99	99
1	1	1	1	1	1	0
2	2	2	2	1	2	2
1	0	1	0	0	0	0
0	0	0	0	0	0	0
1	0	2	1	1	2	2
1	0	0	0	1	0	0
0	0	0	0	0	0	0
0	0	0	0	0	0	0
0	2	1	2	0	0	0
0	0	0	0	0	0	0
0	0	1	0	1	4	4
0	0	2	2	0	3	4
0	0	0	0	0	3	4
0	0	1	0	2	4	0
0	0	1	0	2	4	4
0	0	0	0	0	0	0
2	0	2	2	2	2	0
1	0	1	1	1	4	0
2	2	2	2	2	2	0
2	0	1	0	2	0	0
2	1	1	2	2	3	2
2	0	2	0	0	4	4
0	0	0	0	0	0	1
0	0	0	0	0	2	0
0	0	0	0	0	0	0
2	1	2	1	2	2	1
0	0	0	0	0	2	2
0	0	0	0	0	1	0
1	1	1	1	1	1	0
0	0	1	0	0	1	0
0	0	0	0	0	1	1
0	0	2	0	0	2	0
0	0	0	0	0	0	0
0	0	0	0	0	3	0

0	0	0	0	1	2	1
1	1	1	1	2	4	4
0	0	0	0	0	4	1
0	0	0	0	0	0	0
0	0	2	1	1	3	3
0	0	0	0	1	0	0
1	0	1	0	0	4	4
0	0	1	99	0	4	2
0	1	1	2	2	1	1
0	0	0	1	0	1	1
0	0	0	0	0	1	1
0	0	0	0	0	0	0
0	0	0	0	1	3	0
0	1	0	1	2	0	1
0	0	0	0	0	1	0
1	0	2	1	2	1	2
0	0	0	0	1	4	4
0	0	0	0	0	1	1
0	1	1	1	2	4	4
1	0	1	1	0	0	0
0	0	0	1	2	1	1
1	0	0	0	2	4	4
0	0	0	0	1	1	0
0	0	0	0	0	2	1
0	0	0	0	0	4	3
0	0	1	0	1	2	0
0	0	0	0	0	1	0
0	1	1	0	0	1	1
2	1	1	1	2	2	1
0	0	0	0	0	2	0
0	0	2	0	2	2	1
0	0	1	0	0	3	4
0	0	1	1	1	2	0
0	0	2	2	2	1	0
0	0	0	0	2	0	0
0	1	0	0	1	0	0
0	0	0	1	0	2	1
0	0	1	0	1	2	1
0	0	1	1	1	2	1
1	2	2	2	2	1	4
1	0	1	0	2	2	2
0	0	0	0	0	0	0
1	0	1	0	0	0	3
0	0	0	0	0	0	0
0	0	2	0	0	1	0
1	0	0	0	2	4	0
0	0	1	0	0	1	0
2	0	2	1	2	2	0

0	0	1	0	0	0	0
0	0	1	0	0	2	1
0	0	1	0	0	1	2
0	0	0	0	0	1	0
0	0	1	0	2	2	0
0	0	0	0	0	1	0
0	0	1	0	0	1	0
0	0	0	0	0	2	0
0	1	2	2	2	1	1
0	1	1	2	2	2	4
0	0	1	0	0	0	0
0	0	0	0	0	0	0
0	0	1	1	1	2	1
1	0	1	0	1	1	2
0	0	1	0	0	4	3
1	0	1	0	1	0	0
0	0	1	0	1	0	0
0	0	2	2	1	3	1
1	1	2	2	1	0	0
1	0	2	2	2	3	3
1	0	2	1	2	3	2
0	0	2	0	2	3	2
0	1	0	1	1	0	0
1	1	2	1	1	0	0
0	1	2	1	0	2	2
0	0	0	0	0	0	0
0	0	0	0	2	1	1
0	0	1	1	1	4	4
99	0	0	0	2	2	0
0	0	0	0	0	1	1
2	0	1	2	2	0	1
0	0	0	0	0	2	1
0	1	2	2	1	4	4
0	0	0	0	0	0	0
0	0	0	0	0	0	2
0	0	0	0	1	4	0
0	0	0	0	1	2	2
0	0	0	0	0	0	0
0	0	1	0	0	0	0
0	0	0	0	0	2	2
0	2	2	2	0	2	2
0	0	0	0	2	1	0
0	0	0	0	0	0	0
0	0	0	1	2	4	1
0	1	1	1	1	2	1
0	0	1	1	2	2	2
0	0	0	0	1	4	0
0	0	0	0	0	0	0

0	0	1	0	2	1	0
0	0	1	1	1	2	1
0	0	1	2	2	1	2
1	0	1	1	1	2	0
0	0	1	0	2	1	2
0	0	0	0	0	0	99
0	0	0	0	0	4	4
0	0	2	2	1	0	1
0	2	2	2	2	0	0
0	0	2	2	2	4	0
0	1	1	1	2	0	1
0	0	1	1	0	0	0
0	0	0	0	1	1	1
2	0	0	2	2	4	2
0	0	1	1	1	2	2
0	2	2	2	2	1	1
0	0	0	0	0	3	1
1	1	1	1	1	2	1
0	0	0	0	1	0	2
0	0	1	0	0	0	0
0	0	0	1	1	1	1
0	0	1	0	0	1	3
1	0	1	0	2	1	0
1	0	1	1	0	1	1
0	1	1	1	1	4	3
0	0	1	0	1	2	1
0	0	1	2	2	0	0
99	99	99	99	99	0	0
0	0	0	0	1	0	0
0	0	1	1	1	0	0
1	0	1	0	0	0	4
0	0	1	1	0	1	0
0	0	1	0	1	0	0
0	0	0	0	1	1	1
0	0	0	0	0	3	4
1	0	0	0	1	0	1
0	2	1	1	0	4	3
0	1	2	0	2	0	0
1	2	2	2	2	0	1
1	0	0	0	2	1	2
0	0	1	0	0	0	0
0	0	0	0	0	0	3
0	0	0	0	0	1	1
1	0	1	0	2	2	1
0	0	0	1	2	4	4
0	0	0	0	0	0	0
0	0	1	0	1	1	1
0	0	0	0	0	0	0

0	0	1	0	1	0	0
0	0	0	0	0	0	0
2	0	1	2	2	0	0
0	0	1	0	1	4	2
0	0	0	0	0	4	4
2	0	2	0	2	0	0
1	0	1	0	0	1	2
0	1	1	1	2	4	4
0	0	1	0	0	3	0
0	0	1	0	0	4	4
0	0	1	0	0	0	0
0	2	2	2	2	2	2
0	0	2	1	1	1	0
0	0	2	0	0	0	0
0	0	0	0	2	0	0
0	0	0	0	1	4	2
0	0	2	2	1	4	4
0	0	0	0	1	2	2
0	0	0	0	0	1	0
0	0	1	0	0	2	1
0	0	0	0	0	2	2
0	1	0	0	1	4	2
0	1	1	1	2	1	1
0	0	0	99	1	1	0
0	0	0	0	0	4	4
0	99	2	2	2	1	1
1	0	1	0	0	2	1
1	1	0	1	1	0	0
0	0	0	0	0	0	0
0	0	0	0	0	1	0
1	0	0	0	2	1	1
1	1	1	1	1	1	1
0	0	0	0	0	2	3
0	0	1	0	1	3	2
0	1	1	1	0	0	3
1	0	1	1	2	0	0
2	2	2	2	2	3	0
0	0	1	1	1	4	4
0	0	1	2	2	0	1
0	0	0	0	0	0	0
2	2	2	2	2	4	4
0	0	0	0	0	4	4
0	2	1	2	2	0	0
99	99	99	99	99	99	99
1	1	2	2	2	2	2
0	0	2	1	2	4	2
2	0	1	0	2	0	0
0	2	1	0	2	4	2

0	0	0	0	2	2	0
0	0	1	0	2	3	2
0	0	0	1	2	0	0
0	0	0	0	0	2	0
0	0	0	0	1	0	1
0	0	0	0	2	3	1
0	0	0	0	0	0	0
1	0	1	1	1	3	2
2	0	0	0	2	3	3
1	1	1	1	1	0	0
2	2	2	2	2	3	2
1	1	1	1	1	2	1
0	2	2	2	2	0	0
2	2	2	2	2	2	1
0	1	1	1	1	1	4
0	0	1	2	2	2	1
0	0	0	0	0	1	0
0	0	0	0	0	1	0
0	0	2	2	0	4	2
1	1	1	1	1	2	1
1	1	1	1	1	0	0
2	1	1	0	1	2	2
1	1	1	1	2	1	2
0	0	1	1	1	0	0
0	2	2	2	2	2	1
0	0	0	0	1	3	2
0	2	2	2	2	4	3
0	0	0	0	0	3	3
0	0	0	0	0	0	1
1	0	1	0	2	2	2
0	0	0	0	0	4	4
1	0	2	2	2	3	0
1	1	1	1	2	0	1
2	1	2	2	2	2	3
2	0	2	1	1	4	1
0	0	0	0	2	0	0
0	2	2	2	2	3	2
0	0	0	2	1	0	1
1	0	1	1	1	0	0
0	0	2	1	2	3	0
1	0	0	0	1	3	4
0	0	1	1	0	1	0
2	1	2	0	2	0	2
0	2	2	2	0	2	1
0	0	1	1	2	0	0
0	0	0	0	0	0	0
0	0	1	0	1	0	0
0	0	2	0	2	2	4

1	0	2	1	0	4	2
0	0	1	0	2	2	1
0	0	1	1	1	3	1
0	0	0	0	0	1	0
1	0	2	2	2	2	1
2	0	0	1	99	2	4
0	0	1	0	0	1	0
0	0	2	1	2	2	1
1	2	2	2	2	1	1
0	0	0	0	0	0	0
2	0	1	1	1	2	0
2	2	2	2	2	0	0
0	0	1	0	0	0	1
0	0	0	0	2	1	2
0	0	0	0	0	0	0
0	0	0	0	0	0	0
0	0	0	0	0	0	0
0	0	0	0	0	0	0
0	0	1	1	0	0	1
0	0	2	1	0	0	0
0	0	0	0	0	0	0
0	0	0	0	1	0	99
0	0	0	0	1	0	0
0	0	1	1	1	3	0
99	99	99	99	99	99	99
0	0	1	0	2	3	4
0	0	0	0	0	2	2
2	0	2	0	0	2	2
0	0	1	0	1	2	2
0	0	0	0	1	1	0
2	0	1	1	1	2	0
0	0	1	0	0	1	0
0	0	1	0	1	1	1
0	0	1	1	1	1	0
0	0	0	0	0	0	0
0	0	2	2	2	1	0
0	0	0	0	0	3	4
0	0	0	0	0	2	4
0	1	2	2	1	2	1
0	1	1	2	1	1	0
0	0	1	0	2	4	1
0	0	0	0	0	0	0
0	0	1	0	2	1	1
0	0	1	1	2	0	1
2	2	2	2	1	4	4
0	0	2	0	2	2	0
2	0	1	1	2	2	0
0	0	0	0	0	0	0

1	0	0	0	2	1	0
0	0	2	0	0	4	2
0	0	1	0	2	4	0
0	1	2	2	1	3	2
0	0	2	1	2	4	3
1	0	1	0	0	0	0
0	0	0	0	0	1	0
0	0	0	0	2	0	0
0	0	0	1	2	1	0
0	0	0	0	1	2	2
99	0	1	0	0	4	3
0	0	0	0	2	0	0
1	0	1	1	0	1	0
0	0	0	0	1	1	0
99	99	99	99	99	99	99
0	0	0	0	2	1	0
0	0	0	0	0	3	0
1	0	1	0	1	1	1
0	0	2	2	2	2	0
1	1	1	2	2	2	2
0	0	0	0	0	1	0
0	0	1	0	0	2	0
0	0	0	0	0	4	4
1	2	0	0	2	4	3
2	0	1	2	2	3	2
0	0	0	0	0	99	99
2	0	1	2	2	4	4
1	0	1	1	2	2	2
0	0	0	0	2	4	4
0	0	1	2	0	0	0
2	0	2	2	2	0	0
0	0	0	0	2	0	0
0	0	0	0	0	0	0
2	0	2	2	1	1	0
1	0	1	0	1	0	0
0	0	0	0	0	0	0
0	0	0	1	0	1	1
0	0	1	2	1	2	3
2	2	2	2	2	4	3
1	1	2	2	2	1	0
0	0	0	0	0	0	0
0	0	2	1	2	4	2
1	2	2	2	2	0	0
1	0	1	0	1	2	1
0	0	0	0	0	0	0
0	0	0	0	0	0	0
1	1	1	1	1	0	0
0	0	1	0	0	0	0

0	0	0	0	0	0	0
0	0	1	1	0	2	0
0	0	1	0	2	1	0
0	0	1	1	2	4	3
1	0	1	1	2	0	0
2	0	1	2	1	2	2
0	0	0	0	0	0	0
0	0	0	1	1	0	0
0	0	2	0	0	2	2
0	0	1	1	0	2	1
0	0	1	0	2	0	0
0	0	0	0	0	0	1
0	0	1	0	99	2	4
0	0	0	2	0	1	4
0	0	2	1	99	0	1
0	0	1	0	99	2	2
0	0	1	0	99	1	0
1	0	1	0	99	1	0
0	1	1	1	99	4	4
0	0	1	0	99	2	0
0	0	1	1	99	3	0
0	1	1	1	99	3	0
0	0	0	0	0	2	2
0	0	0	0	0	0	0
0	0	1	1	99	4	1
0	0	1	0	99	0	0
99	99	99	99	99	99	99
0	1	2	0	99	0	0
1	1	1	1	99	2	1
0	0	0	1	0	0	0
99	99	99	99	99	99	99
0	0	0	0	99	0	0
0	0	0	1	0	0	0
0	0	0	0	0	2	1
0	0	0	0	0	0	0
0	0	0	0	0	0	0
0	0	0	0	0	0	0
0	0	2	0	99	4	4
0	0	0	0	99	1	0
0	0	0	0	0	2	0
0	0	0	0	0	0	0
0	99	99	0	99	0	1
0	0	0	0	0	1	1
0	0	1	0	99	2	2
0	1	0	0	99	2	0
1	0	1	1	99	1	0
0	0	0	0	99	0	0
0	0	0	0	0	0	0

0	0	0	0	0	1	0
0	2	2	2	99	0	1
0	0	1	0	99	0	0
0	0	0	1	99	1	1
0	0	0	0	1	3	0
0	0	0	0	0	0	0
1	0	1	1	99	0	0
1	0	1	1	99	3	1
0	0	0	1	1	2	1
0	1	1	1	99	0	0
0	0	0	0	99	1	0
0	0	0	1	99	2	3
2	0	2	2	99	0	0
0	99	99	1	99	1	2
0	0	0	0	0	0	0
1	0	0	2	99	2	2
99	99	99	99	99	4	4
0	0	0	0	99	0	2
1	0	1	1	99	1	0
0	2	2	2	99	2	2
0	0	0	0	99	4	4
0	0	1	0	99	0	0
0	0	0	0	99	0	0
0	0	0	0	0	0	0
0	0	1	2	99	1	0
0	0	0	0	0	0	0
0	0	0	2	0	4	4
0	0	0	0	99	0	0
0	0	0	0	99	0	0
2	0	2	0	99	4	1
0	0	2	0	99	2	2
0	0	0	0	99	0	0
0	0	1	0	99	0	0
1	1	2	2	99	0	0
1	0	1	1	99	0	0
0	0	1	0	99	0	0
0	0	0	0	99	0	0
2	0	2	0	99	0	0
0	0	0	1	99	0	0
0	0	1	1	99	0	0
2	1	2	2	99	3	4
0	0	0	0	99	0	0
1	1	1	0	99	0	0
2	0	1	1	99	2	2
0	0	0	0	99	0	0
0	0	1	0	99	0	0
1	1	0	0	99	1	0
0	0	1	0	99	2	1

0	0	0	0	0	1	0
0	0	0	0	99	0	0
0	0	0	0	99	0	0
0	0	2	2	99	0	0
0	0	1	0	99	0	0
0	0	1	0	99	1	0
0	0	0	0	99	1	0
0	1	1	1	99	2	1
0	0	2	2	99	2	2
0	0	2	0	99	0	0
0	2	2	2	99	0	0
1	2	2	2	99	2	3
0	1	1	0	99	3	1
0	0	1	0	99	0	0
0	0	0	1	99	1	1
99	99	99	99	99	99	99
0	0	0	0	99	2	2
0	1	2	2	99	4	4
2	0	0	1	99	2	1
0	0	1	0	99	2	0
0	0	1	1	99	2	0
1	1	2	2	99	0	0
0	0	0	0	99	0	0
0	0	1	0	99	0	0
0	0	1	0	99	2	4
0	0	0	0	0	99	99
0	0	0	0	1	2	0
0	0	0	0	0	2	1
2	0	1	1	2	4	2
0	0	0	0	0	0	1
0	0	0	0	1	0	0
0	0	0	0	1	1	2
0	0	0	0	0	1	1
99	99	99	99	99	99	99
0	1	0	0	0	1	0
99	0	1	0	2	1	0
0	0	1	0	0	2	1
0	0	0	0	0	1	1
0	0	1	1	2	1	0
0	0	0	0	0	4	0
0	1	1	0	1	3	2
0	1	2	2	2	3	1
2	2	2	1	2	4	4
0	0	2	1	2	1	0
99	99	99	99	99	0	0
0	0	0	0	0	4	4
99	99	0	0	0	1	0
0	0	2	0	2	3	1

0	0	0	0	0	2	2
0	0	0	1	0	1	1
0	0	0	0	2	3	3
1	99	1	2	0	4	4
0	0	0	0	0	1	1
0	0	0	0	0	4	0
0	0	0	1	1	0	0
1	0	0	0	2	3	1
0	0	0	0	2	2	1
0	0	1	0	2	4	4
0	0	1	1	2	1	0
1	0	1	1	0	3	2
0	0	0	0	1	4	2
1	0	2	2	2	4	4
99	99	2	1	0	2	4
0	0	1	0	0	4	3
0	0	1	0	0	1	1
99	0	0	0	0	2	0
0	0	0	0	0	2	0
99	99	99	99	99	0	0
1	0	2	2	2	4	4
0	0	1	0	1	0	0
0	0	1	0	2	0	4
0	0	1	0	2	2	2
0	0	0	1	1	0	0
2	1	2	1	2	2	3
0	1	0	1	1	2	2
0	0	1	0	2	0	0
0	0	0	0	0	0	0
0	0	2	1	2	4	2
0	0	0	0	1	0	0
0	0	1	1	1	2	1
0	0	0	0	0	2	1
2	1	2	2	2	0	1
0	0	0	0	2	0	1
0	0	0	0	1	2	1
0	0	0	0	0	0	0
0	0	0	0	1	4	4
0	0	0	0	0	0	0
0	0	1	0	2	4	4
0	0	0	0	1	4	1
1	1	1	2	0	2	2
0	0	1	0	2	0	0
0	0	0	0	1	2	1
0	0	1	0	0	0	0
0	0	1	0	1	1	0
0	0	1	0	1	1	1
0	0	1	0	1	3	3

0	0	2	1	0	0	0
0	0	0	0	0	1	0
0	0	1	0	1	1	1
0	0	1	0	0	0	0
2	0	2	2	2	2	3
1	0	1	0	2	2	2
0	0	0	1	2	0	0
0	0	0	0	1	4	0
0	0	0	0	0	0	0
0	0	2	0	2	4	1
0	0	1	1	1	1	0
2	2	2	2	2	0	0
99	99	99	99	99	99	99
0	0	0	0	0	2	0
0	0	0	0	0	0	0
0	0	2	1	2	2	1
1	0	2	2	2	2	2
0	0	1	0	0	1	0
0	0	1	0	2	2	2
1	1	2	2	2	0	0
0	0	0	0	1	2	1
0	0	0	0	1	1	1
0	0	0	0	2	1	1
1	0	2	2	2	2	0
1	0	0	2	0	2	3
0	0	1	0	1	2	0
2	2	2	2	2	3	0
0	1	1	1	1	1	2
0	0	0	0	2	0	0
0	0	0	0	0	1	1
0	0	2	2	1	4	4
0	0	0	0	2	2	3
0	0	1	0	0	1	0
1	1	2	1	2	2	0
0	0	1	0	1	1	0
0	0	0	0	0	0	0
0	0	2	0	2	1	0
0	0	0	0	1	1	0
0	0	2	0	2	2	0
0	0	0	0	0	0	0
0	0	2	1	2	2	1
0	0	1	0	0	0	0
0	0	0	0	1	2	1
0	0	1	0	0	0	0
0	0	0	0	0	1	0
0	0	0	0	0	1	1
0	0	1	1	1	0	0
0	0	0	0	1	4	1

0	0	1	0	0	1	0
2	1	1	2	2	2	3
0	1	0	0	0	1	1
0	0	1	0	0	1	0
0	0	1	1	0	0	1
0	0	0	0	0	2	1
0	0	0	0	0	2	1
0	0	1	0	0	0	0
0	0	0	0	1	0	0
0	0	0	0	0	1	0
0	0	1	1	1	2	2
0	0	1	1	1	3	2
0	0	1	1	2	2	1
0	0	0	0	0	0	0
0	0	0	0	0	0	0
0	0	1	0	2	2	4
0	0	1	1	1	1	0
0	0	1	0	2	4	1
0	0	2	1	1	0	0
99	99	0	0	0	4	4
0	0	0	1	2	2	1
0	0	2	0	0	4	4
0	0	0	0	1	4	1
0	1	1	1	1	0	0
0	0	1	0	1	4	0
0	0	1	0	2	2	2
0	0	0	0	0	2	1
1	0	1	0	2	0	0
0	0	1	1	1	4	2
0	1	2	1	1	1	1
0	0	2	2	1	2	2
0	0	0	0	1	4	4
0	0	0	0	0	0	0
0	1	2	2	2	2	2
0	0	1	1	1	1	0
1	0	1	1	1	1	1
0	0	1	1	0	0	0
1	1	1	1	1	2	4
1	0	1	0	1	2	1
1	0	0	1	2	0	2
0	0	0	0	0	0	0
0	0	0	0	2	2	0
0	0	0	0	2	1	0
0	0	1	0	2	2	2
0	0	0	0	1	1	3
0	0	2	0	0	0	0
1	1	2	2	0	2	1
2	2	2	2	2	2	0

0	0	1	0	2	2	1
0	0	0	0	1	0	2
0	0	1	0	0	2	4
0	1	0	0	0	2	0
0	0	0	0	0	4	0
1	2	2	2	2	1	0
0	0	0	0	0	0	0
0	0	1	1	1	3	3
0	0	1	0	1	2	2
0	0	0	0	0	2	1
0	0	1	0	1	2	0
1	1	1	1	1	3	2
0	1	2	2	2	0	0
0	0	1	1	0	2	2
2	1	2	1	1	3	2
1	0	1	1	1	3	4
0	0	1	0	1	1	0
0	1	1	0	0	0	0
1	0	2	2	2	4	4
1	0	1	1	2	4	1
1	1	1	1	1	0	0
0	0	0	0	0	0	0
0	0	0	0	1	0	0
0	0	0	0	0	0	0
0	1	1	1	1	2	99
1	1	2	1	0	0	0
0	0	0	0	1	0	0
2	2	2	2	1	2	2
0	1	1	1	1	0	0
0	0	2	2	2	0	0
1	1	2	1	2	0	2
0	0	0	0	2	1	0
0	1	1	0	0	1	0
0	1	1	0	1	0	0
0	0	1	1	1	4	4
0	0	1	1	0	2	0
0	0	0	0	0	0	0
0	0	1	1	1	1	0
0	0	1	2	2	2	2
0	0	1	0	0	0	0
2	2	2	2	2	1	1
1	1	2	2	2	4	4
99	99	99	0	1	1	4
1	1	2	1	2	2	1
0	0	1	0	2	2	0
0	2	2	1	2	3	3
0	0	1	0	0	1	0
1	0	2	2	1	0	0

0	0	1	1	0	4	1
0	0	0	0	0	0	0
0	0	1	1	1	0	0
0	0	0	0	0	1	1
99	99	99	99	99	99	99
1	1	2	1	2	0	4
0	99	2	2	0	2	3
0	0	0	0	0	1	0
99	0	1	0	0	2	1
0	0	0	0	0	0	0
1	0	1	1	1	4	1
0	0	0	0	1	1	0
1	0	0	0	2	4	0
0	0	0	0	0	0	0
0	0	0	0	0	0	99
2	2	2	2	0	4	4
99	0	2	0	0	1	2
0	1	1	0	0	2	4
2	0	1	1	2	0	0
0	0	1	1	1	2	4
0	0	1	0	0	3	2
0	0	0	0	1	1	1
0	0	1	1	1	2	1
1	0	1	1	1	1	1
0	0	1	0	0	4	3
0	0	1	1	1	1	0
0	0	1	1	1	0	0
0	0	1	0	1	2	1
0	0	0	0	2	4	2
0	0	1	0	0	2	1
0	1	0	0	1	0	0
0	0	0	2	0	1	0
0	0	0	0	2	0	1
0	0	0	0	0	0	4
99	0	0	0	1	0	3
0	0	1	0	0	1	0
0	0	0	0	0	3	1
0	0	2	1	0	2	2
0	0	1	0	0	2	0
0	1	0	0	0	0	0
0	0	1	1	0	1	1
0	0	0	0	0	0	0
0	0	0	1	1	2	0
0	0	1	1	1	1	2
0	0	1	0	0	0	1
0	0	0	0	0	2	99
0	0	1	0	1	2	1
0	0	1	0	0	0	1

1	0	1	2	2	1	0
0	0	0	0	2	4	4
0	0	0	0	0	0	1
0	0	2	1	1	2	3
0	1	1	1	0	3	2
0	0	0	0	0	2	2
0	0	1	1	0	2	0
0	0	1	1	1	3	2
0	0	0	0	0	0	1
0	0	1	0	1	0	0
0	0	0	0	0	2	2
0	0	0	1	0	2	2
0	0	1	2	2	2	1
2	0	2	2	2	3	4
0	0	0	0	0	1	0
0	0	1	0	1	1	0
0	0	1	0	1	0	0
99	0	0	1	99	4	1
0	0	1	0	0	0	0
0	0	1	0	0	1	0
0	0	0	1	0	0	0
0	1	2	2	2	1	4
99	0	1	1	1	1	1
2	2	2	2	2	4	4
0	0	0	0	2	0	0
0	0	0	0	0	1	99
0	0	0	0	0	0	2
1	0	1	0	0	4	0
1	1	1	2	1	3	4
0	0	1	0	99	0	1
0	0	0	0	0	0	0
0	0	0	0	99	4	2
0	0	0	0	0	0	0
0	0	1	2	99	4	4
0	0	0	0	0	1	0
1	0	1	0	2	2	0
0	0	0	0	0	0	0
1	0	1	1	1	2	3
0	0	0	0	2	0	0
1	0	1	1	1	1	1
0	0	1	0	1	1	0
0	0	1	0	0	2	2
0	0	0	0	2	1	2
0	0	1	0	2	0	0
0	1	2	2	2	2	0
0	0	1	0	0	2	0
0	0	0	0	1	2	3
0	0	1	0	1	0	1

0	1	1	1	1	0	1
0	0	2	0	2	0	2
0	0	1	1	1	0	0
1	0	0	0	1	1	0
99	99	99	99	99	99	99
99	0	1	1	99	0	0
2	2	2	2	2	0	0
0	0	0	0	0	0	0
0	0	1	1	1	0	0
0	0	0	0	1	2	1
0	0	0	1	99	1	0
0	1	1	1	99	3	4
0	0	1	1	99	0	0
2	2	2	2	99	99	99
0	0	1	0	99	0	0
1	2	2	2	99	1	0
0	0	0	0	0	1	1
0	0	0	0	0	4	4
0	0	0	0	1	2	0
0	1	1	1	99	0	0
0	0	0	0	0	0	0
0	0	1	2	99	4	0
0	0	0	1	99	0	0
1	0	2	2	99	0	3
1	0	2	0	99	2	1
2	1	2	2	99	4	4
0	0	0	0	99	0	0
0	0	0	0	0	0	0
0	0	1	0	99	4	4
0	0	1	1	99	0	0
1	1	1	1	99	1	0
0	0	1	1	99	0	0
0	0	2	1	99	4	3
0	0	0	0	99	3	1
0	0	0	0	99	2	2
0	0	0	0	0	4	4
2	1	1	1	99	2	2
1	0	2	2	99	2	2
0	0	0	0	0	2	2
0	0	2	1	99	3	1
1	1	1	1	99	2	2
2	0	1	0	99	1	2
0	0	0	1	99	1	1
1	1	1	1	1	2	1
0	0	2	2	99	0	0
1	1	1	1	99	2	1
1	0	1	0	99	0	0
0	0	0	0	2	0	0

0	0	1	0	1	4	2
0	0	0	0	0	0	0
0	0	0	0	1	0	1
0	0	0	0	2	1	1
0	0	0	0	0	0	0
0	0	1	0	2	3	1
0	0	0	0	0	0	0
0	0	0	0	1	3	2
0	0	1	0	1	0	0
0	1	2	2	2	4	3
0	0	0	2	2	1	0
0	0	1	0	1	3	3
1	1	1	1	1	1	0
0	0	1	0	1	2	1
0	0	0	0	0	1	1
0	0	0	0	0	1	3
0	0	0	0	2	2	0
0	0	0	0	0	3	0
0	0	0	0	2	2	3
0	0	0	0	0	2	0
0	1	2	2	2	2	2
0	0	0	0	0	1	0
0	1	1	0	2	0	0
0	0	1	0	0	2	3
0	0	1	0	0	0	0
0	0	1	0	2	2	4
0	1	2	2	2	0	0
1	1	1	1	1	1	1
0	0	1	0	0	0	0
0	0	1	0	1	4	2
0	0	0	0	1	1	0
2	0	1	2	1	1	2
0	0	2	1	2	4	2
1	1	1	1	1	2	2
0	0	2	0	2	3	0
0	0	0	0	1	2	2
0	0	1	0	2	4	0
1	0	2	2	2	1	2
1	0	2	0	0	2	0
0	1	2	2	2	2	2
1	0	1	0	0	3	1
0	0	2	2	0	4	0
99	99	99	99	99	99	99
1	0	2	1	0	2	99
0	0	1	0	1	2	0
0	0	0	0	0	2	0
0	0	1	0	2	0	0
0	0	1	1	2	2	4

0	0	0	0	1	2	2
0	0	1	0	1	0	0
0	0	0	0	1	2	1
0	0	2	2	2	2	2
1	0	0	0	2	3	4
0	0	1	0	2	1	0
0	0	0	0	1	2	1
2	2	2	2	2	0	0
0	0	2	0	2	0	0
0	0	1	0	2	1	0
0	0	0	0	0	0	0
0	0	1	1	2	2	1
1	0	1	0	0	3	0
0	0	0	0	0	0	0
0	1	1	1	1	1	1
0	1	1	1	1	1	1
0	0	0	0	2	4	2
0	0	0	0	0	0	0
1	0	2	0	2	1	0
1	1	1	0	0	2	1
0	0	1	1	0	0	0
99	99	99	99	1	4	2
0	1	0	0	0	0	0
0	0	2	2	2	4	0
0	0	1	0	1	3	2
0	0	0	0	0	0	0
1	0	0	0	0	2	1
99	99	99	99	99	99	99
0	0	0	0	1	1	0
0	0	1	0	2	0	0
99	99	99	99	99	99	99
2	0	1	1	2	0	0
0	0	1	0	0	1	0
0	1	2	1	1	3	2
0	1	1	1	2	0	0
0	0	1	0	2	2	2
2	0	1	1	1	4	4
0	0	2	1	2	0	0
1	0	1	2	2	99	99
0	0	1	0	0	0	1
1	1	1	1	1	1	0
2	2	2	2	2	0	0
1	0	0	0	1	1	1
2	0	1	1	2	2	2
0	1	1	1	1	1	0
1	0	1	0	2	1	1
0	2	2	2	2	1	0
1	0	0	0	2	1	0

0	0	1	0	1	3	2
0	2	2	2	1	0	0
0	0	0	0	0	4	4
0	0	0	0	0	2	0
2	1	2	2	1	4	4
1	2	0	1	2	4	0
0	0	0	0	0	0	0
1	1	1	1	1	1	1
99	99	0	0	0	0	0
0	0	0	0	0	0	0
0	1	1	0	0	3	2
1	0	2	0	2	2	1
0	1	1	2	2	2	0
1	1	1	0	2	1	0
1	0	1	1	1	1	1
0	0	1	0	0	2	1
0	0	2	2	0	4	4
0	0	1	1	2	1	2
0	0	1	1	0	1	0
0	0	0	0	0	1	0
1	1	2	1	2	2	1
1	0	1	2	0	0	0
1	0	1	1	1	1	0
0	0	1	1	1	1	1
0	0	1	0	0	3	3
1	0	2	2	1	0	0
0	0	1	0	0	0	0
2	0	2	2	0	0	1
0	0	1	0	0	4	4
0	0	0	0	1	1	0
0	0	1	0	1	2	2
0	0	0	0	0	0	0
2	0	1	1	2	4	4
0	0	0	0	2	2	0
1	0	0	0	0	1	0
2	1	2	0	2	4	1
0	0	1	1	1	4	4
0	0	0	0	1	1	1
0	0	1	0	0	1	0
0	0	1	1	1	1	0
2	2	2	2	2	4	4
0	0	1	0	0	0	0
1	0	1	1	2	2	1
0	0	2	2	2	3	0
0	0	0	0	0	2	0
0	0	1	0	0	3	4
0	0	1	0	1	0	0
1	0	0	1	2	0	0

2	0	2	1	2	4	4
0	0	0	0	0	0	1
0	0	0	0	1	1	1
0	1	2	2	2	3	3
0	0	0	0	0	0	0
1	0	1	1	2	0	1
0	0	1	0	0	0	0
2	0	1	0	0	4	4
2	2	2	2	0	0	4
0	0	2	2	1	3	0
1	99	2	2	2	3	2
0	0	0	0	0	0	0
0	0	0	0	1	0	0
0	0	1	1	0	1	0
0	0	1	1	1	3	2
1	2	1	2	2	3	1
0	0	1	0	2	2	1
0	0	0	0	0	1	0
0	0	0	0	1	4	3
1	0	1	1	2	2	1
0	0	0	0	2	0	0
0	0	1	1	2	2	3
2	0	0	0	0	3	99
0	0	0	0	2	1	0
0	0	0	0	0	1	0
0	0	0	0	0	0	0
0	0	0	0	0	0	0
1	0	0	0	1	0	0
0	0	1	0	0	0	0
0	0	0	0	0	1	0
2	0	1	0	2	1	0
0	0	0	0	0	0	0
0	0	1	1	2	4	3
0	0	0	0	0	4	3
0	0	1	2	0	1	0
0	1	0	0	1	0	0
0	0	0	0	2	0	0
1	0	1	0	1	1	3
99	99	99	99	99	0	0
0	0	1	1	1	1	1
0	0	0	0	0	4	4
0	2	2	2	2	0	1
2	2	2	2	2	2	2
2	2	2	2	2	1	0
0	0	0	0	0	0	0
0	2	2	0	2	0	0
0	0	1	0	2	3	3
1	0	2	0	2	1	4

0	0	0	2	2	0	0
1	1	1	1	1	0	0
0	0	0	1	1	0	0
0	0	0	0	2	0	0
1	1	1	1	1	2	0
0	0	0	0	0	0	0
0	0	0	0	0	0	0
1	0	0	0	2	1	0
0	0	2	2	0	3	0
1	1	1	1	1	1	1
1	0	1	1	1	2	3
1	0	1	2	2	4	3
0	0	2	1	2	2	2
0	0	1	1	1	3	3
0	0	2	2	2	2	1
0	0	0	1	1	1	0
2	2	2	2	2	2	2
1	0	1	2	2	2	2
0	0	1	0	0	4	4
0	0	1	0	2	3	3
2	0	1	0	0	0	0
0	0	2	1	2	0	0
0	0	1	1	0	2	2
0	1	1	1	0	1	2
0	1	1	1	0	1	2
0	0	1	1	2	3	4
0	0	1	1	1	3	4
0	0	0	0	2	4	4
0	0	0	0	#NULL!	0	4
0	0	0	0	#NULL!	4	0
0	0	1	0	#NULL!	1	2
0	0	0	0	#NULL!	1	1
1	1	1	1	#NULL!	2	4
0	0	0	0	#NULL!	1	1
0	0	0	0	#NULL!	1	0
99	1	2	2	#NULL!	0	0
0	0	0	0	#NULL!	0	0
0	0	0	0	#NULL!	0	1
0	0	0	0	#NULL!	1	2
0	0	0	0	#NULL!	1	0
0	0	1	1	#NULL!	1	0
0	0	1	0	#NULL!	0	0
0	0	0	0	#NULL!	0	0
0	0	0	0	#NULL!	0	0
0	0	1	0	#NULL!	2	0
0	0	0	0	#NULL!	0	0
0	0	1	0	#NULL!	0	1
1	0	2	2	#NULL!	1	0

0	0	1	0	#NULL!	0	0
0	0	0	0	#NULL!	2	1
0	0	0	0	#NULL!	0	0
0	0	0	0	#NULL!	0	0
0	1	1	99	#NULL!	1	1
0	0	0	0	#NULL!	1	1
2	1	2	2	#NULL!	2	0
0	0	1	1	#NULL!	1	0
0	0	0	0	#NULL!	1	0
0	0	2	0	#NULL!	1	2
0	0	0	0	#NULL!	4	4
0	0	0	0	#NULL!	0	0
0	0	99	0	#NULL!	0	0
0	0	0	0	#NULL!	0	0
0	0	0	0	#NULL!	1	0
1	0	1	0	#NULL!	1	0
0	0	1	1	#NULL!	2	1
1	0	0	2	#NULL!	2	1
1	0	1	1	#NULL!	3	1
0	0	2	2	#NULL!	0	1
0	0	0	0	#NULL!	0	0
0	0	0	0	#NULL!	0	1
0	0	1	1	#NULL!	2	3
0	99	99	1	#NULL!	2	1
0	0	0	0	#NULL!	0	0
2	0	2	2	#NULL!	1	0
0	0	0	0	#NULL!	2	0
0	0	0	0	#NULL!	0	1
0	0	0	0	#NULL!	0	0
0	0	0	0	#NULL!	0	0
0	0	0	0	#NULL!	0	0
1	1	1	1	#NULL!	0	0
0	0	1	1	#NULL!	1	2
0	0	0	1	#NULL!	1	0
0	0	1	0	#NULL!	1	0
0	0	0	0	#NULL!	0	0
0	0	0	0	#NULL!	0	0
2	2	2	2	#NULL!	0	0
0	1	0	0	#NULL!	4	99
0	0	0	0	#NULL!	2	0
0	0	0	0	#NULL!	0	0
0	1	0	1	#NULL!	0	0
0	0	1	1	#NULL!	2	3
1	0	2	1	#NULL!	1	0
0	0	2	1	#NULL!	2	1
0	0	2	0	#NULL!	0	2
0	0	0	0	#NULL!	0	0
2	1	1	2	#NULL!	1	0

0	0	0	0	#NULL!	0	0
0	0	0	0	#NULL!	0	3
0	0	0	0	#NULL!	99	99
0	0	0	0	#NULL!	0	0
0	0	0	0	#NULL!	1	1
0	0	0	1	#NULL!	1	0
0	0	0	0	#NULL!	0	0
0	0	0	0	#NULL!	1	0
1	1	1	1	#NULL!	0	0
0	0	1	0	#NULL!	2	1
1	0	2	0	#NULL!	0	1
1	0	2	2	#NULL!	4	1
0	0	0	0	#NULL!	0	0
1	0	1	1	#NULL!	1	0
0	1	1	1	#NULL!	0	0
1	0	1	1	#NULL!	0	0
1	1	1	1	#NULL!	0	0
0	0	0	0	#NULL!	2	1
0	0	0	0	#NULL!	0	0
1	0	0	99	#NULL!	2	1
0	0	0	0	#NULL!	1	0
0	0	0	0	#NULL!	0	0
0	0	0	0	#NULL!	0	0
1	1	1	1	#NULL!	2	1
0	0	0	0	#NULL!	0	0
0	0	0	0	#NULL!	0	0
0	0	0	0	#NULL!	0	4
0	0	0	0	#NULL!	0	0
0	0	0	0	#NULL!	0	0
0	0	1	1	#NULL!	0	0
2	0	0	2	#NULL!	2	0
0	0	0	0	#NULL!	0	0
0	1	1	1	#NULL!	2	1
0	1	0	1	#NULL!	0	0
2	2	2	2	#NULL!	2	3
1	2	2	2	#NULL!	4	2
0	0	1	1	#NULL!	2	1
0	0	0	0	#NULL!	0	1
0	0	1	0	#NULL!	1	0
1	1	1	1	#NULL!	2	3
0	0	1	0	#NULL!	2	0
0	0	0	0	#NULL!	0	0
1	0	1	1	#NULL!	4	3
0	0	0	0	#NULL!	0	0
0	0	0	0	#NULL!	0	0
1	1	1	1	#NULL!	0	0
0	1	1	1	#NULL!	2	1
0	0	1	0	#NULL!	2	2

0	0	0	0	#NULL!	0	0
0	0	0	0	#NULL!	1	0
0	0	0	0	#NULL!	0	0
0	0	1	0	#NULL!	3	3
0	0	0	0	#NULL!	0	0
0	0	0	0	#NULL!	1	1
0	0	0	0	#NULL!	2	3
0	0	1	0	#NULL!	3	2
1	0	0	0	#NULL!	4	1
1	0	0	0	#NULL!	1	0
0	0	0	0	#NULL!	4	2
0	0	0	0	#NULL!	1	0
0	0	1	0	#NULL!	1	4
1	1	2	2	#NULL!	2	3
0	0	0	0	#NULL!	1	1
0	0	0	0	#NULL!	2	1
0	0	1	1	#NULL!	3	3
0	0	0	0	#NULL!	0	0
1	1	2	2	#NULL!	3	0
0	0	0	0	#NULL!	4	2
0	0	1	0	#NULL!	2	2
0	0	0	0	#NULL!	2	2
0	0	1	0	#NULL!	1	0
0	0	0	1	#NULL!	4	1
0	0	0	0	#NULL!	1	0
0	0	1	0	#NULL!	1	1
0	0	0	1	#NULL!	4	2
0	0	0	0	#NULL!	1	0
0	0	0	0	#NULL!	2	1
0	0	1	1	#NULL!	1	1
0	0	1	0	#NULL!	1	1
0	0	0	0	#NULL!	1	0
0	0	0	0	#NULL!	4	2
0	0	1	0	#NULL!	0	0
0	0	0	0	#NULL!	1	0
0	0	0	0	#NULL!	1	1
0	0	0	0	#NULL!	1	4
0	0	0	0	#NULL!	1	0
0	0	0	0	#NULL!	1	0
0	0	0	0	#NULL!	0	0
0	1	0	0	#NULL!	4	0
0	0	1	0	#NULL!	0	1
0	0	1	0	#NULL!	0	0
1	0	2	2	#NULL!	2	0
0	0	2	0	#NULL!	0	1
0	0	0	0	#NULL!	3	3
0	0	0	0	#NULL!	0	0
1	2	2	0	#NULL!	2	1

0	0	1	0	#NULL!	1	0
1	0	0	2	#NULL!	1	0
1	1	1	1	#NULL!	1	1
0	1	1	1	#NULL!	4	1
0	0	0	0	#NULL!	0	0
0	1	2	2	#NULL!	0	0
0	0	0	1	#NULL!	1	0
1	0	0	0	#NULL!	1	1
0	0	0	0	#NULL!	0	0
0	1	1	1	#NULL!	2	99
1	0	0	0	#NULL!	2	3
0	0	0	0	#NULL!	0	0
0	0	1	1	#NULL!	1	1
1	1	2	1	#NULL!	2	1
0	0	0	0	#NULL!	0	0
2	2	2	2	#NULL!	0	1
0	0	1	0	#NULL!	1	0
0	0	2	0	#NULL!	2	0
1	0	0	1	#NULL!	4	2
1	0	1	0	#NULL!	0	0
0	2	2	2	#NULL!	4	3
0	1	1	1	#NULL!	0	0
0	0	0	0	#NULL!	0	0
0	0	1	0	#NULL!	2	1
1	0	1	1	#NULL!	2	3
99	99	99	99	#NULL!	0	0
1	2	2	2	#NULL!	3	4
0	0	0	0	#NULL!	2	2
0	0	1	2	#NULL!	4	2
0	0	0	0	#NULL!	0	1
1	0	2	2	#NULL!	2	1
0	0	0	0	#NULL!	0	0
0	0	0	0	#NULL!	2	0
1	0	0	1	#NULL!	2	0
0	0	2	2	#NULL!	0	0
0	0	1	0	#NULL!	0	0
0	1	1	1	#NULL!	2	0
0	2	2	2	#NULL!	2	2
0	0	0	0	#NULL!	0	0
0	0	1	1	#NULL!	1	1
0	0	2	0	#NULL!	4	2
1	1	1	1	#NULL!	0	0
0	0	1	1	#NULL!	1	1
2	0	2	2	#NULL!	0	2
0	0	0	1	#NULL!	0	0
1	1	1	1	#NULL!	0	0
0	0	2	1	#NULL!	1	1
0	0	1	1	#NULL!	4	1

0	0	1	0	#NULL!	1	0
0	0	1	1	#NULL!	2	1
0	0	1	0	#NULL!	2	0
0	1	2	2	#NULL!	0	0
0	0	1	0	#NULL!	0	0
0	0	0	0	#NULL!	0	0
0	0	2	2	#NULL!	2	1
1	0	1	1	#NULL!	0	0
1	0	1	2	#NULL!	4	2
0	0	1	1	#NULL!	1	1
0	0	2	2	#NULL!	0	0
0	1	0	1	#NULL!	0	1
0	0	0	0	#NULL!	0	0
1	2	2	2	#NULL!	0	0
0	0	2	2	#NULL!	2	0
0	0	1	1	#NULL!	2	3
0	0	0	0	#NULL!	0	0
0	0	0	0	#NULL!	0	0
0	0	2	0	#NULL!	4	4
1	0	1	1	#NULL!	2	3
0	0	0	0	#NULL!	1	0
0	0	0	1	#NULL!	2	1
0	0	0	0	#NULL!	2	3
1	0	2	2	#NULL!	3	3
0	0	0	0	#NULL!	2	1
0	0	2	0	#NULL!	3	1
0	0	1	2	#NULL!	2	0
0	1	2	2	#NULL!	2	1
0	0	0	0	#NULL!	0	1
0	0	0	0	#NULL!	0	1
0	0	1	0	#NULL!	0	0
0	0	1	1	#NULL!	2	1
0	1	1	1	#NULL!	4	0
0	0	1	0	#NULL!	2	0
1	0	2	2	#NULL!	3	1
0	0	1	2	#NULL!	3	2
0	0	1	0	#NULL!	2	1
0	0	0	0	#NULL!	0	0
0	0	2	2	#NULL!	3	4
0	0	0	1	#NULL!	3	2
1	1	1	1	#NULL!	1	0
0	0	0	0	#NULL!	1	0
0	0	1	2	#NULL!	0	0
0	0	0	0	#NULL!	2	2
0	0	0	0	#NULL!	1	0
0	0	0	0	#NULL!	1	0
0	0	1	0	#NULL!	2	4
0	0	0	0	#NULL!	4	0

1	1	1	1	#NULL!	1	0
0	0	2	1	#NULL!	1	0
0	0	0	2	#NULL!	0	0
99	99	99	99	#NULL!	99	99
1	0	1	1	#NULL!	0	1
0	0	1	0	#NULL!	2	1
0	0	1	0	#NULL!	1	0
0	0	1	0	#NULL!	1	3
0	0	0	0	#NULL!	0	0
0	0	0	0	#NULL!	2	2
0	0	0	0	#NULL!	0	0
0	0	0	0	#NULL!	1	1
2	1	2	2	#NULL!	99	99
0	0	0	0	#NULL!	1	2
0	1	2	2	#NULL!	2	4
0	0	2	2	#NULL!	1	0
0	0	1	0	#NULL!	0	0
1	0	2	0	#NULL!	3	2
0	0	2	2	#NULL!	1	0
1	0	1	0	#NULL!	1	1
0	0	2	0	#NULL!	0	0
0	0	1	0	#NULL!	2	1
0	0	2	2	#NULL!	1	3
1	0	0	0	#NULL!	1	0
0	0	1	1	#NULL!	0	0
0	0	0	0	#NULL!	3	1
1	0	1	2	#NULL!	4	4
0	0	1	0	#NULL!	0	0
2	0	0	2	#NULL!	3	2
0	0	1	0	#NULL!	1	0
0	0	0	0	#NULL!	0	0
0	2	2	0	#NULL!	0	0
1	0	1	1	#NULL!	2	4
0	0	0	0	#NULL!	0	0
1	2	2	2	#NULL!	0	1
0	0	0	0	#NULL!	4	1
0	0	0	0	#NULL!	0	0
0	1	1	0	#NULL!	2	1
0	0	1	0	#NULL!	1	1
0	0	0	0	#NULL!	1	1
0	0	0	0	#NULL!	2	3
0	0	1	0	#NULL!	0	0
0	0	2	0	#NULL!	0	0
0	0	0	0	#NULL!	0	0
0	0	1	0	#NULL!	1	0
1	99	2	2	#NULL!	0	2
0	0	1	1	#NULL!	1	0
0	0	0	0	#NULL!	2	2

0	0	0	0	#NULL!	99	99
0	0	0	0	#NULL!	0	0
1	0	1	0	#NULL!	0	0
1	1	1	1	#NULL!	2	2
0	0	1	0	#NULL!	3	3
0	0	0	0	#NULL!	0	0
0	0	2	1	#NULL!	0	0
1	0	0	0	#NULL!	0	0
0	0	1	1	#NULL!	0	0
0	1	1	1	#NULL!	2	2
0	1	1	1	#NULL!	3	3
2	2	2	2	#NULL!	0	1
0	0	2	0	#NULL!	2	0
0	0	1	1	#NULL!	3	3
2	0	0	0	#NULL!	2	4
0	0	1	0	#NULL!	1	0
99	99	99	99	#NULL!	99	99
0	0	0	0	#NULL!	1	0
0	0	0	1	#NULL!	0	0
0	1	2	1	#NULL!	2	2
0	1	2	2	#NULL!	0	0
0	0	0	0	#NULL!	1	1
0	1	1	0	#NULL!	2	4
0	1	1	1	#NULL!	0	0
1	0	2	1	#NULL!	0	0
0	0	0	0	#NULL!	1	0
99	99	1	99	#NULL!	99	99
0	0	0	0	#NULL!	0	0
0	0	0	1	#NULL!	4	3
0	0	0	0	#NULL!	0	0
0	0	1	0	#NULL!	2	0
99	0	0	0	#NULL!	3	0
0	0	1	1	#NULL!	99	99
2	0	2	0	#NULL!	3	2
0	0	1	0	#NULL!	1	0
0	0	2	1	#NULL!	3	2
0	0	0	0	#NULL!	0	0
0	0	1	1	#NULL!	2	0
0	0	1	0	#NULL!	1	0
0	0	0	0	#NULL!	2	0
0	1	1	1	#NULL!	3	3
0	0	0	0	#NULL!	1	0
0	99	99	99	#NULL!	1	0
0	0	0	0	#NULL!	1	0
0	0	0	0	#NULL!	0	0
0	0	0	0	#NULL!	1	0
0	0	1	0	#NULL!	1	1
0	0	0	0	#NULL!	3	1

0	0	2	0	#NULL!	4	1
1	0	1	1	#NULL!	4	1
0	0	0	0	#NULL!	0	0
0	0	0	0	#NULL!	0	0
1	0	1	1	#NULL!	2	2
0	0	0	0	#NULL!	2	0
0	0	0	0	#NULL!	0	0
0	0	0	0	#NULL!	1	1
0	0	0	0	#NULL!	1	0
0	0	1	2	#NULL!	4	4
1	1	2	2	#NULL!	1	2
1	1	2	2	#NULL!	1	99
0	0	0	0	#NULL!	3	1
0	0	0	0	#NULL!	1	1
0	0	1	0	#NULL!	0	0
0	2	2	2	#NULL!	1	0
0	0	0	1	#NULL!	0	1
1	0	1	0	#NULL!	0	4
0	0	0	0	#NULL!	2	1
0	0	0	0	#NULL!	2	0
0	0	1	0	#NULL!	1	0
0	0	0	0	#NULL!	0	0
0	0	1	0	#NULL!	0	0
99	99	99	99	#NULL!	99	99
0	0	0	0	#NULL!	0	1
0	0	0	0	#NULL!	3	1
0	0	2	0	#NULL!	2	4
0	1	1	1	#NULL!	2	2
0	0	0	0	#NULL!	0	0
0	0	1	0	#NULL!	0	0
0	1	99	1	#NULL!	4	2
99	99	99	99	#NULL!	99	99
0	0	0	0	#NULL!	0	0
0	0	1	2	#NULL!	2	2
0	0	1	0	#NULL!	0	0
1	0	2	2	#NULL!	2	1
0	0	0	0	#NULL!	0	0
0	0	1	0	#NULL!	0	0
0	0	0	0	#NULL!	0	0
0	1	2	2	#NULL!	1	0
0	0	0	1	#NULL!	0	0
1	0	1	0	#NULL!	4	1
0	0	0	0	#NULL!	0	0
1	0	1	1	#NULL!	3	4
1	0	1	1	#NULL!	0	1
0	0	0	0	#NULL!	2	2
0	0	0	1	#NULL!	3	2
1	1	2	2	#NULL!	1	0

0	1	1	0	#NULL!	0	0
1	0	1	1	#NULL!	2	1
1	1	1	1	#NULL!	0	0
1	0	0	1	#NULL!	1	0
1	0	2	2	#NULL!	2	0
0	0	1	0	#NULL!	0	0
0	0	0	0	#NULL!	0	0
0	0	0	0	#NULL!	4	4
0	0	1	1	#NULL!	4	2
0	0	0	0	#NULL!	2	2
0	0	2	1	#NULL!	0	0
0	0	0	0	#NULL!	0	0
0	0	1	0	#NULL!	0	0
0	1	2	1	#NULL!	4	3
0	0	2	2	#NULL!	1	0
0	1	2	2	#NULL!	1	1
1	1	1	1	#NULL!	4	3
1	1	2	2	#NULL!	2	1
0	0	1	0	#NULL!	4	2
0	0	2	1	#NULL!	2	3
0	0	0	0	#NULL!	0	0
0	0	1	1	#NULL!	2	2
0	0	2	2	#NULL!	0	0
1	1	1	1	#NULL!	0	0
0	0	0	1	#NULL!	2	2
0	0	0	0	#NULL!	0	0
0	1	0	0	#NULL!	0	0
0	0	0	0	#NULL!	1	1
0	0	0	1	#NULL!	1	2
99	0	1	1	#NULL!	1	0
0	1	2	1	#NULL!	1	2
0	1	2	1	#NULL!	4	2
1	0	1	1	#NULL!	1	1
2	0	1	2	#NULL!	0	1
1	0	2	2	#NULL!	1	2
0	0	0	0	#NULL!	2	1
0	0	0	0	#NULL!	0	0
0	0	1	0	#NULL!	1	3
0	0	0	0	#NULL!	1	0
0	0	0	0	#NULL!	0	0
0	0	2	1	#NULL!	3	1
0	0	1	0	#NULL!	2	0
0	0	1	2	#NULL!	1	0
0	0	0	0	#NULL!	0	0
0	0	1	0	#NULL!	0	0
0	0	0	0	#NULL!	0	0
0	0	0	0	#NULL!	0	0
0	0	0	0	#NULL!	1	0

99	0	0	0	#NULL!	0	0
0	0	0	1	#NULL!	4	4
1	1	2	2	#NULL!	2	2
2	2	2	2	#NULL!	1	0
0	0	1	1	#NULL!	2	1
0	0	0	0	#NULL!	4	2
0	0	0	0	#NULL!	1	1
0	1	2	0	#NULL!	0	0
0	0	0	0	#NULL!	1	1
0	0	0	0	#NULL!	1	1
0	0	0	1	#NULL!	1	0
0	1	1	0	#NULL!	2	2
2	1	2	2	#NULL!	4	4
0	0	0	0	#NULL!	0	1
99	99	99	99	#NULL!	0	0
0	0	1	0	#NULL!	4	0
0	0	2	1	#NULL!	0	0
0	0	0	0	#NULL!	4	0
0	0	0	0	#NULL!	0	0
99	99	99	99	#NULL!	99	99
0	0	1	1	#NULL!	0	0
0	0	1	0	#NULL!	0	1
0	0	0	0	#NULL!	2	4
1	0	2	2	#NULL!	3	2
0	0	1	1	#NULL!	1	0
0	0	0	0	#NULL!	2	1
0	1	1	1	#NULL!	1	0
0	0	0	0	#NULL!	2	2
0	0	0	0	#NULL!	0	0
0	0	0	0	#NULL!	2	2
2	2	2	2	#NULL!	0	0
0	0	0	0	#NULL!	0	0
2	0	1	1	#NULL!	3	3
1	1	1	1	#NULL!	2	0
2	2	2	2	#NULL!	0	0
0	0	1	0	#NULL!	4	4
0	0	1	0	#NULL!	1	3
0	0	1	1	#NULL!	2	1
0	0	1	2	#NULL!	0	1
0	0	0	0	#NULL!	2	0
0	0	0	0	#NULL!	0	0
99	1	0	0	#NULL!	1	0
0	0	0	0	#NULL!	1	0
0	0	2	2	#NULL!	0	0
0	0	2	2	#NULL!	0	0
0	0	1	0	#NULL!	4	4
0	0	1	0	#NULL!	4	0
1	2	2	2	#NULL!	3	4

1	0	2	1	#NULL!	4	4
0	0	0	0	#NULL!	0	0
0	0	0	0	#NULL!	1	0
0	0	0	1	#NULL!	2	2
0	0	1	1	#NULL!	0	1
0	0	1	0	#NULL!	4	0
0	0	0	0	#NULL!	1	0
0	0	0	0	#NULL!	3	4
0	0	0	0	#NULL!	0	0
0	0	2	2	#NULL!	2	0
99	0	2	2	#NULL!	2	1
0	2	2	2	#NULL!	1	1
0	1	0	0	#NULL!	1	0
0	0	1	0	#NULL!	3	4
0	0	2	2	#NULL!	1	2
0	0	1	0	#NULL!	2	2
0	0	1	0	#NULL!	0	0
0	0	0	0	#NULL!	2	1
0	0	1	1	#NULL!	0	0
0	0	2	1	#NULL!	0	0
0	0	0	0	#NULL!	1	2
0	0	0	1	#NULL!	1	0
0	0	0	0	#NULL!	3	0
0	0	2	2	#NULL!	2	3
0	0	1	1	#NULL!	0	0
0	0	0	0	#NULL!	0	0
0	0	1	1	#NULL!	0	0
0	0	0	0	#NULL!	0	0
0	0	0	0	#NULL!	3	1
0	0	1	0	#NULL!	2	99
0	0	0	0	#NULL!	0	0
1	0	1	1	#NULL!	0	0
0	0	0	1	#NULL!	1	1
0	0	0	1	#NULL!	2	2
0	0	1	1	#NULL!	2	2
0	0	2	1	#NULL!	2	3
0	0	1	0	#NULL!	1	0
0	0	1	1	#NULL!	2	1
0	0	1	1	#NULL!	0	0
1	1	1	1	#NULL!	0	99
0	0	2	2	#NULL!	4	2
2	0	2	0	#NULL!	4	4
1	1	1	1	#NULL!	4	2
0	0	2	1	#NULL!	2	1
1	0	2	1	#NULL!	2	0
0	0	1	0	#NULL!	2	0
0	1	1	0	#NULL!	4	1
0	0	0	0	#NULL!	0	0

2	0	2	2	#NULL!	2	0
0	0	1	1	#NULL!	2	1
0	0	1	0	#NULL!	0	0
0	0	1	1	#NULL!	0	0
1	0	1	2	#NULL!	4	1
0	0	1	0	#NULL!	1	0
0	0	0	0	#NULL!	2	0
0	0	1	0	#NULL!	0	0
2	0	2	2	#NULL!	3	0
0	0	1	1	#NULL!	1	0
0	0	1	0	#NULL!	0	0
0	0	0	0	#NULL!	2	4
0	0	0	0	#NULL!	1	0
0	0	0	0	#NULL!	0	0
0	0	0	0	#NULL!	3	1
0	0	0	0	#NULL!	0	0
0	0	0	0	#NULL!	4	1
0	0	99	1	#NULL!	1	0
0	0	0	0	#NULL!	1	1
0	1	2	2	#NULL!	1	1
0	1	1	1	#NULL!	2	2
0	0	0	0	#NULL!	2	1
1	0	2	1	#NULL!	0	1
0	1	0	0	#NULL!	4	4
99	0	0	0	#NULL!	2	0
0	0	0	0	#NULL!	2	0
0	0	0	1	#NULL!	2	0
0	0	0	0	#NULL!	2	1
0	0	1	0	#NULL!	1	0
1	0	2	1	#NULL!	4	3
1	0	1	1	#NULL!	3	3
0	0	1	0	#NULL!	1	1
0	0	0	0	#NULL!	4	4
0	1	1	2	#NULL!	0	1
1	0	1	1	#NULL!	4	3
0	0	1	0	#NULL!	2	0
99	99	99	99	#NULL!	99	99
0	0	0	0	#NULL!	2	0
0	0	0	1	#NULL!	1	0
0	0	0	0	#NULL!	1	0
0	0	1	0	#NULL!	0	0
0	0	2	2	#NULL!	3	0
1	1	2	2	#NULL!	2	0
1	0	0	0	#NULL!	0	0
0	0	1	1	#NULL!	3	4
0	0	2	2	#NULL!	3	2
0	0	1	0	#NULL!	1	0
0	0	0	0	#NULL!	1	0

0	0	0	0	#NULL!	4	4
0	0	0	1	#NULL!	2	99
0	0	1	1	#NULL!	2	1
0	0	0	0	#NULL!	4	1
0	0	0	0	#NULL!	4	3
1	0	1	2	#NULL!	2	0
0	0	1	0	#NULL!	1	0
0	0	1	1	#NULL!	1	0
1	1	1	1	#NULL!	0	0
0	0	99	0	#NULL!	1	0
1	0	1	0	#NULL!	3	0
0	0	0	0	#NULL!	0	0
2	2	2	2	#NULL!	2	2
0	0	0	0	#NULL!	0	0
0	0	1	0	#NULL!	2	4
0	0	0	0	#NULL!	2	0
0	0	0	0	#NULL!	1	0
1	0	2	0	#NULL!	3	3
0	0	0	0	#NULL!	0	1
0	0	0	1	#NULL!	1	1
0	0	1	1	#NULL!	0	0
0	0	1	1	#NULL!	1	0
0	0	0	0	#NULL!	2	1
0	0	0	1	#NULL!	2	1
0	0	0	0	#NULL!	0	0
0	0	0	1	#NULL!	2	0
0	0	1	1	#NULL!	2	2
1	0	1	1	#NULL!	0	0
1	0	1	0	#NULL!	4	3
0	0	2	2	#NULL!	2	2
1	1	1	1	#NULL!	0	0
0	0	0	1	#NULL!	1	0
0	0	0	0	#NULL!	0	0
0	1	1	2	#NULL!	2	3
0	1	1	1	#NULL!	0	0
0	1	1	1	#NULL!	2	1
1	0	1	1	#NULL!	0	2
1	1	1	1	#NULL!	2	0
0	0	0	0	#NULL!	0	0
1	1	1	0	#NULL!	1	0
0	0	0	0	#NULL!	2	0
1	0	1	2	#NULL!	4	0
0	0	0	0	#NULL!	0	0
0	0	1	0	#NULL!	2	1
0	0	0	0	#NULL!	0	0
0	0	1	0	#NULL!	1	0
0	2	2	2	#NULL!	2	0
0	0	2	2	#NULL!	2	2

0	0	0	0	#NULL!	0	0
0	0	1	1	#NULL!	2	2
0	0	0	0	#NULL!	0	0
0	0	1	0	#NULL!	0	0
0	0	0	0	#NULL!	0	0
99	0	0	0	#NULL!	0	0
0	1	2	1	#NULL!	4	3
0	0	1	1	#NULL!	2	0
0	0	0	0	#NULL!	0	0
0	0	1	1	#NULL!	1	0
0	0	1	1	#NULL!	0	0
0	0	1	0	#NULL!	0	0
0	0	0	0	#NULL!	4	1
0	0	1	0	#NULL!	2	1
0	0	0	0	#NULL!	3	3
1	0	1	0	#NULL!	0	0
0	0	0	0	#NULL!	0	1
0	0	0	0	#NULL!	2	1
0	0	0	0	#NULL!	0	0
0	0	0	0	#NULL!	4	0
0	0	0	0	#NULL!	1	1
0	0	0	0	#NULL!	0	1
0	1	1	0	#NULL!	4	4
0	0	0	0	#NULL!	4	3
0	0	1	1	#NULL!	0	0
0	0	0	0	#NULL!	1	0
0	0	1	0	#NULL!	1	1
0	0	0	0	#NULL!	0	0
0	0	0	0	#NULL!	1	0
0	0	0	0	#NULL!	1	2
0	0	0	0	#NULL!	3	2
0	0	0	0	#NULL!	0	0
0	0	0	0	#NULL!	0	0
99	99	1	99	#NULL!	2	0
0	0	1	1	#NULL!	0	1
0	0	0	0	#NULL!	2	0
0	0	0	0	#NULL!	0	1
0	0	1	0	#NULL!	1	0
0	0	1	1	#NULL!	0	0
0	0	0	0	#NULL!	1	2
1	0	1	0	#NULL!	0	0
0	0	0	0	#NULL!	0	0
0	0	0	0	#NULL!	0	0
0	0	0	0	#NULL!	0	0
1	1	2	2	#NULL!	2	0
1	0	1	1	#NULL!	1	0
0	0	0	0	#NULL!	2	0
0	0	0	1	#NULL!	0	0

0	0	0	0	#NULL!	2	0
1	1	1	1	#NULL!	3	4
0	0	0	0	#NULL!	1	0
0	0	0	0	#NULL!	2	0
0	0	1	1	#NULL!	4	1
0	1	0	0	#NULL!	99	99
0	0	0	0	#NULL!	1	0
0	0	2	1	#NULL!	1	1
0	0	0	0	#NULL!	1	0
0	0	0	0	#NULL!	2	0
0	0	1	1	#NULL!	1	0
0	0	0	0	#NULL!	2	1
0	0	1	1	#NULL!	0	0
2	2	2	2	#NULL!	0	0
0	0	2	1	#NULL!	0	0
0	0	0	0	#NULL!	0	1
0	0	0	0	#NULL!	0	1
0	0	1	0	#NULL!	0	0
1	0	2	2	#NULL!	2	0
0	1	1	1	#NULL!	3	2
0	1	1	1	#NULL!	0	0
99	99	99	99	#NULL!	3	4
0	0	1	1	#NULL!	2	0
1	0	1	1	#NULL!	3	1
0	0	1	1	#NULL!	2	0
0	99	0	99	#NULL!	0	2
0	0	1	0	#NULL!	0	0
0	0	1	1	#NULL!	1	0
2	0	1	1	#NULL!	3	2
0	0	0	0	#NULL!	2	1
1	0	1	1	#NULL!	2	1
0	0	2	0	#NULL!	3	1
2	1	1	2	#NULL!	1	1
1	0	2	2	#NULL!	0	0
0	0	1	1	#NULL!	1	1
0	0	0	0	#NULL!	1	2
99	99	99	99	#NULL!	99	99
0	0	2	2	#NULL!	2	1
0	0	0	0	#NULL!	2	1
0	0	1	1	#NULL!	0	0
0	0	1	1	#NULL!	1	0
2	2	2	2	#NULL!	1	0
0	0	1	1	#NULL!	0	0
0	0	0	0	#NULL!	0	0
0	0	1	0	#NULL!	1	0
0	0	0	0	#NULL!	0	0
0	1	0	0	#NULL!	1	0
0	0	1	0	#NULL!	1	0

0	99	0	0	#NULL!	2	3
0	0	0	0	#NULL!	1	0
0	0	0	0	#NULL!	0	0
0	0	1	0	#NULL!	0	0
0	0	0	0	#NULL!	2	0
0	0	0	0	#NULL!	0	0
0	0	0	0	#NULL!	2	2
0	0	0	0	#NULL!	3	2
0	0	0	0	#NULL!	0	0
0	0	0	0	#NULL!	0	0
0	0	0	0	#NULL!	0	0
0	0	0	1	#NULL!	1	1
1	0	1	0	#NULL!	2	0
0	0	0	0	#NULL!	1	1
0	0	1	1	#NULL!	2	1
0	0	0	0	#NULL!	0	1
1	0	0	1	#NULL!	0	0
0	0	0	0	#NULL!	1	0
0	0	1	0	#NULL!	0	1
0	0	0	0	#NULL!	0	0
0	0	0	0	#NULL!	0	0
0	0	0	0	#NULL!	0	0
99	99	99	99	#NULL!	99	99
0	0	0	0	#NULL!	0	0
0	0	0	0	#NULL!	4	4
0	0	1	0	#NULL!	2	0
1	1	2	1	#NULL!	0	0
0	0	1	0	#NULL!	2	1
0	0	0	0	#NULL!	2	2
0	0	0	0	#NULL!	0	0
0	0	0	0	#NULL!	0	0
0	0	0	0	#NULL!	0	0
0	0	1	0	#NULL!	1	0
0	0	0	0	#NULL!	0	0
0	1	1	2	#NULL!	1	1
2	0	1	0	#NULL!	3	1
0	0	0	0	#NULL!	0	0
99	99	99	99	#NULL!	99	99
0	0	1	1	#NULL!	2	1
1	0	2	2	#NULL!	2	0
0	0	0	0	#NULL!	2	2
0	0	0	2	#NULL!	2	2
2	0	1	1	#NULL!	2	2
0	0	0	0	#NULL!	2	2
0	0	0	0	#NULL!	0	0
1	0	2	1	#NULL!	2	0
0	0	0	0	#NULL!	4	0
0	0	0	0	#NULL!	3	2

99	99	99	99	#NULL!	99	99
0	1	1	1	#NULL!	0	0
0	0	1	0	#NULL!	1	0
0	0	0	0	#NULL!	0	99
0	0	1	0	#NULL!	2	0
0	0	0	1	#NULL!	0	0
99	99	1	1	#NULL!	0	0
0	0	1	1	#NULL!	1	1
0	0	1	1	#NULL!	1	1
0	0	1	0	#NULL!	1	0
0	0	0	0	#NULL!	2	1
0	0	1	1	#NULL!	1	0
0	0	1	0	#NULL!	1	0
0	0	0	0	#NULL!	0	0
0	1	1	2	#NULL!	1	0
0	0	1	1	#NULL!	3	3
0	0	0	0	#NULL!	0	0
1	0	2	0	#NULL!	4	4
0	0	0	0	#NULL!	1	1
2	2	2	2	#NULL!	4	0
1	0	1	2	#NULL!	1	0
1	1	1	1	#NULL!	2	2
99	99	99	99	#NULL!	99	99
0	0	0	0	#NULL!	0	0
0	0	1	1	#NULL!	3	3
0	0	0	1	#NULL!	2	0
0	0	0	0	#NULL!	2	99
1	1	2	1	#NULL!	1	0
0	0	1	0	#NULL!	3	3
0	0	0	0	#NULL!	0	0
0	0	0	0	#NULL!	0	0
1	0	0	0	#NULL!	99	0
0	0	0	0	#NULL!	4	3
0	0	1	0	#NULL!	4	4
1	0	1	1	#NULL!	3	2
0	0	1	0	#NULL!	0	0
0	0	0	0	#NULL!	1	1
99	99	99	99	#NULL!	0	0
0	0	0	0	#NULL!	0	1
0	0	2	0	#NULL!	4	4
1	0	2	2	#NULL!	1	0
2	2	2	2	#NULL!	2	4
0	0	1	1	#NULL!	4	3
0	0	0	0	#NULL!	0	0
1	1	1	1	#NULL!	3	3
0	0	0	0	#NULL!	0	0
99	99	99	99	#NULL!	99	99
2	1	2	2	#NULL!	1	0

0	0	0	0	#NULL!	4	4
0	2	2	2	#NULL!	4	2
0	0	0	0	#NULL!	1	1
1	0	1	0	#NULL!	0	4
0	0	1	0	#NULL!	4	3
0	0	0	0	#NULL!	0	0
1	0	0	0	#NULL!	0	0
0	0	0	0	#NULL!	3	0
0	1	1	99	#NULL!	2	1
0	0	2	0	#NULL!	4	1
0	0	1	1	#NULL!	2	1
0	0	0	0	#NULL!	0	0
0	0	0	0	#NULL!	1	1
0	0	0	0	#NULL!	0	0
0	0	1	1	#NULL!	1	0
0	0	0	0	#NULL!	4	2
0	0	1	1	#NULL!	0	0
0	0	0	0	#NULL!	2	0
0	0	0	0	#NULL!	0	0
0	0	0	0	#NULL!	2	2
0	0	1	1	#NULL!	1	0
0	0	0	0	#NULL!	0	2
0	0	1	0	#NULL!	1	0
0	0	0	0	#NULL!	0	0
0	0	0	0	#NULL!	4	3
1	1	2	1	#NULL!	1	1
0	0	0	0	#NULL!	0	0
0	0	1	0	#NULL!	0	0
1	0	2	2	#NULL!	0	0
0	0	0	0	#NULL!	0	0
0	0	1	1	#NULL!	1	1
0	0	0	1	#NULL!	0	0
0	0	1	1	#NULL!	2	1
0	0	1	0	#NULL!	2	0
0	0	1	0	#NULL!	2	1
0	0	0	0	#NULL!	0	3
0	0	0	0	#NULL!	1	99
1	0	1	1	#NULL!	0	0
99	99	99	99	#NULL!	99	99
0	0	0	0	#NULL!	0	0
0	0	0	0	#NULL!	0	0
0	0	1	1	#NULL!	3	2
0	0	0	1	#NULL!	0	0
0	0	0	0	#NULL!	0	0
0	0	0	0	#NULL!	0	0
0	0	0	0	#NULL!	0	0
1	0	2	1	#NULL!	2	0
0	1	0	0	#NULL!	1	0

0	1	2	2	#NULL!	0	2
1	0	0	0	#NULL!	1	1
0	1	1	1	#NULL!	2	0
0	0	2	1	#NULL!	0	0
2	99	0	1	#NULL!	2	1
0	0	0	0	#NULL!	1	1
0	0	1	1	#NULL!	2	1
0	0	0	0	#NULL!	4	1
0	0	0	0	#NULL!	0	0
0	0	1	1	#NULL!	0	0
0	0	0	0	#NULL!	4	4
0	1	1	1	#NULL!	0	1
0	1	1	0	#NULL!	2	1
0	0	1	1	#NULL!	1	1
0	1	1	1	#NULL!	0	0
0	0	2	1	#NULL!	4	3
0	0	0	0	#NULL!	0	0
1	0	1	0	#NULL!	3	2
1	0	1	1	#NULL!	2	3
1	1	1	1	#NULL!	0	0
0	0	1	0	#NULL!	3	4
0	0	1	2	#NULL!	2	0
0	0	1	0	#NULL!	0	0
0	2	1	2	#NULL!	0	0
0	0	1	1	#NULL!	1	1
0	1	1	1	#NULL!	1	0
0	0	1	0	#NULL!	99	99
1	0	1	2	#NULL!	4	4
2	0	2	2	#NULL!	2	2
99	99	99	99	#NULL!	2	2
99	0	2	1	#NULL!	1	1
0	0	1	0	#NULL!	3	0
1	0	2	0	#NULL!	0	0
0	0	0	0	#NULL!	1	0
0	0	0	0	#NULL!	1	1
0	0	0	0	#NULL!	0	0
0	0	0	0	#NULL!	1	4
0	0	2	0	#NULL!	0	1
0	0	0	0	#NULL!	1	0
0	0	2	1	#NULL!	1	0
0	0	2	2	#NULL!	1	1
99	99	2	99	#NULL!	99	99
1	99	1	99	#NULL!	0	0
0	0	1	1	#NULL!	0	0
0	0	1	2	#NULL!	0	0
1	0	1	0	#NULL!	2	1
1	1	1	1	#NULL!	2	1
0	0	0	1	#NULL!	2	2

0	0	1	0	#NULL!	0	0
0	0	2	2	#NULL!	3	2
0	0	1	0	#NULL!	0	0
0	0	1	1	#NULL!	2	0
0	0	0	0	#NULL!	1	0
0	0	1	1	#NULL!	1	1
1	0	0	0	#NULL!	0	0
0	0	0	1	#NULL!	4	4
0	0	0	0	#NULL!	0	0
0	0	1	0	#NULL!	1	0
0	0	1	0	#NULL!	2	1
0	0	0	0	#NULL!	0	0
0	0	1	0	#NULL!	2	1
0	0	2	1	#NULL!	2	2
0	0	0	1	#NULL!	0	0
0	0	0	1	#NULL!	2	2
0	0	0	0	#NULL!	0	1
1	1	1	1	#NULL!	1	3
0	0	0	0	#NULL!	0	0
0	0	1	1	#NULL!	3	4
2	1	1	2	#NULL!	0	1
0	0	2	1	#NULL!	3	1
0	0	1	0	#NULL!	4	2
0	0	1	2	#NULL!	0	0
0	0	0	0	#NULL!	2	1
0	0	0	0	#NULL!	2	0
0	0	1	0	#NULL!	0	0
0	0	0	0	#NULL!	1	0
0	0	0	0	#NULL!	0	0
0	0	1	0	#NULL!	3	2
0	1	1	2	#NULL!	2	0
0	0	1	0	#NULL!	0	1
1	0	2	1	#NULL!	0	0
0	0	1	0	#NULL!	2	0
0	0	1	0	#NULL!	0	0
0	0	0	0	#NULL!	1	0
2	1	2	1	#NULL!	1	0
1	0	1	2	#NULL!	1	1
0	0	1	0	#NULL!	99	99
0	1	1	2	#NULL!	0	0
0	0	0	2	#NULL!	1	0
0	0	2	0	#NULL!	3	3
0	0	0	0	#NULL!	3	2
0	0	1	1	#NULL!	3	4
0	0	0	0	#NULL!	1	0
0	0	2	1	#NULL!	2	2
2	1	2	2	#NULL!	0	0
1	0	2	1	#NULL!	3	3

1	0	2	1	#NULL!	4	2
0	0	2	0	#NULL!	4	4
0	0	1	0	#NULL!	1	3
1	0	2	2	#NULL!	3	2
1	1	1	1	#NULL!	2	1
2	0	2	0	#NULL!	2	0
0	0	1	1	#NULL!	1	0
2	2	0	2	#NULL!	2	2
1	0	1	1	#NULL!	2	2
0	0	0	1	#NULL!	0	0
0	0	1	1	#NULL!	2	0
0	0	0	0	#NULL!	2	1
1	0	1	1	#NULL!	1	0
0	1	2	1	#NULL!	1	0
1	1	1	2	#NULL!	2	3
0	1	2	1	#NULL!	4	3
0	1	2	0	#NULL!	3	2
0	0	0	0	#NULL!	0	0
99	99	99	2	#NULL!	1	99
0	1	1	0	#NULL!	1	1
0	0	1	0	#NULL!	0	1
1	0	2	0	#NULL!	2	2
0	0	0	0	#NULL!	0	1
0	0	0	0	#NULL!	0	0
0	0	0	0	#NULL!	0	0
99	0	0	0	#NULL!	0	0
0	0	1	2	#NULL!	0	0
0	0	0	0	#NULL!	1	1
0	0	0	0	#NULL!	2	1
2	2	2	2	#NULL!	4	4
0	0	0	0	#NULL!	0	0
0	0	0	0	#NULL!	0	1
0	0	0	0	#NULL!	0	0
0	0	0	0	#NULL!	0	0
0	0	0	0	#NULL!	0	0
1	0	1	0	#NULL!	2	1
0	0	1	2	#NULL!	0	0
1	1	2	2	#NULL!	0	0
0	0	0	0	#NULL!	1	0
0	0	0	0	#NULL!	2	0
0	0	1	0	#NULL!	0	0
0	0	0	0	#NULL!	0	0
0	0	1	0	#NULL!	2	2
1	0	0	0	#NULL!	1	1
0	0	0	1	#NULL!	4	2
0	0	2	99	#NULL!	4	0
0	0	1	0	#NULL!	0	0
0	2	0	1	#NULL!	4	4

0	0	0	2	#NULL!	1	0
0	0	1	1	#NULL!	0	0
0	0	0	1	#NULL!	0	0
0	0	2	0	#NULL!	3	2
0	1	1	1	#NULL!	0	0
0	0	1	0	#NULL!	2	2
0	0	1	0	#NULL!	4	3
0	0	1	0	#NULL!	2	0
0	1	2	2	#NULL!	2	0
0	0	0	0	#NULL!	1	0
99	99	99	99	#NULL!	99	99
1	0	2	2	#NULL!	2	2
0	0	1	0	#NULL!	0	0
0	0	1	1	#NULL!	2	0
1	1	1	0	#NULL!	0	0
0	0	1	0	#NULL!	3	1
0	0	0	0	#NULL!	2	2
0	0	0	0	#NULL!	0	0
0	1	1	2	#NULL!	1	0
0	0	2	0	#NULL!	3	2
2	0	1	1	#NULL!	3	2
0	0	0	0	#NULL!	1	2
0	0	1	0	#NULL!	0	0
2	2	2	0	#NULL!	2	2
0	0	2	1	#NULL!	0	0
2	2	2	2	#NULL!	2	4
0	0	0	0	#NULL!	0	0
0	0	0	0	#NULL!	4	4
1	0	2	0	#NULL!	4	1
2	1	2	2	#NULL!	2	0
0	0	2	0	#NULL!	2	3
0	0	0	1	#NULL!	4	2
0	0	0	0	#NULL!	3	2
0	1	1	2	#NULL!	2	1
0	0	0	1	#NULL!	2	2
0	0	2	1	#NULL!	1	0
0	0	0	2	#NULL!	1	0
0	0	1	1	#NULL!	1	1
0	0	1	2	#NULL!	0	0
0	0	2	0	#NULL!	0	0
0	0	1	0	#NULL!	0	0
0	0	0	0	#NULL!	1	0
1	0	1	1	#NULL!	0	0
1	0	1	1	#NULL!	2	0
0	0	1	0	#NULL!	2	1
0	0	0	0	#NULL!	4	4
0	0	1	0	#NULL!	1	2
0	0	0	2	#NULL!	2	1

0	0	0	0	#NULL!	0	0
1	0	1	2	#NULL!	1	0
0	0	0	1	#NULL!	2	2
0	1	1	1	#NULL!	4	4
0	2	2	2	#NULL!	4	4
0	0	1	1	#NULL!	0	0
0	0	0	0	#NULL!	0	0
0	1	2	1	#NULL!	0	0
1	0	1	1	#NULL!	0	0
0	0	1	1	#NULL!	4	0
0	0	0	0	#NULL!	3	2
0	0	2	0	#NULL!	1	1
0	1	1	2	#NULL!	0	1
0	0	0	0	#NULL!	1	1
0	1	1	1	#NULL!	1	2
0	0	0	1	#NULL!	1	1
0	0	0	0	#NULL!	0	0
0	0	1	1	#NULL!	1	1
0	0	0	0	#NULL!	0	0
0	0	0	0	#NULL!	0	0
0	0	0	0	#NULL!	0	1
1	0	1	1	#NULL!	2	2
0	0	1	0	#NULL!	2	2
99	99	99	99	#NULL!	0	0
0	0	2	1	#NULL!	2	2
0	0	1	1	#NULL!	2	1
0	0	0	1	#NULL!	4	1
0	0	1	0	#NULL!	1	0
0	0	0	0	#NULL!	3	2
0	0	0	0	#NULL!	0	0
0	0	1	2	#NULL!	2	1
0	0	2	2	#NULL!	2	0
0	0	1	0	#NULL!	0	0
0	0	0	0	#NULL!	3	1
1	1	2	0	#NULL!	1	0
0	0	1	1	#NULL!	2	0
1	0	2	2	#NULL!	4	4
0	0	0	1	#NULL!	2	1
0	0	0	0	#NULL!	2	3
0	0	1	1	#NULL!	1	2
0	0	0	0	#NULL!	2	0
0	0	2	2	#NULL!	4	2
0	0	0	0	#NULL!	0	0
0	1	1	1	#NULL!	3	1
0	0	1	0	#NULL!	0	0
0	1	1	1	#NULL!	2	2
0	0	1	1	#NULL!	0	0
2	2	2	2	#NULL!	0	0

0	0	99	0	#NULL!	2	1
0	0	1	0	#NULL!	2	1
0	0	1	0	#NULL!	0	0
99	99	99	99	#NULL!	2	1
0	0	0	0	#NULL!	0	0
0	0	0	0	#NULL!	0	0
2	2	2	2	#NULL!	2	1
1	0	1	0	#NULL!	2	4
0	0	0	0	#NULL!	0	0
0	1	0	0	#NULL!	2	0
0	0	1	2	#NULL!	4	3
0	1	1	1	#NULL!	2	1
0	0	1	0	#NULL!	0	0
0	0	0	1	#NULL!	1	0
0	0	99	0	#NULL!	1	0
2	2	2	2	#NULL!	1	1
2	1	2	1	#NULL!	0	0
1	1	1	1	#NULL!	1	0
1	1	1	1	#NULL!	1	2
0	0	1	1	#NULL!	1	4
1	0	0	1	#NULL!	0	0
0	0	0	0	#NULL!	0	0
1	0	0	1	#NULL!	1	1
1	0	2	1	#NULL!	2	0
0	0	1	0	#NULL!	3	1
1	1	2	1	#NULL!	2	2
1	1	1	2	#NULL!	0	0
0	1	1	1	#NULL!	0	0
0	0	1	1	#NULL!	4	4
0	0	0	0	#NULL!	1	0
0	0	1	0	#NULL!	3	4
0	0	1	1	#NULL!	2	1
0	1	1	1	#NULL!	1	2
0	0	0	0	#NULL!	2	0
0	1	2	1	#NULL!	2	1
0	0	1	1	#NULL!	0	0
0	2	2	2	#NULL!	0	0
0	0	0	0	#NULL!	0	0
0	0	1	0	#NULL!	2	1
1	1	1	1	#NULL!	1	1
0	0	0	0	#NULL!	0	0
0	0	0	0	#NULL!	2	0
0	1	1	1	#NULL!	2	1
0	0	0	1	#NULL!	1	0
0	0	0	0	#NULL!	4	3
0	0	0	0	#NULL!	1	4
0	0	0	0	#NULL!	2	4
1	0	0	1	#NULL!	1	0

0	0	0	0	#NULL!	1	4
2	2	0	2	#NULL!	0	0
0	0	0	0	#NULL!	2	1
0	0	0	0	#NULL!	0	0
0	0	0	0	#NULL!	2	1
0	0	0	0	#NULL!	0	0
0	0	0	0	#NULL!	0	0
0	0	0	0	#NULL!	1	0
0	0	0	0	#NULL!	1	0
0	0	0	1	#NULL!	3	0
0	0	0	2	#NULL!	1	0
0	1	0	1	#NULL!	1	0
0	0	0	0	#NULL!	1	0
0	0	0	0	#NULL!	0	0
0	0	0	0	#NULL!	1	1
0	1	1	0	#NULL!	2	2
0	0	1	0	#NULL!	0	0
0	0	2	0	#NULL!	2	2
0	0	1	0	#NULL!	2	4
0	0	1	1	#NULL!	2	0
0	0	1	0	#NULL!	2	1
2	0	2	2	#NULL!	4	2
0	0	1	1	#NULL!	0	0
0	0	0	0	#NULL!	0	0
0	0	0	0	#NULL!	0	0
0	0	0	0	#NULL!	1	0
0	0	0	1	#NULL!	1	0
0	0	0	0	#NULL!	0	0
0	1	0	0	#NULL!	0	0
0	0	0	0	#NULL!	2	2
2	0	2	2	#NULL!	4	4
0	0	0	0	#NULL!	0	1
0	0	1	1	#NULL!	3	2
0	0	0	0	#NULL!	3	4
0	0	1	0	#NULL!	1	0
0	0	0	0	#NULL!	0	0
0	0	1	2	#NULL!	0	2
0	1	1	1	#NULL!	2	2
0	0	0	0	#NULL!	2	2
0	0	2	1	#NULL!	0	0
0	0	0	0	#NULL!	2	0
0	0	0	0	#NULL!	2	0
0	0	0	0	#NULL!	4	3
0	0	1	1	#NULL!	1	1
0	0	2	1	#NULL!	4	4
0	1	0	1	#NULL!	0	0
0	0	0	0	#NULL!	0	0
0	1	99	1	#NULL!	2	2

0	0	0	0	#NULL!	2	0
2	1	2	2	#NULL!	4	4
0	0	0	0	#NULL!	0	0
0	0	1	0	#NULL!	1	1
0	0	1	0	#NULL!	0	0
0	0	0	0	#NULL!	3	3
1	0	0	0	#NULL!	3	0
0	0	1	1	#NULL!	4	3
1	0	2	1	#NULL!	4	3
1	1	2	2	#NULL!	1	0
1	0	2	2	#NULL!	2	3
0	0	0	0	#NULL!	0	0
0	0	2	0	#NULL!	3	2
0	0	0	0	#NULL!	1	1
2	0	2	2	#NULL!	3	2
0	1	1	1	#NULL!	0	0
0	0	0	1	#NULL!	0	0
0	0	0	0	#NULL!	2	2
0	0	1	1	#NULL!	1	0
0	0	0	0	#NULL!	0	1
0	0	0	0	#NULL!	0	0
0	0	1	0	#NULL!	2	2
99	99	2	2	#NULL!	2	1
0	0	0	0	#NULL!	0	0
0	0	0	2	#NULL!	0	0
1	0	1	1	#NULL!	3	2
0	0	0	0	#NULL!	2	0
0	0	0	0	#NULL!	0	1
0	0	1	0	#NULL!	0	0
0	0	1	1	#NULL!	1	2
1	1	1	1	#NULL!	1	0
0	1	1	0	#NULL!	0	0
0	0	1	0	#NULL!	0	0
0	0	0	0	#NULL!	3	4
1	1	1	1	#NULL!	1	0
0	0	1	1	#NULL!	1	2
0	0	0	0	#NULL!	0	0
0	0	0	1	#NULL!	1	0
0	0	1	0	#NULL!	0	0
0	0	0	0	#NULL!	0	0
1	0	1	0	#NULL!	4	3
99	99	99	99	#NULL!	1	0
1	0	1	0	#NULL!	3	2
1	0	2	0	#NULL!	3	2
0	0	0	0	#NULL!	0	0
0	0	0	2	#NULL!	2	3
0	0	1	1	#NULL!	0	0
1	0	1	0	#NULL!	0	1

0	1	1	0	#NULL!	4	2
0	0	2	1	#NULL!	1	0
0	0	1	2	#NULL!	2	1
0	0	0	0	#NULL!	3	3
0	0	0	0	#NULL!	0	1
1	0	2	2	#NULL!	4	4
0	0	0	0	#NULL!	0	0
0	0	0	0	#NULL!	0	0
0	0	1	1	#NULL!	0	0
2	2	1	2	#NULL!	0	0
0	0	0	0	#NULL!	0	0
0	0	1	0	#NULL!	0	0
0	0	1	0	#NULL!	0	0
0	0	0	0	#NULL!	2	1
0	1	1	1	#NULL!	3	4
0	0	0	0	#NULL!	1	0
2	1	2	2	#NULL!	2	0
0	0	0	0	#NULL!	2	3
0	0	0	0	#NULL!	0	0
0	0	1	1	#NULL!	0	0
0	0	2	1	#NULL!	1	0
0	0	0	1	#NULL!	2	2
0	0	0	1	#NULL!	0	0
0	0	1	1	#NULL!	2	0
0	0	0	0	#NULL!	2	0
0	0	1	0	#NULL!	3	1
0	0	0	0	#NULL!	0	0
0	1	1	1	#NULL!	2	1
0	0	1	0	#NULL!	0	0
0	0	0	0	#NULL!	0	0
0	0	1	0	#NULL!	1	2
1	0	1	1	#NULL!	0	0
0	0	1	0	#NULL!	1	2
0	0	0	1	#NULL!	3	2
1	0	2	2	#NULL!	4	3
0	0	1	1	#NULL!	0	0
0	0	0	0	#NULL!	0	0
0	0	0	0	#NULL!	1	1
0	0	2	2	#NULL!	3	2
0	0	1	0	#NULL!	0	0
0	1	99	2	#NULL!	0	0
0	2	1	2	#NULL!	3	2
0	0	0	0	#NULL!	1	0
0	0	1	0	#NULL!	2	1
0	0	2	2	#NULL!	2	2
0	2	2	2	#NULL!	0	0
1	0	1	2	#NULL!	3	1
1	1	1	1	#NULL!	0	0

0	0	1	0	#NULL!	3	3
0	0	1	0	#NULL!	1	0
0	0	1	1	#NULL!	2	0
0	0	0	0	0	#NULL!	#NULL!
0	0	2	1	2	#NULL!	#NULL!
0	0	0	0	1	#NULL!	#NULL!
0	0	0	0	0	#NULL!	#NULL!
2	0	1	0	1	#NULL!	#NULL!
0	0	2	2	2	#NULL!	#NULL!
0	0	2	2	2	#NULL!	#NULL!
1	0	1	1	1	#NULL!	#NULL!
1	1	2	2	1	#NULL!	#NULL!
2	2	2	2	2	#NULL!	#NULL!
1	0	1	0	1	#NULL!	#NULL!
2	0	1	1	2	#NULL!	#NULL!
0	0	1	1	1	#NULL!	#NULL!
0	0	1	0	1	#NULL!	#NULL!
1	0	2	2	2	#NULL!	#NULL!
0	0	1	1	0	#NULL!	#NULL!
2	0	2	2	1	#NULL!	#NULL!
2	1	2	2	2	#NULL!	#NULL!
0	0	2	1	2	#NULL!	#NULL!
0	2	2	1	1	#NULL!	#NULL!
0	0	0	0	0	#NULL!	#NULL!
0	0	0	0	2	#NULL!	#NULL!
1	1	1	1	1	#NULL!	#NULL!
0	0	1	1	1	#NULL!	#NULL!
0	0	0	0	2	#NULL!	#NULL!
0	0	1	0	0	#NULL!	#NULL!
0	0	0	0	1	#NULL!	#NULL!
0	0	0	0	0	#NULL!	#NULL!
2	2	2	2	2	#NULL!	#NULL!
1	0	2	2	2	#NULL!	#NULL!
0	0	1	0	0	#NULL!	#NULL!
0	0	2	0	0	#NULL!	#NULL!
2	0	2	2	2	#NULL!	#NULL!
0	0	0	0	0	#NULL!	#NULL!
0	0	0	0	0	#NULL!	#NULL!
0	0	1	0	0	#NULL!	#NULL!
0	0	0	0	0	#NULL!	#NULL!
0	0	0	0	0	#NULL!	#NULL!
0	1	1	1	1	#NULL!	#NULL!
2	0	2	0	2	#NULL!	#NULL!
0	0	0	0	0	#NULL!	#NULL!
1	0	0	0	0	#NULL!	#NULL!
1	1	1	1	1	#NULL!	#NULL!
0	0	0	0	0	#NULL!	#NULL!
0	0	0	0	1	#NULL!	#NULL!

0	0	1	0	0	#NULL!	#NULL!
0	0	1	0	0	#NULL!	#NULL!
1	0	0	0	1	#NULL!	#NULL!
0	0	0	0	2	#NULL!	#NULL!
#NULL!	0	1	0	1	#NULL!	#NULL!
0	0	0	0	0	#NULL!	#NULL!
1	0	1	0	1	#NULL!	#NULL!
0	0	0	0	0	#NULL!	#NULL!
0	0	0	0	0	#NULL!	#NULL!
0	0	1	0	2	#NULL!	#NULL!
0	0	0	0	0	#NULL!	#NULL!
0	0	0	0	0	#NULL!	#NULL!
#NULL!	#NULL!	#NULL!	#NULL!	#NULL!	#NULL!	#NULL!
0	0	1	0	1	#NULL!	#NULL!
1	0	0	0	2	#NULL!	#NULL!
0	0	0	0	0	#NULL!	#NULL!
0	0	0	0	2	#NULL!	#NULL!
0	1	2	2	2	#NULL!	#NULL!
0	0	0	0	0	#NULL!	#NULL!
0	0	1	0	2	#NULL!	#NULL!
0	0	0	0	2	#NULL!	#NULL!
1	0	0	0	2	#NULL!	#NULL!
0	0	0	0	0	#NULL!	#NULL!
0	0	0	0	1	#NULL!	#NULL!
0	0	1	0	1	#NULL!	#NULL!
0	0	0	0	1	#NULL!	#NULL!
2	1	1	1	2	#NULL!	#NULL!
0	0	0	0	2	#NULL!	#NULL!
0	0	0	0	0	#NULL!	#NULL!
0	0	0	0	0	#NULL!	#NULL!
0	0	1	0	1	#NULL!	#NULL!
0	0	0	0	0	#NULL!	#NULL!
1	0	1	1	1	#NULL!	#NULL!
0	0	0	0	0	#NULL!	#NULL!
0	0	1	0	1	#NULL!	#NULL!
0	0	0	0	0	#NULL!	#NULL!
0	0	1	0	0	#NULL!	#NULL!
0	0	0	0	0	#NULL!	#NULL!
0	0	0	0	0	#NULL!	#NULL!
0	0	1	0	1	#NULL!	#NULL!
0	0	0	0	0	#NULL!	#NULL!
0	0	0	0	0	#NULL!	#NULL!
0	0	0	0	1	#NULL!	#NULL!
0	0	0	0	0	#NULL!	#NULL!
0	0	0	0	0	#NULL!	#NULL!
2	0	1	1	1	#NULL!	#NULL!
0	0	2	0	2	#NULL!	#NULL!
0	0	0	0	1	#NULL!	#NULL!

0	0	0	0	2	#NULL!	#NULL!
0	0	0	0	0	#NULL!	#NULL!
0	0	1	0	0	#NULL!	#NULL!
0	0	0	0	0	#NULL!	#NULL!
0	0	0	0	2	#NULL!	#NULL!
0	0	0	0	2	#NULL!	#NULL!
0	0	0	0	2	#NULL!	#NULL!
0	0	0	0	2	#NULL!	#NULL!
0	0	0	0	1	#NULL!	#NULL!
0	0	1	1	0	#NULL!	#NULL!
#NULL!	0	0	0	0	#NULL!	#NULL!
0	0	0	0	1	#NULL!	#NULL!
0	0	0	0	0	#NULL!	#NULL!
1	0	1	1	2	#NULL!	#NULL!
0	0	2	0	2	#NULL!	#NULL!
0	0	0	0	0	#NULL!	#NULL!
#NULL!	#NULL!	#NULL!	#NULL!	#NULL!	#NULL!	#NULL!
2	2	2	2	2	#NULL!	#NULL!
0	0	0	1	1	#NULL!	#NULL!
0	0	0	0	2	#NULL!	#NULL!
0	0	0	0	0	#NULL!	#NULL!

bullyc_pre	bullyd_pre	bullye_pre	bullyf_pre	pconnecta_pre	pconnectb_pre	pconnectc_pre
0	0	0	0	4	4	4
0	0	0	0	99	99	99
4	0	0	0	1	1	0
1	2	4	4	4	4	3
0	0	0	0	4	4	4
99	99	99	99	99	99	99
0	0	0	0	4	4	4
0	2	0	0	2	3	0
99	99	99	99	99	99	99
2	0	0	0	3	2	2
1	1	0	0	99	99	99
0	1	0	0	3	4	3
0	0	0	0	4	4	3
0	2	0	1	0	1	0
0	0	0	0	4	4	4
1	1	0	0	0	0	0
0	0	0	0	4	4	4
0	1	1	0	1	4	4
0	1	0	0	3	4	4
0	2	0	0	2	4	2
99	99	99	99	99	99	99
0	0	0	0	0	99	4
0	0	0	0	3	4	99
0	0	0	0	4	4	4
0	0	0	0	4	4	4
0	0	0	0	4	4	4
0	0	0	0	4	4	4
0	1	0	0	4	2	1
0	0	0	0	4	4	3
0	99	1	0	4	4	4
0	2	0	0	4	4	4
0	4	0	0	4	4	2
1	0	1	0	4	4	4
0	0	0	0	4	4	4
0	0	1	0	1	99	99
0	0	0	0	4	4	2
0	0	2	0	4	3	4
0	0	1	0	4	4	2
2	0	0	0	4	4	4
0	4	4	0	4	3	2
0	1	2	4	4	4	4
0	1	0	0	4	4	4
99	99	99	99	3	2	3
0	0	0	0	4	4	4
0	0	0	0	4	4	4
0	1	0	0	3	4	4
0	2	1	0	2	3	3

0	2	0	0	4	2	1
0	1	1	0	4	4	4
0	0	0	0	3	4	4
3	4	2	0	3	3	99
3	2	3	0	4	4	4
0	2	3	3	2	2	2
0	0	0	0	4	4	4
2	1	2	0	2	4	3
0	0	0	0	4	4	4
0	0	0	0	4	4	4
0	0	0	0	0	3	0
0	1	0	0	4	4	4
0	0	0	0	1	4	1
0	0	0	0	1	1	1
0	0	0	0	4	4	4
0	1	4	0	2	1	0
1	2	3	0	4	4	3
0	4	1	1	4	3	4
0	0	0	0	2	1	2
0	0	0	0	4	3	4
1	0	0	0	2	2	2
0	0	0	0	4	4	4
1	2	2	0	3	3	2
3	4	0	0	4	2	0
0	0	0	0	4	4	4
0	0	0	0	4	4	4
0	4	1	0	4	4	4
0	0	0	0	2	2	2
0	3	1	0	3	2	3
0	1	0	0	4	4	4
0	0	0	0	4	4	4
0	2	1	0	4	4	4
0	1	1	0	4	4	4
2	2	0	1	3	4	2
0	0	0	0	4	2	3
0	1	0	0	0	0	0
0	0	0	0	2	1	1
1	2	0	0	4	4	3
2	3	2	0	4	4	3
0	0	0	0	4	4	4
0	1	0	0	3	4	2
0	0	0	0	3	4	4
1	2	0	2	3	4	4
0	0	0	0	4	4	4
0	0	0	0	3	4	4
0	0	0	0	4	4	4
0	1	0	0	4	4	4
99	1	0	0	2	2	1

1	0	0	0	1	2	2
2	2	2	2	2	2	2
0	1	0	0	4	4	4
0	1	0	0	4	4	4
0	0	0	0	4	4	4
0	0	0	0	4	4	4
0	0	0	0	4	4	2
0	0	0	0	4	4	4
0	4	2	0	4	4	4
4	4	4	4	2	2	1
0	1	0	0	4	4	4
2	0	4	0	3	3	2
0	0	0	0	3	3	3
1	0	0	0	3	4	3
0	0	0	0	0	4	0
3	4	0	0	2	2	0
0	1	3	3	4	4	4
3	0	0	0	3	4	3
0	0	0	0	4	4	4
0	0	0	0	0	2	2
4	2	0	0	4	4	4
0	2	0	0	4	4	4
0	0	0	0	0	0	0
2	3	4	0	4	4	4
0	0	0	0	4	4	4
2	0	0	0	2	2	2
1	0	2	2	3	3	4
0	0	0	0	4	4	4
4	0	0	0	4	4	4
0	1	0	0	3	4	4
0	2	0	0	99	99	2
2	0	0	0	3	2	3
99	99	99	99	99	99	99
0	99	0	0	4	4	4
0	0	0	0	4	4	4
0	0	0	0	4	4	4
0	0	0	0	4	4	3
0	1	0	0	1	4	2
0	3	2	1	2	3	3
0	4	2	0	3	4	3
0	0	0	0	4	4	4
0	0	3	0	4	4	3
0	2	2	2	4	4	4
0	2	0	0	2	3	2
0	0	0	0	4	4	4
99	99	99	99	99	99	99
4	4	4	4	3	2	2
0	1	2	2	1	3	2

0	0	0	0	2	3	3
0	0	0	0	4	4	4
0	1	0	0	4	4	4
0	2	1	2	4	4	4
0	1	0	0	4	4	4
99	99	99	99	3	4	4
0	0	0	0	0	0	0
0	0	0	0	4	4	4
0	0	1	0	4	4	4
1	0	0	0	2	1	1
0	1	0	0	4	4	4
0	0	0	0	0	0	0
0	1	0	0	2	3	3
0	1	0	0	4	4	4
0	0	0	0	4	4	3
0	0	0	0	4	4	3
0	0	2	0	4	4	4
0	0	0	0	3	4	3
0	2	1	0	3	1	1
0	0	0	0	3	4	99
4	0	0	0	1	1	2
0	0	0	0	4	4	4
0	0	0	0	4	4	4
0	0	0	0	4	4	4
0	0	0	0	4	4	4
0	0	0	0	4	4	4
0	1	0	0	3	99	99
2	3	4	0	4	4	4
0	0	0	0	3	4	0
0	0	0	0	4	4	4
0	0	0	0	0	0	0
0	0	0	0	4	4	4
0	0	0	0	4	4	0
0	0	0	0	4	4	4
0	0	0	0	4	4	4
2	0	0	0	4	4	3
0	0	0	0	4	4	4
0	1	0	0	4	4	0
0	3	0	2	4	4	4
0	0	0	0	4	4	4
0	0	0	0	4	4	4
1	0	0	0	4	4	4
0	0	0	0	4	4	3
0	0	0	0	3	2	1
0	0	0	0	4	4	4
0	0	0	0	4	4	4
0	1	0	0	2	4	3
0	0	0	0	4	4	4

0	0	0	0	4	4	4
0	0	0	0	4	4	4
2	0	1	0	4	4	4
0	1	0	0	4	4	2
0	3	3	0	3	4	1
2	0	0	0	3	3	3
0	0	0	0	4	4	3
1	0	0	0	2	2	1
0	0	0	0	0	0	0
4	3	99	0	1	2	2
0	0	0	0	4	4	4
0	0	0	0	4	4	4
0	2	0	0	2	4	1
99	99	99	99	4	4	4
0	0	0	0	3	3	4
0	0	0	0	4	4	4
0	0	0	0	4	4	4
0	0	0	0	0	99	1
1	0	0	0	4	4	4
0	0	0	0	1	2	2
0	0	0	0	4	4	4
0	0	0	0	4	4	4
0	0	0	0	2	0	2
0	0	0	0	2	1	2
4	4	4	4	4	4	2
1	0	4	1	4	4	4
1	0	0	0	2	1	3
2	1	2	0	2	2	0
3	2	0	0	0	0	0
4	0	0	0	4	4	4
1	0	0	0	4	4	4
0	0	0	0	4	3	4
0	0	0	0	4	4	4
0	0	0	0	99	2	2
1	4	4	4	4	4	4
1	4	4	0	1	2	0
0	0	0	0	1	2	2
0	0	0	0	3	4	2
0	0	0	0	3	3	3
1	0	0	0	4	3	4
1	2	99	99	2	2	1
2	0	0	0	3	3	3
0	0	0	0	4	4	4
0	0	0	0	4	4	4
0	1	0	0	0	1	0
0	0	0	0	2	1	3
0	0	0	0	4	4	4
0	0	0	0	3	4	0

1	1	1	1	3	4	4
1	4	1	0	4	4	4
0	0	0	0	2	2	3
0	0	0	0	3	3	4
2	1	0	0	4	4	4
0	1	0	0	4	4	4
3	4	4	2	4	4	4
2	1	0	0	4	4	4
0	2	1	1	3	4	3
0	0	0	0	4	4	4
0	1	0	0	2	4	4
0	0	0	0	4	4	4
0	0	4	0	4	4	3
0	0	0	0	3	4	3
0	0	0	0	4	4	4
2	0	0	0	3	4	3
4	2	0	0	0	1	1
1	1	0	2	4	4	4
3	2	2	2	99	99	99
0	0	0	0	4	4	3
0	1	1	0	4	4	4
3	4	3	0	3	3	3
0	2	1	1	4	4	3
1	0	0	0	3	4	3
2	3	0	0	3	4	3
0	1	0	0	4	4	4
0	0	0	0	4	4	4
0	0	0	0	4	4	3
1	0	0	0	4	3	3
0	0	0	0	4	4	4
0	0	0	0	3	4	3
0	1	0	0	4	3	3
0	1	0	0	4	3	4
0	0	0	0	4	4	4
0	0	0	0	2	4	4
0	0	0	0	3	4	3
1	1	0	1	3	3	3
0	0	0	0	4	4	4
0	2	2	0	4	4	4
0	4	0	0	4	4	4
2	3	2	2	2	2	0
0	0	0	0	3	3	3
0	1	2	0	3	2	1
0	0	0	0	4	4	3
0	2	1	0	4	4	4
0	1	3	1	1	0	2
1	2	1	0	4	4	4
0	1	0	0	4	4	3

0	0	0	0	4	4	4
1	1	0	0	4	4	4
0	0	1	0	3	3	3
0	1	0	0	99	99	99
0	0	0	0	4	4	4
0	0	0	0	1	4	0
1	0	0	0	4	3	3
0	1	0	0	4	4	4
0	0	0	0	4	4	4
2	0	0	0	4	4	4
0	0	0	0	4	4	4
0	0	0	0	3	3	3
0	0	0	0	3	4	2
1	1	0	0	4	4	3
3	0	2	0	3	4	3
0	0	0	0	4	4	4
0	0	0	0	4	4	4
0	2	0	0	4	4	4
0	0	0	0	4	4	4
2	0	0	0	2	4	3
1	0	0	0	4	4	3
1	3	1	0	1	3	2
0	0	0	0	3	4	4
0	1	0	0	1	2	2
1	99	1	0	4	4	4
0	0	0	0	4	4	2
1	0	1	0	4	4	4
1	2	2	1	2	3	3
0	2	2	0	4	4	4
0	0	0	0	2	2	0
0	1	0	0	4	4	4
0	1	0	0	2	3	2
2	3	3	3	4	4	4
0	0	0	0	4	3	4
0	0	0	0	4	4	4
0	0	0	0	3	4	2
0	0	0	0	3	2	4
0	0	0	0	4	4	3
0	0	0	0	4	4	4
1	0	0	0	4	4	4
1	3	2	1	3	4	3
2	0	0	0	4	4	4
0	0	0	0	3	3	2
0	4	2	0	4	3	4
0	1	1	0	4	4	4
0	1	0	0	4	4	3
4	4	2	0	4	2	4
0	0	0	0	4	4	4

0	1	1	0	4	4	4
0	2	2	0	3	4	2
0	0	0	0	4	4	3
0	1	0	0	4	4	2
1	1	0	1	3	3	2
99	99	99	99	99	99	99
2	4	3	0	1	2	0
0	0	0	0	3	4	4
0	0	0	0	4	4	4
0	4	0	0	2	2	2
0	0	0	0	4	4	4
0	0	0	0	4	4	4
1	0	1	1	2	2	2
0	3	4	0	3	4	3
0	1	2	0	4	4	4
0	0	0	0	3	4	3
0	2	2	0	2	2	3
0	0	0	0	4	4	3
0	1	0	0	4	4	4
0	0	0	0	3	4	3
0	0	0	0	4	4	3
1	0	0	0	3	4	2
2	0	0	0	4	4	4
0	1	1	0	3	2	2
2	3	0	0	2	3	2
0	2	1	0	3	4	4
0	0	0	0	4	4	4
0	0	0	0	99	99	99
0	0	0	0	2	4	3
0	0	1	0	4	4	4
0	0	0	0	4	3	3
0	0	0	0	4	4	0
0	0	0	0	4	4	4
0	0	0	0	4	2	3
0	3	4	99	4	4	4
0	0	0	0	4	4	4
1	4	1	0	4	3	4
0	0	0	1	4	4	4
0	0	0	1	4	4	3
0	1	0	0	4	4	2
0	0	0	0	2	4	3
0	4	0	0	4	4	4
1	0	0	0	2	3	2
1	0	0	0	2	3	3
0	0	0	0	4	4	2
0	0	0	0	4	4	4
0	0	0	0	4	4	4
0	0	0	0	4	4	4

0	0	0	0	4	4	4
0	0	0	0	4	4	4
0	0	0	0	3	3	4
1	4	2	0	2	2	2
0	2	3	4	3	4	4
99	1	0	0	4	4	4
0	0	0	0	4	4	4
4	4	3	0	0	0	0
0	2	3	2	4	4	4
4	4	4	4	4	4	4
0	0	0	0	4	4	4
1	0	0	0	3	4	3
0	1	0	0	2	2	2
0	0	0	0	4	4	3
0	0	0	0	4	4	4
1	2	3	1	4	2	3
3	4	4	1	0	1	0
0	0	1	0	4	4	4
0	0	0	0	3	3	3
1	4	0	0	4	4	4
1	1	4	2	3	3	3
0	1	0	0	1	4	4
0	0	0	0	3	4	2
0	0	2	0	2	3	2
3	2	0	0	4	3	3
1	0	0	0	3	1	2
0	2	2	0	4	2	3
0	0	0	0	3	4	4
0	0	0	0	1	2	4
0	0	0	0	4	4	4
0	1	1	0	4	4	4
0	0	0	0	3	4	4
1	4	0	0	4	4	4
0	0	0	0	4	4	4
0	2	0	0	0	0	0
0	0	0	0	4	4	4
2	4	3	0	4	4	4
2	4	2	0	4	3	4
0	99	2	2	4	4	3
0	0	0	0	4	4	4
2	4	0	0	4	4	4
2	3	2	0	2	2	1
0	0	0	0	4	3	4
99	99	99	99	99	99	99
0	0	0	1	2	4	3
1	1	0	0	3	3	3
0	2	0	0	3	4	3
1	0	0	0	4	4	4

4	4	0	0	4	4	4
1	3	0	0	4	4	3
0	0	0	0	4	4	4
0	1	0	0	4	4	4
0	0	0	0	4	4	4
3	0	1	0	3	3	4
0	0	0	0	4	4	4
0	1	3	0	3	3	3
3	3	3	0	3	3	2
0	0	0	0	3	3	3
2	0	0	0	2	2	1
1	2	1	0	4	4	4
0	0	0	0	4	4	4
0	1	0	1	4	4	3
0	2	0	1	2	2	2
0	99	1	0	4	4	4
2	0	0	0	4	4	4
0	0	0	0	4	4	4
1	3	0	0	4	4	4
1	1	0	0	3	2	3
0	0	0	0	4	4	4
2	0	0	0	0	1	99
0	2	1	0	3	3	2
0	0	0	0	4	4	4
2	0	0	0	2	3	1
0	2	0	0	4	4	3
2	99	0	0	1	0	0
2	1	1	0	4	4	4
0	0	0	0	3	4	3
1	2	1	0	4	4	3
0	2	4	0	1	1	2
0	0	0	0	3	3	2
0	1	0	0	4	4	3
0	0	0	1	4	3	4
0	0	0	0	99	99	4
0	1	0	0	4	4	4
2	99	1	0	2	2	2
0	0	0	0	3	3	2
0	0	0	0	4	4	4
0	0	0	0	3	4	4
0	2	1	0	1	1	0
0	99	99	99	99	99	99
1	0	3	4	4	3	4
0	1	0	0	4	4	4
0	0	0	0	4	4	4
0	0	0	0	0	1	3
0	0	0	0	3	3	3
1	0	0	0	4	99	1

4	1	1	0	4	4	4
0	0	0	0	4	4	4
0	1	2	0	4	4	4
0	0	0	0	3	4	3
0	3	2	0	3	2	2
0	0	0	0	4	4	3
0	2	0	0	4	4	4
3	4	4	4	2	3	1
0	0	0	0	3	1	4
0	2	0	0	4	4	4
0	0	0	0	4	4	4
1	99	0	0	4	4	4
0	0	0	0	4	4	4
1	1	0	0	4	4	4
0	0	0	0	0	0	0
0	0	0	0	4	4	4
0	0	0	0	4	4	4
0	0	0	0	4	4	4
0	0	0	0	4	4	4
0	0	0	0	4	4	3
0	0	0	0	4	4	3
99	0	0	0	4	2	3
0	1	0	0	4	4	4
0	2	0	0	4	3	2
99	99	1	99	99	99	99
0	4	1	0	99	3	3
0	1	0	0	4	4	4
1	3	2	0	4	4	4
0	2	2	0	4	4	4
0	0	0	0	4	4	4
3	0	0	0	4	4	4
0	0	0	0	4	4	4
0	0	0	0	4	4	4
0	0	0	0	4	4	0
0	0	0	0	2	99	4
0	0	0	0	4	4	4
0	0	0	0	2	1	2
1	0	2	0	2	1	3
0	3	3	0	3	4	4
1	2	0	0	4	4	4
4	1	0	0	3	2	3
0	0	0	0	4	4	2
0	1	0	0	4	4	4
0	0	0	0	2	4	4
3	4	2	0	4	4	4
1	0	0	0	4	4	4
0	2	0	0	4	4	4
0	1	0	0	2	4	4

0	0	0	0	4	4	4
0	2	0	0	2	3	1
0	1	1	0	2	4	2
0	2	0	0	4	4	4
4	3	99	0	2	3	2
0	0	0	0	4	4	4
1	0	0	0	4	4	4
2	0	0	0	3	2	3
0	1	1	0	4	4	4
0	2	0	0	3	4	3
0	3	1	0	4	1	4
0	0	0	0	4	4	4
0	1	1	0	2	3	1
0	1	1	0	2	4	2
99	99	99	99	99	99	99
0	0	0	0	3	3	3
0	1	1	0	4	3	3
0	0	0	0	4	4	4
0	0	0	0	4	4	4
1	1	0	0	4	4	3
2	0	0	0	4	4	4
0	0	0	0	2	4	3
0	4	0	0	0	0	0
1	3	0	4	2	4	2
4	1	2	0	3	3	2
99	99	99	99	2	2	2
2	99	1	0	4	2	3
0	1	1	0	4	4	4
0	4	0	0	4	4	4
0	0	0	0	4	4	4
0	0	0	0	4	4	4
0	1	0	0	4	4	4
0	0	0	0	3	4	3
0	1	2	0	2	4	1
0	1	0	0	4	4	99
0	0	0	0	4	4	4
0	0	0	0	2	2	3
0	0	0	0	2	2	2
4	3	2	3	2	3	2
0	0	0	0	4	4	3
0	0	0	0	4	4	4
1	1	0	0	3	4	3
0	0	0	0	4	4	4
0	1	1	0	4	4	2
0	0	0	0	4	4	4
0	0	0	0	0	0	0
0	0	0	0	3	4	3
0	0	0	0	4	4	4

0	0	0	0	4	4	4
3	99	0	0	4	4	4
0	2	0	2	4	4	4
2	2	1	0	4	4	4
0	0	0	0	4	4	4
0	0	0	0	4	4	3
0	0	0	0	0	0	0
0	0	0	0	4	4	4
0	1	0	0	4	4	4
3	1	2	0	4	4	3
0	0	0	0	3	4	4
0	0	0	0	2	1	2
1	0	0	0	1	4	0
0	4	0	1	0	4	4
0	0	0	0	4	4	4
0	2	1	1	1	1	0
0	0	0	0	4	4	4
0	0	0	0	4	4	4
2	2	0	0	4	4	4
0	0	0	0	4	4	4
1	0	1	0	1	4	3
0	1	2	0	4	4	4
2	0	0	0	0	1	0
0	0	0	0	3	3	4
0	4	1	0	4	4	3
0	0	0	0	4	4	99
99	99	99	99	4	4	4
0	1	0	0	99	4	2
0	99	1	1	4	4	4
0	0	0	0	4	4	4
99	99	99	99	99	99	99
0	0	0	0	4	4	4
0	0	0	0	4	4	4
0	0	0	0	4	4	99
0	0	0	0	4	4	4
0	0	0	0	4	4	3
0	0	0	0	4	4	4
0	0	0	0	4	4	4
0	0	0	0	0	0	0
0	1	0	0	4	4	4
0	0	0	0	4	4	4
0	0	0	0	3	4	2
0	0	1	0	4	4	4
0	0	0	0	4	3	4
0	0	0	0	3	4	4
0	0	1	0	4	4	4
0	0	0	0	4	4	4
0	0	0	0	4	4	4

99	0	0	0	4	4	4
0	1	2	0	4	4	4
0	0	0	0	4	4	4
0	0	0	0	2	3	3
0	1	3	0	2	4	2
0	0	0	0	4	4	4
0	0	0	0	4	4	99
1	3	1	0	4	4	3
0	0	0	0	4	4	4
0	0	0	0	4	4	4
0	0	0	0	4	4	4
0	2	2	0	4	4	3
0	0	0	0	4	4	4
0	0	1	0	4	4	3
0	0	0	0	4	4	4
0	0	3	0	4	4	4
2	3	4	3	2	4	4
0	1	0	0	4	4	4
1	0	1	0	3	4	4
2	0	0	0	4	4	4
4	4	4	4	1	3	2
0	0	0	0	4	4	4
0	0	0	0	4	4	4
0	0	1	0	3	4	0
0	1	0	0	4	4	3
0	0	0	0	0	0	0
4	4	4	4	4	4	4
0	0	0	0	3	4	3
0	1	0	0	99	99	99
3	1	0	0	4	4	4
4	0	0	0	2	3	2
0	0	0	0	4	3	3
0	0	0	0	4	4	4
0	0	0	0	4	4	4
0	2	0	2	4	4	4
0	0	0	0	4	4	4
0	0	0	0	0	0	0
0	0	0	0	4	4	4
0	0	0	0	4	3	3
0	0	0	0	4	4	4
2	2	2	2	0	1	0
0	0	0	0	4	4	2
0	1	0	0	4	3	3
0	1	0	0	4	4	4
0	0	0	0	3	4	4
0	1	0	1	4	4	99
0	0	0	0	4	4	4
0	1	0	0	2	2	2

0	0	0	0	4	4	4
0	0	0	0	4	4	99
0	0	0	0	3	4	2
0	0	0	0	4	4	4
0	0	0	0	4	4	4
2	1	0	0	3	4	3
0	0	0	0	4	4	4
0	1	0	1	0	1	1
0	0	0	0	4	4	4
1	2	0	0	3	3	3
1	1	0	0	4	4	4
2	3	2	2	2	3	3
0	0	2	0	4	4	4
2	0	0	1	2	4	3
0	0	0	0	2	2	2
99	99	99	99	99	99	99
0	1	1	0	3	4	4
4	1	2	0	1	1	1
1	2	4	0	2	4	1
0	1	0	0	0	4	0
0	2	1	1	3	3	3
0	0	0	0	4	4	4
0	0	0	0	4	4	4
0	0	0	0	2	3	2
0	2	4	0	4	4	4
0	0	0	0	1	2	1
1	2	0	0	4	4	4
2	0	0	0	3	2	3
1	2	0	1	4	4	4
0	0	0	0	1	4	0
0	0	0	0	3	99	4
0	0	0	0	2	4	2
0	0	0	0	99	4	0
99	99	99	99	99	99	99
0	0	0	0	4	3	4
0	0	0	0	4	4	4
1	3	1	0	4	4	4
1	0	0	0	3	2	1
0	0	0	0	4	4	3
0	2	1	1	3	1	1
1	1	0	0	4	4	4
1	2	0	0	4	4	3
0	2	0	0	4	4	4
0	1	0	0	4	4	4
0	0	0	0	99	99	99
2	1	3	1	0	1	0
2	0	0	0	4	4	4
2	4	0	0	4	4	4

2	1	0	0	2	3	1
0	0	0	0	4	2	4
0	2	1	0	2	3	2
0	0	0	0	4	4	4
0	1	0	0	1	3	0
0	0	0	0	4	4	4
0	0	0	0	4	4	4
2	3	4	0	4	3	3
1	1	0	0	4	4	3
4	4	0	0	4	4	1
0	0	3	0	3	4	4
1	1	0	0	3	4	2
0	0	0	0	4	4	4
4	4	0	0	4	4	4
0	0	99	0	4	4	4
3	4	1	0	4	4	4
0	0	0	0	4	4	3
0	0	0	0	99	4	99
0	0	0	0	4	4	4
0	0	0	0	99	4	99
0	4	0	0	3	99	1
0	0	0	0	4	4	4
0	4	0	0	4	4	4
2	0	0	0	4	4	4
0	1	1	0	2	3	2
0	2	0	0	4	3	4
0	3	1	0	4	99	4
1	0	0	0	4	4	4
0	0	0	0	0	3	0
0	4	2	1	2	3	2
0	1	0	0	4	4	4
0	0	0	0	4	3	3
0	2	0	0	4	4	4
0	1	2	0	4	4	0
0	0	0	0	2	4	3
2	1	0	0	3	4	3
0	0	0	0	4	4	4
0	0	0	0	3	1	99
0	0	0	0	0	0	0
1	4	4	0	4	4	4
2	0	0	0	4	3	2
0	1	1	0	4	4	4
0	0	0	0	4	4	4
2	2	0	0	4	4	4
1	0	0	0	3	4	3
0	2	0	0	4	4	3
0	0	0	0	3	4	3
1	2	0	0	3	2	2

0	0	0	0	4	4	4
0	0	0	0	3	3	4
0	0	0	0	4	4	4
0	0	0	0	4	4	3
0	4	4	0	3	4	3
0	2	0	0	4	3	4
0	1	0	0	4	4	4
0	4	2	0	4	4	3
0	0	0	0	4	4	4
0	2	0	0	2	4	2
0	0	0	0	4	4	4
0	0	0	0	0	0	0
99	99	99	99	99	99	99
0	2	2	0	4	4	4
0	0	0	0	4	4	4
1	1	2	1	3	4	3
0	0	2	0	3	2	2
0	0	0	0	3	3	3
2	3	0	0	4	3	4
0	0	0	0	2	4	2
0	0	1	1	4	4	4
1	1	1	1	4	4	4
2	3	1	1	4	4	4
0	1	1	0	4	4	4
1	4	0	0	4	3	2
0	1	0	0	4	4	3
99	0	0	0	0	0	0
0	4	0	0	4	4	4
0	0	0	0	2	4	3
0	1	0	0	4	4	1
4	4	4	4	4	4	4
2	0	0	0	4	4	4
0	0	1	0	4	4	2
0	2	1	0	2	1	3
1	0	0	0	4	4	4
0	0	1	0	3	4	3
0	1	0	0	1	3	4
1	1	0	0	2	2	4
0	4	0	0	4	4	4
0	0	0	0	0	4	0
1	0	1	0	4	3	2
0	0	0	0	4	4	4
1	1	0	0	4	4	4
1	0	1	0	4	4	4
0	0	0	0	4	3	3
0	0	0	0	4	4	4
0	1	0	0	4	4	4
2	0	0	0	1	3	0

0	1	0	0	4	4	2
1	0	3	0	3	4	3
1	0	0	0	1	4	4
0	0	0	0	4	4	4
0	0	0	0	4	4	4
0	0	1	0	3	4	0
0	0	0	0	2	2	2
0	0	0	0	4	4	0
0	0	0	0	4	4	4
0	0	0	0	4	4	4
0	1	0	0	4	4	4
0	2	2	0	3	4	3
0	1	0	0	2	3	2
1	4	0	0	4	4	4
0	0	0	0	4	4	4
3	2	0	1	4	4	3
0	0	0	0	0	0	0
0	1	0	0	4	3	4
0	2	0	0	3	2	3
1	4	0	0	0	2	0
0	1	0	0	4	3	2
4	4	4	4	4	4	4
1	0	0	0	4	4	4
0	0	0	0	2	4	4
0	4	0	0	1	3	1
2	1	1	0	0	1	1
0	0	1	0	4	4	3
0	0	0	0	4	4	4
0	1	0	0	3	2	3
0	0	0	0	4	4	4
1	3	3	1	4	4	4
3	2	3	0	3	2	4
1	0	0	0	4	4	99
0	2	0	0	3	2	2
0	0	0	0	4	3	3
0	1	0	0	4	4	4
0	2	0	0	4	4	4
1	1	0	0	3	3	3
0	0	1	0	4	3	2
0	0	2	0	4	3	4
0	0	0	0	4	4	4
0	0	0	0	4	4	4
0	0	0	0	2	4	4
0	2	2	0	4	4	4
2	0	0	0	3	4	3
0	0	0	0	4	4	4
0	1	1	0	4	3	3
1	0	0	0	4	4	4

0	2	0	0	3	4	3
2	0	0	0	0	1	0
0	0	0	0	4	4	4
0	0	0	0	3	2	2
2	0	0	0	3	3	2
1	0	0	0	4	4	4
0	0	0	0	3	4	4
3	1	0	0	1	3	1
1	0	0	0	4	4	3
0	0	1	0	3	4	3
0	0	0	0	4	3	4
0	1	1	0	2	2	2
1	1	0	0	4	4	4
1	1	3	0	4	4	4
0	0	0	0	4	4	4
1	4	3	0	3	4	4
99	1	0	1	2	3	2
0	0	0	0	0	0	0
3	2	2	0	4	4	4
1	0	0	0	4	4	4
0	0	0	0	2	3	2
0	0	0	0	4	3	4
0	0	0	0	2	3	2
0	0	0	0	0	0	0
1	0	0	0	2	4	4
0	0	0	0	4	4	4
0	0	0	0	2	1	2
1	2	1	0	4	4	4
0	0	0	0	3	3	3
0	0	0	0	4	4	3
1	1	0	0	4	4	3
1	0	0	0	3	4	3
0	0	0	0	3	4	4
0	0	0	0	4	4	4
4	4	1	0	4	4	4
0	1	0	0	2	3	1
0	0	0	0	0	0	0
0	1	0	0	4	4	4
2	4	2	0	4	4	2
0	0	0	0	4	4	4
0	1	1	1	4	4	4
3	2	2	0	2	2	2
2	4	3	3	0	1	2
0	0	0	1	4	4	4
0	0	0	0	4	4	4
2	1	4	0	4	4	3
0	0	0	1	4	4	4
0	0	0	0	4	4	4

0	2	2	0	4	4	4
0	0	0	0	0	2	0
0	0	0	0	4	4	3
0	0	0	0	4	4	3
99	99	99	99	99	99	99
0	2	0	0	4	4	3
0	0	0	0	4	4	4
0	0	0	0	4	4	4
2	2	0	0	4	4	4
0	0	0	0	4	4	4
2	4	0	0	4	4	4
0	1	0	0	4	4	4
2	0	0	0	4	4	4
0	1	0	0	4	4	4
99	0	0	0	0	4	0
0	4	4	3	4	4	4
0	4	0	0	2	4	99
3	2	2	0	1	0	1
1	1	0	0	4	4	4
0	0	0	0	4	4	3
0	4	0	0	2	2	1
4	0	0	0	4	4	4
0	2	1	0	4	4	4
0	0	0	0	4	4	4
4	4	0	0	4	4	2
0	0	0	0	4	4	4
0	0	0	0	4	3	4
0	1	1	0	4	4	4
1	4	0	0	3	2	3
0	0	0	0	4	4	4
0	0	0	0	4	4	4
1	1	0	0	4	4	99
0	0	0	0	4	4	4
0	0	0	0	4	4	3
1	4	2	0	2	0	1
0	0	0	0	3	1	3
0	2	0	0	2	4	2
0	1	0	0	4	4	3
1	0	1	0	3	3	4
0	0	1	0	3	3	0
0	2	0	0	4	4	4
0	0	0	0	4	4	4
0	0	0	0	4	4	4
0	0	0	0	4	4	4
0	0	0	0	3	4	3
2	4	4	0	4	4	4
0	1	0	0	4	4	3
1	0	0	0	4	4	3

0	1	0	0	4	4	4
0	2	0	0	4	4	3
0	1	0	2	4	4	4
1	2	2	0	4	4	3
0	2	2	0	4	4	4
0	4	0	0	4	4	2
0	0	0	0	4	4	4
2	3	3	4	4	4	4
1	1	3	1	0	1	1
0	0	0	0	4	4	4
0	1	0	0	4	4	4
1	0	0	0	99	99	99
0	0	0	0	4	3	3
2	2	4	0	1	2	1
0	0	0	0	4	4	4
0	1	0	0	3	4	3
0	0	0	0	4	4	4
1	99	0	0	2	4	2
0	0	0	0	4	4	4
0	0	0	0	4	4	3
0	0	0	0	4	4	4
0	0	0	0	4	4	4
0	1	0	0	2	3	2
4	4	4	3	4	4	4
0	0	0	0	4	4	4
0	1	1	0	4	4	4
2	0	0	0	2	4	2
0	0	0	0	4	4	4
1	1	2	0	3	4	1
0	0	0	0	4	4	4
0	0	0	0	4	4	4
3	4	2	4	4	4	4
0	0	0	0	3	4	3
4	0	0	0	0	1	1
0	0	2	0	4	4	0
0	1	2	0	4	4	4
0	0	0	0	4	4	4
1	4	1	0	2	3	3
0	0	0	0	3	4	3
0	0	0	0	2	2	1
0	0	0	0	4	4	4
0	0	0	0	4	4	4
0	1	0	0	4	3	4
0	0	0	0	2	4	2
0	1	0	0	4	4	3
0	2	2	0	4	4	4
0	0	0	0	1	1	1
0	0	0	0	4	4	4

99	99	0	0	4	4	3
2	3	3	0	3	3	3
0	0	0	0	2	2	2
0	0	0	0	2	3	2
99	99	99	99	99	99	99
0	0	0	0	4	4	4
0	0	0	0	4	4	4
0	0	0	0	0	0	0
0	0	0	0	2	3	3
1	0	1	0	4	4	4
0	0	0	0	4	4	3
0	99	2	0	2	1	1
0	1	0	0	4	4	3
0	1	4	0	0	4	4
0	0	0	0	0	4	1
0	0	0	0	3	3	3
0	1	0	1	0	0	0
0	4	3	0	4	4	4
4	3	3	1	0	2	1
0	0	0	0	4	4	3
0	0	0	0	99	99	99
0	2	0	0	3	4	4
0	0	0	0	4	4	4
0	0	0	0	4	4	4
0	0	0	0	4	4	4
4	3	1	0	2	4	1
0	0	0	0	4	4	4
0	0	0	0	2	4	3
0	1	0	0	4	4	4
0	0	0	0	4	4	3
1	2	1	0	3	4	4
0	1	0	0	2	3	3
2	2	4	0	3	2	2
0	1	0	0	4	4	4
0	0	0	0	2	3	2
4	0	0	0	0	2	3
1	0	2	0	4	4	4
0	0	0	0	4	4	4
1	0	0	0	3	4	4
0	1	0	0	4	4	4
1	0	0	0	0	1	0
0	2	0	0	4	3	4
0	2	0	0	3	3	3
0	0	0	0	3	3	2
0	1	0	0	2	1	4
0	2	2	3	2	1	3
2	4	2	0	1	1	1
0	0	0	0	3	4	3

2	0	0	0	1	2	2
0	1	1	0	3	4	3
0	0	0	0	3	4	4
0	1	0	0	3	4	0
0	0	0	0	4	4	4
2	2	0	0	3	4	3
0	0	0	0	4	4	4
0	1	0	0	2	2	1
0	1	0	0	4	4	4
99	4	4	4	4	3	2
4	0	0	0	4	4	2
1	1	1	0	2	1	1
4	0	0	0	4	4	4
1	0	0	0	4	4	3
0	0	1	0	4	2	2
3	1	0	0	3	2	4
1	0	0	0	3	4	2
0	1	0	0	4	4	4
1	2	1	1	4	4	4
0	99	0	0	4	4	4
0	1	1	0	4	4	4
0	0	0	0	4	4	4
0	0	0	0	4	4	4
0	0	2	0	3	4	3
0	0	0	0	4	4	3
2	4	2	1	2	3	2
0	0	0	0	4	4	4
0	0	0	0	4	4	4
0	1	0	0	4	4	3
0	2	0	0	4	4	4
0	2	0	0	3	4	2
1	0	1	0	2	4	1
3	0	0	0	3	4	3
2	2	2	0	3	3	2
0	0	1	0	3	3	2
0	1	2	0	4	4	4
0	0	4	0	2	4	2
0	2	0	0	3	4	2
2	2	2	0	4	4	4
2	2	0	0	3	2	1
0	0	0	0	4	4	4
0	0	4	4	0	2	0
99	99	99	99	1	1	1
0	3	0	0	4	4	3
0	2	0	0	4	4	4
1	0	0	0	4	4	4
0	0	0	0	3	4	3
2	3	1	2	4	4	4

2	1	2	0	3	2	2
0	0	0	0	4	4	4
2	3	3	0	4	4	3
4	4	2	0	2	3	2
0	1	1	0	3	3	3
0	2	0	0	3	4	4
0	0	0	0	4	4	4
0	0	1	0	4	4	4
0	0	0	0	4	4	4
0	1	0	0	4	4	4
0	0	0	0	0	0	0
0	3	2	0	4	4	4
2	0	0	0	1	3	1
1	1	0	0	3	3	4
0	2	0	1	2	2	2
0	1	0	1	2	2	2
0	2	2	0	3	4	2
0	1	0	0	4	4	4
0	1	0	1	4	4	4
4	2	1	2	4	1	99
0	0	0	0	3	4	3
4	2	2	0	4	4	3
0	0	0	0	3	3	3
0	4	0	0	4	4	4
2	2	2	0	4	4	4
0	0	0	0	4	2	2
0	0	0	0	2	2	2
99	99	99	99	3	4	4
2	0	0	0	3	3	3
0	0	0	0	4	4	4
99	99	99	99	4	3	3
0	0	1	0	4	4	4
0	0	0	0	3	2	3
0	0	0	0	3	3	3
3	2	0	2	2	2	4
0	0	1	0	4	4	4
1	2	2	0	4	4	4
0	0	0	0	4	4	4
99	99	99	99	4	4	4
0	0	0	0	3	4	3
0	0	0	0	4	4	4
0	0	0	0	0	0	0
0	1	1	0	3	4	3
4	0	0	0	4	4	4
0	0	0	0	3	4	4
0	0	0	0	4	4	4
0	1	0	0	4	4	4
0	0	0	0	4	4	4

2	0	0	0	4	3	3
0	4	0	0	4	4	4
1	4	4	4	2	0	4
1	1	0	0	3	4	4
3	1	0	0	2	1	2
2	3	3	0	1	4	2
0	0	0	0	4	4	4
1	1	1	1	0	0	0
0	0	0	0	2	2	2
0	0	0	0	0	0	0
3	2	2	0	3	4	2
0	2	1	0	2	4	1
0	0	1	0	4	4	4
0	1	1	0	2	2	2
0	1	2	0	4	4	3
2	0	0	0	2	3	2
1	4	2	2	0	1	0
0	1	0	0	4	4	4
0	0	0	0	3	4	4
2	0	0	0	4	4	4
0	2	2	0	4	4	4
0	1	0	0	4	4	4
0	0	0	0	4	4	3
0	0	0	0	4	3	2
0	0	0	0	2	2	2
0	0	0	0	3	4	2
0	0	0	0	4	4	4
0	1	0	0	4	4	4
0	4	4	4	2	0	99
0	0	0	0	4	4	4
0	2	0	0	4	4	4
0	0	0	0	4	4	4
2	1	4	1	2	3	2
0	0	0	0	4	4	4
0	0	0	0	3	4	3
0	4	2	0	4	4	4
1	4	0	0	4	4	3
0	0	0	0	4	4	4
1	0	2	0	2	4	4
0	0	0	0	4	4	4
4	4	1	4	4	4	4
0	0	0	0	4	4	4
0	0	2	0	2	4	3
1	2	1	3	4	3	4
0	0	0	0	3	4	3
1	2	0	0	4	4	3
2	1	0	0	4	4	4
0	1	0	0	4	4	4

0	4	4	4	0	0	0
3	1	0	0	4	4	1
0	1	0	0	4	4	4
0	1	0	0	4	4	4
0	0	0	0	4	4	4
0	0	0	0	99	4	3
0	0	0	0	0	0	0
2	4	4	0	0	1	0
4	4	4	0	1	0	4
0	0	0	0	4	4	3
0	2	2	0	4	4	4
0	0	0	0	4	4	4
0	0	0	0	3	4	3
0	0	99	0	3	3	3
1	0	0	0	4	4	4
0	0	0	0	2	1	1
0	1	2	0	4	4	4
0	0	0	0	3	2	3
2	2	4	0	3	3	3
0	0	0	0	4	4	3
4	0	0	0	4	3	2
0	2	2	0	3	4	3
2	3	2	0	3	3	3
0	1	0	0	4	4	4
0	0	0	0	4	4	3
0	0	0	1	4	4	4
0	0	0	0	4	4	4
0	0	0	0	4	4	4
0	0	0	0	4	4	3
0	0	0	0	4	4	4
0	0	1	0	4	4	4
0	0	0	0	4	4	4
0	2	1	0	3	3	2
0	0	0	0	4	4	4
0	4	0	0	4	4	4
0	0	0	0	2	3	3
0	0	0	0	4	4	4
0	0	1	0	2	2	0
1	1	0	0	4	4	4
0	1	1	1	3	3	4
0	3	2	0	1	4	2
2	3	0	0	4	4	4
0	2	0	0	0	1	0
1	1	1	0	4	4	4
0	0	0	0	1	1	1
0	0	0	0	4	4	99
0	0	1	0	3	3	1
0	1	0	0	4	4	4

0	0	0	0	4	4	4
0	0	0	0	3	3	3
0	0	0	0	4	4	4
0	0	0	0	4	4	4
0	2	1	0	4	4	4
0	0	0	0	0	0	0
0	0	0	0	0	0	0
0	0	0	0	4	4	4
0	0	0	0	4	4	4
1	0	0	0	4	4	4
0	2	1	0	2	3	3
2	2	3	0	3	4	3
0	0	0	0	3	4	3
1	1	0	0	3	4	3
0	0	0	0	3	4	3
0	0	0	0	4	4	4
2	2	2	2	4	4	4
0	2	2	0	3	4	3
99	2	3	0	1	4	4
4	2	0	0	2	0	2
0	1	0	0	2	2	2
0	0	0	0	4	4	4
2	2	0	0	4	4	4
0	0	1	0	2	4	4
0	0	0	0	2	4	4
0	1	2	0	4	4	4
2	0	1	0	4	4	3
2	1	3	3	4	4	4
0	0	0	0	4	4	2
2	0	2	0	4	4	0
99	99	2	0	4	4	4
0	0	0	0	3	4	4
0	2	0	0	4	4	4
0	0	0	0	4	4	4
1	0	1	0	3	4	2
0	0	0	0	4	4	4
0	0	0	0	4	4	4
0	0	0	0	99	99	99
1	1	0	0	3	2	3
0	0	0	0	3	3	3
0	1	0	0	4	4	4
0	0	0	0	4	4	4
0	0	0	0	4	4	4
0	0	0	0	4	4	4
4	0	1	0	4	4	3
0	0	0	0	4	4	4
1	0	0	0	4	4	4
0	0	0	0	4	4	4

0	0	0	0	4	4	3
0	0	0	0	4	4	4
0	0	0	0	4	4	4
0	1	0	0	4	4	4
0	0	0	0	4	4	4
0	0	0	0	3	4	3
0	0	1	0	4	4	4
0	1	0	0	3	4	3
0	0	1	0	2	2	0
1	1	1	1	4	3	3
4	4	4	0	4	4	4
0	0	0	0	4	4	3
99	0	0	0	4	4	99
0	0	0	0	4	4	99
0	1	0	0	4	4	4
0	1	0	0	4	4	3
1	1	1	0	4	4	4
4	1	0	0	4	4	99
0	2	1	2	3	3	4
0	0	0	0	4	2	4
0	0	0	0	4	4	4
0	0	0	0	4	4	3
2	1	1	0	3	3	3
3	0	2	0	3	4	1
0	0	0	0	0	0	0
0	0	0	0	4	4	4
0	0	0	0	4	4	4
1	0	0	0	4	4	4
0	0	0	0	4	4	4
0	0	0	0	4	4	4
0	0	0	0	4	4	4
0	0	0	0	4	4	4
0	0	1	0	4	4	4
0	0	0	0	4	4	4
0	1	0	0	4	3	4
0	0	0	0	4	4	3
0	0	0	0	3	1	0
0	0	0	0	4	4	4
99	0	0	0	3	2	3
0	0	0	0	4	4	4
0	0	0	0	4	4	4
0	0	0	0	4	4	4
2	1	0	0	4	4	4
0	1	0	0	4	4	4
3	3	1	0	2	3	2
0	0	0	0	4	4	4
0	0	0	0	4	4	3
0	0	0	0	3	4	3

0	0	0	0	4	4	4
0	0	0	0	4	4	4
99	99	99	99	4	4	4
0	0	0	0	4	4	4
0	1	1	0	4	4	4
0	1	0	0	3	4	4
0	0	0	0	4	4	4
0	1	1	1	4	4	4
0	0	0	0	4	4	3
0	0	0	0	4	4	4
2	0	0	0	1	3	1
0	0	3	0	2	3	1
0	0	0	0	2	2	0
0	0	0	0	4	4	4
0	0	0	0	4	4	4
0	0	0	0	4	4	4
0	1	1	0	4	4	4
1	0	0	0	2	4	1
1	0	0	0	4	4	4
1	1	0	0	4	4	4
0	2	0	0	4	4	4
0	0	0	0	4	4	4
0	0	0	0	4	4	4
1	1	1	1	1	4	1
0	0	0	0	0	0	0
0	0	0	0	4	4	4
1	2	0	2	4	4	4
0	0	0	0	4	4	4
0	0	0	0	4	4	4
0	0	0	0	4	4	4
1	1	0	1	3	4	4
0	0	0	0	2	2	0
0	0	0	0	3	3	3
0	0	0	0	4	4	4
1	0	0	0	4	4	4
4	4	2	0	2	3	1
0	0	0	0	4	4	4
0	0	0	0	3	4	3
0	2	0	0	4	4	4
1	0	2	0	4	4	4
1	2	2	0	4	4	4
0	0	0	0	3	3	3
1	3	2	0	2	4	2
0	0	0	0	2	2	2
0	0	0	0	4	4	4
0	0	0	0	4	4	4
1	1	1	1	4	3	3
1	2	0	0	3	3	2

0	0	0	0	4	4	4
0	0	0	0	4	4	4
0	0	0	0	4	4	4
1	3	0	0	4	4	4
0	0	0	0	0	0	0
1	1	1	1	1	4	3
1	2	0	0	4	4	4
0	0	0	0	4	4	4
1	1	0	0	4	4	4
1	1	2	0	4	4	3
0	1	0	0	4	4	4
0	0	0	0	4	3	3
0	0	0	0	4	4	3
0	0	0	0	4	4	4
0	0	0	0	4	4	4
1	2	0	0	4	4	4
0	4	3	0	2	4	3
2	0	0	0	4	4	4
0	3	1	0	4	4	4
2	4	0	0	3	4	2
0	0	0	0	4	4	4
0	0	0	0	4	4	4
0	0	0	0	4	3	3
0	0	0	0	4	4	4
0	0	0	0	4	4	4
0	2	0	0	4	4	4
0	4	3	0	4	4	4
0	0	0	0	2	4	0
1	3	2	0	4	4	4
0	0	0	0	4	4	3
0	0	0	0	4	4	3
0	1	1	0	4	4	4
1	2	0	0	0	0	0
1	0	0	0	4	4	4
0	1	0	0	2	4	4
0	1	0	0	4	4	4
0	1	0	0	4	4	4
0	0	0	0	4	4	4
0	0	0	0	3	4	3
0	0	0	0	4	4	3
0	4	0	0	4	4	4
0	0	0	0	4	4	4
0	0	0	0	3	4	3
2	0	0	0	4	4	4
0	2	0	0	4	4	4
2	0	0	1	4	4	4
1	0	0	0	4	4	4
2	0	0	0	1	2	3

0	4	3	0	4	2	4
0	0	0	0	4	4	4
1	0	1	1	4	3	3
3	2	1	0	4	4	99
0	0	0	0	3	3	99
0	0	0	0	4	4	4
0	1	0	0	4	3	4
0	0	0	0	4	4	4
0	0	0	0	4	4	4
99	0	0	0	4	4	4
0	0	2	0	2	3	1
0	0	0	0	4	4	4
0	0	1	0	4	4	3
0	0	0	0	3	4	3
0	0	0	0	2	4	4
0	1	0	0	4	4	4
0	1	0	0	4	4	4
0	1	1	0	4	4	4
2	2	3	0	3	4	3
0	1	0	0	4	4	4
4	2	1	4	1	2	0
0	2	0	0	2	2	2
0	0	0	0	4	4	4
2	1	0	0	3	4	4
1	0	0	0	1	1	1
0	0	0	0	99	99	99
0	4	3	0	4	4	4
0	1	0	0	4	4	99
0	1	2	0	4	3	3
1	0	0	0	4	4	4
0	1	1	0	4	4	4
0	0	0	0	4	4	4
1	2	2	0	2	2	2
0	0	1	0	4	4	4
0	0	0	0	4	4	3
0	0	0	0	4	4	4
0	2	0	2	4	4	4
2	1	1	0	4	4	4
0	0	0	0	3	4	4
1	0	0	0	4	4	4
1	1	1	1	3	4	2
0	0	0	0	4	4	3
0	1	0	0	4	4	4
0	1	0	0	3	2	2
0	0	0	0	1	3	2
0	0	0	0	3	3	2
0	1	1	0	4	4	4
0	0	0	0	4	3	99

0	0	0	0	4	4	4
0	1	0	0	3	3	3
0	0	0	0	4	4	4
0	0	0	0	2	3	2
0	0	0	0	4	4	3
0	0	0	0	4	4	4
0	0	0	0	3	3	2
0	0	0	0	0	2	2
2	1	1	0	4	4	4
0	0	0	0	3	2	3
1	0	0	0	4	4	4
0	2	0	0	4	4	4
0	0	0	0	4	4	4
0	0	0	0	4	4	4
0	0	0	0	3	3	4
1	0	0	0	3	4	3
0	0	0	0	0	4	0
0	1	0	0	4	4	4
0	4	0	0	4	4	4
4	4	1	1	4	4	3
1	1	0	0	1	3	2
1	0	0	0	3	4	2
0	4	0	0	4	4	4
4	4	4	0	4	4	4
0	0	1	0	3	4	3
1	2	0	1	4	4	4
0	0	0	0	3	4	3
2	2	3	1	4	2	3
0	0	0	0	1	1	0
0	0	0	0	4	4	3
1	4	1	0	0	4	4
0	0	0	0	4	4	4
0	0	0	0	4	4	4
0	0	0	0	4	4	4
0	99	1	0	4	4	3
0	2	1	0	3	3	2
0	0	0	0	4	4	2
0	0	0	0	4	4	4
0	4	0	0	4	4	4
1	2	2	0	3	3	1
0	0	0	0	4	4	4
1	0	0	0	0	1	0
0	0	0	0	4	4	4
0	2	1	0	4	4	2
1	0	0	0	2	1	2
0	0	0	0	4	4	99
0	0	1	1	3	3	3
0	3	0	0	4	4	0

0	1	1	2	2	2	2
1	0	0	0	2	2	1
0	1	0	0	4	4	4
99	99	99	99	99	99	99
0	0	0	0	4	4	4
0	0	1	0	0	4	0
0	1	1	0	4	3	3
0	2	1	0	2	3	3
0	0	0	0	2	3	4
4	2	2	2	4	2	2
0	0	0	0	99	99	99
0	1	0	0	4	4	4
99	99	99	99	1	3	99
0	99	0	0	4	4	4
0	4	2	0	3	3	3
0	0	0	0	4	4	4
0	0	0	0	4	4	4
0	3	4	0	3	2	2
0	1	0	0	2	3	2
0	0	0	0	4	4	4
0	0	0	0	4	4	4
2	0	0	0	4	4	3
0	2	2	0	2	0	2
0	2	1	1	2	4	2
0	0	0	0	2	3	2
0	0	0	0	4	4	2
2	0	2	0	2	4	3
0	0	0	0	4	4	4
0	4	1	0	2	2	1
0	0	0	0	4	4	99
0	0	0	0	3	4	4
0	0	0	0	4	4	4
0	1	1	0	4	4	4
0	0	0	0	4	4	4
0	0	0	0	2	4	2
0	2	0	0	1	1	1
0	0	0	0	4	4	4
99	1	4	0	3	2	99
0	2	0	0	3	3	2
0	0	0	0	4	4	3
0	1	0	0	3	4	2
0	0	0	0	4	4	4
0	0	0	0	4	4	4
0	0	0	0	4	4	4
1	1	0	0	3	3	3
2	1	2	2	2	3	1
0	0	0	0	4	4	3
0	2	2	0	4	4	4

99	99	99	99	99	99	99
0	0	0	0	0	0	0
0	0	0	0	3	4	3
1	0	0	0	4	4	4
2	0	0	0	4	4	4
0	0	0	0	4	3	4
0	0	0	0	4	4	4
0	0	0	0	4	3	3
0	0	0	0	4	4	4
0	0	0	0	4	4	3
0	1	0	0	3	4	3
0	0	0	0	4	4	4
0	2	0	0	2	2	2
0	2	3	0	4	4	4
99	1	1	2	3	4	0
0	2	0	0	2	3	4
99	99	99	99	99	99	99
0	0	0	0	4	3	4
0	0	0	0	4	4	3
0	1	1	0	4	4	4
0	0	0	0	4	4	4
0	1	0	0	4	4	4
3	4	1	4	4	3	4
0	0	0	0	4	4	4
0	0	0	0	4	4	4
0	0	0	0	4	3	3
99	99	99	99	99	4	3
0	0	0	0	4	4	4
0	1	0	0	4	4	4
0	0	0	0	0	0	0
0	0	0	0	4	4	4
0	1	0	0	4	4	4
0	0	0	0	2	4	0
0	2	0	0	4	4	4
1	4	0	0	2	4	3
1	0	3	0	3	4	3
0	0	0	0	3	3	3
0	0	0	0	4	4	4
0	0	0	0	4	4	4
0	0	0	0	4	4	4
3	2	2	2	4	4	3
0	1	0	1	4	4	4
0	0	0	0	99	4	99
0	0	0	0	3	4	2
0	0	0	0	1	4	0
0	0	0	0	3	3	3
0	0	0	0	4	4	4
0	2	0	0	2	2	1

4	0	0	0	4	4	4
0	1	0	0	4	4	3
0	0	0	0	4	4	3
0	0	0	0	4	4	4
0	2	2	1	4	4	4
0	0	0	1	4	4	3
0	0	0	0	4	4	4
0	0	0	0	2	4	1
0	0	0	0	1	99	0
0	99	99	0	4	4	3
0	0	0	0	4	4	4
0	0	0	0	4	4	4
3	3	0	4	0	1	1
0	0	0	0	4	4	4
0	0	0	0	4	4	3
0	0	0	0	4	4	4
0	0	2	0	4	4	3
4	4	1	4	4	4	0
0	0	0	0	0	1	0
0	0	0	0	4	4	4
0	0	0	0	4	4	3
0	0	0	0	4	4	4
0	0	0	0	4	4	4
99	99	99	99	99	99	99
0	0	0	0	3	3	3
0	1	0	0	1	2	2
1	1	0	0	4	2	3
0	1	1	0	4	4	4
0	0	0	0	4	4	4
0	0	0	0	3	4	3
1	99	0	0	1	3	99
99	99	99	99	99	99	99
0	0	0	0	2	2	3
0	1	0	0	4	4	4
0	0	0	0	4	4	4
2	4	1	0	4	4	4
0	0	0	0	4	4	4
0	0	0	0	4	4	4
0	0	0	0	4	4	3
0	0	0	0	4	3	3
0	0	0	0	4	4	4
1	2	1	0	1	2	0
0	0	0	0	4	4	4
1	4	3	0	4	4	4
0	0	0	0	4	4	4
1	1	0	0	4	4	3
0	3	0	0	2	2	2
0	1	2	0	4	4	4

0	0	0	0	3	2	1
0	1	0	0	3	4	3
0	0	0	0	4	4	4
2	0	0	0	3	4	3
0	0	0	0	4	4	4
0	0	0	0	1	1	2
0	0	0	0	4	4	4
4	4	0	0	4	4	4
2	2	2	0	3	4	3
2	2	2	0	3	4	3
0	1	0	0	2	3	2
0	0	0	0	2	4	3
0	0	0	0	2	4	2
1	4	1	2	2	1	1
0	1	1	0	4	4	99
1	0	0	0	3	4	3
4	2	2	0	3	3	2
3	2	2	2	3	4	2
0	4	2	0	4	4	3
1	2	1	3	3	4	3
0	0	0	0	3	3	3
0	2	0	0	4	4	4
0	0	0	0	2	2	2
0	0	0	0	3	3	3
0	1	0	0	2	4	3
0	0	0	0	2	4	3
0	0	0	0	1	1	1
0	1	0	0	3	3	3
2	3	0	0	4	4	4
0	0	0	0	3	4	4
2	1	1	0	4	4	4
0	4	0	0	3	2	3
0	0	0	0	4	4	3
0	1	1	0	0	1	2
0	0	0	0	4	4	4
0	0	0	0	3	3	3
0	0	0	0	3	3	2
0	0	0	0	4	4	4
0	0	0	0	4	4	4
0	0	0	0	0	2	0
1	2	0	0	2	2	1
0	0	0	0	4	4	0
0	0	0	0	4	4	4
0	0	0	0	2	2	0
0	1	0	0	0	3	2
0	0	0	0	4	4	4
0	0	0	0	4	4	4
0	0	0	0	4	4	4

0	0	0	0	3	4	99
1	2	0	0	4	2	4
99	1	0	0	4	4	4
0	0	0	0	4	4	4
0	1	0	0	2	3	1
99	1	0	0	4	4	2
0	0	0	0	2	4	0
0	1	0	0	4	4	3
0	0	0	0	4	4	2
0	0	1	0	4	4	3
2	2	1	0	3	4	2
1	2	0	0	4	4	4
3	4	3	3	4	4	4
0	0	0	0	3	4	4
0	1	0	0	3	2	2
0	0	0	0	4	4	4
2	4	0	2	3	4	2
1	4	4	0	4	3	4
0	4	0	4	4	4	4
99	99	99	99	99	99	99
0	0	1	0	4	3	3
0	1	0	0	4	4	3
1	1	2	0	1	4	3
3	2	0	0	4	4	3
0	0	0	0	4	4	3
1	2	0	0	4	4	4
0	0	0	0	4	3	4
0	2	1	0	4	4	4
0	0	0	0	4	4	4
0	4	2	0	4	4	4
1	2	0	0	2	3	4
0	0	0	0	4	3	4
2	0	1	0	4	4	4
0	1	1	0	4	4	4
0	0	0	0	4	4	4
0	2	2	0	4	4	3
1	99	0	0	4	4	4
0	2	1	0	4	4	4
1	0	0	0	4	3	3
0	1	1	0	4	3	2
0	0	0	0	4	4	4
0	0	0	0	4	4	99
0	1	0	0	4	4	4
0	0	0	0	4	4	4
0	1	0	0	4	4	4
0	4	2	0	4	4	4
2	1	0	0	2	3	2
1	4	3	1	1	2	1

0	2	0	0	4	4	4
0	0	0	0	4	4	4
0	1	0	0	0	2	1
0	0	0	0	4	4	3
1	0	1	0	4	3	2
2	0	0	0	3	4	4
1	1	1	2	3	2	3
1	2	3	1	4	4	0
0	0	0	0	4	4	4
0	2	0	0	3	4	4
3	1	0	0	4	1	1
1	2	0	0	0	2	0
0	3	0	0	4	4	0
1	2	4	2	4	4	3
0	0	0	0	4	4	4
1	2	2	0	4	4	3
0	0	0	0	4	4	4
1	1	1	0	4	4	3
0	1	0	0	4	4	4
0	0	0	0	3	4	3
0	0	0	0	4	4	4
0	0	0	0	4	4	4
1	1	1	0	4	4	4
0	0	1	0	3	4	3
0	1	0	0	4	3	3
0	0	0	0	0	0	0
0	0	0	0	3	4	3
0	0	0	0	0	4	0
0	0	0	0	2	2	2
0	0	0	0	4	3	4
0	0	0	0	4	4	4
0	0	0	0	3	4	4
1	1	0	0	2	2	2
0	0	0	0	4	4	4
0	1	2	0	3	4	4
0	4	0	0	4	4	0
1	0	0	0	4	4	4
0	2	2	0	4	4	4
0	3	0	0	2	2	2
2	0	0	0	3	3	3
0	4	4	0	4	4	4
4	4	4	4	4	4	4
1	0	0	0	4	4	4
0	1	0	0	2	4	2
0	0	0	0	2	3	1
0	1	0	0	2	3	2
0	2	0	0	2	2	2
0	0	0	0	4	4	3

2	0	0	0	4	4	4
0	0	0	0	4	4	4
0	0	0	0	4	4	4
0	0	0	0	4	4	4
0	2	1	0	4	4	4
1	1	1	0	3	3	2
0	0	0	0	4	4	4
0	0	0	0	4	4	3
3	2	0	0	4	4	4
0	1	0	0	4	4	4
0	1	0	0	0	1	3
0	0	0	0	99	4	99
0	0	0	0	4	4	4
0	0	0	0	4	4	4
3	0	0	0	4	4	4
0	0	0	0	2	99	2
0	1	0	0	4	4	3
0	1	2	0	4	4	3
0	0	0	0	4	4	3
0	1	1	1	4	4	4
1	0	0	0	4	4	4
2	1	0	0	3	4	3
0	0	0	0	4	4	4
3	0	0	0	2	4	0
0	1	0	0	4	4	4
0	1	0	0	4	4	4
0	1	2	0	3	4	3
2	1	0	0	3	4	3
0	1	1	0	4	4	4
2	3	2	1	2	4	3
2	2	2	0	4	4	4
1	1	1	1	4	4	2
0	4	0	0	4	4	4
0	0	0	0	4	4	4
3	2	3	0	3	3	3
0	1	0	0	4	4	2
99	99	0	0	99	99	99
0	2	1	0	1	4	2
0	0	0	0	4	4	4
1	0	0	0	4	3	4
0	0	0	0	4	4	99
1	0	1	0	4	4	3
0	0	0	0	4	4	3
0	3	3	0	4	4	4
0	3	1	0	4	4	4
2	3	2	1	3	3	2
0	0	0	0	4	4	4
0	0	0	0	2	3	1

1	3	4	0	3	4	3
1	1	1	0	4	4	4
0	4	2	0	2	3	2
2	2	0	0	4	4	2
1	1	0	0	4	4	4
0	1	2	0	3	4	4
0	1	0	1	4	4	4
0	0	0	0	4	4	4
0	1	0	0	2	4	3
0	0	0	0	4	4	4
0	1	0	0	4	4	4
0	0	0	0	4	4	4
2	2	2	0	4	4	4
0	1	0	0	4	4	4
0	1	0	0	3	4	3
0	0	0	0	4	4	4
0	0	0	0	3	4	3
2	0	0	0	4	4	4
0	2	0	0	2	3	3
1	1	0	1	1	2	2
0	0	0	0	4	4	4
0	1	0	0	4	3	3
0	1	1	0	4	4	4
0	1	1	0	3	4	3
0	0	0	0	0	0	0
2	3	0	0	4	4	4
2	0	0	0	3	2	2
0	0	0	0	4	4	4
3	1	0	0	4	4	4
0	0	0	0	4	4	4
0	0	0	0	3	3	3
0	0	0	0	3	4	3
0	0	0	0	3	3	3
0	3	0	0	4	4	4
0	0	0	0	4	4	4
1	0	0	0	4	4	4
0	4	0	0	4	4	3
2	2	1	0	3	4	3
0	0	0	0	4	4	4
2	0	0	0	4	4	4
0	2	2	0	4	4	4
4	1	4	0	4	4	4
0	0	0	0	4	2	3
1	0	0	0	4	4	4
99	99	0	0	3	4	3
1	99	0	1	2	4	2
0	1	0	0	4	4	4
0	2	2	0	3	3	3

0	0	0	0	4	4	4
2	1	0	0	2	4	2
0	0	0	0	3	4	2
0	0	0	0	4	4	4
0	0	0	0	4	4	4
0	0	0	0	4	4	4
4	4	4	1	2	4	1
0	0	0	0	3	4	3
0	0	0	0	3	4	4
0	1	0	1	1	1	3
0	0	0	0	4	4	4
0	0	0	0	4	4	4
0	0	4	0	4	4	4
0	3	3	1	3	4	1
1	0	0	0	4	4	2
0	0	0	0	4	4	4
0	0	0	0	2	4	0
0	2	0	0	4	4	3
0	0	0	0	4	4	4
0	1	0	0	2	2	2
0	0	0	0	3	4	1
0	0	0	0	2	3	2
4	4	4	4	3	99	99
0	1	2	1	4	4	4
1	0	0	0	4	4	4
0	0	0	0	4	4	4
1	0	0	0	4	4	0
0	0	0	0	4	4	4
0	3	0	0	4	4	4
0	0	0	0	4	3	4
1	0	0	0	4	4	4
0	0	0	0	4	4	4
0	0	0	0	4	4	4
0	1	2	0	3	2	1
0	0	0	0	4	4	4
0	0	0	0	2	4	1
0	0	0	0	4	4	3
0	0	0	0	4	4	4
0	0	0	0	3	4	3
0	0	0	0	4	4	3
0	0	0	0	4	4	4
0	0	0	0	99	99	99
0	0	0	0	2	3	2
0	0	0	0	4	4	4
0	0	0	0	4	3	3
0	0	0	0	4	4	4
2	1	0	0	4	4	4
0	0	0	0	4	4	4

0	0	1	0	4	4	4
0	2	2	0	3	4	3
1	0	0	0	4	4	3
0	0	0	0	4	4	4
1	4	1	0	2	2	2
99	99	99	99	99	99	99
0	0	0	0	4	3	4
0	1	0	0	4	4	4
0	0	1	0	4	4	4
0	0	0	0	4	4	4
0	0	0	0	4	4	4
0	0	1	0	4	4	4
0	1	0	0	4	4	99
0	0	0	0	0	0	0
0	1	0	0	4	4	4
0	0	0	0	4	4	4
0	0	0	0	4	4	4
0	0	0	2	4	4	4
0	1	0	0	4	4	4
1	3	3	2	2	3	3
0	0	0	0	3	4	4
2	3	1	0	2	99	99
0	0	1	0	4	4	4
1	2	1	0	4	4	4
0	1	1	0	4	4	4
0	0	0	0	0	0	0
0	0	0	0	0	4	0
0	0	0	0	99	99	99
1	0	1	0	4	4	3
0	2	0	0	4	4	3
1	1	2	0	4	4	4
2	0	0	0	0	2	0
0	0	0	0	4	4	4
0	0	0	0	4	4	4
0	0	1	0	4	4	4
1	2	0	0	4	4	4
99	99	99	99	99	99	99
0	2	0	0	3	3	4
0	0	0	0	4	4	4
0	1	0	0	4	4	4
0	0	0	0	3	4	3
1	0	0	0	4	4	4
0	0	0	0	4	4	4
0	1	0	0	3	4	4
0	0	0	0	4	4	4
0	0	0	0	4	4	4
0	0	0	0	1	1	1
0	0	0	0	4	4	4

0	1	0	0	4	3	3
0	1	0	0	4	4	99
0	0	0	0	3	3	3
0	0	0	0	4	4	4
0	0	0	0	4	2	3
0	0	0	0	4	4	4
1	0	2	1	4	4	4
3	2	0	0	4	4	4
0	1	0	0	4	4	4
0	0	0	0	4	4	4
0	0	0	0	0	99	0
0	0	0	0	4	4	4
2	2	0	0	4	4	3
0	0	0	0	4	4	4
0	0	0	0	4	4	4
0	0	0	0	4	4	4
0	0	0	0	4	4	4
1	1	0	0	2	1	1
1	1	1	0	3	3	3
0	0	0	0	99	4	99
0	0	0	0	4	4	4
0	0	0	0	3	3	2
99	99	99	99	4	4	4
0	1	0	0	2	3	3
2	1	2	0	3	4	2
0	2	1	1	4	4	4
0	0	0	0	4	4	4
0	0	0	0	3	4	3
0	1	1	0	4	4	4
0	0	0	0	4	4	4
0	0	0	0	3	4	3
0	0	0	0	4	4	3
0	0	0	0	4	4	4
0	0	0	0	4	3	3
0	1	1	0	4	4	3
1	1	2	0	2	99	2
0	0	0	0	4	4	4
99	99	99	99	99	99	99
0	2	0	0	4	4	3
0	1	0	0	3	4	2
2	0	0	0	4	3	2
0	0	1	1	4	4	4
0	1	0	0	2	2	2
0	0	0	0	4	4	3
0	0	0	0	3	4	4
0	0	0	0	3	4	4
0	4	0	0	2	1	0
0	2	1	0	3	4	4

99	99	99	99	4	4	99
0	1	0	0	1	0	1
1	0	0	0	4	4	4
0	0	0	0	99	4	99
0	0	0	0	4	4	4
0	1	99	0	2	4	2
0	0	0	0	4	4	4
0	1	0	0	3	2	2
0	1	0	0	3	3	2
0	1	0	0	4	4	4
0	2	2	0	4	4	4
0	1	0	0	4	4	4
0	0	0	0	4	4	4
1	0	0	0	2	1	2
0	1	0	0	4	4	4
1	0	2	0	2	4	3
0	0	0	0	4	4	4
0	1	0	0	4	4	4
4	99	1	0	4	4	4
1	2	1	0	4	4	4
0	1	0	0	4	4	3
0	2	1	0	3	4	4
99	99	99	99	4	4	99
0	0	0	0	4	2	3
1	3	3	1	1	1	0
0	0	2	0	4	4	4
99	99	99	99	99	99	99
0	0	0	0	4	4	4
3	2	2	2	2	3	2
0	0	0	0	0	0	0
0	0	0	0	3	4	2
0	99	0	0	3	4	3
0	3	0	0	0	4	0
1	4	1	0	3	3	2
1	1	0	0	4	4	4
0	0	0	0	0	0	0
0	99	0	0	4	4	4
0	0	0	0	4	4	4
0	0	0	0	3	3	3
4	4	4	4	0	1	0
0	0	0	0	4	4	4
1	0	0	0	4	3	4
2	2	0	0	4	4	3
0	0	0	0	4	4	4
3	2	1	0	4	4	3
0	0	0	0	0	2	0
99	99	99	99	99	99	99
2	1	0	0	3	2	3

0	2	0	0	2	4	1
0	0	0	0	4	4	4
0	0	0	0	0	0	0
0	0	0	0	3	4	3
0	1	0	0	4	4	4
0	0	0	0	4	4	4
0	0	0	0	4	4	4
0	1	0	0	4	4	4
2	0	0	0	4	4	99
2	2	0	0	4	4	4
0	0	0	0	3	4	3
0	0	0	0	4	4	0
0	0	0	0	4	4	4
0	0	0	0	0	4	4
0	1	0	0	4	4	4
1	1	0	0	4	4	4
0	0	0	0	4	4	4
0	0	0	0	4	4	3
0	0	0	0	4	4	4
0	0	0	0	4	4	4
0	0	0	0	3	2	1
0	0	0	0	4	4	4
0	0	0	0	2	4	4
1	0	0	0	4	4	99
0	4	0	1	4	2	3
1	1	0	0	4	4	4
0	0	0	0	4	3	3
0	0	0	0	4	4	4
0	0	0	0	4	4	4
0	0	0	0	2	2	3
0	0	0	0	3	3	3
0	0	0	0	4	4	4
0	0	0	0	4	4	4
0	1	0	0	4	4	4
2	1	0	0	4	4	4
0	0	0	0	4	4	4
99	99	1	0	4	4	3
0	0	0	0	3	3	4
99	99	99	99	99	99	99
0	0	0	0	4	4	4
0	0	0	0	4	4	4
1	2	2	0	4	4	4
0	2	0	0	3	4	3
0	0	0	0	1	3	3
0	0	0	0	1	1	1
0	0	0	0	3	4	4
0	0	0	0	4	4	4
0	1	0	0	4	4	2

0	0	0	0	4	4	4
0	2	1	1	4	4	4
0	2	0	0	4	4	4
0	0	0	0	4	4	4
0	2	2	0	4	4	4
0	0	0	0	4	4	4
0	2	1	0	4	4	4
0	4	0	0	4	4	3
0	0	0	0	4	4	3
0	0	0	0	4	4	4
4	4	4	0	1	1	0
0	1	1	0	3	4	3
0	0	0	0	4	4	4
0	0	0	0	3	3	2
1	2	0	0	2	4	4
2	1	2	0	4	4	2
0	0	0	0	4	3	4
0	1	2	0	3	4	4
0	4	0	0	4	4	4
0	0	0	0	4	4	4
4	1	2	0	0	0	0
0	2	2	0	4	4	4
0	0	0	0	3	4	2
0	0	0	0	4	4	4
0	0	0	0	4	4	4
0	0	0	0	4	4	4
99	99	99	99	99	4	99
3	3	1	0	4	4	4
2	0	0	0	3	4	3
0	0	0	0	2	4	4
3	2	0	0	3	4	4
0	1	0	0	4	4	4
0	1	0	0	4	4	4
0	0	0	0	4	4	4
0	0	0	0	3	4	2
0	0	0	0	4	4	4
3	2	0	0	4	4	4
1	0	0	0	4	4	4
1	0	0	0	4	4	3
0	1	0	0	4	4	99
2	0	0	0	4	4	4
99	99	99	99	99	99	99
0	0	0	0	4	4	4
0	0	0	0	2	3	3
0	0	0	0	4	4	4
0	0	0	0	4	4	4
1	0	0	0	4	4	4
1	3	1	0	3	2	3

1	0	0	0	4	4	4
1	2	2	0	4	4	4
0	0	0	0	4	4	4
0	0	0	0	0	2	0
0	2	0	0	4	4	3
0	0	0	0	4	4	4
0	1	0	0	0	0	3
2	2	4	0	1	0	3
0	0	0	0	4	4	4
0	0	0	0	3	4	3
3	1	1	0	1	1	2
0	0	0	0	4	4	4
0	1	1	0	3	4	3
0	0	0	0	3	4	3
0	0	0	0	2	4	2
0	0	1	0	4	4	4
0	0	1	0	4	4	3
0	0	0	0	3	4	2
0	0	0	0	2	4	2
0	4	0	0	4	4	2
0	0	1	99	4	4	3
0	2	1	1	4	4	3
1	4	1	0	4	4	3
0	0	2	1	4	4	4
0	2	2	0	4	4	4
0	1	0	0	3	4	4
0	1	0	0	3	4	2
0	2	0	1	1	0	4
0	0	0	0	4	4	4
0	0	0	0	4	4	4
0	1	0	0	3	4	4
0	0	0	0	4	4	4
0	0	0	0	4	4	3
0	1	1	1	4	4	3
0	0	0	0	4	4	4
0	0	0	0	4	4	4
1	2	0	0	4	4	4
0	1	0	0	3	4	4
0	99	99	99	3	4	3
0	1	0	0	4	4	4
0	2	0	0	3	4	2
1	4	0	0	4	4	3
99	2	0	0	4	4	4
0	4	3	1	4	4	4
0	0	0	0	4	4	4
0	1	1	0	3	2	3
0	0	0	0	4	4	4
0	1	1	0	4	4	3

0	0	0	0	4	4	4
2	3	0	0	4	4	4
0	0	0	0	4	4	4
1	99	0	0	4	4	4
0	0	0	0	4	4	3
0	0	2	0	3	4	2
0	0	0	0	4	4	4
2	2	2	0	4	3	4
2	0	0	0	4	4	4
0	0	0	0	3	4	4
0	1	0	0	3	4	4
0	99	2	0	3	4	3
0	1	0	0	4	4	4
0	2	1	0	4	4	3
0	1	0	0	4	4	4
0	0	0	0	4	4	4
0	3	2	2	4	4	4
0	0	0	0	3	3	3
99	99	99	99	0	99	99
0	0	0	0	0	0	0
0	1	0	0	4	4	3
2	3	0	0	3	4	4
0	0	0	0	2	4	2
0	0	0	0	4	4	4
0	0	0	0	4	4	4
0	1	0	0	99	1	1
0	0	0	0	3	4	3
0	1	0	0	4	4	0
1	0	0	0	2	4	3
3	4	4	4	4	4	3
0	0	0	0	0	0	0
0	1	0	0	4	4	4
0	1	0	0	4	4	3
0	0	0	0	4	4	4
0	0	0	0	4	4	4
0	1	1	0	4	4	4
0	0	0	0	4	4	4
0	0	0	0	4	4	4
0	0	0	0	99	4	0
0	0	1	0	4	4	2
0	0	0	0	4	4	4
0	0	0	0	4	4	99
1	0	0	0	3	2	3
0	0	0	0	4	4	4
0	0	2	4	4	4	3
0	0	0	0	4	4	4
0	0	0	0	4	4	4
2	1	3	0	0	0	1

0	0	0	0	4	4	4
0	1	0	0	4	4	4
0	1	0	0	4	4	4
0	0	2	0	4	4	3
0	0	0	1	3	4	4
0	0	2	0	1	1	0
0	4	2	0	4	4	4
2	0	0	0	4	4	3
2	3	0	0	4	4	4
0	0	0	0	4	3	4
99	99	99	99	99	99	99
0	2	2	0	4	4	4
0	0	0	0	4	4	4
1	1	1	0	4	4	4
0	0	0	1	4	4	3
2	1	0	0	4	4	4
0	1	0	0	4	4	4
0	0	0	0	4	4	4
0	0	0	1	4	4	3
1	4	0	0	0	1	0
1	2	0	0	4	4	3
0	1	2	0	4	4	4
0	0	0	0	4	4	4
2	2	2	2	0	1	0
0	0	0	0	4	4	4
2	2	2	0	2	2	2
0	2	0	0	4	4	4
0	4	3	0	2	3	2
1	0	0	0	4	4	4
1	1	0	0	4	4	4
0	4	4	0	3	3	3
2	4	4	0	4	4	1
0	2	1	0	3	3	3
0	1	0	0	4	4	4
0	2	2	0	4	4	4
0	0	0	0	4	4	4
0	0	0	0	4	4	4
0	0	0	0	4	4	4
0	0	0	0	4	4	4
0	1	0	0	0	4	4
0	0	0	0	4	4	3
1	2	0	0	3	3	4
0	2	0	0	0	1	4
0	0	0	0	4	4	4
0	2	2	0	2	2	2
0	3	0	0	4	4	4
3	99	0	0	4	4	4
1	3	1	0	1	2	1

0	0	0	0	4	4	4
0	1	0	0	4	4	2
1	2	2	0	4	4	4
1	4	0	0	2	2	3
0	1	3	0	4	4	4
0	0	0	0	2	3	2
0	0	0	0	3	4	3
0	0	0	0	4	4	4
0	0	0	0	0	0	0
0	1	2	0	4	4	4
1	3	4	1	3	4	4
1	0	0	0	3	4	4
0	0	0	0	4	4	4
0	0	0	0	0	0	0
1	0	0	0	4	4	99
0	4	0	0	4	4	4
0	0	0	0	0	0	0
0	0	1	0	4	3	99
0	0	0	0	4	4	0
1	0	0	0	3	4	2
1	0	0	0	0	0	0
1	1	0	0	4	4	4
0	1	0	0	4	4	3
0	99	99	99	4	4	0
0	1	1	0	3	3	2
0	2	2	1	3	4	2
0	1	1	0	4	4	4
0	0	0	0	4	4	4
0	3	0	0	4	3	3
0	0	0	0	2	3	3
0	3	0	4	4	4	4
0	0	0	0	4	4	4
1	2	0	0	4	4	4
4	0	0	0	2	3	1
2	0	0	0	4	4	3
0	3	0	1	3	3	4
1	4	0	0	1	2	1
0	0	0	0	3	4	2
4	1	0	0	3	3	3
0	0	0	0	4	4	4
0	0	0	0	4	4	4
1	1	0	0	4	4	4
0	0	0	0	4	4	4
1	3	2	0	4	3	3
0	0	0	0	3	3	3
1	1	2	0	4	4	3
0	0	0	0	4	4	4
0	0	0	0	4	4	4

0	3	1	0	4	4	4
3	1	0	0	4	4	4
0	0	0	0	4	4	4
0	0	0	0	4	4	99
0	0	0	0	4	4	4
0	0	0	0	3	4	2
1	1	0	0	3	4	4
1	0	2	2	4	4	4
2	0	0	0	4	4	4
2	0	0	0	4	4	4
0	4	4	0	4	3	4
1	0	0	0	4	4	4
0	0	0	0	4	4	4
0	0	0	0	4	4	3
0	4	0	0	2	3	2
0	1	1	0	4	4	4
2	2	0	0	2	4	3
0	0	0	0	4	4	4
2	0	0	0	4	4	4
0	0	0	0	99	99	99
0	0	0	0	4	4	4
0	0	0	0	4	4	4
2	2	2	2	3	4	3
0	0	0	0	2	4	2
1	2	1	0	4	4	4
0	0	0	0	4	4	4
0	0	0	0	4	4	4
0	0	0	0	4	4	4
0	0	2	0	4	3	3
0	0	0	0	3	4	3
99	99	0	0	4	4	4
99	1	0	1	3	2	3
0	0	0	0	3	3	3
1	0	0	0	4	4	4
0	0	0	0	4	4	4
0	0	0	0	2	3	2
0	0	0	0	2	0	0
0	0	0	0	0	0	0
0	0	0	0	4	4	4
1	1	1	0	4	4	4
0	1	0	0	4	4	4
0	2	0	0	4	4	4
0	2	0	0	4	4	4
0	0	0	0	3	3	3
0	3	1	0	4	2	1
0	0	0	0	99	4	4
0	0	0	0	4	4	99
0	1	0	0	2	2	3

0	0	0	0	4	4	3
0	0	0	0	4	4	4
0	1	0	0	0	1	0
0	0	0	0	3	3	2
0	0	0	0	4	4	4
0	0	0	0	4	4	4
0	0	0	0	4	4	4
0	2	0	2	4	4	4
0	1	0	0	4	4	4
0	1	0	0	4	4	4
0	0	0	0	2	4	1
4	99	2	0	99	99	99
0	4	0	0	4	4	4
0	0	0	0	4	4	4
0	0	1	0	99	4	0
2	2	2	0	4	4	4
0	0	0	0	4	4	4
0	1	0	0	3	4	4
0	0	1	0	4	4	4
0	0	0	0	4	4	4
0	0	0	0	4	4	4
1	0	2	0	4	4	4
0	0	0	0	3	4	4
0	0	0	0	4	4	4
0	0	0	0	3	4	3
0	1	0	1	2	4	0
0	0	1	0	1	1	0
0	0	0	0	0	99	0
0	0	0	0	1	1	0
1	3	1	3	4	4	4
1	4	1	2	4	4	4
0	0	1	0	4	3	4
0	0	2	0	4	4	4
2	2	2	0	4	4	4
0	0	0	0	4	4	4
1	0	0	0	0	2	0
0	0	1	0	1	2	3
0	2	0	0	4	4	3
0	0	0	0	3	3	2
0	0	0	0	4	4	4
0	0	0	0	3	3	3
1	0	0	0	3	4	3
3	3	3	2	4	4	4
0	0	0	0	4	4	4
0	4	4	0	3	4	3
0	0	0	0	4	4	4
0	0	0	0	4	4	4
2	2	1	0	2	2	2

0	0	0	0	4	4	3
0	4	4	0	2	2	2
0	0	0	0	4	4	4
0	0	0	0	4	4	4
0	0	0	0	4	4	4
3	3	3	0	4	4	4
1	0	1	0	4	4	4
0	4	3	0	4	4	3
1	2	4	0	2	3	2
0	2	0	0	4	4	4
1	0	1	0	3	4	4
0	0	0	0	4	4	3
1	1	1	0	4	4	3
1	2	1	0	3	4	3
0	2	1	1	4	4	4
0	0	0	0	4	4	3
0	0	0	0	4	4	4
0	2	0	0	3	3	3
0	1	0	0	4	4	3
0	0	0	0	4	4	4
0	1	0	1	4	4	3
0	2	2	0	1	4	2
99	99	0	0	4	4	1
0	0	0	0	4	4	4
0	0	0	0	4	4	4
99	1	1	0	3	3	2
1	0	2	0	4	2	3
0	0	0	0	4	4	4
0	0	0	0	4	4	4
0	0	0	0	4	4	4
0	1	0	0	4	4	3
0	0	0	0	4	4	4
0	0	0	0	4	4	4
0	0	0	0	4	4	4
1	0	0	0	4	4	4
0	0	1	0	4	4	4
0	0	0	0	0	1	0
0	1	0	0	4	4	4
0	1	0	0	4	3	4
0	0	0	0	4	4	3
0	4	2	1	2	4	1
0	0	1	0	3	4	4
1	4	3	0	4	4	2
3	2	0	0	4	4	3
0	0	0	0	4	4	4
4	1	2	0	3	3	2
0	0	0	0	4	4	4
0	0	0	0	3	3	3

1	3	2	0	3	3	1
0	0	0	0	3	4	4
4	2	1	0	3	3	2
0	0	0	0	4	4	4
0	1	0	0	4	4	4
2	3	4	3	3	4	4
0	0	0	0	4	4	4
0	0	0	0	4	4	4
0	0	0	0	4	4	4
0	2	0	0	3	4	4
0	0	0	0	4	4	4
0	0	0	0	4	4	4
0	0	0	0	4	4	4
0	0	0	0	4	4	4
0	2	0	0	3	3	2
0	0	0	0	4	4	4
0	2	0	0	3	3	3
1	3	0	0	0	4	0
0	0	0	0	99	99	99
0	0	0	0	4	4	4
1	0	0	0	4	4	4
0	4	1	0	4	4	4
0	1	0	0	4	4	4
0	0	0	0	3	4	3
0	0	2	0	0	2	0
0	2	0	0	4	2	3
0	1	0	0	1	4	1
0	1	0	0	4	4	4
0	0	0	0	4	4	4
0	0	0	0	4	4	4
0	0	0	0	4	4	4
0	0	0	0	4	4	4
1	0	0	0	4	4	4
1	3	1	0	2	2	3
0	1	3	0	4	4	3
0	0	0	0	4	4	4
2	0	0	0	4	4	4
0	0	0	0	3	3	2
2	2	2	1	4	4	4
0	0	0	0	4	4	4
0	2	0	0	4	4	4
0	1	0	0	3	3	3
1	0	1	0	4	4	4
0	0	0	0	4	4	4
1	2	0	0	3	4	3
0	1	0	2	4	4	4
2	0	0	0	3	4	2
0	0	0	0	4	4	4

0	0	0	0	2	2	2
0	1	1	0	4	4	4
0	0	0	0	4	4	4
#NULL!	#NULL!	#NULL!	#NULL!	2	4	4
#NULL!	#NULL!	#NULL!	#NULL!	4	4	4
#NULL!	#NULL!	#NULL!	#NULL!	4	4	3
#NULL!	#NULL!	#NULL!	#NULL!	4	4	4
#NULL!	#NULL!	#NULL!	#NULL!	1	1	1
#NULL!	#NULL!	#NULL!	#NULL!	2	2	1
#NULL!	#NULL!	#NULL!	#NULL!	2	2	1
#NULL!	#NULL!	#NULL!	#NULL!	4	4	3
#NULL!	#NULL!	#NULL!	#NULL!	0	2	0
#NULL!	#NULL!	#NULL!	#NULL!	4	4	4
#NULL!	#NULL!	#NULL!	#NULL!	4	4	3
#NULL!	#NULL!	#NULL!	#NULL!	3	4	3
#NULL!	#NULL!	#NULL!	#NULL!	2	4	3
#NULL!	#NULL!	#NULL!	#NULL!	2	3	2
#NULL!	#NULL!	#NULL!	#NULL!	4	4	3
#NULL!	#NULL!	#NULL!	#NULL!	4	4	4
#NULL!	#NULL!	#NULL!	#NULL!	2	4	3
#NULL!	#NULL!	#NULL!	#NULL!	4	4	4
#NULL!	#NULL!	#NULL!	#NULL!	4	4	4
#NULL!	#NULL!	#NULL!	#NULL!	3	4	4
#NULL!	#NULL!	#NULL!	#NULL!	0	0	0
#NULL!	#NULL!	#NULL!	#NULL!	4	4	4
#NULL!	#NULL!	#NULL!	#NULL!	4	4	4
#NULL!	#NULL!	#NULL!	#NULL!	4	4	4
#NULL!	#NULL!	#NULL!	#NULL!	4	4	4
#NULL!	#NULL!	#NULL!	#NULL!	4	4	4
#NULL!	#NULL!	#NULL!	#NULL!	1	2	2
#NULL!	#NULL!	#NULL!	#NULL!	3	4	3
#NULL!	#NULL!	#NULL!	#NULL!	4	4	4
#NULL!	#NULL!	#NULL!	#NULL!	4	4	4
#NULL!	#NULL!	#NULL!	#NULL!	4	3	4
#NULL!	#NULL!	#NULL!	#NULL!	2	1	2
#NULL!	#NULL!	#NULL!	#NULL!	2	2	2
#NULL!	#NULL!	#NULL!	#NULL!	4	4	4
#NULL!	#NULL!	#NULL!	#NULL!	4	4	4
#NULL!	#NULL!	#NULL!	#NULL!	1	2	1
#NULL!	#NULL!	#NULL!	#NULL!	1	2	1
#NULL!	#NULL!	#NULL!	#NULL!	0	0	0
#NULL!	#NULL!	#NULL!	#NULL!	4	4	4
#NULL!	#NULL!	#NULL!	#NULL!	4	4	4
#NULL!	#NULL!	#NULL!	#NULL!	0	4	2
#NULL!	#NULL!	#NULL!	#NULL!	4	2	4
#NULL!	#NULL!	#NULL!	#NULL!	3	4	3
#NULL!	#NULL!	#NULL!	#NULL!	3	4	2
#NULL!	#NULL!	#NULL!	#NULL!	4	4	3

#NULL!	#NULL!	#NULL!	#NULL!	4	4	4
#NULL!	#NULL!	#NULL!	#NULL!	3	4	3
#NULL!	#NULL!	#NULL!	#NULL!	2	3	2
#NULL!	#NULL!	#NULL!	#NULL!	2	3	2
#NULL!	#NULL!	#NULL!	#NULL!	3	3	3
#NULL!	#NULL!	#NULL!	#NULL!	2	4	2
#NULL!	#NULL!	#NULL!	#NULL!	2	3	3
#NULL!	#NULL!	#NULL!	#NULL!	0	0	0
#NULL!	#NULL!	#NULL!	#NULL!	0	0	0
#NULL!	#NULL!	#NULL!	#NULL!	4	4	4
#NULL!	#NULL!	#NULL!	#NULL!	4	4	0
#NULL!	#NULL!	#NULL!	#NULL!	4	1	1
#NULL!	#NULL!	#NULL!	#NULL!	#NULL!	#NULL!	#NULL!
#NULL!	#NULL!	#NULL!	#NULL!	4	4	4
#NULL!	#NULL!	#NULL!	#NULL!	2	3	3
#NULL!	#NULL!	#NULL!	#NULL!	4	4	4
#NULL!	#NULL!	#NULL!	#NULL!	4	4	4
#NULL!	#NULL!	#NULL!	#NULL!	4	3	4
#NULL!	#NULL!	#NULL!	#NULL!	4	4	4
#NULL!	#NULL!	#NULL!	#NULL!	4	4	4
#NULL!	#NULL!	#NULL!	#NULL!	3	3	3
#NULL!	#NULL!	#NULL!	#NULL!	4	4	4
#NULL!	#NULL!	#NULL!	#NULL!	0	0	0
#NULL!	#NULL!	#NULL!	#NULL!	4	4	4
#NULL!	#NULL!	#NULL!	#NULL!	4	4	4
#NULL!	#NULL!	#NULL!	#NULL!	4	4	4
#NULL!	#NULL!	#NULL!	#NULL!	2	3	2
#NULL!	#NULL!	#NULL!	#NULL!	4	4	4
#NULL!	#NULL!	#NULL!	#NULL!	3	3	3
#NULL!	#NULL!	#NULL!	#NULL!	4	3	3
#NULL!	#NULL!	#NULL!	#NULL!	3	2	3
#NULL!	#NULL!	#NULL!	#NULL!	4	4	4
#NULL!	#NULL!	#NULL!	#NULL!	4	4	4
#NULL!	#NULL!	#NULL!	#NULL!	3	3	3
#NULL!	#NULL!	#NULL!	#NULL!	4	4	4
#NULL!	#NULL!	#NULL!	#NULL!	4	4	4
#NULL!	#NULL!	#NULL!	#NULL!	4	4	4
#NULL!	#NULL!	#NULL!	#NULL!	4	4	4
#NULL!	#NULL!	#NULL!	#NULL!	4	4	4
#NULL!	#NULL!	#NULL!	#NULL!	4	4	4
#NULL!	#NULL!	#NULL!	#NULL!	4	4	4
#NULL!	#NULL!	#NULL!	#NULL!	4	4	4
#NULL!	#NULL!	#NULL!	#NULL!	4	4	4
#NULL!	#NULL!	#NULL!	#NULL!	3	3	2
#NULL!	#NULL!	#NULL!	#NULL!	4	2	4
#NULL!	#NULL!	#NULL!	#NULL!	3	3	3
#NULL!	#NULL!	#NULL!	#NULL!	4	4	4
#NULL!	#NULL!	#NULL!	#NULL!	4	4	4

#NULL!	#NULL!	#NULL!	#NULL!	4	3	4
#NULL!	#NULL!	#NULL!	#NULL!	4	4	4
#NULL!	#NULL!	#NULL!	#NULL!	4	4	4
#NULL!	#NULL!	#NULL!	#NULL!	4	4	4
#NULL!	#NULL!	#NULL!	#NULL!	4	4	4
#NULL!	#NULL!	#NULL!	#NULL!	3	4	4
#NULL!	#NULL!	#NULL!	#NULL!	4	4	4
#NULL!	#NULL!	#NULL!	#NULL!	4	4	4
#NULL!	#NULL!	#NULL!	#NULL!	4	4	4
#NULL!	#NULL!	#NULL!	#NULL!	4	4	3
#NULL!	#NULL!	#NULL!	#NULL!	4	4	4
#NULL!	#NULL!	#NULL!	#NULL!	4	4	4
#NULL!	#NULL!	#NULL!	#NULL!	4	4	4
#NULL!	#NULL!	#NULL!	#NULL!	4	4	4
#NULL!	#NULL!	#NULL!	#NULL!	2	4	2
#NULL!	#NULL!	#NULL!	#NULL!	3	4	3
#NULL!	#NULL!	#NULL!	#NULL!	#NULL!	#NULL!	#NULL!
#NULL!	#NULL!	#NULL!	#NULL!	4	4	4
#NULL!	#NULL!	#NULL!	#NULL!	4	4	4
#NULL!	#NULL!	#NULL!	#NULL!	4	4	4
#NULL!	#NULL!	#NULL!	#NULL!	3	4	3

pconnectd_pre	pconnecte_pre	sconnecta_pre	sconnectb_pre	sconnectc_pre	sconnectd_pre
4	4	2	3	3	3
99	99	99	4	4	4
0	1	1	3	1	3
4	4	3	4	3	2
4	4	3	4	4	4
99	99	99	99	99	99
4	4	99	4	4	4
0	0	2	1	2	2
99	99	99	99	99	99
3	2	3	3	3	2
99	99	3	4	2	2
4	4	3	3	3	3
4	4	3	3	3	3
1	0	3	3	3	3
4	4	99	4	4	4
0	0	2	3	3	3
4	4	4	4	3	4
3	2	3	2	3	2
4	4	3	3	3	3
1	0	4	4	4	3
99	99	99	99	99	99
4	4	1	4	4	4
4	3	3	4	4	3
4	4	4	3	3	3
4	4	3	4	3	4
4	4	3	3	3	3
4	4	3	3	3	3
2	1	2	3	4	4
4	4	3	3	4	3
4	4	2	3	3	3
4	4	4	4	4	2
3	3	2	3	3	2
4	2	3	2	3	3
4	4	3	3	3	3
99	99	4	4	4	4
3	3	1	3	1	4
3	4	3	3	3	4
3	4	4	2	3	3
4	2	3	3	3	3
3	3	3	3	3	3
4	4	3	3	3	3
4	99	2	3	3	3
4	0	2	2	2	3
4	4	3	3	3	4
4	4	2	4	4	4
4	4	3	3	3	3
3	3	3	3	1	1

1	1	3	3	1	1
4	4	4	4	3	4
4	4	2	3	2	3
4	4	2	3	2	3
4	4	3	3	3	2
2	2	2	3	2	3
4	4	3	3	3	3
4	4	3	2	2	3
4	4	3	3	1	2
4	4	4	4	3	2
3	2	3	3	3	3
4	4	3	4	4	4
0	3	3	3	4	3
2	1	2	2	2	4
3	3	4	3	99	2
4	3	3	3	1	2
4	4	3	3	2	3
4	4	4	4	3	1
1	2	2	2	3	2
4	3	3	3	99	4
2	2	3	3	99	1
4	4	2	3	3	4
3	3	1	1	1	3
2	1	4	1	3	1
4	4	4	3	3	4
4	4	4	4	3	4
4	4	4	4	4	4
2	2	4	3	2	2
99	99	99	1	1	1
4	4	3	2	2	3
4	4	4	4	4	4
4	4	2	2	3	3
4	4	3	3	3	3
3	3	2	2	2	2
4	4	3	2	2	3
0	0	3	3	3	3
1	1	3	1	1	1
4	4	2	2	3	2
4	3	3	3	2	2
4	4	4	4	4	3
3	3	3	3	3	2
4	4	3	3	3	2
4	0	3	1	1	1
4	4	3	3	1	1
4	4	2	2	2	3
4	4	3	3	3	4
4	4	3	4	99	4
2	1	3	3	3	3

3	3	4	2	2	3
2	1	2	2	3	3
3	3	3	3	3	2
4	4	3	3	3	3
4	4	3	3	3	4
4	4	3	3	3	4
4	4	3	2	1	4
4	4	3	3	3	2
4	4	3	3	3	3
1	1	3	3	2	2
4	4	3	3	3	3
3	3	3	1	1	1
2	3	2	3	2	3
3	3	3	2	3	3
4	0	2	4	2	4
1	1	3	1	3	3
3	4	4	3	4	3
4	4	3	1	1	1
4	4	3	3	3	3
2	2	3	3	3	3
4	4	3	3	3	1
4	4	3	3	1	1
0	0	4	4	4	4
4	4	2	1	1	3
4	4	4	4	4	4
2	2	3	3	3	3
3	4	3	4	1	4
4	4	3	3	3	3
4	4	3	3	2	2
4	4	3	3	2	3
99	99	99	99	99	99
4	3	1	1	1	2
99	99	99	99	99	99
4	4	3	3	3	3
4	4	3	3	3	3
4	4	3	3	3	3
4	3	3	3	3	3
2	2	3	3	3	3
3	3	3	2	3	3
4	3	2	2	2	3
4	4	3	3	2	3
4	4	2	3	2	2
4	4	3	3	3	2
2	2	3	3	3	3
4	4	3	3	3	1
99	99	99	99	99	99
2	2	3	3	3	3
3	3	3	2	1	1

4	4	3	3	1	1
4	4	3	4	2	2
4	4	3	3	2	4
4	4	3	3	2	2
4	4	3	3	2	2
4	4	3	3	2	2
0	0	3	3	3	3
4	4	3	3	3	3
4	4	2	3	4	4
3	0	2	2	1	2
4	4	4	4	4	4
0	0	4	4	4	4
3	3	2	3	2	3
4	4	2	2	3	2
4	4	3	3	3	3
4	4	3	3	3	3
4	4	3	3	2	3
1	1	4	4	4	4
3	1	4	3	4	3
99	3	3	2	3	3
1	0	4	4	3	3
4	99	3	3	3	3
4	4	4	4	1	4
4	4	3	4	3	3
4	4	2	3	3	3
4	4	2	2	2	2
4	3	2	3	3	3
4	4	3	2	3	2
4	0	2	3	4	4
4	4	3	4	4	4
0	0	4	4	4	4
4	4	3	2	3	2
4	2	3	3	3	3
4	4	3	3	4	4
4	4	4	4	4	3
4	99	3	3	2	4
4	4	3	3	4	4
0	0	3	3	3	3
4	4	4	4	3	4
4	4	3	3	3	4
4	4	3	3	3	4
4	4	3	3	3	3
4	4	3	3	3	4
3	2	1	2	1	2
4	4	3	3	2	3
4	4	1	1	1	1
4	3	3	4	3	3
4	4	4	4	4	4

3	3	3	3	4	4
4	3	3	3	3	3
4	4	1	4	4	4
3	2	3	2	2	2
3	3	3	3	4	2
3	3	4	4	1	1
4	4	3	2	3	3
2	1	3	2	1	2
0	0	99	3	3	3
2	1	1	3	4	2
4	4	3	3	3	3
4	4	4	3	1	1
3	3	3	3	3	3
4	4	2	3	2	2
3	3	4	4	99	4
4	4	4	3	3	3
4	4	3	3	3	3
1	2	3	3	4	99
4	4	3	3	3	3
2	2	3	2	3	2
4	4	4	4	4	4
4	4	4	4	4	4
0	0	2	4	4	4
2	2	3	3	3	3
2	1	4	2	1	1
1	0	2	2	2	3
1	1	3	3	2	2
2	1	4	4	3	3
0	0	1	1	1	2
4	4	3	4	4	4
4	4	4	4	4	3
4	3	3	2	2	3
4	4	4	4	4	4
2	2	3	3	2	99
4	4	2	1	2	2
2	1	3	2	3	4
2	2	3	3	3	3
2	2	3	3	3	3
2	2	2	1	2	2
3	3	3	2	3	2
1	1	2	2	2	3
99	99	3	2	1	2
4	4	4	4	4	4
4	4	3	3	3	3
3	4	3	2	1	1
1	1	3	2	1	2
4	4	3	3	3	2
4	0	4	4	4	4

4	4	3	2	3	4
4	4	2	3	2	2
2	3	3	2	1	3
3	3	4	4	4	3
4	4	3	99	4	3
4	4	3	3	4	4
4	4	2	3	4	4
4	4	2	3	1	3
4	4	2	3	1	2
4	4	2	2	3	3
4	99	99	2	3	3
4	4	3	3	3	3
3	2	3	2	2	2
2	2	3	3	2	2
4	4	4	4	3	3
4	2	2	3	1	2
0	0	4	4	4	3
4	4	3	3	2	3
99	99	2	3	3	2
4	4	4	3	4	2
4	4	3	3	2	2
3	3	1	1	1	2
4	4	3	3	2	2
3	3	3	3	3	3
3	1	2	2	2	2
4	4	4	3	3	4
4	4	4	4	4	4
2	4	2	4	4	2
4	3	3	3	3	2
4	4	2	3	3	3
3	0	3	1	1	3
4	3	2	99	3	3
3	3	3	3	3	2
4	4	3	3	3	3
4	2	99	1	99	99
4	4	4	3	4	1
4	3	2	2	2	3
4	4	3	2	2	3
4	4	3	3	3	3
4	4	3	3	3	1
0	4	2	1	1	2
3	3	3	3	3	4
0	3	2	3	2	2
3	3	3	3	3	3
4	4	3	3	3	99
1	0	4	4	3	2
4	4	2	99	3	2
4	4	4	4	4	4

4	4	3	3	3	2
4	4	4	4	4	4
3	3	3	3	3	3
99	99	99	99	99	99
4	4	4	4	3	4
4	4	3	3	3	3
3	2	3	3	2	2
4	4	2	3	3	3
4	4	3	2	3	1
4	4	2	3	3	2
4	4	4	3	3	3
3	3	3	3	3	3
3	2	3	3	3	2
4	4	3	2	3	4
4	2	4	3	2	2
4	4	3	4	3	3
4	4	3	3	2	3
4	3	4	4	3	3
4	4	4	4	3	4
4	4	2	3	4	3
4	4	3	3	4	2
2	1	2	1	1	2
4	4	2	1	1	3
1	1	3	3	3	3
4	4	3	3	3	3
2	2	3	3	3	2
4	4	3	3	2	2
3	3	2	2	2	1
4	4	4	4	99	4
0	0	3	3	3	2
4	4	3	3	3	3
2	3	2	2	3	99
4	4	2	3	2	3
2	2	2	3	3	3
4	4	3	3	3	3
3	2	4	1	1	1
3	4	2	2	2	3
3	4	4	3	3	3
4	4	4	4	4	4
4	4	4	4	3	3
4	4	4	3	3	1
4	4	1	3	2	2
2	2	3	3	3	2
3	2	3	3	2	1
4	4	3	3	3	1
3	3	4	3	3	2
2	1	3	3	3	1
4	4	3	3	2	3

4	4	1	3	99	3
4	3	3	2	2	3
4	4	99	99	99	99
1	0	2	2	3	3
3	4	3	3	3	2
99	99	99	99	99	99
1	1	1	1	1	1
3	3	3	3	1	3
4	4	4	3	2	1
0	0	4	4	4	4
4	4	4	4	1	2
4	4	4	1	1	1
2	1	3	2	3	1
4	4	3	3	2	2
4	4	2	3	3	3
4	3	3	2	2	2
2	3	3	3	2	3
4	4	4	4	3	4
4	4	3	3	3	1
3	99	3	3	3	2
4	4	3	3	2	3
2	2	2	1	3	3
4	4	3	4	4	3
2	2	2	2	2	2
2	2	3	3	3	99
4	4	3	3	3	3
4	4	4	4	4	3
99	99	4	4	4	4
4	4	3	3	3	3
4	4	3	3	3	4
4	4	3	3	2	2
3	0	4	4	4	4
4	4	4	4	4	3
4	3	2	2	3	3
4	3	2	2	2	2
4	4	3	3	4	3
4	3	2	1	3	3
4	4	3	4	4	2
3	2	3	3	3	3
4	2	3	2	3	2
4	4	4	4	4	4
4	4	3	3	3	2
3	2	3	3	4	3
4	2	2	2	2	4
4	2	4	4	4	4
4	4	4	4	4	1
4	4	3	4	4	4
4	4	3	3	3	3

4	4	3	3	3	3
4	4	3	3	3	4
4	4	3	2	3	2
1	0	3	3	3	99
4	2	1	3	3	4
4	4	4	4	3	3
4	4	4	3	4	2
0	0	4	2	1	4
4	4	3	3	3	3
4	4	4	4	4	4
4	4	2	2	1	1
2	4	4	4	4	3
3	3	4	3	3	3
4	4	3	3	3	3
4	4	3	3	2	2
2	2	3	2	1	3
0	2	3	3	3	3
4	4	2	2	1	1
3	3	3	3	3	2
4	4	3	4	4	4
3	3	3	3	3	1
4	3	3	3	1	2
2	2	2	2	2	2
2	2	3	3	3	3
3	3	3	1	1	1
2	2	3	1	1	1
3	3	3	2	2	2
4	4	4	4	3	3
3	1	3	3	99	4
4	4	3	3	3	3
4	4	3	3	3	3
4	4	3	3	3	2
4	4	2	3	3	3
4	3	3	3	3	3
0	0	1	1	1	3
4	4	3	3	4	3
4	3	4	4	2	1
2	3	3	4	4	4
4	4	3	1	1	3
4	4	4	4	2	4
4	4	3	2	2	4
2	1	3	1	2	2
4	4	4	4	4	2
99	99	99	99	99	99
2	2	2	2	2	2
2	2	2	2	3	2
4	4	3	3	3	2
4	2	3	2	99	3

4	4	3	3	2	3
3	4	1	2	3	3
4	4	3	3	4	3
4	4	2	4	4	4
4	4	3	3	3	3
4	2	3	3	3	3
4	4	3	3	2	1
2	2	2	1	2	2
3	2	3	3	3	3
3	3	3	3	2	2
0	0	3	3	3	3
4	3	3	2	3	2
4	4	4	2	1	2
4	4	2	1	1	1
2	2	3	3	2	3
4	4	3	3	3	2
4	4	3	1	1	3
4	4	4	4	1	4
4	4	4	2	4	4
2	3	3	2	2	2
4	4	1	1	1	1
99	99	99	99	99	99
3	3	3	3	3	2
4	4	4	4	3	2
2	2	2	3	2	2
3	2	2	2	1	2
1	3	2	3	2	3
4	3	2	1	1	1
3	3	2	3	3	2
4	4	3	3	3	3
1	2	2	1	1	1
3	3	3	1	1	2
4	4	3	3	2	2
4	4	4	2	1	1
99	99	4	4	1	3
4	4	4	4	4	2
2	2	2	2	2	2
2	2	3	3	3	1
4	4	3	3	3	3
3	3	3	3	3	2
0	1	3	2	3	3
99	99	99	99	99	99
4	3	3	2	3	2
4	4	3	3	3	2
4	4	4	4	4	1
3	1	4	3	1	1
3	3	3	3	2	2
4	0	4	4	4	3

4	4	3	2	3	1
4	2	3	3	3	2
4	4	3	3	3	2
3	3	1	3	3	99
0	0	4	3	1	1
4	3	3	3	2	3
4	99	99	99	3	4
3	2	2	1	1	2
0	2	4	4	3	1
4	4	4	4	1	99
4	4	3	3	1	3
4	4	4	4	1	1
4	4	3	3	2	3
4	4	4	3	3	3
0	0	1	1	1	1
4	4	4	4	4	4
4	4	3	2	3	1
4	4	2	3	3	3
4	4	3	4	4	3
4	2	4	3	2	2
4	4	3	3	1	2
2	1	3	2	1	2
4	4	3	4	4	4
2	2	4	3	3	1
99	99	99	99	99	99
3	2	3	99	2	2
3	3	2	3	2	3
4	4	2	1	2	1
4	4	3	3	4	4
4	2	3	1	3	1
4	4	3	3	3	2
4	4	3	3	3	3
2	2	3	3	2	2
4	2	3	2	1	1
2	4	4	4	99	1
4	4	3	3	3	4
1	2	3	2	3	3
2	4	3	3	4	3
4	2	1	3	2	4
4	4	3	2	1	1
3	2	3	3	2	3
3	2	2	3	3	2
4	4	3	3	1	2
4	4	3	3	3	3
4	4	2	2	3	2
4	4	3	3	3	4
4	4	2	2	2	3
4	4	3	3	3	3

4	4	3	3	3	1
2	2	4	3	3	4
3	4	99	3	99	3
4	4	3	3	3	3
1	2	3	3	3	3
4	4	3	3	3	3
4	4	3	3	3	3
2	2	1	1	1	2
4	4	3	3	3	3
4	3	2	3	3	3
1	3	3	3	2	2
4	4	3	3	3	4
3	3	4	4	4	3
3	4	2	2	1	3
99	99	99	99	99	99
3	3	3	3	3	3
3	3	3	3	3	3
4	4	3	4	99	3
4	4	3	3	3	3
4	4	3	4	4	3
4	4	4	4	3	3
4	4	3	3	4	4
0	0	1	1	1	4
0	3	4	3	2	1
2	1	2	2	1	2
99	99	1	1	2	3
2	1	3	2	3	3
4	4	3	3	2	2
4	4	3	3	3	3
4	4	3	3	3	2
4	4	3	3	3	2
4	4	4	3	1	2
2	3	3	2	1	2
2	3	3	3	3	2
4	4	3	3	2	2
4	4	4	1	1	1
3	2	3	3	2	99
3	3	2	2	2	3
3	3	3	3	1	1
4	4	3	4	4	4
4	4	3	3	1	1
3	3	3	3	3	2
3	3	3	1	2	2
4	3	3	3	2	2
3	3	3	3	99	99
0	0	4	4	4	4
3	3	99	3	3	2
4	4	3	2	1	3

4	4	3	2	1	2
4	4	1	1	1	3
4	4	3	1	1	2
4	4	3	3	2	2
4	4	3	3	3	2
4	4	3	3	4	4
0	0	4	4	4	4
4	4	3	3	2	3
4	4	3	1	3	2
4	4	1	2	3	2
4	3	4	3	1	2
3	4	2	2	3	3
2	2	4	3	2	3
4	4	3	4	4	4
4	4	3	2	3	3
0	0	4	3	3	3
4	4	3	3	3	3
4	4	3	4	4	3
4	4	3	3	2	3
4	4	4	4	4	4
4	4	3	3	3	3
4	4	3	4	4	4
0	99	4	3	4	4
3	3	2	3	3	3
4	4	3	3	3	3
4	4	4	4	4	4
4	4	99	99	99	99
4	4	3	3	3	3
4	4	3	1	1	1
4	4	4	4	4	4
99	99	99	99	99	99
4	4	2	3	3	4
4	4	3	4	3	3
4	4	3	3	4	4
4	4	3	3	3	4
3	3	3	2	3	99
4	4	2	3	4	3
4	3	4	4	4	4
0	0	4	4	4	4
4	4	3	4	4	4
4	4	3	3	4	4
4	3	4	4	3	4
4	4	3	3	3	3
3	3	4	4	4	4
4	4	3	4	4	4
4	4	4	4	4	4
4	4	4	4	4	4
4	4	4	4	4	4

4	4	3	4	4	4
4	4	3	3	3	3
4	4	4	4	4	4
4	2	3	3	3	3
2	99	3	3	3	3
4	4	3	4	4	4
99	99	3	4	4	4
4	4	3	3	3	3
4	4	4	3	4	4
4	4	3	4	3	4
4	4	2	3	3	3
4	4	3	3	3	3
4	4	2	3	4	4
3	4	3	3	2	3
4	4	3	3	3	3
4	4	2	2	3	1
4	4	1	1	1	4
4	4	3	3	2	2
4	4	3	3	3	3
4	4	4	2	2	2
2	2	1	1	1	3
4	4	4	4	3	3
4	4	3	2	3	3
4	0	4	4	3	4
4	4	2	1	3	4
0	0	3	4	4	3
4	4	1	1	1	1
4	4	1	2	3	3
99	99	3	4	4	4
4	4	3	3	2	3
2	2	4	4	3	3
3	3	3	3	3	3
4	4	4	2	2	2
4	4	3	3	3	3
4	4	3	2	2	99
4	4	3	3	1	1
0	0	2	2	2	2
4	4	4	4	4	4
4	4	4	3	4	4
4	4	3	4	3	4
0	1	99	99	3	1
4	4	3	3	3	3
3	4	3	2	1	2
4	4	2	4	4	3
4	4	4	4	4	4
99	0	3	3	3	2
4	4	2	3	3	3
2	2	3	3	3	3

4	4	3	4	3	3
4	4	4	4	4	4
3	2	3	2	3	2
4	4	3	3	3	3
4	4	4	3	1	1
4	4	3	2	3	1
4	4	3	4	3	4
2	0	4	3	3	2
4	4	3	3	3	2
3	3	3	3	2	2
4	4	3	1	1	1
2	3	4	3	3	2
4	4	3	2	4	3
3	2	3	3	3	3
2	2	3	3	3	3
99	99	99	99	99	99
4	4	3	3	3	3
1	1	2	2	2	1
3	2	2	3	2	1
4	0	3	3	3	3
3	3	1	1	1	3
4	4	3	3	3	3
4	4	3	3	3	3
3	0	2	3	3	3
4	4	3	3	3	3
0	1	4	3	99	4
4	0	3	2	2	3
4	2	2	2	2	3
4	4	3	3	3	4
4	4	2	4	4	4
3	3	4	3	3	3
2	2	3	2	2	3
99	99	2	2	3	3
99	99	99	99	99	99
4	4	3	3	3	3
4	4	3	4	3	4
4	3	2	3	3	3
2	2	2	2	2	2
4	4	3	4	4	4
1	1	3	2	2	2
4	4	3	3	3	4
4	2	3	4	3	2
3	4	2	1	2	2
4	4	4	4	4	4
99	99	99	3	3	3
1	1	2	4	4	4
4	4	4	4	4	4
3	4	2	2	3	4

2	1	3	3	3	3
2	1	3	4	4	4
3	3	2	3	2	3
4	4	4	4	4	4
1	2	3	4	4	3
4	4	3	4	4	4
4	4	3	4	4	4
4	3	3	2	3	3
4	4	3	3	3	3
0	4	4	4	4	4
4	4	3	3	4	3
3	2	3	3	2	3
4	3	2	3	3	3
4	4	2	1	2	2
3	3	3	2	2	3
4	4	4	2	4	3
4	4	3	3	3	4
4	99	3	3	3	3
4	4	3	3	2	3
4	4	99	4	3	4
2	1	3	2	4	3
3	3	3	3	3	3
4	4	1	1	1	1
4	4	4	4	4	4
3	3	3	3	3	3
3	4	2	2	3	3
3	3	2	3	3	3
4	4	4	4	3	2
2	3	3	3	2	3
1	2	2	3	1	1
4	4	3	2	2	3
3	3	3	3	3	3
4	4	99	3	99	99
4	4	4	4	4	4
4	4	4	4	4	4
4	4	4	4	3	1
4	4	3	4	4	4
2	2	3	1	1	1
0	0	3	3	3	2
4	4	2	2	2	3
2	2	3	3	99	2
4	4	3	3	3	3
4	4	3	3	4	4
4	4	3	2	3	3
4	4	4	4	3	2
2	2	4	3	99	99
4	3	3	2	2	3
1	1	3	2	3	2

4	4	3	3	2	2
4	3	3	3	2	2
4	4	3	99	2	99
4	4	3	2	2	3
4	4	2	2	2	1
2	1	3	3	2	3
4	4	3	3	3	1
4	4	3	4	3	1
4	4	3	4	2	3
3	3	3	3	2	1
4	4	3	1	2	2
0	0	4	4	4	4
99	99	99	99	99	99
4	4	4	4	4	4
4	4	4	4	4	4
4	4	3	3	3	3
1	2	2	2	2	2
3	4	3	3	2	3
4	4	3	3	2	3
3	3	3	2	2	1
4	4	2	2	2	2
4	4	3	2	2	3
4	4	2	2	2	1
4	4	3	3	2	3
3	2	3	3	4	2
4	3	3	2	1	2
0	0	4	4	4	4
4	4	2	3	3	3
3	4	4	4	4	3
0	0	2	3	3	3
4	4	1	4	4	4
4	4	3	3	3	2
4	4	3	3	3	3
4	4	3	3	2	1
4	4	4	4	4	1
3	3	3	3	3	3
3	4	2	3	2	2
4	4	2	2	1	2
4	4	2	3	1	4
0	4	3	4	3	4
3	4	2	2	3	2
4	4	3	3	3	3
4	4	3	4	3	99
4	4	4	4	4	4
3	3	3	2	1	2
4	4	3	4	3	4
4	4	3	3	4	3
4	3	3	2	3	3

4	3	3	3	2	3
4	2	2	3	3	3
4	4	2	3	3	3
4	4	3	4	3	4
4	4	3	3	3	2
1	1	3	2	2	3
2	2	99	3	2	2
4	4	4	4	4	4
4	4	3	4	4	4
4	4	3	3	2	3
4	4	3	3	3	3
3	3	3	3	3	3
3	3	3	99	3	2
4	4	3	3	3	3
4	4	3	3	3	3
4	2	3	2	2	1
0	0	3	3	3	3
4	4	3	3	2	3
3	3	3	2	99	1
3	2	4	4	2	4
3	1	3	3	4	2
4	4	4	4	4	1
2	2	2	3	2	2
3	3	3	2	3	2
2	2	2	1	1	3
4	4	4	2	1	1
3	3	3	3	3	3
4	4	3	3	3	3
2	3	3	2	2	3
4	4	4	3	3	3
4	4	1	1	1	1
4	4	3	2	2	2
4	4	99	3	2	3
2	2	3	3	3	2
3	4	3	2	2	2
4	4	3	2	2	2
4	4	1	1	1	4
2	2	1	1	3	1
3	3	2	2	1	1
4	4	3	3	4	3
4	4	4	3	3	2
3	4	3	3	3	1
4	4	3	3	3	3
4	4	3	3	2	2
4	3	4	3	2	3
4	4	3	3	3	3
1	2	4	1	1	2
4	4	4	4	4	3

4	3	4	3	4	2
0	0	1	1	1	1
4	4	2	3	3	3
2	2	3	3	1	2
3	3	2	2	1	1
4	4	4	3	1	1
4	3	3	3	2	2
2	1	3	2	1	2
4	4	4	4	4	3
4	99	3	1	3	4
3	3	3	3	2	2
2	2	2	2	1	3
4	4	2	3	2	3
4	4	3	3	3	2
4	4	3	3	3	3
4	4	3	3	2	2
3	3	2	2	2	2
0	0	4	4	4	1
4	1	3	3	2	1
4	4	3	3	3	3
2	2	3	3	2	2
4	4	3	2	2	3
4	2	3	3	3	3
0	0	2	3	3	3
3	4	99	4	1	2
4	4	4	4	4	4
0	0	4	1	4	4
4	4	2	1	1	3
3	3	3	3	2	1
4	4	4	2	3	4
4	3	3	3	4	2
4	3	3	3	3	2
4	4	3	2	2	3
4	4	3	3	3	3
4	4	2	1	1	1
3	2	3	3	3	1
0	0	3	3	3	3
4	4	2	3	2	2
4	3	4	3	4	1
4	4	3	3	3	3
4	4	3	3	2	2
2	2	99	2	99	99
3	4	4	3	2	1
4	4	3	3	2	1
4	4	4	4	3	3
3	2	2	2	3	2
4	4	3	3	3	3
4	4	2	3	3	99

4	4	3	3	3	3
1	0	3	3	3	3
3	4	3	3	2	3
3	99	3	2	3	2
99	99	99	99	99	99
3	3	2	2	1	2
4	4	3	3	3	3
4	4	3	3	3	3
4	4	3	3	4	3
4	4	3	4	3	4
4	4	3	2	2	3
4	4	3	4	4	3
4	4	3	3	3	2
4	4	3	3	3	3
1	2	2	3	3	2
4	4	4	4	4	4
4	3	3	4	4	4
1	1	1	2	1	3
3	4	2	3	3	3
4	4	4	3	3	4
2	2	2	2	3	2
4	4	3	3	3	3
4	4	2	3	3	2
4	4	3	4	4	3
4	4	4	1	3	99
4	4	3	4	3	3
4	4	4	3	4	2
4	4	4	4	3	4
2	2	3	3	1	1
4	2	3	3	3	3
4	4	3	2	3	4
99	4	3	2	3	3
4	4	3	4	4	4
3	3	3	2	2	3
1	1	3	2	1	2
1	3	4	2	3	2
2	2	3	3	2	2
4	3	3	2	1	4
2	3	3	3	3	3
0	0	3	3	3	3
4	4	3	3	4	3
4	4	3	4	4	3
4	4	3	4	4	4
4	4	3	3	4	4
4	4	3	3	3	4
4	4	2	2	2	3
4	4	4	4	3	3
3	3	2	3	4	4

4	4	4	4	4	4
4	4	3	2	3	3
4	4	4	4	4	4
4	4	3	3	2	3
4	4	3	3	3	3
4	4	3	3	4	3
4	4	3	2	3	2
4	4	3	3	3	99
1	0	4	3	3	3
4	4	3	3	3	3
4	4	3	4	4	4
99	99	3	3	1	2
4	4	3	3	3	4
2	1	2	2	3	1
4	4	2	2	3	99
4	4	3	4	1	2
4	4	3	3	2	3
3	4	2	3	2	3
4	4	3	3	3	2
3	4	3	3	3	3
4	4	3	3	2	3
4	3	4	4	4	4
2	2	3	3	2	3
4	4	1	2	1	1
4	4	4	3	2	2
4	4	99	99	2	2
2	99	2	2	2	2
4	4	2	2	2	3
3	3	3	2	3	3
4	3	3	3	3	4
4	4	3	2	3	3
4	4	3	2	2	3
3	3	3	3	2	2
0	0	1	1	2	2
4	4	3	4	4	3
4	4	4	4	4	4
4	4	3	2	3	1
3	2	3	3	3	2
3	4	3	2	1	1
2	1	2	2	2	3
4	4	3	3	3	2
3	3	3	4	3	2
4	3	4	4	4	3
4	3	3	3	3	3
4	4	3	3	3	2
4	4	2	2	2	3
0	1	2	2	4	4
4	4	3	3	4	4

3	3	3	2	1	2
3	3	4	3	3	1
0	0	4	3	4	2
2	2	3	3	3	3
99	99	99	99	99	99
4	4	3	3	3	3
4	4	1	1	3	2
0	0	2	2	2	2
3	2	2	2	2	3
4	4	3	3	3	2
4	4	3	3	3	3
2	3	3	3	1	3
4	4	3	3	3	3
4	4	1	3	3	2
2	0	4	4	3	3
2	2	2	2	2	3
0	0	3	2	3	2
4	4	3	3	3	3
2	3	4	4	4	2
3	4	4	3	2	3
99	99	4	4	4	4
3	3	4	4	3	3
4	4	4	4	3	3
4	4	2	3	2	2
4	4	2	2	3	3
2	2	2	2	2	3
4	4	3	3	99	3
3	3	4	3	3	3
4	4	4	4	4	4
4	4	3	3	3	3
4	4	2	3	2	99
3	3	2	3	2	2
3	2	2	3	4	3
4	4	3	3	3	4
2	2	1	3	3	3
2	2	3	2	2	1
4	4	3	4	3	3
4	4	3	3	3	3
4	4	3	3	2	3
4	4	3	3	2	2
3	2	3	2	4	2
3	4	3	2	2	4
4	4	3	2	1	3
1	2	2	3	3	3
4	4	4	4	1	1
2	2	2	2	1	1
1	1	4	1	1	1
3	4	3	3	3	3

3	4	4	3	4	2
4	3	3	3	2	3
4	4	2	2	3	2
2	1	3	2	3	4
4	4	4	4	4	4
3	4	4	4	4	3
4	4	1	1	1	1
2	2	2	2	3	2
4	4	3	4	3	4
1	2	3	2	1	2
1	3	3	4	3	3
0	1	3	3	4	4
4	4	4	4	4	4
4	4	3	3	3	2
1	4	3	3	3	3
3	2	4	4	1	1
3	4	4	3	1	4
4	4	4	4	4	4
4	4	3	2	2	3
4	4	3	3	4	3
4	4	4	4	3	4
4	4	3	3	3	3
3	3	3	3	3	2
3	3	3	4	4	4
3	4	4	4	1	4
2	1	4	4	3	3
4	4	3	3	2	3
4	4	3	3	3	3
4	4	3	3	3	3
4	4	3	3	4	2
4	4	3	2	1	4
3	3	4	3	4	3
3	3	3	3	3	2
2	3	3	2	3	1
3	2	3	3	2	2
4	4	2	2	3	3
3	3	4	3	3	3
4	4	3	1	2	1
4	4	3	3	2	2
1	1	2	2	1	1
4	4	3	3	3	2
4	1	4	3	4	4
1	1	1	2	1	99
4	4	3	4	4	2
4	4	3	3	3	4
4	4	2	3	1	3
4	4	4	4	3	2
4	0	3	2	1	1

2	2	3	3	2	2
4	4	3	3	3	99
4	4	2	1	2	3
1	1	3	2	2	1
2	2	3	1	3	1
3	4	4	1	1	1
4	3	3	3	2	2
4	4	4	1	4	2
4	4	4	2	3	3
4	4	3	3	4	2
0	0	1	1	1	1
4	4	3	2	3	3
1	1	3	3	3	3
4	3	3	3	3	1
2	2	3	3	3	3
2	2	3	3	3	3
4	4	4	4	4	2
4	4	3	2	2	2
4	4	2	2	2	3
2	2	3	2	1	99
4	4	3	3	2	2
4	4	2	2	2	3
3	3	2	2	2	3
4	4	3	2	2	2
3	4	3	2	1	3
2	2	3	2	1	2
2	2	3	3	3	3
4	4	4	3	2	3
3	3	2	2	2	2
4	4	3	3	2	2
3	3	3	3	3	3
4	4	4	4	4	2
3	2	4	2	2	3
1	2	3	3	2	1
1	1	1	2	1	1
4	4	3	3	2	3
4	4	2	2	1	4
4	4	3	2	2	3
3	4	2	4	4	3
3	4	3	3	1	2
4	4	3	3	3	3
0	0	3	3	3	3
4	4	3	3	2	1
4	4	3	1	2	3
4	4	2	3	2	2
4	4	3	2	2	3
4	4	3	3	3	3
4	4	4	3	3	2

3	3	4	4	4	4
3	3	3	3	3	1
0	0	1	1	1	1
4	4	2	2	2	3
1	1	3	3	2	3
3	3	2	3	3	2
4	4	3	3	3	2
0	0	99	4	99	2
2	2	3	3	2	3
0	0	4	4	4	4
2	2	2	2	2	2
4	2	3	3	1	1
2	2	2	2	1	2
1	1	4	4	3	3
3	3	2	3	2	3
2	2	2	2	3	3
0	2	3	3	3	4
4	4	4	3	3	2
4	4	3	3	3	2
4	4	3	3	2	3
4	4	3	3	4	4
4	4	2	3	3	3
2	2	3	3	3	3
3	2	3	2	3	2
2	2	2	2	3	2
3	2	1	3	2	4
4	3	3	3	3	3
4	0	1	3	3	4
0	2	1	1	1	1
4	4	4	4	4	4
4	4	4	3	3	2
4	4	3	4	3	3
1	1	1	2	2	3
4	4	3	3	2	3
4	4	2	3	2	3
4	3	3	3	3	2
3	2	2	2	2	3
4	4	3	3	3	3
4	3	3	3	3	3
4	4	3	3	2	3
4	4	4	1	1	1
4	4	4	3	2	2
4	3	1	3	3	4
3	3	4	4	3	2
4	4	4	3	4	3
3	4	1	1	1	4
4	4	4	3	3	1
4	4	3	2	3	3

1	0	2	1	3	1
4	3	2	2	1	1
4	4	2	2	2	1
4	4	3	3	4	3
3	3	3	4	3	4
4	4	99	4	3	2
0	0	3	3	3	3
1	1	2	2	1	1
2	4	99	3	3	1
3	3	3	1	1	3
4	4	3	3	3	3
4	4	3	4	4	4
3	4	3	3	99	99
3	3	3	3	3	2
4	4	99	2	99	4
0	0	3	1	1	3
4	4	3	3	3	3
3	3	3	3	3	3
3	3	3	2	1	1
4	4	3	3	1	2
3	3	4	4	3	3
2	3	3	3	2	2
3	4	2	2	3	2
4	4	2	2	2	2
4	4	3	3	3	3
4	4	3	3	3	3
4	4	3	2	1	3
4	4	4	4	4	2
4	4	3	3	3	4
4	4	2	3	2	3
4	4	4	4	4	3
4	4	4	4	4	1
3	3	2	2	3	3
4	4	3	2	3	3
3	3	1	1	2	3
3	4	2	2	2	2
4	4	4	4	4	3
2	2	3	1	1	2
4	4	2	2	1	2
4	4	3	3	2	2
4	4	1	1	1	1
4	4	2	1	1	2
1	1	1	3	1	3
4	4	4	4	4	3
1	1	3	3	3	3
4	4	3	3	3	3
2	2	2	3	99	99
4	4	3	2	1	3

4	4	4	3	3	2
3	3	4	4	4	4
4	4	2	2	3	2
4	4	3	3	1	3
4	4	3	4	3	2
0	0	4	4	4	4
0	0	1	1	1	1
4	4	4	4	4	2
4	4	3	3	2	1
4	4	3	3	3	3
3	2	2	2	3	2
4	3	3	2	2	2
3	3	1	2	1	1
4	4	3	2	1	1
4	4	3	3	2	3
4	4	3	3	3	3
4	4	4	4	4	4
4	3	3	3	3	2
2	2	3	2	1	1
1	0	4	3	3	4
2	2	3	3	3	3
4	4	4	4	3	3
4	4	4	4	4	4
4	4	3	3	3	3
4	4	3	2	2	2
4	4	2	2	3	3
4	4	3	3	2	2
4	4	4	3	3	3
0	4	1	1	1	1
4	4	1	3	3	3
4	4	3	4	4	4
3	4	3	3	3	3
4	4	2	3	3	3
4	4	3	3	3	3
4	4	3	3	4	4
4	4	3	4	4	4
4	4	3	3	3	3
99	99	99	99	3	99
2	2	3	3	3	4
3	4	4	4	4	4
4	4	3	4	4	3
4	3	4	4	3	3
4	4	4	4	4	4
4	0	4	4	4	4
2	2	3	4	4	3
4	4	4	4	4	4
4	4	4	4	4	4
4	3	3	4	4	4

4	4	2	2	3	3
4	4	3	1	4	4
4	4	3	3	4	4
4	4	3	4	4	4
3	4	3	3	3	3
4	3	3	3	3	3
4	4	3	4	4	4
4	4	2	3	3	3
2	0	3	3	3	3
4	3	3	3	3	3
4	4	4	4	4	4
3	4	3	3	4	4
99	99	99	2	4	3
4	4	3	3	3	3
4	4	3	1	3	3
4	3	3	3	3	3
4	4	3	3	3	3
4	2	99	99	99	99
3	2	3	3	4	3
2	4	4	4	4	1
4	4	3	3	4	2
4	4	3	3	3	4
3	3	3	4	4	3
3	4	3	3	4	3
0	0	2	4	4	4
4	4	3	3	2	3
4	4	4	4	4	3
4	4	3	4	4	4
4	4	4	4	3	4
4	4	3	3	2	2
4	4	4	4	4	4
4	4	3	3	3	2
4	4	3	3	3	3
4	4	3	3	3	3
4	4	4	3	3	3
4	3	4	4	4	4
0	0	4	4	4	4
4	4	3	4	2	3
1	0	4	1	4	1
4	4	4	4	4	4
4	4	3	3	3	3
4	4	4	4	4	4
4	4	3	3	3	2
4	4	3	3	3	3
2	1	3	3	1	3
1	4	3	2	3	4
4	4	2	2	2	3
3	3	3	3	1	2

4	4	3	3	4	4
4	4	4	4	4	4
4	4	4	4	3	2
4	4	4	4	4	4
4	4	3	3	3	3
4	3	3	3	3	3
4	4	4	4	4	3
4	4	3	3	3	3
4	4	4	4	4	4
4	4	3	4	2	3
2	2	2	3	4	4
2	2	3	3	4	3
0	0	3	3	3	3
4	2	4	4	4	3
4	4	3	3	3	3
3	4	3	3	3	3
4	4	3	2	3	3
3	4	3	4	2	1
4	4	3	3	2	3
4	3	3	3	3	3
4	4	2	99	99	2
4	4	4	4	4	4
4	4	4	4	4	4
4	4	2	4	3	3
0	0	4	4	4	4
4	4	3	3	3	3
4	4	3	3	3	2
4	4	3	4	3	4
4	4	4	4	4	4
4	4	3	3	3	3
3	4	3	3	2	2
0	0	3	3	3	3
3	3	3	3	3	3
4	4	2	3	4	4
4	3	2	1	1	3
4	3	99	1	3	2
4	4	4	4	4	4
4	4	3	3	3	4
4	4	3	3	3	3
4	4	3	3	3	3
4	4	2	3	3	3
3	3	3	3	3	3
3	3	3	3	3	2
2	2	3	2	2	3
4	4	3	2	2	2
4	4	4	4	4	3
3	3	3	3	2	1
2	1	2	3	3	3

4	4	3	3	3	3
4	4	3	3	2	2
4	4	3	3	4	3
3	3	1	1	1	3
0	0	2	3	3	3
3	2	3	3	3	3
4	4	4	4	4	4
4	4	3	3	3	3
4	4	3	4	4	4
2	3	4	3	99	4
4	4	2	3	1	3
3	3	3	3	1	3
4	4	4	3	2	3
4	4	4	4	3	3
4	4	4	4	4	4
4	4	4	1	1	3
4	0	2	3	4	4
4	4	4	3	4	4
4	4	4	2	2	2
2	3	4	4	3	3
4	4	3	4	3	4
3	3	3	3	3	3
4	3	3	2	2	2
4	4	4	4	4	4
4	4	2	3	3	3
4	4	3	3	3	3
4	4	3	3	3	4
1	1	2	3	3	3
4	4	3	4	3	4
4	4	4	4	3	3
2	2	2	3	3	3
4	4	3	3	3	2
0	0	3	3	3	3
4	4	4	4	3	3
4	4	3	3	3	3
4	4	3	3	4	4
4	4	1	1	1	4
4	4	3	4	1	1
4	4	2	3	4	3
4	4	4	3	3	2
4	4	4	1	1	4
4	4	3	2	3	3
4	3	4	4	3	2
4	4	3	3	3	3
3	4	4	3	4	2
4	4	3	2	1	3
4	4	4	4	2	2
4	1	3	4	1	2

2	2	3	2	99	1
4	4	1	1	1	1
4	4	3	2	3	2
4	3	3	3	3	2
99	2	4	4	3	3
4	4	4	4	2	2
3	3	3	3	3	3
4	4	2	2	2	1
4	4	3	3	99	99
4	4	3	2	1	1
2	1	1	2	2	3
4	4	4	4	4	3
3	3	3	3	3	3
4	4	3	3	3	3
4	2	3	2	2	3
4	4	4	2	3	2
4	4	3	4	4	3
4	4	4	4	4	2
4	4	2	3	3	2
4	4	3	3	2	2
0	1	4	4	4	1
1	0	4	3	2	1
4	4	3	4	3	3
3	1	4	4	3	4
1	0	2	2	1	1
99	99	99	99	99	99
4	4	2	3	2	1
4	4	3	1	1	2
3	3	3	2	2	2
4	4	3	3	3	3
4	4	3	3	3	2
4	4	2	3	3	2
2	1	3	2	3	3
4	4	3	3	3	2
4	4	2	2	2	2
4	4	4	4	4	3
4	4	3	3	3	3
2	2	2	2	2	2
4	3	3	2	3	2
4	4	3	3	3	3
3	3	2	2	2	3
4	3	3	2	3	3
4	4	1	2	3	3
3	3	3	4	3	4
3	2	3	2	1	2
3	3	3	2	2	2
4	4	3	3	3	2
3	3	3	3	3	99

4	4	3	3	3	3
3	3	3	3	3	2
4	4	3	3	2	1
4	4	3	2	2	2
4	3	4	4	3	3
4	4	4	4	4	4
3	4	4	4	4	3
2	2	3	3	3	3
4	4	3	2	1	1
2	1	3	3	3	3
4	4	4	4	3	3
4	4	3	3	2	2
4	4	3	3	99	99
4	4	2	2	3	3
3	3	3	3	99	99
3	3	3	4	3	3
1	0	2	3	3	3
4	4	3	3	3	3
4	4	4	4	4	1
3	4	3	3	2	2
3	3	2	2	3	2
4	3	3	3	4	3
4	4	3	3	3	3
3	4	4	3	1	2
3	2	3	2	2	2
4	4	4	4	2	2
3	4	3	3	1	3
2	3	3	2	99	2
0	0	2	1	4	4
4	3	3	3	3	2
4	3	1	99	99	4
4	4	3	4	3	4
4	4	3	3	3	3
4	4	2	3	3	3
4	4	3	2	3	3
3	1	3	3	2	2
3	4	4	4	4	3
4	4	3	3	3	3
4	4	3	3	1	3
2	2	3	3	3	3
4	4	3	2	2	3
3	3	4	3	3	3
4	4	3	3	3	2
4	0	3	2	3	2
99	0	3	3	3	3
4	4	1	3	3	3
3	3	2	1	2	1
4	4	3	4	4	2

2	2	3	3	3	3
2	2	3	2	2	3
4	4	4	4	3	3
99	99	99	99	99	99
4	4	3	4	4	4
0	3	2	4	4	4
3	2	3	3	2	2
4	3	4	3	2	2
4	3	3	3	3	2
4	4	4	4	3	1
99	99	99	99	1	99
4	4	3	4	3	2
3	99	1	1	1	1
4	4	1	1	1	2
3	3	2	2	2	2
4	4	3	3	3	2
4	4	3	3	1	3
3	3	3	3	2	1
2	3	4	4	4	1
4	4	2	2	2	4
4	4	2	3	1	2
4	4	3	3	2	2
0	0	3	1	1	1
2	2	3	2	99	2
4	0	2	4	3	3
4	4	3	3	3	3
4	2	3	2	2	2
4	4	4	4	4	2
2	1	3	2	2	3
4	99	3	3	3	2
4	4	3	3	3	3
4	99	3	3	2	1
4	4	3	2	1	2
4	4	3	2	1	3
4	4	2	1	1	3
1	1	4	4	4	4
4	4	3	3	3	2
2	1	3	1	1	1
3	3	3	2	2	1
4	4	3	3	2	3
3	3	2	99	2	99
4	4	4	4	4	4
4	4	4	4	1	1
4	4	4	4	3	1
3	3	3	3	3	3
3	2	3	4	2	3
4	4	3	3	3	4
3	2	3	99	2	2

99	99	99	99	99	99
0	0	4	4	4	4
3	4	3	3	2	3
4	4	3	4	3	4
4	4	3	3	1	1
3	4	2	1	2	1
4	4	3	3	1	3
4	4	2	99	1	2
4	4	3	3	3	2
3	4	1	3	4	3
4	3	2	3	99	1
4	4	4	4	4	4
2	2	3	2	2	1
4	4	3	3	2	3
2	0	1	1	1	4
4	4	3	2	1	1
99	99	99	99	99	99
4	4	2	2	1	1
3	3	3	3	3	3
3	3	3	3	3	3
4	4	3	4	3	1
4	4	3	4	4	3
3	3	3	3	3	2
4	4	4	3	3	3
4	4	2	3	3	3
3	4	3	3	2	1
4	3	3	99	99	99
4	4	3	2	2	3
4	3	3	3	3	3
0	0	1	1	1	3
4	4	3	3	3	3
4	4	3	3	3	3
1	1	2	3	3	3
4	4	3	2	2	3
4	4	3	4	4	3
4	2	3	3	3	2
3	2	3	3	4	4
4	4	4	4	4	3
4	4	4	4	4	4
4	4	4	4	3	4
2	2	4	3	2	1
4	4	4	4	4	2
4	4	3	4	3	3
2	3	3	3	3	3
3	1	2	3	3	2
4	3	4	3	3	2
4	4	4	4	4	4
99	99	4	2	3	2

4	4	3	1	1	2
4	4	3	3	3	2
4	4	3	3	3	3
4	4	4	4	4	3
4	4	3	3	3	2
4	4	3	3	3	3
4	4	3	3	3	3
4	3	3	3	1	2
1	0	2	3	3	3
4	4	1	2	1	1
4	4	3	3	3	2
4	4	3	3	3	2
1	1	4	3	3	3
4	4	3	3	4	4
4	3	4	3	2	3
4	4	4	2	1	2
4	4	3	4	4	4
4	4	2	1	2	2
0	1	2	3	2	3
4	4	4	4	4	3
4	3	3	3	3	3
4	4	1	3	4	4
4	4	2	3	2	3
99	99	99	99	99	99
3	3	3	3	3	3
2	1	2	2	2	1
2	3	1	2	4	3
4	4	3	3	3	2
4	4	4	4	4	1
4	4	3	3	4	3
0	0	2	3	3	3
99	99	99	99	99	99
2	2	2	3	3	3
4	4	2	2	3	3
4	4	4	4	4	4
4	3	3	3	3	3
4	4	3	3	3	3
4	4	3	4	3	3
4	4	3	2	2	2
3	2	3	2	3	3
4	4	3	3	3	3
2	2	4	4	3	1
4	4	3	2	3	3
4	4	2	2	2	99
4	4	3	3	2	3
4	4	1	3	3	3
2	1	4	3	4	2
4	4	3	3	3	2

0	1	4	1	1	1
4	4	4	4	3	4
4	4	4	4	3	3
2	3	3	2	2	2
4	4	4	4	4	3
1	1	3	3	2	2
4	4	4	4	4	4
4	4	4	4	1	4
99	3	3	2	1	99
2	2	3	2	2	2
3	2	1	2	1	2
4	4	3	2	2	3
4	4	3	3	3	3
0	0	2	2	3	3
4	4	3	3	3	2
4	4	3	3	2	1
3	1	2	1	1	3
3	2	3	1	1	1
4	2	3	3	3	1
4	3	3	3	2	1
3	3	4	4	3	3
4	4	2	2	2	3
1	2	3	3	3	2
3	3	3	3	3	3
4	4	2	2	1	3
3	0	3	3	2	2
1	1	3	3	3	3
4	3	4	4	4	4
4	3	3	2	2	2
4	4	2	3	3	3
4	4	3	3	2	3
3	2	4	4	4	1
4	4	3	3	3	3
3	4	2	3	4	1
4	4	3	2	1	3
3	2	3	3	1	2
3	3	3	1	1	1
4	4	3	3	2	3
4	4	4	4	4	4
0	0	3	3	3	3
2	2	2	2	2	2
4	4	3	3	3	3
4	4	4	3	3	2
2	2	3	3	3	3
2	2	3	99	3	3
4	4	4	4	4	3
4	4	4	4	4	3
4	4	3	3	4	3

99	99	3	3	2	3
1	1	2	2	3	2
4	4	3	3	2	2
4	4	4	4	4	1
0	3	3	3	3	2
2	2	3	2	3	2
2	2	3	3	2	3
4	4	3	1	1	1
4	4	3	4	4	4
4	99	3	3	3	99
4	4	3	99	99	1
4	4	3	3	3	3
4	4	1	1	2	1
4	4	4	4	4	4
2	3	3	3	2	3
3	4	1	1	4	3
2	2	4	3	3	2
4	4	3	3	3	3
4	4	99	99	99	2
99	99	99	99	99	99
3	4	3	3	2	2
4	4	3	3	3	2
3	4	3	3	3	2
4	4	3	3	3	3
4	3	3	3	3	3
4	4	3	2	3	2
3	3	3	1	2	2
4	4	3	2	2	3
4	4	3	3	3	3
3	4	3	3	3	3
4	2	3	3	2	2
4	4	3	4	4	4
4	4	3	4	4	2
4	4	3	3	3	2
4	4	4	4	4	2
4	3	3	3	1	2
4	4	3	3	3	3
4	4	2	2	2	3
4	4	2	2	2	3
2	2	2	3	2	2
4	4	4	4	4	2
4	4	2	4	4	3
4	4	3	3	3	2
4	3	4	3	3	4
4	4	3	3	3	2
4	4	3	2	3	3
2	2	4	1	1	2
99	1	2	3	1	2

3	2	2	2	3	3
4	4	2	3	3	3
0	0	2	2	2	3
4	4	3	2	1	1
3	3	3	3	2	2
4	4	3	3	3	3
3	2	2	1	1	1
3	2	2	3	3	2
4	4	4	4	4	4
4	4	3	3	2	1
2	0	4	4	1	1
0	0	2	2	1	3
4	4	2	4	2	3
4	3	4	4	4	4
4	4	4	4	4	3
3	3	3	3	99	99
4	4	3	3	3	2
4	4	3	3	2	3
4	3	3	2	3	3
3	4	2	3	99	99
4	4	3	3	4	4
4	4	3	3	3	3
4	4	4	4	4	3
4	4	2	3	3	3
3	3	2	3	1	2
0	0	3	2	2	3
3	4	3	3	3	3
0	0	2	3	3	2
2	1	3	3	3	3
4	3	2	2	2	3
4	4	2	3	2	2
4	3	3	3	3	3
2	2	3	2	2	3
4	4	3	3	3	3
4	4	4	4	4	3
2	0	4	4	4	4
3	4	4	3	3	2
4	4	2	2	1	1
2	2	1	1	1	1
3	3	3	2	1	3
4	4	2	2	2	1
4	4	1	1	1	1
4	4	4	4	4	4
3	3	3	3	3	2
2	1	2	2	1	2
2	2	4	3	3	2
2	2	3	2	2	3
4	4	3	4	1	1

4	3	2	3	1	3
4	4	3	3	3	2
4	4	2	2	3	3
4	4	3	4	3	4
4	4	3	4	3	3
3	3	2	2	2	3
4	4	3	3	3	3
4	4	3	3	3	99
4	4	4	4	4	3
4	4	4	4	3	3
4	3	4	4	2	2
3	99	99	3	2	3
4	4	4	4	4	3
4	4	3	3	3	3
4	4	3	3	3	3
4	2	2	2	3	3
4	4	4	4	3	1
4	4	2	3	2	3
4	4	3	3	4	3
4	4	4	4	3	4
4	4	4	3	3	3
3	3	4	3	3	4
4	4	4	4	4	3
99	2	99	1	1	99
4	4	4	4	4	4
3	3	3	3	2	3
4	4	2	3	3	3
2	1	3	3	3	3
4	4	3	3	3	4
2	4	3	4	4	4
4	4	3	3	3	3
4	4	3	3	2	3
4	4	3	4	4	4
4	4	2	2	3	4
3	3	2	2	2	2
2	3	3	3	1	3
99	99	99	99	99	99
4	3	3	3	3	3
4	4	3	3	2	3
3	3	4	4	2	2
4	4	3	4	4	4
4	4	4	4	4	4
3	3	3	3	3	3
4	4	4	4	4	3
4	4	4	4	4	4
4	4	3	3	2	3
4	4	3	3	4	3
3	2	1	2	3	3

3	4	2	2	1	1
4	4	3	3	3	3
2	3	2	2	1	1
2	4	2	2	2	1
4	4	2	2	3	3
4	4	3	4	4	3
4	4	3	3	3	3
4	4	4	3	3	2
4	3	4	4	2	3
4	4	3	3	3	2
4	4	3	3	3	4
4	4	3	2	3	2
4	4	2	2	2	2
4	4	4	4	4	3
2	3	3	3	2	2
4	4	3	3	3	3
4	3	2	4	4	4
4	4	3	3	3	3
4	3	3	3	2	3
2	2	3	3	2	2
4	4	2	3	3	3
4	4	4	4	2	2
4	4	4	2	3	2
4	4	4	2	4	3
0	0	1	1	1	1
3	4	3	3	3	2
2	2	3	3	4	4
4	4	4	4	3	3
4	4	3	3	3	3
4	4	3	3	3	3
3	3	3	3	3	1
4	4	2	3	2	2
3	3	3	4	3	3
4	4	2	2	2	2
4	4	3	3	3	2
4	4	3	3	3	2
4	3	2	2	1	2
3	2	3	2	3	3
4	4	3	3	3	3
4	4	4	3	3	3
4	4	3	2	3	3
4	4	2	1	1	1
4	4	3	2	2	3
4	4	3	3	3	3
3	2	2	2	3	1
99	2	3	1	2	2
4	4	3	4	3	3
3	3	3	3	3	4

4	4	4	4	4	3
2	2	3	2	2	2
3	2	4	3	4	2
4	4	2	3	2	3
4	4	4	4	4	4
4	4	3	3	4	4
4	4	4	3	3	2
3	3	3	3	3	3
4	4	4	4	4	4
4	4	3	2	3	3
4	4	4	4	4	4
4	4	4	3	4	4
4	1	4	4	4	4
4	3	2	3	2	3
4	4	4	4	4	4
4	3	3	4	4	4
4	3	4	4	4	4
4	4	4	3	4	4
4	4	3	3	3	3
1	1	4	3	4	4
3	0	2	3	4	3
4	3	3	3	3	4
1	0	1	1	99	3
4	4	2	3	3	3
4	4	3	4	4	4
4	4	4	4	4	4
4	4	4	4	4	4
4	4	3	3	4	4
4	4	3	3	3	3
4	3	3	3	2	3
4	4	4	4	4	4
4	4	3	4	3	4
4	4	2	2	3	3
1	1	2	3	2	3
4	4	4	3	4	2
1	3	4	4	4	3
3	4	3	2	1	2
4	4	3	2	2	3
4	4	4	4	4	3
4	4	3	3	3	3
4	4	4	4	3	3
99	99	4	4	4	4
2	3	3	3	3	3
4	4	4	4	4	4
3	4	3	3	3	4
4	4	4	3	3	3
4	4	3	3	3	3
4	4	3	3	3	3

4	4	3	4	3	2
4	4	2	2	3	2
4	4	3	3	3	3
4	4	4	4	4	4
1	1	3	3	3	3
99	99	99	99	99	99
4	4	3	3	3	3
4	4	3	3	4	4
4	4	3	3	3	3
4	4	4	4	4	4
4	4	3	3	4	4
4	4	3	3	3	2
4	99	3	3	3	3
0	0	2	2	3	3
4	4	4	4	4	4
4	4	3	3	3	2
4	4	3	3	3	3
4	4	4	4	4	4
4	4	3	3	3	3
3	4	3	2	2	2
4	4	3	3	3	3
99	99	3	3	3	3
4	4	3	3	3	3
3	3	4	3	3	3
4	4	3	3	3	4
0	0	2	2	2	2
3	0	2	3	3	3
99	99	99	99	99	99
4	3	2	2	3	3
4	3	3	2	3	3
4	4	3	3	3	2
2	0	4	4	3	2
4	4	2	1	1	2
4	4	3	3	3	3
4	4	3	3	4	3
4	4	3	4	3	3
99	99	99	99	99	99
3	2	3	3	3	3
4	4	99	3	3	3
4	4	3	3	2	3
3	3	3	2	2	2
4	4	3	3	4	4
4	4	3	4	4	4
4	4	2	3	3	3
4	4	3	3	3	3
4	4	4	4	4	4
3	4	3	4	4	3
4	4	2	3	3	4

3	3	3	3	99	4
99	4	2	3	4	3
3	3	3	3	4	4
4	99	3	3	3	3
3	2	3	3	3	4
4	4	3	4	3	3
4	4	3	4	3	3
4	4	3	4	3	2
4	4	3	3	3	3
4	4	1	1	1	1
4	99	3	4	4	4
4	4	4	4	4	4
4	4	4	4	4	2
4	4	3	2	2	3
4	4	3	3	3	3
4	4	1	1	1	1
4	3	4	4	4	4
0	1	4	3	3	2
4	3	3	3	4	3
4	4	2	4	4	4
4	4	3	4	4	3
3	2	4	3	3	2
4	4	2	99	99	99
3	3	3	2	3	3
4	4	3	1	3	3
4	4	3	3	3	2
4	4	4	4	4	4
4	4	2	3	3	3
4	4	3	3	3	3
4	4	3	3	3	3
3	3	3	3	3	3
4	4	3	3	3	3
4	4	4	4	4	4
2	3	3	3	4	3
4	3	3	3	3	3
99	2	3	3	3	2
4	4	3	3	3	3
99	99	99	99	99	99
3	3	3	3	3	3
3	4	3	3	4	1
3	2	3	3	3	2
4	99	3	2	2	1
2	2	3	4	2	3
4	3	3	3	3	3
3	2	3	2	1	2
4	3	3	3	3	3
0	0	4	4	4	3
4	3	4	4	3	2

99	3	99	99	99	99
1	3	3	3	3	3
4	4	4	4	4	4
99	99	99	99	4	4
4	4	4	4	3	3
4	2	3	3	3	2
4	4	3	3	4	4
3	2	2	3	3	3
3	2	2	3	3	3
4	4	4	4	3	3
3	3	3	3	3	3
4	4	3	3	3	3
4	4	4	4	3	3
2	1	3	3	3	2
3	4	3	3	2	3
3	3	3	3	2	2
4	4	4	4	4	4
4	2	4	2	2	3
4	4	2	2	2	1
4	4	3	2	3	3
4	4	3	3	4	3
4	4	4	3	2	3
99	99	99	99	99	99
4	3	3	3	4	3
0	0	3	3	2	3
4	4	3	3	3	3
99	99	99	99	99	99
4	4	3	3	2	3
3	1	2	2	3	3
0	0	2	3	3	3
2	3	4	4	4	2
3	4	4	4	3	4
2	3	4	4	4	4
2	2	2	2	2	99
4	4	3	4	4	4
0	0	4	4	4	4
3	3	4	3	4	3
4	4	2	3	3	3
3	3	4	1	4	3
0	4	3	1	1	1
4	4	3	3	4	4
4	3	3	3	3	3
4	4	3	2	4	3
4	4	4	4	4	4
3	3	3	3	3	3
0	0	4	4	4	4
99	99	99	99	99	99
4	3	2	2	1	3

4	2	2	3	4	3
4	4	4	4	4	2
0	0	2	3	3	3
4	3	3	4	3	4
4	4	3	4	3	4
4	4	4	4	4	4
4	4	4	4	4	4
4	4	3	3	4	2
4	4	2	3	3	2
4	4	3	4	3	4
4	4	3	3	3	4
4	99	2	3	3	3
4	4	3	3	4	4
4	0	4	4	4	2
4	4	3	3	3	3
4	4	3	3	99	3
4	4	4	4	3	2
3	3	3	3	3	2
4	4	3	3	3	2
4	4	4	4	4	1
2	3	3	2	1	2
4	4	3	3	3	3
4	4	3	2	2	2
3	4	2	99	2	3
3	3	3	2	3	4
4	4	3	2	1	2
3	4	3	4	3	3
4	4	2	3	4	4
4	4	3	3	3	3
2	2	2	3	2	3
4	4	3	3	3	3
4	2	3	4	4	1
4	4	3	3	3	3
4	4	4	3	3	2
4	4	3	4	4	3
4	3	3	4	4	4
4	3	3	4	4	4
3	3	4	4	4	4
99	99	99	99	99	2
4	4	3	3	3	3
4	4	4	4	4	4
4	4	3	4	4	3
4	3	3	3	2	3
3	3	3	3	3	3
1	1	3	3	2	2
4	4	2	3	3	3
4	4	4	4	3	3
4	3	3	2	1	2

4	3	3	3	3	2
4	4	2	3	3	2
4	4	3	3	3	3
4	4	4	3	3	3
4	4	4	4	4	4
4	4	4	3	3	3
4	4	3	3	3	2
3	3	99	2	2	99
3	3	3	3	3	2
4	4	3	2	3	1
1	1	1	1	1	2
2	2	4	3	4	4
4	4	3	2	2	2
3	4	2	3	3	3
4	4	3	3	3	2
3	4	2	1	1	1
4	4	4	4	4	3
4	3	2	3	2	2
3	4	3	4	4	2
4	4	3	3	3	3
0	0	3	2	2	3
4	4	3	3	3	3
2	3	3	3	3	4
4	4	3	2	3	3
4	4	3	3	4	4
4	4	3	3	3	3
99	99	3	3	3	3
4	4	2	3	3	3
4	2	2	3	99	2
4	4	3	2	2	2
3	4	3	4	4	1
4	4	3	3	2	4
4	4	3	3	3	3
4	4	3	3	2	2
2	2	3	3	3	3
4	4	3	3	3	3
4	4	3	3	3	3
4	3	3	3	3	4
4	3	3	3	2	2
4	4	4	3	4	2
4	4	3	3	3	4
99	99	99	99	99	99
4	4	4	4	4	4
4	3	2	3	3	3
4	4	3	3	3	3
4	4	4	4	4	4
4	4	3	4	2	3
2	2	3	3	2	2

4	4	4	4	4	2
4	3	3	3	3	2
4	4	4	4	3	4
4	1	3	3	3	3
4	3	3	3	3	3
4	4	3	3	3	3
4	1	2	2	3	2
0	0	1	1	1	3
4	4	4	3	4	3
4	3	3	3	3	3
1	2	2	2	3	2
4	4	3	3	2	3
4	4	3	4	4	4
4	2	2	3	3	1
4	4	3	3	3	3
4	4	3	3	4	4
4	4	3	3	3	4
3	4	99	99	3	3
4	4	2	2	2	3
4	4	3	3	3	3
4	4	4	3	2	3
3	3	3	3	4	4
4	4	2	1	1	3
4	4	4	4	4	3
4	4	3	4	4	3
4	4	3	3	3	3
3	3	4	3	3	2
4	4	3	4	3	4
4	4	3	3	3	3
4	4	3	3	3	4
3	3	4	4	4	4
4	4	2	2	4	4
4	4	2	4	1	2
3	3	3	3	3	3
4	4	4	4	4	4
4	4	3	3	4	3
2	4	4	3	4	4
4	4	4	4	4	3
3	4	3	3	3	99
4	4	3	3	3	3
2	4	4	4	4	2
4	4	3	4	2	99
4	4	4	4	4	4
4	4	3	3	3	2
4	4	3	3	3	2
2	2	4	3	3	2
4	4	4	4	3	3
4	3	2	3	2	2

4	4	3	4	3	4
4	4	1	1	1	1
4	4	3	4	4	3
4	4	3	3	3	4
3	4	3	3	2	3
4	3	1	1	1	3
4	4	3	3	3	3
4	0	2	2	1	1
4	4	3	3	3	3
4	4	2	3	2	4
4	4	3	3	3	3
2	2	2	4	2	3
4	4	2	2	3	4
3	4	4	1	3	3
4	4	3	3	3	3
4	4	4	1	4	3
4	4	4	2	4	3
4	3	3	3	3	3
99	99	99	99	2	99
0	0	4	4	4	4
4	3	3	3	3	2
4	3	4	3	3	1
3	3	3	3	3	3
4	4	1	4	4	4
4	4	3	4	4	4
1	2	3	3	3	3
4	4	3	3	3	2
4	4	2	3	4	3
4	4	4	4	4	3
4	4	2	3	4	4
0	0	1	1	1	1
4	99	3	2	3	2
4	4	4	4	4	3
4	4	3	3	4	4
4	4	3	3	4	4
4	4	3	3	2	4
4	4	3	4	3	3
4	4	4	4	4	2
1	1	4	4	3	4
4	4	3	3	3	3
4	4	4	3	3	3
4	3	4	4	2	99
2	3	2	3	3	3
4	4	2	3	3	4
4	4	3	2	1	3
4	4	4	4	1	1
4	4	2	3	3	4
1	0	2	2	1	1

4	4	3	4	3	3
4	4	3	3	3	99
4	4	4	4	4	4
4	4	3	3	3	2
4	4	3	3	3	3
4	4	4	4	4	2
4	4	4	3	2	2
4	2	3	4	3	3
4	3	3	3	3	2
4	4	2	3	1	99
99	99	99	99	99	99
2	1	3	3	3	3
4	4	3	3	3	2
4	4	3	3	3	3
4	4	4	99	3	3
4	4	3	2	1	3
4	4	2	3	3	3
4	4	3	3	3	4
3	4	2	3	4	4
1	0	3	3	3	3
4	4	3	3	3	3
4	4	4	4	4	3
4	4	1	1	1	1
1	0	3	3	3	3
4	4	4	3	3	4
2	2	3	1	1	3
4	4	3	3	3	99
3	2	3	2	1	2
4	4	2	2	3	2
4	4	2	3	2	1
2	2	3	1	1	1
2	2	3	2	1	1
3	2	4	3	4	3
4	4	3	3	3	2
4	4	3	3	3	3
4	4	4	3	3	99
4	4	2	3	3	3
4	4	3	4	3	3
4	4	3	4	4	4
3	4	3	3	3	4
4	4	2	2	3	3
3	3	3	3	3	3
1	1	3	3	3	3
4	4	4	4	4	4
2	2	3	2	1	3
4	4	2	1	2	2
4	4	3	3	3	3
1	3	3	2	1	2

4	4	1	4	1	3
4	4	2	3	2	2
4	4	4	2	4	2
4	3	3	4	2	3
4	4	3	3	4	2
2	2	3	3	3	3
4	4	3	3	3	3
4	4	3	3	3	3
0	0	3	3	3	3
99	3	3	3	3	3
2	4	3	3	2	2
4	2	3	3	3	2
4	4	4	4	3	4
0	0	2	3	3	3
99	99	3	1	1	1
4	4	3	3	4	4
0	0	3	3	3	2
3	99	3	99	3	2
4	4	4	4	1	4
3	2	3	1	1	2
0	0	4	4	3	2
4	4	3	3	3	3
4	3	3	4	3	3
4	4	4	1	1	1
3	3	3	3	2	2
4	4	3	3	3	3
4	4	2	3	3	3
4	4	4	4	2	4
4	3	3	3	3	2
3	2	2	3	3	4
4	3	4	3	3	3
4	4	3	3	4	4
4	4	4	2	4	4
2	3	4	4	3	4
4	4	3	2	2	3
4	3	2	2	2	3
0	4	4	2	4	4
2	1	2	3	2	3
3	3	4	3	3	1
4	4	3	3	99	99
4	4	4	3	3	2
4	4	3	4	2	1
4	4	4	4	4	3
3	4	4	3	3	3
3	3	4	3	3	2
4	4	3	3	3	3
99	99	3	2	3	2
4	4	3	3	3	2

4	4	3	3	1	3
4	4	3	3	4	3
4	4	4	4	4	4
4	99	3	3	2	3
4	4	3	3	3	4
3	99	3	3	4	2
4	4	3	2	1	3
4	4	2	2	3	3
4	4	3	3	4	4
4	4	4	4	4	4
3	2	2	2	2	2
4	4	3	3	3	3
4	4	3	4	4	3
4	3	4	4	4	3
3	2	3	3	2	99
4	4	1	1	1	1
4	4	4	2	1	3
4	4	3	1	2	1
4	4	2	4	4	4
99	99	2	2	3	3
4	4	3	4	3	4
4	4	4	4	4	4
4	4	2	2	2	3
2	2	2	2	2	3
4	4	3	3	4	2
4	4	3	4	4	3
4	4	3	3	3	1
4	4	4	3	4	3
2	2	2	1	1	1
3	3	3	2	3	2
4	4	2	3	2	3
4	4	4	2	2	2
2	1	2	2	2	3
4	4	4	4	3	3
4	4	3	3	3	3
2	2	3	3	3	3
0	0	3	3	3	3
0	0	3	4	4	4
4	4	2	3	3	3
4	4	4	3	1	2
4	4	4	4	4	4
4	4	3	3	4	3
4	4	4	4	4	4
2	4	3	2	2	2
1	1	3	2	2	2
99	99	4	1	1	1
99	99	1	3	3	4
2	2	3	3	3	3

4	2	3	3	3	3
4	4	3	3	3	4
0	0	2	3	3	4
2	0	3	3	3	3
4	4	3	4	4	4
4	4	3	3	3	2
4	4	99	99	99	99
4	4	3	3	3	3
4	4	4	4	4	4
4	4	3	3	2	4
3	99	4	4	4	3
99	99	2	4	4	4
4	4	4	4	4	3
4	4	3	3	3	3
4	99	3	2	1	99
4	4	2	2	4	4
4	4	3	3	3	2
4	4	4	4	4	2
4	4	3	2	3	2
4	4	4	3	3	1
4	4	3	3	2	3
4	3	3	3	3	2
4	4	3	3	4	3
4	4	4	2	2	2
4	4	3	3	3	3
4	4	3	3	3	2
1	0	4	4	2	3
1	1	4	4	3	4
1	1	3	3	3	3
4	4	99	99	1	1
4	4	3	2	2	3
3	4	3	3	3	3
4	4	3	3	99	99
4	4	3	3	2	1
4	4	3	3	2	3
0	0	4	2	4	4
2	2	3	4	2	3
4	4	3	3	3	3
3	3	3	3	3	4
4	4	3	3	3	2
4	3	3	3	3	2
4	4	3	3	3	2
4	4	3	4	3	2
4	4	4	4	4	2
4	4	2	2	2	1
4	4	4	4	4	4
4	4	4	4	3	3
1	1	3	2	4	2

4	3	3	2	3	3
2	2	3	3	2	2
4	4	4	4	3	3
4	4	3	3	3	3
4	4	3	4	4	4
4	4	2	2	3	3
3	4	3	3	3	3
4	4	3	3	3	4
3	2	3	3	2	2
4	4	3	3	4	4
4	4	3	3	2	2
3	3	1	1	1	1
4	2	2	2	2	2
3	3	4	4	2	1
4	4	4	4	4	4
3	3	3	3	3	3
4	4	2	3	3	3
3	3	3	3	3	3
4	4	4	4	3	3
4	4	3	4	3	3
4	2	1	3	1	2
4	3	4	3	2	3
4	4	99	99	3	99
4	4	4	4	3	3
4	4	4	4	4	4
2	2	4	3	2	3
2	4	3	3	3	3
4	4	3	4	3	2
4	3	3	4	2	4
4	4	3	4	4	4
4	4	4	4	3	2
3	3	3	2	1	2
4	4	3	3	3	2
4	4	1	1	4	4
3	4	3	3	2	3
4	4	3	4	4	4
0	0	3	4	4	4
4	4	3	3	3	3
4	2	4	2	1	3
4	3	4	3	4	4
4	3	3	3	2	2
4	4	3	3	3	3
4	2	3	4	4	3
4	3	3	3	2	4
4	4	3	3	3	3
4	2	3	3	4	4
4	4	3	4	3	3
3	3	3	3	3	3

3	2	3	3	3	3
4	4	3	3	2	3
0	3	3	3	2	2
4	4	2	3	2	1
4	4	2	2	1	2
3	2	2	2	4	4
4	4	3	3	3	3
4	4	3	3	3	3
4	4	2	3	2	3
3	3	3	3	3	3
4	4	2	2	4	4
4	4	3	1	1	1
4	4	4	4	4	3
4	4	4	4	3	3
4	3	3	2	2	1
3	2	2	3	3	3
2	2	99	1	1	2
1	4	3	2	4	2
99	99	99	99	99	99
4	4	3	3	4	4
4	4	4	4	4	3
4	4	1	2	4	4
4	4	3	4	3	3
3	3	3	3	3	3
0	2	2	3	3	3
2	2	3	3	2	2
4	4	4	3	4	3
4	4	3	3	3	3
4	4	3	3	3	3
4	4	3	2	3	3
4	4	4	3	3	3
4	4	3	3	3	3
3	2	3	4	1	3
4	3	3	2	1	2
4	3	2	2	3	2
4	4	4	3	3	4
4	4	3	2	2	3
2	2	2	3	2	2
4	4	2	2	2	2
4	4	4	4	4	4
4	4	4	3	3	3
3	1	2	2	3	4
4	4	3	3	2	3
4	4	3	3	3	3
4	3	3	4	3	4
4	4	2	3	2	3
3	3	3	2	3	3
4	4	4	4	4	4

1	3	3	3	3	3
4	4	3	3	3	2
4	4	4	4	1	1
4	4	1	3	1	2
4	4	2	3	3	3
4	3	3	4	4	3
4	4	4	4	3	4
1	1	2	2	2	2
2	2	3	3	2	3
2	2	3	3	2	3
4	4	3	2	1	3
0	0	3	3	2	3
4	4	3	3	3	4
4	4	3	3	3	3
3	4	3	3	3	2
4	3	2	2	3	3
2	2	3	3	3	2
4	4	3	3	3	3
4	4	3	3	3	2
3	4	3	4	3	3
4	4	4	4	4	4
4	1	3	3	3	4
3	3	3	4	1	2
0	0	1	1	1	1
4	4	4	3	3	3
4	4	3	3	3	3
4	3	4	4	3	3
4	4	3	3	3	3
4	4	3	3	3	3
1	2	3	3	3	2
4	4	3	3	3	3
4	4	3	3	2	2
4	4	3	3	3	1
4	4	2	1	2	1
1	3	3	3	3	4
2	3	2	3	2	3
2	3	4	4	4	4
4	4	3	3	3	3
1	1	4	4	4	3
1	1	2	2	3	3
0	0	4	4	4	4
4	4	3	3	3	2
1	1	1	1	1	1
2	2	3	3	3	3
2	2	4	4	2	4
4	4	3	4	2	4
2	2	1	3	2	3
3	3	3	4	4	4

4	4	3	3	3	3
4	4	3	3	3	3
2	2	2	2	2	3
3	2	2	2	2	3
2	2	3	3	2	3
2	2	2	3	3	3
3	3	3	3	4	4
0	0	4	4	4	4
4	0	4	4	1	3
4	4	4	4	4	4
4	4	3	4	4	4
1	1	4	4	4	4
#NULL!	#NULL!	#NULL!	#NULL!	#NULL!	#NULL!
4	4	2	2	1	3
3	1	2	1	1	2
4	4	3	3	3	3
4	4	4	1	1	4
3	3	3	3	3	1
4	4	4	3	3	2
4	4	3	2	3	4
3	3	3	3	3	3
4	4	1	1	1	1
0	0	2	3	2	3
4	4	2	3	3	3
4	4	1	1	1	3
4	4	3	3	2	3
2	1	3	2	1	3
4	3	3	2	3	3
3	3	2	3	3	3
4	4	2	3	4	3
3	2	1	1	1	3
4	4	3	3	3	2
4	4	4	4	4	4
2	3	3	3	2	4
4	3	4	3	2	4
4	4	3	3	3	3
4	4	3	3	1	3
4	4	3	3	3	3
4	4	4	4	4	4
4	4	3	3	3	4
4	4	4	4	4	4
4	4	4	2	4	4
4	4	3	3	3	3
3	3	2	3	2	3
1	3	1	1	1	4
3	3	2	2	2	3
4	4	4	4	3	3
4	4	4	4	4	4

4	3	3	3	3	3
4	4	4	4	2	2
4	4	2	3	3	3
4	4	3	3	3	3
4	4	4	4	4	4
3	4	3	3	3	3
2	3	4	4	4	4
4	4	4	3	1	3
4	4	4	3	3	3
4	4	2	3	2	4
4	4	3	3	3	3
4	4	3	4	3	3
4	4	3	4	2	4
4	4	3	2	3	3
3	3	3	3	3	3
4	4	3	3	3	3
#NULL!	#NULL!	#NULL!	#NULL!	#NULL!	#NULL!
4	4	1	3	4	4
3	3	3	3	4	2
4	4	4	3	3	3
4	3	2	2	2	4

sconnecte_pre	sexknowa_pre	sexknowb_pre	sexknowc_pre	sexknowd_pre	sexknowe_pre
3	2	2	2	2	2
4	2	2	2	2	2
3	2	2	0	1	2
2	2	1	0	0	2
4	2	2	99	99	2
99	99	99	99	99	99
4	2	1	1	0	1
2	2	1	0	0	1
99	99	99	99	99	99
2	2	0	0	0	0
4	2	2	2	2	2
3	2	0	0	0	0
3	2	2	2	2	2
3	2	2	2	2	2
4	2	2	2	2	2
3	2	2	2	2	2
4	2	2	2	2	2
2	2	2	0	2	0
4	2	0	2	0	2
4	1	1	1	0	0
99	99	99	99	99	99
4	2	2	2	2	2
4	2	2	2	2	2
4	2	2	2	0	2
4	2	2	2	2	2
3	2	2	2	2	2
3	0	0	0	0	2
4	2	2	2	2	2
4	2	2	2	2	2
3	2	2	2	1	1
4	2	1	0	1	1
3	0	0	0	1	1
3	2	1	0	0	2
3	99	1	99	0	1
4	99	99	99	99	99
4	2	2	2	2	2
3	0	0	1	0	1
3	2	2	0	2	2
3	2	1	0	1	2
3	1	0	0	0	0
4	2	2	2	2	2
1	2	2	2	2	2
3	2	2	0	0	0
4	2	2	2	2	2
4	2	2	0	0	2
4	2	2	0	0	2
2	1	0	0	0	1

1	1	0	0	0	0
4	2	0	0	0	2
3	2	2	0	0	2
2	1	0	0	0	1
3	2	1	1	0	0
3	2	1	0	0	1
3	2	2	2	2	2
2	2	2	1	0	1
3	2	2	0	2	2
4	2	2	2	2	2
3	2	2	2	2	2
3	2	2	2	2	2
4	2	2	2	2	2
3	2	0	0	0	1
3	0	0	0	0	1
1	2	1	0	0	2
3	0	1	0	0	1
1	2	2	0	2	1
2	1	2	0	2	2
4	1	0	1	0	1
3	2	0	0	2	2
4	2	0	0	0	0
3	2	0	0	0	1
4	2	0	2	2	1
3	2	2	0	2	2
4	2	2	2	2	2
4	2	2	2	2	2
4	0	1	1	0	1
2	2	0	0	0	0
3	2	1	0	2	1
4	2	0	2	0	2
3	0	2	2	2	0
3	0	0	0	2	2
3	1	2	0	0	1
2	2	2	2	0	2
2	2	1	0	0	2
1	2	2	0	2	2
3	1	2	1	0	1
3	1	0	0	0	2
4	1	0	0	0	1
3	1	0	2	0	1
2	1	0	2	0	0
1	0	0	2	0	1
1	2	2	2	2	2
99	2	1	0	0	2
3	1	0	0	0	1
3	1	0	0	0	1
3	1	0	0	0	1

3	0	1	0	2	2
3	1	0	0	0	0
3	0	0	0	0	1
4	1	0	0	0	1
4	1	1	0	0	0
3	0	0	0	0	1
1	2	0	0	0	1
3	0	2	0	0	1
3	1	0	0	0	2
3	1	0	0	0	1
3	0	0	0	0	0
1	1	0	0	0	1
3	1	0	2	0	1
3	0	2	1	0	1
3	2	2	0	2	2
4	1	0	0	0	0
4	1	0	0	0	0
2	2	0	0	0	0
3	1	2	0	0	1
3	2	2	2	2	2
3	1	1	0	0	0
3	0	1	0	0	1
4	1	1	1	0	0
1	0	0	0	0	1
4	0	0	0	0	0
3	1	1	1	0	1
3	0	0	0	0	1
4	1	0	0	0	1
3	1	0	0	0	1
3	1	0	0	0	1
99	99	99	99	99	99
1	1	0	0	0	0
99	99	99	99	99	99
99	1	0	0	0	1
3	0	0	0	0	1
2	1	1	0	0	1
3	0	0	0	0	0
3	1	1	0	1	1
3	0	0	0	0	1
2	0	1	0	0	1
3	1	0	0	0	1
3	1	1	0	0	1
3	2	0	0	0	0
3	1	1	0	0	0
3	1	0	0	0	0
99	99	99	99	99	99
3	1	0	0	0	1
2	99	0	0	0	0

2	1	1	0	0	1
3	1	0	0	0	1
4	1	0	0	0	1
1	1	0	1	0	0
2	0	99	0	0	1
3	0	0	0	0	0
3	0	1	0	1	0
3	1	1	0	0	1
2	0	0	0	1	0
1	0	1	0	0	0
4	0	0	0	0	0
4	1	2	99	99	99
2	0	1	0	0	1
3	0	1	0	0	1
3	0	0	0	1	0
3	0	0	0	1	0
3	0	1	0	1	0
4	0	1	0	1	1
3	1	1	0	1	1
3	0	0	0	1	0
3	1	0	1	1	0
4	0	1	0	1	0
4	1	1	0	0	1
3	1	0	0	0	1
3	0	0	0	0	0
3	0	0	0	0	1
3	1	0	0	0	1
3	0	1	0	1	0
4	1	1	0	0	0
4	1	0	0	0	0
4	99	99	99	99	99
3	0	1	0	0	0
3	0	0	0	1	1
3	0	0	0	0	0
3	0	1	0	0	1
3	99	1	0	0	0
4	1	0	0	0	0
2	0	0	0	1	0
4	1	1	1	1	0
3	1	0	0	0	1
4	1	0	0	0	0
3	1	1	0	1	1
4	0	1	0	0	1
1	1	0	0	0	1
2	1	0	0	0	1
1	0	1	0	0	1
4	0	0	0	0	0
4	1	1	0	0	0

4	99	99	0	1	99
4	0	1	0	0	0
4	2	2	2	2	2
3	1	1	0	0	0
4	1	0	0	0	1
1	1	0	0	0	1
3	1	1	0	0	1
2	1	0	0	0	1
3	0	0	1	1	1
3	1	0	0	0	1
3	99	0	0	0	1
2	1	1	0	0	1
2	1	0	0	0	1
2	1	0	0	0	1
4	0	0	0	0	0
3	1	1	0	0	1
3	0	0	0	0	1
3	1	99	0	1	1
3	1	0	0	0	1
2	1	0	0	0	1
4	1	0	0	0	1
4	1	1	1	0	1
4	1	0	0	1	1
3	0	0	0	0	0
1	1	0	99	0	1
2	1	1	0	0	0
1	0	0	0	1	0
3	1	0	0	0	1
1	1	0	0	0	1
4	1	0	0	0	1
3	1	0	0	0	1
3	1	0	0	0	1
4	1	0	0	0	1
99	1	1	99	0	1
2	0	0	0	0	0
1	1	0	0	0	1
3	0	0	0	0	1
3	1	1	0	1	0
1	0	1	0	0	1
2	1	1	0	0	1
3	1	0	0	1	1
2	1	0	0	0	1
4	1	0	0	0	1
3	1	1	0	0	0
1	0	1	0	0	0
1	0	0	0	1	1
3	0	0	0	0	0
4	0	0	0	0	0

4	0	1	0	0	1
1	1	0	0	0	1
2	0	1	0	0	0
3	1	1	0	1	0
4	1	1	99	0	0
3	1	1	0	0	1
3	1	0	0	0	0
1	1	1	1	0	1
1	1	1	1	0	1
2	1	0	0	0	1
3	99	99	0	0	99
3	99	99	0	0	99
3	0	0	0	0	0
3	1	1	0	0	1
4	1	1	0	1	1
2	1	1	0	0	1
3	1	1	0	1	1
1	99	99	99	99	99
2	1	0	0	1	0
4	0	0	1	0	0
3	1	1	0	0	1
1	1	0	0	0	0
3	1	0	0	0	1
3	0	1	0	1	0
2	1	0	0	1	0
3	1	1	0	0	1
4	1	0	0	1	0
3	1	1	0	0	0
3	0	0	0	0	0
3	99	99	99	0	0
3	1	1	0	1	0
3	1	1	0	1	0
3	0	1	0	0	1
3	1	1	0	0	0
4	99	2	2	2	99
3	1	1	0	0	0
2	99	99	99	99	99
2	99	1	0	0	99
3	0	0	0	0	1
3	1	1	0	0	0
1	1	0	0	1	1
3	99	99	0	99	0
3	0	1	0	0	0
3	1	1	0	0	0
99	0	1	0	0	0
2	1	0	0	0	0
2	0	1	0	0	0
4	1	0	0	0	1

3	1	1	0	0	1
4	0	0	0	0	0
3	0	1	0	0	1
99	99	99	99	99	99
4	0	0	0	0	0
3	0	0	0	0	1
2	0	0	1	0	1
2	99	1	0	0	0
1	0	0	0	0	1
3	1	0	0	0	1
3	0	0	0	0	1
3	1	0	0	0	1
3	0	0	0	0	0
2	0	1	0	1	1
3	1	0	0	0	1
4	1	0	0	0	0
3	1	1	0	0	0
2	1	0	0	1	1
4	1	0	0	0	1
4	0	1	0	0	1
3	1	0	0	0	1
4	1	0	0	0	1
3	1	0	0	0	1
3	1	0	0	0	1
3	1	0	0	0	1
3	0	99	1	0	1
3	1	0	0	0	1
1	1	1	0	0	1
4	99	99	99	99	99
2	99	99	99	99	99
3	1	0	0	0	0
3	1	0	0	0	1
3	1	1	0	0	1
3	0	0	0	0	1
3	1	1	0	0	1
3	1	0	0	0	1
2	1	0	0	0	1
3	1	1	0	0	1
4	0	0	0	0	0
3	1	0	0	0	1
3	0	0	0	0	1
3	0	0	0	0	1
2	1	1	0	0	1
2	1	0	0	0	1
2	1	0	0	0	0
3	1	0	0	0	1
2	0	1	0	0	1
3	0	0	0	0	0

3	0	0	0	0	1
2	0	0	0	0	1
99	1	1	0	0	0
3	1	0	0	0	1
3	0	1	0	1	0
99	0	0	0	0	0
1	1	0	0	0	1
2	1	0	0	0	1
2	0	0	0	0	1
4	1	0	0	0	1
3	1	0	0	0	1
1	99	99	99	99	99
2	1	0	0	0	1
3	99	0	0	0	1
3	1	0	0	0	1
1	1	0	0	0	1
3	1	0	0	0	1
4	1	1	0	0	1
3	2	1	0	1	1
1	0	0	0	0	1
2	0	0	1	1	1
1	1	1	0	0	1
3	1	0	0	0	1
1	0	1	0	0	1
3	1	0	0	0	0
3	0	1	1	0	1
4	0	0	0	0	1
4	99	99	99	99	99
3	1	0	0	0	1
4	1	1	99	1	1
2	1	1	0	0	1
4	0	1	0	0	0
3	0	0	0	0	1
4	99	99	99	99	99
3	1	1	1	1	1
4	0	0	0	0	0
1	0	0	0	0	1
4	1	0	1	0	1
3	0	1	0	1	0
3	99	1	0	0	1
4	1	0	0	0	1
3	0	1	0	0	1
3	1	0	0	1	0
1	0	1	0	1	0
2	1	1	1	1	1
1	0	0	0	0	0
3	99	99	0	99	99
3	1	1	0	1	1

4	1	0	0	0	1
4	1	0	0	0	1
3	1	0	0	0	1
4	0	1	0	99	99
3	1	0	0	1	0
3	0	1	0	0	1
3	0	1	0	0	0
1	1	0	1	0	1
3	1	0	1	1	1
1	1	0	0	0	0
2	0	0	0	0	0
4	0	1	0	0	1
3	99	0	0	0	1
3	0	1	1	0	1
3	99	0	0	0	1
3	0	1	0	1	0
3	1	0	0	0	1
1	0	0	0	0	1
3	1	0	0	0	1
4	0	0	0	0	1
2	1	0	1	0	1
4	0	1	0	0	0
2	0	1	0	0	1
99	99	99	99	99	99
3	1	1	0	0	1
1	1	1	0	0	1
2	1	0	0	0	1
3	1	0	0	0	1
4	1	0	0	0	0
3	0	1	0	1	0
3	1	0	0	1	1
2	0	0	0	0	1
4	1	1	0	1	1
3	0	0	0	1	0
3	1	1	0	0	0
3	1	0	0	0	1
4	0	0	0	0	1
3	1	0	0	0	0
1	0	0	0	0	1
4	2	2	2	2	2
4	99	0	0	0	1
2	1	0	0	0	1
2	0	0	1	0	1
99	99	99	99	99	99
3	99	0	0	0	1
2	99	0	0	0	1
3	0	0	0	0	1
99	1	0	99	0	1

4	0	0	0	0	0
4	0	99	0	0	1
4	99	99	99	99	99
4	1	0	0	1	1
3	99	0	0	0	0
3	0	0	0	0	1
2	1	0	0	0	0
2	0	0	0	0	1
3	1	99	0	0	1
2	0	0	0	0	1
2	1	0	0	0	1
3	0	0	0	0	1
4	1	1	0	0	1
2	1	0	0	0	1
3	0	0	0	0	1
1	0	0	0	0	0
1	0	0	0	0	0
3	0	1	1	1	1
4	0	0	0	0	1
2	1	1	0	1	1
1	0	0	0	0	1
99	99	99	99	99	99
2	0	0	0	0	1
3	1	1	0	0	1
2	1	0	0	0	1
2	1	1	0	0	1
3	1	0	0	0	1
1	0	0	0	0	1
3	0	1	0	0	1
3	0	1	0	0	1
2	1	1	0	0	1
1	1	0	0	0	1
3	99	0	0	0	1
2	1	99	0	0	1
4	1	0	0	0	1
4	0	0	0	0	0
2	1	0	0	0	1
2	0	1	0	0	1
3	1	0	0	0	0
2	1	1	0	0	1
3	1	0	0	0	1
99	99	99	99	99	99
1	1	1	0	0	0
3	0	1	0	0	1
3	1	0	1	0	1
2	1	0	0	0	0
3	1	0	1	0	1
2	1	0	0	0	0

2	1	1	0	1	0
3	99	0	0	0	99
3	0	1	0	0	1
3	99	99	99	99	99
2	0	0	0	0	1
3	1	0	0	0	1
4	0	0	0	1	0
1	0	1	0	1	1
4	0	0	1	0	0
1	0	1	0	0	99
3	99	0	99	0	99
1	0	0	0	1	1
3	1	0	0	0	0
3	0	0	0	0	1
1	99	99	99	99	99
4	1	1	0	0	1
2	0	0	0	0	0
3	1	1	0	1	1
3	1	0	0	0	0
2	0	1	1	0	1
2	0	0	0	0	1
2	0	1	0	0	0
4	1	1	0	0	0
3	0	0	1	0	1
99	99	99	99	99	99
3	1	0	1	0	1
3	0	0	1	0	1
2	1	0	1	1	1
4	0	0	0	1	0
2	1	0	0	0	0
3	0	0	1	0	0
4	99	1	1	0	0
2	0	0	0	1	1
1	99	1	99	99	99
4	99	99	99	99	99
3	0	0	1	0	1
2	1	1	0	0	1
2	0	1	0	0	0
3	1	0	0	0	1
1	0	1	1	0	1
3	1	1	0	0	1
3	0	0	0	0	0
2	1	0	0	0	1
2	1	0	1	0	1
3	1	0	0	0	1
3	1	0	0	0	0
99	99	0	0	0	1
2	1	0	0	1	1

2	1	0	0	0	0
3	1	0	0	0	1
99	99	0	0	0	0
3	1	0	0	0	0
3	1	0	0	0	1
3	1	0	0	0	1
3	99	99	0	99	99
1	99	99	99	99	99
4	1	0	1	0	0
3	0	0	0	0	0
1	1	0	1	0	1
4	0	0	0	0	0
4	1	1	0	1	0
3	0	0	0	0	1
99	99	99	99	99	99
3	1	0	0	0	1
3	1	0	0	99	1
4	0	0	0	0	1
3	1	0	0	0	1
3	1	0	0	0	1
3	0	0	1	0	1
3	0	1	0	0	1
2	0	1	0	1	0
4	1	0	0	0	1
2	1	0	0	0	1
99	1	0	0	0	1
1	99	0	0	99	99
3	0	0	0	0	1
3	1	1	0	0	0
3	1	1	0	0	1
3	1	1	0	0	1
3	0	0	0	0	0
1	1	0	0	0	0
3	1	0	0	0	1
2	99	99	99	99	99
1	99	99	99	99	99
3	1	0	0	1	1
3	1	1	0	0	0
3	0	0	0	0	1
3	0	0	0	1	1
1	99	0	0	0	1
3	1	0	0	0	1
3	0	0	0	0	1
2	1	0	1	0	1
99	1	0	0	0	99
4	1	1	1	1	0
3	99	99	0	99	99
3	0	0	0	0	0

3	1	1	1	1	1
1	0	0	0	0	0
2	0	0	0	1	1
2	0	0	0	0	1
3	0	0	0	0	1
4	1	0	0	0	1
4	1	0	0	1	1
3	0	0	0	0	1
3	1	1	0	0	1
1	0	1	0	0	0
2	0	0	1	0	0
2	0	0	0	0	0
3	99	99	99	99	99
4	99	99	99	99	99
3	99	99	99	99	99
3	99	99	99	99	99
4	99	99	99	99	99
4	99	99	99	99	99
3	99	99	99	99	99
4	99	99	99	99	99
3	99	99	99	99	99
4	99	99	99	99	99
4	99	99	99	99	99
3	99	99	99	99	99
3	99	99	99	99	99
4	99	99	99	99	99
99	99	99	99	99	99
4	99	99	99	99	99
3	99	99	99	99	99
4	99	99	99	99	99
99	99	99	99	99	99
4	99	99	99	99	99
4	99	99	99	99	99
4	99	99	99	99	99
3	99	99	99	99	99
3	99	99	99	99	99
3	99	99	99	99	99
4	99	99	99	99	99
4	99	99	99	99	99
4	99	99	99	99	99
4	99	99	99	99	99
4	99	99	99	99	99
3	99	99	99	99	99
4	99	99	99	99	99
3	99	99	99	99	99
4	99	99	99	99	99
4	99	99	99	99	99
4	99	99	99	99	99

4	99	99	99	99	99
3	99	99	99	99	99
4	99	99	99	99	99
4	99	99	99	99	99
4	99	99	99	99	99
4	99	99	99	99	99
4	99	99	99	99	99
3	99	99	99	99	99
4	99	99	99	99	99
4	99	99	99	99	99
3	99	99	99	99	99
3	99	99	99	99	99
3	99	99	99	99	99
3	99	99	99	99	99
3	99	99	99	99	99
3	99	99	99	99	99
4	99	99	99	99	99
3	99	99	99	99	99
3	99	99	99	99	99
2	99	99	99	99	99
1	99	99	99	99	99
3	99	99	99	99	99
3	99	99	99	99	99
4	99	99	99	99	99
3	99	99	99	99	99
4	99	99	99	99	99
1	99	99	99	99	99
3	99	99	99	99	99
4	99	99	99	99	99
3	99	99	99	99	99
2	99	99	99	99	99
3	99	99	99	99	99
1	99	99	99	99	99
3	99	99	99	99	99
99	99	99	99	99	99
2	99	99	99	99	99
2	99	99	99	99	99
4	99	99	99	99	99
3	99	99	99	99	99
3	99	99	99	99	99
1	99	99	99	99	99
3	99	99	99	99	99
3	99	99	99	99	99
3	99	99	99	99	99
4	99	99	99	99	99
2	99	99	99	99	99
2	99	99	99	99	99
3	99	99	99	99	99

3	99	99	99	99	99
4	99	99	99	99	99
2	99	99	99	99	99
3	99	99	99	99	99
3	99	99	99	99	99
1	99	99	99	99	99
4	99	99	99	99	99
3	99	99	99	99	99
3	99	99	99	99	99
3	99	99	99	99	99
1	99	99	99	99	99
2	99	99	99	99	99
2	99	99	99	99	99
3	99	99	99	99	99
3	99	99	99	99	99
99	99	99	99	99	99
3	99	99	99	99	99
1	99	99	99	99	99
3	99	99	99	99	99
3	99	99	99	99	99
1	99	99	99	99	99
2	99	99	99	99	99
3	99	99	99	99	99
2	99	99	99	99	99
2	99	99	99	99	99
3	2	2	2	2	99
3	2	2	2	2	2
1	2	1	0	0	1
4	2	0	1	0	1
4	2	2	2	2	2
4	1	0	0	0	1
3	2	1	2	0	2
3	2	2	2	2	2
99	99	99	99	99	99
3	2	1	2	2	1
4	2	2	2	2	2
3	2	1	0	2	2
3	2	2	2	2	2
3	2	0	0	0	1
1	2	2	0	2	2
4	2	2	2	2	2
2	2	0	1	0	0
3	2	0	0	0	1
3	2	2	2	0	2
3	2	2	2	2	2
4	2	0	1	2	2
4	2	2	0	2	2
1	2	1	2	2	2

3	2	2	2	2	2
3	2	2	2	2	2
2	2	2	0	2	1
4	2	2	2	2	2
4	2	2	0	2	2
4	2	2	0	2	2
4	2	1	2	1	2
2	2	2	0	0	2
2	2	2	0	2	2
3	2	1	2	0	1
2	2	2	2	2	2
3	2	0	0	0	2
3	2	2	2	2	2
4	2	0	0	0	2
3	2	2	0	0	2
3	2	0	2	2	2
3	2	2	0	0	2
3	1	0	2	2	0
3	2	1	0	2	2
4	2	2	2	2	2
4	2	0	2	0	1
4	2	1	0	0	1
1	2	2	0	2	2
4	2	2	0	2	2
3	2	2	0	2	2
2	2	0	0	2	1
3	2	1	0	0	2
3	2	1	0	0	2
3	2	1	0	0	2
2	2	2	0	0	1
3	2	2	2	2	2
3	2	0	2	2	2
99	2	2	0	2	2
4	2	2	0	2	2
4	2	2	2	1	2
4	2	1	0	1	2
4	2	2	0	2	2
1	2	2	0	2	1
3	0	0	0	0	0
1	2	2	2	2	2
3	2	0	2	2	2
3	2	2	0	2	2
4	2	2	2	2	2
3	2	2	2	2	2
2	2	2	2	2	1
4	2	1	0	1	1
2	2	2	0	0	2
3	2	2	0	2	1

2	2	0	2	0	1
3	2	2	2	2	2
4	2	0	0	2	2
3	2	2	2	2	2
3	1	2	0	0	2
4	2	2	2	2	1
3	2	2	0	0	1
3	99	0	2	0	1
4	2	2	0	0	1
3	2	99	1	0	2
2	2	2	0	0	2
4	1	1	1	1	1
99	99	99	99	99	99
4	0	0	0	0	0
4	1	0	0	0	1
3	1	0	2	0	1
2	0	0	0	2	0
3	0	1	1	0	1
2	1	0	2	2	2
3	1	0	0	0	2
2	2	2	2	2	2
2	0	0	0	1	1
4	2	2	0	0	0
3	1	0	0	0	1
3	2	0	0	0	0
3	1	0	2	0	1
4	1	1	1	1	1
4	1	2	2	2	2
3	2	2	0	1	1
3	2	2	0	0	2
4	1	1	0	1	1
4	2	2	0	2	2
3	1	1	0	1	1
1	1	0	0	0	1
3	2	0	0	2	2
4	2	2	2	1	2
3	2	0	1	0	0
2	2	1	2	0	1
4	2	2	0	2	2
4	2	2	2	2	2
2	2	2	0	0	0
4	2	2	0	2	0
99	2	0	1	0	1
4	2	0	2	2	2
3	2	2	2	2	2
3	2	2	2	2	2
3	2	2	2	2	2
2	1	0	0	0	1

3	2	0	0	0	2
2	1	1	0	0	1
3	1	1	0	0	2
4	2	2	0	2	2
4	2	2	2	2	2
2	2	1	0	0	2
3	2	2	2	0	2
4	2	1	0	0	2
4	2	2	2	2	2
3	2	2	2	2	2
3	1	2	0	2	1
3	1	2	2	2	1
3	2	2	2	2	2
3	2	2	2	2	2
3	2	2	0	2	2
1	2	0	0	0	1
3	1	1	0	1	1
3	2	2	2	2	1
1	1	1	1	2	0
4	2	2	2	1	2
3	2	1	0	1	1
1	2	2	2	2	2
2	2	0	0	0	1
2	2	0	0	1	0
3	2	0	0	0	2
2	0	1	0	0	0
4	2	2	2	2	1
3	2	0	0	0	1
3	1	0	0	0	2
3	1	0	0	0	1
1	2	0	2	2	1
3	2	2	0	0	0
3	2	2	0	2	2
2	2	0	0	0	0
2	2	0	0	0	1
2	2	2	0	0	1
4	2	2	2	2	2
2	1	0	0	0	1
1	99	99	99	99	99
4	1	2	0	0	1
3	2	2	2	2	2
2	2	2	0	2	2
2	2	0	0	2	2
3	2	2	0	0	1
3	2	0	0	0	1
3	2	2	0	0	2
2	1	0	2	0	1
3	2	0	0	0	1

3	2	0	0	2	1
1	2	0	0	0	2
4	2	2	2	2	2
3	2	0	2	0	1
1	2	2	1	0	1
2	1	1	0	0	2
3	0	0	0	0	2
2	2	0	0	2	1
3	2	0	0	2	1
1	1	2	0	2	2
3	1	0	0	0	0
2	2	1	0	0	1
3	1	2	0	0	2
3	2	0	0	0	2
3	2	0	0	0	0
2	1	0	0	0	1
2	2	0	2	2	2
3	2	2	2	2	2
2	1	2	1	0	1
2	1	1	0	0	0
2	1	1	0	0	0
3	0	1	0	0	2
3	0	0	0	0	1
3	1	1	2	2	2
1	1	1	0	0	1
4	0	0	0	0	0
4	0	0	0	0	2
3	1	0	0	0	2
2	0	0	0	0	1
1	0	1	2	0	1
3	0	0	0	0	1
3	1	0	0	0	1
3	2	0	2	0	2
3	2	2	2	2	2
1	1	0	0	0	0
2	1	1	0	0	1
3	99	99	99	99	99
3	2	0	0	0	0
1	1	0	1	0	1
3	1	2	0	0	1
2	2	2	2	2	2
2	1	0	0	0	1
1	1	0	2	2	2
2	2	0	0	0	1
3	2	2	2	2	0
1	2	0	1	0	2
3	2	0	0	1	2
99	2	0	0	0	2

3	2	0	0	2	2
2	2	1	0	0	2
3	0	99	0	0	2
3	2	2	2	2	2
99	1	1	1	1	1
1	2	1	0	0	0
3	1	2	2	2	2
3	2	0	0	0	2
4	2	2	2	2	2
3	2	2	2	2	2
2	2	0	0	0	1
4	2	2	2	2	2
3	2	2	2	2	2
4	2	1	0	2	2
3	0	0	0	0	0
4	2	99	1	1	1
3	2	1	0	2	2
2	2	2	0	0	0
3	1	1	0	0	2
4	2	0	0	2	1
2	2	2	2	2	2
3	2	2	1	0	2
3	2	2	0	2	2
4	2	2	2	2	2
4	2	0	0	0	2
4	2	2	2	2	2
4	2	2	0	0	0
4	2	2	2	2	2
1	2	0	2	2	2
2	2	2	2	0	2
3	2	2	2	0	2
2	1	1	1	2	0
4	1	2	2	2	2
3	2	0	0	0	2
3	2	2	2	2	0
3	2	0	0	1	2
3	1	0	2	0	1
1	2	1	1	0	0
3	2	2	2	2	2
3	0	2	2	2	2
4	2	0	0	2	2
4	2	2	2	2	2
4	2	0	0	2	1
3	0	0	1	0	2
4	2	0	2	0	2
2	2	2	2	0	2
3	2	2	0	2	2
3	2	1	0	0	0

4	2	2	0	2	1
3	1	0	0	0	1
4	2	0	0	0	1
3	2	0	0	2	2
4	2	1	1	0	0
3	2	2	0	2	2
3	2	2	0	0	2
99	2	1	99	99	99
3	1	1	0	1	1
4	2	0	0	2	2
3	2	2	2	2	2
99	1	2	0	2	1
3	2	0	2	2	1
3	2	0	1	1	1
2	2	2	0	2	2
3	2	0	1	0	1
3	0	1	0	0	1
2	0	2	0	2	2
3	2	0	0	2	1
3	2	2	0	2	0
3	1	0	0	0	1
4	2	0	0	2	1
3	2	0	0	2	2
1	1	0	0	0	1
2	0	0	0	2	2
99	2	2	2	2	2
2	2	0	99	0	0
3	99	99	99	99	99
4	2	0	0	0	1
4	1	0	0	0	2
3	2	2	0	2	2
1	2	2	0	2	2
2	0	1	0	0	2
3	2	1	0	2	2
4	0	1	0	2	1
4	0	2	1	0	2
1	1	1	0	0	1
2	2	0	0	0	1
1	0	1	0	0	0
3	2	2	0	0	1
3	2	0	2	2	2
3	2	1	0	1	2
3	2	2	2	2	2
3	99	1	0	2	2
3	2	2	2	1	2
2	2	1	0	2	1
4	1	2	0	0	0
4	2	1	0	0	1

1	2	1	0	1	0
3	2	2	2	0	1
3	1	0	2	2	2
3	2	2	0	0	2
99	99	99	99	99	99
3	99	99	99	99	99
2	99	99	99	99	99
2	99	99	99	99	99
1	99	99	99	99	99
2	99	99	99	99	99
3	99	99	99	99	99
3	99	99	99	99	99
3	99	99	99	99	99
4	99	99	99	99	99
3	99	99	99	99	99
2	99	99	99	99	99
3	99	99	99	99	99
3	99	99	99	99	99
1	99	99	99	99	99
3	99	99	99	99	99
4	99	99	99	99	99
4	99	99	99	99	99
4	99	99	99	99	99
3	99	99	99	99	99
2	99	99	99	99	99
1	99	99	99	99	99
3	99	99	99	99	99
3	99	99	99	99	99
4	99	99	99	99	99
3	99	99	99	99	99
4	99	99	99	99	99
3	99	99	99	99	99
2	99	99	99	99	99
3	99	99	99	99	99
2	99	99	99	99	99
1	99	99	99	99	99
3	99	99	99	99	99
3	99	99	99	99	99
3	99	99	99	99	99
3	99	99	99	99	99
4	99	99	99	99	99
2	99	99	99	99	99
3	99	99	99	99	99
3	99	99	99	99	99
3	99	99	99	99	99
1	99	99	99	99	99
1	99	99	99	99	99
3	2	2	0	2	2

3	2	1	2	0	1
3	0	0	0	2	2
3	2	0	2	2	2
3	2	1	2	2	2
4	2	1	2	2	2
3	0	2	0	0	2
1	2	2	2	2	2
3	2	1	0	0	2
4	2	2	0	2	2
3	2	0	0	0	1
3	2	2	2	2	2
3	2	0	0	2	2
4	1	0	0	0	1
3	2	1	2	1	1
3	2	2	0	2	2
3	2	2	0	0	2
4	2	0	0	0	2
4	2	2	0	2	2
3	2	0	0	1	1
4	2	2	2	2	2
4	2	2	0	0	2
3	2	2	0	2	2
2	2	1	0	0	1
4	2	2	0	2	1
4	2	0	0	0	1
4	0	0	0	0	2
3	2	1	0	0	2
3	1	0	0	0	1
99	2	2	2	2	2
3	2	2	2	2	2
99	2	2	0	2	2
4	0	0	0	0	1
3	2	2	1	2	1
2	2	0	0	0	2
3	2	0	0	0	1
3	2	0	0	0	2
3	2	0	0	0	0
2	2	0	2	0	1
2	0	0	2	0	1
2	2	2	0	0	2
2	2	2	2	2	2
1	2	2	0	0	2
99	2	2	0	0	2
3	2	1	0	0	2
4	2	2	0	0	0
3	2	2	2	1	2
4	2	0	0	2	2
2	2	0	2	0	2

3	2	2	2	2	2
3	2	0	2	0	2
3	1	0	0	0	2
3	2	2	0	0	2
1	2	2	0	0	1
1	2	0	0	0	2
3	0	0	0	2	0
4	1	2	0	0	2
3	2	2	0	2	2
3	2	2	2	2	2
1	99	99	1	1	1
2	2	1	0	1	2
3	2	1	0	0	0
2	2	0	0	0	1
3	1	1	1	1	1
3	1	1	1	1	1
4	2	0	2	2	1
2	2	2	2	1	0
3	1	0	0	0	1
4	1	2	0	1	2
3	2	0	0	0	1
2	2	99	0	0	1
2	1	1	0	0	2
2	1	0	2	0	1
1	1	0	0	0	1
3	2	2	2	2	2
3	0	1	1	0	0
2	2	0	0	0	2
2	1	1	0	0	1
2	2	2	2	2	2
3	0	0	0	0	0
2	1	0	2	0	1
3	1	0	0	0	1
1	1	0	0	0	1
4	1	0	0	0	1
2	1	2	0	2	2
1	2	0	0	0	1
3	1	0	2	0	1
4	1	0	0	0	1
2	0	0	0	0	1
3	1	2	0	0	2
3	1	1	1	1	1
99	1	2	0	0	1
2	2	2	0	0	1
2	99	99	99	99	99
3	2	0	0	0	1
3	1	0	1	0	1
3	1	0	0	0	1

4	1	0	0	0	1
1	0	0	1	0	1
1	0	1	1	1	0
3	0	0	0	0	2
3	1	0	0	0	1
1	0	0	1	0	1
2	2	2	2	2	2
2	0	0	2	2	2
2	1	0	0	0	1
4	2	2	2	2	2
2	2	0	0	0	2
2	2	0	0	2	1
1	2	1	0	2	2
3	2	0	0	0	0
3	1	0	0	0	1
2	2	2	1	2	2
4	1	2	0	0	2
4	2	2	0	0	2
2	2	1	0	0	0
2	2	0	0	0	1
4	2	0	2	2	0
4	2	2	2	2	1
3	2	2	0	2	1
3	2	1	0	0	2
3	2	2	0	0	2
4	0	0	0	0	1
3	2	1	0	0	0
4	2	2	2	2	2
1	2	1	0	0	1
4	2	2	2	2	2
3	2	2	2	2	2
3	2	2	0	2	2
3	2	0	0	0	1
3	2	2	0	2	2
3	1	0	0	0	0
3	2	99	0	0	1
2	2	99	0	0	2
3	2	0	0	0	2
3	2	2	0	2	2
2	2	1	0	0	2
1	1	1	0	2	1
3	2	2	0	2	2
2	2	0	2	2	2
1	2	2	0	2	1
4	2	1	0	0	2
2	2	2	0	2	2
2	2	2	1	2	0
3	2	1	0	2	2

2	2	0	0	0	1
3	1	0	1	0	1
3	2	1	0	1	0
4	1	0	0	0	2
4	1	0	0	0	1
1	2	2	0	2	2
3	2	2	2	2	2
2	1	0	0	0	1
1	1	0	0	0	1
3	1	0	0	0	2
3	1	1	0	0	1
4	2	2	0	0	2
99	1	0	2	0	1
3	1	0	0	0	1
4	1	0	0	0	2
3	1	1	0	0	1
3	2	2	0	0	0
1	0	1	0	0	2
1	2	0	0	0	0
2	2	0	0	0	0
3	2	2	0	0	2
2	2	0	0	0	2
3	1	0	0	0	2
3	2	0	0	0	2
3	2	2	2	2	2
3	2	2	2	2	2
3	2	1	2	1	2
3	2	0	0	2	2
4	2	2	2	2	2
3	2	2	0	2	2
3	2	0	0	0	1
99	2	2	2	2	2
3	2	0	0	0	2
3	2	1	2	0	1
4	2	0	0	0	1
2	1	1	0	0	1
4	1	0	2	0	1
2	1	0	0	0	1
2	1	0	0	0	1
3	99	99	99	99	99
1	1	2	0	0	1
4	1	0	0	0	0
2	1	0	0	2	1
3	1	1	0	2	1
3	1	1	2	2	2
3	2	2	2	2	2
3	1	2	0	0	1
2	2	0	0	2	1

4	1	2	1	0	1
4	2	2	2	2	2
2	0	0	2	0	0
3	1	0	0	0	1
2	1	0	0	0	1
4	1	0	0	0	1
1	0	1	1	1	0
4	1	2	0	2	2
3	1	0	0	0	1
3	2	0	2	0	1
2	2	0	0	0	0
2	1	0	0	0	1
3	2	2	0	0	2
1	99	99	99	99	99
3	1	0	2	2	1
3	1	0	0	0	1
4	1	1	1	1	1
2	1	0	0	0	1
2	1	0	0	0	1
3	1	0	2	0	1
3	1	0	0	0	1
3	2	1	0	2	2
3	1	0	0	2	2
4	2	0	1	0	1
3	2	0	1	0	1
3	1	0	1	0	0
3	1	0	0	2	2
3	0	0	0	0	0
4	#NULL!	#NULL!	#NULL!	#NULL!	#NULL!
4	#NULL!	#NULL!	#NULL!	#NULL!	#NULL!
4	#NULL!	#NULL!	#NULL!	#NULL!	#NULL!
3	#NULL!	#NULL!	#NULL!	#NULL!	#NULL!
3	#NULL!	#NULL!	#NULL!	#NULL!	#NULL!
3	#NULL!	#NULL!	#NULL!	#NULL!	#NULL!
3	#NULL!	#NULL!	#NULL!	#NULL!	#NULL!
4	#NULL!	#NULL!	#NULL!	#NULL!	#NULL!
3	#NULL!	#NULL!	#NULL!	#NULL!	#NULL!
3	#NULL!	#NULL!	#NULL!	#NULL!	#NULL!
4	#NULL!	#NULL!	#NULL!	#NULL!	#NULL!
4	#NULL!	#NULL!	#NULL!	#NULL!	#NULL!
4	#NULL!	#NULL!	#NULL!	#NULL!	#NULL!
2	#NULL!	#NULL!	#NULL!	#NULL!	#NULL!
4	#NULL!	#NULL!	#NULL!	#NULL!	#NULL!
4	#NULL!	#NULL!	#NULL!	#NULL!	#NULL!
4	#NULL!	#NULL!	#NULL!	#NULL!	#NULL!
4	#NULL!	#NULL!	#NULL!	#NULL!	#NULL!
4	#NULL!	#NULL!	#NULL!	#NULL!	#NULL!
3	#NULL!	#NULL!	#NULL!	#NULL!	#NULL!

3	#NULL!	#NULL!	#NULL!	#NULL!	#NULL!
4	#NULL!	#NULL!	#NULL!	#NULL!	#NULL!
4	#NULL!	#NULL!	#NULL!	#NULL!	#NULL!
4	#NULL!	#NULL!	#NULL!	#NULL!	#NULL!
3	#NULL!	#NULL!	#NULL!	#NULL!	#NULL!
3	#NULL!	#NULL!	#NULL!	#NULL!	#NULL!
3	#NULL!	#NULL!	#NULL!	#NULL!	#NULL!
3	#NULL!	#NULL!	#NULL!	#NULL!	#NULL!
3	#NULL!	#NULL!	#NULL!	#NULL!	#NULL!
3	#NULL!	#NULL!	#NULL!	#NULL!	#NULL!
4	#NULL!	#NULL!	#NULL!	#NULL!	#NULL!
4	#NULL!	#NULL!	#NULL!	#NULL!	#NULL!
3	#NULL!	#NULL!	#NULL!	#NULL!	#NULL!
3	#NULL!	#NULL!	#NULL!	#NULL!	#NULL!
2	#NULL!	#NULL!	#NULL!	#NULL!	#NULL!
3	#NULL!	#NULL!	#NULL!	#NULL!	#NULL!
3	#NULL!	#NULL!	#NULL!	#NULL!	#NULL!
4	#NULL!	#NULL!	#NULL!	#NULL!	#NULL!
3	#NULL!	#NULL!	#NULL!	#NULL!	#NULL!
4	#NULL!	#NULL!	#NULL!	#NULL!	#NULL!
3	#NULL!	#NULL!	#NULL!	#NULL!	#NULL!
4	#NULL!	#NULL!	#NULL!	#NULL!	#NULL!
3	#NULL!	#NULL!	#NULL!	#NULL!	#NULL!
2	#NULL!	#NULL!	#NULL!	#NULL!	#NULL!
4	#NULL!	#NULL!	#NULL!	#NULL!	#NULL!
3	#NULL!	#NULL!	#NULL!	#NULL!	#NULL!
3	#NULL!	#NULL!	#NULL!	#NULL!	#NULL!
4	#NULL!	#NULL!	#NULL!	#NULL!	#NULL!
4	#NULL!	#NULL!	#NULL!	#NULL!	#NULL!
2	#NULL!	#NULL!	#NULL!	#NULL!	#NULL!
4	#NULL!	#NULL!	#NULL!	#NULL!	#NULL!
3	#NULL!	#NULL!	#NULL!	#NULL!	#NULL!
3	#NULL!	#NULL!	#NULL!	#NULL!	#NULL!
3	#NULL!	#NULL!	#NULL!	#NULL!	#NULL!
3	#NULL!	#NULL!	#NULL!	#NULL!	#NULL!
4	#NULL!	#NULL!	#NULL!	#NULL!	#NULL!
3	#NULL!	#NULL!	#NULL!	#NULL!	#NULL!
4	#NULL!	#NULL!	#NULL!	#NULL!	#NULL!
1	#NULL!	#NULL!	#NULL!	#NULL!	#NULL!
4	#NULL!	#NULL!	#NULL!	#NULL!	#NULL!
3	#NULL!	#NULL!	#NULL!	#NULL!	#NULL!
4	#NULL!	#NULL!	#NULL!	#NULL!	#NULL!
3	#NULL!	#NULL!	#NULL!	#NULL!	#NULL!
3	#NULL!	#NULL!	#NULL!	#NULL!	#NULL!
3	#NULL!	#NULL!	#NULL!	#NULL!	#NULL!
4	#NULL!	#NULL!	#NULL!	#NULL!	#NULL!
3	#NULL!	#NULL!	#NULL!	#NULL!	#NULL!
3	#NULL!	#NULL!	#NULL!	#NULL!	#NULL!

4	#NULL!	#NULL!	#NULL!	#NULL!	#NULL!
4	#NULL!	#NULL!	#NULL!	#NULL!	#NULL!
3	#NULL!	#NULL!	#NULL!	#NULL!	#NULL!
4	#NULL!	#NULL!	#NULL!	#NULL!	#NULL!
3	#NULL!	#NULL!	#NULL!	#NULL!	#NULL!
3	#NULL!	#NULL!	#NULL!	#NULL!	#NULL!
4	#NULL!	#NULL!	#NULL!	#NULL!	#NULL!
2	#NULL!	#NULL!	#NULL!	#NULL!	#NULL!
4	#NULL!	#NULL!	#NULL!	#NULL!	#NULL!
4	#NULL!	#NULL!	#NULL!	#NULL!	#NULL!
1	#NULL!	#NULL!	#NULL!	#NULL!	#NULL!
3	#NULL!	#NULL!	#NULL!	#NULL!	#NULL!
3	#NULL!	#NULL!	#NULL!	#NULL!	#NULL!
4	#NULL!	#NULL!	#NULL!	#NULL!	#NULL!
3	#NULL!	#NULL!	#NULL!	#NULL!	#NULL!
3	#NULL!	#NULL!	#NULL!	#NULL!	#NULL!
3	#NULL!	#NULL!	#NULL!	#NULL!	#NULL!
3	#NULL!	#NULL!	#NULL!	#NULL!	#NULL!
3	#NULL!	#NULL!	#NULL!	#NULL!	#NULL!
2	#NULL!	#NULL!	#NULL!	#NULL!	#NULL!
2	#NULL!	#NULL!	#NULL!	#NULL!	#NULL!
4	#NULL!	#NULL!	#NULL!	#NULL!	#NULL!
4	#NULL!	#NULL!	#NULL!	#NULL!	#NULL!
4	#NULL!	#NULL!	#NULL!	#NULL!	#NULL!
4	#NULL!	#NULL!	#NULL!	#NULL!	#NULL!
3	#NULL!	#NULL!	#NULL!	#NULL!	#NULL!
4	#NULL!	#NULL!	#NULL!	#NULL!	#NULL!
3	#NULL!	#NULL!	#NULL!	#NULL!	#NULL!
4	#NULL!	#NULL!	#NULL!	#NULL!	#NULL!
3	#NULL!	#NULL!	#NULL!	#NULL!	#NULL!
3	#NULL!	#NULL!	#NULL!	#NULL!	#NULL!
3	#NULL!	#NULL!	#NULL!	#NULL!	#NULL!
3	#NULL!	#NULL!	#NULL!	#NULL!	#NULL!
4	#NULL!	#NULL!	#NULL!	#NULL!	#NULL!
99	#NULL!	#NULL!	#NULL!	#NULL!	#NULL!
2	#NULL!	#NULL!	#NULL!	#NULL!	#NULL!
4	#NULL!	#NULL!	#NULL!	#NULL!	#NULL!
4	#NULL!	#NULL!	#NULL!	#NULL!	#NULL!
3	#NULL!	#NULL!	#NULL!	#NULL!	#NULL!
2	#NULL!	#NULL!	#NULL!	#NULL!	#NULL!
3	#NULL!	#NULL!	#NULL!	#NULL!	#NULL!
3	#NULL!	#NULL!	#NULL!	#NULL!	#NULL!
2	#NULL!	#NULL!	#NULL!	#NULL!	#NULL!
1	#NULL!	#NULL!	#NULL!	#NULL!	#NULL!
3	#NULL!	#NULL!	#NULL!	#NULL!	#NULL!
3	#NULL!	#NULL!	#NULL!	#NULL!	#NULL!
2	#NULL!	#NULL!	#NULL!	#NULL!	#NULL!
3	#NULL!	#NULL!	#NULL!	#NULL!	#NULL!

3	#NULL!	#NULL!	#NULL!	#NULL!	#NULL!
3	#NULL!	#NULL!	#NULL!	#NULL!	#NULL!
3	#NULL!	#NULL!	#NULL!	#NULL!	#NULL!
2	#NULL!	#NULL!	#NULL!	#NULL!	#NULL!
3	#NULL!	#NULL!	#NULL!	#NULL!	#NULL!
3	#NULL!	#NULL!	#NULL!	#NULL!	#NULL!
4	#NULL!	#NULL!	#NULL!	#NULL!	#NULL!
3	#NULL!	#NULL!	#NULL!	#NULL!	#NULL!
4	#NULL!	#NULL!	#NULL!	#NULL!	#NULL!
3	#NULL!	#NULL!	#NULL!	#NULL!	#NULL!
3	#NULL!	#NULL!	#NULL!	#NULL!	#NULL!
4	#NULL!	#NULL!	#NULL!	#NULL!	#NULL!
2	#NULL!	#NULL!	#NULL!	#NULL!	#NULL!
4	#NULL!	#NULL!	#NULL!	#NULL!	#NULL!
4	#NULL!	#NULL!	#NULL!	#NULL!	#NULL!
1	#NULL!	#NULL!	#NULL!	#NULL!	#NULL!
4	#NULL!	#NULL!	#NULL!	#NULL!	#NULL!
4	#NULL!	#NULL!	#NULL!	#NULL!	#NULL!
4	#NULL!	#NULL!	#NULL!	#NULL!	#NULL!
3	#NULL!	#NULL!	#NULL!	#NULL!	#NULL!
3	#NULL!	#NULL!	#NULL!	#NULL!	#NULL!
3	#NULL!	#NULL!	#NULL!	#NULL!	#NULL!
3	#NULL!	#NULL!	#NULL!	#NULL!	#NULL!
4	#NULL!	#NULL!	#NULL!	#NULL!	#NULL!
3	#NULL!	#NULL!	#NULL!	#NULL!	#NULL!
4	#NULL!	#NULL!	#NULL!	#NULL!	#NULL!
3	#NULL!	#NULL!	#NULL!	#NULL!	#NULL!
4	#NULL!	#NULL!	#NULL!	#NULL!	#NULL!
3	#NULL!	#NULL!	#NULL!	#NULL!	#NULL!
4	#NULL!	#NULL!	#NULL!	#NULL!	#NULL!
3	#NULL!	#NULL!	#NULL!	#NULL!	#NULL!
2	#NULL!	#NULL!	#NULL!	#NULL!	#NULL!
2	#NULL!	#NULL!	#NULL!	#NULL!	#NULL!
4	#NULL!	#NULL!	#NULL!	#NULL!	#NULL!
3	#NULL!	#NULL!	#NULL!	#NULL!	#NULL!
4	#NULL!	#NULL!	#NULL!	#NULL!	#NULL!
3	#NULL!	#NULL!	#NULL!	#NULL!	#NULL!
3	#NULL!	#NULL!	#NULL!	#NULL!	#NULL!
4	#NULL!	#NULL!	#NULL!	#NULL!	#NULL!
4	#NULL!	#NULL!	#NULL!	#NULL!	#NULL!
4	#NULL!	#NULL!	#NULL!	#NULL!	#NULL!
2	#NULL!	#NULL!	#NULL!	#NULL!	#NULL!
4	#NULL!	#NULL!	#NULL!	#NULL!	#NULL!
3	#NULL!	#NULL!	#NULL!	#NULL!	#NULL!
4	#NULL!	#NULL!	#NULL!	#NULL!	#NULL!
2	#NULL!	#NULL!	#NULL!	#NULL!	#NULL!
3	#NULL!	#NULL!	#NULL!	#NULL!	#NULL!
1	#NULL!	#NULL!	#NULL!	#NULL!	#NULL!

1	#NULL!	#NULL!	#NULL!	#NULL!	#NULL!
1	#NULL!	#NULL!	#NULL!	#NULL!	#NULL!
2	#NULL!	#NULL!	#NULL!	#NULL!	#NULL!
3	#NULL!	#NULL!	#NULL!	#NULL!	#NULL!
4	#NULL!	#NULL!	#NULL!	#NULL!	#NULL!
3	#NULL!	#NULL!	#NULL!	#NULL!	#NULL!
3	#NULL!	#NULL!	#NULL!	#NULL!	#NULL!
3	#NULL!	#NULL!	#NULL!	#NULL!	#NULL!
3	#NULL!	#NULL!	#NULL!	#NULL!	#NULL!
2	#NULL!	#NULL!	#NULL!	#NULL!	#NULL!
3	#NULL!	#NULL!	#NULL!	#NULL!	#NULL!
4	#NULL!	#NULL!	#NULL!	#NULL!	#NULL!
3	#NULL!	#NULL!	#NULL!	#NULL!	#NULL!
3	#NULL!	#NULL!	#NULL!	#NULL!	#NULL!
4	#NULL!	#NULL!	#NULL!	#NULL!	#NULL!
3	#NULL!	#NULL!	#NULL!	#NULL!	#NULL!
4	#NULL!	#NULL!	#NULL!	#NULL!	#NULL!
3	#NULL!	#NULL!	#NULL!	#NULL!	#NULL!
2	#NULL!	#NULL!	#NULL!	#NULL!	#NULL!
2	#NULL!	#NULL!	#NULL!	#NULL!	#NULL!
1	#NULL!	#NULL!	#NULL!	#NULL!	#NULL!
3	#NULL!	#NULL!	#NULL!	#NULL!	#NULL!
3	#NULL!	#NULL!	#NULL!	#NULL!	#NULL!
4	#NULL!	#NULL!	#NULL!	#NULL!	#NULL!
2	#NULL!	#NULL!	#NULL!	#NULL!	#NULL!
99	#NULL!	#NULL!	#NULL!	#NULL!	#NULL!
1	#NULL!	#NULL!	#NULL!	#NULL!	#NULL!
1	#NULL!	#NULL!	#NULL!	#NULL!	#NULL!
2	#NULL!	#NULL!	#NULL!	#NULL!	#NULL!
2	#NULL!	#NULL!	#NULL!	#NULL!	#NULL!
3	#NULL!	#NULL!	#NULL!	#NULL!	#NULL!
4	#NULL!	#NULL!	#NULL!	#NULL!	#NULL!
2	#NULL!	#NULL!	#NULL!	#NULL!	#NULL!
3	#NULL!	#NULL!	#NULL!	#NULL!	#NULL!
2	#NULL!	#NULL!	#NULL!	#NULL!	#NULL!
3	#NULL!	#NULL!	#NULL!	#NULL!	#NULL!
3	#NULL!	#NULL!	#NULL!	#NULL!	#NULL!
2	#NULL!	#NULL!	#NULL!	#NULL!	#NULL!
3	#NULL!	#NULL!	#NULL!	#NULL!	#NULL!
3	#NULL!	#NULL!	#NULL!	#NULL!	#NULL!
99	#NULL!	#NULL!	#NULL!	#NULL!	#NULL!
3	#NULL!	#NULL!	#NULL!	#NULL!	#NULL!
3	#NULL!	#NULL!	#NULL!	#NULL!	#NULL!
3	#NULL!	#NULL!	#NULL!	#NULL!	#NULL!
4	#NULL!	#NULL!	#NULL!	#NULL!	#NULL!
2	#NULL!	#NULL!	#NULL!	#NULL!	#NULL!
3	#NULL!	#NULL!	#NULL!	#NULL!	#NULL!
3	#NULL!	#NULL!	#NULL!	#NULL!	#NULL!

3	#NULL!	#NULL!	#NULL!	#NULL!	#NULL!
2	#NULL!	#NULL!	#NULL!	#NULL!	#NULL!
2	#NULL!	#NULL!	#NULL!	#NULL!	#NULL!
2	#NULL!	#NULL!	#NULL!	#NULL!	#NULL!
3	#NULL!	#NULL!	#NULL!	#NULL!	#NULL!
4	#NULL!	#NULL!	#NULL!	#NULL!	#NULL!
3	#NULL!	#NULL!	#NULL!	#NULL!	#NULL!
3	#NULL!	#NULL!	#NULL!	#NULL!	#NULL!
2	#NULL!	#NULL!	#NULL!	#NULL!	#NULL!
3	#NULL!	#NULL!	#NULL!	#NULL!	#NULL!
2	#NULL!	#NULL!	#NULL!	#NULL!	#NULL!
1	#NULL!	#NULL!	#NULL!	#NULL!	#NULL!
2	#NULL!	#NULL!	#NULL!	#NULL!	#NULL!
3	#NULL!	#NULL!	#NULL!	#NULL!	#NULL!
99	#NULL!	#NULL!	#NULL!	#NULL!	#NULL!
3	#NULL!	#NULL!	#NULL!	#NULL!	#NULL!
3	#NULL!	#NULL!	#NULL!	#NULL!	#NULL!
3	#NULL!	#NULL!	#NULL!	#NULL!	#NULL!
4	#NULL!	#NULL!	#NULL!	#NULL!	#NULL!
3	#NULL!	#NULL!	#NULL!	#NULL!	#NULL!
3	#NULL!	#NULL!	#NULL!	#NULL!	#NULL!
4	#NULL!	#NULL!	#NULL!	#NULL!	#NULL!
3	#NULL!	#NULL!	#NULL!	#NULL!	#NULL!
3	#NULL!	#NULL!	#NULL!	#NULL!	#NULL!
3	#NULL!	#NULL!	#NULL!	#NULL!	#NULL!
3	#NULL!	#NULL!	#NULL!	#NULL!	#NULL!
4	#NULL!	#NULL!	#NULL!	#NULL!	#NULL!
3	#NULL!	#NULL!	#NULL!	#NULL!	#NULL!
3	#NULL!	#NULL!	#NULL!	#NULL!	#NULL!
2	#NULL!	#NULL!	#NULL!	#NULL!	#NULL!
2	#NULL!	#NULL!	#NULL!	#NULL!	#NULL!
4	#NULL!	#NULL!	#NULL!	#NULL!	#NULL!
3	#NULL!	#NULL!	#NULL!	#NULL!	#NULL!
3	#NULL!	#NULL!	#NULL!	#NULL!	#NULL!
3	#NULL!	#NULL!	#NULL!	#NULL!	#NULL!
3	#NULL!	#NULL!	#NULL!	#NULL!	#NULL!
4	#NULL!	#NULL!	#NULL!	#NULL!	#NULL!
2	#NULL!	#NULL!	#NULL!	#NULL!	#NULL!
4	#NULL!	#NULL!	#NULL!	#NULL!	#NULL!
3	#NULL!	#NULL!	#NULL!	#NULL!	#NULL!
2	#NULL!	#NULL!	#NULL!	#NULL!	#NULL!
4	#NULL!	#NULL!	#NULL!	#NULL!	#NULL!
4	#NULL!	#NULL!	#NULL!	#NULL!	#NULL!
3	#NULL!	#NULL!	#NULL!	#NULL!	#NULL!
3	#NULL!	#NULL!	#NULL!	#NULL!	#NULL!
3	#NULL!	#NULL!	#NULL!	#NULL!	#NULL!
2	#NULL!	#NULL!	#NULL!	#NULL!	#NULL!
4	#NULL!	#NULL!	#NULL!	#NULL!	#NULL!

4	#NULL!	#NULL!	#NULL!	#NULL!	#NULL!
3	#NULL!	#NULL!	#NULL!	#NULL!	#NULL!
4	#NULL!	#NULL!	#NULL!	#NULL!	#NULL!
99	#NULL!	#NULL!	#NULL!	#NULL!	#NULL!
4	#NULL!	#NULL!	#NULL!	#NULL!	#NULL!
4	#NULL!	#NULL!	#NULL!	#NULL!	#NULL!
2	#NULL!	#NULL!	#NULL!	#NULL!	#NULL!
3	#NULL!	#NULL!	#NULL!	#NULL!	#NULL!
2	#NULL!	#NULL!	#NULL!	#NULL!	#NULL!
3	#NULL!	#NULL!	#NULL!	#NULL!	#NULL!
1	#NULL!	#NULL!	#NULL!	#NULL!	#NULL!
3	#NULL!	#NULL!	#NULL!	#NULL!	#NULL!
1	#NULL!	#NULL!	#NULL!	#NULL!	#NULL!
4	#NULL!	#NULL!	#NULL!	#NULL!	#NULL!
3	#NULL!	#NULL!	#NULL!	#NULL!	#NULL!
4	#NULL!	#NULL!	#NULL!	#NULL!	#NULL!
3	#NULL!	#NULL!	#NULL!	#NULL!	#NULL!
2	#NULL!	#NULL!	#NULL!	#NULL!	#NULL!
1	#NULL!	#NULL!	#NULL!	#NULL!	#NULL!
2	#NULL!	#NULL!	#NULL!	#NULL!	#NULL!
3	#NULL!	#NULL!	#NULL!	#NULL!	#NULL!
3	#NULL!	#NULL!	#NULL!	#NULL!	#NULL!
1	#NULL!	#NULL!	#NULL!	#NULL!	#NULL!
2	#NULL!	#NULL!	#NULL!	#NULL!	#NULL!
3	#NULL!	#NULL!	#NULL!	#NULL!	#NULL!
3	#NULL!	#NULL!	#NULL!	#NULL!	#NULL!
3	#NULL!	#NULL!	#NULL!	#NULL!	#NULL!
4	#NULL!	#NULL!	#NULL!	#NULL!	#NULL!
2	#NULL!	#NULL!	#NULL!	#NULL!	#NULL!
2	#NULL!	#NULL!	#NULL!	#NULL!	#NULL!
3	#NULL!	#NULL!	#NULL!	#NULL!	#NULL!
3	#NULL!	#NULL!	#NULL!	#NULL!	#NULL!
1	#NULL!	#NULL!	#NULL!	#NULL!	#NULL!
2	#NULL!	#NULL!	#NULL!	#NULL!	#NULL!
1	#NULL!	#NULL!	#NULL!	#NULL!	#NULL!
4	#NULL!	#NULL!	#NULL!	#NULL!	#NULL!
3	#NULL!	#NULL!	#NULL!	#NULL!	#NULL!
1	#NULL!	#NULL!	#NULL!	#NULL!	#NULL!
3	#NULL!	#NULL!	#NULL!	#NULL!	#NULL!
1	#NULL!	#NULL!	#NULL!	#NULL!	#NULL!
3	#NULL!	#NULL!	#NULL!	#NULL!	#NULL!
4	#NULL!	#NULL!	#NULL!	#NULL!	#NULL!
1	#NULL!	#NULL!	#NULL!	#NULL!	#NULL!
3	#NULL!	#NULL!	#NULL!	#NULL!	#NULL!
3	#NULL!	#NULL!	#NULL!	#NULL!	#NULL!
1	#NULL!	#NULL!	#NULL!	#NULL!	#NULL!
4	#NULL!	#NULL!	#NULL!	#NULL!	#NULL!
99	#NULL!	#NULL!	#NULL!	#NULL!	#NULL!

99	#NULL!	#NULL!	#NULL!	#NULL!	#NULL!
4	#NULL!	#NULL!	#NULL!	#NULL!	#NULL!
2	#NULL!	#NULL!	#NULL!	#NULL!	#NULL!
4	#NULL!	#NULL!	#NULL!	#NULL!	#NULL!
1	#NULL!	#NULL!	#NULL!	#NULL!	#NULL!
2	#NULL!	#NULL!	#NULL!	#NULL!	#NULL!
3	#NULL!	#NULL!	#NULL!	#NULL!	#NULL!
2	#NULL!	#NULL!	#NULL!	#NULL!	#NULL!
3	#NULL!	#NULL!	#NULL!	#NULL!	#NULL!
3	#NULL!	#NULL!	#NULL!	#NULL!	#NULL!
3	#NULL!	#NULL!	#NULL!	#NULL!	#NULL!
4	#NULL!	#NULL!	#NULL!	#NULL!	#NULL!
1	#NULL!	#NULL!	#NULL!	#NULL!	#NULL!
3	#NULL!	#NULL!	#NULL!	#NULL!	#NULL!
1	#NULL!	#NULL!	#NULL!	#NULL!	#NULL!
1	#NULL!	#NULL!	#NULL!	#NULL!	#NULL!
99	#NULL!	#NULL!	#NULL!	#NULL!	#NULL!
1	#NULL!	#NULL!	#NULL!	#NULL!	#NULL!
3	#NULL!	#NULL!	#NULL!	#NULL!	#NULL!
3	#NULL!	#NULL!	#NULL!	#NULL!	#NULL!
3	#NULL!	#NULL!	#NULL!	#NULL!	#NULL!
4	#NULL!	#NULL!	#NULL!	#NULL!	#NULL!
3	#NULL!	#NULL!	#NULL!	#NULL!	#NULL!
3	#NULL!	#NULL!	#NULL!	#NULL!	#NULL!
3	#NULL!	#NULL!	#NULL!	#NULL!	#NULL!
3	#NULL!	#NULL!	#NULL!	#NULL!	#NULL!
3	#NULL!	#NULL!	#NULL!	#NULL!	#NULL!
2	#NULL!	#NULL!	#NULL!	#NULL!	#NULL!
3	#NULL!	#NULL!	#NULL!	#NULL!	#NULL!
3	#NULL!	#NULL!	#NULL!	#NULL!	#NULL!
3	#NULL!	#NULL!	#NULL!	#NULL!	#NULL!
3	#NULL!	#NULL!	#NULL!	#NULL!	#NULL!
3	#NULL!	#NULL!	#NULL!	#NULL!	#NULL!
2	#NULL!	#NULL!	#NULL!	#NULL!	#NULL!
4	#NULL!	#NULL!	#NULL!	#NULL!	#NULL!
3	#NULL!	#NULL!	#NULL!	#NULL!	#NULL!
4	#NULL!	#NULL!	#NULL!	#NULL!	#NULL!
3	#NULL!	#NULL!	#NULL!	#NULL!	#NULL!
4	#NULL!	#NULL!	#NULL!	#NULL!	#NULL!
4	#NULL!	#NULL!	#NULL!	#NULL!	#NULL!
3	#NULL!	#NULL!	#NULL!	#NULL!	#NULL!
4	#NULL!	#NULL!	#NULL!	#NULL!	#NULL!
3	#NULL!	#NULL!	#NULL!	#NULL!	#NULL!
3	#NULL!	#NULL!	#NULL!	#NULL!	#NULL!
4	#NULL!	#NULL!	#NULL!	#NULL!	#NULL!
2	#NULL!	#NULL!	#NULL!	#NULL!	#NULL!
3	#NULL!	#NULL!	#NULL!	#NULL!	#NULL!
2	#NULL!	#NULL!	#NULL!	#NULL!	#NULL!

4	#NULL!	#NULL!	#NULL!	#NULL!	#NULL!
2	#NULL!	#NULL!	#NULL!	#NULL!	#NULL!
3	#NULL!	#NULL!	#NULL!	#NULL!	#NULL!
4	#NULL!	#NULL!	#NULL!	#NULL!	#NULL!
3	#NULL!	#NULL!	#NULL!	#NULL!	#NULL!
3	#NULL!	#NULL!	#NULL!	#NULL!	#NULL!
4	#NULL!	#NULL!	#NULL!	#NULL!	#NULL!
1	#NULL!	#NULL!	#NULL!	#NULL!	#NULL!
3	#NULL!	#NULL!	#NULL!	#NULL!	#NULL!
2	#NULL!	#NULL!	#NULL!	#NULL!	#NULL!
3	#NULL!	#NULL!	#NULL!	#NULL!	#NULL!
2	#NULL!	#NULL!	#NULL!	#NULL!	#NULL!
3	#NULL!	#NULL!	#NULL!	#NULL!	#NULL!
4	#NULL!	#NULL!	#NULL!	#NULL!	#NULL!
3	#NULL!	#NULL!	#NULL!	#NULL!	#NULL!
1	#NULL!	#NULL!	#NULL!	#NULL!	#NULL!
4	#NULL!	#NULL!	#NULL!	#NULL!	#NULL!
1	#NULL!	#NULL!	#NULL!	#NULL!	#NULL!
3	#NULL!	#NULL!	#NULL!	#NULL!	#NULL!
3	#NULL!	#NULL!	#NULL!	#NULL!	#NULL!
3	#NULL!	#NULL!	#NULL!	#NULL!	#NULL!
4	#NULL!	#NULL!	#NULL!	#NULL!	#NULL!
3	#NULL!	#NULL!	#NULL!	#NULL!	#NULL!
99	#NULL!	#NULL!	#NULL!	#NULL!	#NULL!
3	#NULL!	#NULL!	#NULL!	#NULL!	#NULL!
1	#NULL!	#NULL!	#NULL!	#NULL!	#NULL!
2	#NULL!	#NULL!	#NULL!	#NULL!	#NULL!
3	#NULL!	#NULL!	#NULL!	#NULL!	#NULL!
4	#NULL!	#NULL!	#NULL!	#NULL!	#NULL!
3	#NULL!	#NULL!	#NULL!	#NULL!	#NULL!
3	#NULL!	#NULL!	#NULL!	#NULL!	#NULL!
99	#NULL!	#NULL!	#NULL!	#NULL!	#NULL!
3	#NULL!	#NULL!	#NULL!	#NULL!	#NULL!
3	#NULL!	#NULL!	#NULL!	#NULL!	#NULL!
3	#NULL!	#NULL!	#NULL!	#NULL!	#NULL!
3	#NULL!	#NULL!	#NULL!	#NULL!	#NULL!
2	#NULL!	#NULL!	#NULL!	#NULL!	#NULL!
4	#NULL!	#NULL!	#NULL!	#NULL!	#NULL!
3	#NULL!	#NULL!	#NULL!	#NULL!	#NULL!
3	#NULL!	#NULL!	#NULL!	#NULL!	#NULL!
3	#NULL!	#NULL!	#NULL!	#NULL!	#NULL!
2	#NULL!	#NULL!	#NULL!	#NULL!	#NULL!
3	#NULL!	#NULL!	#NULL!	#NULL!	#NULL!
3	#NULL!	#NULL!	#NULL!	#NULL!	#NULL!
3	#NULL!	#NULL!	#NULL!	#NULL!	#NULL!
1	#NULL!	#NULL!	#NULL!	#NULL!	#NULL!
3	#NULL!	#NULL!	#NULL!	#NULL!	#NULL!
3	#NULL!	#NULL!	#NULL!	#NULL!	#NULL!

1	#NULL!	#NULL!	#NULL!	#NULL!	#NULL!
4	#NULL!	#NULL!	#NULL!	#NULL!	#NULL!
4	#NULL!	#NULL!	#NULL!	#NULL!	#NULL!
3	#NULL!	#NULL!	#NULL!	#NULL!	#NULL!
3	#NULL!	#NULL!	#NULL!	#NULL!	#NULL!
3	#NULL!	#NULL!	#NULL!	#NULL!	#NULL!
4	#NULL!	#NULL!	#NULL!	#NULL!	#NULL!
1	#NULL!	#NULL!	#NULL!	#NULL!	#NULL!
1	#NULL!	#NULL!	#NULL!	#NULL!	#NULL!
2	#NULL!	#NULL!	#NULL!	#NULL!	#NULL!
1	#NULL!	#NULL!	#NULL!	#NULL!	#NULL!
3	#NULL!	#NULL!	#NULL!	#NULL!	#NULL!
3	#NULL!	#NULL!	#NULL!	#NULL!	#NULL!
2	#NULL!	#NULL!	#NULL!	#NULL!	#NULL!
3	#NULL!	#NULL!	#NULL!	#NULL!	#NULL!
1	#NULL!	#NULL!	#NULL!	#NULL!	#NULL!
1	#NULL!	#NULL!	#NULL!	#NULL!	#NULL!
1	#NULL!	#NULL!	#NULL!	#NULL!	#NULL!
3	#NULL!	#NULL!	#NULL!	#NULL!	#NULL!
1	#NULL!	#NULL!	#NULL!	#NULL!	#NULL!
3	#NULL!	#NULL!	#NULL!	#NULL!	#NULL!
3	#NULL!	#NULL!	#NULL!	#NULL!	#NULL!
2	#NULL!	#NULL!	#NULL!	#NULL!	#NULL!
3	#NULL!	#NULL!	#NULL!	#NULL!	#NULL!
3	#NULL!	#NULL!	#NULL!	#NULL!	#NULL!
2	#NULL!	#NULL!	#NULL!	#NULL!	#NULL!
3	#NULL!	#NULL!	#NULL!	#NULL!	#NULL!
4	#NULL!	#NULL!	#NULL!	#NULL!	#NULL!
3	#NULL!	#NULL!	#NULL!	#NULL!	#NULL!
3	#NULL!	#NULL!	#NULL!	#NULL!	#NULL!
3	#NULL!	#NULL!	#NULL!	#NULL!	#NULL!
2	#NULL!	#NULL!	#NULL!	#NULL!	#NULL!
3	#NULL!	#NULL!	#NULL!	#NULL!	#NULL!
2	#NULL!	#NULL!	#NULL!	#NULL!	#NULL!
2	#NULL!	#NULL!	#NULL!	#NULL!	#NULL!
3	#NULL!	#NULL!	#NULL!	#NULL!	#NULL!
1	#NULL!	#NULL!	#NULL!	#NULL!	#NULL!
3	#NULL!	#NULL!	#NULL!	#NULL!	#NULL!
4	#NULL!	#NULL!	#NULL!	#NULL!	#NULL!
3	#NULL!	#NULL!	#NULL!	#NULL!	#NULL!
2	#NULL!	#NULL!	#NULL!	#NULL!	#NULL!
4	#NULL!	#NULL!	#NULL!	#NULL!	#NULL!
3	#NULL!	#NULL!	#NULL!	#NULL!	#NULL!
3	#NULL!	#NULL!	#NULL!	#NULL!	#NULL!
3	#NULL!	#NULL!	#NULL!	#NULL!	#NULL!
4	#NULL!	#NULL!	#NULL!	#NULL!	#NULL!
4	#NULL!	#NULL!	#NULL!	#NULL!	#NULL!
4	#NULL!	#NULL!	#NULL!	#NULL!	#NULL!

3	#NULL!	#NULL!	#NULL!	#NULL!	#NULL!
3	#NULL!	#NULL!	#NULL!	#NULL!	#NULL!
2	#NULL!	#NULL!	#NULL!	#NULL!	#NULL!
3	#NULL!	#NULL!	#NULL!	#NULL!	#NULL!
2	#NULL!	#NULL!	#NULL!	#NULL!	#NULL!
3	#NULL!	#NULL!	#NULL!	#NULL!	#NULL!
3	#NULL!	#NULL!	#NULL!	#NULL!	#NULL!
3	#NULL!	#NULL!	#NULL!	#NULL!	#NULL!
4	#NULL!	#NULL!	#NULL!	#NULL!	#NULL!
3	#NULL!	#NULL!	#NULL!	#NULL!	#NULL!
2	#NULL!	#NULL!	#NULL!	#NULL!	#NULL!
3	#NULL!	#NULL!	#NULL!	#NULL!	#NULL!
1	#NULL!	#NULL!	#NULL!	#NULL!	#NULL!
4	#NULL!	#NULL!	#NULL!	#NULL!	#NULL!
3	#NULL!	#NULL!	#NULL!	#NULL!	#NULL!
1	#NULL!	#NULL!	#NULL!	#NULL!	#NULL!
2	#NULL!	#NULL!	#NULL!	#NULL!	#NULL!
2	#NULL!	#NULL!	#NULL!	#NULL!	#NULL!
2	#NULL!	#NULL!	#NULL!	#NULL!	#NULL!
99	#NULL!	#NULL!	#NULL!	#NULL!	#NULL!
2	#NULL!	#NULL!	#NULL!	#NULL!	#NULL!
3	#NULL!	#NULL!	#NULL!	#NULL!	#NULL!
2	#NULL!	#NULL!	#NULL!	#NULL!	#NULL!
2	#NULL!	#NULL!	#NULL!	#NULL!	#NULL!
3	#NULL!	#NULL!	#NULL!	#NULL!	#NULL!
3	#NULL!	#NULL!	#NULL!	#NULL!	#NULL!
3	#NULL!	#NULL!	#NULL!	#NULL!	#NULL!
3	#NULL!	#NULL!	#NULL!	#NULL!	#NULL!
3	#NULL!	#NULL!	#NULL!	#NULL!	#NULL!
1	#NULL!	#NULL!	#NULL!	#NULL!	#NULL!
3	#NULL!	#NULL!	#NULL!	#NULL!	#NULL!
3	#NULL!	#NULL!	#NULL!	#NULL!	#NULL!
3	#NULL!	#NULL!	#NULL!	#NULL!	#NULL!
3	#NULL!	#NULL!	#NULL!	#NULL!	#NULL!
1	#NULL!	#NULL!	#NULL!	#NULL!	#NULL!
1	#NULL!	#NULL!	#NULL!	#NULL!	#NULL!
3	#NULL!	#NULL!	#NULL!	#NULL!	#NULL!
2	#NULL!	#NULL!	#NULL!	#NULL!	#NULL!
3	#NULL!	#NULL!	#NULL!	#NULL!	#NULL!
3	#NULL!	#NULL!	#NULL!	#NULL!	#NULL!
1	#NULL!	#NULL!	#NULL!	#NULL!	#NULL!
4	#NULL!	#NULL!	#NULL!	#NULL!	#NULL!
3	#NULL!	#NULL!	#NULL!	#NULL!	#NULL!
3	#NULL!	#NULL!	#NULL!	#NULL!	#NULL!
3	#NULL!	#NULL!	#NULL!	#NULL!	#NULL!
3	#NULL!	#NULL!	#NULL!	#NULL!	#NULL!
1	#NULL!	#NULL!	#NULL!	#NULL!	#NULL!
1	#NULL!	#NULL!	#NULL!	#NULL!	#NULL!

3	#NULL!	#NULL!	#NULL!	#NULL!	#NULL!
3	#NULL!	#NULL!	#NULL!	#NULL!	#NULL!
3	#NULL!	#NULL!	#NULL!	#NULL!	#NULL!
3	#NULL!	#NULL!	#NULL!	#NULL!	#NULL!
3	#NULL!	#NULL!	#NULL!	#NULL!	#NULL!
3	#NULL!	#NULL!	#NULL!	#NULL!	#NULL!
1	#NULL!	#NULL!	#NULL!	#NULL!	#NULL!
4	#NULL!	#NULL!	#NULL!	#NULL!	#NULL!
4	#NULL!	#NULL!	#NULL!	#NULL!	#NULL!
4	#NULL!	#NULL!	#NULL!	#NULL!	#NULL!
3	#NULL!	#NULL!	#NULL!	#NULL!	#NULL!
2	#NULL!	#NULL!	#NULL!	#NULL!	#NULL!
2	#NULL!	#NULL!	#NULL!	#NULL!	#NULL!
4	#NULL!	#NULL!	#NULL!	#NULL!	#NULL!
3	#NULL!	#NULL!	#NULL!	#NULL!	#NULL!
2	#NULL!	#NULL!	#NULL!	#NULL!	#NULL!
3	#NULL!	#NULL!	#NULL!	#NULL!	#NULL!
2	#NULL!	#NULL!	#NULL!	#NULL!	#NULL!
3	#NULL!	#NULL!	#NULL!	#NULL!	#NULL!
99	#NULL!	#NULL!	#NULL!	#NULL!	#NULL!
4	#NULL!	#NULL!	#NULL!	#NULL!	#NULL!
3	#NULL!	#NULL!	#NULL!	#NULL!	#NULL!
3	#NULL!	#NULL!	#NULL!	#NULL!	#NULL!
2	#NULL!	#NULL!	#NULL!	#NULL!	#NULL!
1	#NULL!	#NULL!	#NULL!	#NULL!	#NULL!
2	#NULL!	#NULL!	#NULL!	#NULL!	#NULL!
3	#NULL!	#NULL!	#NULL!	#NULL!	#NULL!
3	#NULL!	#NULL!	#NULL!	#NULL!	#NULL!
3	#NULL!	#NULL!	#NULL!	#NULL!	#NULL!
3	#NULL!	#NULL!	#NULL!	#NULL!	#NULL!
2	#NULL!	#NULL!	#NULL!	#NULL!	#NULL!
3	#NULL!	#NULL!	#NULL!	#NULL!	#NULL!
2	#NULL!	#NULL!	#NULL!	#NULL!	#NULL!
3	#NULL!	#NULL!	#NULL!	#NULL!	#NULL!
3	#NULL!	#NULL!	#NULL!	#NULL!	#NULL!
1	#NULL!	#NULL!	#NULL!	#NULL!	#NULL!
3	#NULL!	#NULL!	#NULL!	#NULL!	#NULL!
1	#NULL!	#NULL!	#NULL!	#NULL!	#NULL!
1	#NULL!	#NULL!	#NULL!	#NULL!	#NULL!
2	#NULL!	#NULL!	#NULL!	#NULL!	#NULL!
1	#NULL!	#NULL!	#NULL!	#NULL!	#NULL!
1	#NULL!	#NULL!	#NULL!	#NULL!	#NULL!
4	#NULL!	#NULL!	#NULL!	#NULL!	#NULL!
2	#NULL!	#NULL!	#NULL!	#NULL!	#NULL!
3	#NULL!	#NULL!	#NULL!	#NULL!	#NULL!
3	#NULL!	#NULL!	#NULL!	#NULL!	#NULL!
3	#NULL!	#NULL!	#NULL!	#NULL!	#NULL!
1	#NULL!	#NULL!	#NULL!	#NULL!	#NULL!

3	#NULL!	#NULL!	#NULL!	#NULL!	#NULL!
3	#NULL!	#NULL!	#NULL!	#NULL!	#NULL!
2	#NULL!	#NULL!	#NULL!	#NULL!	#NULL!
4	#NULL!	#NULL!	#NULL!	#NULL!	#NULL!
1	#NULL!	#NULL!	#NULL!	#NULL!	#NULL!
1	#NULL!	#NULL!	#NULL!	#NULL!	#NULL!
2	#NULL!	#NULL!	#NULL!	#NULL!	#NULL!
99	#NULL!	#NULL!	#NULL!	#NULL!	#NULL!
3	#NULL!	#NULL!	#NULL!	#NULL!	#NULL!
4	#NULL!	#NULL!	#NULL!	#NULL!	#NULL!
3	#NULL!	#NULL!	#NULL!	#NULL!	#NULL!
1	#NULL!	#NULL!	#NULL!	#NULL!	#NULL!
4	#NULL!	#NULL!	#NULL!	#NULL!	#NULL!
4	#NULL!	#NULL!	#NULL!	#NULL!	#NULL!
3	#NULL!	#NULL!	#NULL!	#NULL!	#NULL!
3	#NULL!	#NULL!	#NULL!	#NULL!	#NULL!
3	#NULL!	#NULL!	#NULL!	#NULL!	#NULL!
3	#NULL!	#NULL!	#NULL!	#NULL!	#NULL!
3	#NULL!	#NULL!	#NULL!	#NULL!	#NULL!
3	#NULL!	#NULL!	#NULL!	#NULL!	#NULL!
3	#NULL!	#NULL!	#NULL!	#NULL!	#NULL!
3	#NULL!	#NULL!	#NULL!	#NULL!	#NULL!
2	#NULL!	#NULL!	#NULL!	#NULL!	#NULL!
99	#NULL!	#NULL!	#NULL!	#NULL!	#NULL!
4	#NULL!	#NULL!	#NULL!	#NULL!	#NULL!
2	#NULL!	#NULL!	#NULL!	#NULL!	#NULL!
3	#NULL!	#NULL!	#NULL!	#NULL!	#NULL!
3	#NULL!	#NULL!	#NULL!	#NULL!	#NULL!
3	#NULL!	#NULL!	#NULL!	#NULL!	#NULL!
3	#NULL!	#NULL!	#NULL!	#NULL!	#NULL!
3	#NULL!	#NULL!	#NULL!	#NULL!	#NULL!
2	#NULL!	#NULL!	#NULL!	#NULL!	#NULL!
4	#NULL!	#NULL!	#NULL!	#NULL!	#NULL!
3	#NULL!	#NULL!	#NULL!	#NULL!	#NULL!
2	#NULL!	#NULL!	#NULL!	#NULL!	#NULL!
2	#NULL!	#NULL!	#NULL!	#NULL!	#NULL!
99	#NULL!	#NULL!	#NULL!	#NULL!	#NULL!
3	#NULL!	#NULL!	#NULL!	#NULL!	#NULL!
99	#NULL!	#NULL!	#NULL!	#NULL!	#NULL!
4	#NULL!	#NULL!	#NULL!	#NULL!	#NULL!
4	#NULL!	#NULL!	#NULL!	#NULL!	#NULL!
4	#NULL!	#NULL!	#NULL!	#NULL!	#NULL!
2	#NULL!	#NULL!	#NULL!	#NULL!	#NULL!
1	#NULL!	#NULL!	#NULL!	#NULL!	#NULL!
4	#NULL!	#NULL!	#NULL!	#NULL!	#NULL!
3	#NULL!	#NULL!	#NULL!	#NULL!	#NULL!
4	#NULL!	#NULL!	#NULL!	#NULL!	#NULL!
2	#NULL!	#NULL!	#NULL!	#NULL!	#NULL!

3	#NULL!	#NULL!	#NULL!	#NULL!	#NULL!
3	#NULL!	#NULL!	#NULL!	#NULL!	#NULL!
1	#NULL!	#NULL!	#NULL!	#NULL!	#NULL!
1	#NULL!	#NULL!	#NULL!	#NULL!	#NULL!
3	#NULL!	#NULL!	#NULL!	#NULL!	#NULL!
3	#NULL!	#NULL!	#NULL!	#NULL!	#NULL!
3	#NULL!	#NULL!	#NULL!	#NULL!	#NULL!
2	#NULL!	#NULL!	#NULL!	#NULL!	#NULL!
2	#NULL!	#NULL!	#NULL!	#NULL!	#NULL!
3	#NULL!	#NULL!	#NULL!	#NULL!	#NULL!
3	#NULL!	#NULL!	#NULL!	#NULL!	#NULL!
3	#NULL!	#NULL!	#NULL!	#NULL!	#NULL!
2	#NULL!	#NULL!	#NULL!	#NULL!	#NULL!
3	#NULL!	#NULL!	#NULL!	#NULL!	#NULL!
1	#NULL!	#NULL!	#NULL!	#NULL!	#NULL!
2	#NULL!	#NULL!	#NULL!	#NULL!	#NULL!
4	#NULL!	#NULL!	#NULL!	#NULL!	#NULL!
2	#NULL!	#NULL!	#NULL!	#NULL!	#NULL!
3	#NULL!	#NULL!	#NULL!	#NULL!	#NULL!
3	#NULL!	#NULL!	#NULL!	#NULL!	#NULL!
3	#NULL!	#NULL!	#NULL!	#NULL!	#NULL!
1	#NULL!	#NULL!	#NULL!	#NULL!	#NULL!
2	#NULL!	#NULL!	#NULL!	#NULL!	#NULL!
3	#NULL!	#NULL!	#NULL!	#NULL!	#NULL!
1	#NULL!	#NULL!	#NULL!	#NULL!	#NULL!
2	#NULL!	#NULL!	#NULL!	#NULL!	#NULL!
4	#NULL!	#NULL!	#NULL!	#NULL!	#NULL!
3	#NULL!	#NULL!	#NULL!	#NULL!	#NULL!
3	#NULL!	#NULL!	#NULL!	#NULL!	#NULL!
3	#NULL!	#NULL!	#NULL!	#NULL!	#NULL!
3	#NULL!	#NULL!	#NULL!	#NULL!	#NULL!
2	#NULL!	#NULL!	#NULL!	#NULL!	#NULL!
2	#NULL!	#NULL!	#NULL!	#NULL!	#NULL!
2	#NULL!	#NULL!	#NULL!	#NULL!	#NULL!
3	#NULL!	#NULL!	#NULL!	#NULL!	#NULL!
3	#NULL!	#NULL!	#NULL!	#NULL!	#NULL!
1	#NULL!	#NULL!	#NULL!	#NULL!	#NULL!
3	#NULL!	#NULL!	#NULL!	#NULL!	#NULL!
3	#NULL!	#NULL!	#NULL!	#NULL!	#NULL!
3	#NULL!	#NULL!	#NULL!	#NULL!	#NULL!
3	#NULL!	#NULL!	#NULL!	#NULL!	#NULL!
4	#NULL!	#NULL!	#NULL!	#NULL!	#NULL!
3	#NULL!	#NULL!	#NULL!	#NULL!	#NULL!
2	#NULL!	#NULL!	#NULL!	#NULL!	#NULL!
1	#NULL!	#NULL!	#NULL!	#NULL!	#NULL!
1	#NULL!	#NULL!	#NULL!	#NULL!	#NULL!
4	#NULL!	#NULL!	#NULL!	#NULL!	#NULL!
4	#NULL!	#NULL!	#NULL!	#NULL!	#NULL!

3	#NULL!	#NULL!	#NULL!	#NULL!	#NULL!
2	#NULL!	#NULL!	#NULL!	#NULL!	#NULL!
1	#NULL!	#NULL!	#NULL!	#NULL!	#NULL!
4	#NULL!	#NULL!	#NULL!	#NULL!	#NULL!
4	#NULL!	#NULL!	#NULL!	#NULL!	#NULL!
4	#NULL!	#NULL!	#NULL!	#NULL!	#NULL!
4	#NULL!	#NULL!	#NULL!	#NULL!	#NULL!
3	#NULL!	#NULL!	#NULL!	#NULL!	#NULL!
4	#NULL!	#NULL!	#NULL!	#NULL!	#NULL!
3	#NULL!	#NULL!	#NULL!	#NULL!	#NULL!
4	#NULL!	#NULL!	#NULL!	#NULL!	#NULL!
4	#NULL!	#NULL!	#NULL!	#NULL!	#NULL!
4	#NULL!	#NULL!	#NULL!	#NULL!	#NULL!
2	#NULL!	#NULL!	#NULL!	#NULL!	#NULL!
4	#NULL!	#NULL!	#NULL!	#NULL!	#NULL!
4	#NULL!	#NULL!	#NULL!	#NULL!	#NULL!
4	#NULL!	#NULL!	#NULL!	#NULL!	#NULL!
4	#NULL!	#NULL!	#NULL!	#NULL!	#NULL!
3	#NULL!	#NULL!	#NULL!	#NULL!	#NULL!
4	#NULL!	#NULL!	#NULL!	#NULL!	#NULL!
4	#NULL!	#NULL!	#NULL!	#NULL!	#NULL!
4	#NULL!	#NULL!	#NULL!	#NULL!	#NULL!
4	#NULL!	#NULL!	#NULL!	#NULL!	#NULL!
3	#NULL!	#NULL!	#NULL!	#NULL!	#NULL!
4	#NULL!	#NULL!	#NULL!	#NULL!	#NULL!
4	#NULL!	#NULL!	#NULL!	#NULL!	#NULL!
4	#NULL!	#NULL!	#NULL!	#NULL!	#NULL!
4	#NULL!	#NULL!	#NULL!	#NULL!	#NULL!
3	#NULL!	#NULL!	#NULL!	#NULL!	#NULL!
4	#NULL!	#NULL!	#NULL!	#NULL!	#NULL!
4	#NULL!	#NULL!	#NULL!	#NULL!	#NULL!
3	#NULL!	#NULL!	#NULL!	#NULL!	#NULL!
3	#NULL!	#NULL!	#NULL!	#NULL!	#NULL!
3	#NULL!	#NULL!	#NULL!	#NULL!	#NULL!
3	#NULL!	#NULL!	#NULL!	#NULL!	#NULL!
4	#NULL!	#NULL!	#NULL!	#NULL!	#NULL!
1	#NULL!	#NULL!	#NULL!	#NULL!	#NULL!
4	#NULL!	#NULL!	#NULL!	#NULL!	#NULL!
4	#NULL!	#NULL!	#NULL!	#NULL!	#NULL!
3	#NULL!	#NULL!	#NULL!	#NULL!	#NULL!
3	#NULL!	#NULL!	#NULL!	#NULL!	#NULL!
4	#NULL!	#NULL!	#NULL!	#NULL!	#NULL!
3	#NULL!	#NULL!	#NULL!	#NULL!	#NULL!
4	#NULL!	#NULL!	#NULL!	#NULL!	#NULL!
3	#NULL!	#NULL!	#NULL!	#NULL!	#NULL!
4	#NULL!	#NULL!	#NULL!	#NULL!	#NULL!
3	#NULL!	#NULL!	#NULL!	#NULL!	#NULL!
3	#NULL!	#NULL!	#NULL!	#NULL!	#NULL!

2	#NULL!	#NULL!	#NULL!	#NULL!	#NULL!
2	#NULL!	#NULL!	#NULL!	#NULL!	#NULL!
3	#NULL!	#NULL!	#NULL!	#NULL!	#NULL!
4	#NULL!	#NULL!	#NULL!	#NULL!	#NULL!
3	#NULL!	#NULL!	#NULL!	#NULL!	#NULL!
99	#NULL!	#NULL!	#NULL!	#NULL!	#NULL!
3	#NULL!	#NULL!	#NULL!	#NULL!	#NULL!
3	#NULL!	#NULL!	#NULL!	#NULL!	#NULL!
3	#NULL!	#NULL!	#NULL!	#NULL!	#NULL!
4	#NULL!	#NULL!	#NULL!	#NULL!	#NULL!
4	#NULL!	#NULL!	#NULL!	#NULL!	#NULL!
3	#NULL!	#NULL!	#NULL!	#NULL!	#NULL!
3	#NULL!	#NULL!	#NULL!	#NULL!	#NULL!
3	#NULL!	#NULL!	#NULL!	#NULL!	#NULL!
4	#NULL!	#NULL!	#NULL!	#NULL!	#NULL!
3	#NULL!	#NULL!	#NULL!	#NULL!	#NULL!
3	#NULL!	#NULL!	#NULL!	#NULL!	#NULL!
3	#NULL!	#NULL!	#NULL!	#NULL!	#NULL!
3	#NULL!	#NULL!	#NULL!	#NULL!	#NULL!
2	#NULL!	#NULL!	#NULL!	#NULL!	#NULL!
3	#NULL!	#NULL!	#NULL!	#NULL!	#NULL!
3	#NULL!	#NULL!	#NULL!	#NULL!	#NULL!
3	#NULL!	#NULL!	#NULL!	#NULL!	#NULL!
3	#NULL!	#NULL!	#NULL!	#NULL!	#NULL!
4	#NULL!	#NULL!	#NULL!	#NULL!	#NULL!
2	#NULL!	#NULL!	#NULL!	#NULL!	#NULL!
3	#NULL!	#NULL!	#NULL!	#NULL!	#NULL!
99	#NULL!	#NULL!	#NULL!	#NULL!	#NULL!
3	#NULL!	#NULL!	#NULL!	#NULL!	#NULL!
3	#NULL!	#NULL!	#NULL!	#NULL!	#NULL!
3	#NULL!	#NULL!	#NULL!	#NULL!	#NULL!
2	#NULL!	#NULL!	#NULL!	#NULL!	#NULL!
1	#NULL!	#NULL!	#NULL!	#NULL!	#NULL!
3	#NULL!	#NULL!	#NULL!	#NULL!	#NULL!
3	#NULL!	#NULL!	#NULL!	#NULL!	#NULL!
3	#NULL!	#NULL!	#NULL!	#NULL!	#NULL!
99	#NULL!	#NULL!	#NULL!	#NULL!	#NULL!
3	#NULL!	#NULL!	#NULL!	#NULL!	#NULL!
3	#NULL!	#NULL!	#NULL!	#NULL!	#NULL!
3	#NULL!	#NULL!	#NULL!	#NULL!	#NULL!
2	#NULL!	#NULL!	#NULL!	#NULL!	#NULL!
4	#NULL!	#NULL!	#NULL!	#NULL!	#NULL!
4	#NULL!	#NULL!	#NULL!	#NULL!	#NULL!
3	#NULL!	#NULL!	#NULL!	#NULL!	#NULL!
3	#NULL!	#NULL!	#NULL!	#NULL!	#NULL!
4	#NULL!	#NULL!	#NULL!	#NULL!	#NULL!
3	#NULL!	#NULL!	#NULL!	#NULL!	#NULL!
3	#NULL!	#NULL!	#NULL!	#NULL!	#NULL!

3	#NULL!	#NULL!	#NULL!	#NULL!	#NULL!
3	#NULL!	#NULL!	#NULL!	#NULL!	#NULL!
4	#NULL!	#NULL!	#NULL!	#NULL!	#NULL!
3	#NULL!	#NULL!	#NULL!	#NULL!	#NULL!
4	#NULL!	#NULL!	#NULL!	#NULL!	#NULL!
3	#NULL!	#NULL!	#NULL!	#NULL!	#NULL!
4	#NULL!	#NULL!	#NULL!	#NULL!	#NULL!
3	#NULL!	#NULL!	#NULL!	#NULL!	#NULL!
3	#NULL!	#NULL!	#NULL!	#NULL!	#NULL!
1	#NULL!	#NULL!	#NULL!	#NULL!	#NULL!
4	#NULL!	#NULL!	#NULL!	#NULL!	#NULL!
4	#NULL!	#NULL!	#NULL!	#NULL!	#NULL!
4	#NULL!	#NULL!	#NULL!	#NULL!	#NULL!
2	#NULL!	#NULL!	#NULL!	#NULL!	#NULL!
4	#NULL!	#NULL!	#NULL!	#NULL!	#NULL!
1	#NULL!	#NULL!	#NULL!	#NULL!	#NULL!
4	#NULL!	#NULL!	#NULL!	#NULL!	#NULL!
3	#NULL!	#NULL!	#NULL!	#NULL!	#NULL!
3	#NULL!	#NULL!	#NULL!	#NULL!	#NULL!
4	#NULL!	#NULL!	#NULL!	#NULL!	#NULL!
3	#NULL!	#NULL!	#NULL!	#NULL!	#NULL!
3	#NULL!	#NULL!	#NULL!	#NULL!	#NULL!
99	#NULL!	#NULL!	#NULL!	#NULL!	#NULL!
3	#NULL!	#NULL!	#NULL!	#NULL!	#NULL!
4	#NULL!	#NULL!	#NULL!	#NULL!	#NULL!
3	#NULL!	#NULL!	#NULL!	#NULL!	#NULL!
4	#NULL!	#NULL!	#NULL!	#NULL!	#NULL!
3	#NULL!	#NULL!	#NULL!	#NULL!	#NULL!
3	#NULL!	#NULL!	#NULL!	#NULL!	#NULL!
3	#NULL!	#NULL!	#NULL!	#NULL!	#NULL!
3	#NULL!	#NULL!	#NULL!	#NULL!	#NULL!
3	#NULL!	#NULL!	#NULL!	#NULL!	#NULL!
4	#NULL!	#NULL!	#NULL!	#NULL!	#NULL!
4	#NULL!	#NULL!	#NULL!	#NULL!	#NULL!
3	#NULL!	#NULL!	#NULL!	#NULL!	#NULL!
99	#NULL!	#NULL!	#NULL!	#NULL!	#NULL!
3	#NULL!	#NULL!	#NULL!	#NULL!	#NULL!
99	#NULL!	#NULL!	#NULL!	#NULL!	#NULL!
2	#NULL!	#NULL!	#NULL!	#NULL!	#NULL!
3	#NULL!	#NULL!	#NULL!	#NULL!	#NULL!
3	#NULL!	#NULL!	#NULL!	#NULL!	#NULL!
2	#NULL!	#NULL!	#NULL!	#NULL!	#NULL!
3	#NULL!	#NULL!	#NULL!	#NULL!	#NULL!
3	#NULL!	#NULL!	#NULL!	#NULL!	#NULL!
3	#NULL!	#NULL!	#NULL!	#NULL!	#NULL!
3	#NULL!	#NULL!	#NULL!	#NULL!	#NULL!
4	#NULL!	#NULL!	#NULL!	#NULL!	#NULL!
2	#NULL!	#NULL!	#NULL!	#NULL!	#NULL!

99	#NULL!	#NULL!	#NULL!	#NULL!	#NULL!
3	#NULL!	#NULL!	#NULL!	#NULL!	#NULL!
4	#NULL!	#NULL!	#NULL!	#NULL!	#NULL!
4	#NULL!	#NULL!	#NULL!	#NULL!	#NULL!
4	#NULL!	#NULL!	#NULL!	#NULL!	#NULL!
3	#NULL!	#NULL!	#NULL!	#NULL!	#NULL!
4	#NULL!	#NULL!	#NULL!	#NULL!	#NULL!
3	#NULL!	#NULL!	#NULL!	#NULL!	#NULL!
3	#NULL!	#NULL!	#NULL!	#NULL!	#NULL!
4	#NULL!	#NULL!	#NULL!	#NULL!	#NULL!
3	#NULL!	#NULL!	#NULL!	#NULL!	#NULL!
3	#NULL!	#NULL!	#NULL!	#NULL!	#NULL!
4	#NULL!	#NULL!	#NULL!	#NULL!	#NULL!
3	#NULL!	#NULL!	#NULL!	#NULL!	#NULL!
3	#NULL!	#NULL!	#NULL!	#NULL!	#NULL!
2	#NULL!	#NULL!	#NULL!	#NULL!	#NULL!
4	#NULL!	#NULL!	#NULL!	#NULL!	#NULL!
2	#NULL!	#NULL!	#NULL!	#NULL!	#NULL!
4	#NULL!	#NULL!	#NULL!	#NULL!	#NULL!
3	#NULL!	#NULL!	#NULL!	#NULL!	#NULL!
3	#NULL!	#NULL!	#NULL!	#NULL!	#NULL!
2	#NULL!	#NULL!	#NULL!	#NULL!	#NULL!
99	#NULL!	#NULL!	#NULL!	#NULL!	#NULL!
3	#NULL!	#NULL!	#NULL!	#NULL!	#NULL!
99	#NULL!	#NULL!	#NULL!	#NULL!	#NULL!
3	#NULL!	#NULL!	#NULL!	#NULL!	#NULL!
99	#NULL!	#NULL!	#NULL!	#NULL!	#NULL!
3	#NULL!	#NULL!	#NULL!	#NULL!	#NULL!
2	#NULL!	#NULL!	#NULL!	#NULL!	#NULL!
3	#NULL!	#NULL!	#NULL!	#NULL!	#NULL!
2	#NULL!	#NULL!	#NULL!	#NULL!	#NULL!
3	#NULL!	#NULL!	#NULL!	#NULL!	#NULL!
4	#NULL!	#NULL!	#NULL!	#NULL!	#NULL!
99	#NULL!	#NULL!	#NULL!	#NULL!	#NULL!
3	#NULL!	#NULL!	#NULL!	#NULL!	#NULL!
4	#NULL!	#NULL!	#NULL!	#NULL!	#NULL!
3	#NULL!	#NULL!	#NULL!	#NULL!	#NULL!
3	#NULL!	#NULL!	#NULL!	#NULL!	#NULL!
3	#NULL!	#NULL!	#NULL!	#NULL!	#NULL!
4	#NULL!	#NULL!	#NULL!	#NULL!	#NULL!
4	#NULL!	#NULL!	#NULL!	#NULL!	#NULL!
2	#NULL!	#NULL!	#NULL!	#NULL!	#NULL!
2	#NULL!	#NULL!	#NULL!	#NULL!	#NULL!
4	#NULL!	#NULL!	#NULL!	#NULL!	#NULL!
3	#NULL!	#NULL!	#NULL!	#NULL!	#NULL!
4	#NULL!	#NULL!	#NULL!	#NULL!	#NULL!
99	#NULL!	#NULL!	#NULL!	#NULL!	#NULL!
1	#NULL!	#NULL!	#NULL!	#NULL!	#NULL!

3	#NULL!	#NULL!	#NULL!	#NULL!	#NULL!
4	#NULL!	#NULL!	#NULL!	#NULL!	#NULL!
3	#NULL!	#NULL!	#NULL!	#NULL!	#NULL!
3	#NULL!	#NULL!	#NULL!	#NULL!	#NULL!
3	#NULL!	#NULL!	#NULL!	#NULL!	#NULL!
4	#NULL!	#NULL!	#NULL!	#NULL!	#NULL!
4	#NULL!	#NULL!	#NULL!	#NULL!	#NULL!
3	#NULL!	#NULL!	#NULL!	#NULL!	#NULL!
3	#NULL!	#NULL!	#NULL!	#NULL!	#NULL!
4	#NULL!	#NULL!	#NULL!	#NULL!	#NULL!
3	#NULL!	#NULL!	#NULL!	#NULL!	#NULL!
3	#NULL!	#NULL!	#NULL!	#NULL!	#NULL!
3	#NULL!	#NULL!	#NULL!	#NULL!	#NULL!
4	#NULL!	#NULL!	#NULL!	#NULL!	#NULL!
3	#NULL!	#NULL!	#NULL!	#NULL!	#NULL!
3	#NULL!	#NULL!	#NULL!	#NULL!	#NULL!
4	#NULL!	#NULL!	#NULL!	#NULL!	#NULL!
99	#NULL!	#NULL!	#NULL!	#NULL!	#NULL!
3	#NULL!	#NULL!	#NULL!	#NULL!	#NULL!
3	#NULL!	#NULL!	#NULL!	#NULL!	#NULL!
1	#NULL!	#NULL!	#NULL!	#NULL!	#NULL!
3	#NULL!	#NULL!	#NULL!	#NULL!	#NULL!
1	#NULL!	#NULL!	#NULL!	#NULL!	#NULL!
3	#NULL!	#NULL!	#NULL!	#NULL!	#NULL!
4	#NULL!	#NULL!	#NULL!	#NULL!	#NULL!
1	#NULL!	#NULL!	#NULL!	#NULL!	#NULL!
4	#NULL!	#NULL!	#NULL!	#NULL!	#NULL!
4	#NULL!	#NULL!	#NULL!	#NULL!	#NULL!
3	#NULL!	#NULL!	#NULL!	#NULL!	#NULL!
3	#NULL!	#NULL!	#NULL!	#NULL!	#NULL!
3	#NULL!	#NULL!	#NULL!	#NULL!	#NULL!
4	#NULL!	#NULL!	#NULL!	#NULL!	#NULL!
3	#NULL!	#NULL!	#NULL!	#NULL!	#NULL!
3	#NULL!	#NULL!	#NULL!	#NULL!	#NULL!
3	#NULL!	#NULL!	#NULL!	#NULL!	#NULL!
4	#NULL!	#NULL!	#NULL!	#NULL!	#NULL!
4	#NULL!	#NULL!	#NULL!	#NULL!	#NULL!
4	#NULL!	#NULL!	#NULL!	#NULL!	#NULL!
99	#NULL!	#NULL!	#NULL!	#NULL!	#NULL!
3	#NULL!	#NULL!	#NULL!	#NULL!	#NULL!
4	#NULL!	#NULL!	#NULL!	#NULL!	#NULL!
3	#NULL!	#NULL!	#NULL!	#NULL!	#NULL!
4	#NULL!	#NULL!	#NULL!	#NULL!	#NULL!
3	#NULL!	#NULL!	#NULL!	#NULL!	#NULL!
2	#NULL!	#NULL!	#NULL!	#NULL!	#NULL!
3	#NULL!	#NULL!	#NULL!	#NULL!	#NULL!
4	#NULL!	#NULL!	#NULL!	#NULL!	#NULL!
2	#NULL!	#NULL!	#NULL!	#NULL!	#NULL!

3	#NULL!	#NULL!	#NULL!	#NULL!	#NULL!
1	#NULL!	#NULL!	#NULL!	#NULL!	#NULL!
3	#NULL!	#NULL!	#NULL!	#NULL!	#NULL!
3	#NULL!	#NULL!	#NULL!	#NULL!	#NULL!
4	#NULL!	#NULL!	#NULL!	#NULL!	#NULL!
3	#NULL!	#NULL!	#NULL!	#NULL!	#NULL!
3	#NULL!	#NULL!	#NULL!	#NULL!	#NULL!
3	#NULL!	#NULL!	#NULL!	#NULL!	#NULL!
2	#NULL!	#NULL!	#NULL!	#NULL!	#NULL!
4	#NULL!	#NULL!	#NULL!	#NULL!	#NULL!
2	#NULL!	#NULL!	#NULL!	#NULL!	#NULL!
4	#NULL!	#NULL!	#NULL!	#NULL!	#NULL!
3	#NULL!	#NULL!	#NULL!	#NULL!	#NULL!
3	#NULL!	#NULL!	#NULL!	#NULL!	#NULL!
2	#NULL!	#NULL!	#NULL!	#NULL!	#NULL!
1	#NULL!	#NULL!	#NULL!	#NULL!	#NULL!
3	#NULL!	#NULL!	#NULL!	#NULL!	#NULL!
2	#NULL!	#NULL!	#NULL!	#NULL!	#NULL!
3	#NULL!	#NULL!	#NULL!	#NULL!	#NULL!
3	#NULL!	#NULL!	#NULL!	#NULL!	#NULL!
3	#NULL!	#NULL!	#NULL!	#NULL!	#NULL!
3	#NULL!	#NULL!	#NULL!	#NULL!	#NULL!
4	#NULL!	#NULL!	#NULL!	#NULL!	#NULL!
2	#NULL!	#NULL!	#NULL!	#NULL!	#NULL!
4	#NULL!	#NULL!	#NULL!	#NULL!	#NULL!
3	#NULL!	#NULL!	#NULL!	#NULL!	#NULL!
3	#NULL!	#NULL!	#NULL!	#NULL!	#NULL!
3	#NULL!	#NULL!	#NULL!	#NULL!	#NULL!
1	#NULL!	#NULL!	#NULL!	#NULL!	#NULL!
3	#NULL!	#NULL!	#NULL!	#NULL!	#NULL!
2	#NULL!	#NULL!	#NULL!	#NULL!	#NULL!
4	#NULL!	#NULL!	#NULL!	#NULL!	#NULL!
4	#NULL!	#NULL!	#NULL!	#NULL!	#NULL!
3	#NULL!	#NULL!	#NULL!	#NULL!	#NULL!
4	#NULL!	#NULL!	#NULL!	#NULL!	#NULL!
3	#NULL!	#NULL!	#NULL!	#NULL!	#NULL!
3	#NULL!	#NULL!	#NULL!	#NULL!	#NULL!
4	#NULL!	#NULL!	#NULL!	#NULL!	#NULL!
3	#NULL!	#NULL!	#NULL!	#NULL!	#NULL!
4	#NULL!	#NULL!	#NULL!	#NULL!	#NULL!
3	#NULL!	#NULL!	#NULL!	#NULL!	#NULL!
99	#NULL!	#NULL!	#NULL!	#NULL!	#NULL!
4	#NULL!	#NULL!	#NULL!	#NULL!	#NULL!
3	#NULL!	#NULL!	#NULL!	#NULL!	#NULL!
3	#NULL!	#NULL!	#NULL!	#NULL!	#NULL!
4	#NULL!	#NULL!	#NULL!	#NULL!	#NULL!
2	#NULL!	#NULL!	#NULL!	#NULL!	#NULL!
2	#NULL!	#NULL!	#NULL!	#NULL!	#NULL!

4	#NULL!	#NULL!	#NULL!	#NULL!	#NULL!
2	#NULL!	#NULL!	#NULL!	#NULL!	#NULL!
4	#NULL!	#NULL!	#NULL!	#NULL!	#NULL!
2	#NULL!	#NULL!	#NULL!	#NULL!	#NULL!
3	#NULL!	#NULL!	#NULL!	#NULL!	#NULL!
3	#NULL!	#NULL!	#NULL!	#NULL!	#NULL!
3	#NULL!	#NULL!	#NULL!	#NULL!	#NULL!
1	#NULL!	#NULL!	#NULL!	#NULL!	#NULL!
4	#NULL!	#NULL!	#NULL!	#NULL!	#NULL!
3	#NULL!	#NULL!	#NULL!	#NULL!	#NULL!
3	#NULL!	#NULL!	#NULL!	#NULL!	#NULL!
3	#NULL!	#NULL!	#NULL!	#NULL!	#NULL!
4	#NULL!	#NULL!	#NULL!	#NULL!	#NULL!
3	#NULL!	#NULL!	#NULL!	#NULL!	#NULL!
3	#NULL!	#NULL!	#NULL!	#NULL!	#NULL!
4	#NULL!	#NULL!	#NULL!	#NULL!	#NULL!
4	#NULL!	#NULL!	#NULL!	#NULL!	#NULL!
3	#NULL!	#NULL!	#NULL!	#NULL!	#NULL!
3	#NULL!	#NULL!	#NULL!	#NULL!	#NULL!
3	#NULL!	#NULL!	#NULL!	#NULL!	#NULL!
4	#NULL!	#NULL!	#NULL!	#NULL!	#NULL!
4	#NULL!	#NULL!	#NULL!	#NULL!	#NULL!
3	#NULL!	#NULL!	#NULL!	#NULL!	#NULL!
4	#NULL!	#NULL!	#NULL!	#NULL!	#NULL!
3	#NULL!	#NULL!	#NULL!	#NULL!	#NULL!
3	#NULL!	#NULL!	#NULL!	#NULL!	#NULL!
3	#NULL!	#NULL!	#NULL!	#NULL!	#NULL!
2	#NULL!	#NULL!	#NULL!	#NULL!	#NULL!
3	#NULL!	#NULL!	#NULL!	#NULL!	#NULL!
3	#NULL!	#NULL!	#NULL!	#NULL!	#NULL!
4	#NULL!	#NULL!	#NULL!	#NULL!	#NULL!
4	#NULL!	#NULL!	#NULL!	#NULL!	#NULL!
4	#NULL!	#NULL!	#NULL!	#NULL!	#NULL!
3	#NULL!	#NULL!	#NULL!	#NULL!	#NULL!
4	#NULL!	#NULL!	#NULL!	#NULL!	#NULL!
3	#NULL!	#NULL!	#NULL!	#NULL!	#NULL!
3	#NULL!	#NULL!	#NULL!	#NULL!	#NULL!
4	#NULL!	#NULL!	#NULL!	#NULL!	#NULL!
99	#NULL!	#NULL!	#NULL!	#NULL!	#NULL!
3	#NULL!	#NULL!	#NULL!	#NULL!	#NULL!
3	#NULL!	#NULL!	#NULL!	#NULL!	#NULL!
3	#NULL!	#NULL!	#NULL!	#NULL!	#NULL!
4	#NULL!	#NULL!	#NULL!	#NULL!	#NULL!
2	#NULL!	#NULL!	#NULL!	#NULL!	#NULL!
3	#NULL!	#NULL!	#NULL!	#NULL!	#NULL!
3	#NULL!	#NULL!	#NULL!	#NULL!	#NULL!
4	#NULL!	#NULL!	#NULL!	#NULL!	#NULL!
3	#NULL!	#NULL!	#NULL!	#NULL!	#NULL!

3	#NULL!	#NULL!	#NULL!	#NULL!	#NULL!
2	#NULL!	#NULL!	#NULL!	#NULL!	#NULL!
3	#NULL!	#NULL!	#NULL!	#NULL!	#NULL!
4	#NULL!	#NULL!	#NULL!	#NULL!	#NULL!
3	#NULL!	#NULL!	#NULL!	#NULL!	#NULL!
3	#NULL!	#NULL!	#NULL!	#NULL!	#NULL!
3	#NULL!	#NULL!	#NULL!	#NULL!	#NULL!
1	#NULL!	#NULL!	#NULL!	#NULL!	#NULL!
3	#NULL!	#NULL!	#NULL!	#NULL!	#NULL!
4	#NULL!	#NULL!	#NULL!	#NULL!	#NULL!
3	#NULL!	#NULL!	#NULL!	#NULL!	#NULL!
3	#NULL!	#NULL!	#NULL!	#NULL!	#NULL!
3	#NULL!	#NULL!	#NULL!	#NULL!	#NULL!
3	#NULL!	#NULL!	#NULL!	#NULL!	#NULL!
3	#NULL!	#NULL!	#NULL!	#NULL!	#NULL!
1	#NULL!	#NULL!	#NULL!	#NULL!	#NULL!
3	#NULL!	#NULL!	#NULL!	#NULL!	#NULL!
3	#NULL!	#NULL!	#NULL!	#NULL!	#NULL!
99	#NULL!	#NULL!	#NULL!	#NULL!	#NULL!
4	#NULL!	#NULL!	#NULL!	#NULL!	#NULL!
3	#NULL!	#NULL!	#NULL!	#NULL!	#NULL!
3	#NULL!	#NULL!	#NULL!	#NULL!	#NULL!
3	#NULL!	#NULL!	#NULL!	#NULL!	#NULL!
4	#NULL!	#NULL!	#NULL!	#NULL!	#NULL!
4	#NULL!	#NULL!	#NULL!	#NULL!	#NULL!
3	#NULL!	#NULL!	#NULL!	#NULL!	#NULL!
3	#NULL!	#NULL!	#NULL!	#NULL!	#NULL!
4	#NULL!	#NULL!	#NULL!	#NULL!	#NULL!
4	#NULL!	#NULL!	#NULL!	#NULL!	#NULL!
4	#NULL!	#NULL!	#NULL!	#NULL!	#NULL!
1	#NULL!	#NULL!	#NULL!	#NULL!	#NULL!
2	#NULL!	#NULL!	#NULL!	#NULL!	#NULL!
4	#NULL!	#NULL!	#NULL!	#NULL!	#NULL!
4	#NULL!	#NULL!	#NULL!	#NULL!	#NULL!
4	#NULL!	#NULL!	#NULL!	#NULL!	#NULL!
4	#NULL!	#NULL!	#NULL!	#NULL!	#NULL!
4	#NULL!	#NULL!	#NULL!	#NULL!	#NULL!
4	#NULL!	#NULL!	#NULL!	#NULL!	#NULL!
4	#NULL!	#NULL!	#NULL!	#NULL!	#NULL!
3	#NULL!	#NULL!	#NULL!	#NULL!	#NULL!
4	#NULL!	#NULL!	#NULL!	#NULL!	#NULL!
3	#NULL!	#NULL!	#NULL!	#NULL!	#NULL!
2	#NULL!	#NULL!	#NULL!	#NULL!	#NULL!
4	#NULL!	#NULL!	#NULL!	#NULL!	#NULL!
1	#NULL!	#NULL!	#NULL!	#NULL!	#NULL!
1	#NULL!	#NULL!	#NULL!	#NULL!	#NULL!
4	#NULL!	#NULL!	#NULL!	#NULL!	#NULL!
2	#NULL!	#NULL!	#NULL!	#NULL!	#NULL!

3	#NULL!	#NULL!	#NULL!	#NULL!	#NULL!
3	#NULL!	#NULL!	#NULL!	#NULL!	#NULL!
4	#NULL!	#NULL!	#NULL!	#NULL!	#NULL!
4	#NULL!	#NULL!	#NULL!	#NULL!	#NULL!
3	#NULL!	#NULL!	#NULL!	#NULL!	#NULL!
4	#NULL!	#NULL!	#NULL!	#NULL!	#NULL!
3	#NULL!	#NULL!	#NULL!	#NULL!	#NULL!
3	#NULL!	#NULL!	#NULL!	#NULL!	#NULL!
3	#NULL!	#NULL!	#NULL!	#NULL!	#NULL!
2	#NULL!	#NULL!	#NULL!	#NULL!	#NULL!
99	#NULL!	#NULL!	#NULL!	#NULL!	#NULL!
3	#NULL!	#NULL!	#NULL!	#NULL!	#NULL!
3	#NULL!	#NULL!	#NULL!	#NULL!	#NULL!
3	#NULL!	#NULL!	#NULL!	#NULL!	#NULL!
2	#NULL!	#NULL!	#NULL!	#NULL!	#NULL!
2	#NULL!	#NULL!	#NULL!	#NULL!	#NULL!
3	#NULL!	#NULL!	#NULL!	#NULL!	#NULL!
4	#NULL!	#NULL!	#NULL!	#NULL!	#NULL!
4	#NULL!	#NULL!	#NULL!	#NULL!	#NULL!
3	#NULL!	#NULL!	#NULL!	#NULL!	#NULL!
3	#NULL!	#NULL!	#NULL!	#NULL!	#NULL!
4	#NULL!	#NULL!	#NULL!	#NULL!	#NULL!
1	#NULL!	#NULL!	#NULL!	#NULL!	#NULL!
3	#NULL!	#NULL!	#NULL!	#NULL!	#NULL!
4	#NULL!	#NULL!	#NULL!	#NULL!	#NULL!
2	#NULL!	#NULL!	#NULL!	#NULL!	#NULL!
3	#NULL!	#NULL!	#NULL!	#NULL!	#NULL!
1	#NULL!	#NULL!	#NULL!	#NULL!	#NULL!
3	#NULL!	#NULL!	#NULL!	#NULL!	#NULL!
1	#NULL!	#NULL!	#NULL!	#NULL!	#NULL!
1	#NULL!	#NULL!	#NULL!	#NULL!	#NULL!
1	#NULL!	#NULL!	#NULL!	#NULL!	#NULL!
4	#NULL!	#NULL!	#NULL!	#NULL!	#NULL!
2	#NULL!	#NULL!	#NULL!	#NULL!	#NULL!
3	#NULL!	#NULL!	#NULL!	#NULL!	#NULL!
3	#NULL!	#NULL!	#NULL!	#NULL!	#NULL!
3	#NULL!	#NULL!	#NULL!	#NULL!	#NULL!
3	#NULL!	#NULL!	#NULL!	#NULL!	#NULL!
4	#NULL!	#NULL!	#NULL!	#NULL!	#NULL!
4	#NULL!	#NULL!	#NULL!	#NULL!	#NULL!
3	#NULL!	#NULL!	#NULL!	#NULL!	#NULL!
2	#NULL!	#NULL!	#NULL!	#NULL!	#NULL!
3	#NULL!	#NULL!	#NULL!	#NULL!	#NULL!
4	#NULL!	#NULL!	#NULL!	#NULL!	#NULL!
3	#NULL!	#NULL!	#NULL!	#NULL!	#NULL!
2	#NULL!	#NULL!	#NULL!	#NULL!	#NULL!
3	#NULL!	#NULL!	#NULL!	#NULL!	#NULL!
2	#NULL!	#NULL!	#NULL!	#NULL!	#NULL!

4	#NULL!	#NULL!	#NULL!	#NULL!	#NULL!
3	#NULL!	#NULL!	#NULL!	#NULL!	#NULL!
2	#NULL!	#NULL!	#NULL!	#NULL!	#NULL!
3	#NULL!	#NULL!	#NULL!	#NULL!	#NULL!
4	#NULL!	#NULL!	#NULL!	#NULL!	#NULL!
3	#NULL!	#NULL!	#NULL!	#NULL!	#NULL!
3	#NULL!	#NULL!	#NULL!	#NULL!	#NULL!
3	#NULL!	#NULL!	#NULL!	#NULL!	#NULL!
3	#NULL!	#NULL!	#NULL!	#NULL!	#NULL!
4	#NULL!	#NULL!	#NULL!	#NULL!	#NULL!
2	#NULL!	#NULL!	#NULL!	#NULL!	#NULL!
2	#NULL!	#NULL!	#NULL!	#NULL!	#NULL!
4	#NULL!	#NULL!	#NULL!	#NULL!	#NULL!
3	#NULL!	#NULL!	#NULL!	#NULL!	#NULL!
1	#NULL!	#NULL!	#NULL!	#NULL!	#NULL!
4	#NULL!	#NULL!	#NULL!	#NULL!	#NULL!
2	#NULL!	#NULL!	#NULL!	#NULL!	#NULL!
2	#NULL!	#NULL!	#NULL!	#NULL!	#NULL!
1	#NULL!	#NULL!	#NULL!	#NULL!	#NULL!
1	#NULL!	#NULL!	#NULL!	#NULL!	#NULL!
4	#NULL!	#NULL!	#NULL!	#NULL!	#NULL!
3	#NULL!	#NULL!	#NULL!	#NULL!	#NULL!
4	#NULL!	#NULL!	#NULL!	#NULL!	#NULL!
1	#NULL!	#NULL!	#NULL!	#NULL!	#NULL!
2	#NULL!	#NULL!	#NULL!	#NULL!	#NULL!
3	#NULL!	#NULL!	#NULL!	#NULL!	#NULL!
3	#NULL!	#NULL!	#NULL!	#NULL!	#NULL!
4	#NULL!	#NULL!	#NULL!	#NULL!	#NULL!
2	#NULL!	#NULL!	#NULL!	#NULL!	#NULL!
4	#NULL!	#NULL!	#NULL!	#NULL!	#NULL!
2	#NULL!	#NULL!	#NULL!	#NULL!	#NULL!
4	#NULL!	#NULL!	#NULL!	#NULL!	#NULL!
4	#NULL!	#NULL!	#NULL!	#NULL!	#NULL!
3	#NULL!	#NULL!	#NULL!	#NULL!	#NULL!
3	#NULL!	#NULL!	#NULL!	#NULL!	#NULL!
1	#NULL!	#NULL!	#NULL!	#NULL!	#NULL!
4	#NULL!	#NULL!	#NULL!	#NULL!	#NULL!
2	#NULL!	#NULL!	#NULL!	#NULL!	#NULL!
3	#NULL!	#NULL!	#NULL!	#NULL!	#NULL!
4	#NULL!	#NULL!	#NULL!	#NULL!	#NULL!
3	#NULL!	#NULL!	#NULL!	#NULL!	#NULL!
3	#NULL!	#NULL!	#NULL!	#NULL!	#NULL!
4	#NULL!	#NULL!	#NULL!	#NULL!	#NULL!
4	#NULL!	#NULL!	#NULL!	#NULL!	#NULL!
3	#NULL!	#NULL!	#NULL!	#NULL!	#NULL!
4	#NULL!	#NULL!	#NULL!	#NULL!	#NULL!
2	#NULL!	#NULL!	#NULL!	#NULL!	#NULL!
2	#NULL!	#NULL!	#NULL!	#NULL!	#NULL!

3	#NULL!	#NULL!	#NULL!	#NULL!	#NULL!
3	#NULL!	#NULL!	#NULL!	#NULL!	#NULL!
4	#NULL!	#NULL!	#NULL!	#NULL!	#NULL!
4	#NULL!	#NULL!	#NULL!	#NULL!	#NULL!
4	#NULL!	#NULL!	#NULL!	#NULL!	#NULL!
3	#NULL!	#NULL!	#NULL!	#NULL!	#NULL!
1	#NULL!	#NULL!	#NULL!	#NULL!	#NULL!
3	#NULL!	#NULL!	#NULL!	#NULL!	#NULL!
3	#NULL!	#NULL!	#NULL!	#NULL!	#NULL!
4	#NULL!	#NULL!	#NULL!	#NULL!	#NULL!
2	#NULL!	#NULL!	#NULL!	#NULL!	#NULL!
3	#NULL!	#NULL!	#NULL!	#NULL!	#NULL!
4	#NULL!	#NULL!	#NULL!	#NULL!	#NULL!
3	#NULL!	#NULL!	#NULL!	#NULL!	#NULL!
99	#NULL!	#NULL!	#NULL!	#NULL!	#NULL!
1	#NULL!	#NULL!	#NULL!	#NULL!	#NULL!
2	#NULL!	#NULL!	#NULL!	#NULL!	#NULL!
2	#NULL!	#NULL!	#NULL!	#NULL!	#NULL!
4	#NULL!	#NULL!	#NULL!	#NULL!	#NULL!
3	#NULL!	#NULL!	#NULL!	#NULL!	#NULL!
4	#NULL!	#NULL!	#NULL!	#NULL!	#NULL!
4	#NULL!	#NULL!	#NULL!	#NULL!	#NULL!
2	#NULL!	#NULL!	#NULL!	#NULL!	#NULL!
3	#NULL!	#NULL!	#NULL!	#NULL!	#NULL!
2	#NULL!	#NULL!	#NULL!	#NULL!	#NULL!
3	#NULL!	#NULL!	#NULL!	#NULL!	#NULL!
3	#NULL!	#NULL!	#NULL!	#NULL!	#NULL!
4	#NULL!	#NULL!	#NULL!	#NULL!	#NULL!
1	#NULL!	#NULL!	#NULL!	#NULL!	#NULL!
3	#NULL!	#NULL!	#NULL!	#NULL!	#NULL!
4	#NULL!	#NULL!	#NULL!	#NULL!	#NULL!
2	#NULL!	#NULL!	#NULL!	#NULL!	#NULL!
2	#NULL!	#NULL!	#NULL!	#NULL!	#NULL!
3	#NULL!	#NULL!	#NULL!	#NULL!	#NULL!
3	#NULL!	#NULL!	#NULL!	#NULL!	#NULL!
3	#NULL!	#NULL!	#NULL!	#NULL!	#NULL!
3	#NULL!	#NULL!	#NULL!	#NULL!	#NULL!
3	#NULL!	#NULL!	#NULL!	#NULL!	#NULL!
4	#NULL!	#NULL!	#NULL!	#NULL!	#NULL!
3	#NULL!	#NULL!	#NULL!	#NULL!	#NULL!
4	#NULL!	#NULL!	#NULL!	#NULL!	#NULL!
3	#NULL!	#NULL!	#NULL!	#NULL!	#NULL!
4	#NULL!	#NULL!	#NULL!	#NULL!	#NULL!
2	#NULL!	#NULL!	#NULL!	#NULL!	#NULL!
3	#NULL!	#NULL!	#NULL!	#NULL!	#NULL!
1	#NULL!	#NULL!	#NULL!	#NULL!	#NULL!
4	#NULL!	#NULL!	#NULL!	#NULL!	#NULL!
4	#NULL!	#NULL!	#NULL!	#NULL!	#NULL!

3	#NULL!	#NULL!	#NULL!	#NULL!	#NULL!
4	#NULL!	#NULL!	#NULL!	#NULL!	#NULL!
3	#NULL!	#NULL!	#NULL!	#NULL!	#NULL!
4	#NULL!	#NULL!	#NULL!	#NULL!	#NULL!
3	#NULL!	#NULL!	#NULL!	#NULL!	#NULL!
3	#NULL!	#NULL!	#NULL!	#NULL!	#NULL!
99	#NULL!	#NULL!	#NULL!	#NULL!	#NULL!
3	#NULL!	#NULL!	#NULL!	#NULL!	#NULL!
4	#NULL!	#NULL!	#NULL!	#NULL!	#NULL!
4	#NULL!	#NULL!	#NULL!	#NULL!	#NULL!
4	#NULL!	#NULL!	#NULL!	#NULL!	#NULL!
4	#NULL!	#NULL!	#NULL!	#NULL!	#NULL!
4	#NULL!	#NULL!	#NULL!	#NULL!	#NULL!
3	#NULL!	#NULL!	#NULL!	#NULL!	#NULL!
3	#NULL!	#NULL!	#NULL!	#NULL!	#NULL!
2	#NULL!	#NULL!	#NULL!	#NULL!	#NULL!
3	#NULL!	#NULL!	#NULL!	#NULL!	#NULL!
3	#NULL!	#NULL!	#NULL!	#NULL!	#NULL!
2	#NULL!	#NULL!	#NULL!	#NULL!	#NULL!
2	#NULL!	#NULL!	#NULL!	#NULL!	#NULL!
3	#NULL!	#NULL!	#NULL!	#NULL!	#NULL!
1	#NULL!	#NULL!	#NULL!	#NULL!	#NULL!
3	#NULL!	#NULL!	#NULL!	#NULL!	#NULL!
3	#NULL!	#NULL!	#NULL!	#NULL!	#NULL!
3	#NULL!	#NULL!	#NULL!	#NULL!	#NULL!
3	#NULL!	#NULL!	#NULL!	#NULL!	#NULL!
3	#NULL!	#NULL!	#NULL!	#NULL!	#NULL!
3	#NULL!	#NULL!	#NULL!	#NULL!	#NULL!
3	#NULL!	#NULL!	#NULL!	#NULL!	#NULL!
1	#NULL!	#NULL!	#NULL!	#NULL!	#NULL!
2	#NULL!	#NULL!	#NULL!	#NULL!	#NULL!
3	#NULL!	#NULL!	#NULL!	#NULL!	#NULL!
3	#NULL!	#NULL!	#NULL!	#NULL!	#NULL!
1	#NULL!	#NULL!	#NULL!	#NULL!	#NULL!
3	#NULL!	#NULL!	#NULL!	#NULL!	#NULL!
4	#NULL!	#NULL!	#NULL!	#NULL!	#NULL!
3	#NULL!	#NULL!	#NULL!	#NULL!	#NULL!
3	#NULL!	#NULL!	#NULL!	#NULL!	#NULL!
3	#NULL!	#NULL!	#NULL!	#NULL!	#NULL!
3	#NULL!	#NULL!	#NULL!	#NULL!	#NULL!
3	#NULL!	#NULL!	#NULL!	#NULL!	#NULL!
2	#NULL!	#NULL!	#NULL!	#NULL!	#NULL!
3	#NULL!	#NULL!	#NULL!	#NULL!	#NULL!
3	#NULL!	#NULL!	#NULL!	#NULL!	#NULL!
2	#NULL!	#NULL!	#NULL!	#NULL!	#NULL!
4	#NULL!	#NULL!	#NULL!	#NULL!	#NULL!
3	#NULL!	#NULL!	#NULL!	#NULL!	#NULL!
2	#NULL!	#NULL!	#NULL!	#NULL!	#NULL!

3	#NULL!	#NULL!	#NULL!	#NULL!	#NULL!
2	#NULL!	#NULL!	#NULL!	#NULL!	#NULL!
3	#NULL!	#NULL!	#NULL!	#NULL!	#NULL!
3	#NULL!	#NULL!	#NULL!	#NULL!	#NULL!
4	#NULL!	#NULL!	#NULL!	#NULL!	#NULL!
2	#NULL!	#NULL!	#NULL!	#NULL!	#NULL!
3	#NULL!	#NULL!	#NULL!	#NULL!	#NULL!
4	#NULL!	#NULL!	#NULL!	#NULL!	#NULL!
2	#NULL!	#NULL!	#NULL!	#NULL!	#NULL!
4	#NULL!	#NULL!	#NULL!	#NULL!	#NULL!
3	#NULL!	#NULL!	#NULL!	#NULL!	#NULL!
1	#NULL!	#NULL!	#NULL!	#NULL!	#NULL!
2	#NULL!	#NULL!	#NULL!	#NULL!	#NULL!
2	#NULL!	#NULL!	#NULL!	#NULL!	#NULL!
3	#NULL!	#NULL!	#NULL!	#NULL!	#NULL!
3	#NULL!	#NULL!	#NULL!	#NULL!	#NULL!
4	#NULL!	#NULL!	#NULL!	#NULL!	#NULL!
3	#NULL!	#NULL!	#NULL!	#NULL!	#NULL!
4	#NULL!	#NULL!	#NULL!	#NULL!	#NULL!
3	#NULL!	#NULL!	#NULL!	#NULL!	#NULL!
2	#NULL!	#NULL!	#NULL!	#NULL!	#NULL!
2	#NULL!	#NULL!	#NULL!	#NULL!	#NULL!
3	#NULL!	#NULL!	#NULL!	#NULL!	#NULL!
4	#NULL!	#NULL!	#NULL!	#NULL!	#NULL!
4	#NULL!	#NULL!	#NULL!	#NULL!	#NULL!
2	#NULL!	#NULL!	#NULL!	#NULL!	#NULL!
3	#NULL!	#NULL!	#NULL!	#NULL!	#NULL!
3	#NULL!	#NULL!	#NULL!	#NULL!	#NULL!
4	#NULL!	#NULL!	#NULL!	#NULL!	#NULL!
4	#NULL!	#NULL!	#NULL!	#NULL!	#NULL!
3	#NULL!	#NULL!	#NULL!	#NULL!	#NULL!
2	#NULL!	#NULL!	#NULL!	#NULL!	#NULL!
3	#NULL!	#NULL!	#NULL!	#NULL!	#NULL!
1	#NULL!	#NULL!	#NULL!	#NULL!	#NULL!
3	#NULL!	#NULL!	#NULL!	#NULL!	#NULL!
4	#NULL!	#NULL!	#NULL!	#NULL!	#NULL!
4	#NULL!	#NULL!	#NULL!	#NULL!	#NULL!
3	#NULL!	#NULL!	#NULL!	#NULL!	#NULL!
4	#NULL!	#NULL!	#NULL!	#NULL!	#NULL!
3	#NULL!	#NULL!	#NULL!	#NULL!	#NULL!
2	#NULL!	#NULL!	#NULL!	#NULL!	#NULL!
3	#NULL!	#NULL!	#NULL!	#NULL!	#NULL!
3	#NULL!	#NULL!	#NULL!	#NULL!	#NULL!
2	#NULL!	#NULL!	#NULL!	#NULL!	#NULL!
3	#NULL!	#NULL!	#NULL!	#NULL!	#NULL!
4	#NULL!	#NULL!	#NULL!	#NULL!	#NULL!
3	#NULL!	#NULL!	#NULL!	#NULL!	#NULL!
2	#NULL!	#NULL!	#NULL!	#NULL!	#NULL!

3	#NULL!	#NULL!	#NULL!	#NULL!	#NULL!
3	#NULL!	#NULL!	#NULL!	#NULL!	#NULL!
3	#NULL!	#NULL!	#NULL!	#NULL!	#NULL!
3	#NULL!	#NULL!	#NULL!	#NULL!	#NULL!
1	#NULL!	#NULL!	#NULL!	#NULL!	#NULL!
2	#NULL!	#NULL!	#NULL!	#NULL!	#NULL!
3	#NULL!	#NULL!	#NULL!	#NULL!	#NULL!
3	#NULL!	#NULL!	#NULL!	#NULL!	#NULL!
3	#NULL!	#NULL!	#NULL!	#NULL!	#NULL!
3	#NULL!	#NULL!	#NULL!	#NULL!	#NULL!
4	#NULL!	#NULL!	#NULL!	#NULL!	#NULL!
1	#NULL!	#NULL!	#NULL!	#NULL!	#NULL!
4	#NULL!	#NULL!	#NULL!	#NULL!	#NULL!
3	#NULL!	#NULL!	#NULL!	#NULL!	#NULL!
2	#NULL!	#NULL!	#NULL!	#NULL!	#NULL!
3	#NULL!	#NULL!	#NULL!	#NULL!	#NULL!
1	#NULL!	#NULL!	#NULL!	#NULL!	#NULL!
4	#NULL!	#NULL!	#NULL!	#NULL!	#NULL!
99	#NULL!	#NULL!	#NULL!	#NULL!	#NULL!
4	#NULL!	#NULL!	#NULL!	#NULL!	#NULL!
4	#NULL!	#NULL!	#NULL!	#NULL!	#NULL!
4	#NULL!	#NULL!	#NULL!	#NULL!	#NULL!
3	#NULL!	#NULL!	#NULL!	#NULL!	#NULL!
3	#NULL!	#NULL!	#NULL!	#NULL!	#NULL!
3	#NULL!	#NULL!	#NULL!	#NULL!	#NULL!
2	#NULL!	#NULL!	#NULL!	#NULL!	#NULL!
4	#NULL!	#NULL!	#NULL!	#NULL!	#NULL!
3	#NULL!	#NULL!	#NULL!	#NULL!	#NULL!
3	#NULL!	#NULL!	#NULL!	#NULL!	#NULL!
3	#NULL!	#NULL!	#NULL!	#NULL!	#NULL!
3	#NULL!	#NULL!	#NULL!	#NULL!	#NULL!
3	#NULL!	#NULL!	#NULL!	#NULL!	#NULL!
3	#NULL!	#NULL!	#NULL!	#NULL!	#NULL!
3	#NULL!	#NULL!	#NULL!	#NULL!	#NULL!
3	#NULL!	#NULL!	#NULL!	#NULL!	#NULL!
4	#NULL!	#NULL!	#NULL!	#NULL!	#NULL!
3	#NULL!	#NULL!	#NULL!	#NULL!	#NULL!
3	#NULL!	#NULL!	#NULL!	#NULL!	#NULL!
2	#NULL!	#NULL!	#NULL!	#NULL!	#NULL!
4	#NULL!	#NULL!	#NULL!	#NULL!	#NULL!
4	#NULL!	#NULL!	#NULL!	#NULL!	#NULL!
4	#NULL!	#NULL!	#NULL!	#NULL!	#NULL!
3	#NULL!	#NULL!	#NULL!	#NULL!	#NULL!
3	#NULL!	#NULL!	#NULL!	#NULL!	#NULL!
3	#NULL!	#NULL!	#NULL!	#NULL!	#NULL!
3	#NULL!	#NULL!	#NULL!	#NULL!	#NULL!
4	#NULL!	#NULL!	#NULL!	#NULL!	#NULL!
4	#NULL!	#NULL!	#NULL!	#NULL!	#NULL!

3	#NULL!	#NULL!	#NULL!	#NULL!	#NULL!
3	#NULL!	#NULL!	#NULL!	#NULL!	#NULL!
1	#NULL!	#NULL!	#NULL!	#NULL!	#NULL!
2	2	2	2	2	2
3	1	1	0	0	1
4	2	1	0	0	1
4	2	2	2	2	2
2	1	0	0	0	1
2	1	0	0	0	1
2	1	0	0	0	1
3	1	0	0	0	2
3	1	1	0	0	1
4	1	1	0	0	1
3	1	0	0	0	1
2	1	0	0	0	1
3	1	0	0	0	1
3	2	0	0	2	1
3	1	0	0	0	1
4	1	0	0	0	1
3	1	0	0	0	1
4	2	1	0	0	1
4	2	0	0	0	0
1	2	2	1	0	2
1	2	2	2	2	2
3	2	0	0	0	2
3	2	0	0	2	2
3	1	0	0	0	0
3	2	2	0	2	2
3	2	0	0	0	1
3	2	2	2	0	2
3	2	2	0	2	2
3	1	0	1	0	1
3	1	1	0	0	1
2	1	0	0	0	1
4	1	0	0	0	1
3	2	1	0	0	1
4	2	2	2	2	2
3	1	0	0	0	1
4	2	2	2	2	1
3	2	2	2	2	2
4	1	1	1	1	1
3	2	0	2	0	2
1	2	0	0	0	1
3	0	0	2	1	2
2	0	0	0	0	1
4	0	0	0	0	1
2	2	2	0	0	1
3	2	1	2	0	2

3	2	0	0	1	1
3	2	0	0	0	1
2	2	2	0	1	2
4	2	0	0	2	2
3	2	1	0	0	0
3	0	2	2	2	2
3	2	0	0	0	1
4	0	0	0	0	0
4	0	2	0	0	2
4	2	1	0	0	0
4	0	0	0	1	0
4	1	1	1	1	1
#NULL!	#NULL!	#NULL!	#NULL!	#NULL!	#NULL!
3	2	2	2	0	2
2	1	0	2	0	2
3	0	0	0	0	2
2	2	2	2	2	2
2	2	2	2	2	2
3	2	2	2	2	2
4	2	2	0	2	2
3	1	0	0	0	1
1	2	2	2	2	2
2	0	0	0	0	0
4	2	2	2	2	2
1	2	2	0	0	2
3	2	2	2	2	2
3	0	1	0	0	1
3	1	2	2	2	2
2	2	2	2	2	2
4	2	2	2	2	2
3	1	2	2	2	2
3	0	0	2	2	2
4	2	0	2	0	1
3	0	2	2	2	2
3	1	0	2	0	2
3	0	0	2	2	2
3	2	2	2	2	2
3	2	2	2	0	2
4	2	2	2	2	2
4	2	2	2	2	2
4	1	0	2	0	2
4	2	2	2	2	2
3	2	2	2	2	2
3	2	2	2	0	2
3	1	1	2	2	0
3	2	2	0	0	2
4	1	1	0	0	1
4	1	1	0	0	1

3	1	1	0	0	1
4	1	1	0	0	1
3	1	1	0	0	1
3	1	1	0	0	1
4	1	1	0	0	1
3	1	1	0	0	1
4	1	1	0	0	1
3	1	1	0	0	1
3	1	1	0	0	1
4	1	0	0	0	1
#NULL!	1	1	0	0	1
4	1	1	0	0	1
4	1	2	0	0	1
3	1	0	0	0	2
3	1	0	0	0	1
3	2	0	0	0	1
#NULL!	#NULL!	#NULL!	#NULL!	#NULL!	#NULL!
3	1	0	0	0	1
3	1	1	0	0	1
3	1	2	0	0	1
4	1	0	0	0	1

dtlrtl_presurvey_complete
2
2
2
2
2
2
2
2
2
2
2
2
2
2
2
2
2
2
2
2
2
2
2
2
2
2
2
2
2
2
2
2
2
2
2
2
2
2
2
2
2
2
2
2
2
2
2

2
2
2
2
2
2
2
2
2
2
2
2
2
2
2
2
2
2
2
2
2
2
2
2
2
2
2
2
2
2
2
2
2
2
2
2
2
2
2
2
2
2
2
2
2
2
2
2

2
2
2
2
2
2
2
2
2
2
2
2
2
2
2
2
2
2
2
2
2
2
2
2
2
2
2
2
2
2
2
2
2
2
2
2
2
2
2
2
2
2
2
2
2
2
2
2

2
2
2
2
2
2
2
2
2
2
2
2
2
2
2
2
2
2
2
2
2
2
2
2
2
2
2
2
2
2
2
2
2
2
2
2
2
2
2
2
2
2
2
2
2
2
2
2

2
2
2
2
2
2
2
2
2
2
2
2
2
2
2
2
2
2
2
2
2
2
2
2
2
2
2
2
2
2
2
2
2
2
2
2
2
2
2
2
2
2
2
2
2
2
2
2

2
2
2
2
2
2
2
2
2
2
2
2
2
2
2
2
2
2
2
2
2
2
2
2
2
2
2
2
2
2
2
2
2
2
2
2
2
2
2
2
2
2
2
2
2
2
2
2

2
2
2
2
2
2
2
2
2
2
2
2
2
2
2
2
2
2
2
2
2
2
2
2
2
2
2
2
2
2
2
2
2
2
2
2
2
2
2
2
2
2
2
2
2
2
2
2

2
2
2
2
2
2
2
2
2
2
2
2
2
2
2
2
2
2
2
2
2
2
2
2
2
2
2
2
2
2
2
2
2
2
2
2
2
2
2
2
2
2
2
2
2
2
2
2

2
2
2
2
2
2
2
2
2
2
2
2
2
2
2
2
2
2
2
2
2
2
2
2
2
2
2
2
2
2
2
2
2
2
2
2
2
2
2
2
2
2
2
2
2
2
2
2

2
2
2
2
2
2
2
2
2
2
2
2
2
2
2
2
2
2
2
2
2
2
2
2
2
2
2
2
2
2
2
2
2
2
2
2
2
2
2
2
2
2
2
2
2
2
2
2

2
2
2
2
2
2
2
2
2
2
2
2
2
2
2
2
2
2
2
2
2
2
2
2
2
2
2
2
2
2
2
2
2
2
2
2
2
2
2
2
2
2
2
2
2
2
2
2

2
2
2
2
2
2
2
2
2
2
2
2
2
2
2
2
2
2
2
2
2
2
2
2
2
2
2
2
2
2
2
2
2
2
2
2
2
2
2
2
2
2
2
2
2
2
2
2

2
2
2
2
2
2
2
2
2
2
2
2
2
2
2
2
2
2
2
2
2
2
2
2
2
2
2
2
2
2
2
2
2
2
2
2
2
2
2
2
2
2
2
2
2
2
2
2

2
2
2
2
2
2
2
2
2
2
2
2
2
2
2
2
2
2
2
2
2
2
2
2
2
2
2
2
2
2
2
2
2
2
2
2
2
2
2
2
2
2
2
2
2
2
2
2

2
2
2
2
2
2
2
2
2
2
2
2
2
2
2
2
2
2
2
2
2
2
2
2
2
2
2
2
2
2
2
2
2
2
2
2
2
2
2
2
2
2
2
2
2
2
2
2

2
2
2
2
2
2
2
2
2
2
2
2
2
2
2
2
2
2
2
2
2
2
2
2
2
2
2
2
2
2
2
2
2
2
2
2
2
2
2
2
2
2
2
2
2
2
2
2

2
2
2
2
2
2
2
2
2
2
2
2
2
2
2
2
2
2
2
2
2
2
2
2
2
2
2
2
2
2
2
2
2
2
2
2
2
2
2
2
2
2
2
2
2
2
2
2

2
2
2
2
2
2
2
2
2
2
2
2
2
2
2
2
2
2
2
2
2
2
2
2
2
2
2
2
2
2
2
2
2
2
2
2
2
2
2
2
2
2
2
2
2
2
2
2

2
2
2
2
2
2
2
2
2
2
2
2
2
2
2
2
2
2
2
2
2
2
2
2
2
2
2
2
2
2
2
2
2
2
2
2
2
2
2
2
2
2
2
2
2
2
2
2

2
2
2
2
2
2
2
2
2
2
2
2
2
2
2
2
2
2
2
2
2
2
2
2
2
2
2
2
2
2
2
2
2
2
2
2
2
2
2
2
2
2
2
2
2
2
2
2

2
2
2
2
2
2
2
2
2
2
2
2
2
2
2
2
2
2
2
2
2
2
2
2
2
2
2
2
2
2
2
2
2
2
2
2
2
2
2
2
2
2
2
2
2
2
2
2

2
2
2
2
2
2
2
2
2
2
2
2
2
2
2
2
2
2
2
2
2
2
2
2
2
2
2
2
2
2
2
2
2
2
2
2
2
2
2
2
2
2
2
2
2
2
2
2

2
2
2
2
2
2
2
2
2
2
2
2
2
2
2
2
2
2
2
2
2
2
2
2
2
2
2
2
2
2
2
2
2
2
2
2
2
2
2
2
2
2
2
2
2
2
2
2

2
2
2
2
2
2
2
2
2
2
2
2
2
2
2
2
2
2
2
2
2
2
2
2
2
2
2
2
2
2
2
2
2
2
2
2
2
2
2
2
2
2
2
2
2
2
2
2

2
2
2
2
2
2
2
2
2
2
2
2
2
2
2
2
2
2
2
2
2
2
2
2
2
2
2
2
2
2
2
2
2
2
2
2
2
2
2
2
2
2
2
2
2
2
2
2

2
2
2
2
2
2
2
2
2
2
2
2
2
2
2
2
2
2
2
2
2
2
2
2
2
2
2
2
2
2
2
2
2
2
2
2
2
2
2
2
2
2
2
2
2
2
2
2

2
2
2
2
2
2
2
2
2
2
2
2
2
2
2
2
2
2
2
2
2
2
2
2
2
2
2
2
2
2
2
2
2
2
2
2
2
2
2
2
2
2
2
2
2
2
2
2

2
2
2
2
2
2
2
2
2
2
2
2
2
2
2
2
2
2
2
2
2
2
2
2
2
2
2
2
2
2
2
2
2
2
2
2
2
2
2
2
2
2
2
2
2
2
2
2

2
2
2
2
2
2
2
2
2
2
2
2
2
2
2
2
2
2
2
2
2
2
2
2
2
2
2
2
2
2
2
2
2
2
2
2
2
2
2
2
2
2
2
2
2
2
2
2

2
2
2
2
2
2
2
2
2
2
2
2
2
2
2
2
2
2
2
2
2
2
2
2
2
2
2
2
2
2
2
2
2
2
2
2
2
2
2
2
2
2
2
2
2
2
2
2

2
2
2
2
2
2
2
2
2
2
2
2
2
2
2
2
2
2
2
2
2
2
2
2
2
2
2
2
2
2
2
2
2
2
2
2
2
2
2
2
2
2
2
2
2
2
2
2

2
2
2
2
2
2
2
2
2
2
2
2
2
2
2
2
1
2
2
2
2
2
2
2
2
2
2
2
2
2
2
2
2
2
2
2
2
2
2
2
2
2
2
2
2
2
2
2

2
2
2
2
2
2
2
2
2
2
2
2
2
2
2
2
2
2
2
2
2
2
2
2
2
2
2
2
2
2
2
2
2
2
2
2
2
2
2
2
2
2
2
2
2
2
2
2

2
2
2
2
2
2
2
2
2
2
2
2
2
2
2
2
2
2
2
2
2
2
2
2
2
2
2
2
2
2
2
2
2
2
2
2
2
2
2
2
2
2
2
2
2
2
2
2

2
2
2
2
2
2
2
2
2
2
2
2
2
2
2
2
2
2
2
2
2
2
2
2
2
2
2
2
2
2
2
2
2
2
2
2
2
2
2
2
2
2
2
2
2
2
2
2

2
2
2
2
2
2
2
2
2
2
2
2
2
2
2
2
2
2
2
2
2
2
2
2
2
2
2
2
2
2
2
2
2
2
2
2
2
2
2
2
2
2
2
2
2
2
2
2

2
2
2
2
2
2
2
2
2
2
2
2
2
2
2
2
2
2
2
2
2
2
2
2
2
2
2
2
2
2
2
2
2
2
2
2
2
2
2
2
2
2
2
2
2
2
2
2

2
2
2
2
2
2
2
2
2
2
2
2
2
2
2
2
2
2
2
2
2
2
2
2
2
2
2
2
2
2
2
2
2
2
2
2
2
2
2
2
2
2
2
2
2
2
2
2

2
2
2
2
2
2
2
2
2
2
2
2
2
2
2
2
2
2
2
2
2
2
2
2
2
2
2
2
2
2
2
2
2
2
2
2
2
2
2
2
2
2
2
2
2
2
2
2

2
2
2
2
2
2
2
2
2
2
2
2
2
2
2
2
2
2
2
2
2
2
2
2
2
2
2
2
2
2
2
2
2
2
2
2
2
2
2
2
2
2
2
2
2
2
2
2

1
1
1
2
2
2
2
2
2
2
2
2
2
2
2
2
2
2
2
2
2
2
2
2
2
2
2
2
2
2
2
2
2
2
2
2
2
2
2
2
2
2
2
2
2
2
2
2

2
2
2
2
2
2
2
2
2
2
2
2
2
2
2
2
2
2
2
2
2
2
2
2
2
2
2
2
2
2
2
2
2
2
2
2
2
2
2
2
2
2
2
2
2
2
2
2

2
2
2
2
2
2
2
2
2
2
2
2
2
2
2
2
2
2
2
2
2
2
2
2
2
2
2
2
2
2
2
2
2
2
2
2
2
2
2
2
2
2
2
2
2
2
2
2

2
2
2
2
2
2
2
2
2
2
2
2
2
2
2
2
2
2
2
2
2
2
2
2
2
2
2
2
2
2
2
2
2
2
2
2
2
2
2
2
2
2
2
2
2
2
2
2

2
2
2
2
2
2
2
2
2
2
2
2
2
2
2
2
2
2
2
2
2
2
2
2
2
2
2
2
2
2
2
2
2
2
2
2
2
2
2
2
2
2
2
2
2
2
2
2

2
2
2
2
2
2
2
2
2
2
2
2
2
2
2
2
2
2
2
2
2
2
2
2
2
2
2
2
2
2
2
2
2
2
2
2
2
2
2
2
2
2
2
2
2
2
2
2

2
2
2
2
2
2
2
2
2
2
2
2
2
2
2
2
2
2
2
2
2
2
2
2
2
2
2
2
2
2
2
2
2
2
2
2
2
2
2
2
2
2
2
2
2
2
2
2

2
2
2
2
2
2
2
2
2
2
2
2
2
2
2
2
2
2
2
2
2
2
2
2
2
2
2
2
2
2
2
2
2
2
2
2
2
2
2
2
2
2
2
2
2
2
2
2

2
2
2
2
2
2
2
2
2
2
2
2
2
2
2
2
2
2
2
2
2
2
2
2
2
2
2
2
2
2
2
2
2
2
2
2
2
2
2
2
2
2
2
2
2
2
2
2

2
2
2
2
2
2
2
2
2
2
2
2
2
2
2
2
2
2
2
2
2
2
2
2
2
2
2
2
2
2
2
2
2
2
2
2
2
2
2
2
2
2
2
2
2
2
2
2

2
2
2
2
2
2
2
2
2
2
2
2
2
2
2
2
2
2
2
2
2
2
2
2
2
2
2
2
2
2
2
2
2
2
2
2
2
2
2
2
2
2
2
2
2
2
2
2

2
2
2
2
2
2
2
2
2
2
2
2
2
2
2
2
2
2
2
2
2
2
2
2
2
2
2
2
2
2
2
2
2
2
2
2
2
2
2
2
2
2
2
2
2
2
2
2

2
2
2
2
2
2
2
2
2
2
2
2
2
2
2
2
2
2
2
2
2
2
2
2
2
2
2
2
2
2
2
2
2
2
2
2
2
2
2
2
2
2
2
2
2
2
2
2

2
2
2
2
2
2
2
2
2
2
2
2
2
2
2
2
2
2
2
2
2
2
2
2
2
2
2
2
2
2
2
2
2
2
2
2
2
2
2
2
2
2
2
2
2
2
2
2

2
2
2
2
2
2
2
2
2
2
2
2
2
2
2
2
2
2
2
2
2
2
2
2
2
2
2
2
2
0
2
2
2
2
2
2
2
2
2
2
2
2
2
2
2
2
2
2

2
2
2
2
2
2
2
2
0
2
0
2
2
0
2
2
2
2
2
0
2
2
2
2
0
2
2
2
2
0
2
2
2
2
2
0
2
2
2
2
2
2
2
2
2
2
2
2

2
2
2
0
2
2
2
2
2
0
2
2
2
2
2
2
0
2
2
2
2
